# Insights into isoprene production using the cyanobacterium *Synechocystis* sp. PCC 6803

**DOI:** 10.1186/s13068-016-0503-4

**Published:** 2016-04-18

**Authors:** Nadin Pade, Sabrina Erdmann, Heike Enke, Frederik Dethloff, Ulf Dühring, Jens Georg, Juliane Wambutt, Joachim Kopka, Wolfgang R. Hess, Ralf Zimmermann, Dan Kramer, Martin Hagemann

**Affiliations:** Plant Physiology Department, Institute of Biological Science, University of Rostock, Albert-Einstein-Str. 3, 18059 Rostock, Germany; Analytic Chemistry Department, University of Rostock, Dr.-Lorenz-Weg 1, 18059 Rostock, Germany; Algenol Biofuels Germany GmbH, Magnusstr. 11, 12489 Berlin, Germany; Department of Molecular Physiology, Applied Metabolome Analysis, Max-Planck-Institute of Molecular Plant Physiology, Am Mühlenberg 1, 14476 Potsdam-Golm, Germany; Genetics & Experimental Bioinformatics, Institute of Biology III, University of Freiburg, Schänzlestr. 1, 79104 Freiburg, Germany

**Keywords:** Cyanobacteria, Glucosylglycerol, Isoprene, Metabolome, Promoter, Transcriptome, Salinity

## Abstract

**Background:**

Cyanobacteria are phototrophic prokaryotes that convert inorganic carbon as CO_2_ into organic compounds at the expense of light energy. They need only inorganic nutrients and can be cultivated to high densities using non-arable land and seawater. This has made cyanobacteria attractive organisms for the production of biofuels and chemical feedstock. *Synechocystis* sp. PCC 6803 is one of the most widely used cyanobacterial model strains. Based on its available genome sequence and genetic tools, *Synechocystis* has been genetically modified to produce different biotechnological products. Efficient isoprene production is an attractive goal because this compound is widely used as chemical feedstock.

**Results:**

Here, we report on our attempts to generate isoprene-producing strains of *Synechocystis* using a plasmid-based strategy. As previously reported, a codon-optimized plant isoprene synthase (IspS) was expressed under the control of different *Synechocystis* promoters that ensure strong constitutive or light-regulated *ispS* expression. The expression of the *ispS* gene was quantified by qPCR and Western blotting, while the amount of isoprene was quantified using GC—MS. In addition to isoprene measurements in the headspace of closed culture vessels, single photon ionization time-of-flight mass spectrometry (SPI-MS) was applied, which allowed online measurements of isoprene production in open-cultivation systems under various conditions. Under standard conditions, a good correlation existed between *ispS* expression and isoprene production rate. The cultivation of isoprene production strains under NaCl-supplemented conditions decreased isoprene production despite enhanced *ispS* mRNA levels. The characterization of the metabolome of isoprene-producing strains indicated that isoprene production might be limited by insufficient precursor levels. Transcriptomic analysis revealed the upregulation of mRNA and regulatory RNAs characteristic of acclimation to metabolic stress.

**Conclusions:**

Our best production strains produced twofold higher isoprene amounts in the presence of low NaCl concentrations than previously reported strains. These results will guide future attempts to establish isoprene production in cyanobacterial hosts.

**Electronic supplementary material:**

The online version of this article (doi:10.1186/s13068-016-0503-4) contains supplementary material, which is available to authorized users.

## Background

The development of sustainable energy and chemical feedstock production is needed to decrease CO_2_ emissions and the dependence on fossil fuels. Harvesting solar energy via photosynthesis is one of the nature’s remarkable achievements that could also be a solution for the future global economy. First generation production of green energy, such as bioethanol production, used photosynthetically fixed carbon from crop plants. However, the impacts on the environment and the food supply raised ethical questions about these practices. Therefore, there is a growing interest in using photosynthetic microorganisms to couple CO_2_ capture to chemical synthesis [1]. The ability of cyanobacteria or microalgae to fix CO_2_ into organic matter using solar energy qualifies them as cellular factories for production of biofuels and chemical feedstock. In addition to sunlight as an energy source for carbon assimilation, cyanobacteria require only water and inorganic and trace nutrients for growth [2]. Photosynthetic microorganisms also show high rates of photosynthesis and have the potential to divert a greater amount of assimilated carbon into biotechnologically useful products than crop plants [3].

Compared to most microalgae, cyanobacteria are amenable to genetic manipulation, allowing the introduction of complex biosynthetic pathways into these cells by synthetic biology approaches. These efforts led to many cyanobacterial strains that produce an impressive range of products. Attempts to produce isoprene [4–7] as well as ethanol [8], isobutanol [9], ethylene [10, 11], 1-butanol [12], acetone [13], isopropanol [14], alkanes [15], sucrose [16], or limonene [17, 18] were previously reported. Isoprene (C_5_H_8_) is a volatile C5 hydrocarbon that is preferentially used as feedstock in the rubber industry. Currently, it is produced from fossil carbon sources [19]. Besides the industrial use, isoprene is also a repeating unit of many natural products, the so-called isoprenoids, such as vitamin A and steroid hormones [20]. Isoprene is naturally synthesized by many plants [21], which release this volatile compound into the atmosphere. However, plants are not suitable for large-scale production of isoprene mostly due to the difficulty in collecting it [3]. In addition to plants, heterotrophic bacteria such as *Bacillus cereus, Pseudomonas aeruginosa*, and *Escherichia coli* also naturally produce isoprene [22–24].

Two major pathways for isoprene synthesis are known: the mevalonic acid (MVA) pathway and the 2-C-methyl-d-erythritol 4-phosphate (MEP) pathway. The MVA pathway is active in archaea and in the cytosol of animals, whereas the MEP pathway is used by bacteria, algae, and plants [25, 26]. In the recent years, the genes encoding enzymes of the MEP pathway have been identified and functionally characterized, mainly in *E. coli* [27, 28]. This knowledge allowed genome searches and revealed that genes for the MEP pathway enzymes are present in all cyanobacteria, where they are mainly involved in the synthesis of photosynthetic pigments (Additional file 1). However, the MVA pathway is not present in these organisms. The initial step of isoprene synthesis via the MEP pathway is catalyzed by 1-deoxy-d-xylulose 5-phosphate synthase (DXS), which uses pyruvate and d-glyceraldehyde 3-phosphate as precursors. It has been shown that DXS activity controls the emission of isoprene in plants [29]. The MEP pathway produces two final products: isopentenyl diphosphate and dimethylallyl diphosphate (DMAPP). DMAPP serves as a precursor for carotenoids, the phytol of chlorophyll, and quinones, which act as essential cofactors for photosynthesis [30]. Moreover, DMAPP also serves as precursor for isoprene synthesis by isoprene synthase (IspS, Additional file 1) in plants [21].

Here, we report on our attempts to establish isoprene synthesis in the model cyanobacterium *Synechocystis* sp. PCC 6803 (hereafter *Synechocystis*). In contrast to previous attempts, we used plasmid-based expression of a codon-optimized *ispS* cDNA of kudzu (*Pueraria montana*). The *ispS* expression was controlled by different strong and regulated promoters. It has been proposed that freshwater will become a limiting factor for the future mass production of basic chemicals and biofuels; therefore, these technologies should preferentially be developed in saltwater-based systems [31, 32]. Thus, we investigated the isoprene production rate in the presence of high and low NaCl concentrations. Moreover, we analyzed the effects of isoprene production on cyanobacterial metabolism and the regulation of gene expression via metabolomics and transcriptomics. A new online measurement of isoprene production by single photon ionization time-of-flight mass spectrometry (SPI-MS) allowed the use of an open-cultivation system, which resulted in higher isoprene production rates than in closed-cultivation systems.

## Results

### Generation of expression cassettes and producing strains

The *ispS* gene from *Pueraria montana* (kudzu vine) was selected to establish isoprene synthesis in *Synechocystis* because it has been successfully used before [4]. The codon-optimized cDNA without the transit peptide sequence for chloroplast import was obtained via gene synthesis (Additional file 2). For the upstream of the *ispS* start codon, we initially inserted the core element of the strong *SynechocystispsaA* promoter comprising the -10 and -35 region and the transcriptional start. The ribosome-binding site from the 5ˈUTR of the iron-regulated *isiA* gene was inserted between the promoter and start codon for high translational efficiency. For the downstream of the stop codon of the *ispS* gene, the phage lambda *oop* terminator was cloned for efficient termination of transcription and increased transcript stability. The entire synthetic DNA fragment was then cloned into the pVZ325 vector (Additional file 3). Using a plasmid-based expression cassette allows for versatile transformation into different production strains. To swap promoters controlling the *ispS* coding sequence, the *psaA* promoter could be removed by *Sal*I/*Nde*I digestion and then replaced by alternative promoters. This strategy allowed the generation of several *Synechocystis* strains that carry different *ispS* promoter combinations for isoprene synthesis (Table 1). Here, we analyzed six different strains carrying four different promoters: P_*rbcL*_, P_psaA*_, P_*psbA2*_, and P_*tac*-*lacI*_. The first three are strong endogenous promoters of *Synechocystis*, while the fourth is an IPTG-inducible promoter from *E. coli*. In addition, two *Synechocystis* strains were generated harboring constructs for the parallel overexpression of the endogenous *dxs* gene under the control of different promoters (Table 1).

**Table 1:** Short description of the isoprene-producing strains, which carry different promoter gene combinations for the isoprene synthesis

No	Genotype	Resistance
# 642	pVZ325-P_*rbcL*_-IspS-oop	Gentamycin/spectinomycin
# 643	pVZ325-P_*tac*-*lacI*_-IspS-oop	Gentamycin/spectinomycin
# 704	pVZ326-P_*rbcL*_-IspS-oop-P_*tac*-*lacI*_-IspS-oop	Gentamycin
# 731	pVZ326-P_*rbcL*_-IspS-oop-P_psaA*_-IspS-oop	Gentamycin
# 796	pVZ326-P_*psbA2*_-dxs-oop-P_psaA*_-IspS-oop	Gentamycin
# 816	pVZ326-P_psaA*_-IspS-oop-P_*rbcL*_-dxs-oop	Gentamycin

### Isoprene production in the presence of low NaCl concentrations

To assess isoprene production, these strains were cultivated under photoautotrophic conditions in standard, low salt (NaCl) BG11 medium in a closed system, where isoprene accumulated in the headspace over 24 h. This time point was selected because previous studies showed linear isoprene accumulation under similar culture conditions over at least 48 h [6, 7]. Headspace samples were analyzed by gas chromatography coupled to mass spectroscopy (GC—MS). The isoprene peak was observed at 5 min GC retention time, consistent with the isoprene standard retention time. The obtained MS spectrum of this peak displayed typical isoprene mass fragments, *m/z* 39 and 53, and the molecular ion *m/z* 68 (Additional file 4). These data showed that the *ispS*-containing strains expressed the plant enzyme in sufficient amounts for isoprene synthesis, whereas no isoprene emission was detected using *Synechocystis* wild type (WT) cells.

**Fig. 1:**
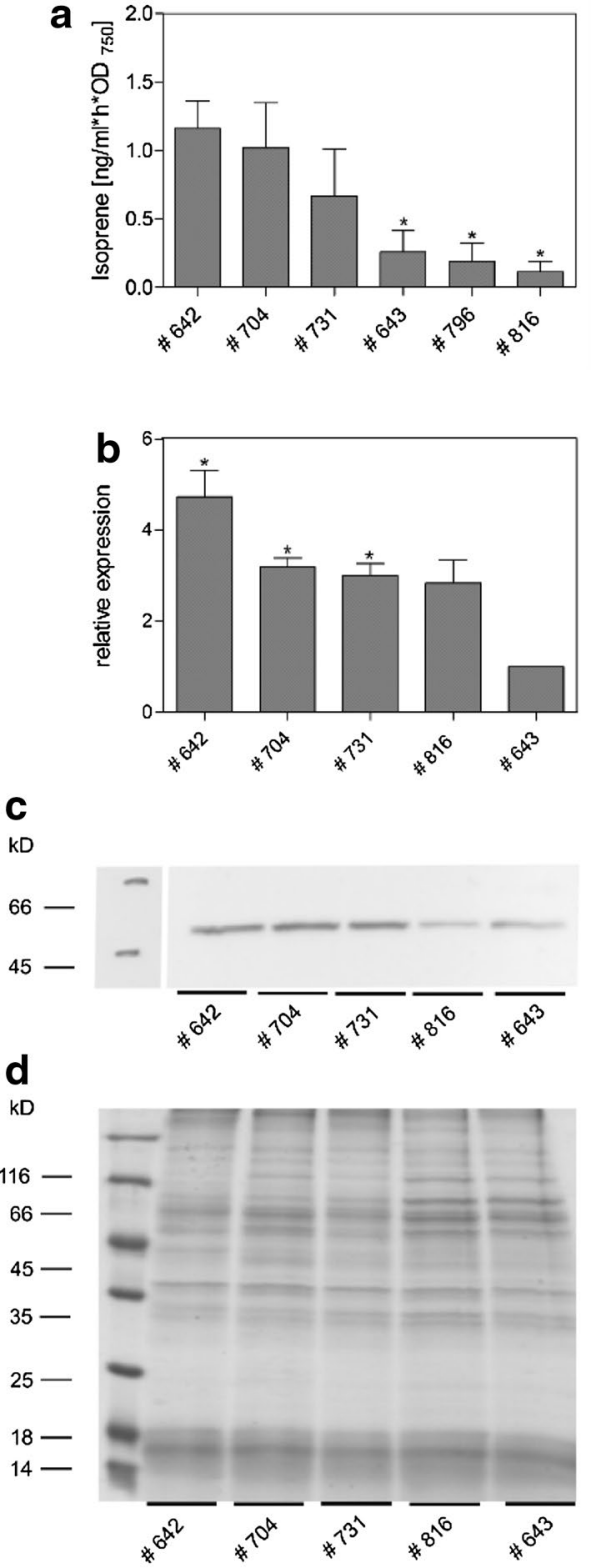
Isoprene production rate and *ispS* expression in standard medium (0 % NaCl) of the different *Synechocystis* strains. **a** Isoprene production is expressed in relation to optical density (OD_750_; a measure of cell number) over 24 h of phototrophic growth in the *Synechocystis* strains carrying various constructs for isoprene synthesis (see Table 1). Mean values and standard deviation from three independent growth experiments with each two technical replicates are given. Statistical significant differences (*p* ≤ 0.05) between strain # 642 and other are marked by asterisk. **b** Expression of the *ispS* gene in the different *Synechocystis* strains. The relative expression (*rnpB* amount was used as internal loading control) of *ispS* was estimated by qPCR. The expression in strain # 643 was set to 1. **c** Accumulation of the IspS protein in the different *Synechocystis* strains. The protein amounts were visualized by immune-blotting. Equal amounts of soluble protein (10 μg) were loaded on gels. The blot was incubated with a specific IspS antibody and the IspS protein was visualized by chemoluminescence. **d** Coomassie-stained SDS-PAGE of proteins which correspond to the Western Blot above

The initial cultivation in NaCl-free, standard medium resulted in highly reproducible, specific isoprene production rates for each strain (Fig. 1a). Strain # 642, in which the *ispS* is under the control of the strong *rbcL* promoter, showed the highest productivity of 1.16 ng/ml h OD_750_. Similar isoprene production rates were observed with strain # 704 (1.02 ng/ml h OD_750_), in which two copies of the *ispS* are present, one under the control of *P*_*rbcL*_ and the other controlled by *P*_*tac*_. Strain # 731, in which *ispS* is co-expressed with *dxs*, showed intermediate isoprene production. Strains # 643 (*ispS* driven by the *E. coli tac* promoter), # 796 and # 816 showed significantly lower isoprene production rates (approximately ten times less than strain # 642). Strain # 816 expresses *ispS* under the control of *P*_psaA*_, and the *dxs* gene is controlled by *P*_*rbcL*_ (Fig. 1a), whereas in strain # 796 these two genes are controlled by *P*_psaA*_. Growth and pigmentation of the different isoprene-producing strains did not differ from WT under these cultivation methods (Additional file 5 A/B).

To determine if the production rates correlate with expression levels of *ispS* in these strains, we isolated total RNA from isoprene-producing cells and performed qPCR analysis. The *ispS* expression was compared to the level of the constitutively expressed *rnpB*, which encodes the RNA subunit of ribonuclease P (RNase P). The *ispS* expression was arbitrarily set to 1 for the low isoprene-producing strain, # 643. The expression analysis indicated that mRNA levels of *ispS* in the different strains (Fig. 1b) are consistent with the strain-specific isoprene production rates under standard, low salt (NaCl) growth conditions because strain # 642 showed the highest isoprene productivity and the highest *ispS* mRNA level, whereas isoprene productivity and *ispS* mRNA level were lower in strain # 816. This finding was supported by Western blotting analyses. Generally, we observed consistent results in the *ispS* expression at the mRNA and protein level (Fig. 1c). Thus, isoprene production appears to depend on *ispS* expression in the different *Synechocystis* strains, whereas the co-expression of *dxs* had no positive effect on isoprene amounts under our standard cultivation conditions.

### Effect of NaCl on isoprene production

**Fig. 2:**
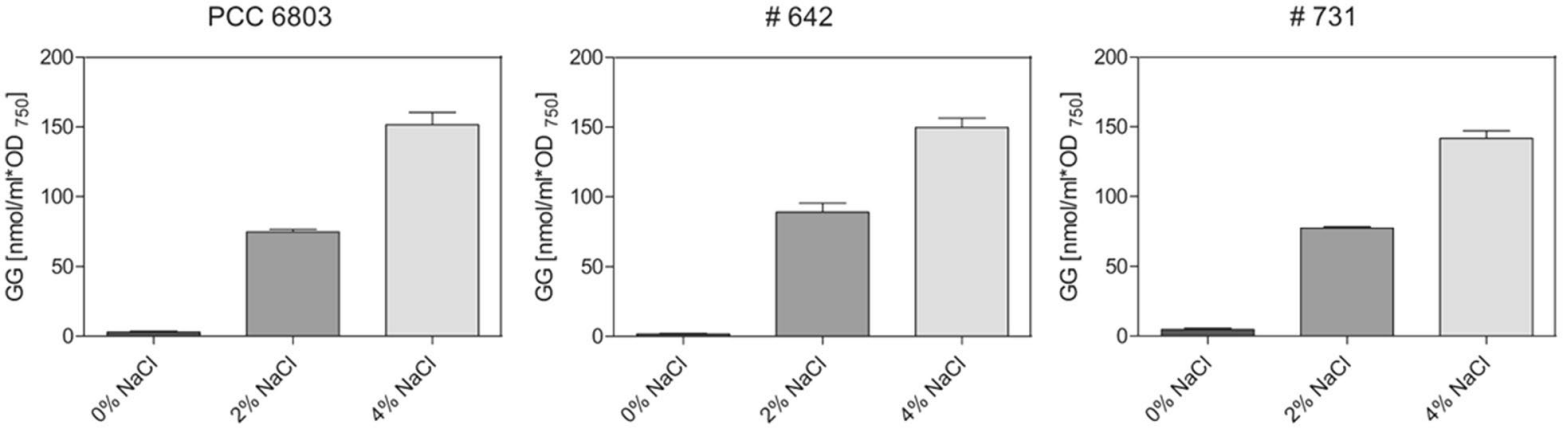
Salt (NaCl)-dependent accumulation of the compatible solute glucosylglycerol (GG) in selected *Synechocystis* strains, which carry different constructs for isoprene synthesis in comparison to the wild type (PCC 6803). For GG analysis, the cells were harvested from the closed-cultivation system after 24 h of isoprene production in the presence of 0, 2, or 4 % NaCl. Data are the mean ± SD of triplicate cultures

To analyze the effects of increasing salinity on isoprene productivity of *Synechocystis* strains, the standard growth medium was supplemented with 2 or 4 % NaCl. The addition of 2 % NaCl had only minor effects on growth, whereas growth of the isoprene producer strains and the WT decreased at 4 % NaCl. Despite the clear effects on growth, pigmentation was not significantly changed at different salinities. The chlorophyll *a* relative to phycocyanin or to carotenoids ratios was similar in isoprene producer and WT cells at different NaCl concentrations (Additional file 5 A). The major compatible solute, glucosylglycerol (GG), which allows *Synechocystis* cells to grow at enhanced salinities, accumulated equally with increasing NaCl concentration in isoprene producer and WT cells (Fig. 2). The GG level rose to approximately 150 nmol/ml OD_750_ in isoprene-producing as well as WT cells at 4 % NaCl.

**Fig. 3:**
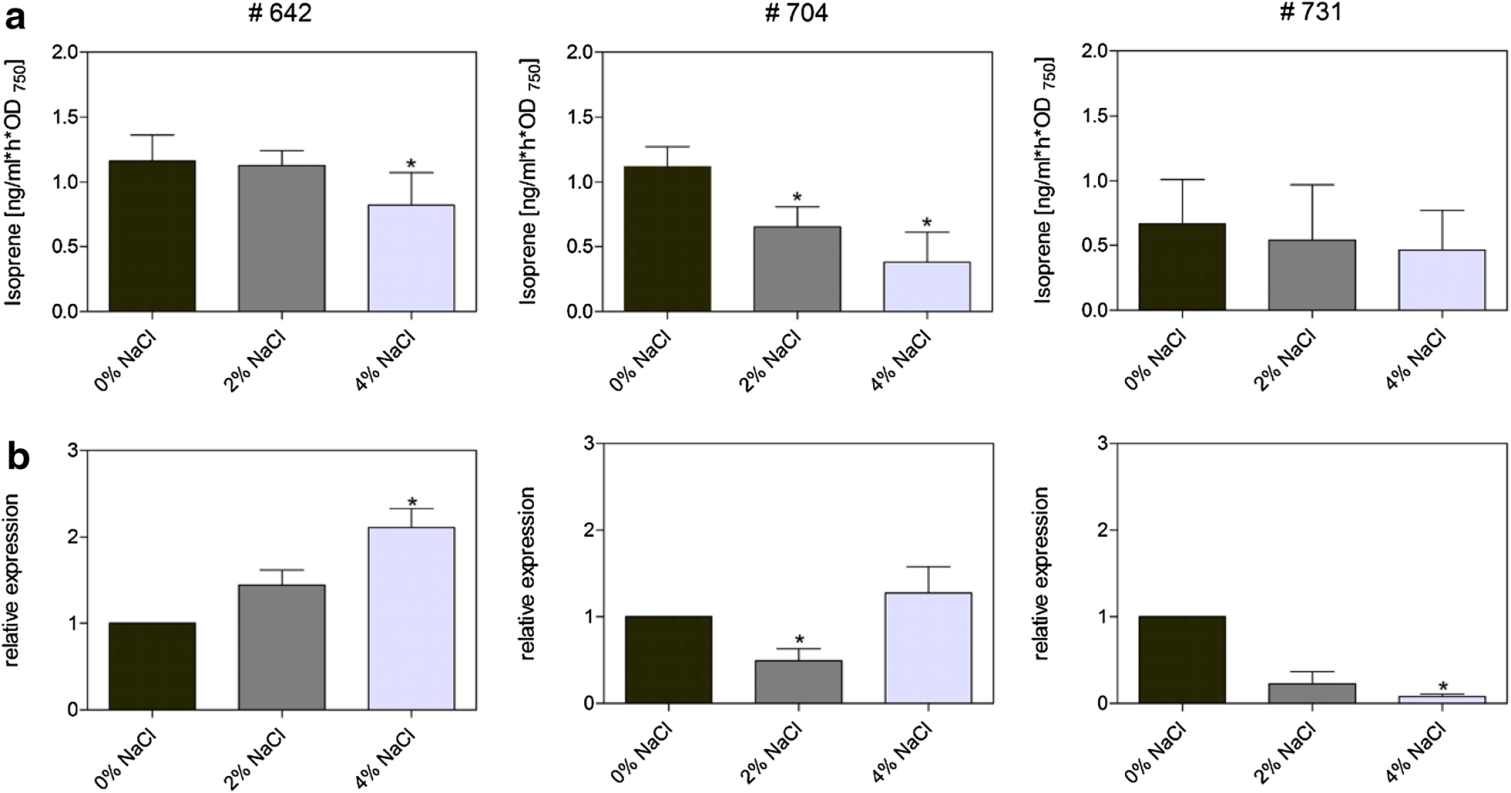
Influence of salinity on isoprene production and *ispS* expression. **a** Isoprene production rates of selected *Synechocystis* strains are shown in the presence of 0, 2, or 4 % NaCl. Isoprene production is expressed in relation to optical density (OD_750_; measure of cell density) over 24 h of phototrophic growth in the *Synechocystis* strains, which carry various constructs for isoprene synthesis (see Table 1). Statistical significant differences (*p* ≤ 0.05) to the strain # 642 at 0 % NaCl are marked by *asterisk*. **b** Salt (NaCl)-dependent expression of the *ispS* gene in the different *Synechocystis* strains. The relative expression (*rnpB* amount was used as internal loading control) of *ispS* was estimated by qPCR. Expression of *ispS* at 0 % NaCl was set to 1

Isoprene productivity decreased in all strains at high NaCl concentrations (Fig. 3a). The relative drop in productivity in cells, supplemented with NaCl, varied between strains harboring different *ispS* expression cartridges. Strain # 704 showed a significant decline of 68 %, while productivity of strain # 642 decreased by only 29 % in the presence of 4 % NaCl compared to 0 % NaCl. Surprisingly, expression of the *ispS* gene was stimulated by NaCl. For example, the *ispS* mRNA level is twofold higher in strain # 642 in the presence of 4 % NaCl compared to standard medium (Fig. 3b). Moreover, an increased *ispS* expression was also found for strain # 704 at 4 % NaCl; however, it showed a slightly lower expression at 2 % NaCl. These findings are consistent with the reported slight stimulation by NaCl of *rbcL* and *psbA* expression in *Synechocystis* WT cells at the mRNA level (see: http://www.cyanoexpress.sysbiolab.eu/). However, the increased mRNA levels of *ispS* are not always translated into higher protein amounts. Therefore, future measurements of enzyme activities would be necessary to support the mRNA data.

### Metabolomic analysis

**Fig. 4:**
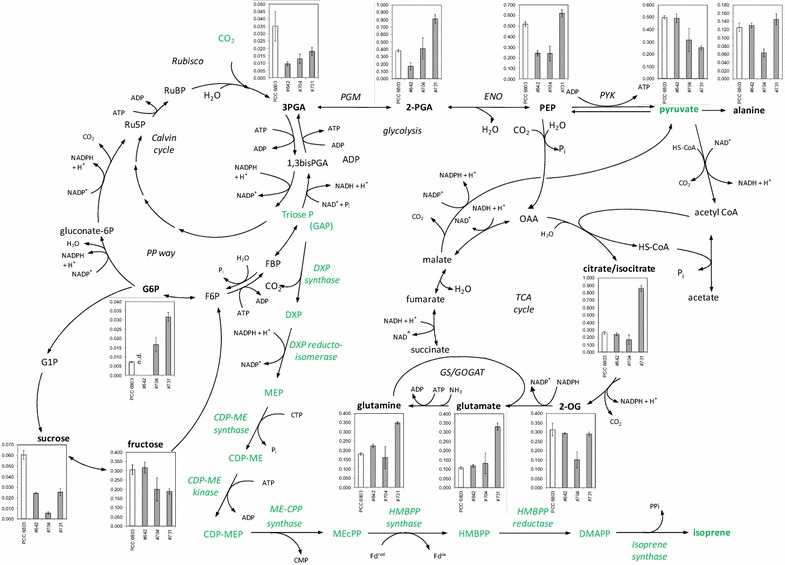
Changes of the metabolism in cells of the wild type (WT) compared to three isoprene-producing strains (see Table 1) of *Synechocystis* sp. PCC 6803, which were cultivated for 24 h in a closed flask system under salt-free (without NaCl) conditions. *Bars* correspond to normalized values of two biological replicates measured by at least two technical replicates. Factors are calculated relative to WT. *Error bars* represent standard error

To analyze how isoprene production affects the overall metabolism of *Synechocystis*, we performed a non-targeted metabolome analysis. To this end, the metabolome of selected high to medium isoprene producer strains, namely # 642, # 704, and # 731 (see Fig. 1a), was analyzed by GC—MS-based metabolite profiling [33] in comparison to WT cells (PCC 6803 in Fig. 4). The samples were obtained under isoprene-producing conditions in the closed-cultivation system, i.e., 24 h after isoprene production initiation under NaCl-free conditions in the presence of added bicarbonate as inorganic carbon source. In total, 154 analytes were annotated in this dataset; however, the majority of these compounds could not be assigned to specific intermediates. Unfortunately, none of the intermediates of the MEP pathway were detected. Nevertheless, we were able to quantify 58 metabolites of carbon and nitrogen metabolism (Fig. 4; Additional file 6 shows total metabolite data), which allowed us to investigate the effects of isoprene production on cyanobacterial primary metabolism.

In general, the metabolic data displayed only small differences between the WT and the isoprene-producing strains (Fig. 4). For example, strain # 642, which showed the highest isoprene production rate, was in most cases more similar to WT than strain # 731, which produced lower isoprene amounts. We quantified pyruvate, one of the substrates of the MEP pathway, but glyceraldehyde 3-phosphate, the second substrate of the MEP pathway, was below the detection limits of the GC—MS profiling method in both WT and the isoprene-producing strains. Strain # 642 with high production rates had unchanged pyruvate levels. In the equally high isoprene-producing strain # 704, pyruvate was variable but did not show a significant reduction. The medium-level production strain # 731, however, had a significantly reduced pyruvate level. The reduced pyruvate levels in this strain coincided with increased levels of isocitrate/citrate, glutamate, and pyroglutamate/glutamine, which were only observed in this strain. The highest producer strain, # 642, showed a significant decrease in PEP, the first product from pyruvate. Alanine and 2-oxoglutarate (2OG) levels remained unchanged except for an approximately twofold depletion in strain # 704. In conclusion, only strain # 642, which has a single copy of *ispS* under the control of P_*rbcL*_, compensated for carbon drain from the pyruvate pool without effects on the downstream products. However, this compensation in strain # 642 was associated with depletion in the upstream PEP and glycerate 3-phosphate (3PGA) pools. The medium producer strain # 731 had a smaller decrease in the 3PGA pool, a significantly increased glycerate 2-phosphate content and unchanged PEP levels, whereas strain # 704 appeared to have an intermediate phenotype, with reduced PEP and 3PGA but unchanged glycerate 2-phosphate pools.

All isoprene producer strains consistently accumulated more lysine, and producer strains # 642 and # 731 accumulated more pyroglutamate, in comparison to the WT. In addition, a significant decrease of the soluble sugar sucrose was detected in all isoprene-producing strains (Fig. 4). This decrease in organic carbon pools was also observed for glycerol 3-phosphate (G3P; Additional file 6) amounts in isoprene producers compared to the *Synechocystis* WT under standard growth conditions. In contrast, the amount of the oxidative pentose phosphate (OPP) cycle intermediate glucose-6-phosphate increased in strains # 704 and # 731 (Fig. 4). Besides these differences in known metabolites, changes in unknown metabolites could also be detected. For example, the unknown compound A273003-101 strongly accumulates in strain # 642.

### Transcriptomic analysis

To analyze how isoprene production affects the overall gene expression of *Synechocystis*, we performed a transcriptomic analysis using an optimized DNA microarray. To this end, we compared the *Synechocystis* WT and isoprene-producing strain # 642, which showed the highest production rates. In total, 505 DNA regions (features) showed significant differential expression, i.e., their log_2_-fold change (FC) was ≥1 or ≤−1 with an adjusted *p* value ≤ 0.05. The altered transcripts included 170 untranslated regions of mRNAs (UTRs), 69 clustered regularly interspaced short palindromic repeat (CRISPR) spacers of all three *Synechocystis* CRISPR clusters, 121 *cis*-antisense RNAs (asRNAs), 30 intergenic encoded ncRNAs (sRNAs), and 115 mRNAs. The complete transcriptomic dataset is available as Additional file 7 and as graphical representations in the supplementary genome plots (Additional file 8).

**Fig. 5:**
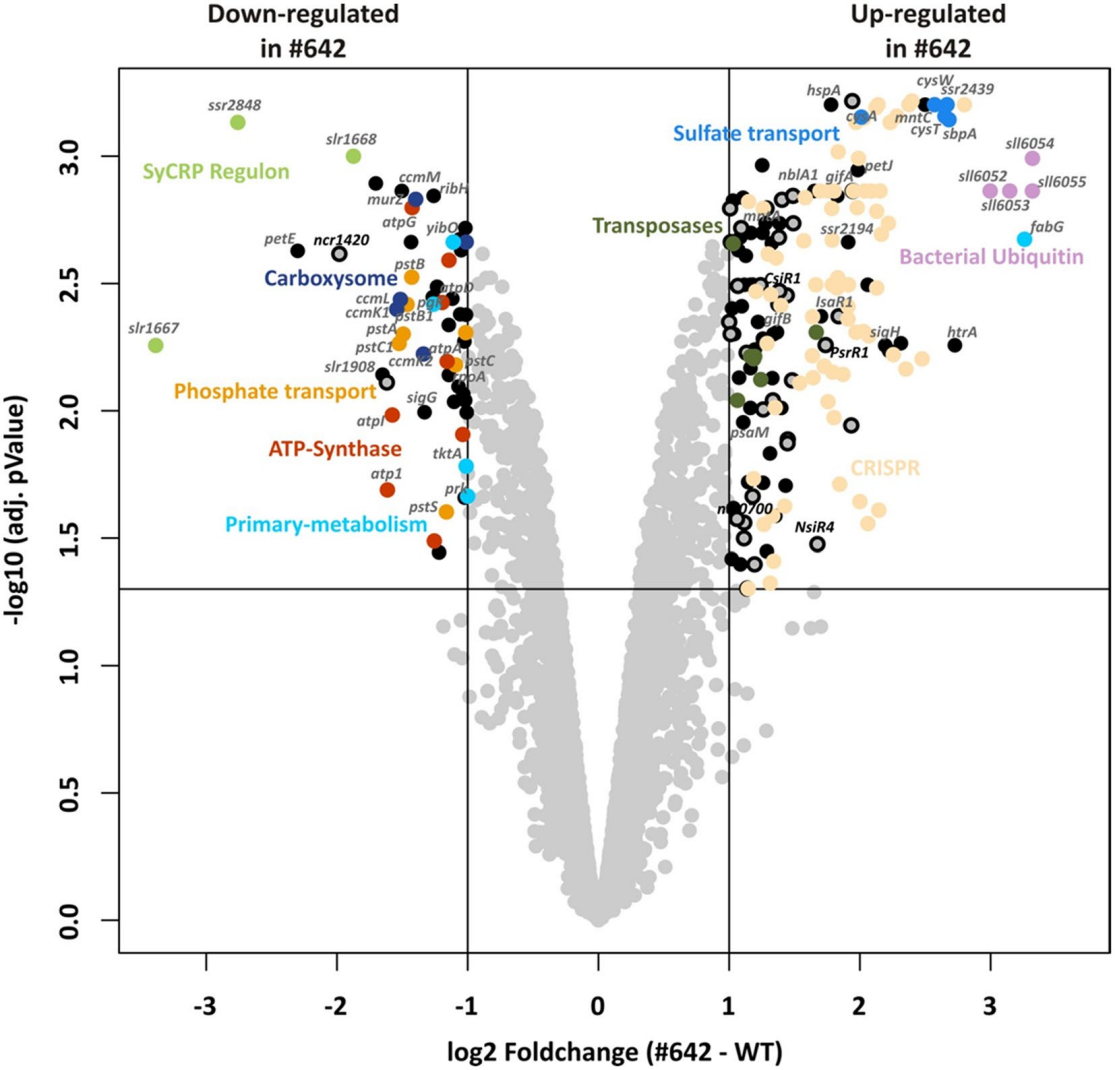
Volcano plot of the microarray results. Only data of protein encoding genes and of intergenic encoded sRNAs are shown. Significantly differential expressed genes (log_2_ fold change ≥ 1 and adjusted *p* value ≤0.05) are in the *upper left* and *upper right* sector. Functional related features are color-coded and selected features are named. Intergenic encoded sRNAs are indicated by a *gray circle* with a *black border*

We concentrated on differentially regulated sRNAs and mRNAs (Fig. 5). Interestingly, three of the strongest downregulated genes (*slr1668*, *slr1667*, *ssr2848*) in strain # 642 are possibly controlled by the cAMP-controlled transcriptional regulator SYCRP1 because the ORFs *slr1668* and *slr1667* showed deregulated expression in the corresponding mutant [34], whereas the upstream region of *ssr2848* was identified in an in silico target prediction for SYCRP1 [35]. Genes *slr1668* and *slr1667* encode proteins that are involved in the construction of cell surface components [36], but the precise function of these proteins is unknown. The gene *ssr2048* encodes a small protein of only 72 amino acids that has no matches to other proteins in the entire database except a single protein in S*ynechocystis* sp. PCC 6714. The transcriptome data indicated that isoprene production is stressful for *Synechocystis* cells because many genes that are known to be induced under environmental stress showed increased RNA levels. For example, we found induction of the mRNA of the serine protease HtrA, the small heat shock protein HspA and several transposases. Four genes encoding a bacterial ubiquitin system (*sll6052*–*sll6055*), which might be involved in degradation of misfolded proteins or proteome remodeling, also showed increased transcription in the isoprene producer cells (Fig. 5). The iron stress-activated sRNA IsaR1 and the high light stress-induced sRNA Syr1/PsrR1 [36] were also upregulated. The observed repression of *petE* and the induction of *petJ* indicate disturbed internal copper availability because these genes are known to respond to changing copper concentrations in *Synechocystis* [37, 38]. Furthermore, the demand for some macronutrients appeared to be changed. Nearly the complete *pho* regulon involved in phosphate uptake (*pst1* operon: *sll0680*–*sll0684*; *pst2* operon: *sll1248* + *sll1249*, *phoA*) was downregulated, whereas the sulfate transport operon was induced (*slr1452*–*slr1455*). Nitrogen-related genes were also differentially transcribed. The sRNA NsiR4 [39], the mRNAs *nblA1* and *nblA2*, and the glutamine synthetase inactivating factors *gifA* and *gifB* were upregulated. We observed no differential expression of genes encoding proteins involved in inorganic carbon uptake, but the expression of the carboxysome shell protein operon (*sll1028*–*sll1032*) decreased. The genes for the core photosynthetic proteins were largely unaffected. Only *psbZ* was significantly downregulated, and *psbM* and *psaM* were upregulated, whereas the full *atp1* operon (*sll1321*–*sll1327*) encoding ATP synthase subunits was repressed.

Only a few genes encoding primary metabolism enzymes showed transcriptional changes. For example, all genes of the MEP pathway showed no significant differences between the isoprene producer strain and the WT. However, genes for the glycolysis enzymes phosphoglycerate kinase (*pgk*, *slr0394*) and phosphoglycerate mutase (*yibO*, *slr1945*) as well as the OPP cycle enzymes transketolase (*tktA*, *sll1070*) and phosphoribulokinase (*prk*, *sll1525*) showed reduced expression. Finally, *sll0330* accumulated to approximately 10-times higher levels and was among the three most strongly induced mRNAs. The corresponding protein is annotated as sepiapterin reductase or 3-ketoacyl-ACP reductase; however, sepiapterin reductase activity was not confirmed in vitro [40].

### Online isoprene measurements via single photon ionization time-of-flight mass spectrometry (SPI-MS)

**Fig. 6:**
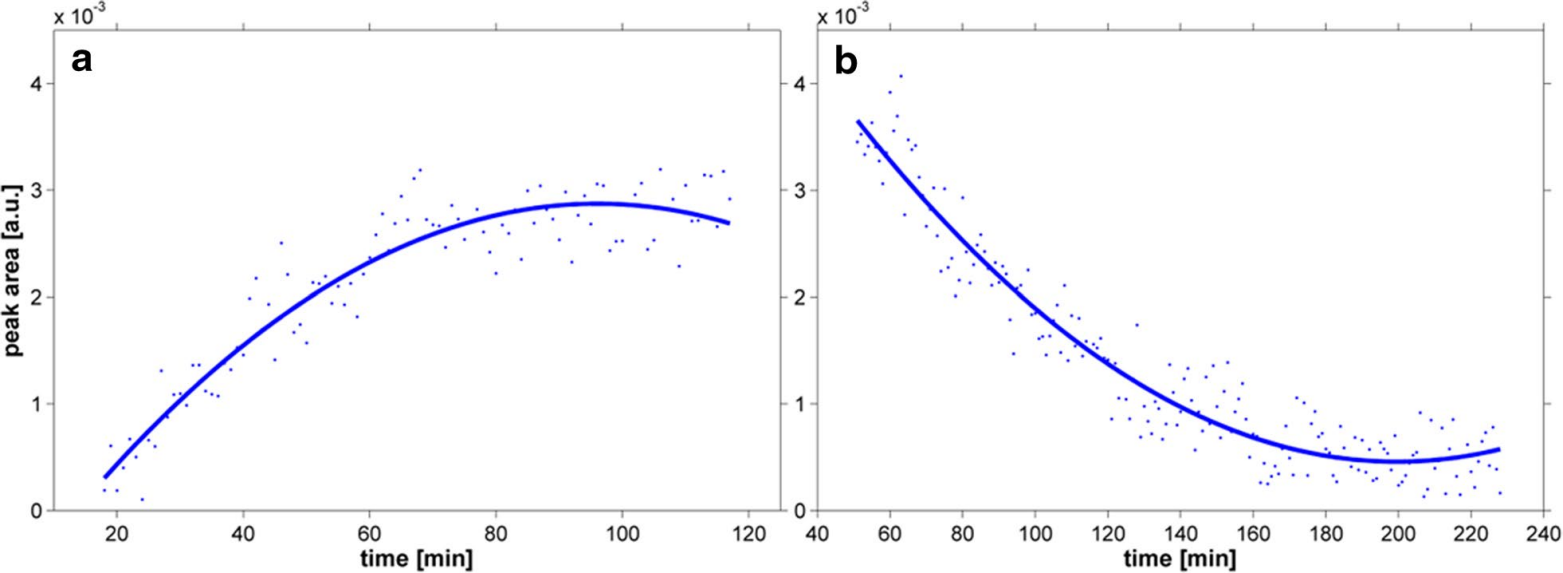
Quantitative online SPI-MS isoprene measurements of strain # 642 under different culture conditions (one data point illustrates an averaged spectrum over 60 s). Line displays values from the fitted data approach. **a** Shift from medium light conditions (70 µmol photons/m^2^ s) to high light conditions (175 µmol photons/m^2^ s); **b** shift from high light conditions to dark (0 μmol photons/m^2^ s)

**Fig. 7:**
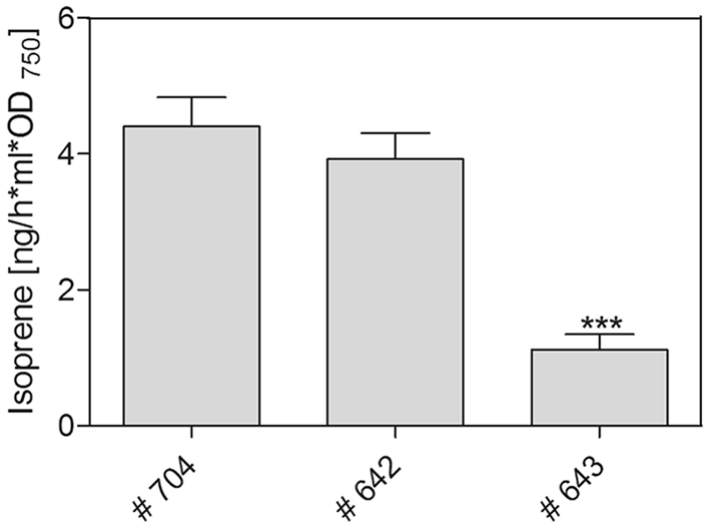
Isoprene production rates by different *Synechocystis* strains, which carry the *ispS* gene under the control of different promoters (see Table 1), in the open-cultivation system at 0 % NaCl. Isoprene production, measured by SPI-MS system, is expressed in relation to optical density (OD_750_; a measure of cell density) over 24 h of phototrophic growth. Mean values and standard deviation from three independent growth experiments with each two technical replicates are shown. Statistical significant differences (***p < 0.001) to the strain # 642 are marked by *asterisk*

All previous isoprene-producing attempts, which are described here or in the literature (e.g., [4]), used closed culture systems to collect the volatile product in the headspace. However, *Synechocystis* grows slower in such systems than in open-cultivation systems. Therefore, we established an online measuring system via SPI-MS to register continuous isoprene production in the gas phase of an open-cultivation system. The SPI-MS measurements also enabled snapshots during the production, which allows for analysis of production profiles and isoprene amounts with changing parameters. For example, the measurements displayed in Fig. 6 show that isoprene immediately accumulated after switching the light on until a steady-state value was reached. At this point, the maximal production rates at the given light intensity could be quantified. In contrast, the isoprene amount decreased within 2–3 h to the limit of detection after switching the light off. Regarding maximal production rates, similar steady-state isoprene production levels were obtained compared to classical GC–MS measurements for the different isoprene-producing strains. Although there were approximately 4.5-times higher isoprene production rates in all strains in the open-cultivation systems, the relations of the different strains to each other were similar in the closed- and open-cultivation systems. As observed previously, strains # 704 and # 642 showed four times higher isoprene production rates compared to # 643 (Fig. 7).

## Discussion

Here, we present an integrative analysis of isoprene production in the model cyanobacterium *Synechocystis* expressing *ispS* from kudzu vine under the control of various promoters. In the widely used closed-cultivation system, we obtained the highest isoprene production rate [approximately 1.2 ng/ml h OD_750_ equivalent to 93 μg/g dry cell weight (DCW)] under low salt (NaCl) conditions with strain # 642, in which *ispS* is under the control of the strong *rbcL* promoter (Table 2). This rate is approximately 2-times higher than the maximum rate reported in the pioneering study by Lindberg et al. [4] and in the same order of magnitude as recent independent studies [5, 7]. The highest isoprene productions rates (4.2 ng/ml h OD_750_ equivalent to 336 μg/g DCW with strain # 642) were found using the open-cultivation system with the newly established online isoprene detection by SPI-MS. Only slightly lower isoprene production rates of 250 μg/g DCW were reported when the *ispS* gene and the complete MVA pathway for DMAPP synthesis were expressed in the cyanobacterial host [6].

**Table 2:** Comparison of our maximum isoprene production per day with data, which were reported in previous studies using cyanobacteria

Maximum isoprene production per day	Expression cassette localized on …	Reference
50 µg/g DCW	Chromosome	[4]
67 µg/g DCW	Chromosome	[5]
250 µg/g DCW	Chromosome	[6]
63 µg/g DCW (pH 10)	Chromosome	[7]
25 µg/g DCW (pH 10 + 600 mM NaCl)	Chromosome	[7]
93 µg/g DCW (0 % NaCl)	Plasmid	This study (# 642 in the closed system)
71 µg/g DCW (4 % NaCl)	Plasmid	
336 µg/g DCW (0 % NaCl)	Plasmid	This study (# 642 in the open system)

The isoprene values of Chaves et al. [7] and of Bentley and Melis [5] were recalculated to the dry weight basis using the reported relation of OD_750_ to dry cell weight (DCW). The calculation of the dry cell weight in our study based on the determined relation OD_750_ to dry cell weight (DCW) shown in the Additional file 10

We found that *ispS* expression and isoprene production rate correlated under standard, low salt (NaCl) conditions. The best isoprene-producing strain, # 642, where *ispS* was under the control of the strong *Synechocystis* promoter of *rbcL*, accumulated the highest *ispS* mRNA and also IspS protein levels. The role of promoter selection for the expression of enzymes producing biotechnological valuable products has been highlighted in many studies. Lindberg et al. [4] and Bentley et al. [6] used *P*_*psbA2*_ to express *ispS* for isoprene production in cyanobacteria. This promoter was also used here (strain # 796) and resulted in considerably lower isoprene production rates than using *P*_*rbcL*_. Angermayr et al. [41] compared the effect of various promoters (*P*_*rnpB*_*, P*_*psbA2*_ and *P*_*trc*_) to express the l-lactate dehydrogenase in cyanobacteria. These authors concluded that neither native nor artificial promoters were strong enough to produce the enzyme in sufficient quantities for considerable l-lactate production in *Synechocystis*. Recently, Zhou et al. [42] discovered and verified an extremely strong promoter upstream of the gene for phycocyanin, which was then used for protein expression in *Synechocystis*. They obtained up to 15 % of recombinant protein per total soluble protein, which is comparable to yields in *E. coli.* Formighieri and Melis [43] also reported that the heterologous promoter *P*_*trc*_ and the native strong promoter *P*_*cpc*_ improved the expression of the isoprenoid synthesis enzyme β-phellandrene synthase 2.5- to fourfold and isoprenoid production 10- to 20-fold, respectively, compared to the application of the widely used *psbA2* promoter in *Synechocystis*.

The regulation of the cyanobacterial MEP pathway has not been characterized, although the genes have been identified [44, 45]. Previous studies showed that MEP pathway products, such as the carotenoids myxoxanthophyll and zeaxanthin, accumulate under high light conditions in plants and cyanobacteria [46, 47]. Expression profiling of *Synechocystis* also showed that *P*_*rbcL*_ and *P*_*psbA2*_ are light-induced (http://www.cyanoexpress.sysbiolab.eu/). Thus, our experiments were performed in the presence of high light intensities to stimulate *ispS* expression and isoprene production. However, we did not observe any change in pigmentation in the different production strains compared to WT (Additional file 5 A). This finding indicates that even in our best isoprene production strain, the capacity of the MEP pathway was not limiting pigment synthesis. This assumption is supported by our transcriptome analysis, which did not reveal any change in the expression of genes for MEP pathway enzymes in isoprene-producing strain # 642. However, the MEP pathway, particularly the DXS activity, was found to be rate-limiting in trials to produce carotenoids and other isoprenoids in *E. coli* [48, 49]. It is widely accepted that DXS is the regulatory enzyme in the MEP pathway and constitutes a bottleneck, suggesting it as a target for pathway engineering [50]. Therefore, we aimed to improve the MEP pathway by co-expression of the *dxs* gene from *Synechocystis*, which, however, did not result in further stimulation of isoprene levels. Several reports indicate that DXS and other MEP pathway enzymes in plants are regulated at the biochemical level, for example by internal isoprene amounts [29, 51–53]. The DXS proteins from plants and cyanobacteria are very similar (approximately 50 % identical amino acid residues, more than 90 % similarity), which makes it likely that the regulatory properties are also conserved. This assumption is supported by our observation that isoprene production is higher in the open than in the closed-cultivation system. In the latter system, isoprene accumulates in the headspace, which might have a negative impact on MEP pathway activity. To circumvent limitations of the native MEP pathway, Bentley et al. [6] expressed the MVA pathway in *Synechocystis*. They obtained a 2.5-fold higher isoprene production in *Synechocystis* strains carrying the *ispS* gene and the MVA pathway compared to strains carrying only the *ispS* gene (see Table 2).

It has been proposed that cyanobacteria should be cultivated for biotechnological purposes in seawater to avoid competition for freshwater resources [31]. *Synechocystis* is a euryhaline strain and can resist up to twofold seawater concentrations [32], thus allowing testing in the presence of high NaCl concentrations. We found a decline in isoprene productivity with increased NaCl concentrations despite increased *ispS* expression. Similar results were reported recently, where isoprene productivity also declined in media supplemented with 600 mM NaCl, mostly due to a prolonged lag phase [7]. In our case, the lowered isoprene yield is likely explained by reduced carbon flow into the MEP pathway. In cells of *Synechocystis*, which were stressed with NaCl, the compatible solute GG is synthesized from G3P and ADP-glucose [54]. Our metabolomic study showed that already in the presence of low NaCl concentrations the amount of G3P decreased in isoprene-producing cells. Hence, it is likely that isoprene producers might become further carbon-limited under high salt (NaCl) conditions due to competing GG synthesis using G3P as a precursor. Sucrose acts as minor compatible solute in *Synechocystis* [54]. Again, our metabolomic analysis showed that this pool is depleted in isoprene-producing cells compared to WT. In conclusion, isoprene production clearly competes with sugar synthesis in *Synechocystis* and may divert carbon flow from expendable metabolite pools, which can be drained under NaCl-free conditions but are required for compatible solute production in the presence of high NaCl concentrations. Similar results were published previously, showing that lowering OPP cycle activity decreased isoprene production rates [55], whereas feeding OPP cycle intermediates enhanced MEP pathway activity and isoprenoid synthesis [56]. These results support the assumption that the branching of carbon for compatible solute production caused decreased isoprene production, which is not compensated by increased *ispS* expression under saline conditions.

Signs of a deregulated carbon metabolism were also obtained by transcriptomic analysis (see Fig. 5) because transcripts for the glycolysis enzymes phosphoglycerate kinase and phosphoglycerate mutase were lower, which correlated with lowered 3PGA pools in isoprene-producing cells. Moreover, the mRNA levels for the OPP cycle enzymes transketolase and phosphoribulokinase declined, which is consistent with glucose 6-phosphate accumulation in isoprene-producing cells. Other transcriptional changes indicate an imbalanced growth because genes encoding proteins involved in nutrient transport or regulation of N-assimilation (e.g., *nblA*, *gifA*, *nsiR4*) showed altered expression in the isoprene producers. Despite the induction of *hspA* and PsrR1, which are induced under salt (NaCl) stress or high light stress conditions, genes characteristic of severe stress of *Synechocystis*, such as *sod*, or genes encoding chaperones and thioredoxins [57] showed no expression changes. This observation supports the notion that our isoprene-producing cells were only weakly stressed, which is consistent with their unchanged growth and pigmentation in comparison to the WT.

## Conclusion

In summary, our integrative analysis provides evidence that carbon partitioning likely limits isoprene production in *Synechocystis* as discussed before by Lindberg et al. [4]. These authors assumed that photosynthetic carbon is primarily converted into sugar (80–85 %), whereas synthesis of fatty acids (~10 %) and terpenoids (3–5 %) lag far behind. This assumption is consistent with our metabolic and transcriptomic analysis of strain # 642, which showed the highest isoprene production rate. The pool of soluble sugar was found to be reduced, and fatty acid synthesis was also affected because the *sll0330* gene, which presumably encodes the 3-oxoacyl-[acyl-carrier-protein, FabG] reductase that catalyzes the first reductive step in the elongation cycle of fatty acid biosynthesis, was highly induced in the isoprene-producing cells. Collectively, our results also indicate that metabolic engineering strategies must be applied to alter the carbon partitioning in the cell for further improvement of isoprene production. The sizes of precursor pools are probably more important for the final yield than higher accumulation of IspS and MEP pathway enzymes, such as DXS. Recently, it has been shown that cyanobacterial 1-butanol production based on a CoA-dependent pathway was highly improved by increasing the rate of acetyl-CoA synthesis [58]. Hence, a better understanding of the regulation of the MEP pathway in combination with optimized flux of carbon to the precursors will be necessary to further increase the isoprene synthesis with cyanobacteria.

## Methods

### Organism and culture conditions

Axenic cultures of the cyanobacterium *Synechocystis* sp. PCC 6803 were obtained from the Pasteur Culture Collection (Paris, France). All cultures were grown photoautotrophically under continuous illumination of 150 μmol photons/m^2^ s (warm white fluorescent tubes, Osram L 32) at 29 °C. High-density cultures (optical density at 750 nm—OD_750_ of approximately 2.0) were grown in BG11 [59] with different NaCl concentrations (ranging from 0 to 4 %) and bubbled with CO_2_-enriched air (5 %, *v/v*). Cultures with lower cell densities (OD_750_ of approximately 0.5) were grown in Erlenmeyer flasks in BG11 medium, which were shaken continuously at 120 rpm. For isoprene production studies, cultures were pre-cultivated at high CO_2_ in presence of different NaCl concentrations. After 24 h, the pre-cultures were used to inoculate the main cultures at OD_750_ of approximately 1 in 50 ml of BG11 with different NaCl concentrations. Isoprene production was induced by adding IPTG (1 mM final concentration) in the strains # 643 and # 704, in which *ispS* is under the control of P_*tac*-*lacI*_. During the cultivation in closed Schott flasks, which allow the sampling of the headspace via sampling ports in the closing caps, 50 mM of NaHCO_3_ as an inorganic carbon source was added to the medium. The cultures were incubated at 30 °C, with an illumination of approximately 150 µmol photons/m^2^ s under continuous stirring at 150 rpm. After 24 h, samples of 500 µl of the head space were taken and injected manually in the GC–MS system. After analyzing the isoprene amounts, samples for GG determination and transcriptomic and metabolomic analyses were taken.

### Growth analysis and pigment determination

Growth curves of *Synechocystis* wild type and the isoprene-producing strains were recorded over 24 h of incubation. The optical density of the culture was determined at 720 nm. To show the correlation of the optical density to the dry cell biomass, 5–10 ml of the culture was collected by filtration on MF Nitrocellulose Membrane Filters (0.45 µm) (Millipore, Darmstadt, Germany). Each sample was dried at 90 °C for 12 h and the dry cell weight was measured.

The chlorophyll *a,* phycocyanin, and carotinoide values were measured spectrophotometrically. These values were corrected according to Sigalat/de Kuckowski [60] and the chlorophyll *a*/phycocyanin and chlorophyll *a*/Carotinoide ratio was determined.

### Synthesis of codon-optimized *ispS* gene

The isoprene synthase (*ispS*) cDNA sequence of *Pueraria montana* (kudzu vine) was obtained from the NCBI database (Acc. No. AY315652). To ensure efficient expression of the plant cDNA in the cyanobacterial host, the codon usage was adopted to that of *Synechocystis*. Rare codons in the kudzu *ispS* sequence, i.e., codon-usage frequency below 10 % in *Synechocystis*, were changed to more frequently used codons. The chloroplast import sequence was removed from the *ispS* gene. The optimized *ispS* sequence is shown in Additional file 2. The optimized *ispS* coding sequence flanked by the engineered P_psaA*_ promoter upstream as well as the *oop* terminator downstream was obtained via gene synthesis service (GeneArt^®^ Gene Synthesis, Life Technologies).

### Plasmid construction and conjugation of *Synechocystis* with the isoprene synthase gene

The synthetic P_psaA*_-ispS-oop DNA fragment contained a *Sal*I restriction site upstream and *Pst*I site downstream, which facilitated subsequent insertion into the shuttle vector pVZ325. In addition, an *Nde*I restriction site overlapping with the start codon of *ispS* gene was inserted, which enabled subsequent promoter swaps. The synthetic DNA fragment was provided in a standard cloning vector, pMA (GeneArt^®^ Gene Synthesis, Life Technologies). The P_psaA*_-ispS-oop fragment was excised from the pMA vector via *Sal*I/*Pst*I digestion and then cloned into the *Sal*I/*Pst*I-cut pVZ325 vector (Additional file 3). To assess the *ispS* expression under different promoters, the psaA* promoter sequence was removed by *Sal*I/*Nde*I and replaced by alternative promoter fragments with compatible cohesive ends. The *rbcL* promoter was obtained from *Synechocystis* and includes the native upstream region −260 to +1 bp relative to the *rbcL* start codon, while for the *psbA2* promoter, the upstream region was chosen from −559 to +1 bp relative to the *psbA2* start codon. The P_tac/lacI_ promoter was amplified by PCR from the *E. coli* cloning vector pGEX-6K-1 (Acc.Nr. U78872.1) and encompassed a 2142 bp DNA fragment that also contains the *lacI* repressor gene and the LacI-binding operator region of P*tac*. The *dxs* gene was PCR-amplified from *Synechocystis* genomic DNA (*sll1945*). The 1923 bp DNA sequence for DXS was fused upstream with the *psbA2* or the *rbcL* promoter via *Nde*I, and the *oop* terminator sequence was added downstream of the *dxs* stop codon. Respective *dxs* expression cassettes were cloned into pVZ325a via *Sal*I/*Xma*I. pVZ325 derivative plasmids harboring an *ispS* expression cassette were transferred into *Synechocystis* cells by conjugation according to Zinchenko et al. [61]. Exconjugants were selected on BG11 agar plates containing 10 μg/ml gentamycin.

### RNA isolation

*Synechocystis* 6803 cells were collected by centrifugation (4000 rpm, 4 °C, 4 min), and the cells were suspended in 500 µl PGTX solution [62] [39.6 % (*w/v*) phenol, 7 % (*v/v*) glycerol, 7 mM 8-hydroxyquinoline, 20 mM EDTA, 97.5 mM sodium acetate, 0.8 M guanidine thiocyanate, 0.48 M guanidine hydrochloride]. The suspensions were incubated for 15 min at 65 °C and then incubated on ice for 5 min. After addition of 500 μl chloroform/isoamyl alcohol (24:1), samples were incubated at room temperature for 10 min and centrifuged at 6000 rpm at 20 °C for 10 min. The upper aqueous phase was transferred into a new tube, and the same volume of chloroform/isoamyl alcohol (24:1) was added. After mixing, samples were centrifuged as described above, and the aqueous phase was removed again and combined with an equal volume of isopropanol. After gently inverting the tube, RNA was precipitated overnight at −20 °C. RNA was pelleted through centrifugation (13,000 rpm, 4 °C, 30 min). The pellet was washed with 1 ml of 70 % ethanol (13,000 rpm, 20 °C, 5 min), allowed to air dry for approximately 10 min and resuspended in 30 μl RNase-free distilled water.

### cDNA synthesis, semi-quantitative RT-PCR and qRT-PCR

DNA-free RNA was reverse transcribed into cDNA using RevertAid H Minus reverse transcriptase (Fermentas, St. Leon-Rot, Germany) according to the manufacturer’s protocol. Before RT-PCR analysis, cDNA amounts were calibrated using the constitutively expressed *rnpB* gene. RT-PCR of *ispS* (primer sequences in Additional file 9) was performed using the Biometra Personal Cycler and PCR Master Mix (Qiagen) as described previously in more detail [63].

Calibrated cDNA was also used for qPCR analysis using the LightCycler 1.5 system (Roche, Basel, Switzerland) and SYBR Green fluorescence (Roche) for detection. To normalize gene expression, the constitutively expressed reference gene was amplified, and the average cycle threshold at each time point (*n* = 3) was used to calculate relative expression values. The expression of the selected genes at the different NaCl concentrations was normalized by subtraction of their cycle threshold values from the mean of the control gene, setting the respective value at 0 mM NaCl arbitrarily to 1.

### Transcriptomic analysis

A new high-resolution microarray was designed based on two recent RNAseq studies [64, 65]. RNA was directly labeled with the Kreatech ULS labeling kit for Agilent gene expression arrays with Cy3 according to the manufacturer’s protocol. Fragmentation and hybridization were performed following the manufacturer’s instructions for Agilent one-color microarrays. Feature extraction was performed with the Agilent “feature extraction” software and protocol “GE1_107_Sep09.” The raw data were analyzed with the R package limma [66]. Raw data were normexp background subtracted and quantile normalized. All probes of one RNA feature were summarized, and control features were deleted. *p* values were adjusted for multiple testing after using the Benjamini–Hochberg procedure. The threshold for significant differentially expressed genes was log_2_ fold change ≥ 1 and adjusted *p* value ≤ 0.05. The data have been deposited in the GEO database under the accession number GSE74940.

### Protein extraction and immune-blotting

For Western Blot analysis, cyanobacterial cells were collected by centrifugation (4000 rpm, 4 °C, 4 min), and suspended in Tris–EDTA-NaCl (TEN) buffer (50 mM Tris–HCl, pH 8; 5 mM EDTA, 100 mM NaCl) with addition of 100 μM PMSF. Cells were disrupted by sonication and insoluble material was removed by centrifugation. The supernatant was collected as the soluble protein fraction and the protein concentration was determined by Bradford [67]. The protein samples were separated by 12 % SDS-PAGE and transferred to a PVDF membrane (GE Healthcare, Freiburg, Germany). For immunodetection, the rabbit serum-containing specific polyclonal antibodies against IspS [4] and horseradish peroxidase-conjugated secondary antibodies were used. Peroxidase activity was detected by chemiluminescence.

### Analysis of low molecular mass organic solutes

Low molecular mass solutes were extracted from freeze-dried cell pellets with 80 % ethanol (HPLC grade, Roth, Germany) at 68 °C for 2 h. For GC analysis, a defined amount of sorbitol was added as an internal standard. The extracts were centrifuged (13,000*g*, 5 min, 20 °C) and the supernatant was lyophilized. The dry extract was resuspended in 500 μl ethanol (99 % HPLC grade, Roth, Germany) and centrifuged. The subsequent supernatant was again dried and then resuspended in 500 μl deionized water (HPLC grade, Carl Roth, Karlsruhe, Germany). After drying, the final extract was dissolved in pyridine, silylated, and analyzed by gas chromatography (GC) according to Hagemann et al. [68].

### GC—MS analyses of isoprene

GC—MS analyses were performed using a GC—MS-QP 5000 (Shimadzu) comprising a Tri Plus auto-sampler. Analytes were ionized by an electrospray ionization (ESI) system, which operated in electron impact mode with ionization energy of 70 eV. Helium gas (99.999 %) was used as a carrier gas at a constant flow rate of 75 ml/min, and an injection volume of 0.5 μl was employed (split-injection). The injector temperature was maintained at 150 °C, the ion-source temperature was 180 °C, and the oven temperature was programmed from 135 °C (isothermal). Mass spectra were taken at 70 eV in a full scan mode and for fragments from 50 to 280 *m/z*. The mass-detector used in this analysis was Turbo-Mass Gold-Perkin-Elmer, and the software used to handle mass spectra and chromatograms was a GC—MS solution system 1.2.

### Single photon ionization time-of-flight mass spectrometry (SPI-MS)

SPI-MS has already been shown to be well suited for the fast, time resolved, online analysis of coffee roasting products [69, 70], cigarette smoke [71, 72], and waste incineration plant fumes [73, 74]. For isoprene production studies using the SPI-MS, cultures were pre-cultivated at high CO_2_ to an optical density (OD_750_) of approximately two. Then, cultures were supplemented with 50 mM NaHCO_3_ and shifted to different culturing conditions [dark, high light, salt (NaCl) etc.]. The cultures were maintained in hybridization vessels (Glasgerätebau Ochs GmbH, Bovenden-Lenglern) equipped with silicon septa at an ambient temperature of 30 °C. By using deactivated gas chromatography capillaries (TSP-fused silica deactivated with DPTMDS, ID 150 μm, OD 375 μm; BGB, Rheinfelden), a stream of compressed air with a constant flow rate of 10 ml/min was maintained. The sample inlet was a metal capillary (Hydroguard MXT, ID 0.28 mm; Restek, Bad Homburg) placed in the center of the septum as well as the upper part of the cultivation vessel gas compartment. The capillary ran through a heatable transfer line (length 2.0 m), which was constantly heated to 220 °C. Its end was aligned with the tip of an also heated, hollow, stainless steel needle, which was pointed to the center of the ion source.

For ionization, UV light was generated by frequency tripling of the given Nd:YAG laser (Surelite III, Continuum, Santa Clara, USA) signal (wavelength 1064 nm, pulse duration 5 ns, repetition rate 10 Hz). As a consequence of repeated frequency tripling of the UV laser pulse within a xenon-filled collision cell, VUV photons with a wavelength of exactly 118 nm, which is equivalent to energy of 10.49 eV, were generated. For a detailed description of the formation process, see Mühlberger et al. [75]. The given photons are transferred to the ionization chamber, focused on the inlet needle and absorbed by gaseous (analyte) molecules. When the ionization energy (IE) of these is exceeded, ions are produced. Therefore, all species with an IE below 10.49 eV, most organic compounds, are accessible, and as a positive side effect, signals originating from matrices, such as oxygen (IE 12.06 eV), nitrogen (IE 15.58 eV), or water (IE 12.62 eV), are suppressed. Transferring only low excess energy, the soft ionization process, leads to an inhibited fragmentation and less complex spectra and facilitates rapid data interpretation.

After ionization, a time-of-flight mass analyzer, which is capable of separating a large amount of ions in very short time intervals, is the next step. In principle, the separation is based on temporal differences of ions with various *m/z* values traveling along a field-free drift path, from ion source to detector. Therefore, ions are accelerated and equipped with a specific amount of kinetic energy. Depending on their *m/z* as well as the resulting velocities, the ions reach the detector at different times. In this case, the use of a reflector TOF analyzer additionally enhances the mass resolution due to a temporal focusing of ions with different kinetic energies. The detection unit is represented by a microchannel plate (MCP, 1.6–1.65 kV). The detailed experimental setup was described elsewhere [76].

Data acquisition was carried out by a LabVIEW routine (National Instruments, Austin, USA) based on custom-written software [77], whereby the spectra were recorded by two transient recorder cards (DP 210, Aquiris, Switzerland) with different gain settings and a signal resolution of eight bit. The processing was also performed by a LabVIEW routine, customized by Photonion GmbH (Schwerin, Germany). In particular, the data from both recorder cards were merged, while the threshold was set to 0.0006 up to 0.02 V, depending on the signal of a single ion event and the noise level. For converting the independent dimension ‘flight time’ into the crucial variable ‘*m/z’*, a standard gas mixture of 1,3-butadiene (concentration 10.20 ppm), acetone (9.58 ppm), isoprene (11.50 ppm), and styrol (9.69 ppm) from Linde (Oberschleißheim) was used. For each standard gas measurement, 150 successive single laser shots, in this case 150 spectra, were recorded and averaged, which equals a duration of 15 s. Using the known *m/z* for 1,3-butadiene and styrol as well as the resulting mass spectra, the flight time was transformed and the spectrum mass calibrated, respectively. Depending on the expected measuring time and data amount for each isoprene sample analysis, the number of recorded spectra was adjusted by presetting the average number for raw data recording (one stored spectra per 0.1 s up to per 10 s). The isoprene signal (*m/z* 68) was extracted from raw data as the peak area (a.u.) per given time period. For medium blank values (BG11), net 150 s and for culture samples, net 1500 s were averaged. For quantification, the resulting values were determined relative to those of the standard gas measurements (11.5 ppm).

### Metabolomic profiling analysis

Cyanobacteria were grown in liquid media in a closed flask system, in the presence of 50 mM NaHCO_3_. After 24 h, 10 ml of cells (OD_750_ of approximately 1.5) was harvested by fast filtration in the light and immediately frozen in liquid nitrogen. Metabolite profiles were determined by gas chromatography electron ionization time-of-flight mass spectrometry (GC-EI-TOF-MS) as described previously [33, 78, 79]. The extraction protocol was slightly modified to enable comparison of high and low salt (NaCl) samples. Frozen samples were incubated in 630 μl of precooled methanol and extracted for 1 h at 4 °C with a final 15 min extraction at 70 °C. After centrifugation, 500 µl of extract was transferred into a new microfuge tube, and 200 µl of chloroform and 200 µl of diethylamine were added. After a 5 min incubation at 37 °C, 500 µl of water was added for phase separation. After phase separation by centrifugation, 600 µl of the upper aqueous phase was dried in a speed vacuum concentrator and further processed for GC—MS measurements as was described previously [33, 78, 79]. Metabolite responses were calculated and normalized to an internal standard, U-^13^C-sorbitol, and biomass using the optical density at 750 nm (OD_750_) of each sample [33, 79]. In this study, relative changes of metabolite pools were routinely assessed as response ratios, i.e., as x-fold changes of metabolite pools of isoprene producers in comparison to the WT pools. All experiments were repeated using three independent cell cultures.

The means of biological repeats, standard errors, and heteroscedastic Student’s *t* test were calculated using Microsoft Excel. One-way analysis of variance (ANOVA) was performed using multi-experiment viewer software, MeV (Version 4.6.2; http://www.tm4.org/mev/; [80]).

## Additional files

10.1186/s13068-016-0503-4 Simplified network of the cyanobacterial metabolism. The MEP pathway including the isoprene synthase from *Pueraria montana* is highlighted in green. The MEP pathway starts from triose-phosphate and consumes reducing equivalents and cellular energy in the form of NADPH, reduced ferredoxin, CTP and ATP, ultimately derived from photosynthesis.
10.1186/s13068-016-0503-4 DNA and protein sequence of the codon-adapted cDNA of the *ispS* gene from *Pueraria montana*, which was used for the expression in *Synechocystis* sp. PCC 6803. Start and Stop codon of the gene sequence are highlighted.
10.1186/s13068-016-0503-4 Map of the autonomous vector pVZ325a [57], which was used to express the codon-adapted cDNA of the *ispS* gene from *Pueraria montana* for the expression in *Synechocystis* sp. PCC 6803. The synthetic P_*psaA**_-ispS-oop DNA fragment contained upstream a *Sal*I restriction site and downstream a *Pst*I site, which facilitated subsequent insertion into the shuttle vector pVZ325.
10.1186/s13068-016-0503-4 Gas chromatography (GC) and mass spectrometry (MS insert) analysis of the head space gas. The arrow marks the GC peak showing the retention time of isoprene in cells of the transformed *Synechocystis* strains. The MS analysis of this peak shows the typical pattern of fragmentation of the isoprene standard. The unknown peak was also found in wild type cells not transformed with the pVZ325a vector. Hence it doesn`t correlate with the expression of *ispS* nor with the occurrence of the shuttle vector.
10.1186/s13068-016-0503-4 Growth and pigmentation of the different isoprene-producing strains in comparison to the wild type in the presence of different NaCl concentrations. A: The pigmentation is expressed in ratios of chlorophyll *a* to phycocyanin and of chlorophyll *a* to carotinoide at different salt concentration ranging from 0 to 4 % NaCl. B: The growth rate of the wild type and the isoprene expressing strains showed a comparable slight decrease with increasing NaCl concentrations.
10.1186/s13068-016-0503-4 Complete data set of metabolic analysis.
10.1186/s13068-016-0503-4 Complete data set of transcriptomic analysis.
10.1186/s13068-016-0503-4 The genome plot shows the graphical overview of array signal intensities mapped along the *Synechocystis* chromosome. Both strands of the respective chromosomal regions are shown with the location of annotated protein-coding genes (blue boxes), antisense RNAs (red), and intergenic sRNA genes (yellow). The color-coded bars represent signals derived from individual microarray probes. The scale for the microarray signal intensities is given at the left y-axis. All probes for the same RNA feature are connected by lines.
10.1186/s13068-016-0503-4 List of primers used in this study for RT-PCR and qPCR.
10.1186/s13068-016-0503-4 Determination of the relation OD_750_ to dry cell weight (DCW). With the help of this correlation, the calculation of our isoprene values to the dry weight basis was achieved.

## Abbreviations

DMAPP: dimethylallyl diphosphate
DXS: 1-deoxy-d-xylulose 5-phosphate synthase
GC: gas chromatography
GG: glucosylglycerol
MEP: 2-C-methyl-d-erythritol 4-phosphate pathway
MVA: mevalonic acid pathway
OPP: oxidative pentose phosphate
WT: wild type

## References

[1] Rosgaard L, de Porcellinis AJ, Jacobsen JH, Frigaard NU, Sakuragi Y (2012). Bioengineering of carbon fixation, biofuels, and biochemicals in cyanobacteria and plants. J Biotechnol.

[2] Stanier RY, Cohen-Bazire G, Starr MP, Ingraham JL, Balows A (1977). Phototrophic prokaryotes: the cyanobacteria. Annual review of microbiology.

[3] Melis A (2009). Solar energy conversion efficiencies in photosynthesis: minimizing the chlorophyll antenna to maximize efficiency. Plant Sci.

[4] Lindberg P, Park S, Melis A (2010). Engineering a platform for photosynthetic isoprene production in cyanobacteria, using *Synechocystis* as the model organism. Met Engin.

[5] Bentley FK, Melis A (2012). Diffusion-based process for carbon dioxide uptake and isoprene emission in gaseous/aqueous two-phase photobioreactors by photosynthetic microorganisms. Biotechnol Bioeng.

[6] Bentley FK, Zurbriggen A, Melis A (2014). Heterologous expression of the mevalonic acid pathway in cyanobacteria enhances endogenous carbon partitioning to isoprene. Mol Plant.

[7] Chaves JE, Kirst H, Melis A (2014). Isoprene production in *Synechocystis* under alkaline and saline growth conditions. J Appl Phycol.

[8] Deng MD, Coleman JR (1999). Ethanol synthesis by genetic engineering in cyanobacteria. Appl Environ Microbiol.

[9] Varman AM, Xiao Y, Pakrasi HB, Tang YJ (2013). Metabolic engineering of *Synechocystis* sp. strain PCC 6803 for isobutanol production. Appl Environ Microbiol.

[10] Sakai M, Ogawa T, Matsuoka M, Fukuda H (1997). Photosynthetic conversion of carbon dioxide to ethylene by the recombinant cyanobacterium, *Synechococcus* sp. PCC 7942, which harbors a gene for the ethylene-forming enzyme of *Pseudomonas syringae*. J Ferment Bioeng.

[11] Takahama K, Matsuoka M, Nagahama K, Ogawa T (2003). Construction and analysis of a recombinant cyanobacterium expressing a chromosomally inserted gene for an ethylene-forming enzyme at the *psbAI* locus. J Biosci Bioeng.

[12] Lan EI, Liao JC (2012). ATP drives direct photosynthetic production of 1-butanol in cyanobacteria. Proc Natl Acad Sci USA.

[13] Zhou J, Zhang HF, Zhang YP, Li Y, Ma YH (2012). Designing and creating a modularized synthetic pathway in cyanobacterium *Synechocystis* enables production of acetone from carbon dioxide. Metab Eng.

[14] Kusakabe T, Tatsuke T, Tsuruno K, Hirokawa Y, Atsumi S, Liao JC, Hanai T (2013). Engineering a synthetic pathway in cyanobacteria for isopropanol production directly from carbon dioxide and light. Metab Eng.

[15] Wang W, Liu X, Lu X (2013). Engineering cyanobacteria to improve photosynthetic production of alka(e)nes. Biotechnol Biofuels.

[16] Ducat DC, Avelar-Rivas JA, Way JC, Silver PA (2012). Rerouting carbon flux to enhance photosynthetic productivity. Appl Environ Microbiol.

[17] Kiyota H, Okuda Y, Ito M, Hirai MY, Ikeuchi M (2014). Engineering of cyanobacteria for the photosynthetic production of limonene from CO_2_. J Biotechnol.

[18] Davies FK, Work VH, Beliaev AS, Posewitz MC (2014). Engineering limonene and bisabolene production in wild type and a glycogen-deficient mutant of *Synechococcus* sp. PCC 7002. Front Bioeng Biotechnol.

[19] Greve HH, Rubber. Natural. In: Ullmann’s encyclopedia of industrial chemistry. Weinheim: Wiley-VCH. 2000. doi:10.1002/14356007.a23_225.

[20] Ruzicka L (1953). The isoprene rule and the biogenesis of terpenic compounds. Experientia.

[21] Sharkey TD, Yeh S (2001). Isoprene emission from plants. Annu Rev Plant Physiol Plant Molec Biol.

[22] Kuzma J, Nemecek-Marshall N, Pollock WH, Fall R (1995). Bacteria produce the volatile hydrocarbon isoprene. Curr Microbiol.

[23] Fischer CR, Klein-Marcuschamer D, Stephanopoulos G (2008). Selection and optimization of microbial hosts for biofuels production. Metab Eng.

[24] Stephanopoulos G (2007). Challenges in engineering microbes for biofuels production. Science.

[25] Lange BM, Rujan T, Martin W, Croteau R (2000). Isoprenoid biosynthesis: the evolution of two ancient and distinct pathways across genomes. Proc Natl Acad Sci USA.

[26] Lichtenthaler HK (2000). Sterols and isoprenoids. Biochem Soc Trans.

[27] Eisenreich W, Rohdich F, Bacher A (2001). Deoxyxylulose phosphate pathway to terpenoids. Trends Plant Sci.

[28] Rohmer M, Barton D, Nakanishi K (1999). A mevalonate-independent route to isopentenyl diphosphate. Comprehensive natural products chemistry.

[29] Wolfertz M, Sharkey TD, Boland W, Kühnemann F (2004). Rapid regulation of the methylerythritol 4-phosphate pathway during isoprene synthesis. Plant Physiol.

[30] Nowicka B, Kruk J (2010). Occurrence; biosynthesis and function of isoprenoid quinines. Biochem Biophys Acta.

[31] Chisti Y (2013). Constraints to commercialization of algal fuels. J Biotechnol.

[32] Pade N, Hagemann M (2014). Salt acclimation of cyanobacteria and their application in biotechnology. Life.

[33] Eisenhut M, Huege J, Schwarz D, Bauwe H, Kopka J, Hagemann M (2008). Metabolome phenotyping of inorganic carbon limitation in cells of wild type and photorespiratory mutants of the cyanobacterium *Synechocystis* sp. strain PCC 6803. Plant Physiol.

[34] Yoshimura H, Yanagisawa S, Kanehisa M, Ohmori M (2002). Screening for the target gene of cyanobacterial cAMP receptor protein SYCRP1. Mol Microbiol.

[35] Xu M, Su Z (2009). Computational prediction of cAMP receptor protein (CRP) binding sites in cyanobacterial genomes. BMC Genom.

[36] Georg J, Dienst D, Schürgers N, Wallner T, Kopp D, Stazic D, Kuchmina E, Klähn S, Lokstein H, Hess WR, Wilde A (2014). The small regulatory RNA SyR1/PsrR1 controls photosynthetic functions in cyanobacteria. Plant Cell.

[37] Zhang L, Mc Spadden B, Pakrasi HB, Whitmarsh J (1992). Copper mediated regulation of cytochrome c553 and plastocyanin in the cyanobacterium *Synechocystis* 6803. J Biol Chem.

[38] Durán RV, Hervás M, La De, Rosa MA, Navarro JA (2004). The efficient functioning of photosynthesis and respiration in *Synechocystis* sp. PCC 6803 strictly requires the presence of either cytochrome c6 or plastocyanin. J Biol Chem.

[39] Klähn S, Schaal C, Georg J, Baumgartner D, Knippen G, Hagemann M, Muro-Pastor AM, Hess WR (2015). The sRNA NsiR4 is involved in nitrogen assimilation control in cyanobacteria by targeting glutamine synthetase inactivating factor IF7. Proc Natl Acad Sci.

[40] Lee SW, Lee HW, Chung HJ, Kim YA, Kim YJ, Hahn Y, Chung JH, Park YS (1999). Identification of the genes encoding enzymes involved in the early biosynthetic pathway of pteridines in *Synechocystis* sp. PCC 6803. FEMS Microbiol Lett.

[41] Angermayr SA, Hellingwerf KJ, Lindblad P, de Mattos MJ (2009). Energy biotechnology with cyanobacteria. Curr Opin Biotechnol.

[42] Zhou J, Zhang H, Meng H, Zhu Y, Bao G, Zhang Y, Li Y, Ma Y (2014). Discovery of a super-strong promoter enables efficient production of heterologous proteins in cyanobacteria. Sci Rep.

[43] Formighieri C, Melis A (2014). Regulation of β-phellandrene synthase gene expression, recombinant protein accumulation, and monoterpene hydrocarbons production in *Synechocystis* transformants. Planta.

[44] Cunningham FX, Lafond TP, Gantt E (2000). Evidence of a role for LytB in the nonmevalonate pathway of isoprenoid biosynthesis. J Bacteriol.

[45] Harker M, Bramley PM (1999). Expression of prokaryotic-1-deoxy-d-xylulose-5-phosphatases in *Escherichia coli* increases carotenoid and ubiquinone biosynthesis. FEBS Lett.

[46] Kilian J, Whitehead D, Horak J, Wanke D, Weinl S, Batistic O, D’Angelo C, Bornberg-Bauer E, Kudla J, Harter K (2007). The AtGenExpress global stress expression data set: protocols, evaluation and model data analysis of UV-B light, drought and cold stress responses. Plant J.

[47] Montero O, Sánchez-Guijo A, Lubián LM, Martínez-Rodríguez G (2012). Changes in membrane lipids and carotenoids during light acclimation in a marine cyanobacterium *Synechococcus* sp.. J Biosci.

[48] Kim SW, Keasling JD (2001). Metabolic engineering of the nonmevalonate isopentenyl diphosphate synthesis pathway in *Escherichia coli* enhances lycopene production. Biotechnol Bioeng.

[49] Ajikumar PK, Xiao WH, Tyo KE, Wang Y, Simeon F, Leonard E, Mucha O, Phon TH, Pfeifer B, Stephanopoulos G (2010). Isoprenoid pathway optimization for Taxol precursor overproduction in *Escherichia coli*. Science.

[50] Davies FK, Jinkerson RE, Posewitz MC (2015). Toward a photosynthetic microbial platform for terpenoid engineering. Photosynth Res..

[51] Brüggemann N, Schnitzler JP (2002). Comparison of isoprene emission, intercellular isoprene concentration and photosynthetic performance in water-limited oak (*Quercus pubescens* Willd. and *Quercus robur* L.) saplings. Plant Biol.

[52] Wolfertz M, Sharkey TD, Boland W, Kühnemann F, Yeh S, Weise SE (2003). Biochemical regulation of isoprene emission. Plant Cell Environ.

[53] Sun Z, Cunningham FX, Gantt E (1998). Differential expression of two isopentenyl pyrophosphate isomerases and enhanced carotenoid accumulation in a unicellular chlorophyte. Proc Natl Acad Sci USA.

[54] Hagemann M (2011). Molecular biology of cyanobacterial salt acclimation. FEMS Microbiol Rev.

[55] Poliquin K, Ershov YV, Cunningham FX, Woreta TT, Gantt RR, Gantt E (2004). Inactivation of *sll1556* in *Synechocystis* strain PCC 6803 impairs isoprenoid biosynthesis from pentose phosphate cycle substrates in vitro. J Bacteriol.

[56] Ershov YV, Gantt RR, Cunningham FX, Gant E (2002). Isoprenoid biosynthesis in *Synechocystis* sp. Strain PCC6803 is stimulated by compounds of the pentose phosphate cycle but not by pyruvate or deoxyxylulose-5-phosphate. J Bacteriol.

[57] Los DA, Suzuki I, Zinchenko VV, Murata N, Herrero A, Flores E (2008). Stress responses in *Synechocystis*: Regulated genes and regulatory systems. The cyanobacteria-molecular biology, genomics and evolution.

[58] Noguchi S, Putri SP, Lan EI, Lavina WA, Dempo Y, Bamba T, Liao JC, Fukusaki E (2016). Quantitative target analysis and kinetic profiling of acyl-CoAs reveal the rate-limiting step in cyanobacterial 1-butanol production. Metabolomics.

[59] Rippka R, Deruelles J, Waterbury JB, Herdman M, Stanier RY (1979). Generic assignments, strain histories and properties of pure cultures of cyanobacteria. J Gen Microbiol.

[60] Sigalat C, de Kouchowski Y. Preparations and properties of the photosynthetic fragments of the unicellular blue-green alga *Anacystis nidulans*. In: Avron M, editors. Proceedings of the third international congress on photosynthesis. Amsterdam: Elsevier; 1975. p. 621–27.

[61] Zinchenko VV, Piven IV, Melnik VA, Shestakov SV (1999). Vectors for the complementation analysis of the cyanobacterial mutants. Russian J Genet.

[62] Pinto FL, Thapper A, Sontheim W, Lindblad P (2009). Analysis of current and alternative phenol based RNA extraction methodologies for cyanobacteria. BMC Mol Biol.

[63] Klähn S, Steglich C, Hess WR, Hagemann M (2010). Glucosylglycerate: a secondary compatible solute common to marine cyanobacteria from nitrogen-poor environments. Environ Microbiol.

[64] Kopf M, Klähn S, Scholz I, Matthiessen JKF, Hess WR, Voß B (2014). Comparative analysis of the primary transcriptome of *Synechocystis* sp. PCC 6803. DNA Res Int J Rapid Publ Rep Genes Genomes.

[65] Mitschke J, Georg J, Scholz I, Sharma CM, Dienst D, Bantscheff J, Voss B, Steglich C, Wilde A, Vogel J, Hess WR (2011). An experimentally anchored map of transcriptional start sites in the model cyanobacterium *Synechocystis* sp. PCC6803. Proc Natl Acad Sci USA.

[66] Ritchie ME, Phipson B, Wu D, Hu Y, Law CW, Shi W, Smyth GK (2015). limma powers differential expression analyses for RNA-sequencing and microarray studies. Nucl Acid Res.

[67] Bradford MM (1976). A rapid and sensitive method for the quantitation of microgram quantities of protein utilizing the principle of protein-dye binding. Anal Biochem.

[68] Hagemann M, Ribbeck-Busch K, Klähn S, Hasse D, Steinbruch R, Berg G (2008). The plant-associated bacterium *Stenotrophomonas rhizophila* expresses a new enzyme for the synthesis of the compatible solute glucosylglycerol. J Bacteriol.

[69] Dorfner R, Ferge T, Yeretzian C, Kettrup A, Zimmermann R (2004). Laser mass spectrometry as on-line sensor for industrial process analysis: process control of coffee roasting. Anal Chem.

[70] Hertz-Schünemann R, Streibel T, Ehlert S, Zimmermann R (2013). Looking into individual coffee beans during the roasting process: direct micro-probe sampling on-line photo ionization mass spectrometric analysis of coffee roasting gases. Anal Bioanal Chem.

[71] Adam T, Baker RR, Zimmermann R (2007). Investigation, by single photon ionisation (SPI)-time-of-flight mass spectrometry (TOFMS), of the effect of different cigarette-lighting devices on the chemical composition of the first cigarette puff. Anal Bioanal Chem.

[72] Busch C, Streibel T, Liu C, McAdam KG, Zimmermann R (2012). Pyrolysis and combustion of tobacco in a cigarette smoking simulator under air and nitrogen atmosphere. Anal Bioanal Chem.

[73] Kuribayashi S, Yamakoshi H, Danno M, Sakai S, Tsuruga S, Futami H, Morii S (2005). VUV single-photon ionization ion trap time-of-flight mass spectrometer for on-line, real-time monitoring of chlorinated organic compounds in waste incineration flue gas. Anal Chem.

[74] Streibel T, Hafner K, Mühlberger F, Adam T, Warnecke R, Zimmermann R (2006). Investigation of NO_x_ precursor compounds and other combustion by-products in the primary combustion zone of a waste-incineration plant using on-line, real-time mass spectrometry and Fourier-transform infrared spectrometry (FTIR). Anal Bioanal Chem.

[75] Mühlberger F. Entwicklung von on-line-Analyseverfahren auf Basis der Einphotonenionisations-Massenspektrometrie, PhD Thesis. Munich: Technische Universität München. 2003.

[76] Adam T, Zimmermann R (2007). Determination of single photon ionization cross sections for quantitative analysis of complex organic mixtures. Anal Bioanal Chem.

[77] Erban A, Schauer N, Fernie AR, Kopka J, Weckwerth W (2007). Non-supervised construction and application of mass spectral and retention time index libraries from time-of-flight GC-MS metabolite profiles: methods in molecular biology. Metabolomics: Methods and protocols.

[78] Dethloff F, Erban A, Orf I, Alpers J, Fehrle I, Beine-Golovchuk O, Schmidt S, Schwachtje J, Kopka J (2014). Profiling methods to identify cold regulated primary metabolites using gas chromatography coupled to mass spectrometry. Methods in Molecular Biology.

[79] Huege J, Goetze J, Schwarz D, Bauwe H, Hagemann M, Kopka J (2011). Modulation of the major paths of carbon in photorespiratory mutants of *Synechocystis*. PLoS One.

[80] Saeed AI, Sharov V, White J, Li J, Liang W, Bhagabati N, Braisted J, Klapa M, Currier T, Thiagarajan M, Sturn A, Snuffin M, Rezantsev A, Popov D, Ryltsov A, Kostukovich E, Borisovsky I, Liu Z, Vinsavich A, Trush V, Quackenbush J (2003). TM4: a free, open-source system for microarray data management and analysis. Biotechniques.

