# Supplementary figures and images for: Insights into isoprene production using the cyanobacterium Synechocystis sp. PCC 6803
Source: Biotechnol Biofuels. 2016 Apr 18;9:89. doi: 10.1186/s13068-016-0503-4 (PMC4836186; doi:10.1186/s13068-016-0503-4)

## Slide 1
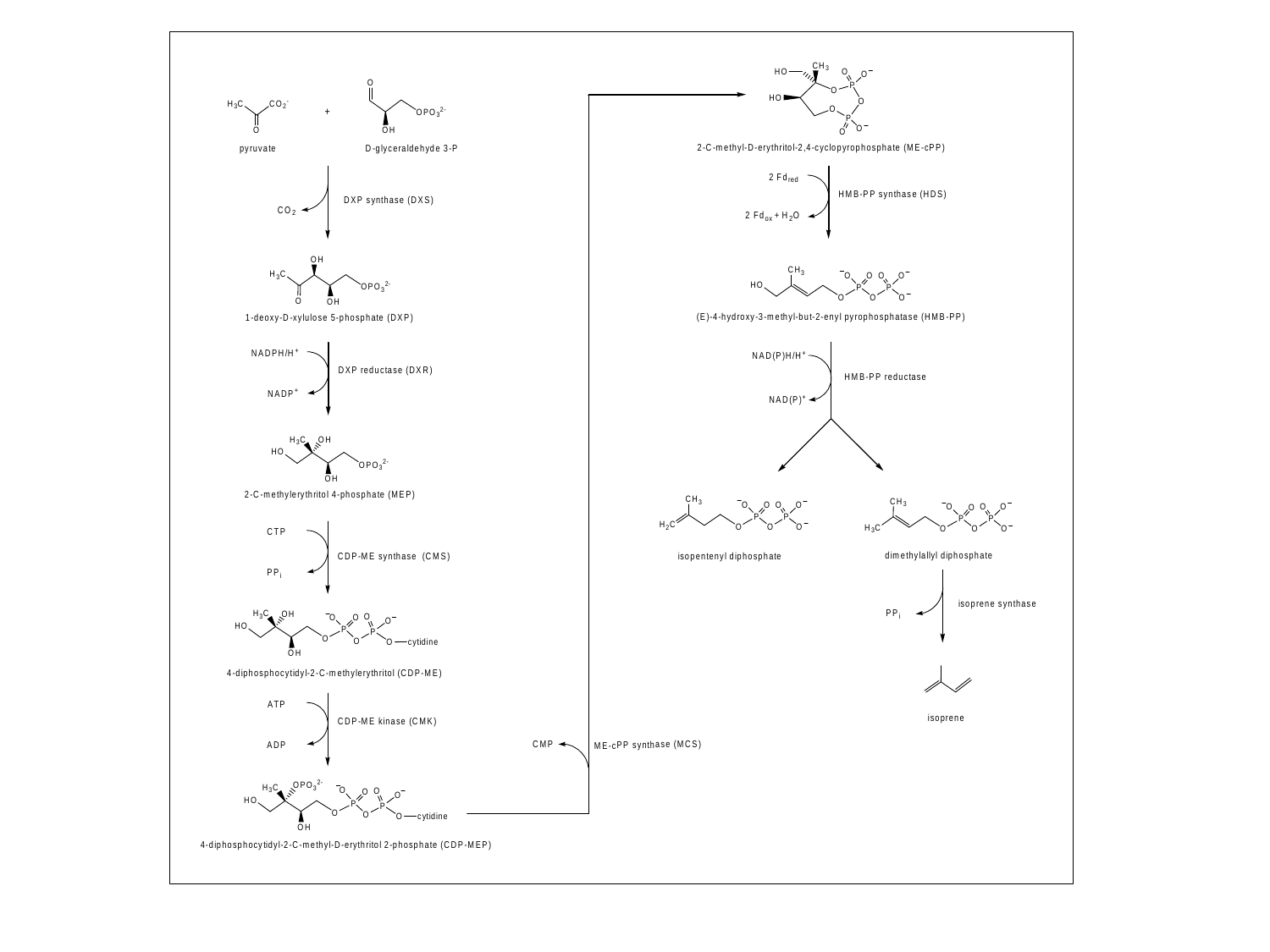

Supplement: Supplementary file 1 — 10.1186/s13068-016-0503-4 Simplified network of the cyanobacterial metabolism. The MEP pathway including the isoprene synthase from Pueraria montana is highlighted in green. The MEP pathway starts from triose-phosphate and consumes reducing equivalents and cellular energy in the form of NADPH, reduced ferredoxin, CTP and ATP, ultimately derived from photosynthesis. [file 13068_2016_503_MOESM1_ESM.pptx]

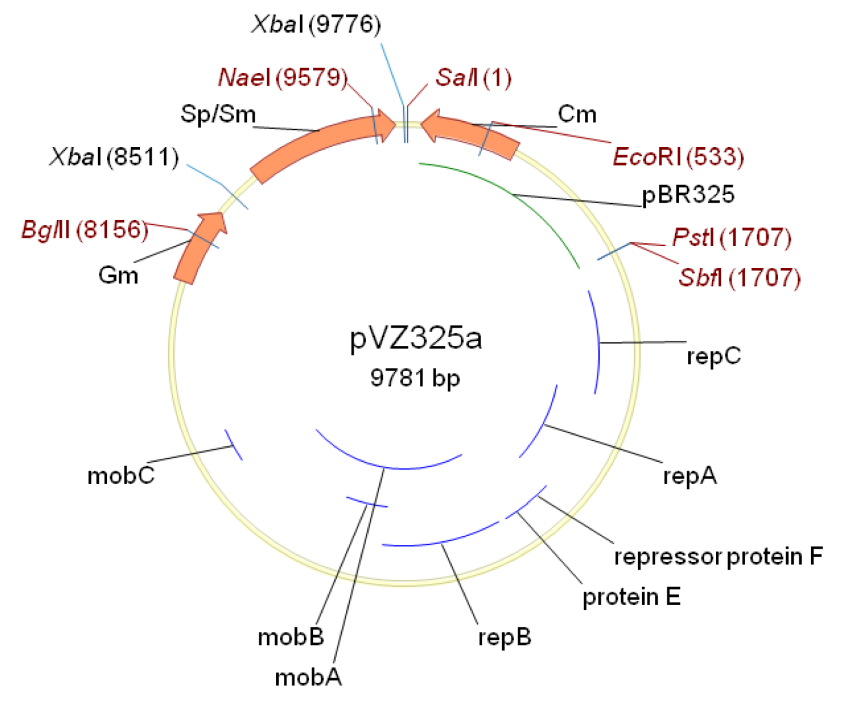

Supplement: Supplementary file 3 — 10.1186/s13068-016-0503-4 Map of the autonomous vector pVZ325a [57], which was used to express the codon-adapted cDNA of the ispS gene from Pueraria montana for the expression in Synechocystis sp. PCC 6803. The synthetic PpsaA*-ispS-oop DNA fragment contained upstream a SalI restriction site and downstream a PstI site, which facilitated subsequent insertion into the shuttle vector pVZ325. [file 13068_2016_503_MOESM3_ESM.docx]

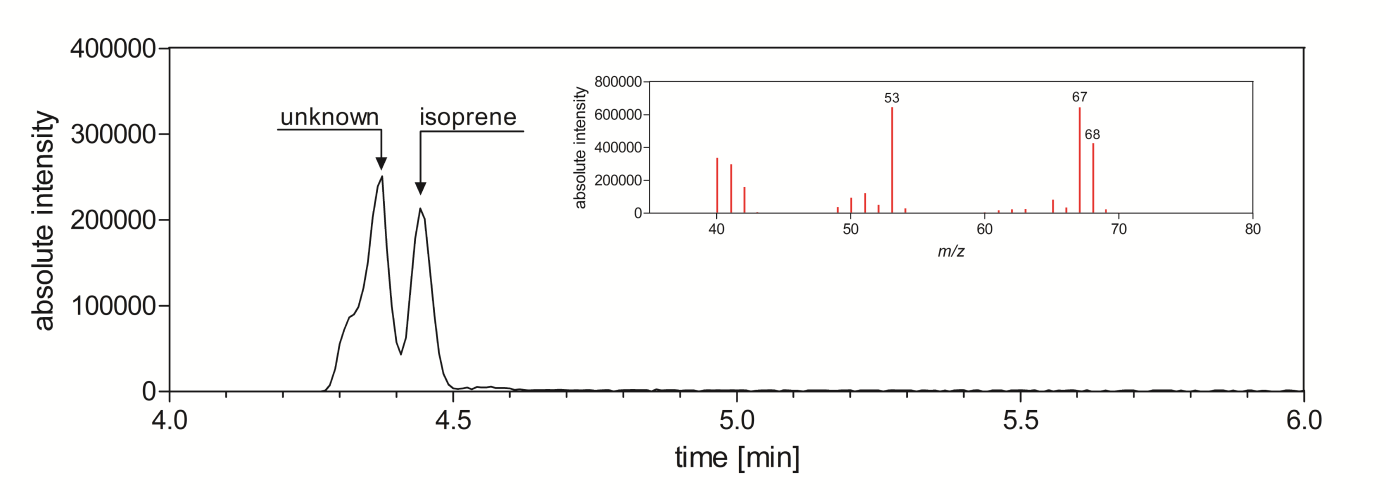

Supplement: Supplementary file 4 — 10.1186/s13068-016-0503-4 Gas chromatography (GC) and mass spectrometry (MS insert) analysis of the head space gas. The arrow marks the GC peak showing the retention time of isoprene in cells of the transformed Synechocystis strains. The MS analysis of this peak shows the typical pattern of fragmentation of the isoprene standard. The unknown peak was also found in wild type cells not transformed with the pVZ325a vector. Hence it doesn`t correlate with the expression of ispS nor with the occurrence of the shuttle vector. [file 13068_2016_503_MOESM4_ESM.docx]

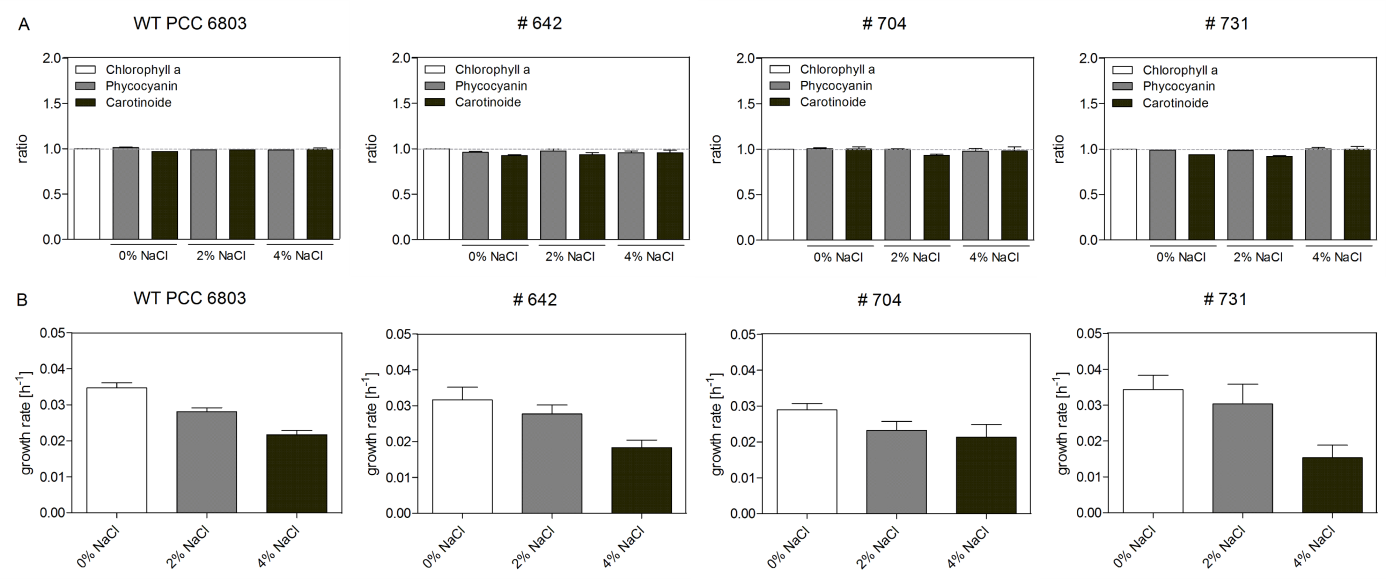

Supplement: Supplementary file 5 — 10.1186/s13068-016-0503-4 Growth and pigmentation of the different isoprene-producing strains in comparison to the wild type in the presence of different NaCl concentrations. A: The pigmentation is expressed in ratios of chlorophyll a to phycocyanin and of chlorophyll a to carotinoide at different salt concentration ranging from 0 to 4 % NaCl. B: The growth rate of the wild type and the isoprene expressing strains showed a comparable slight decrease with increasing NaCl concentrations. [file 13068_2016_503_MOESM5_ESM.docx]

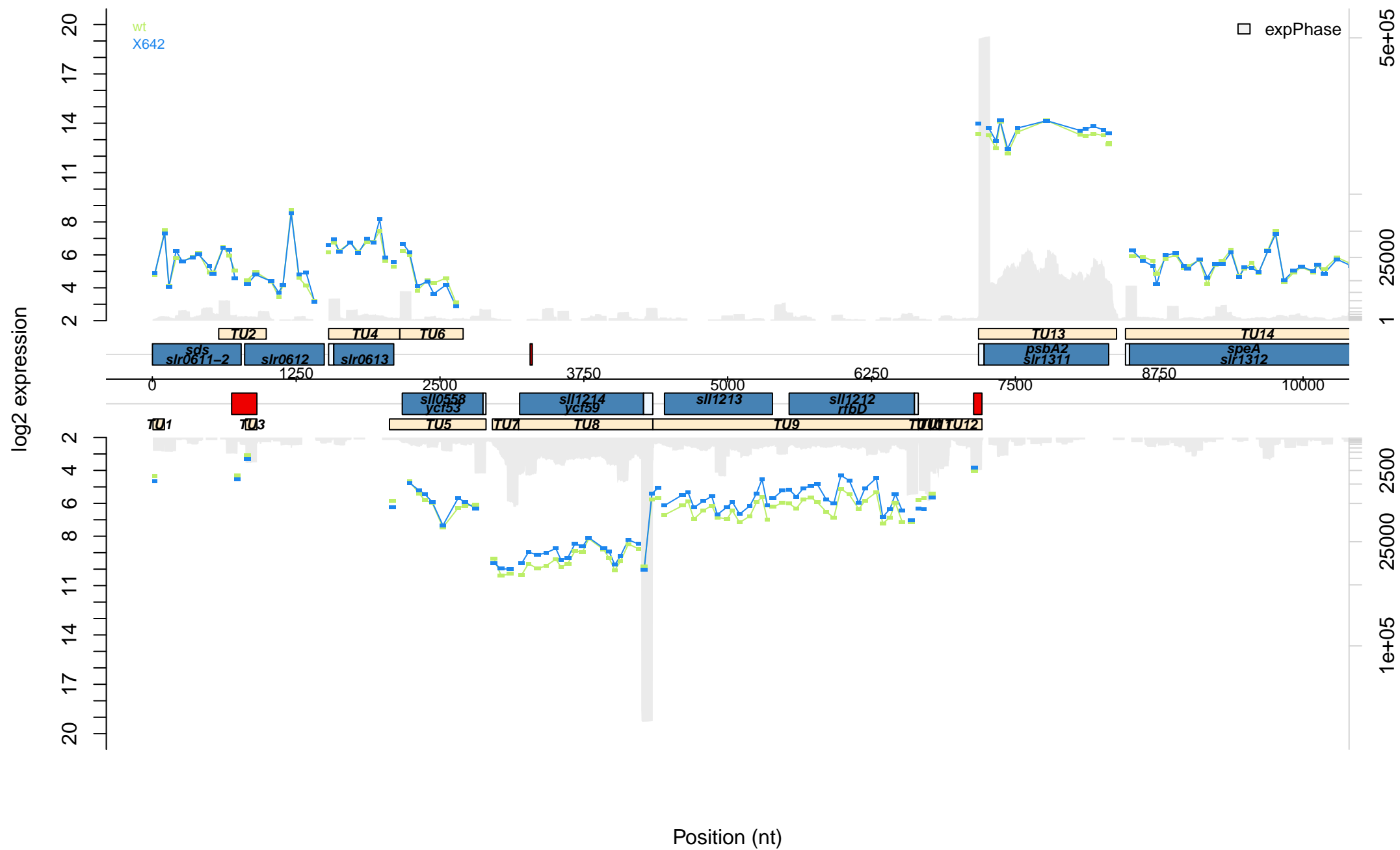

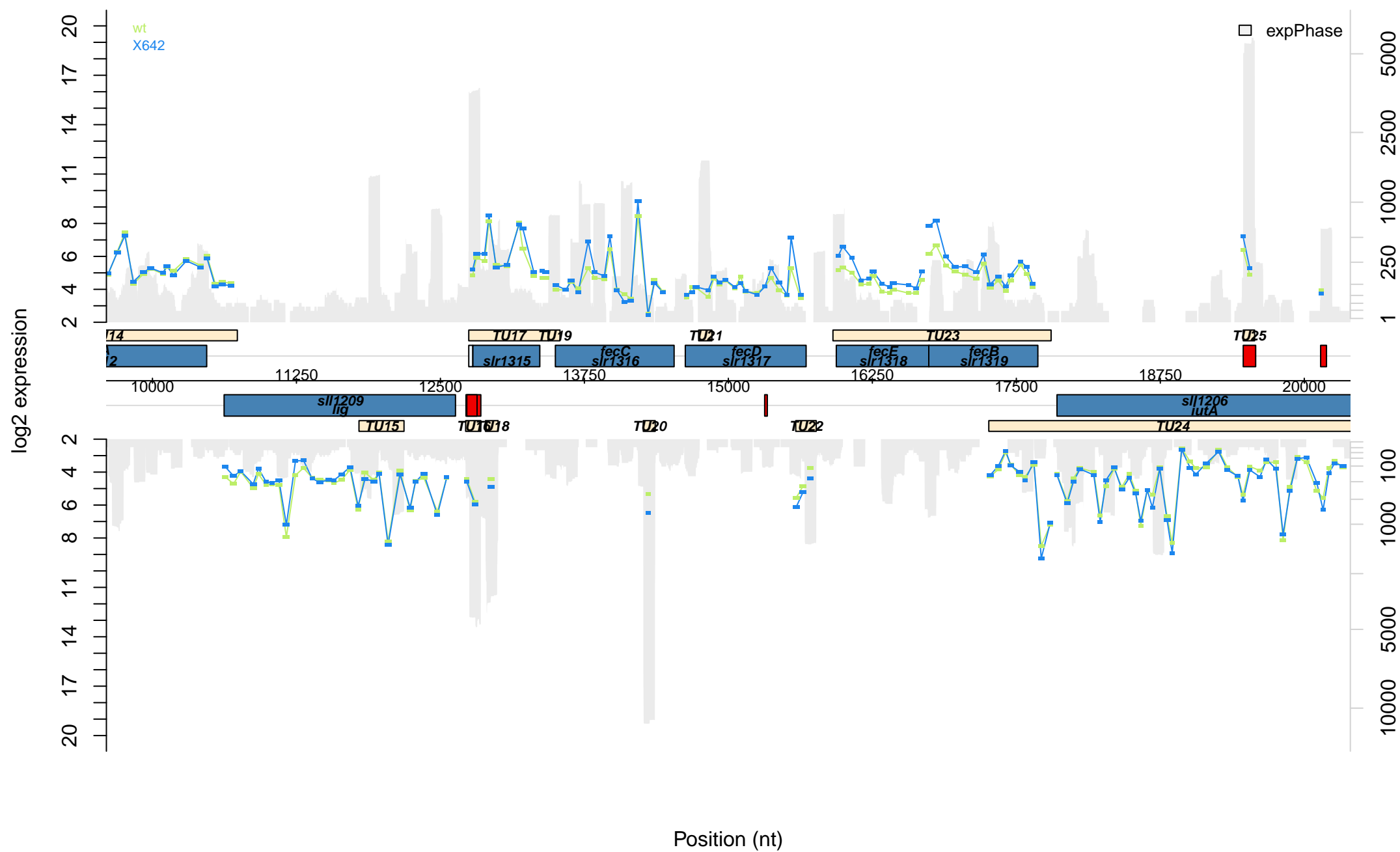

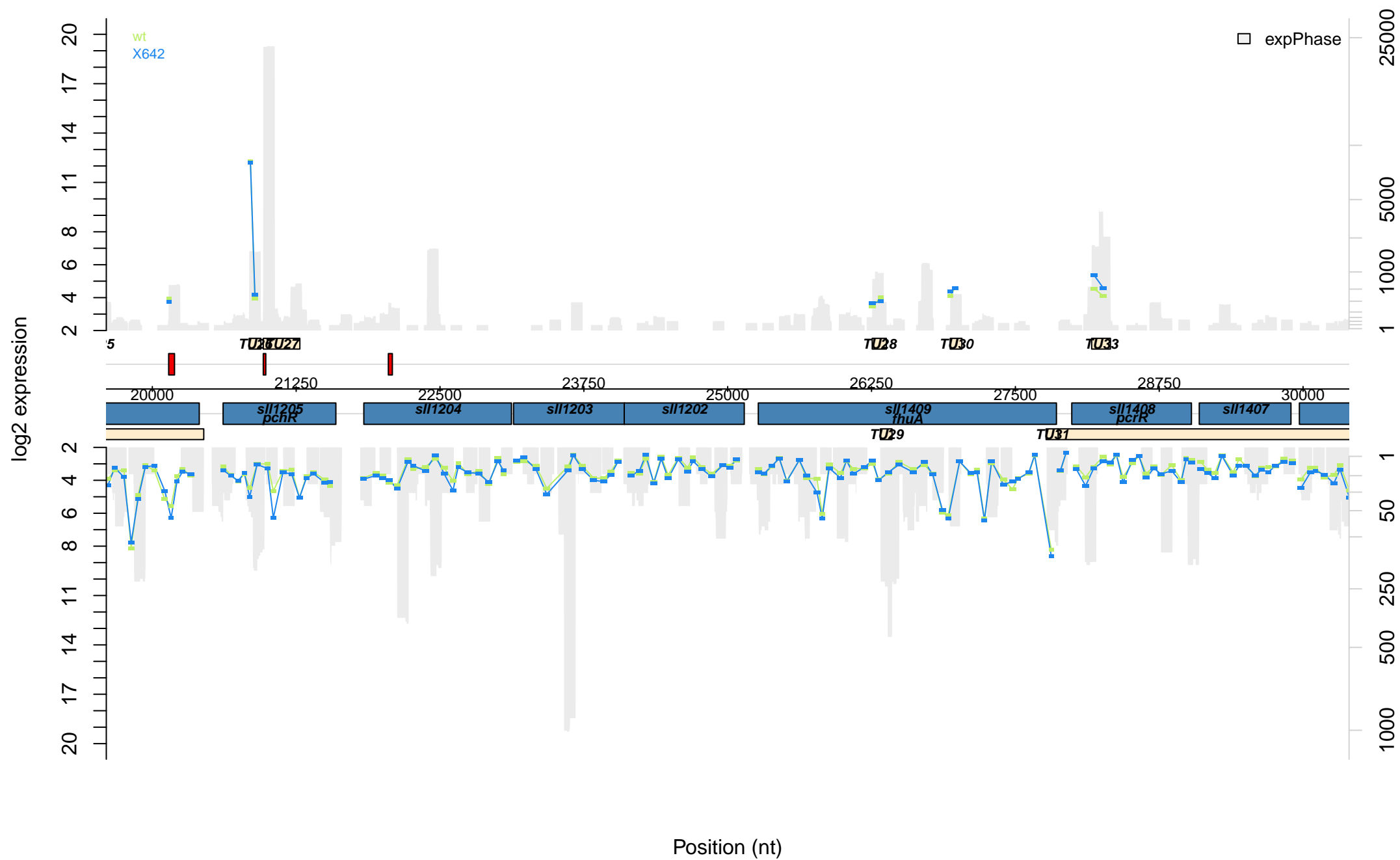

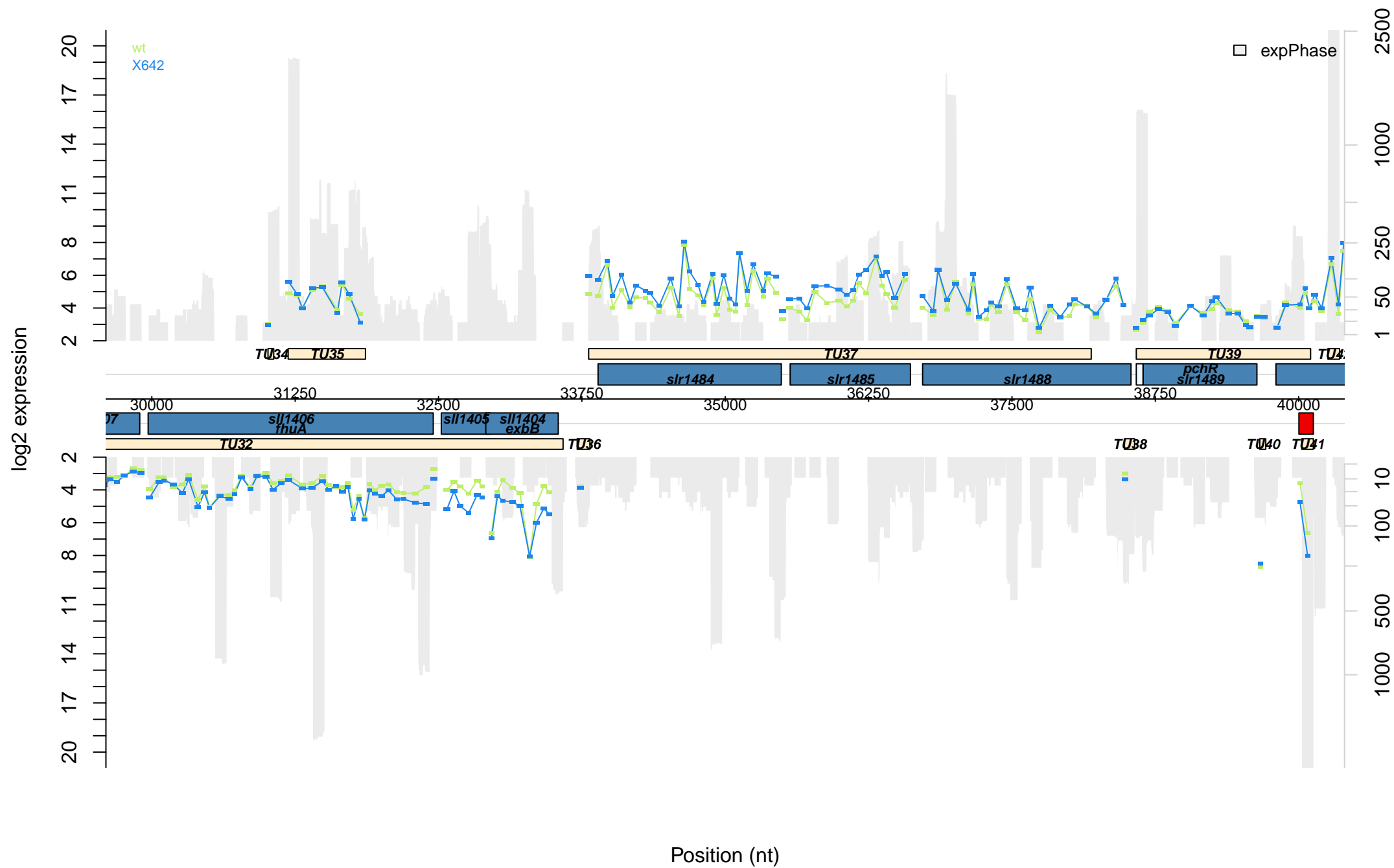

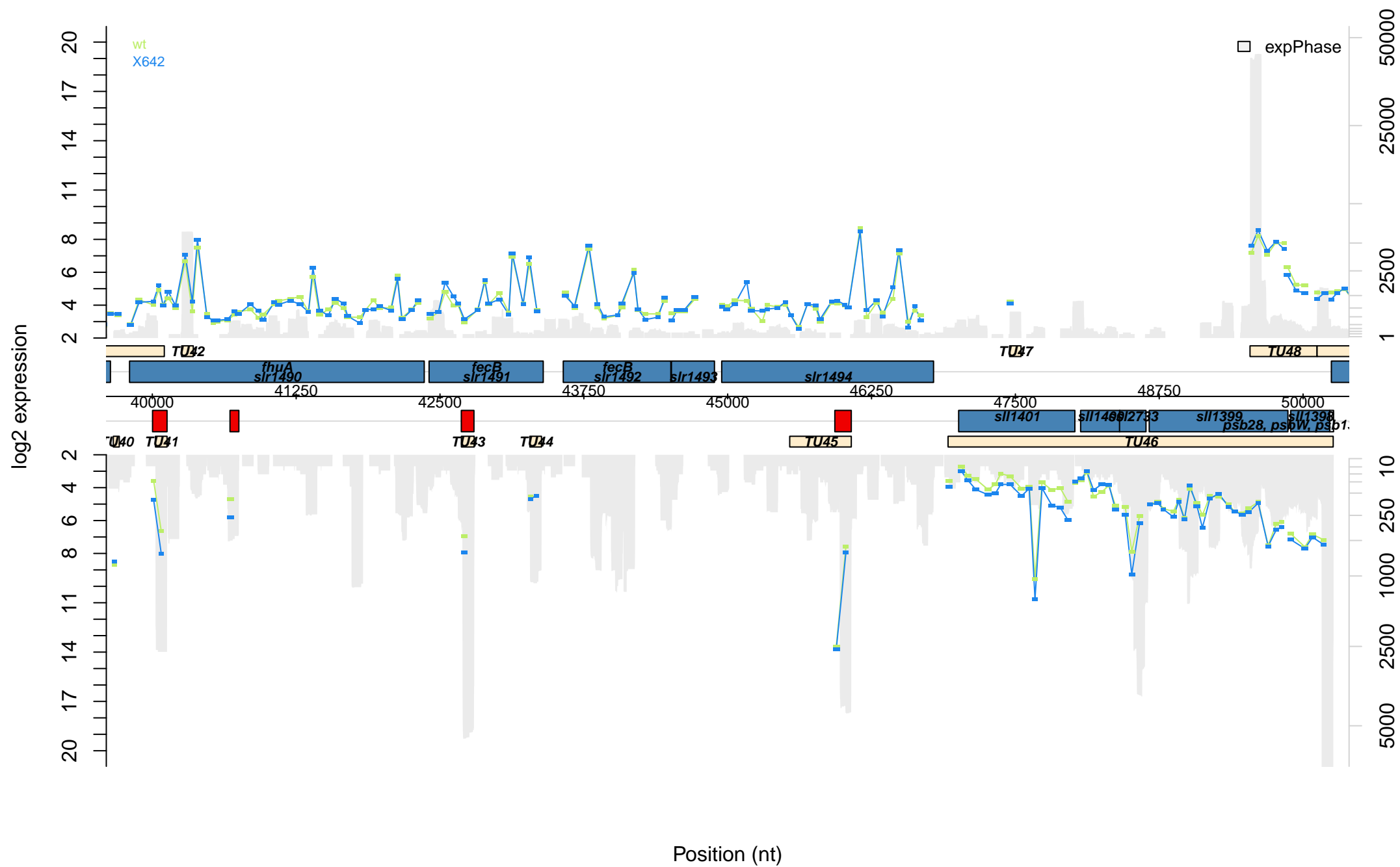

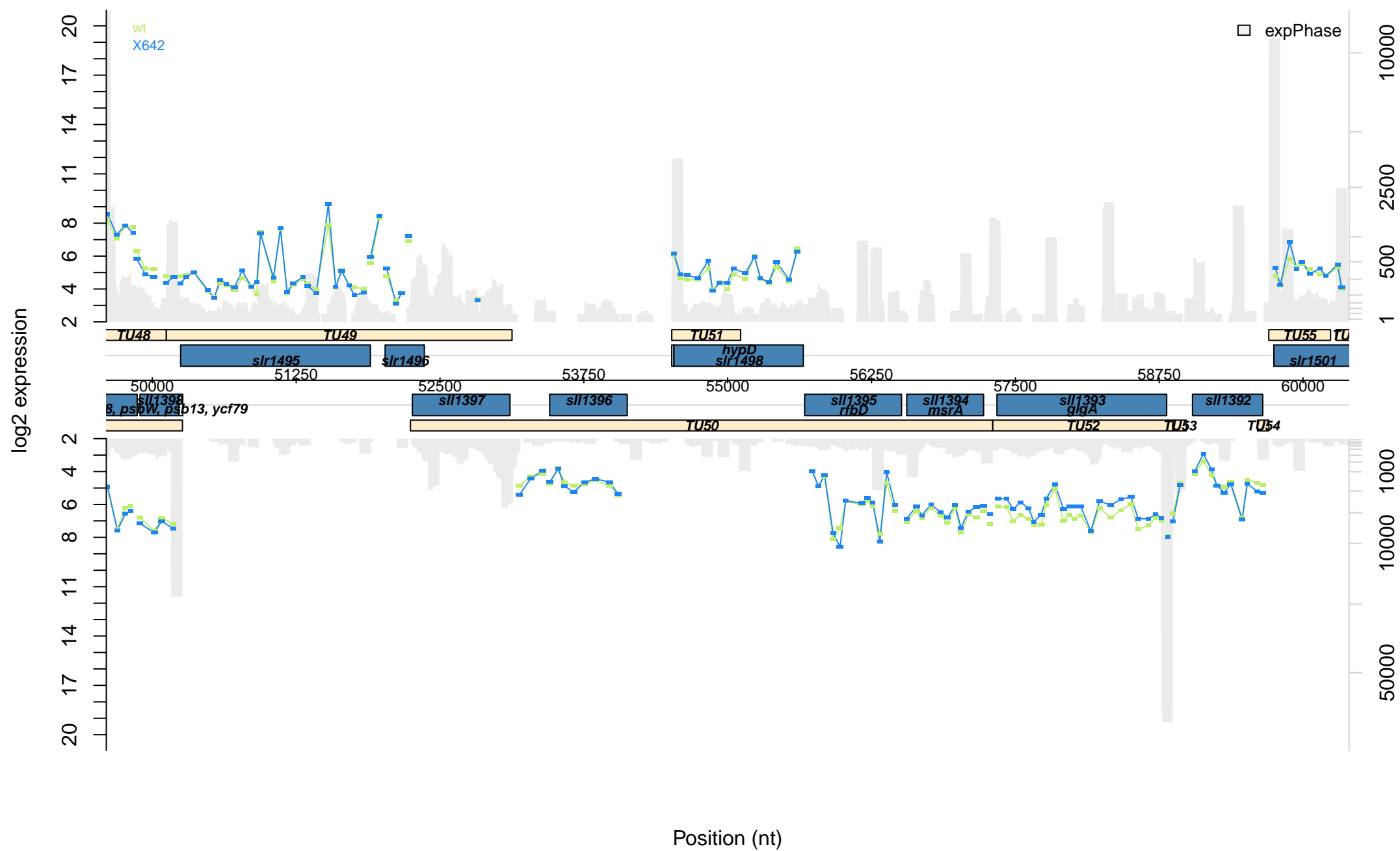

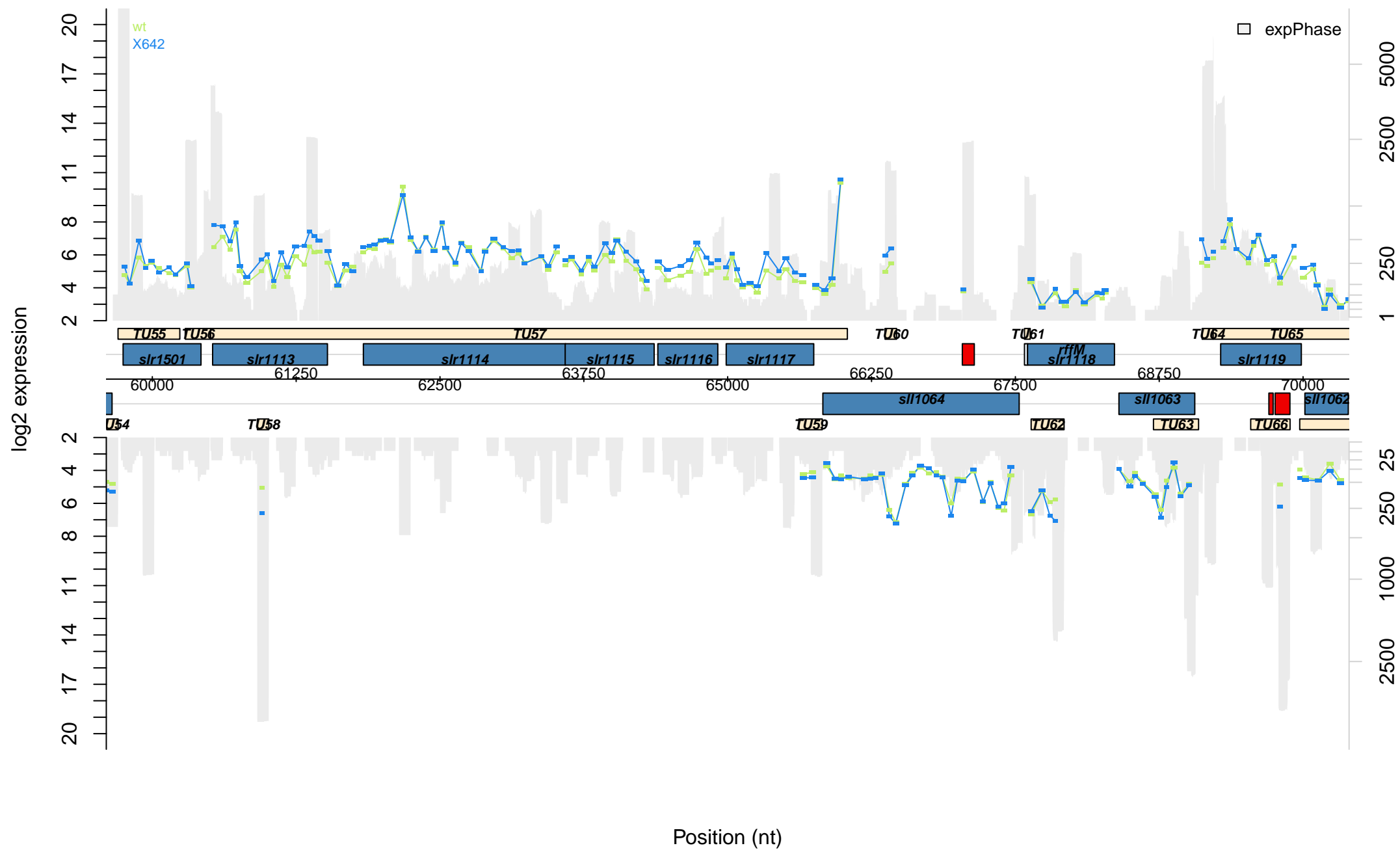

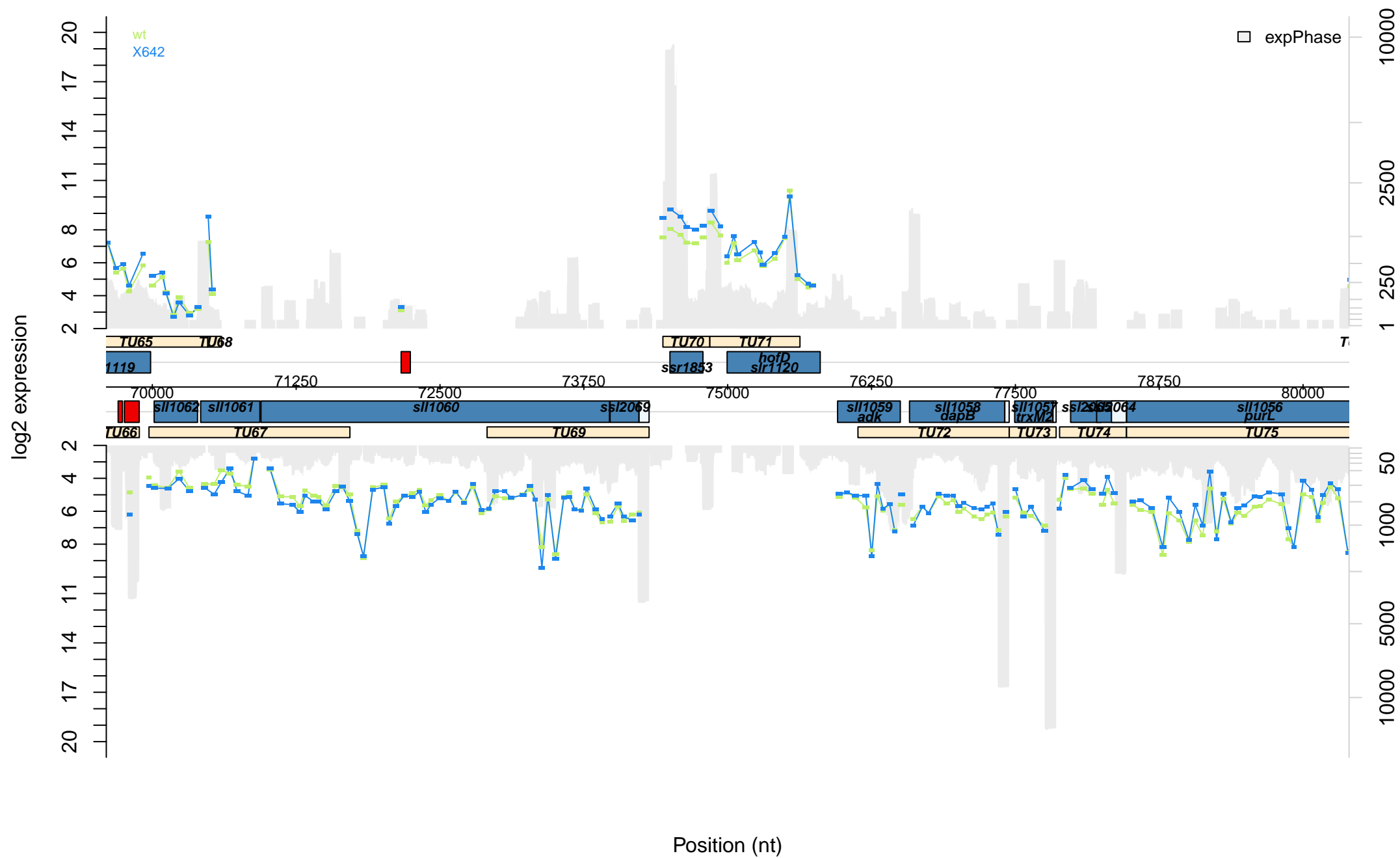

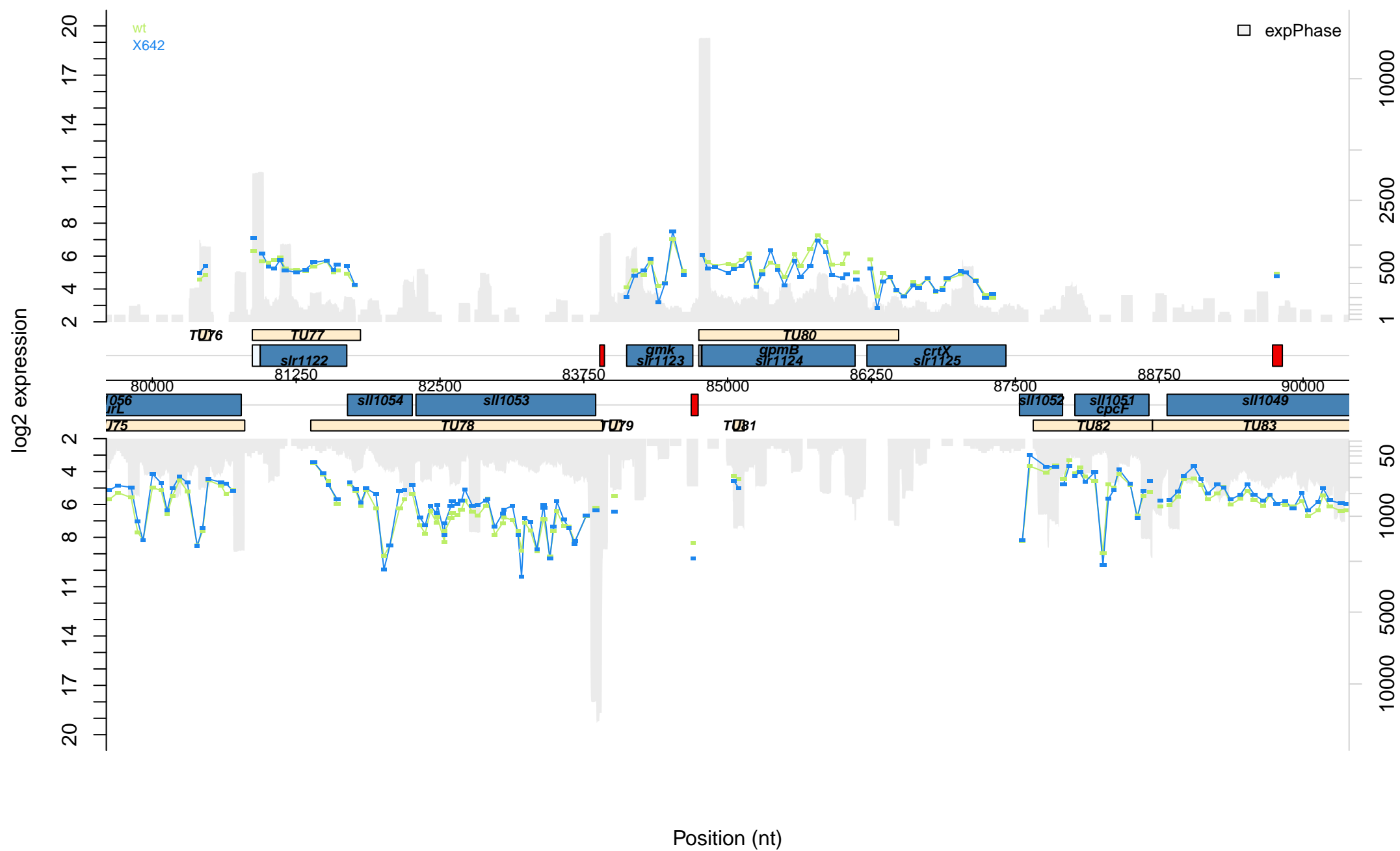

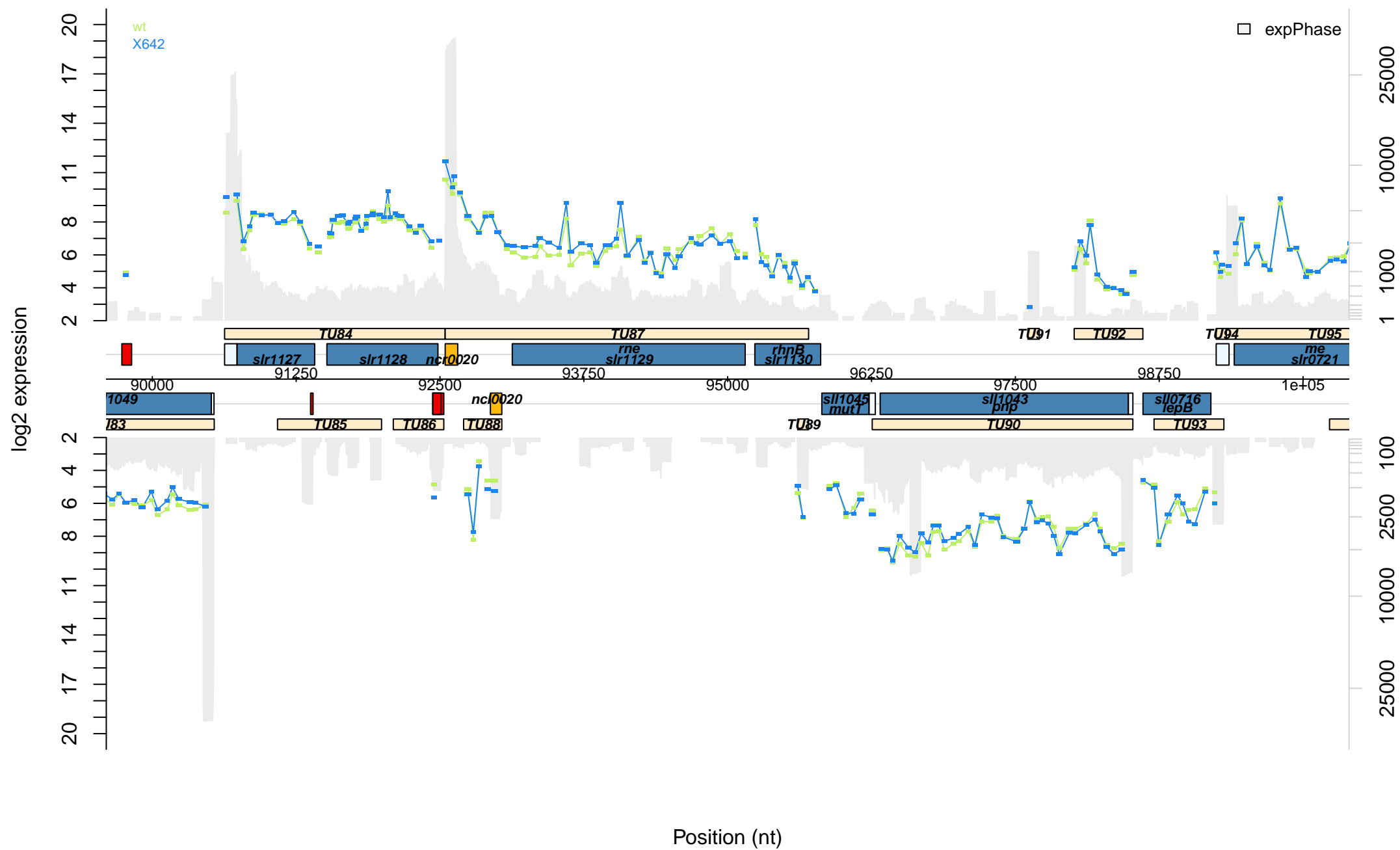

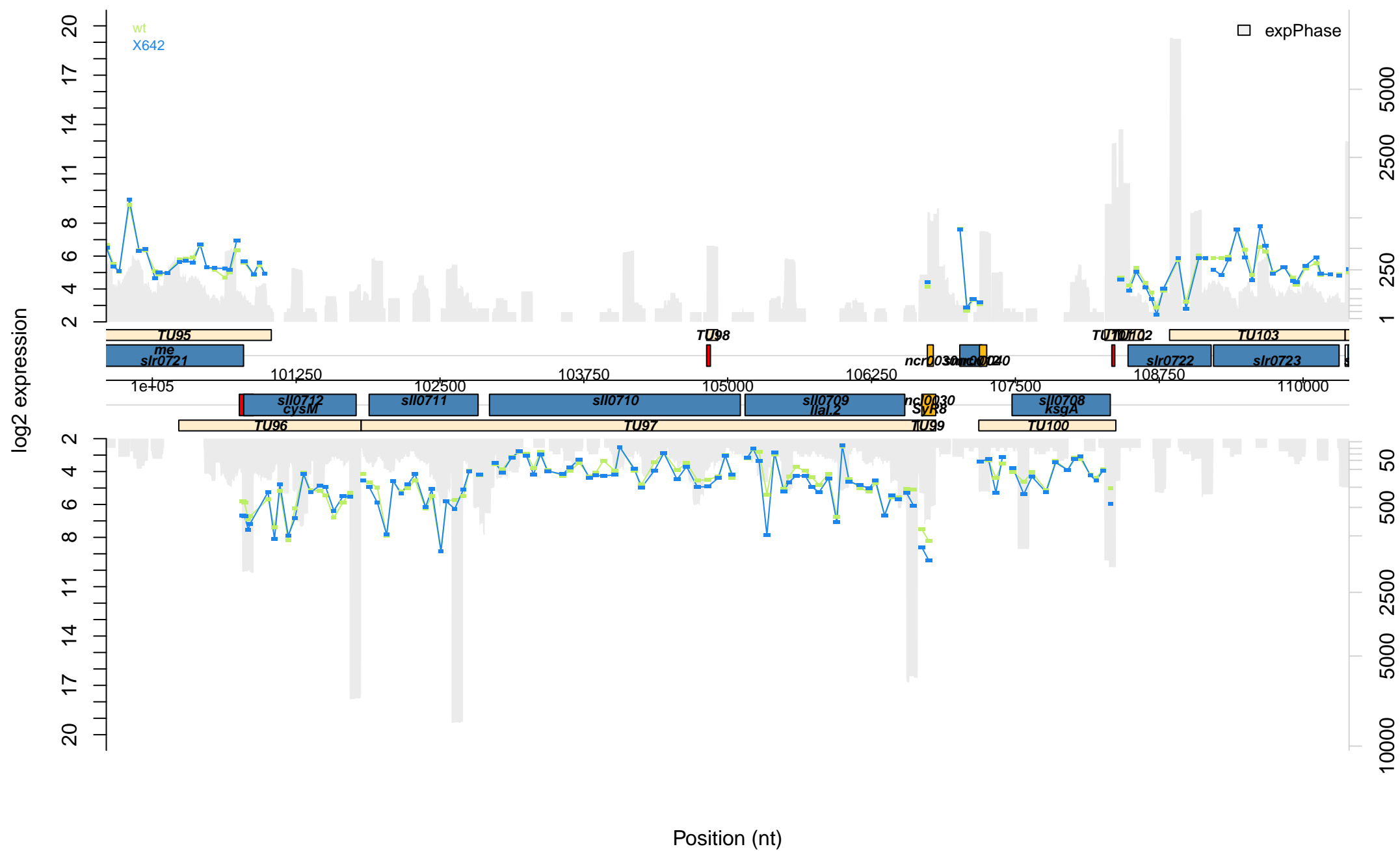

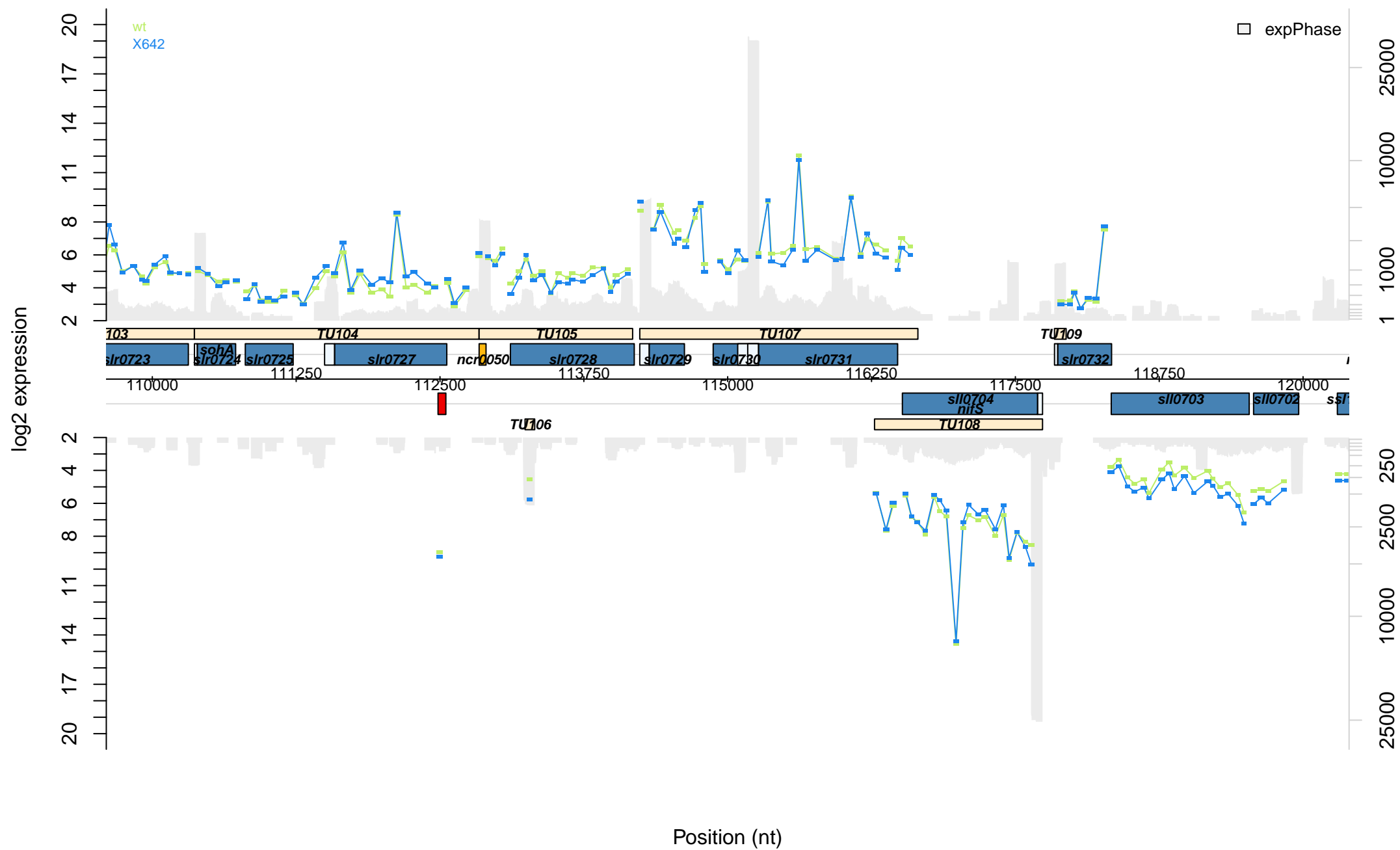

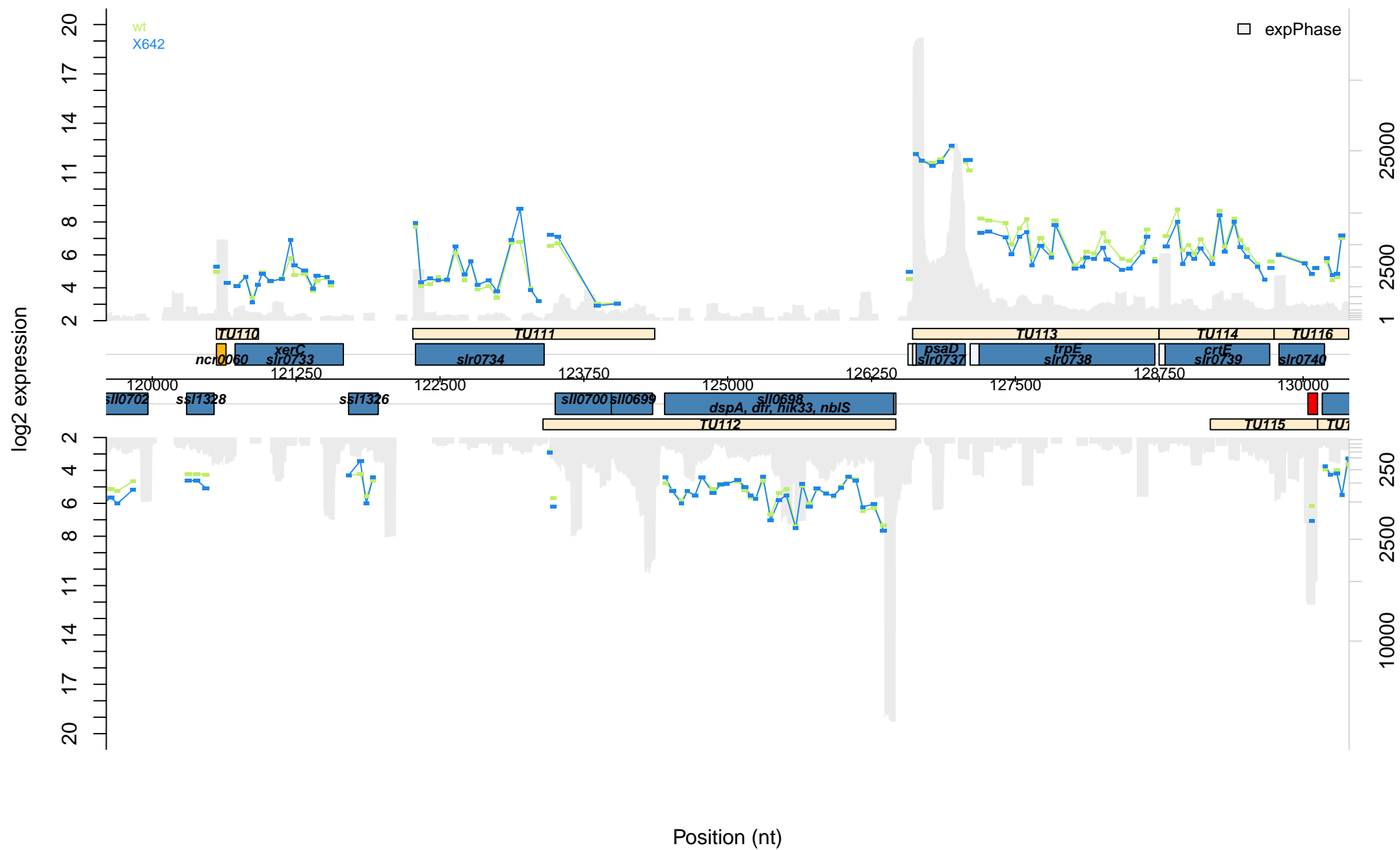

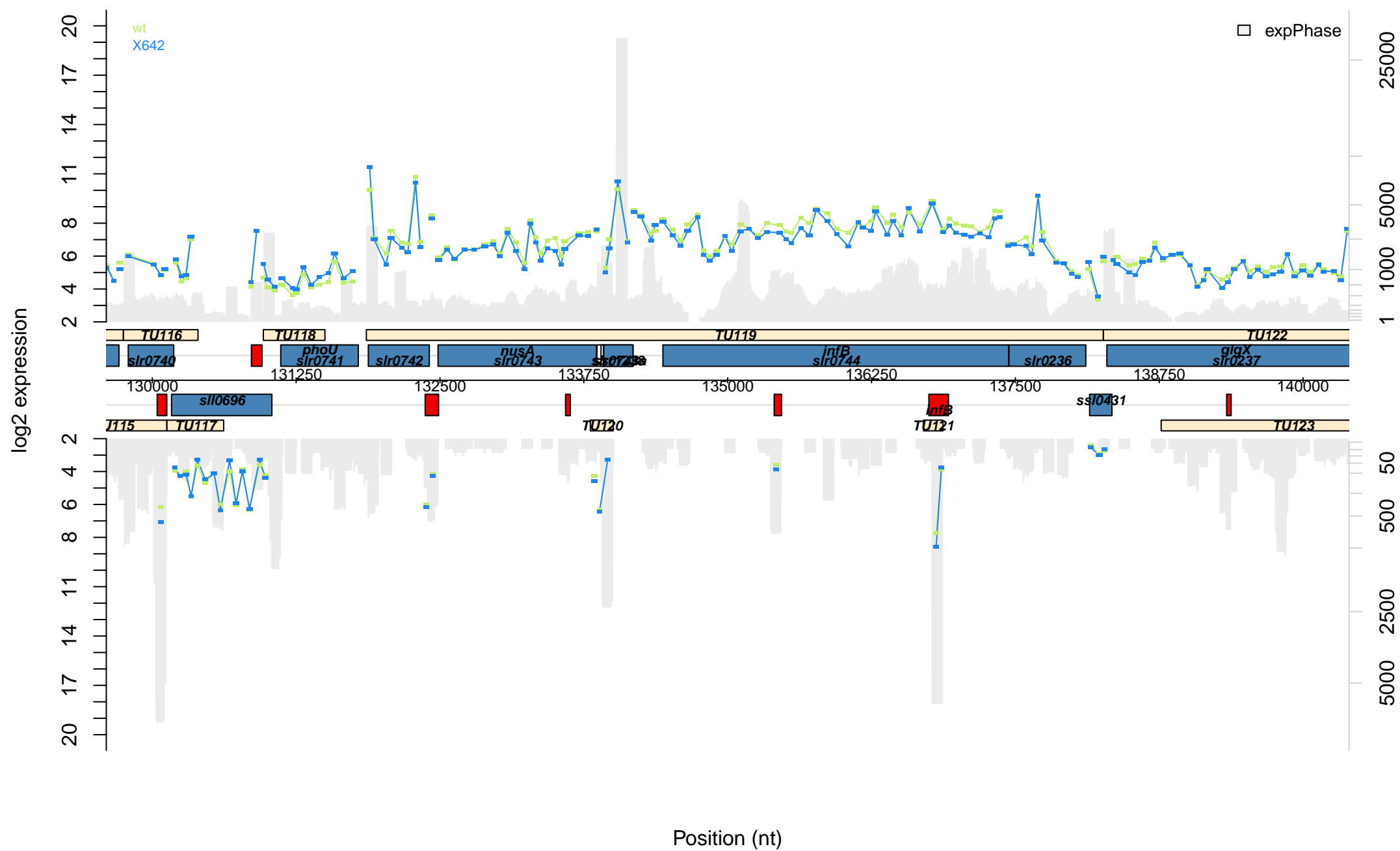

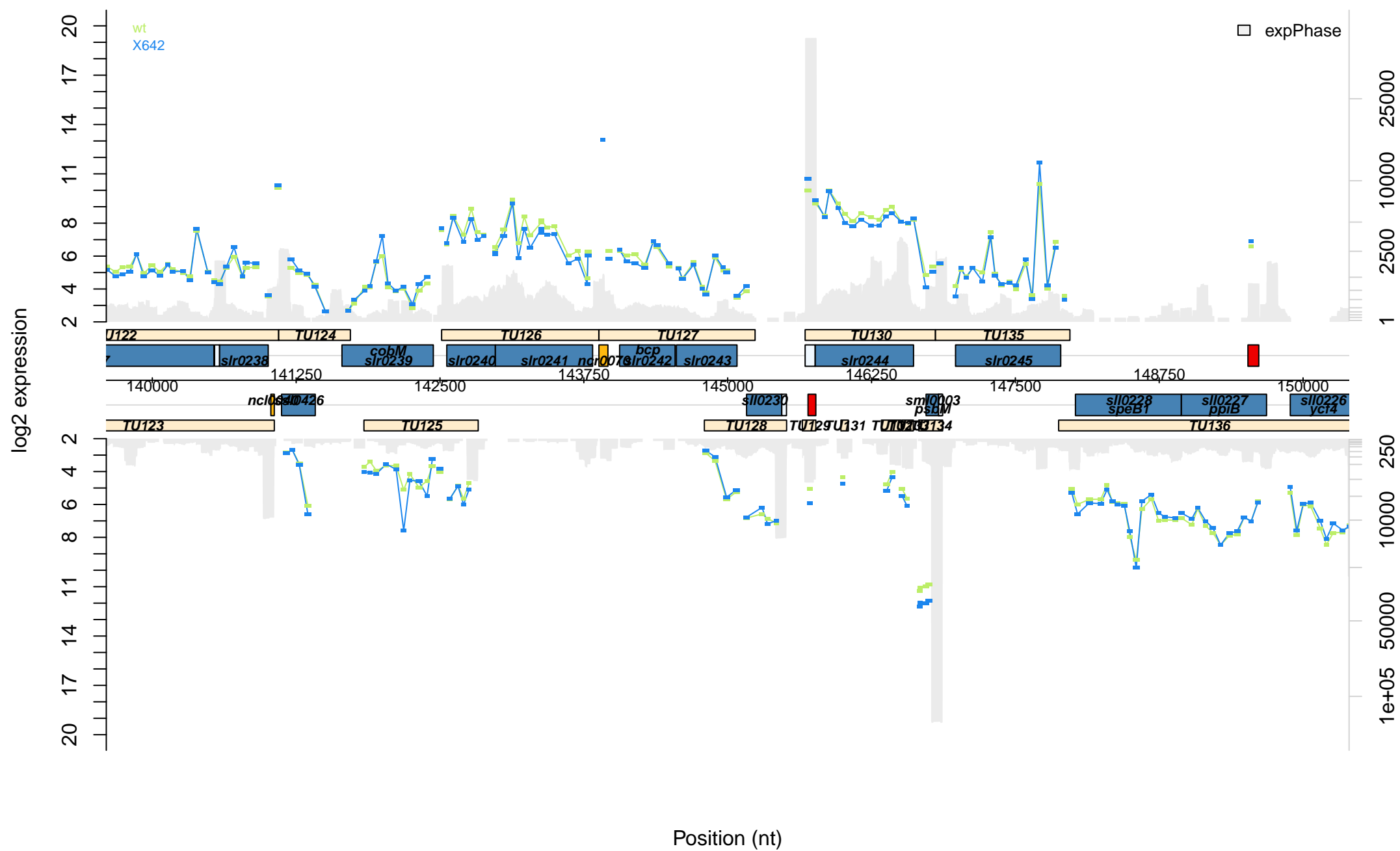

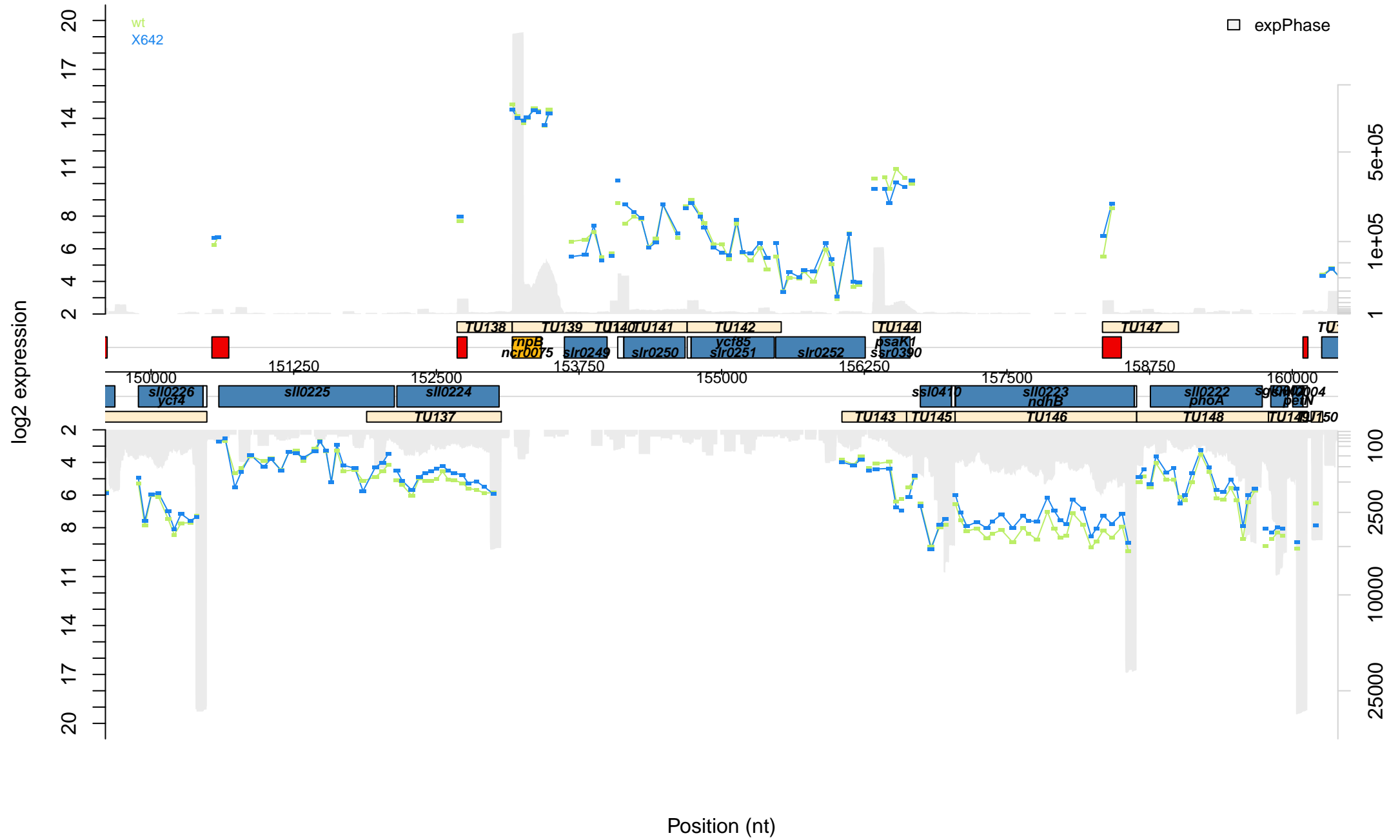

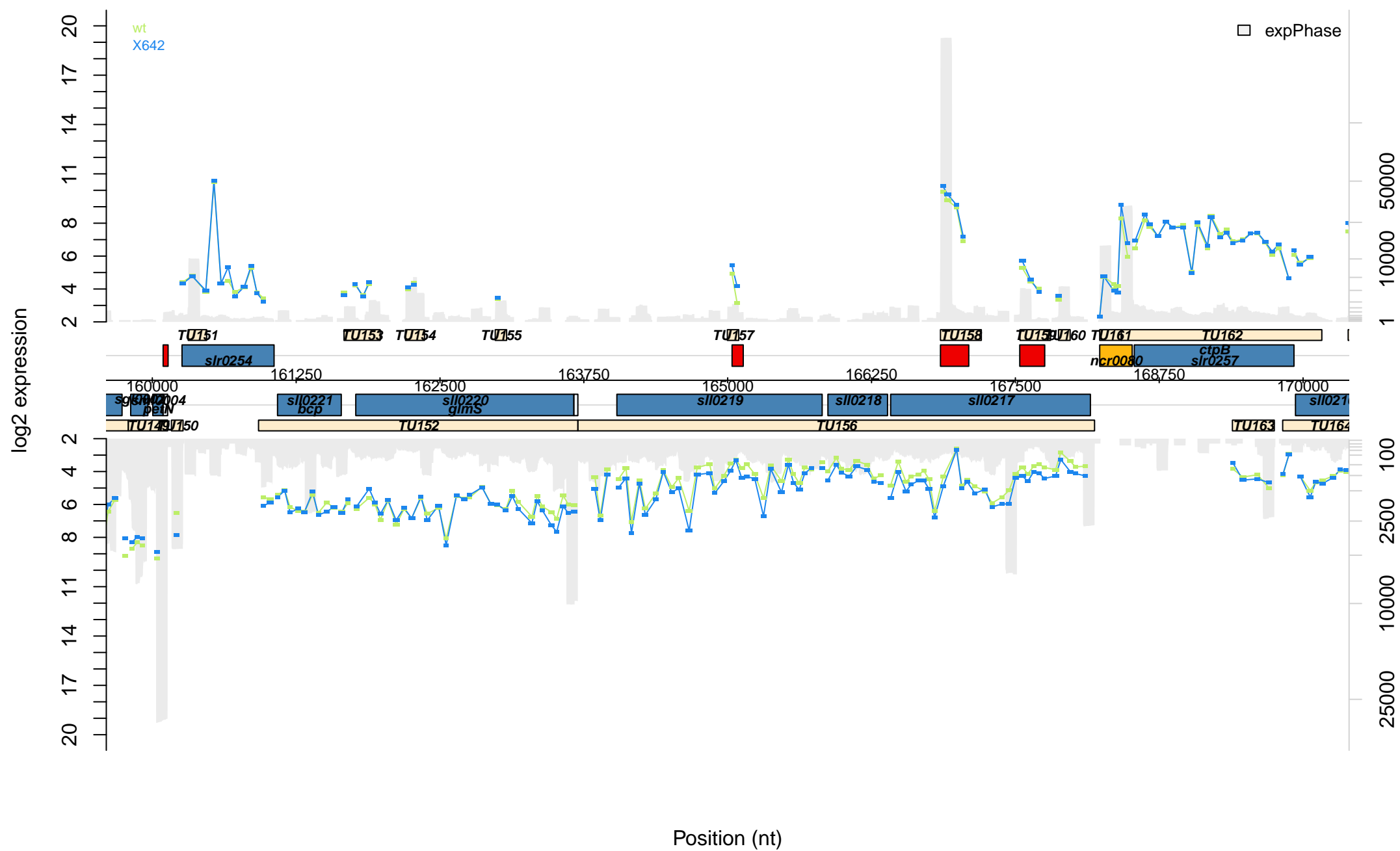

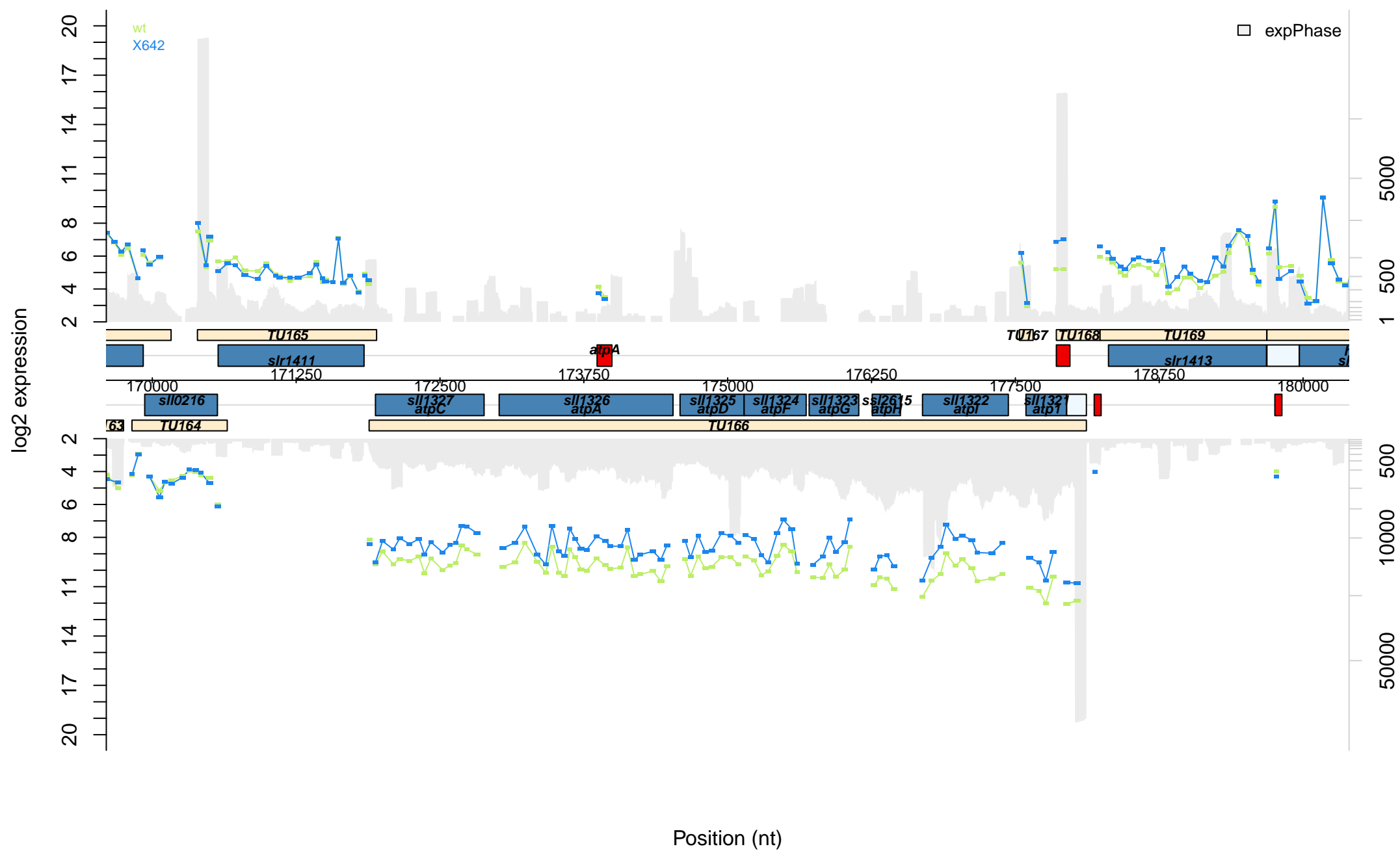

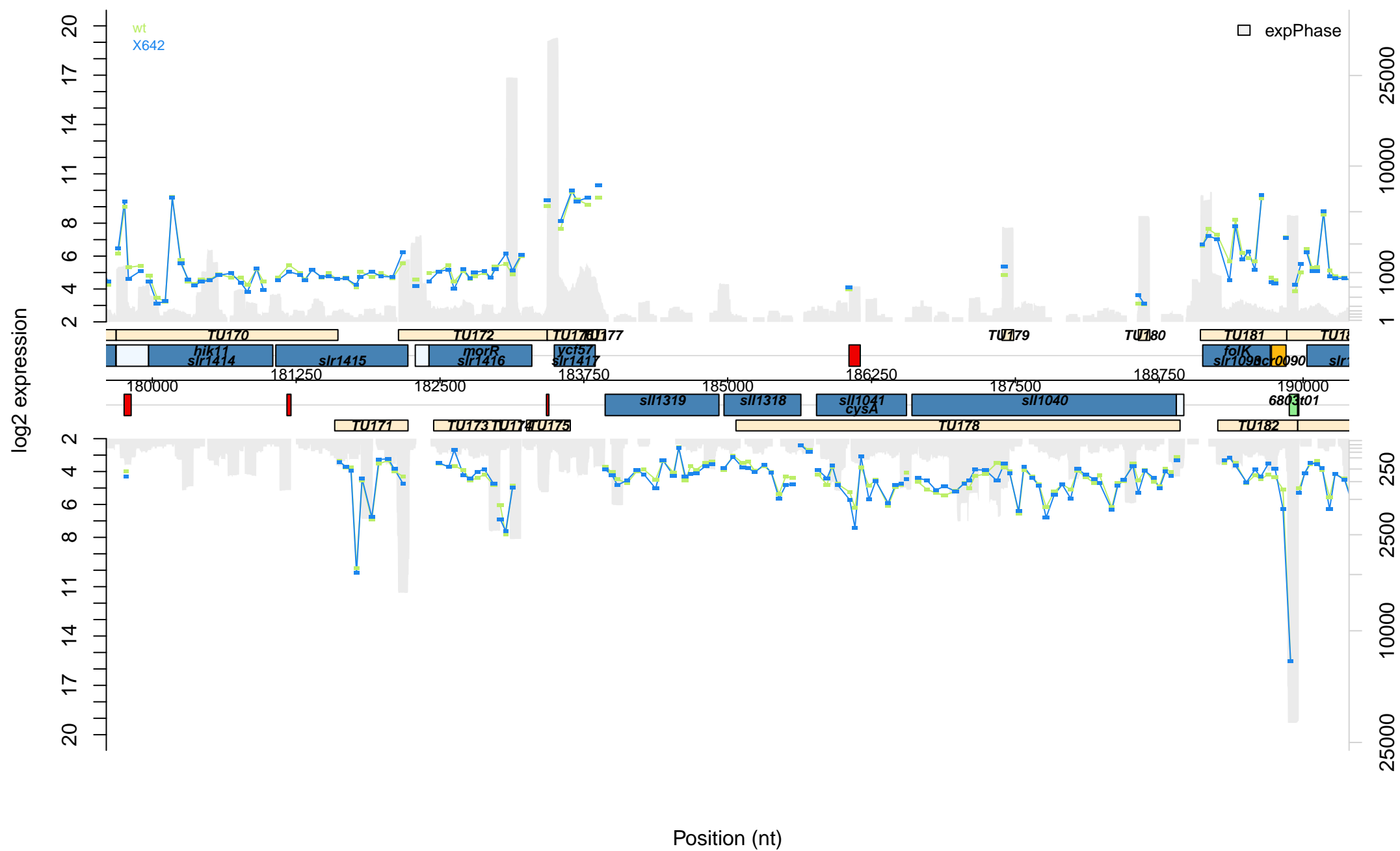

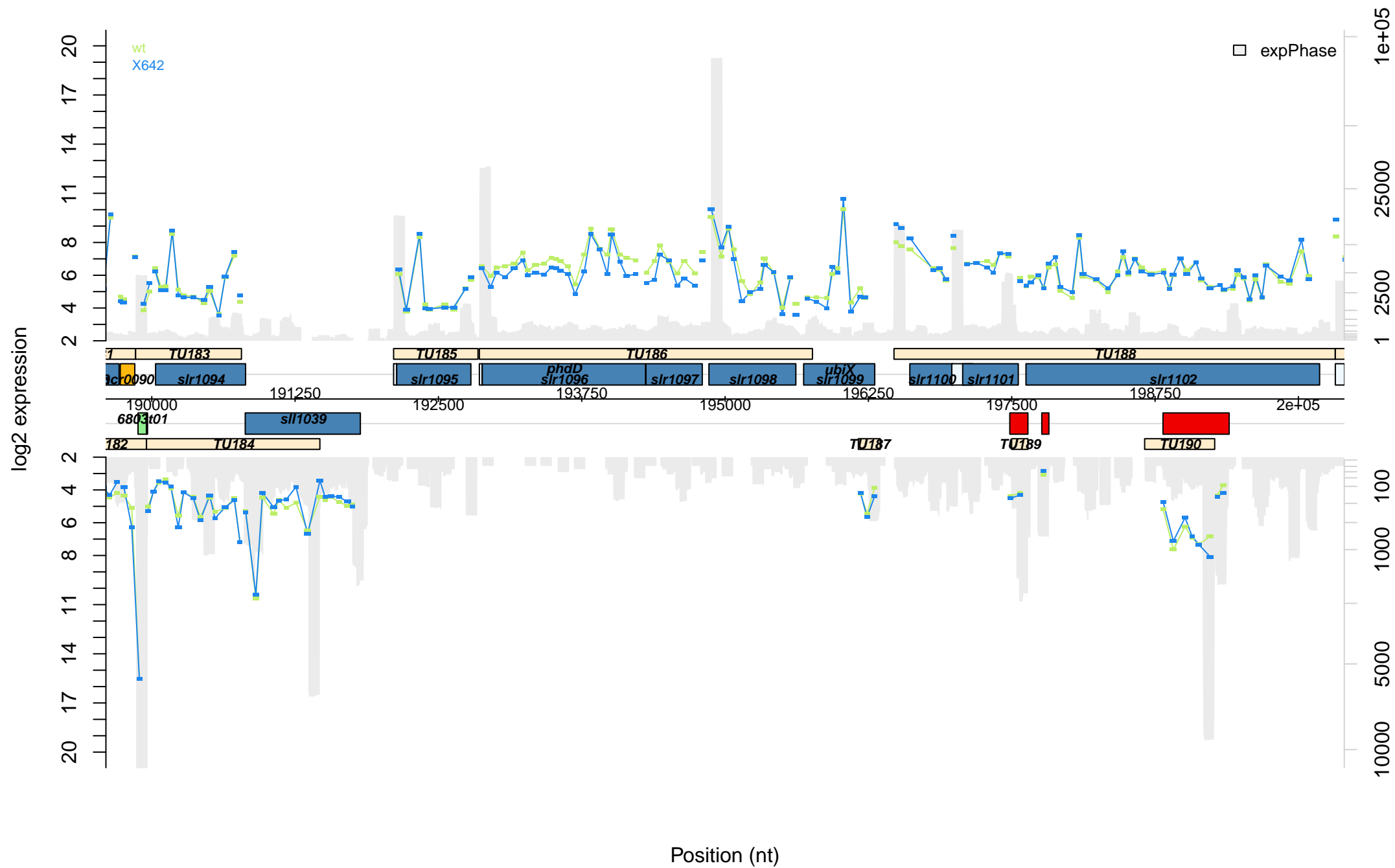

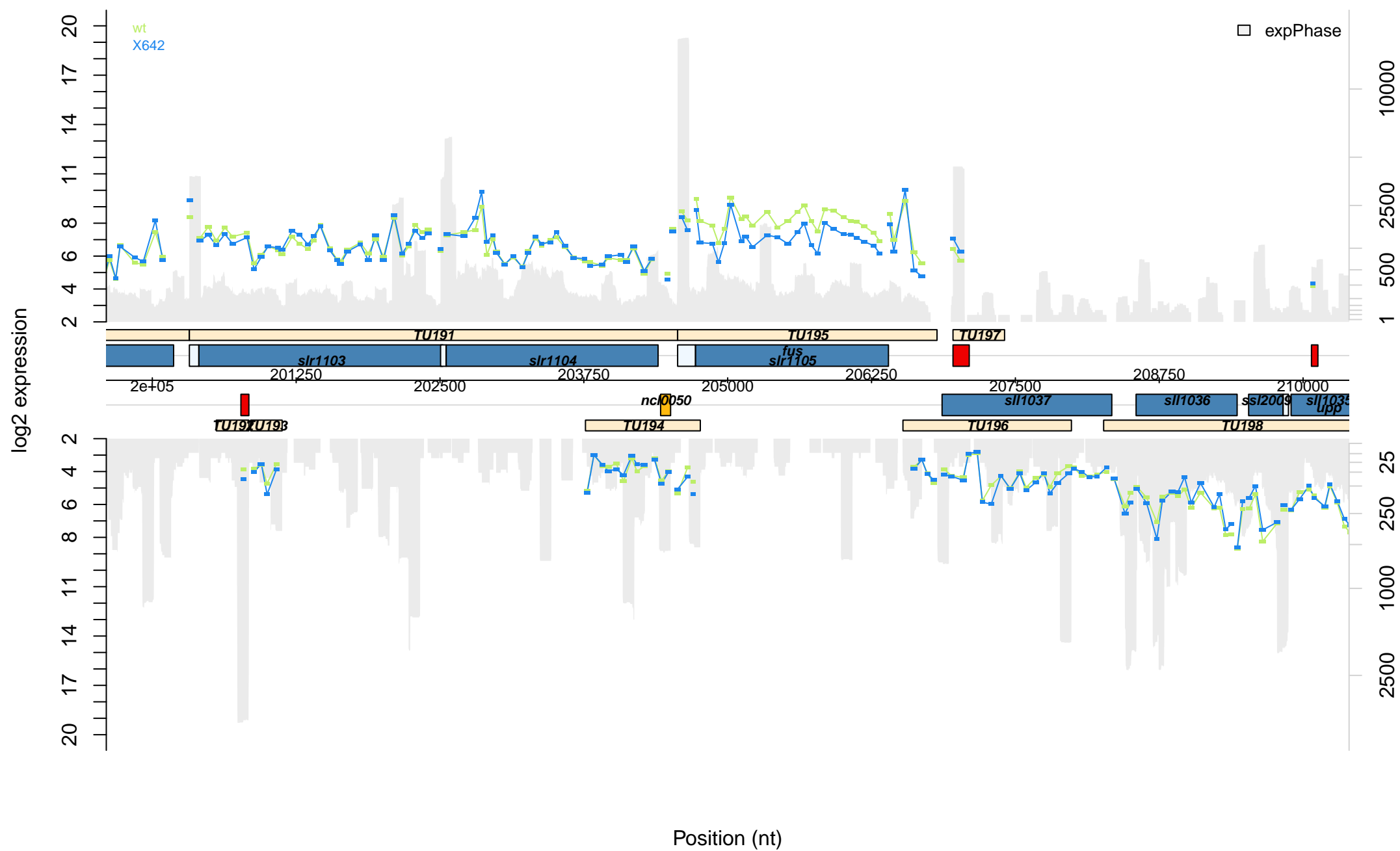

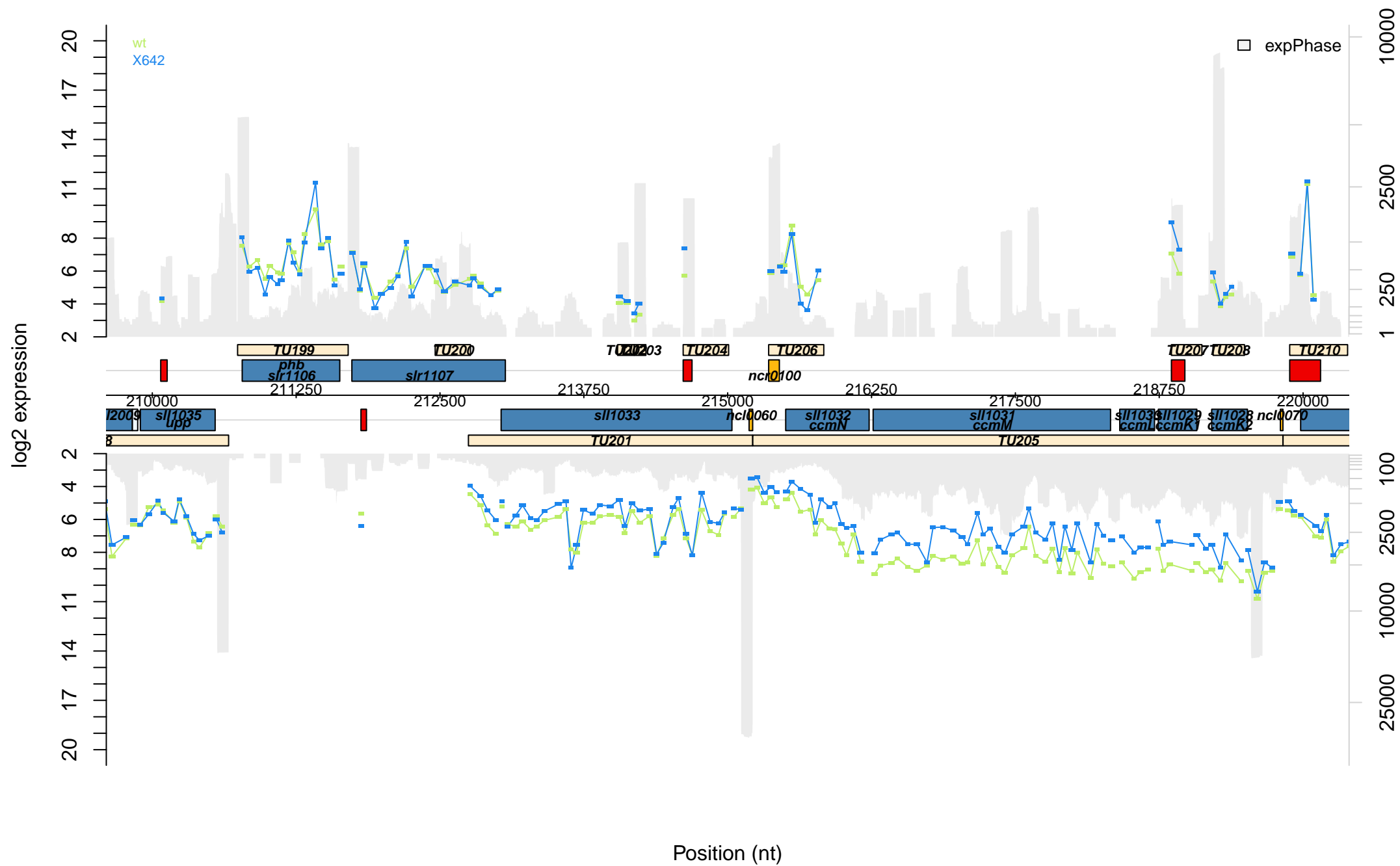

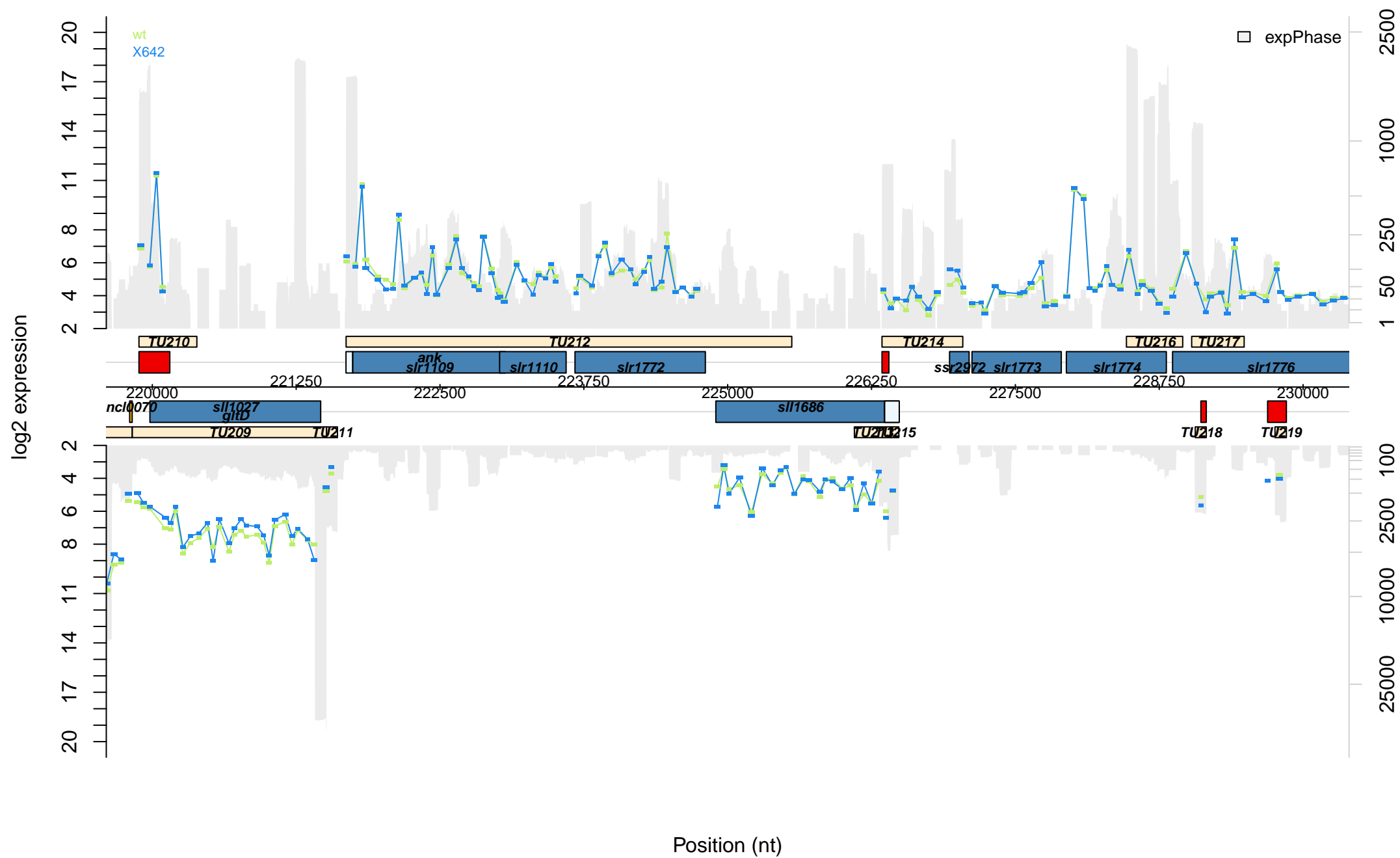

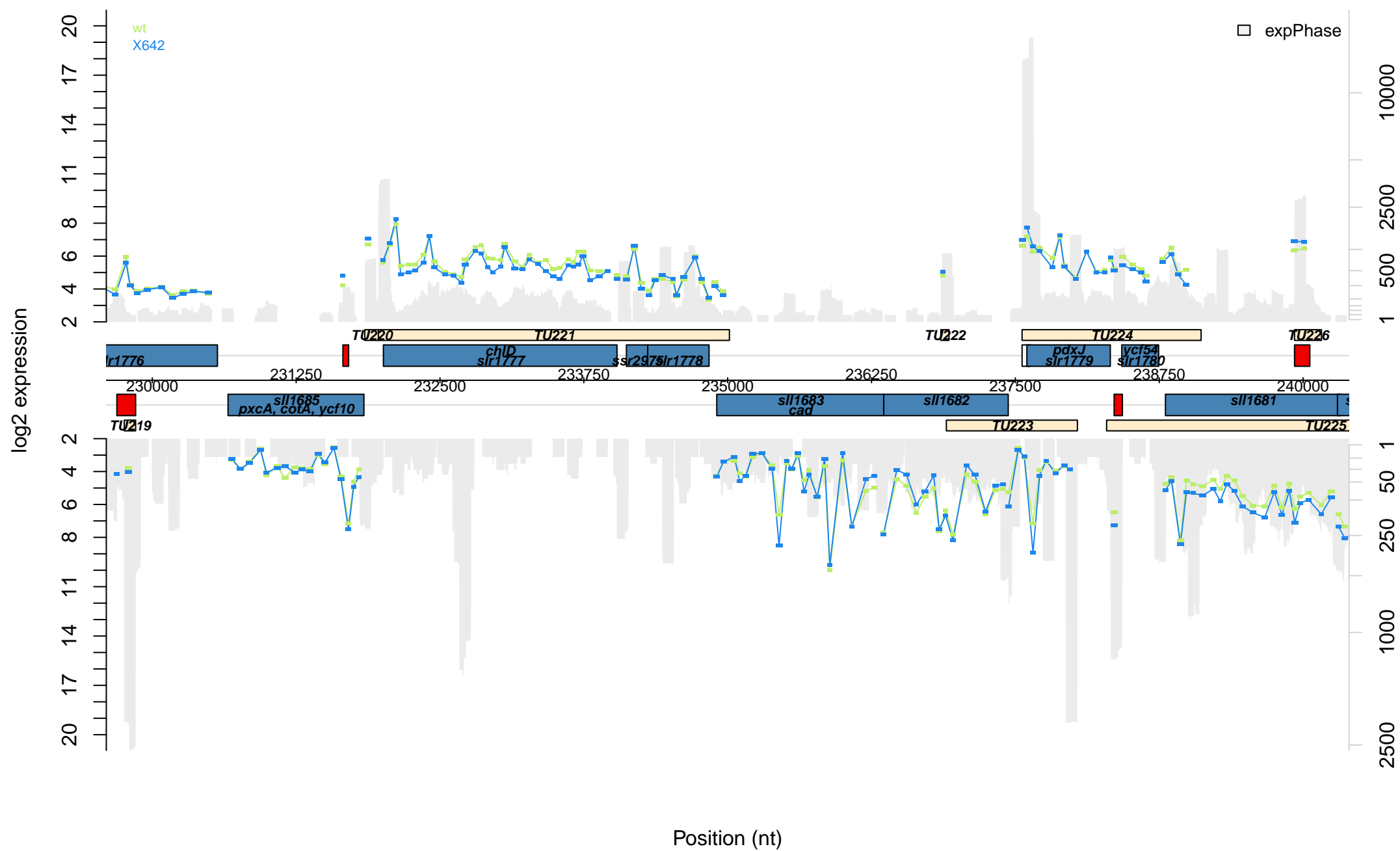



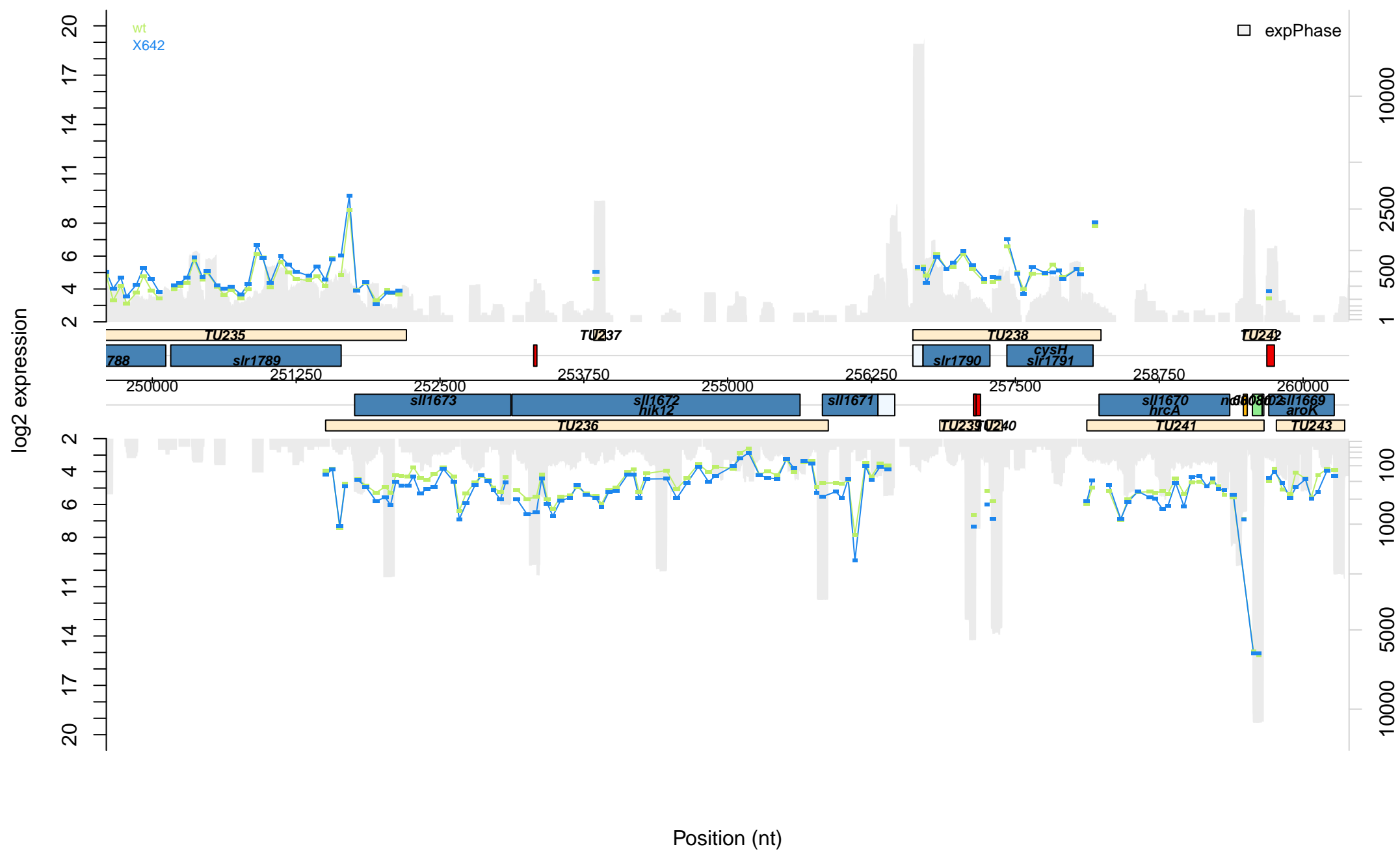

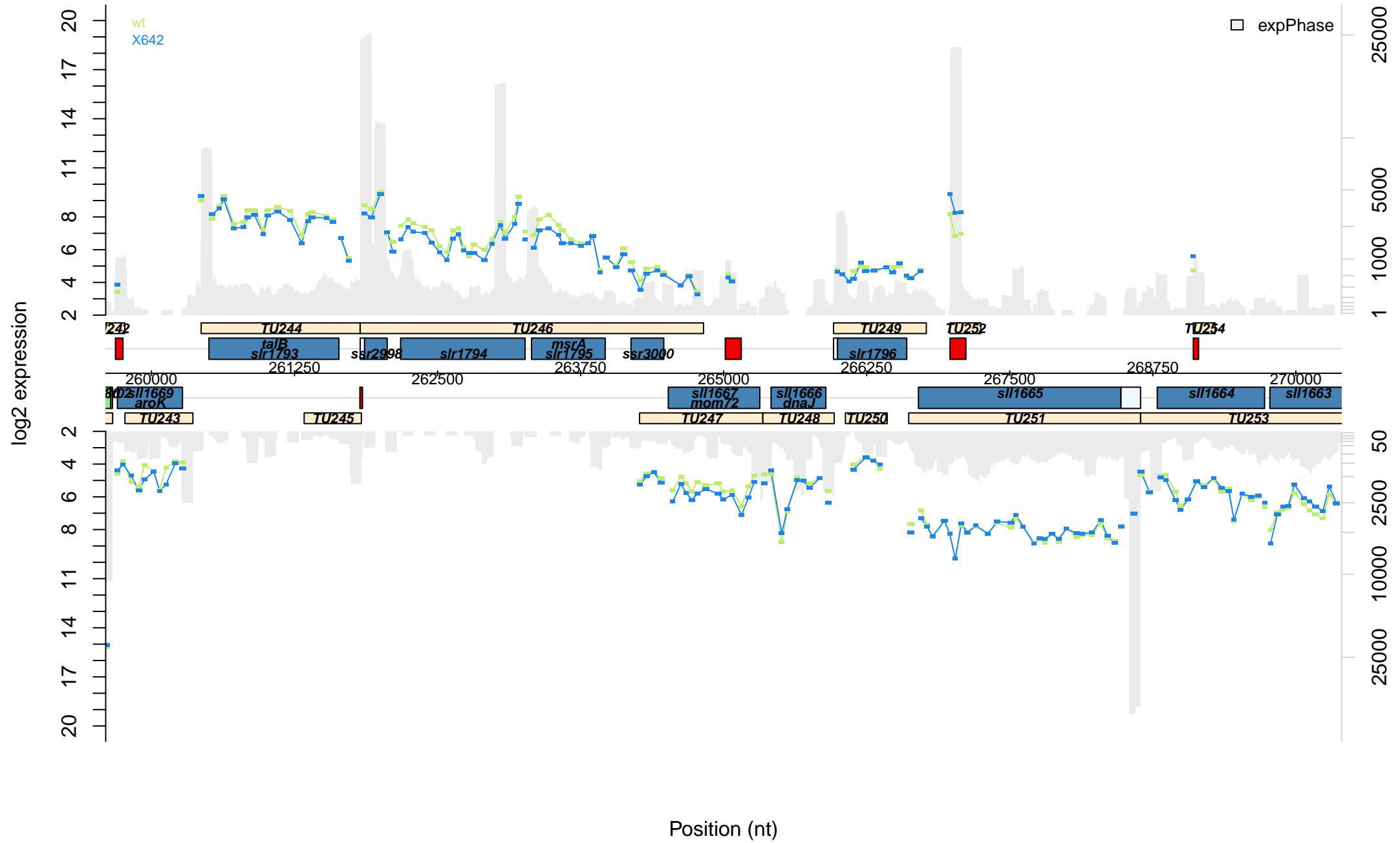

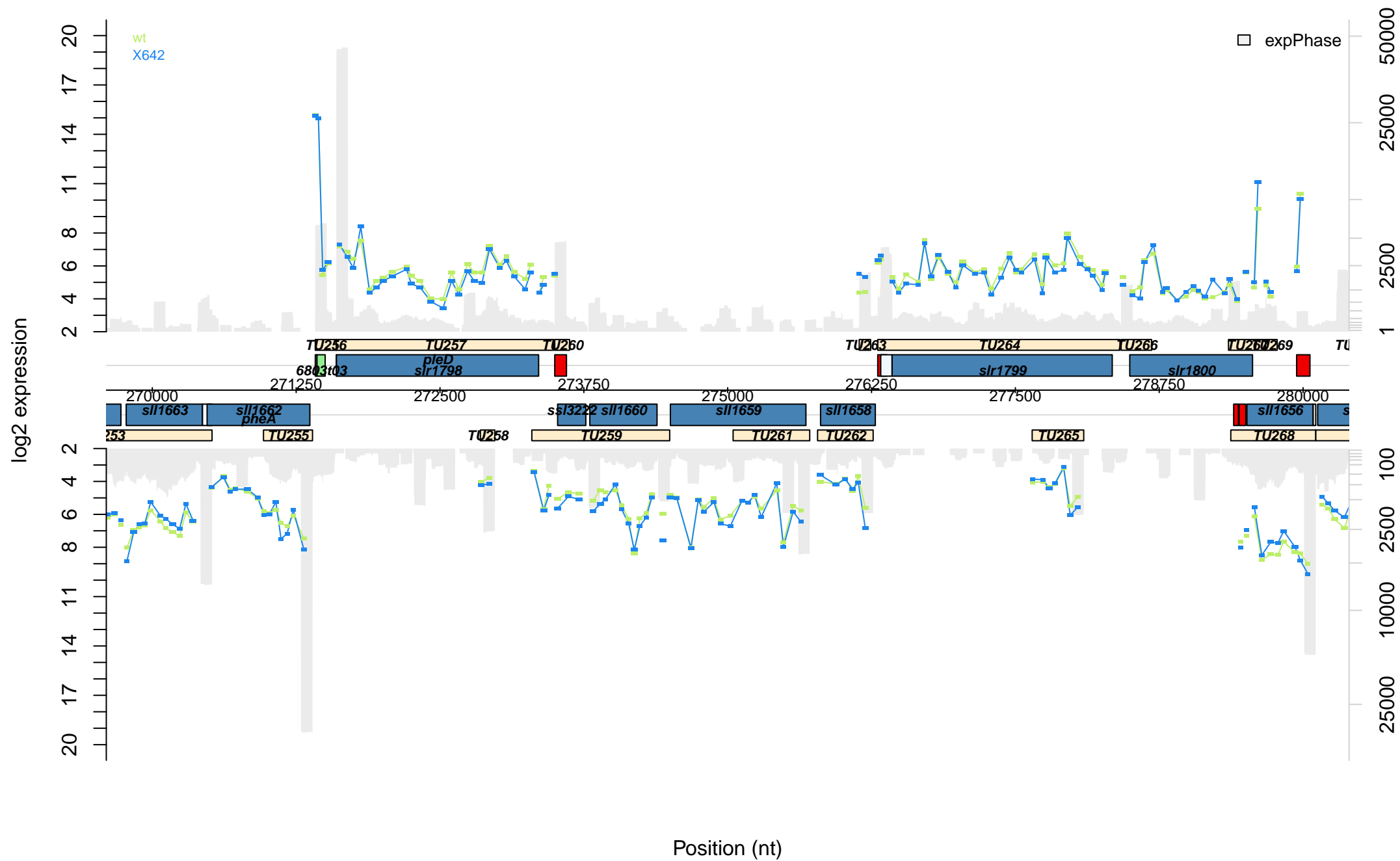

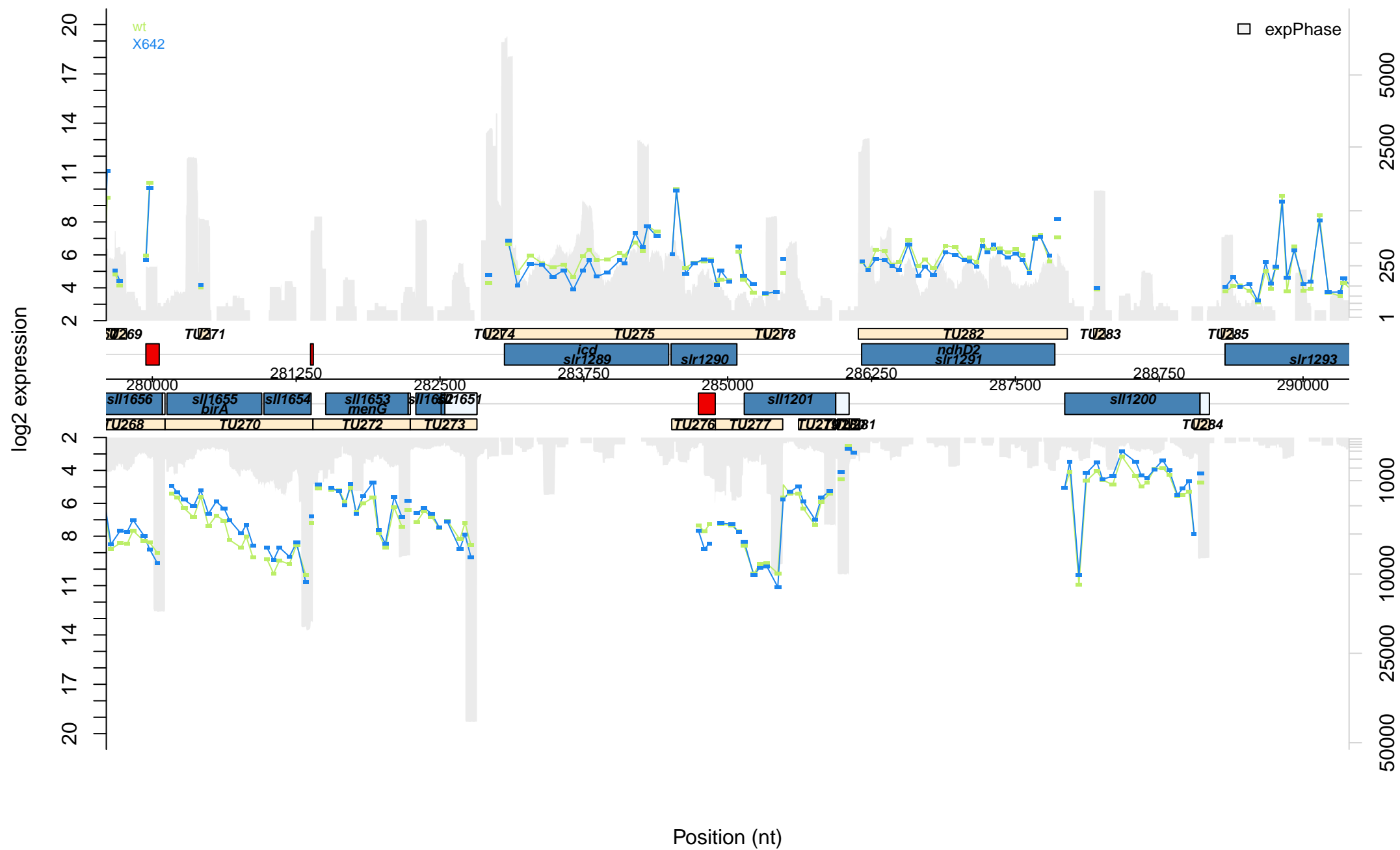

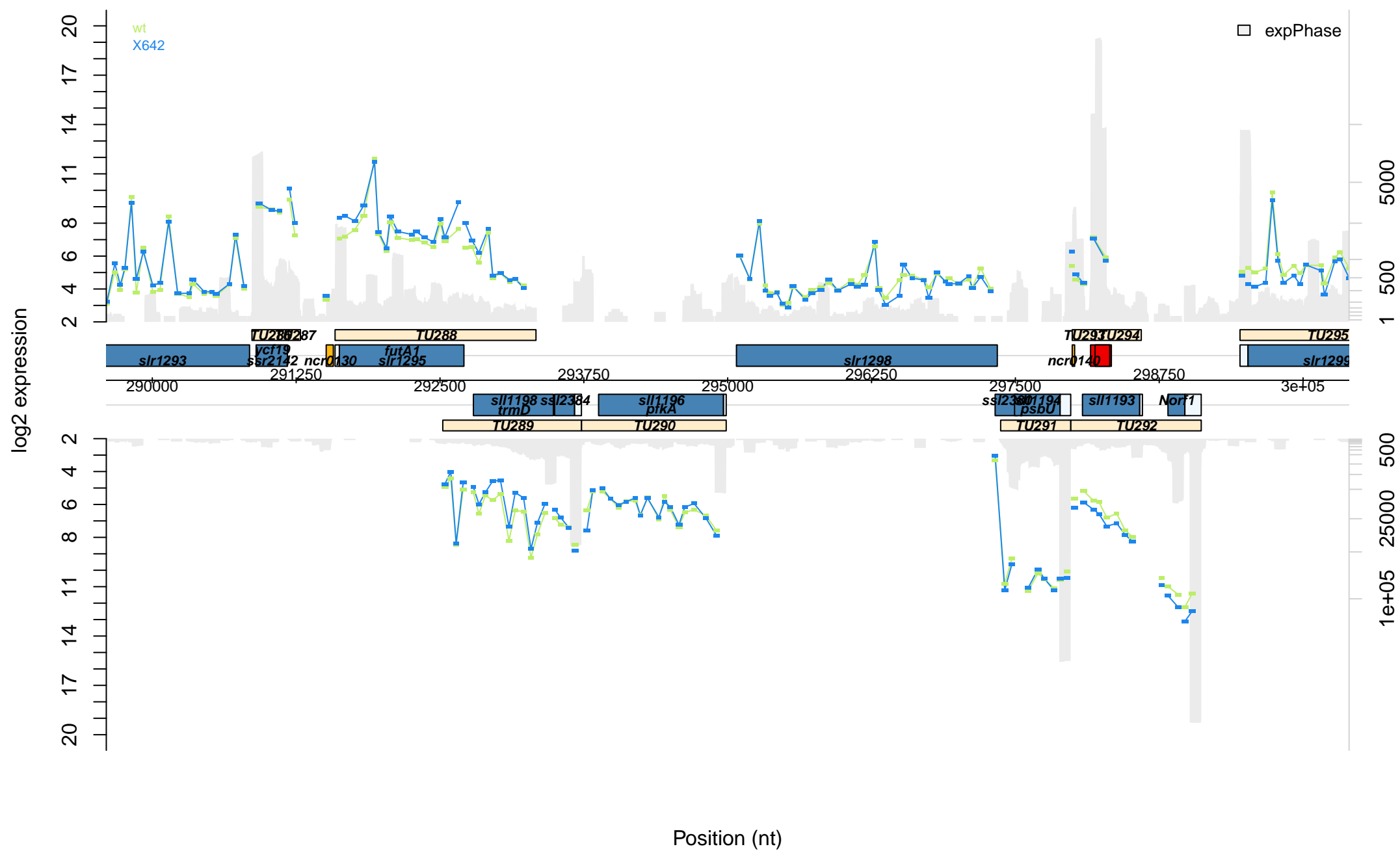

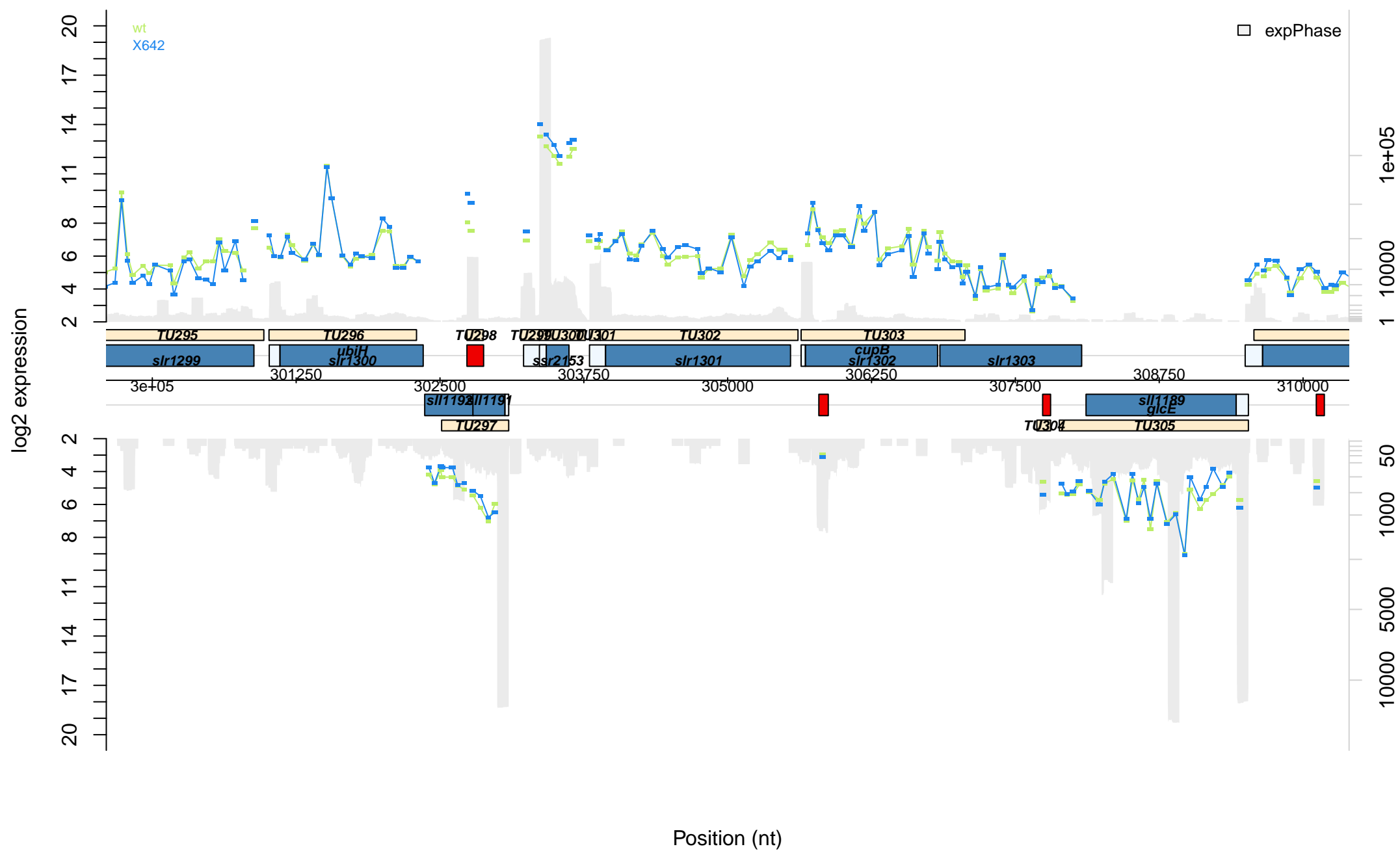

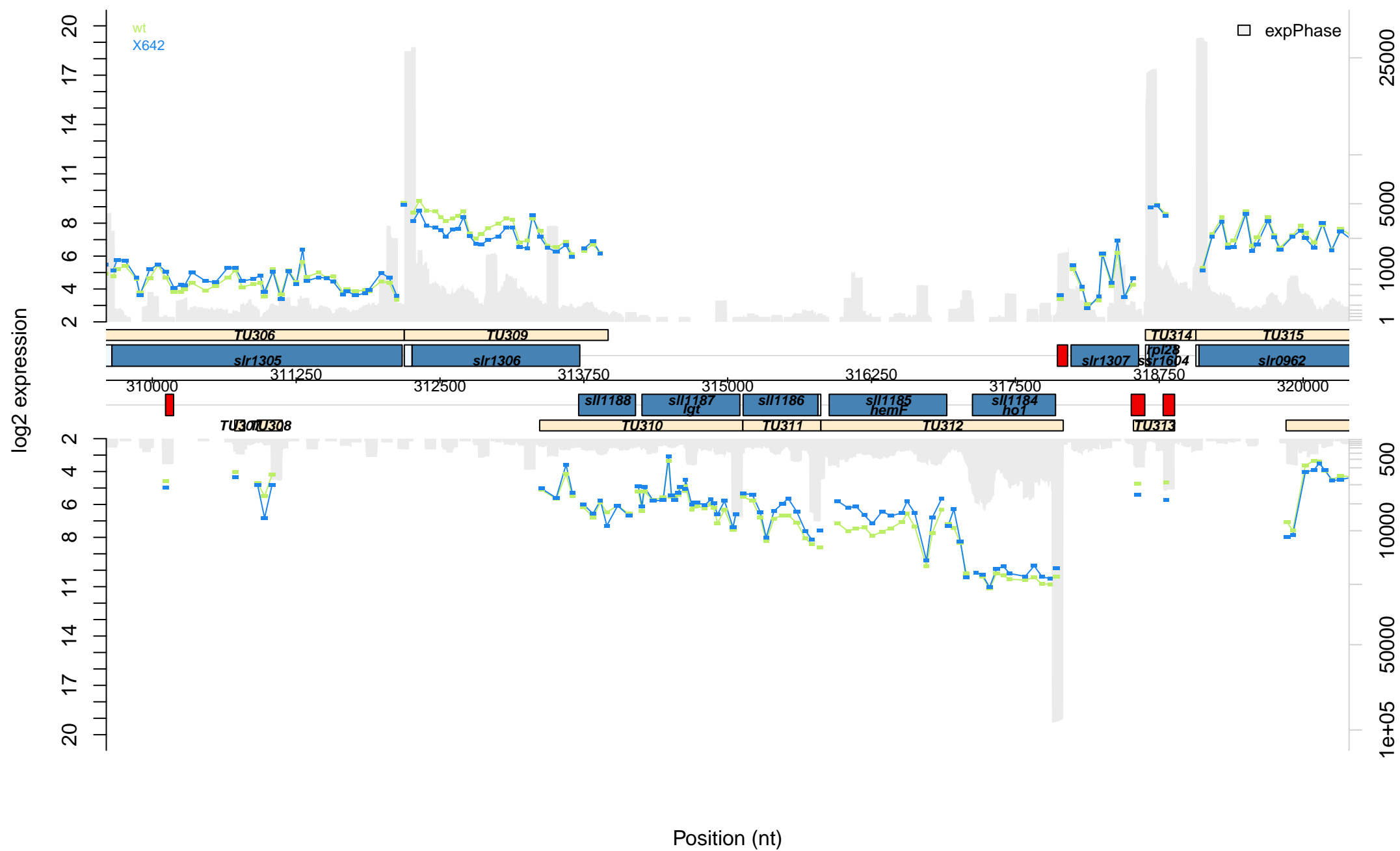

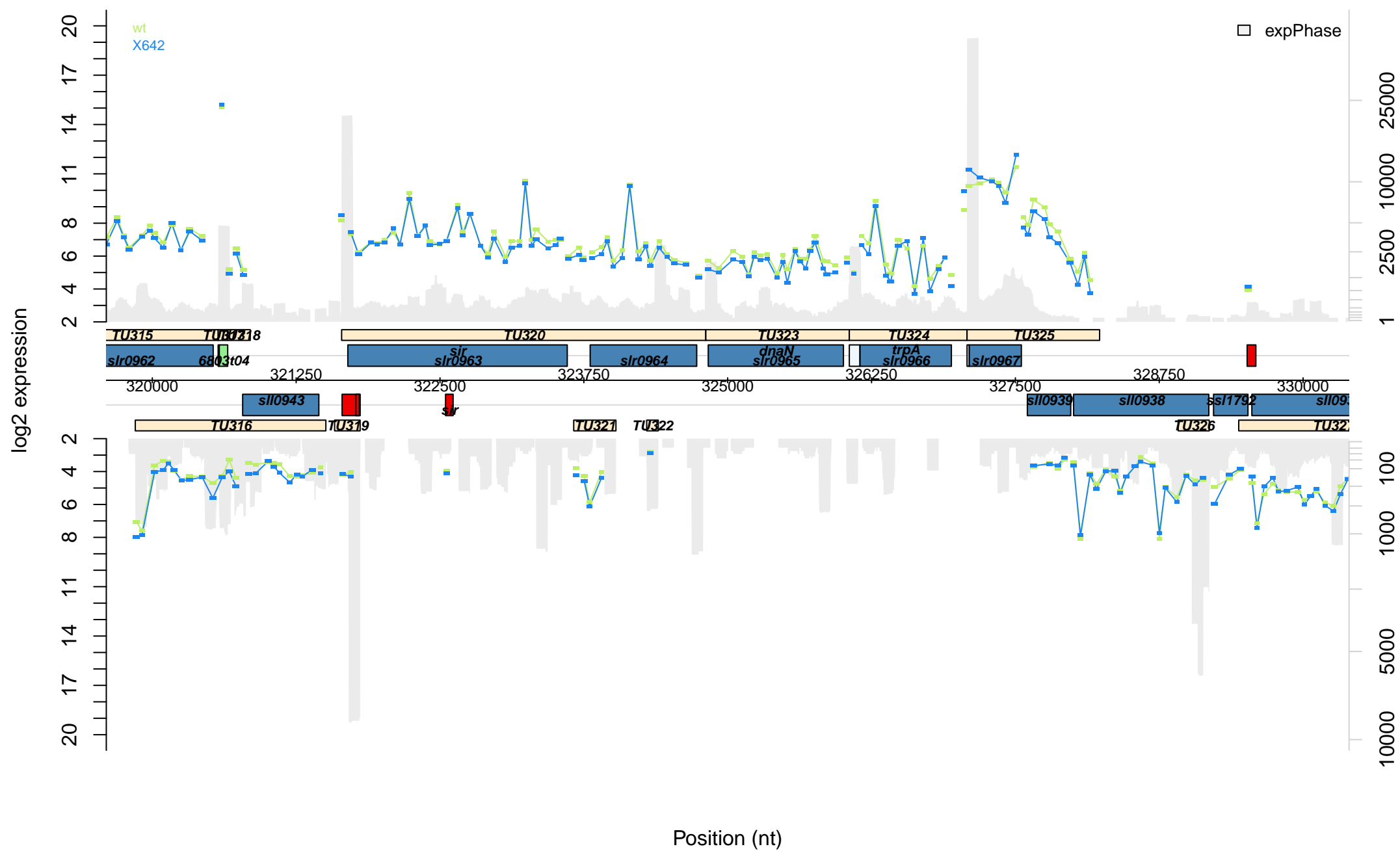

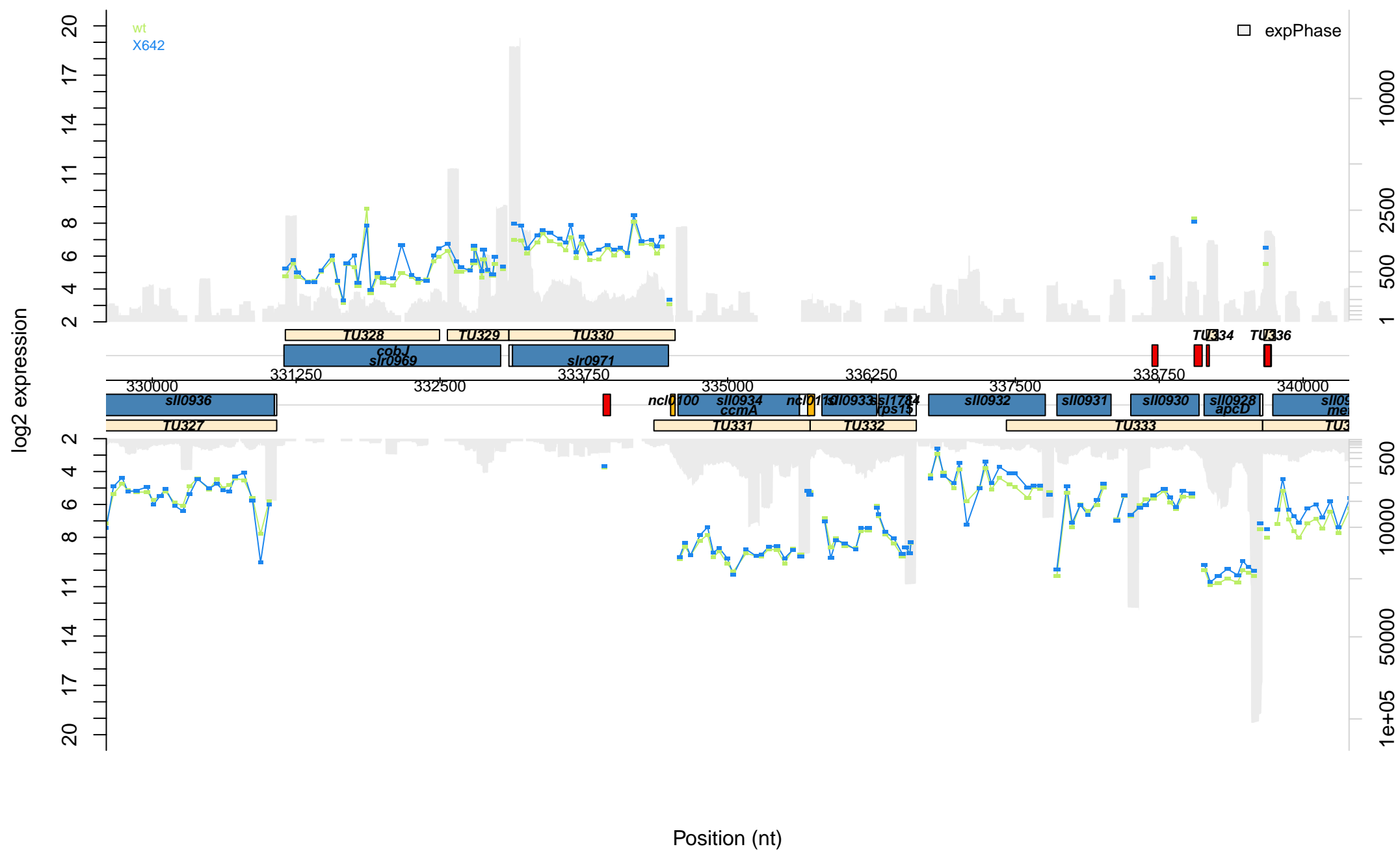

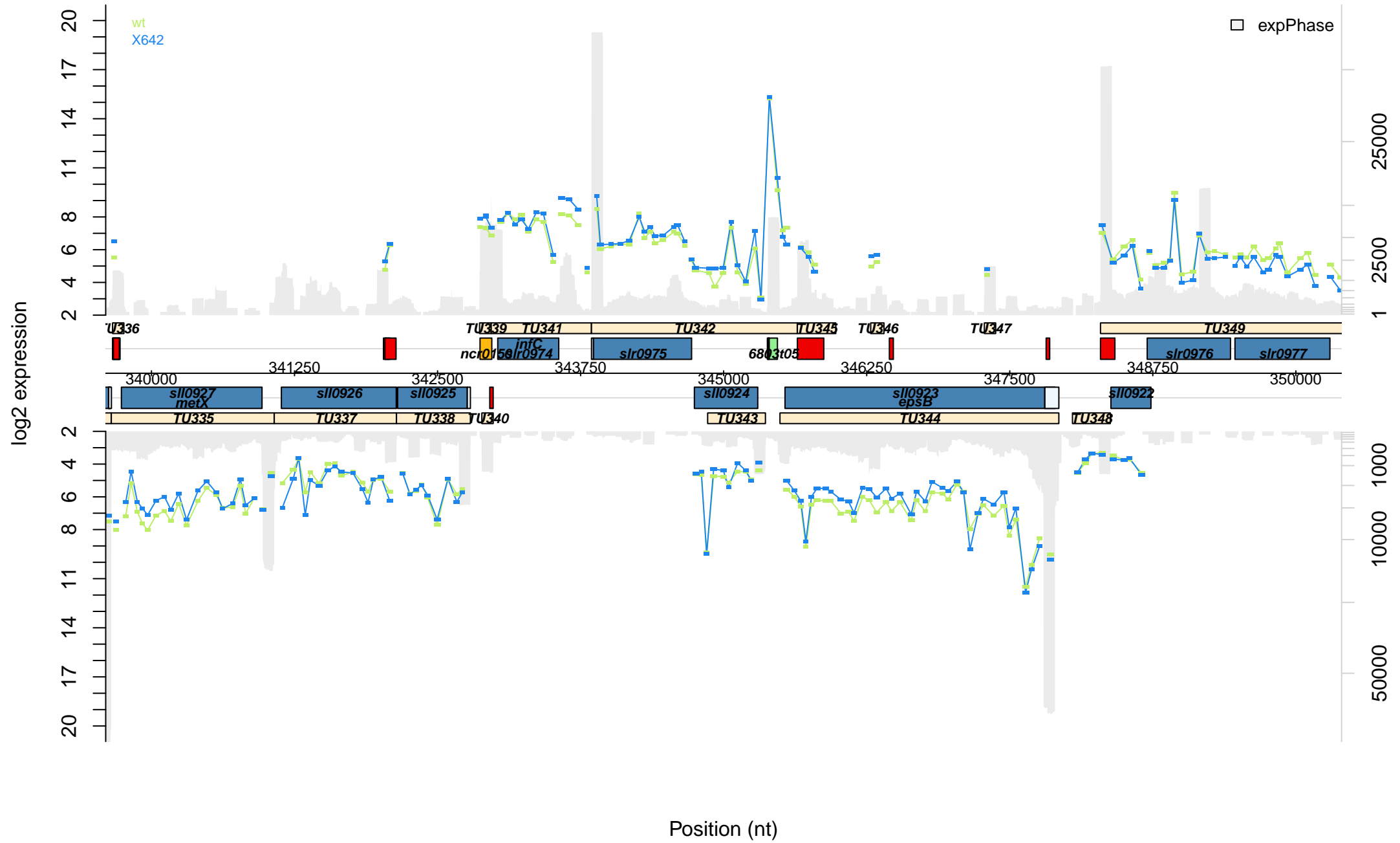

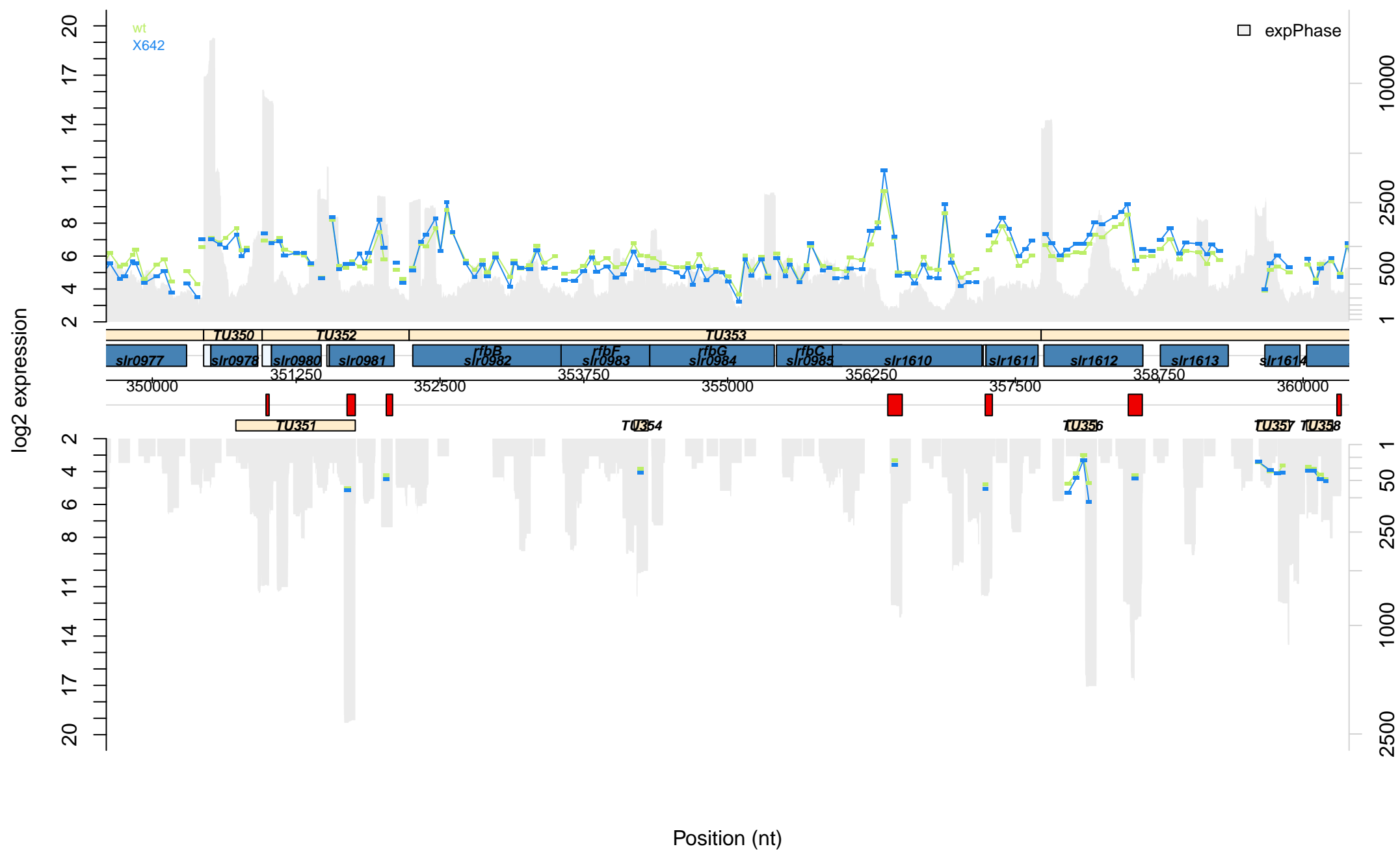

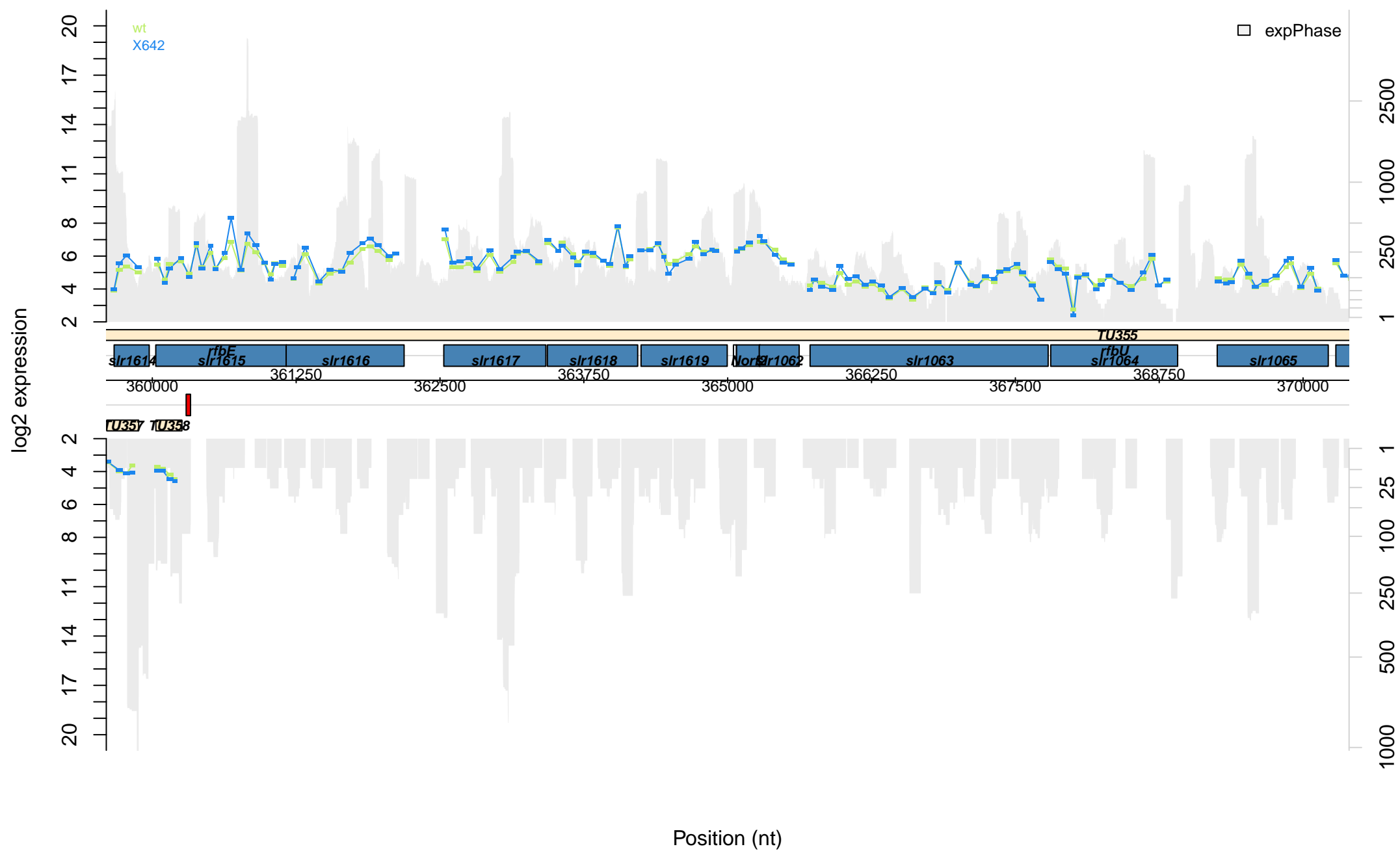

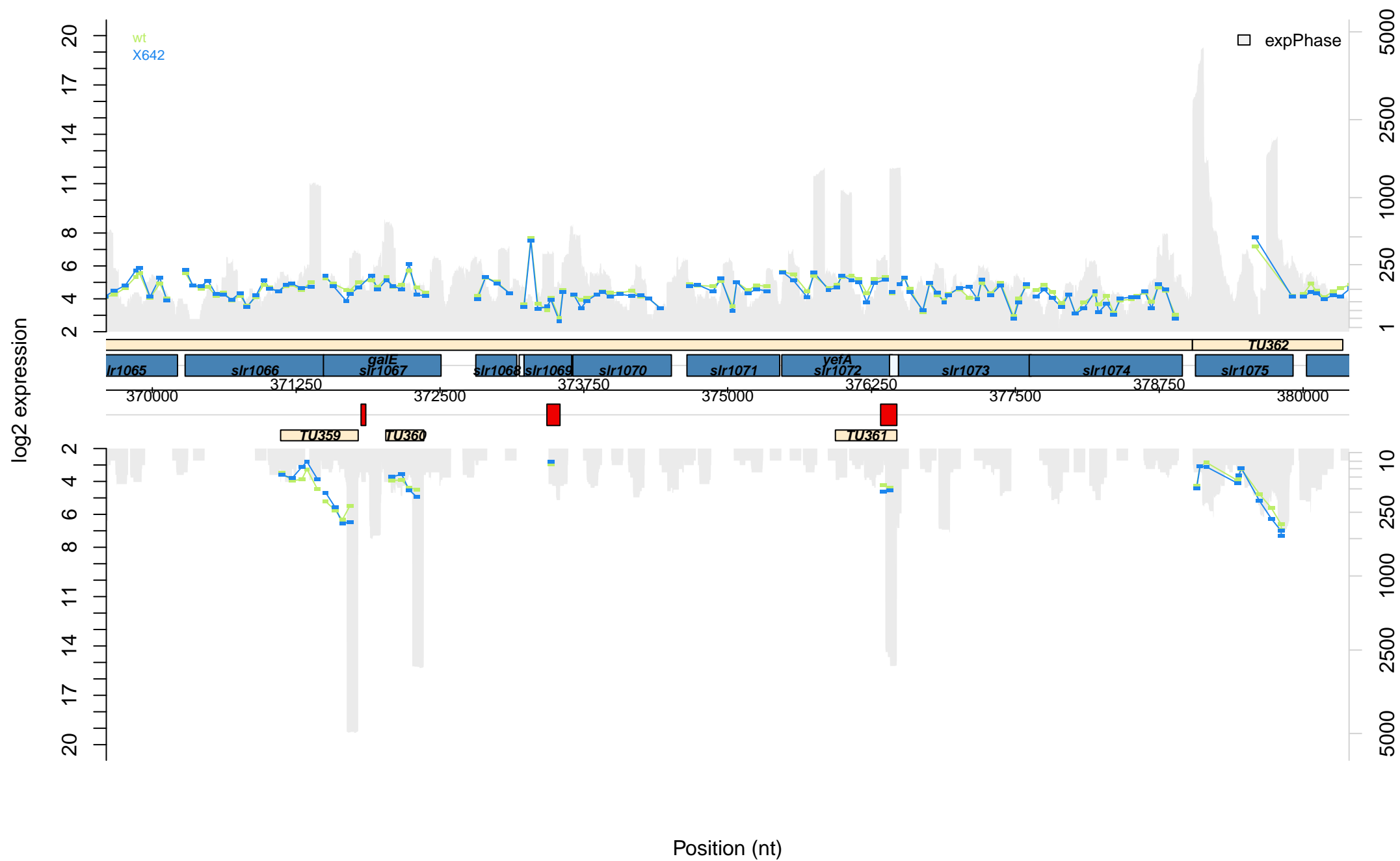

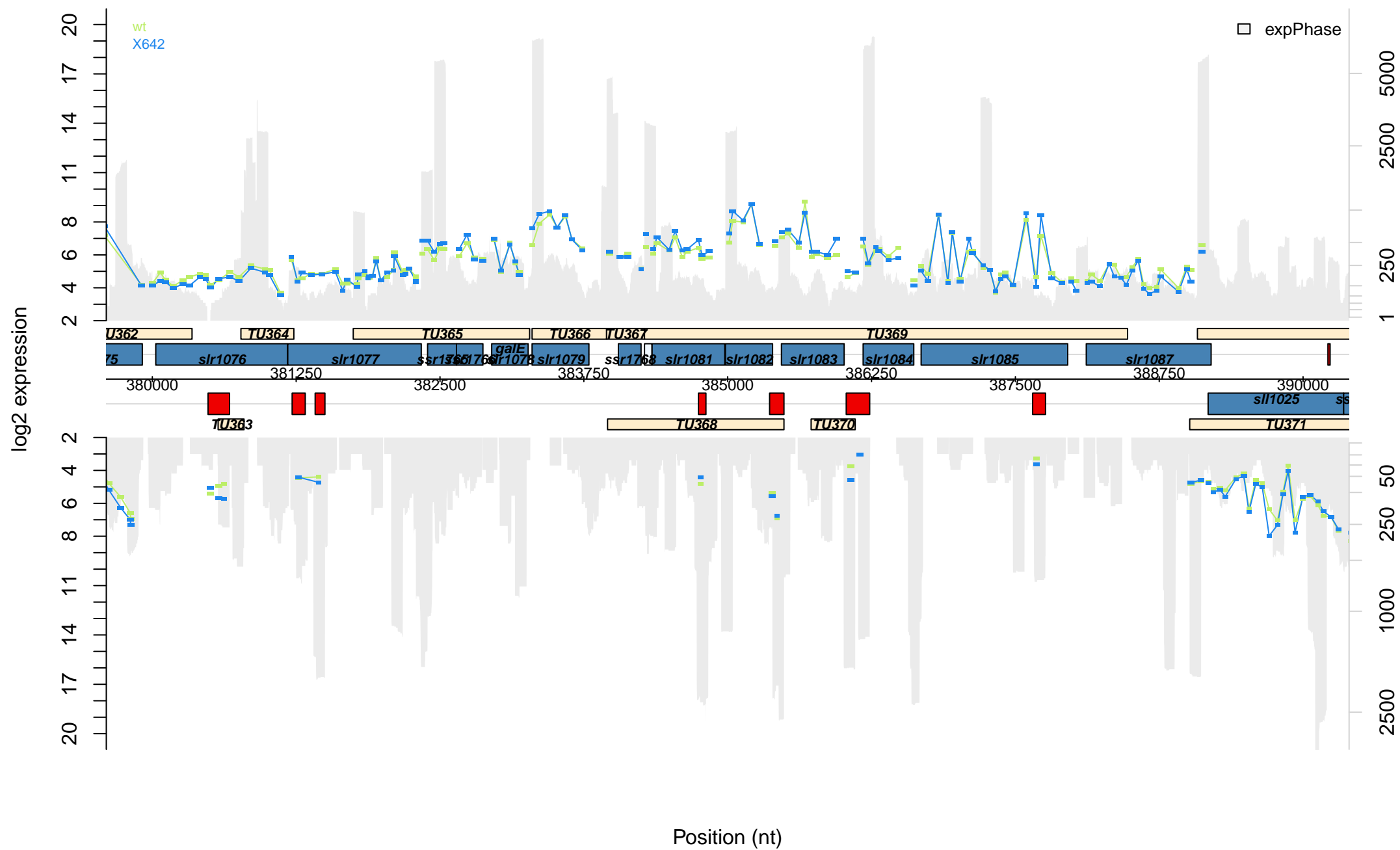

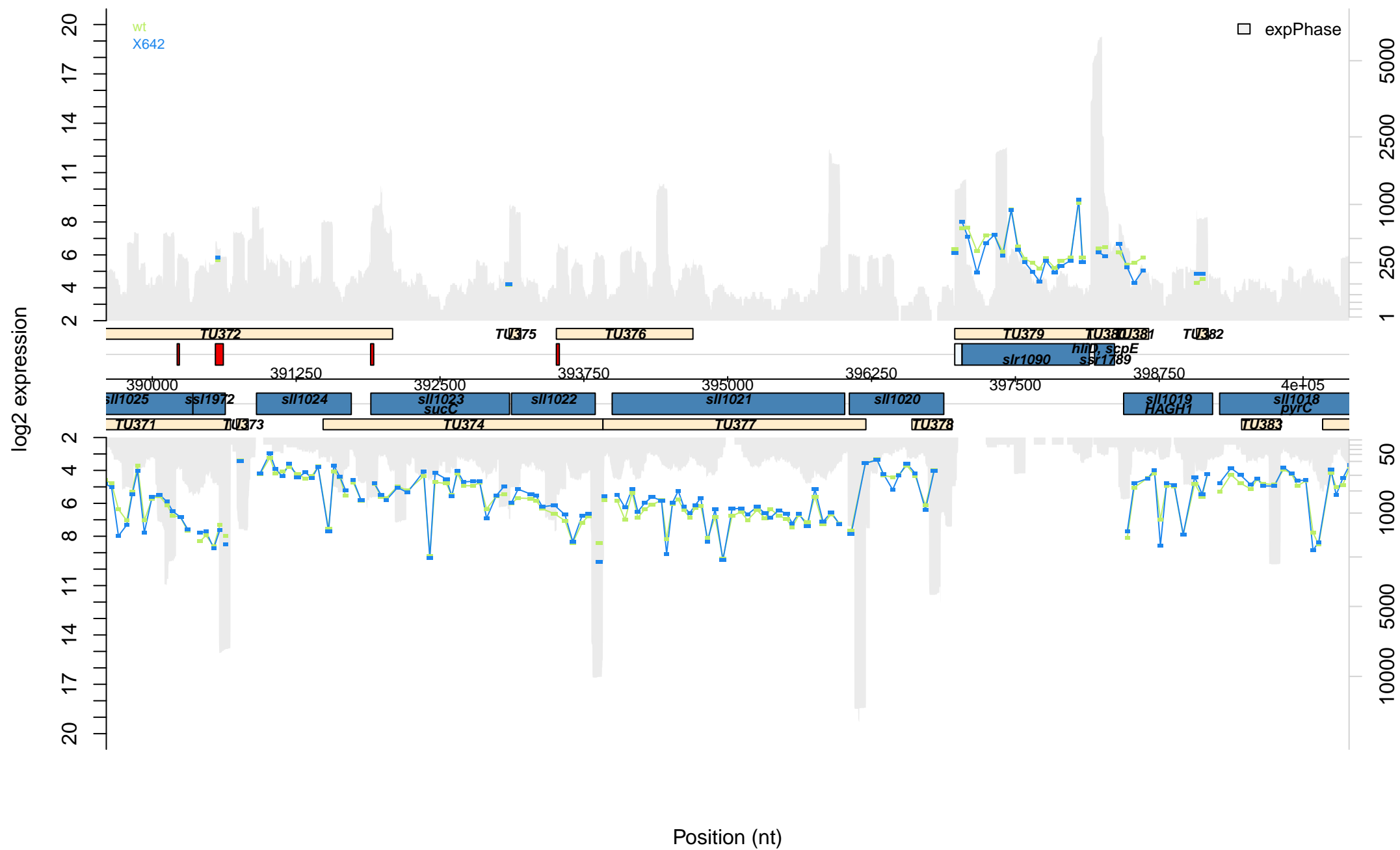

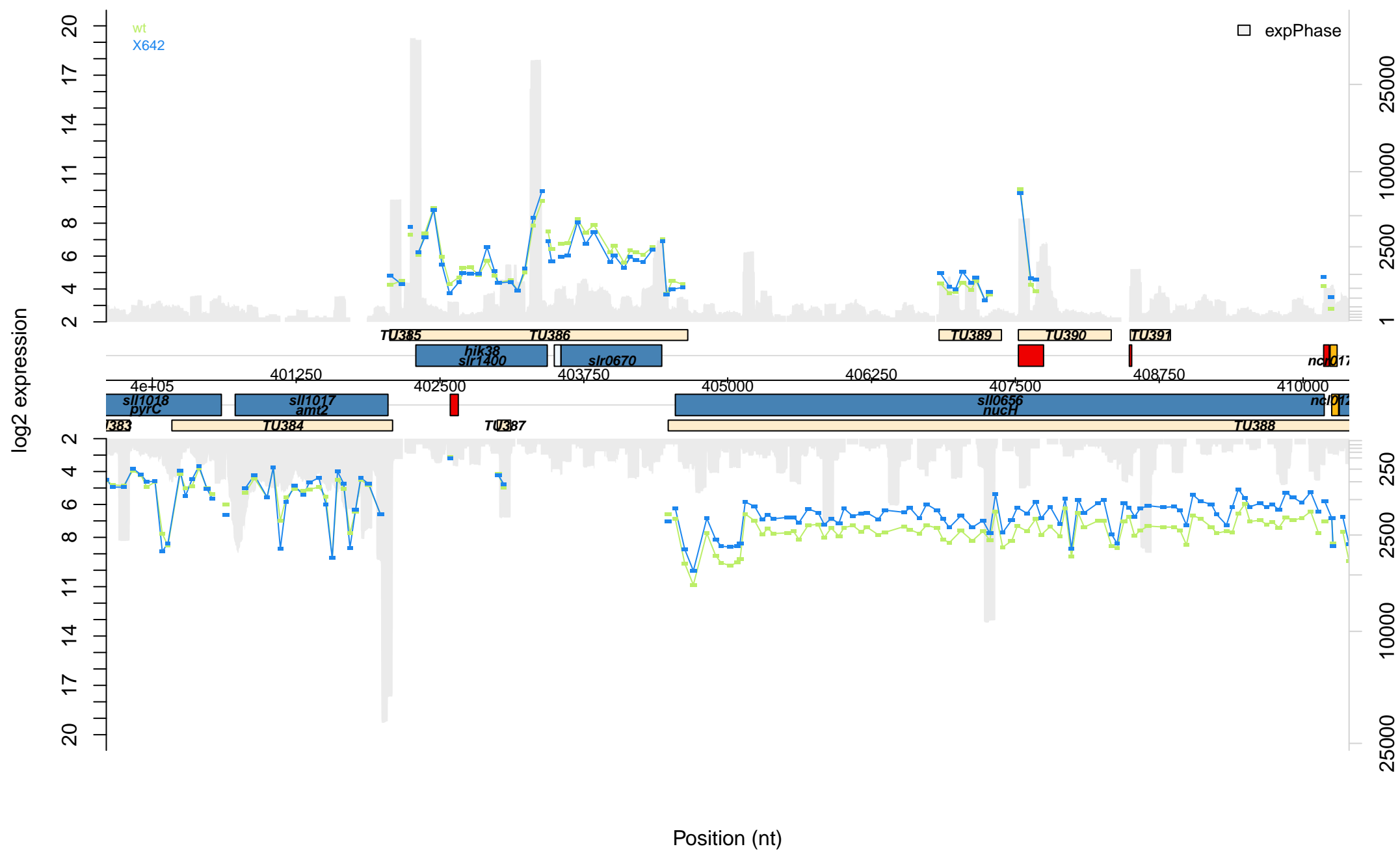

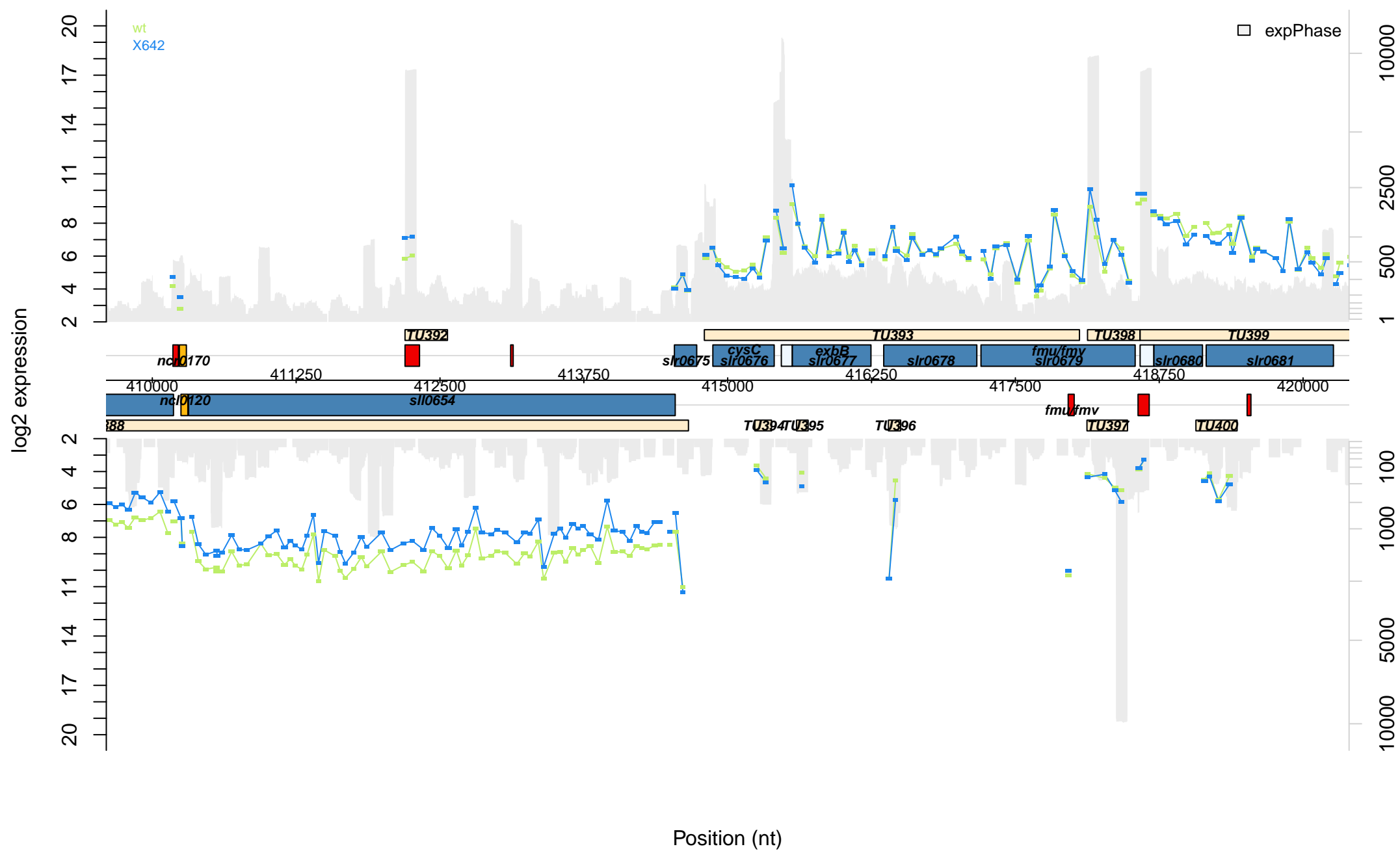

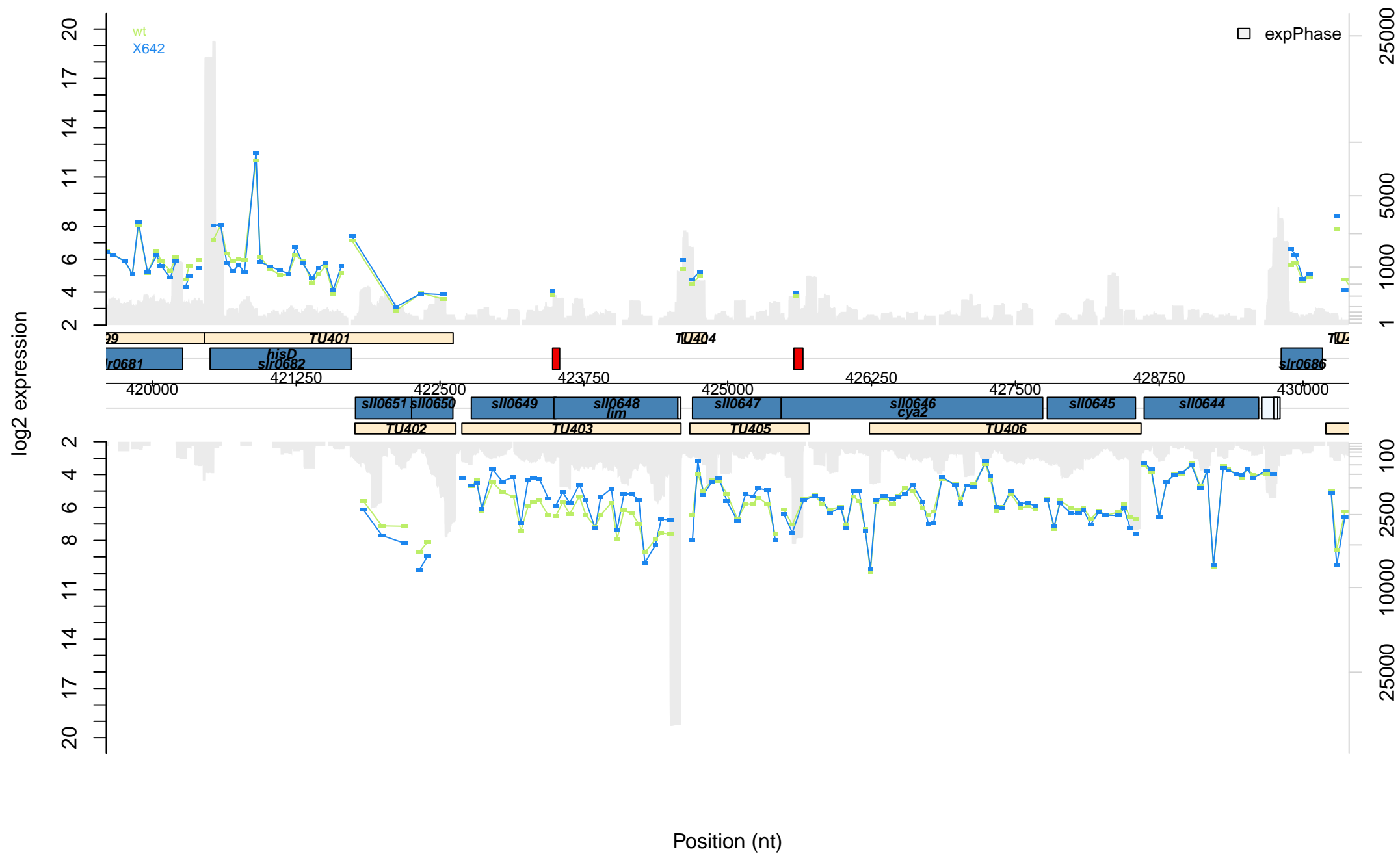

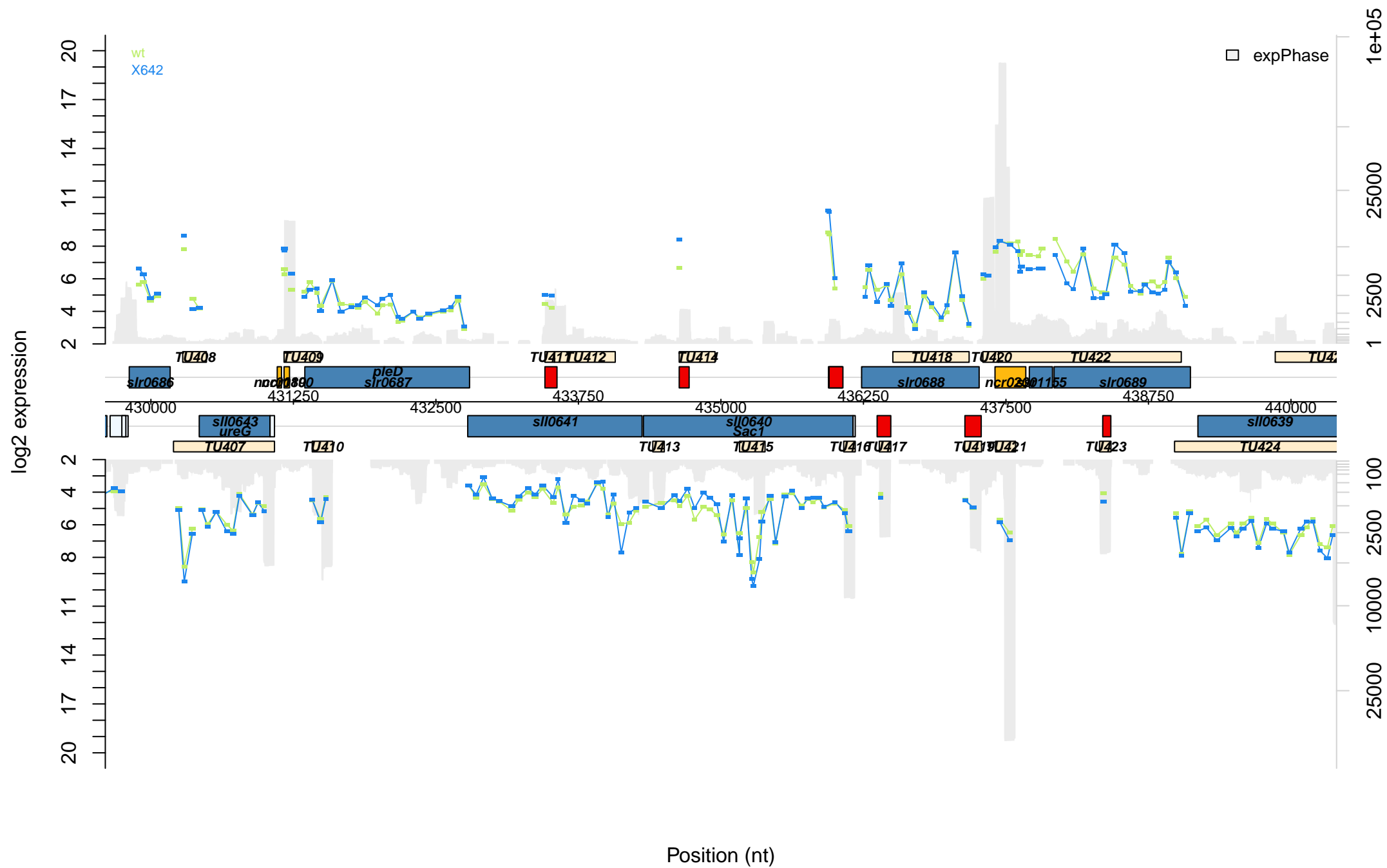

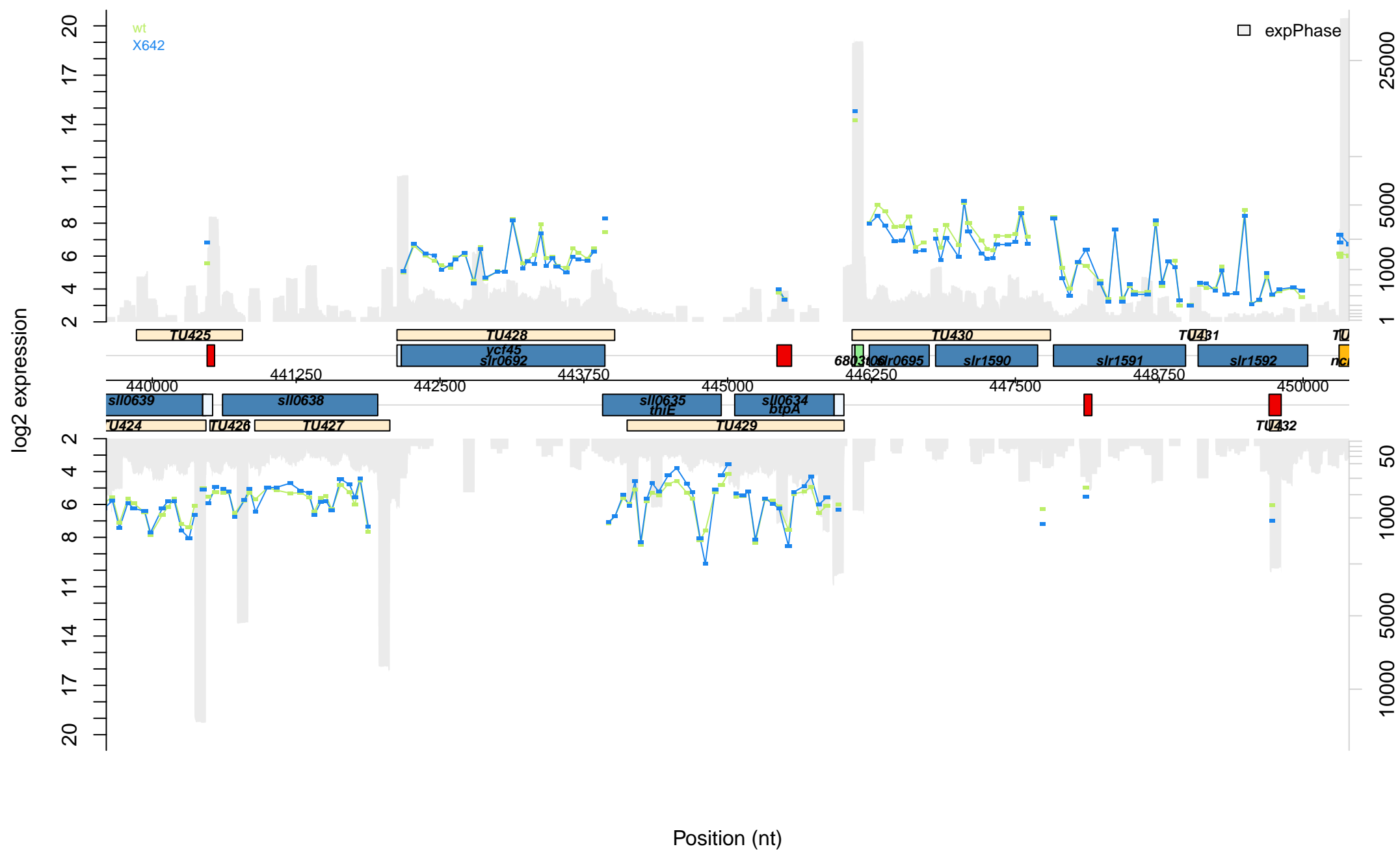

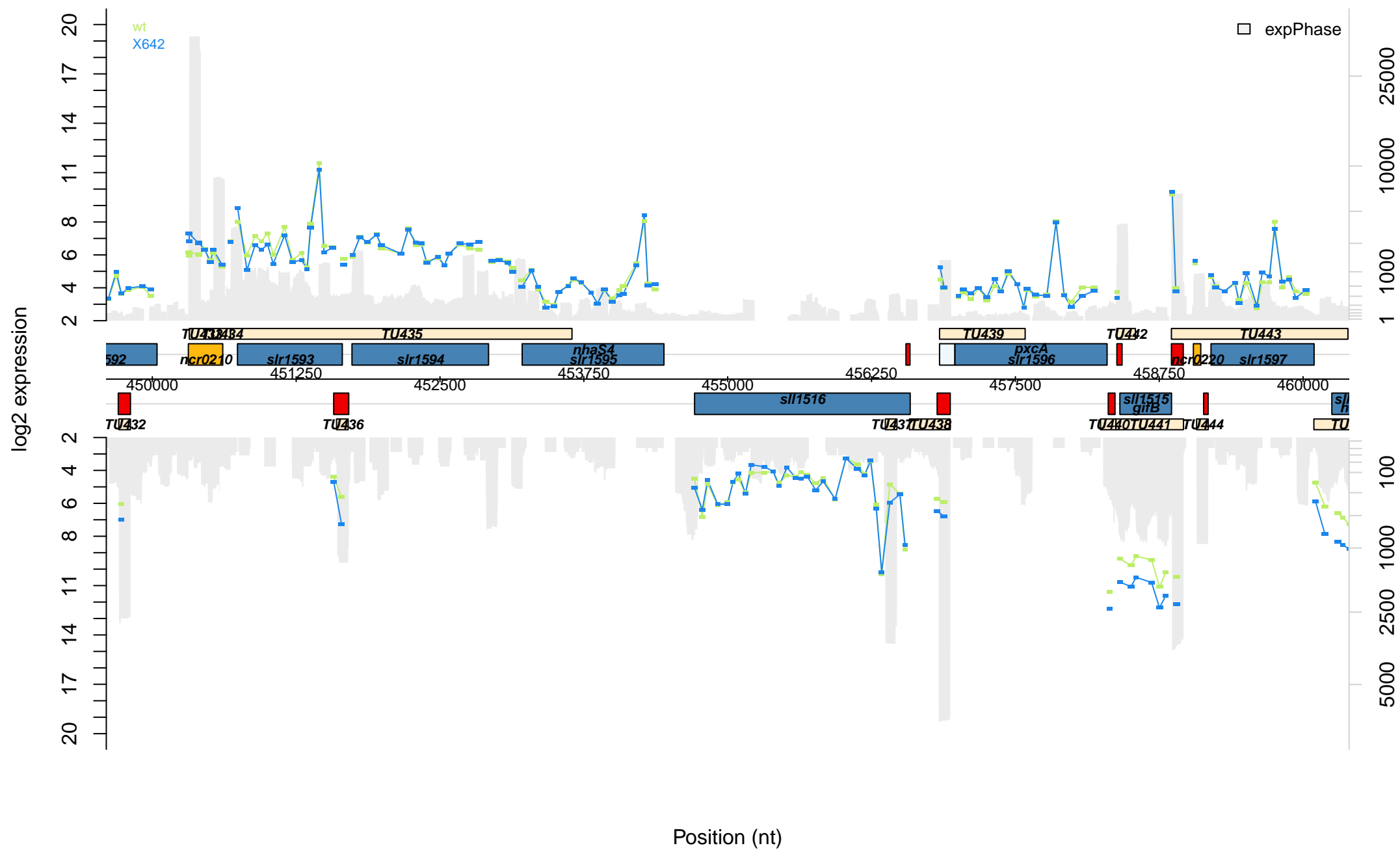



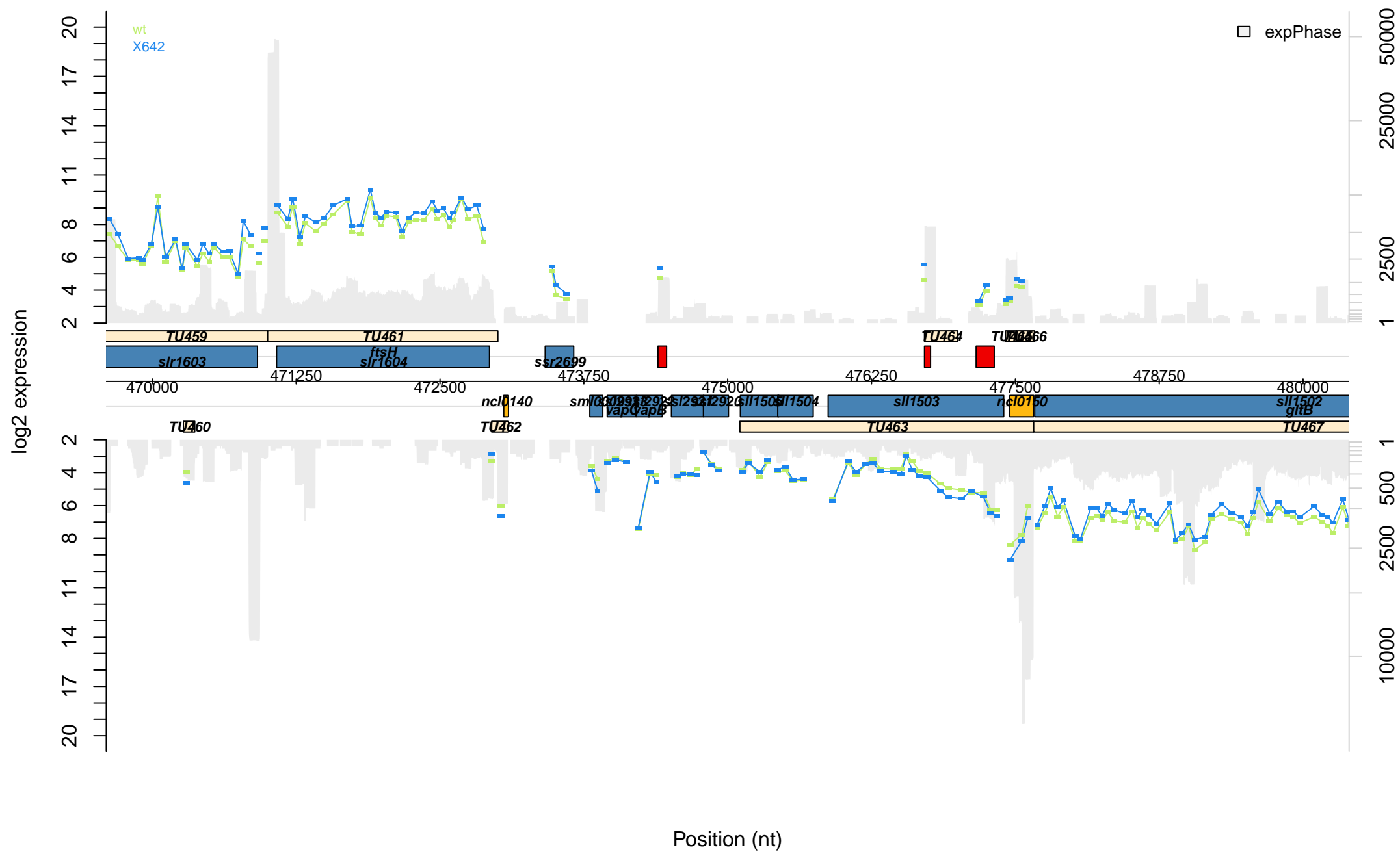

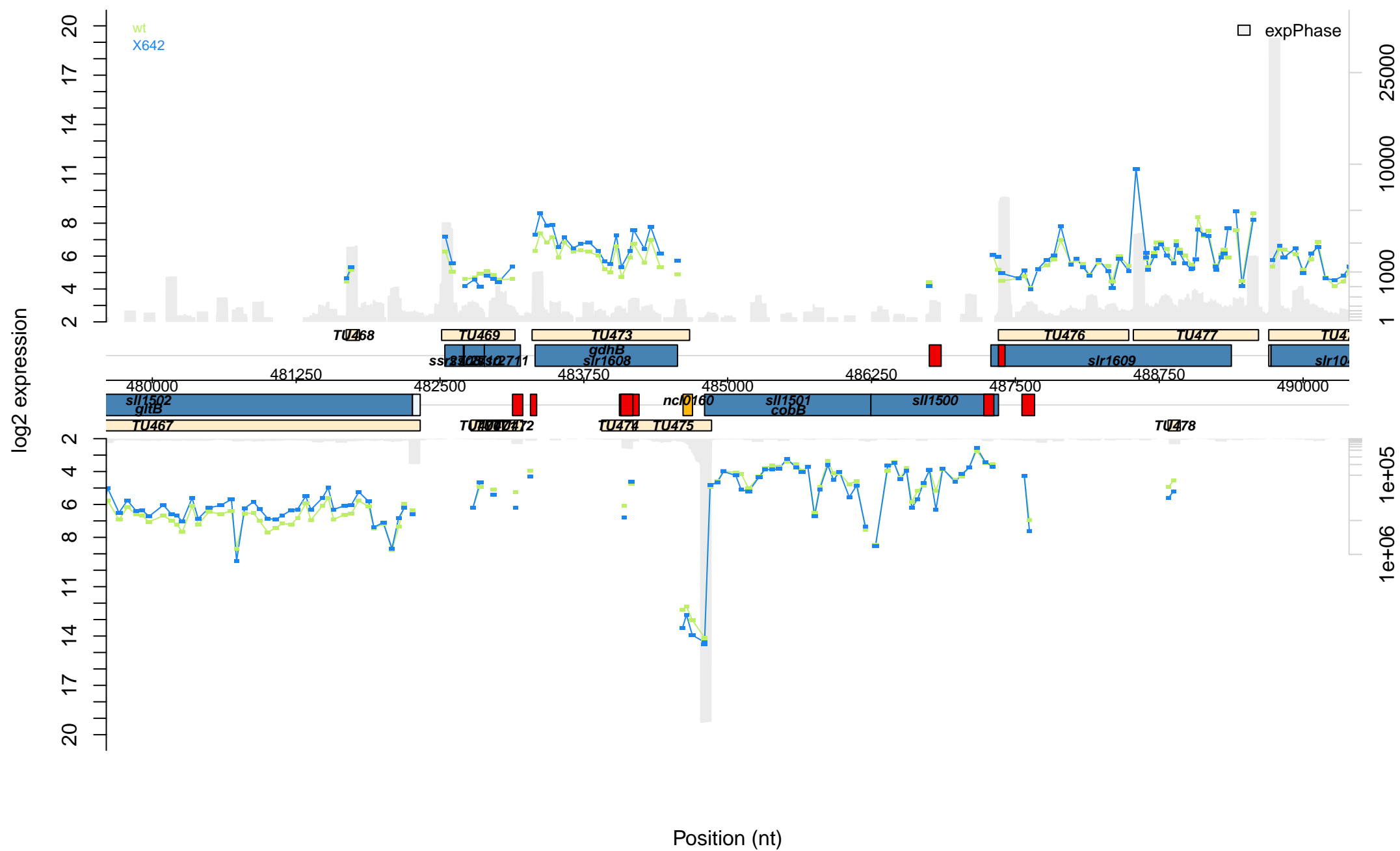

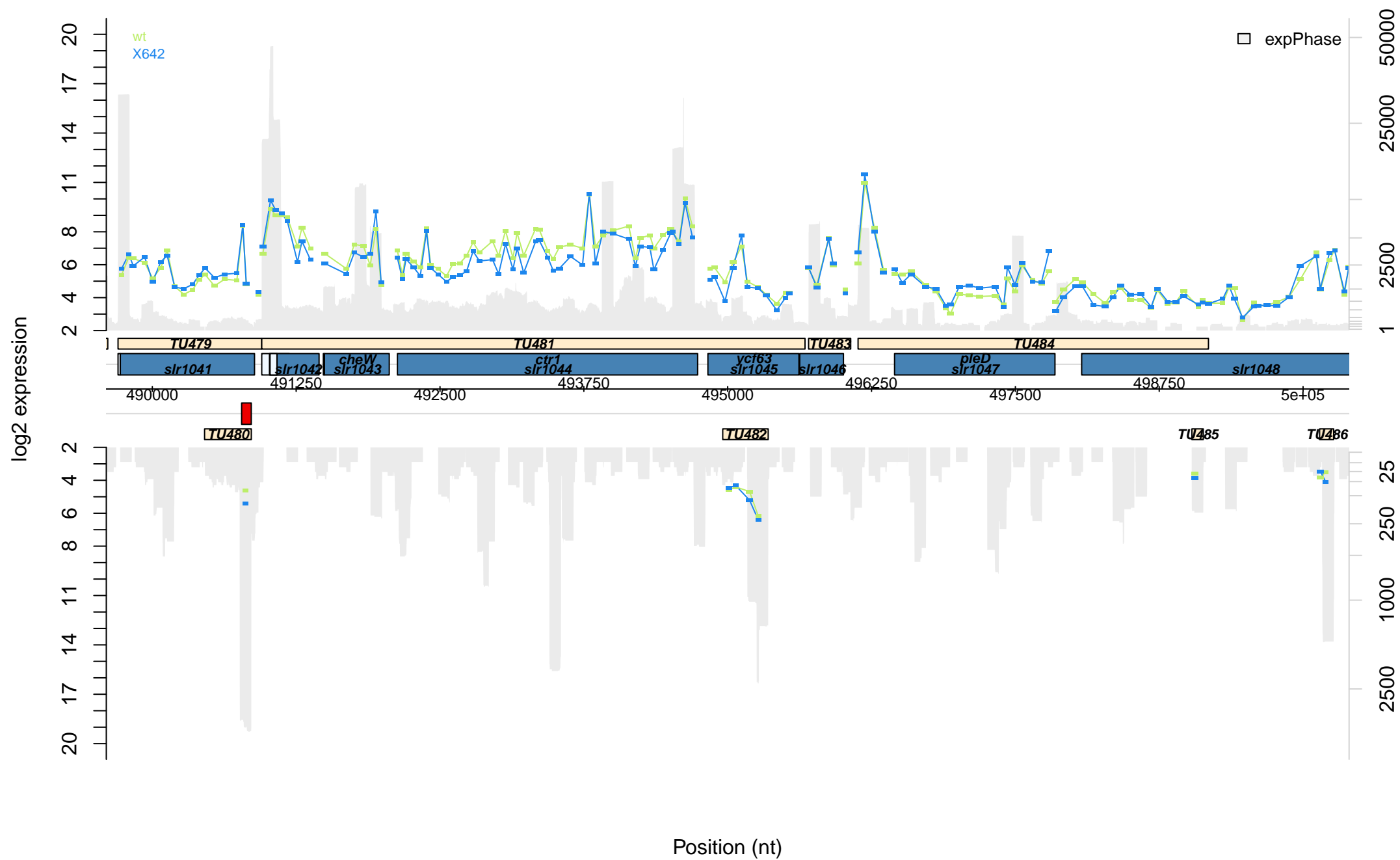

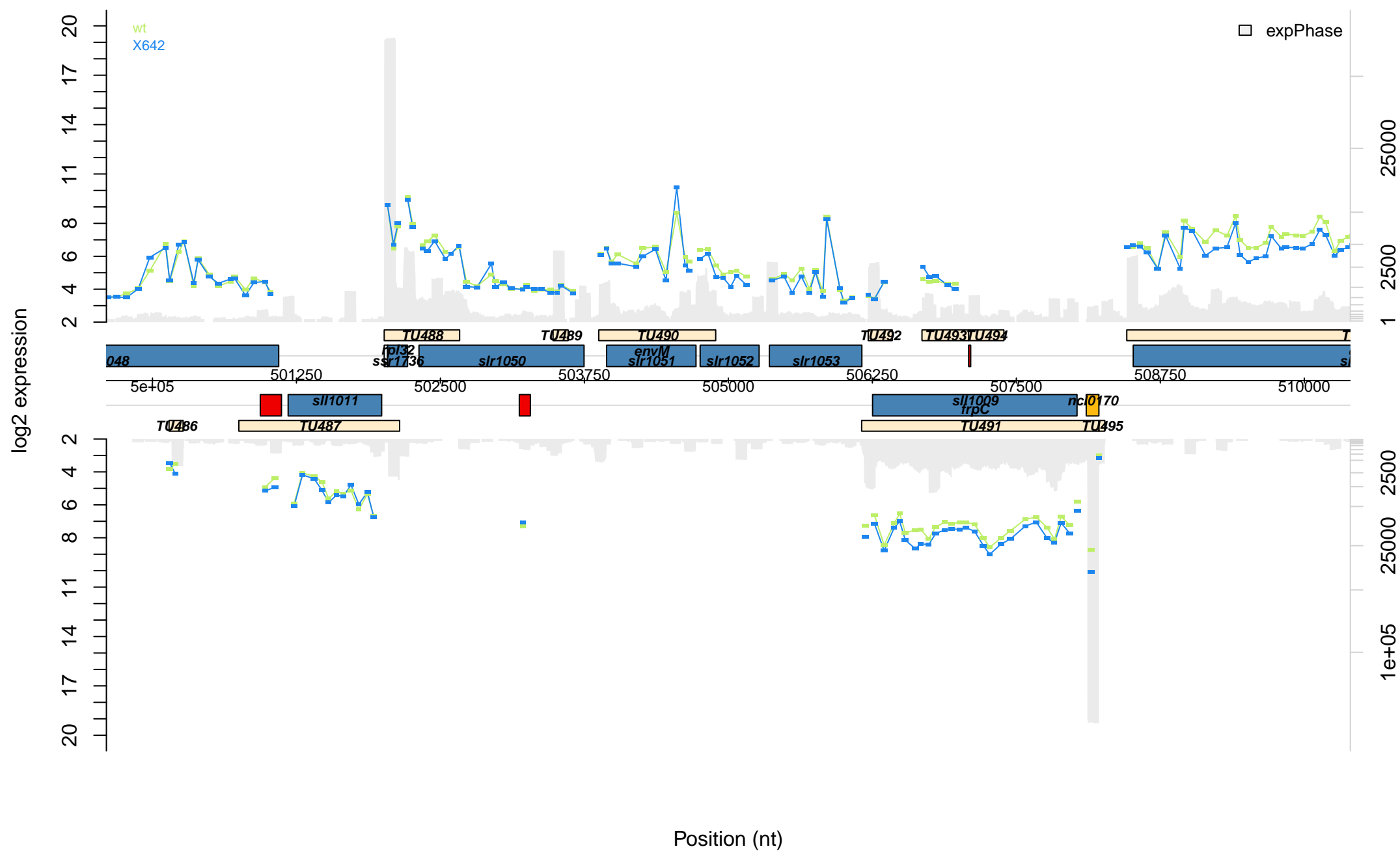

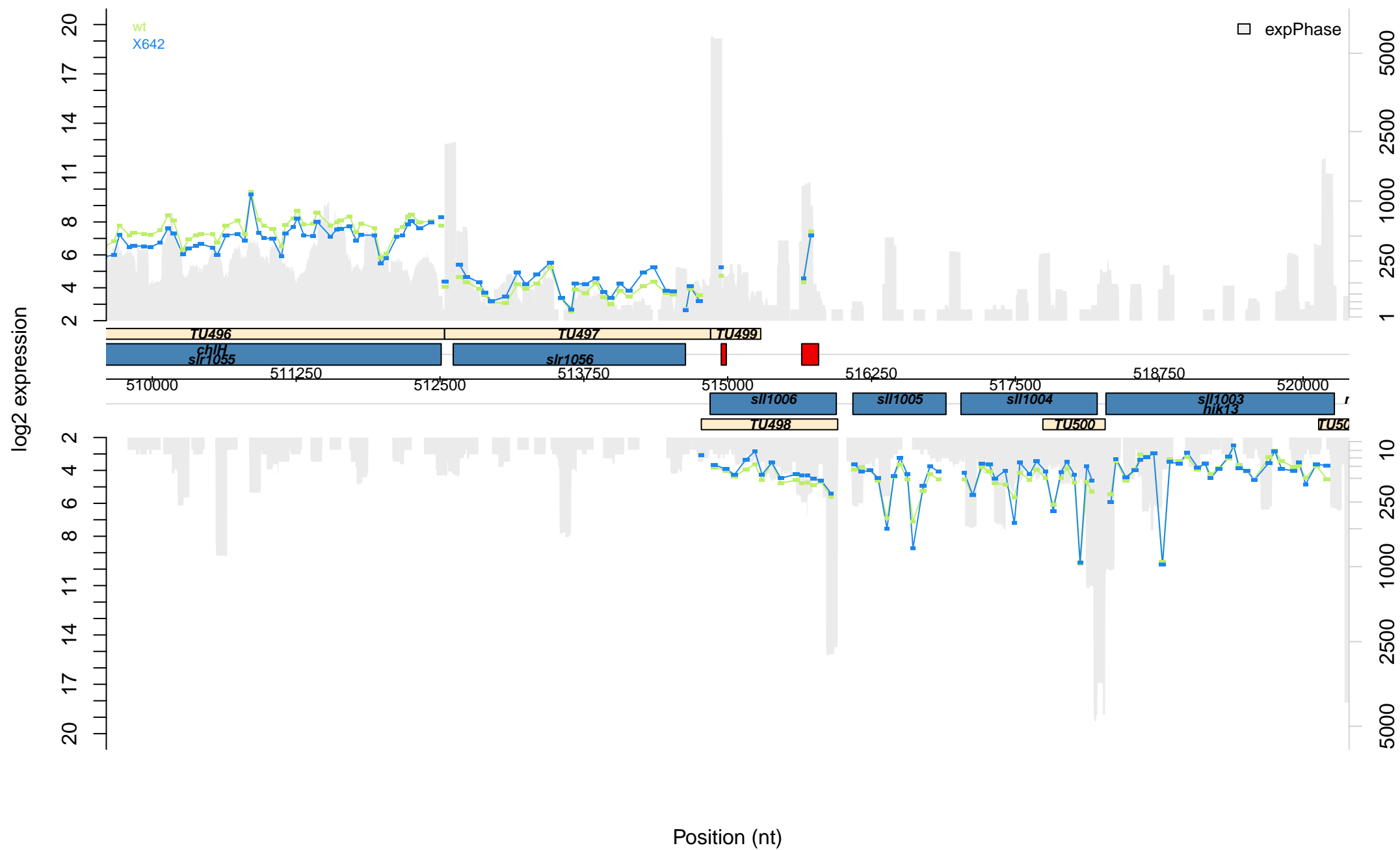

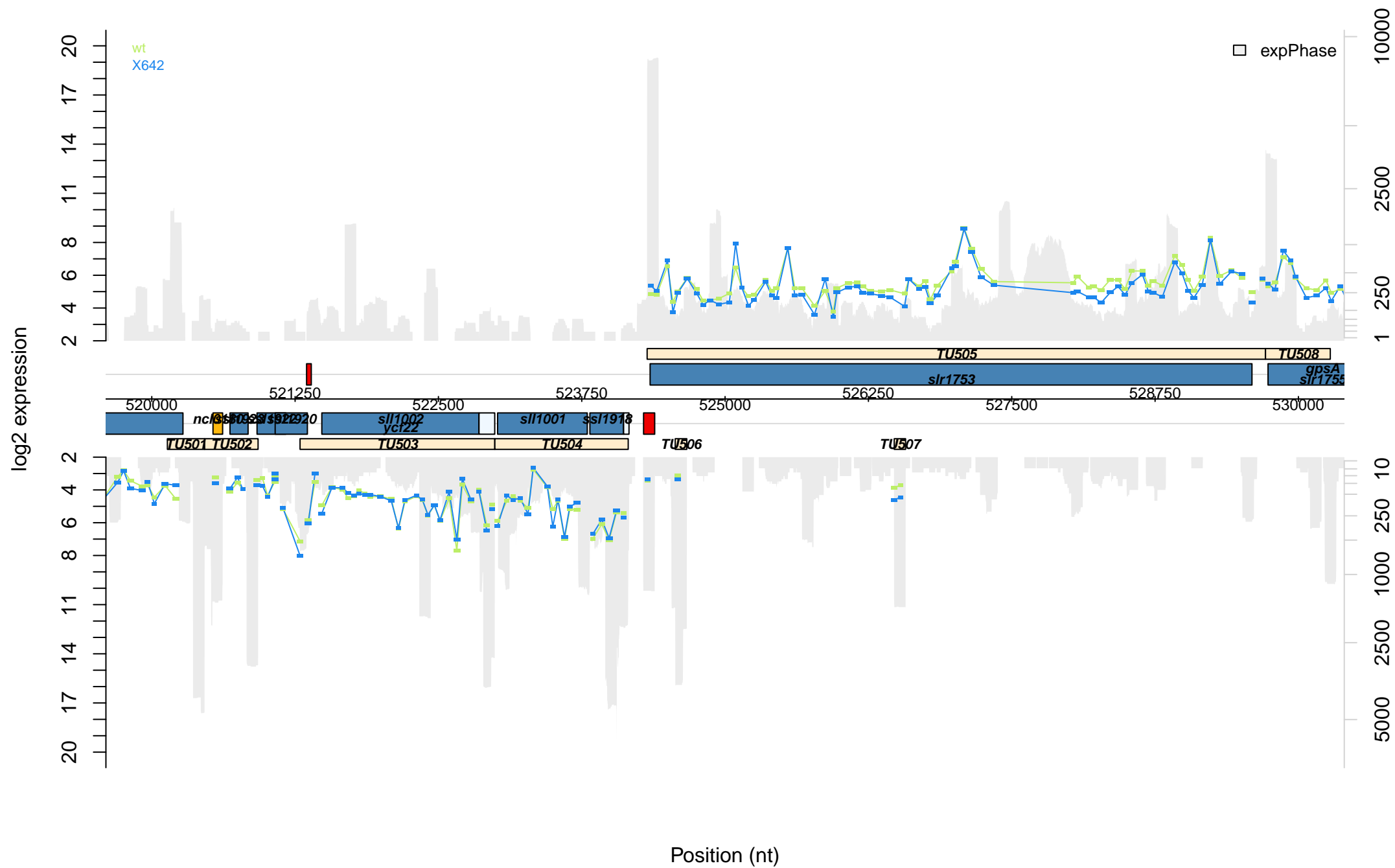

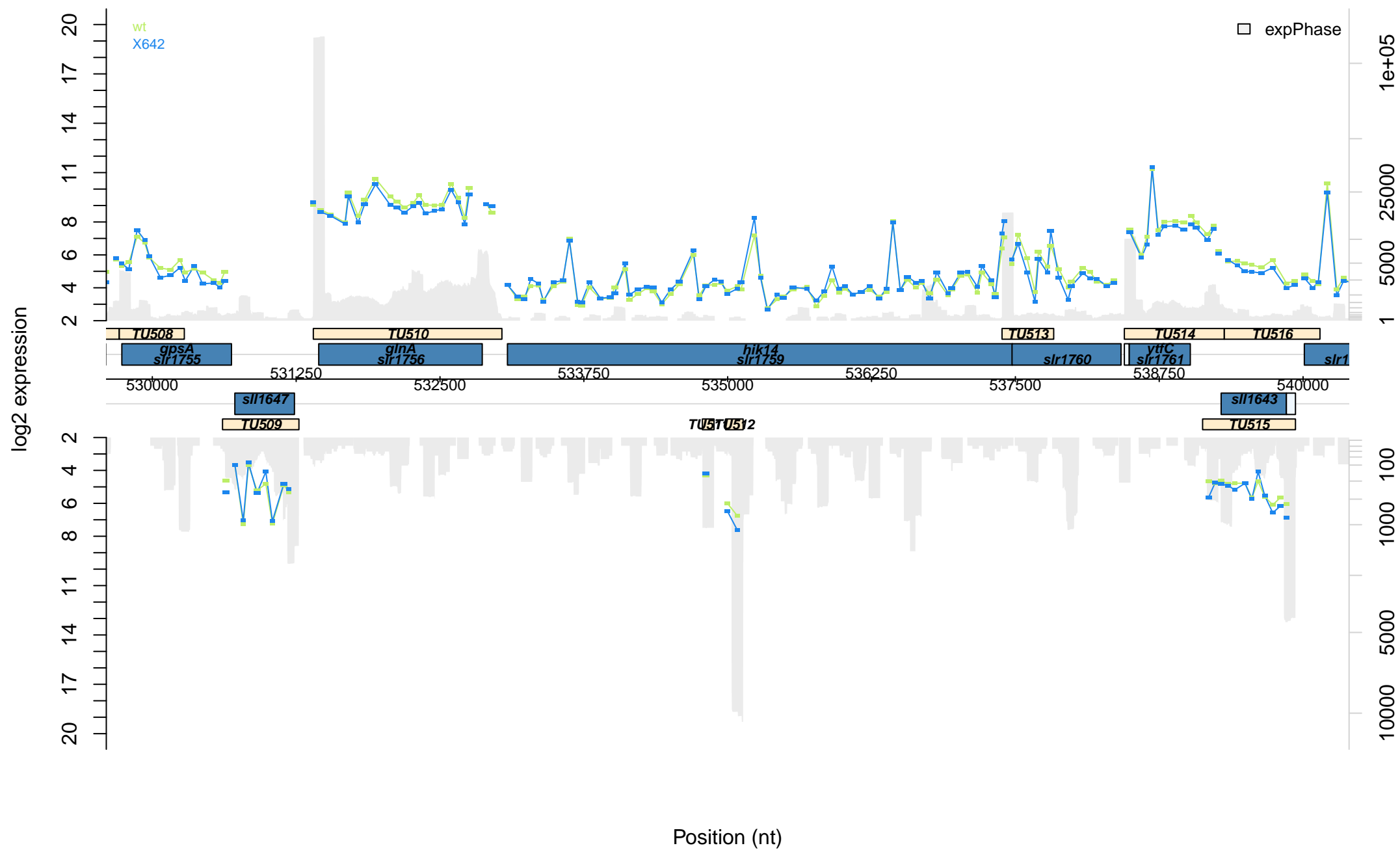

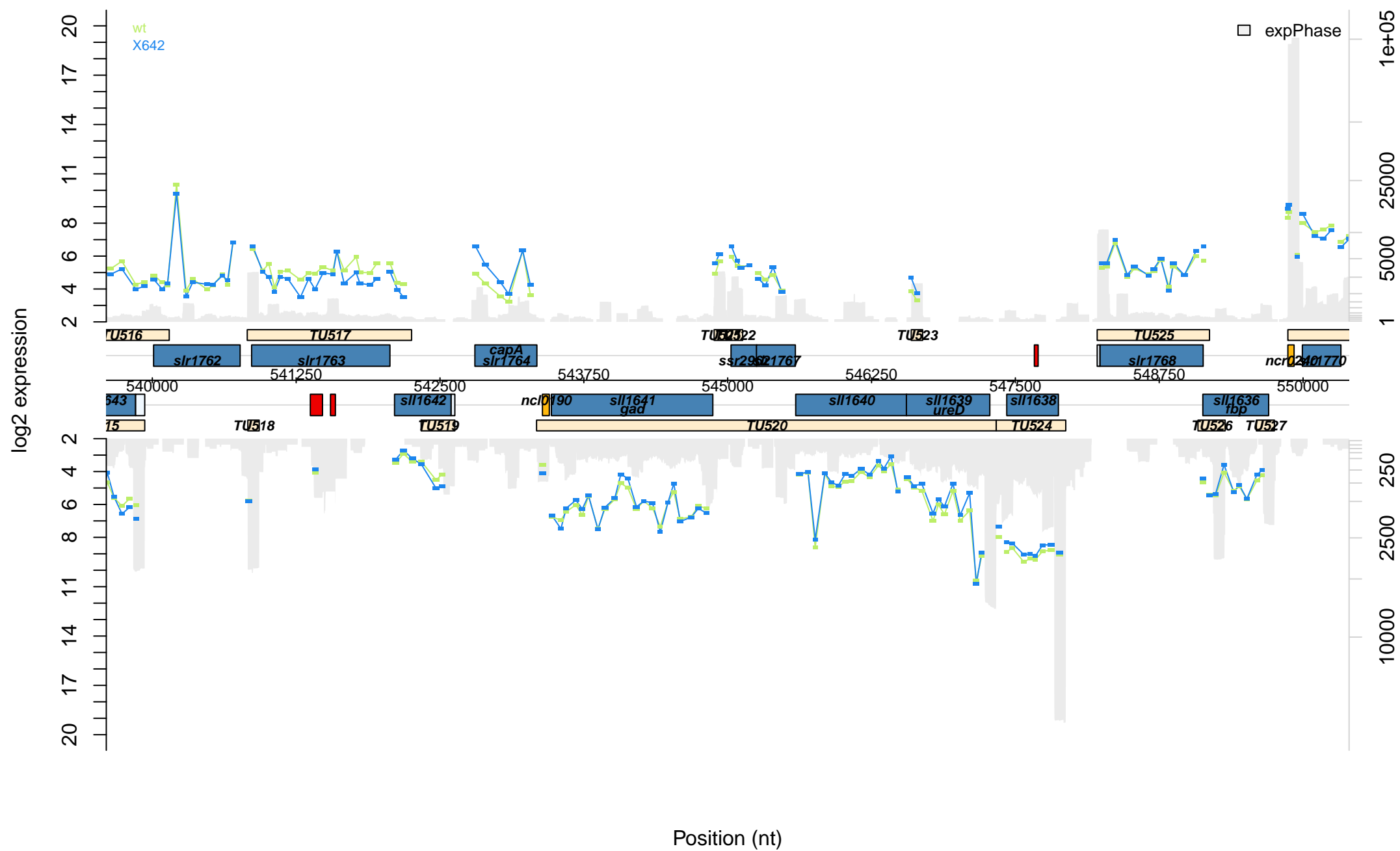

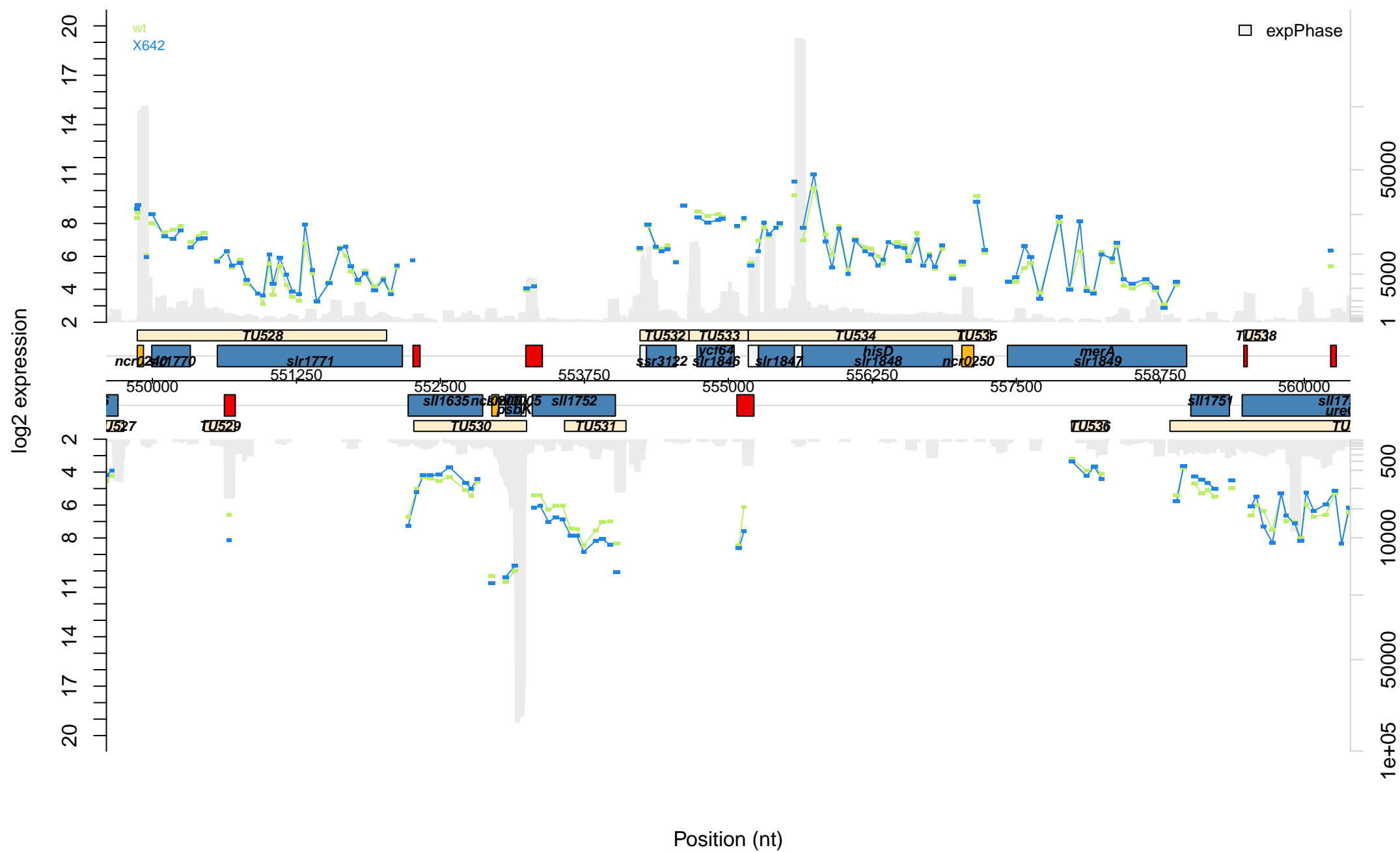

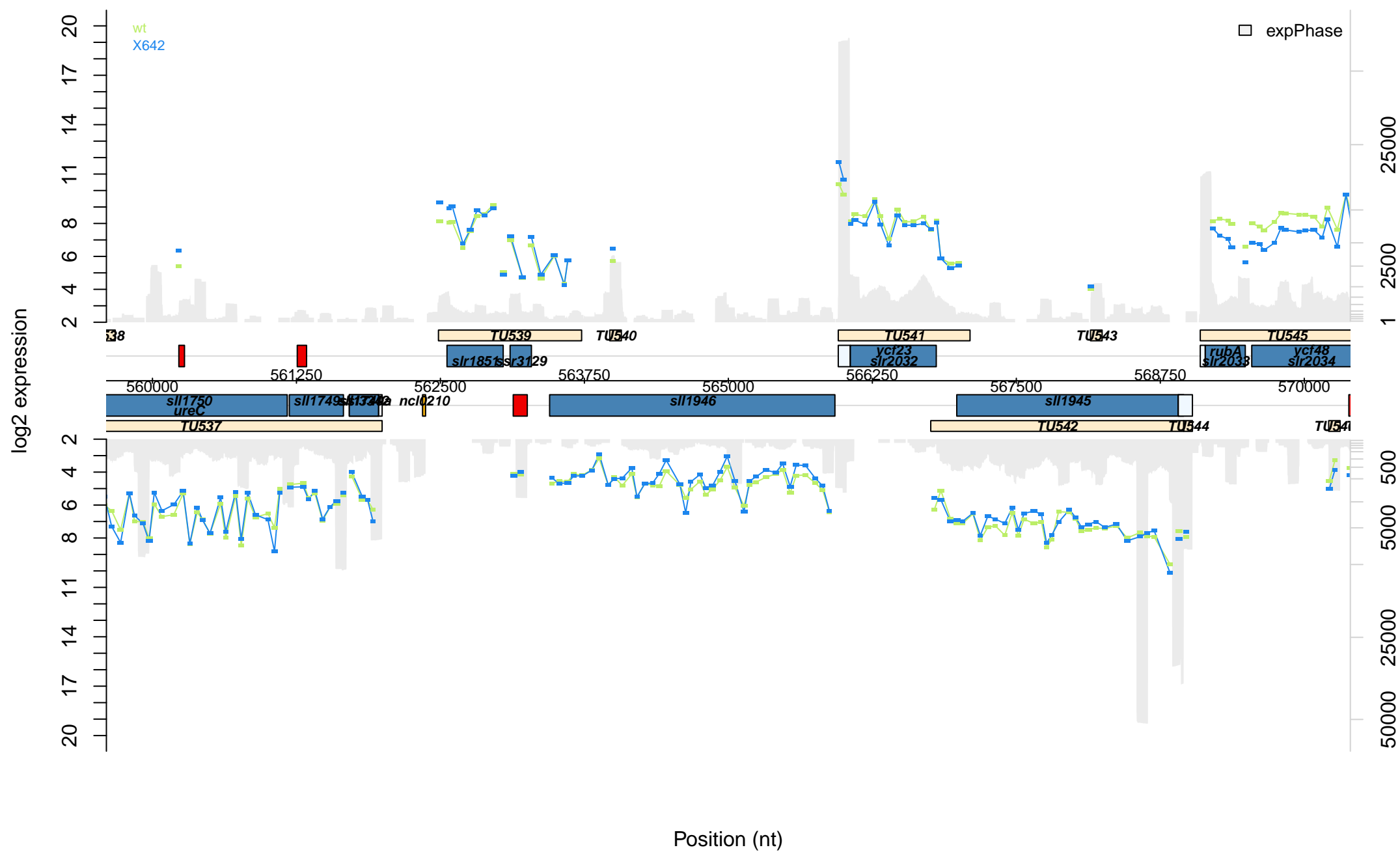

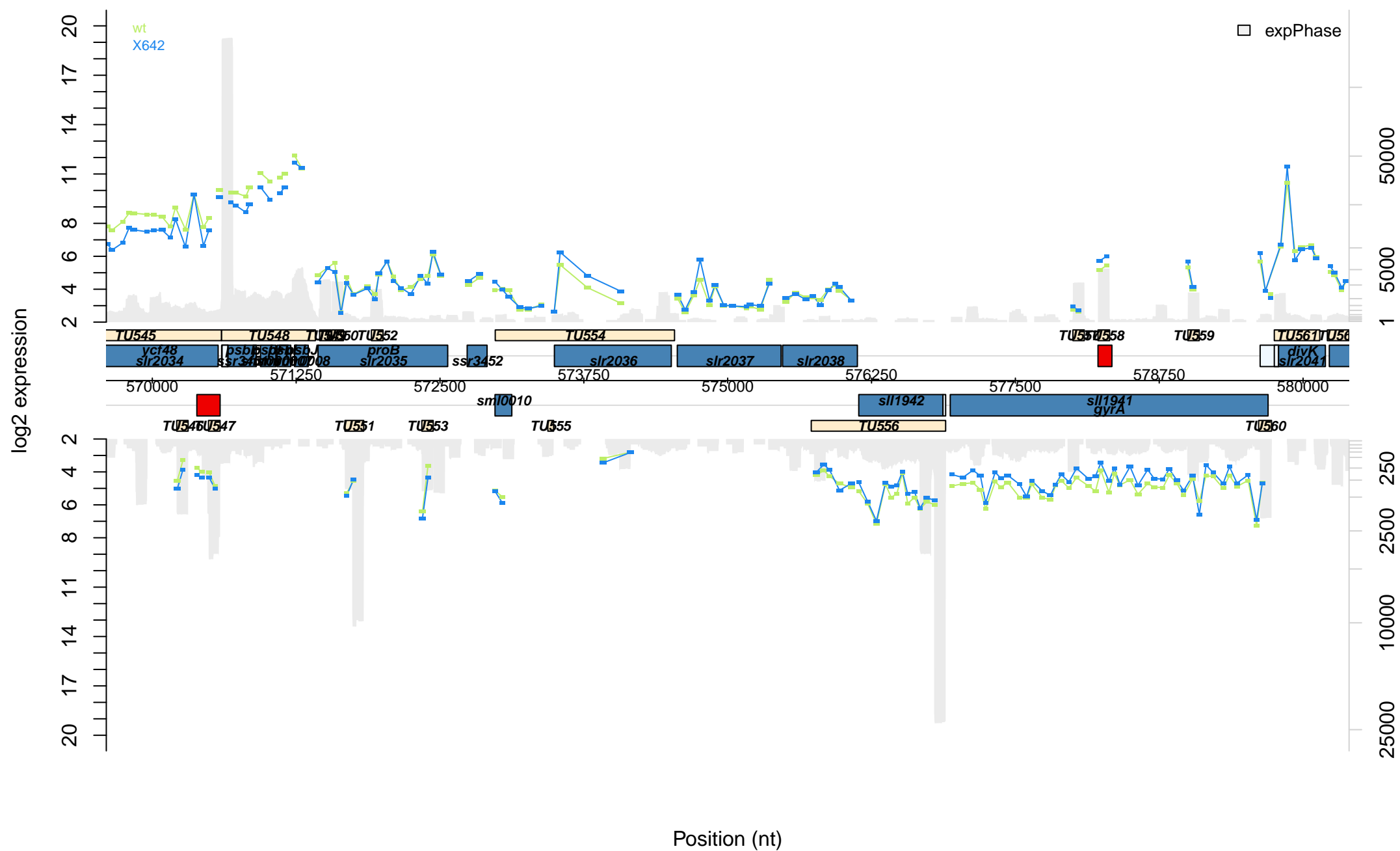

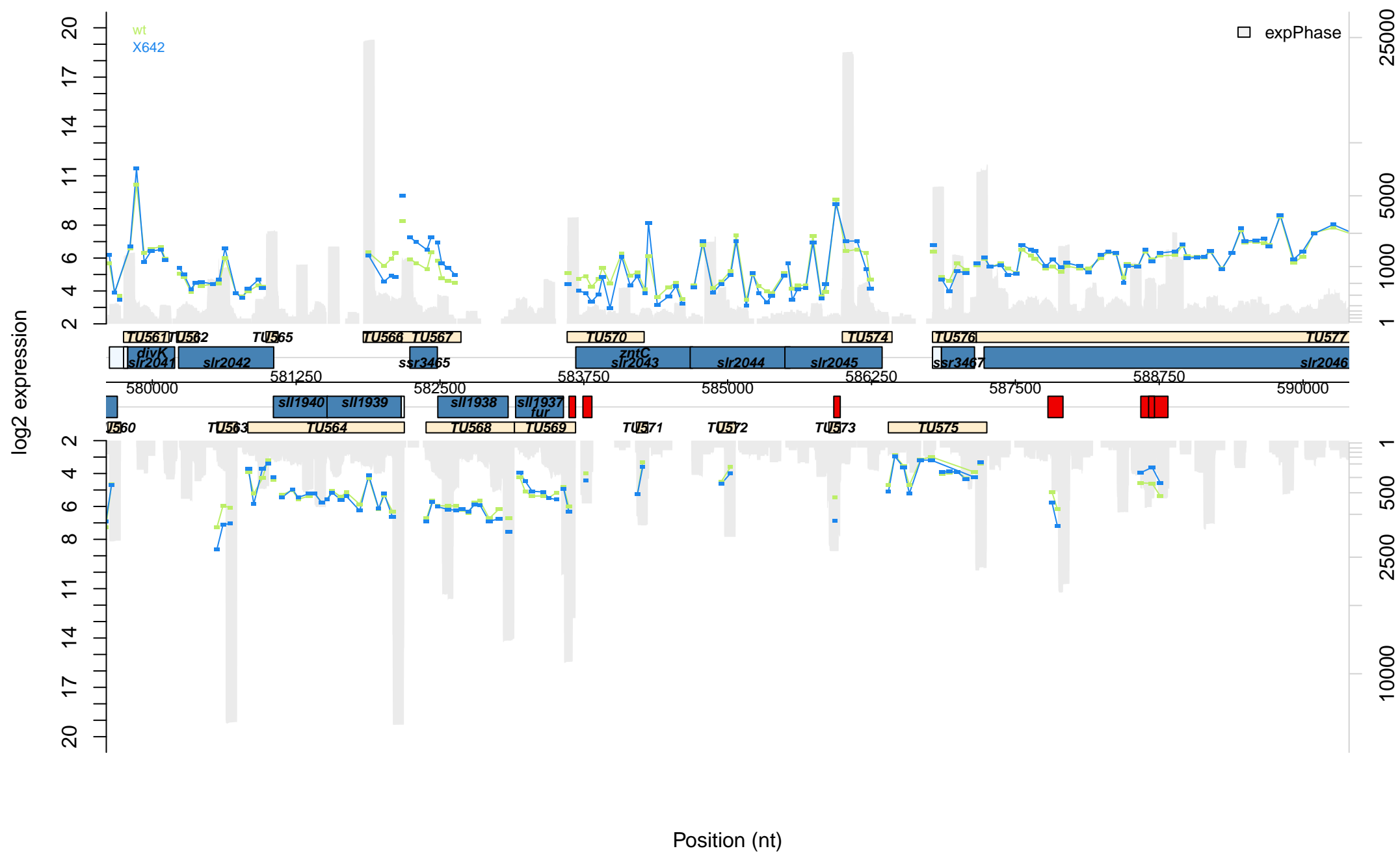

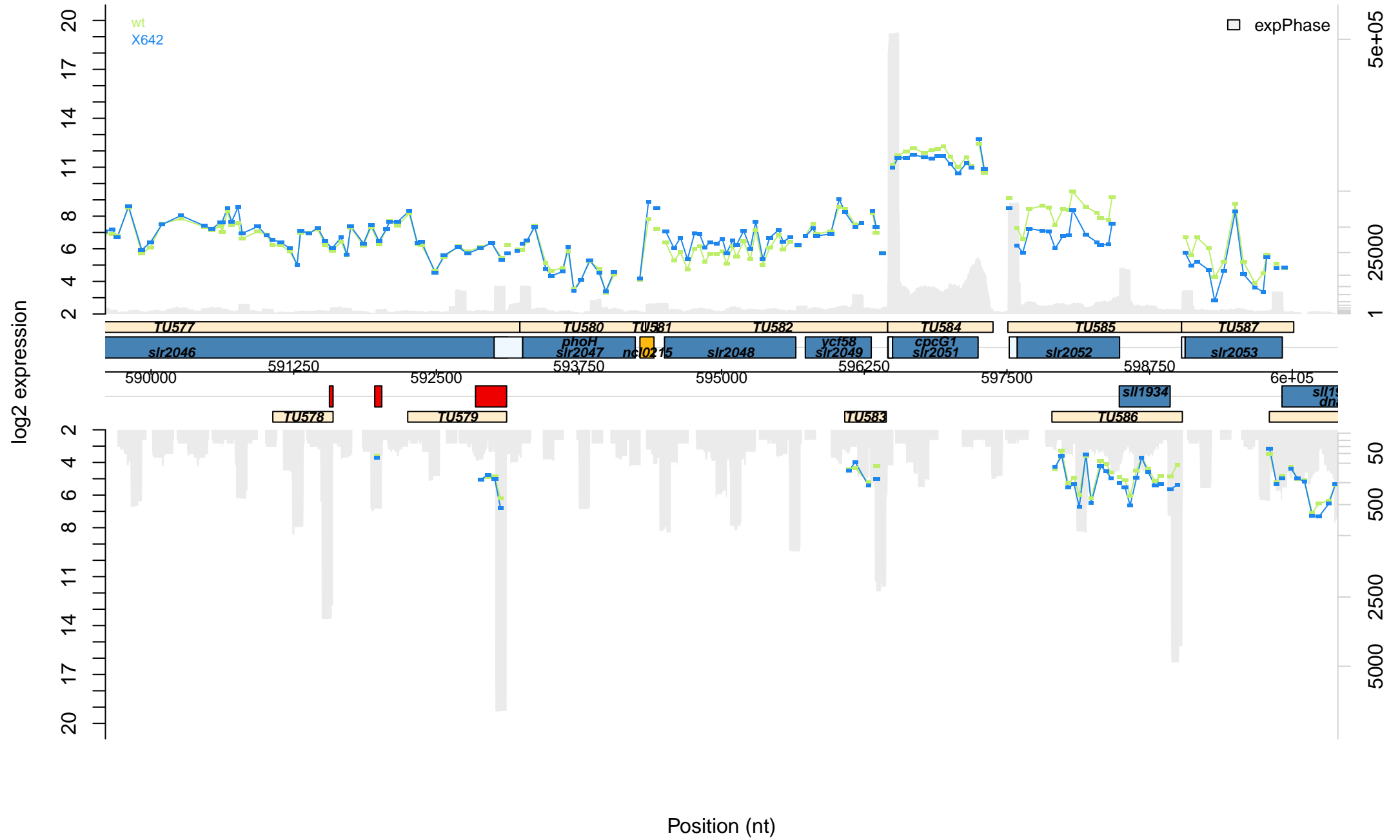

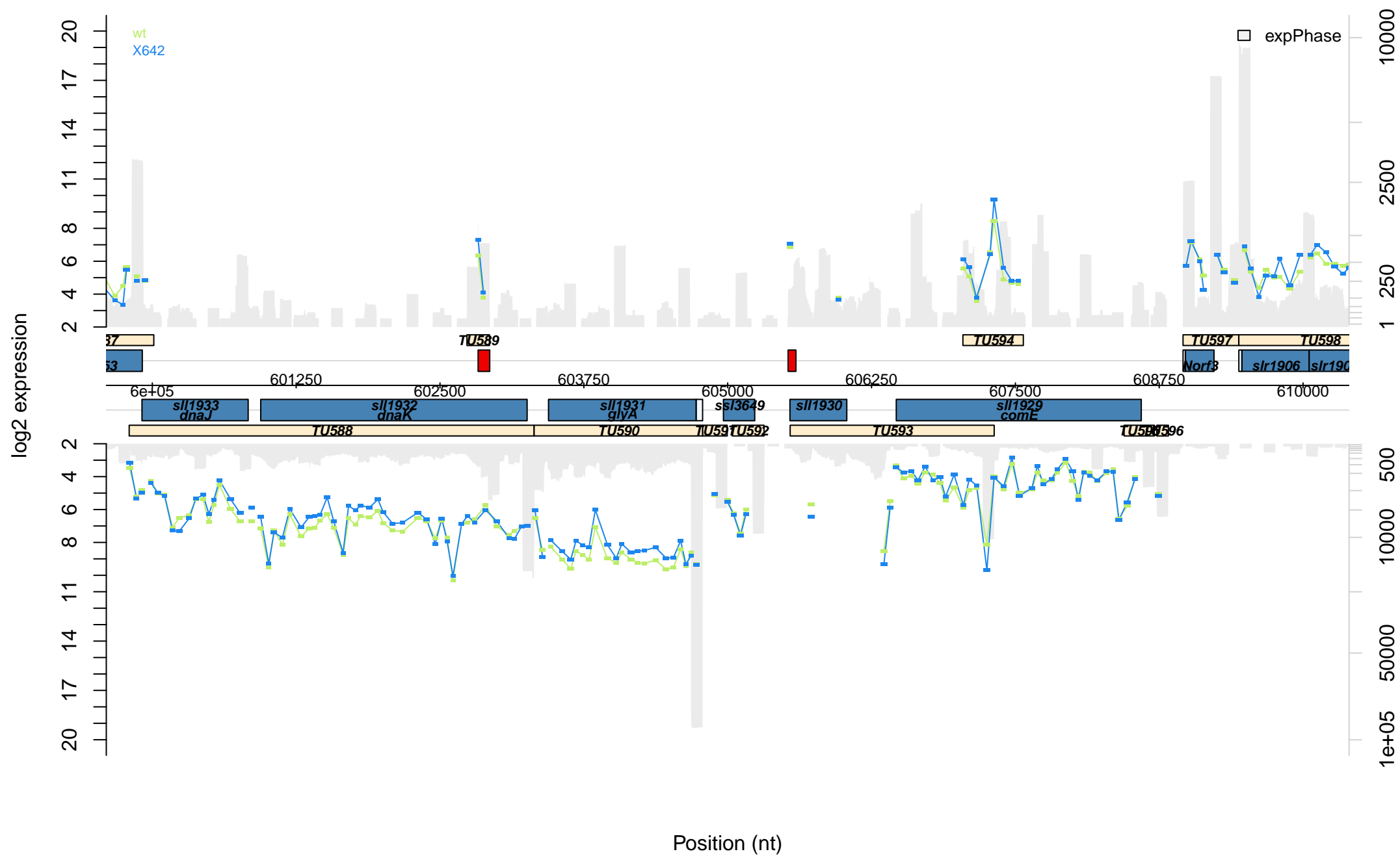

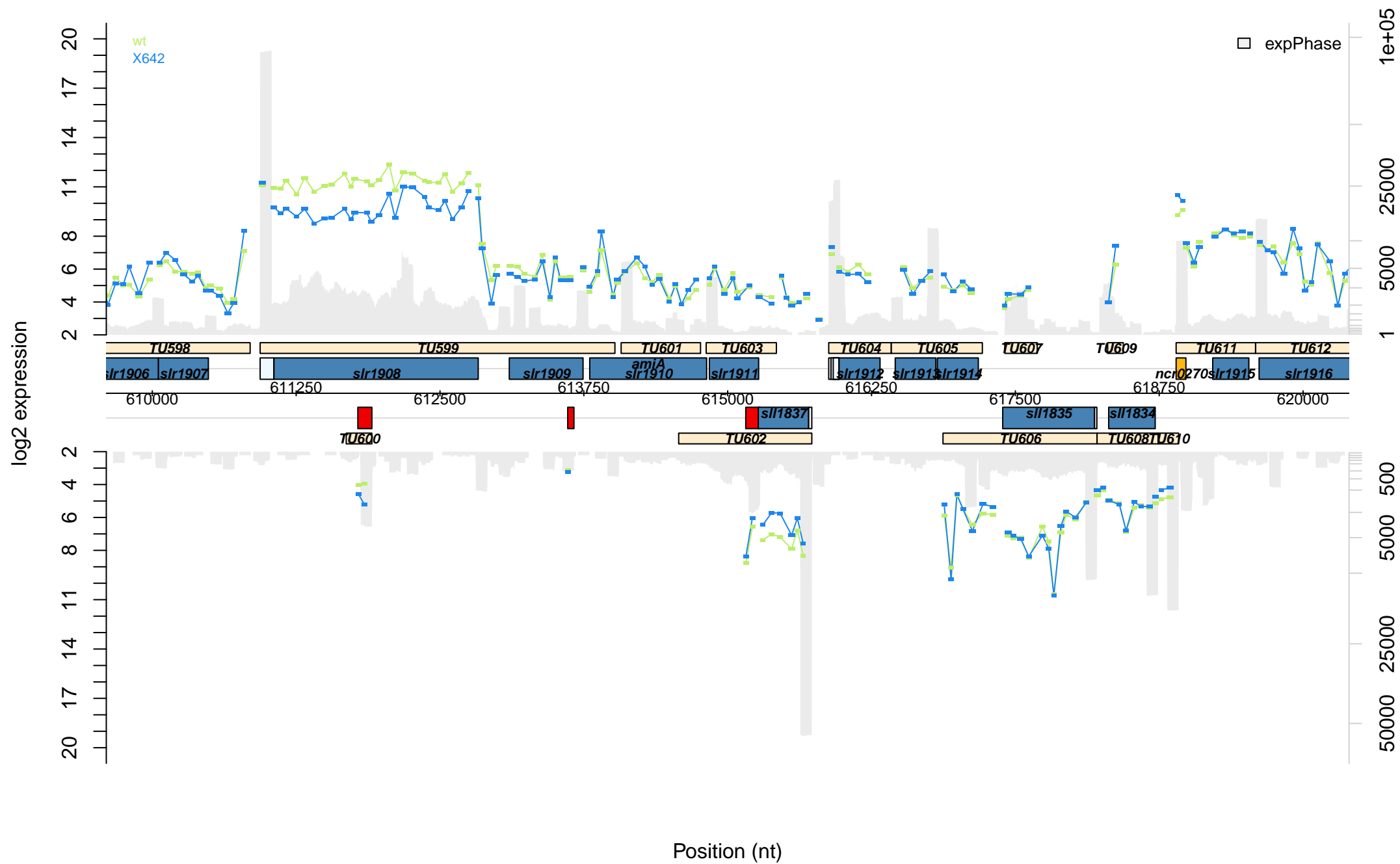

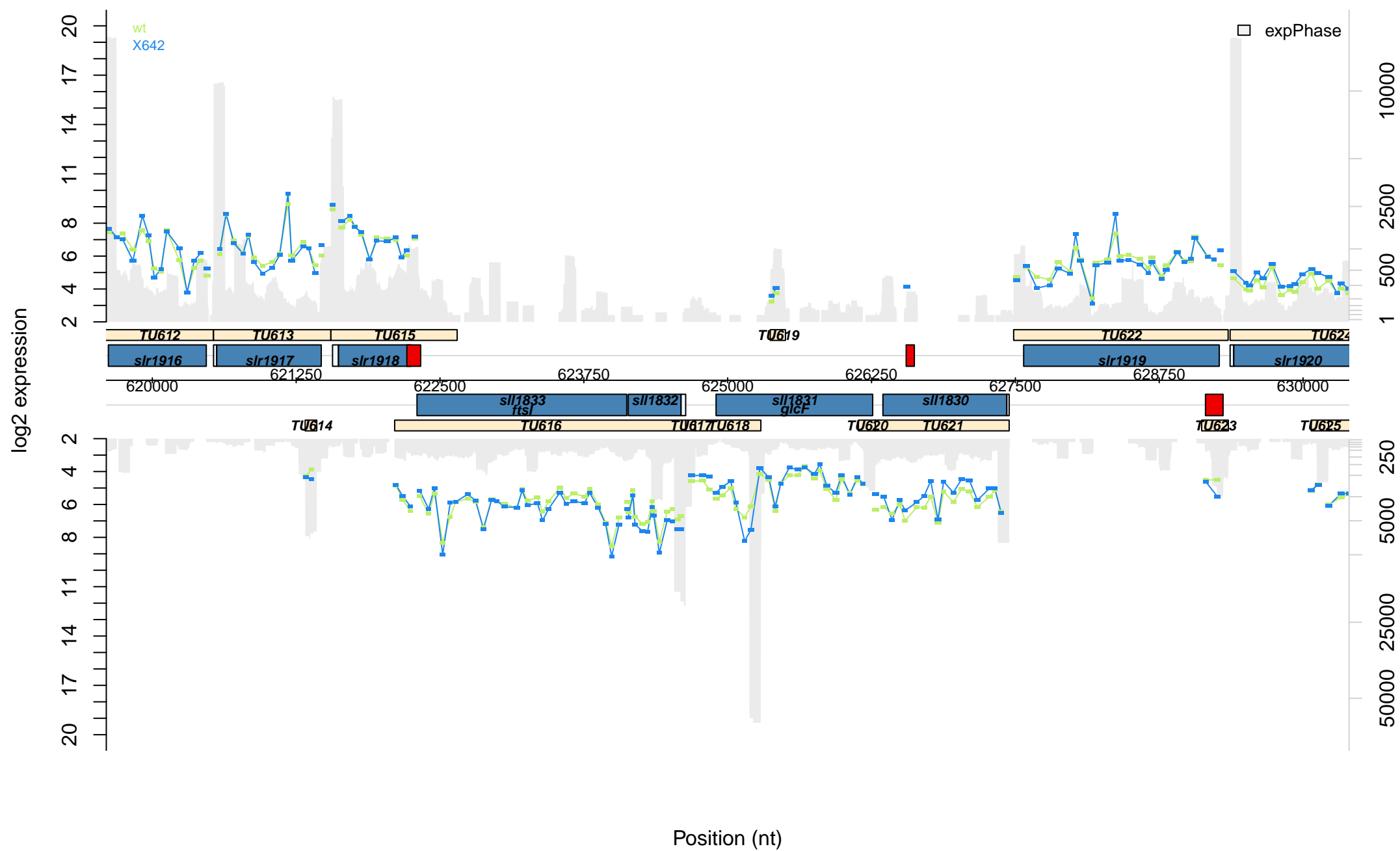

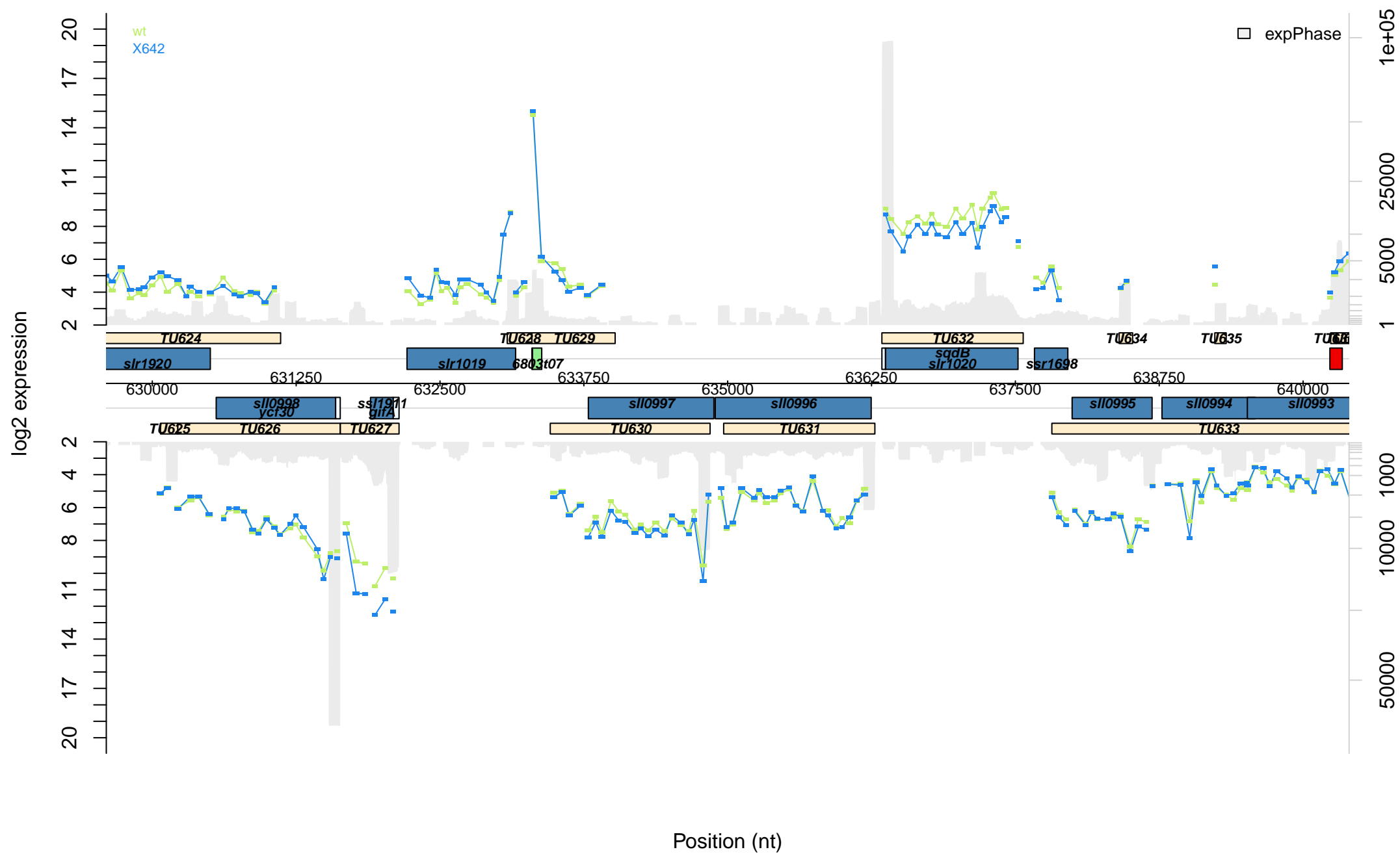

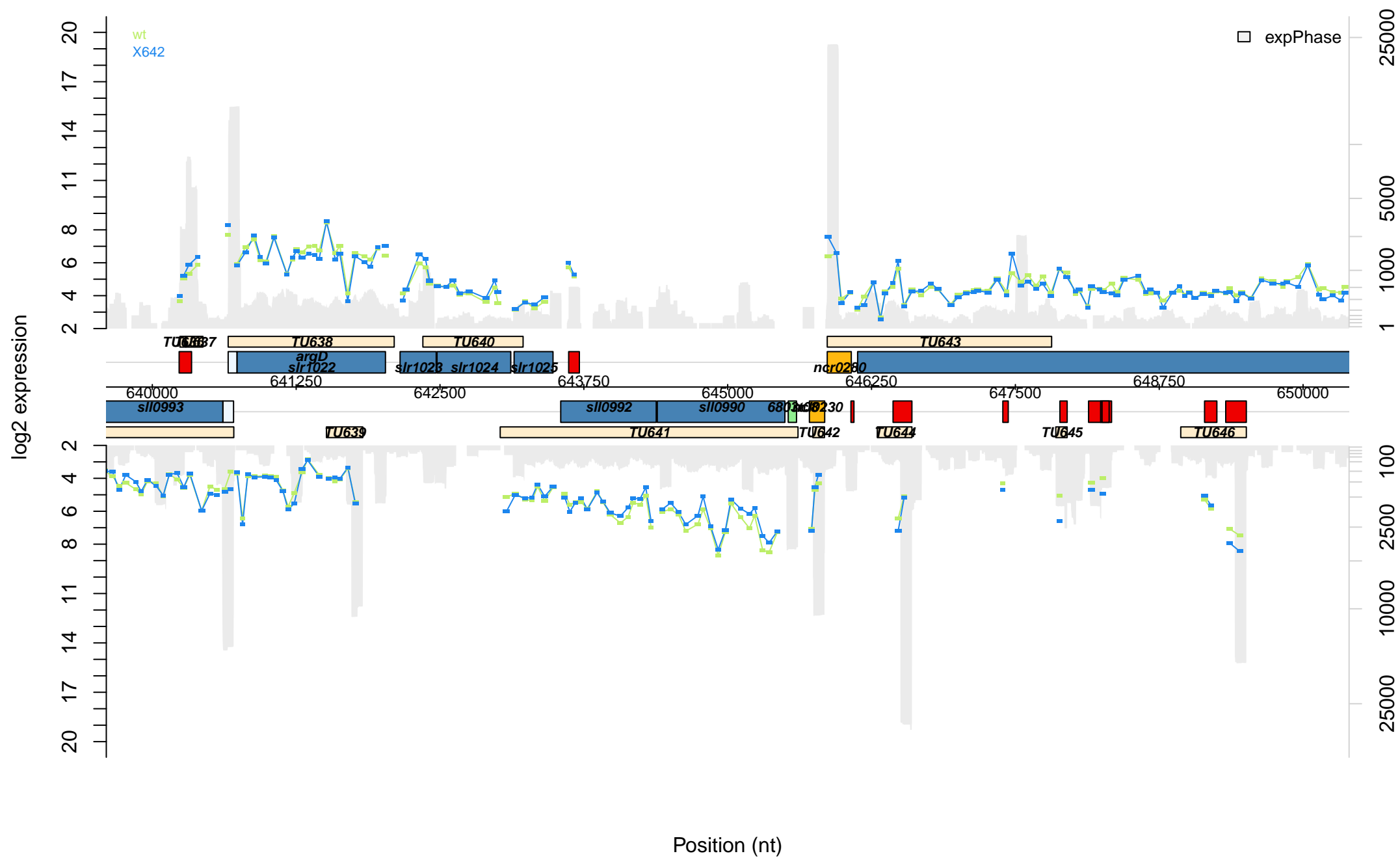

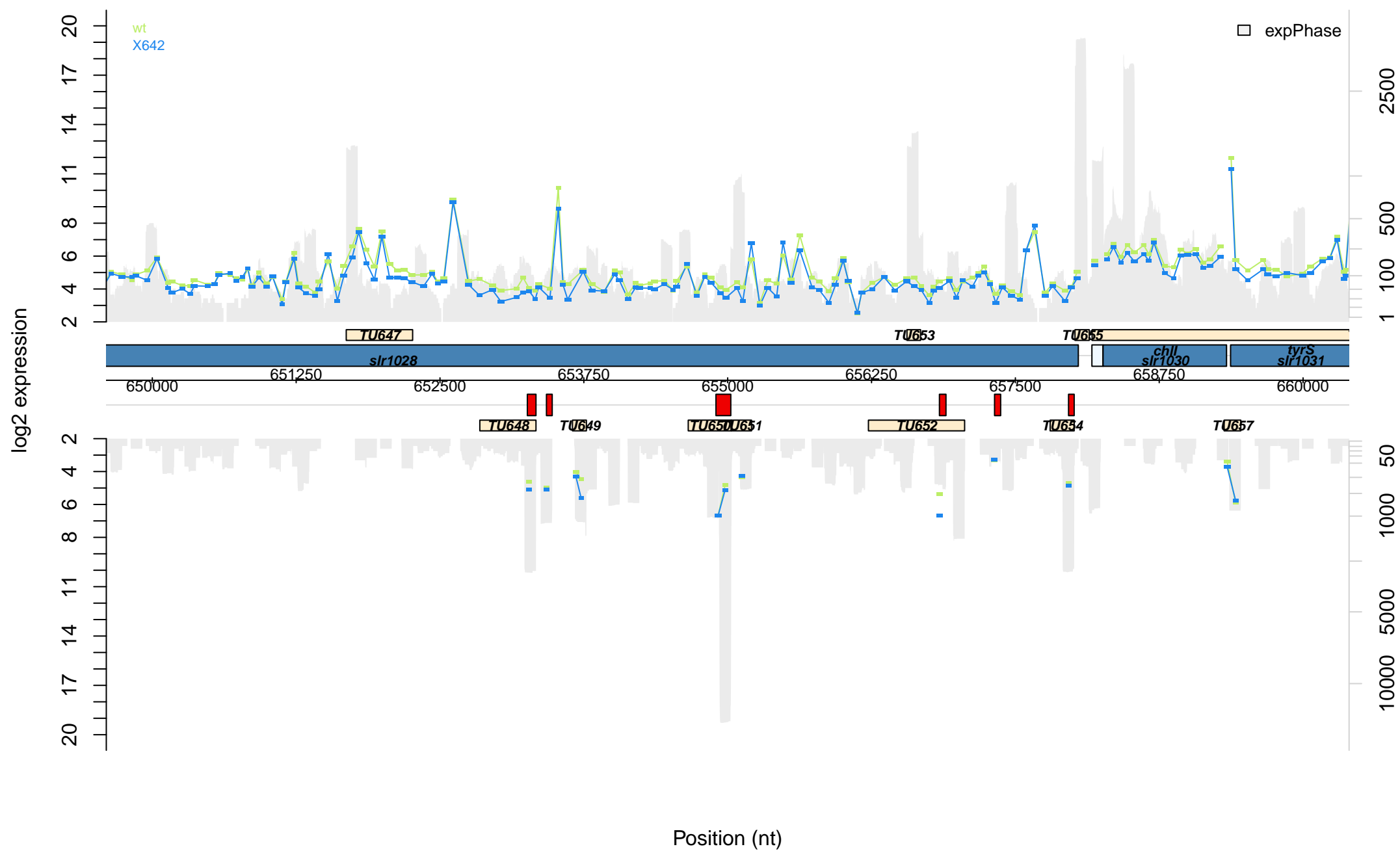

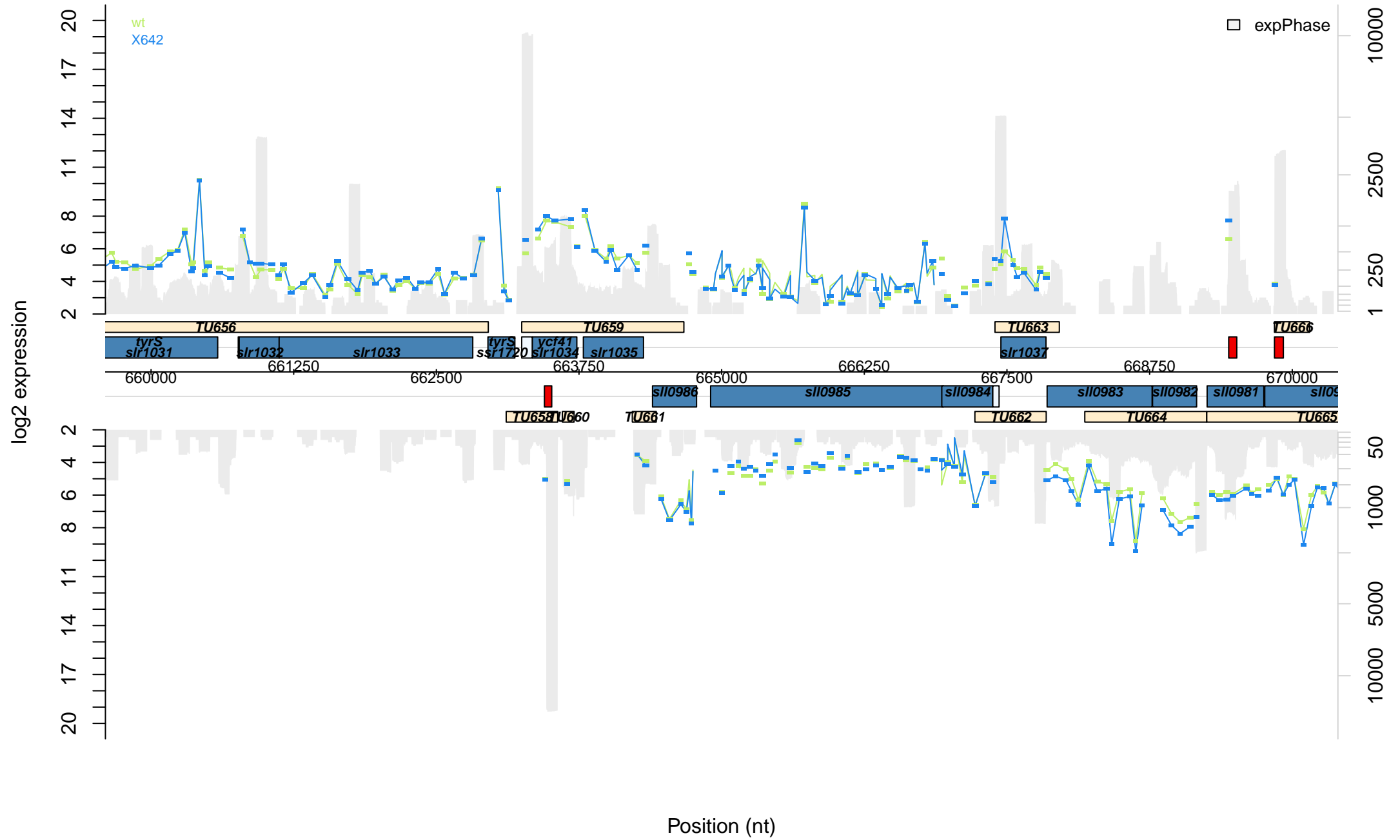

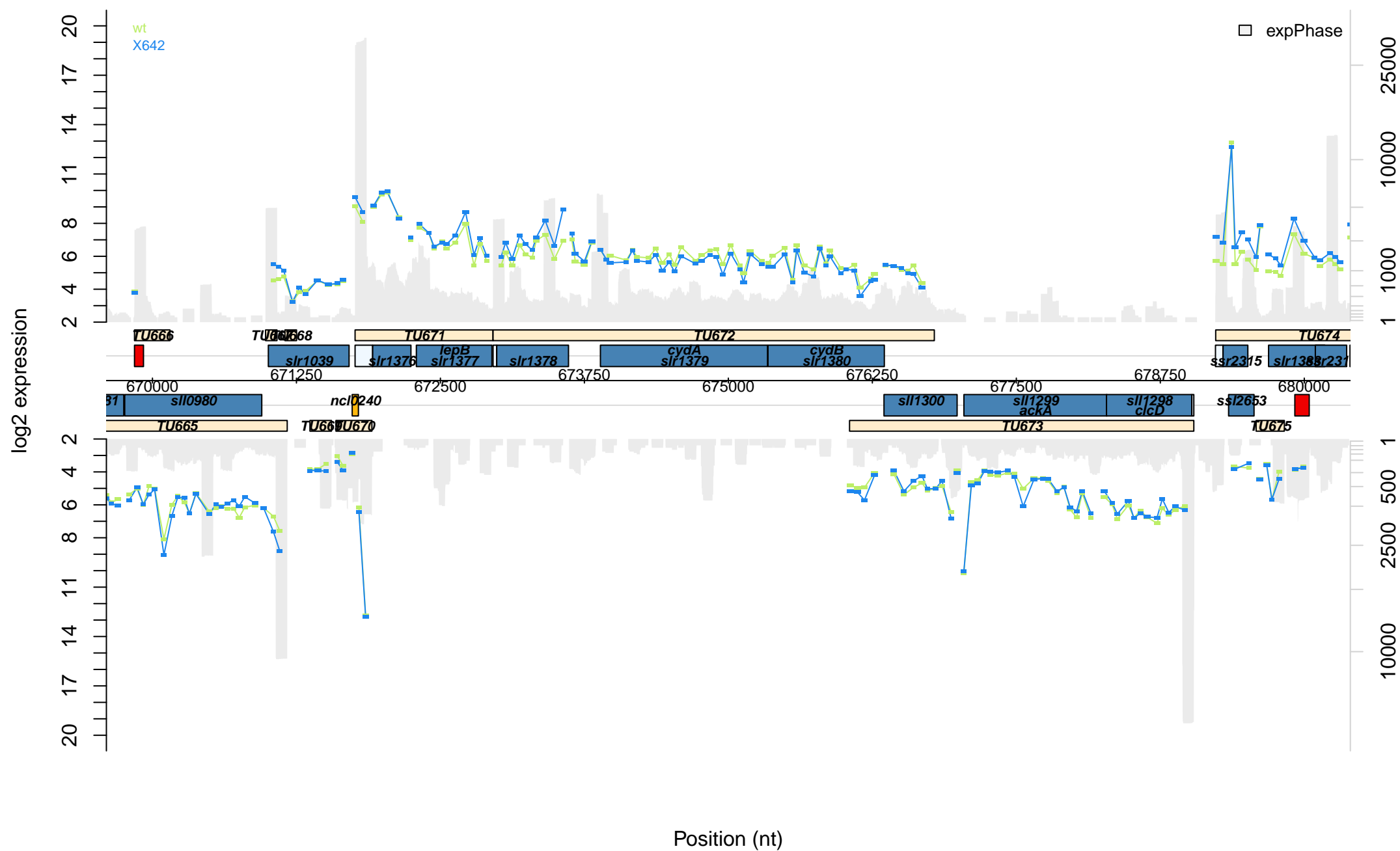

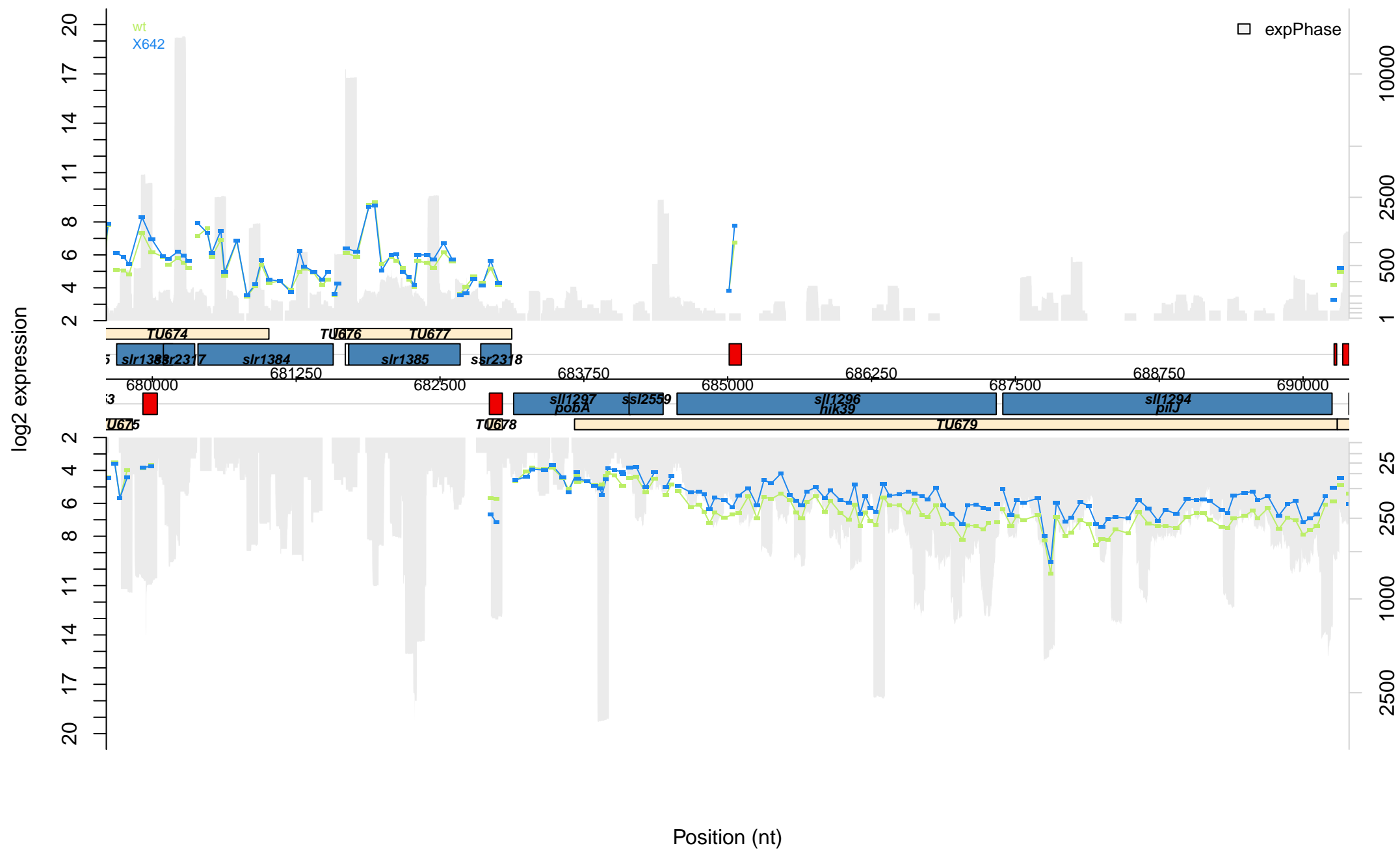

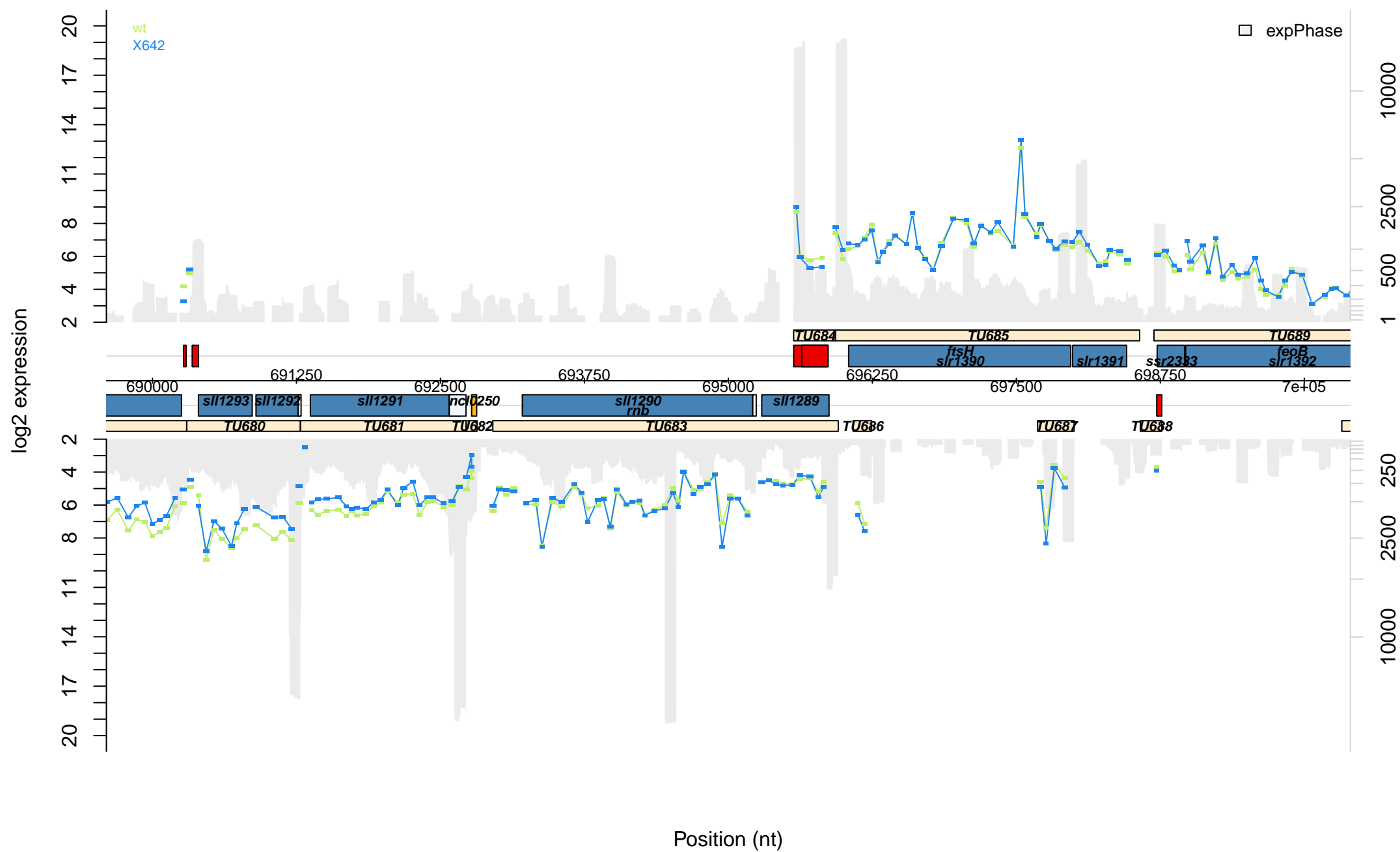

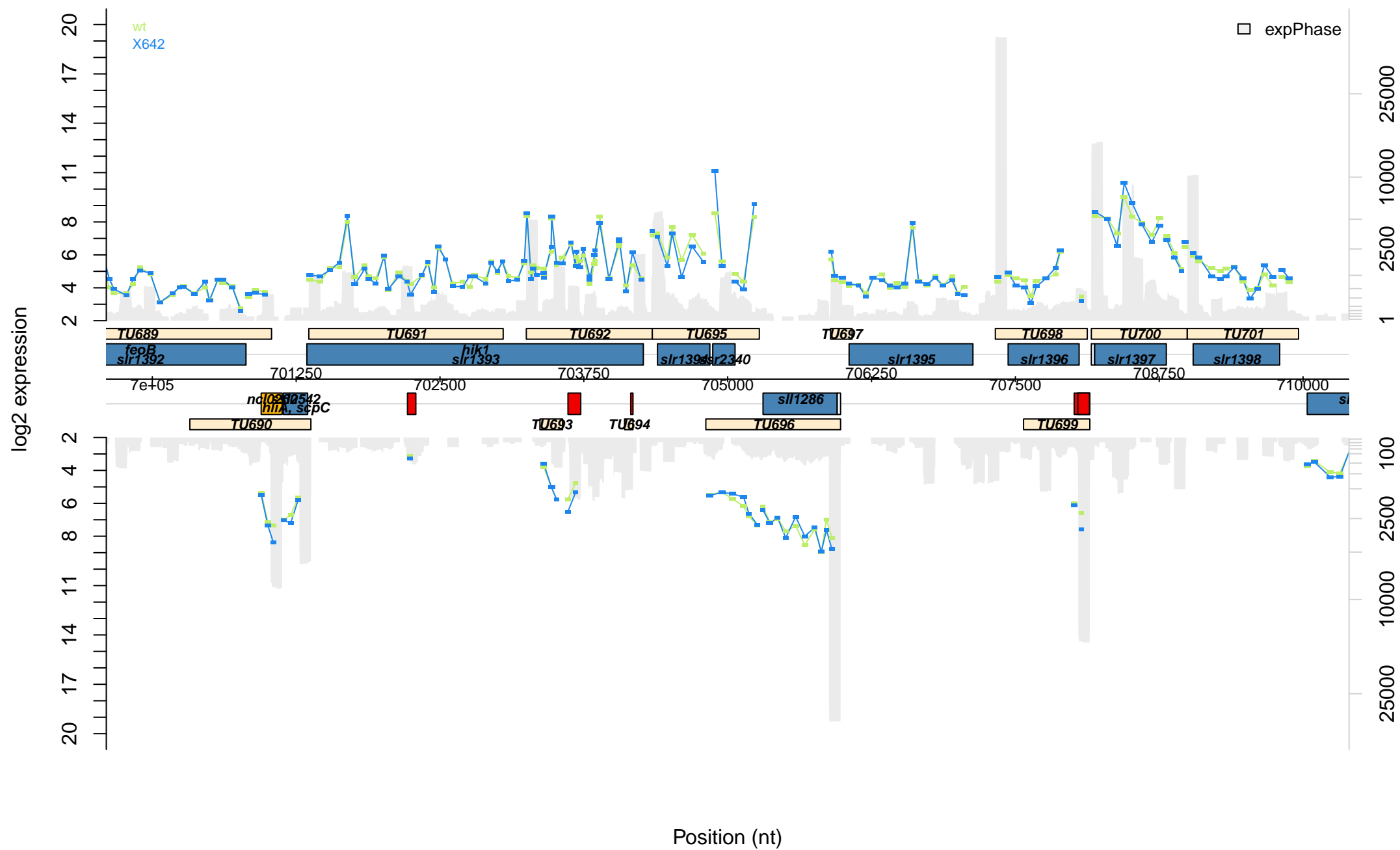

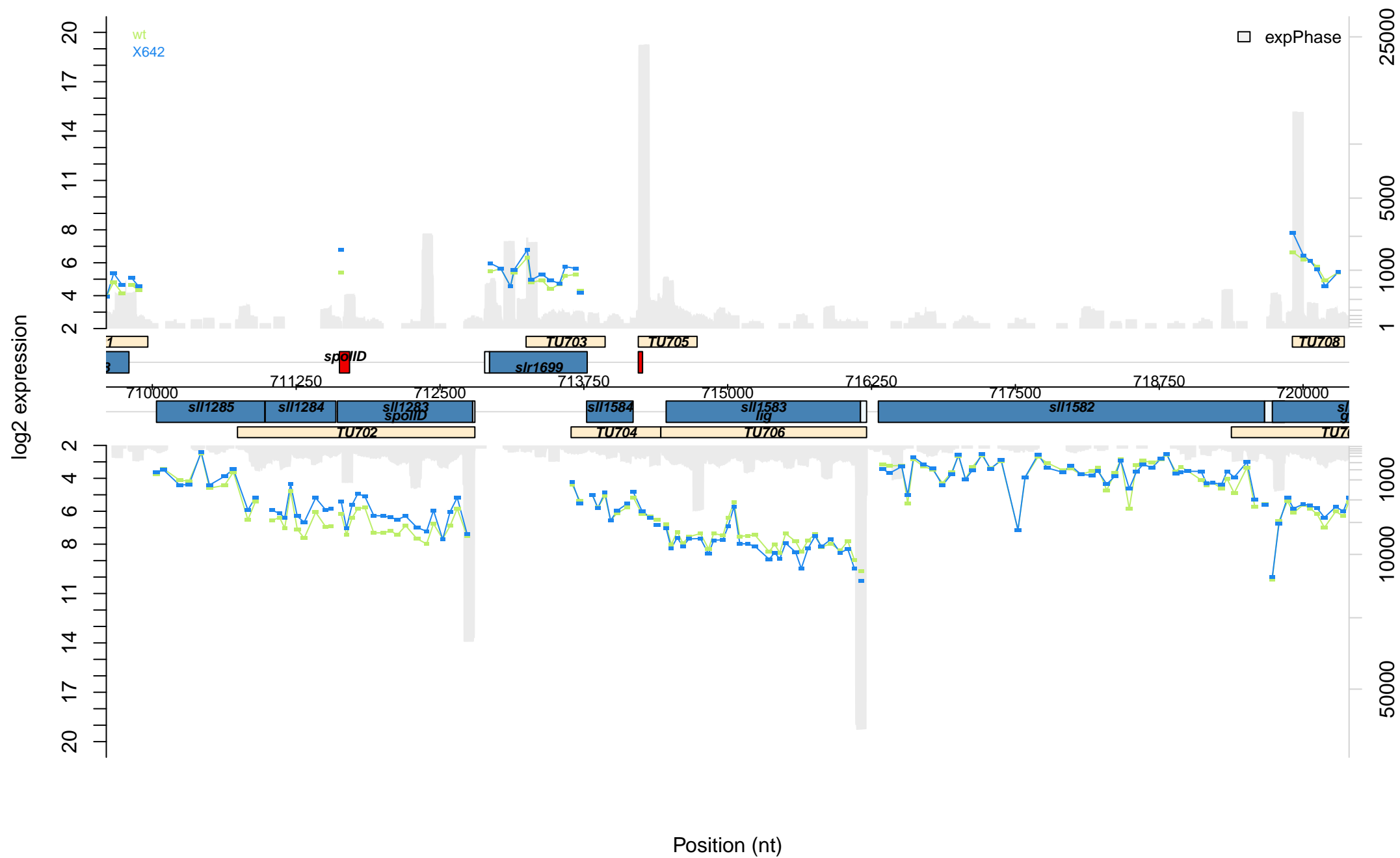

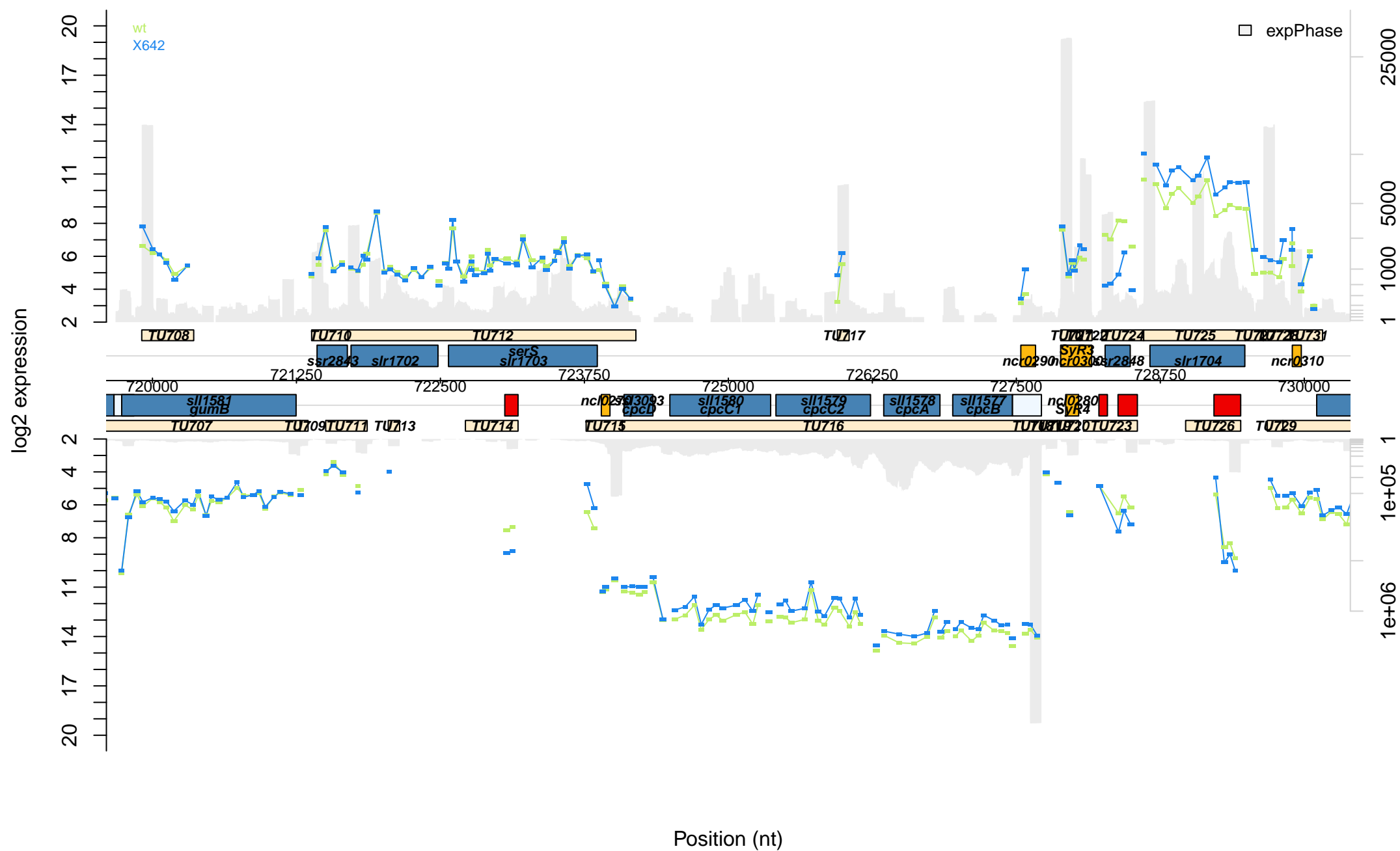

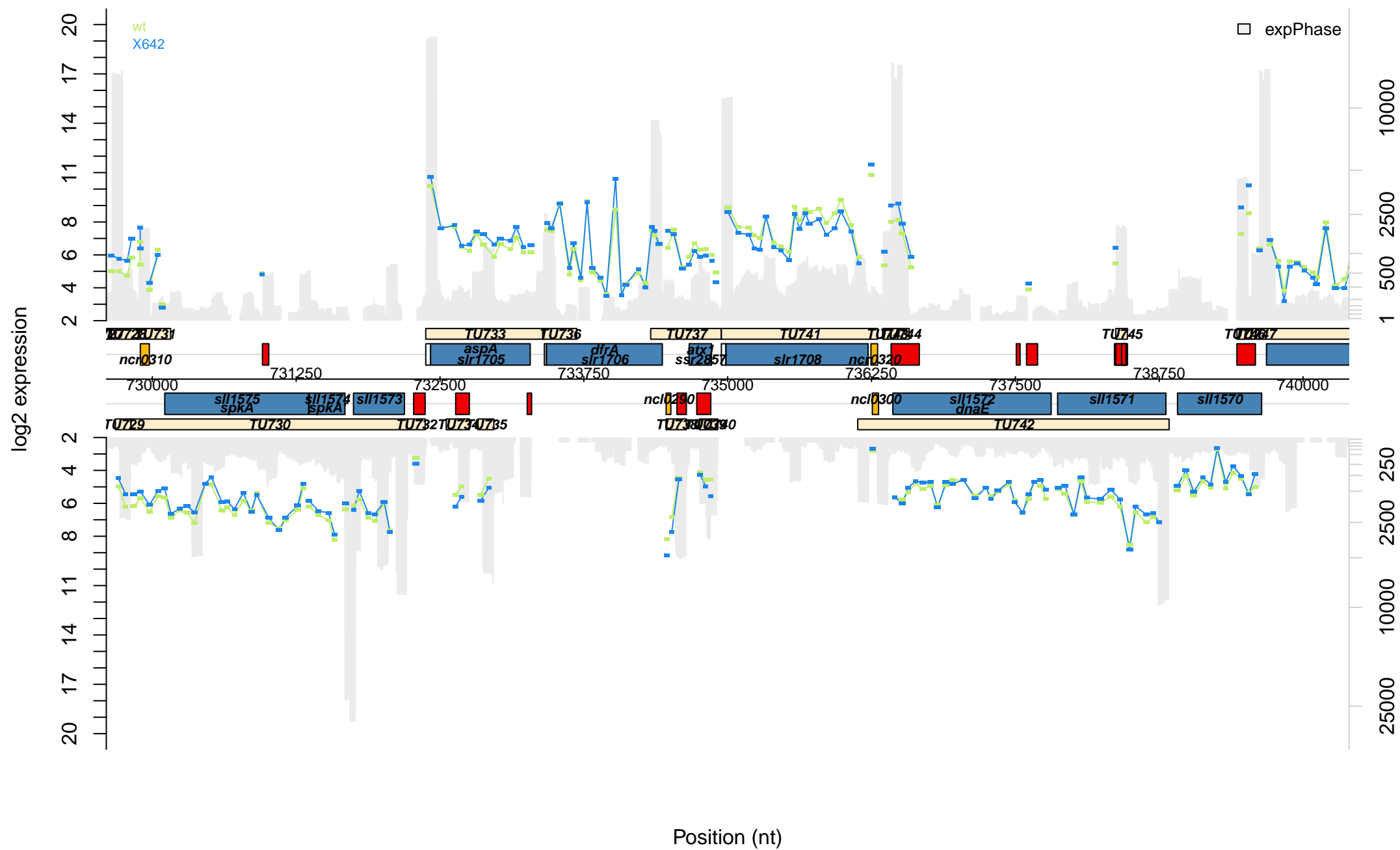

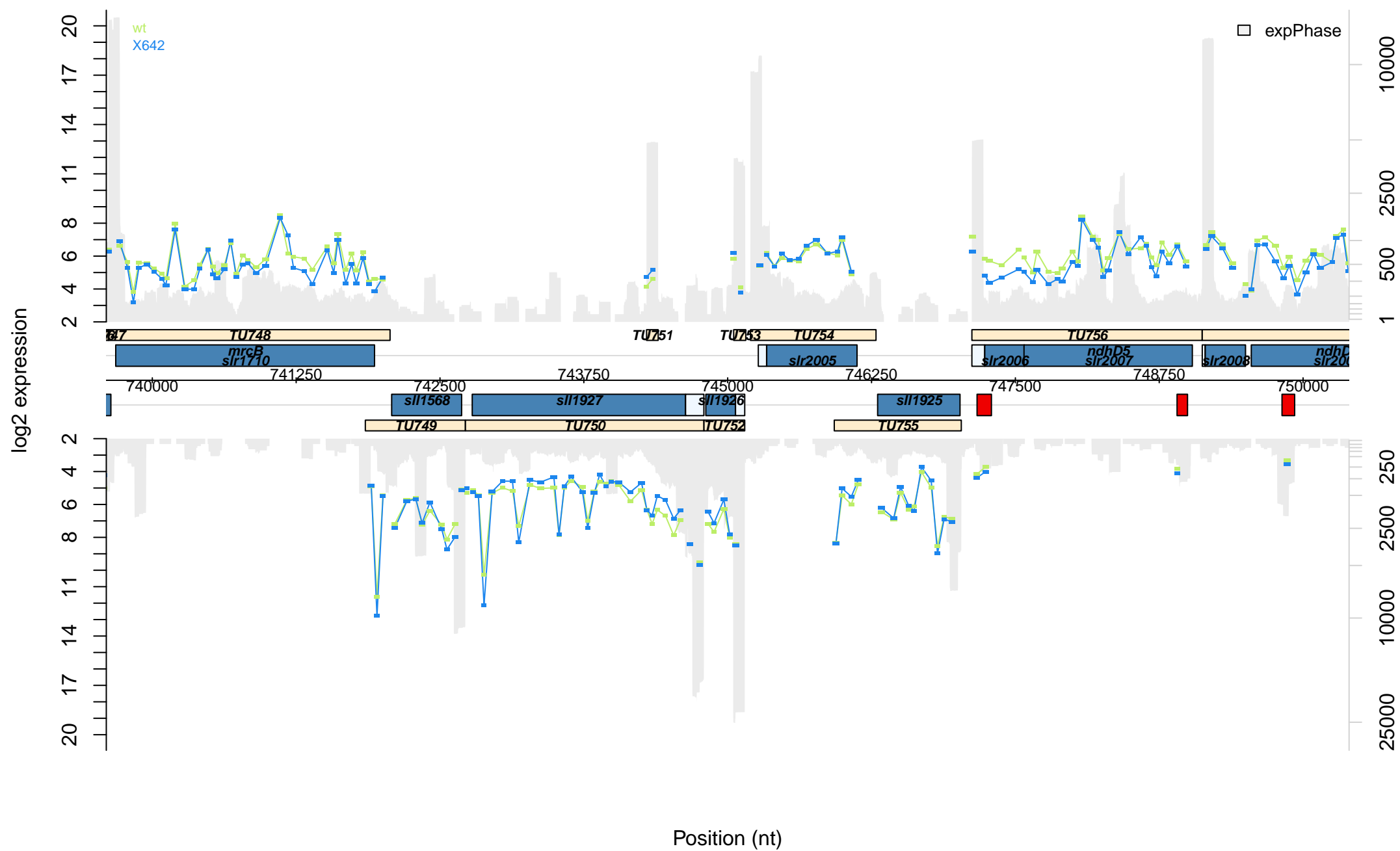

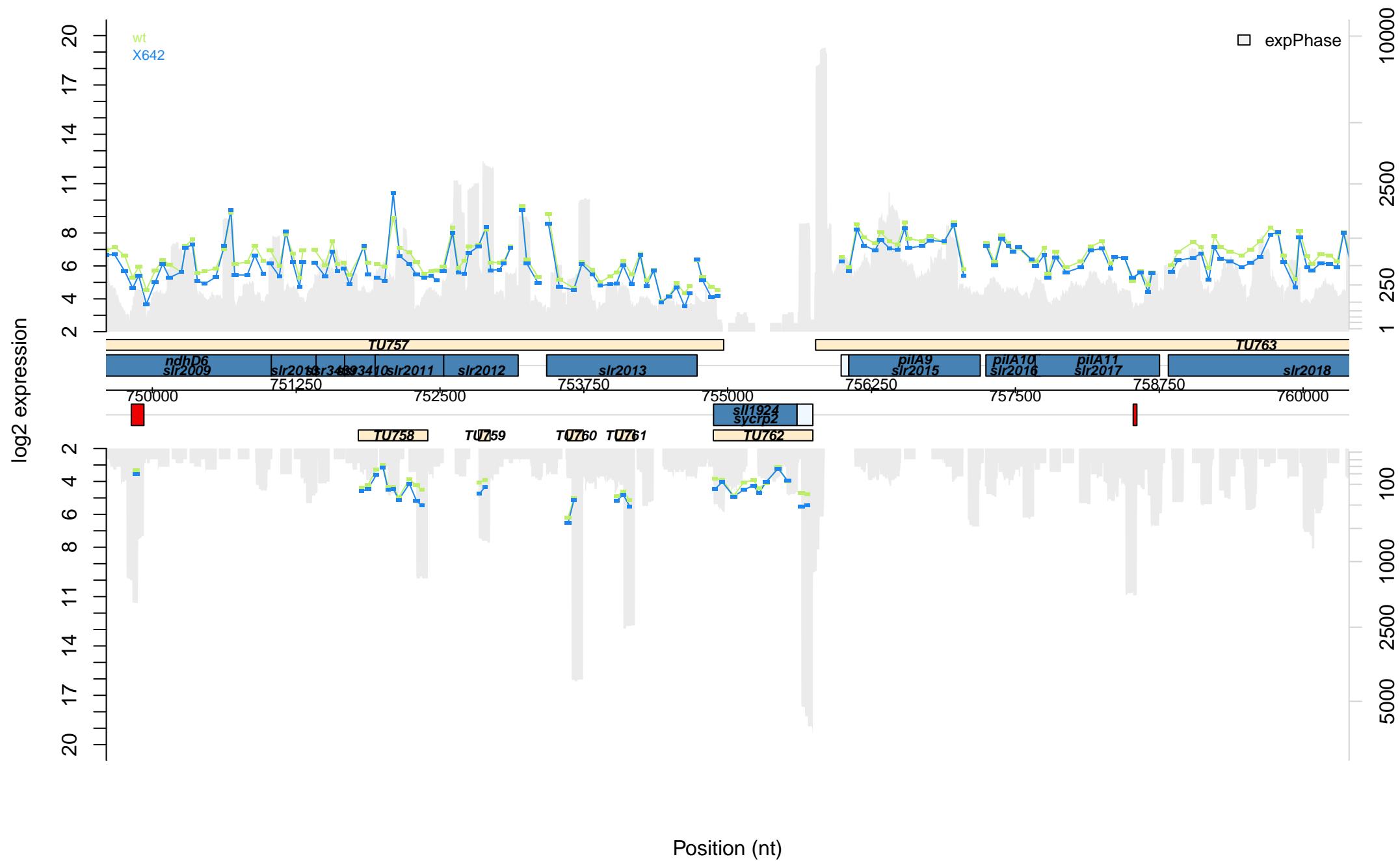

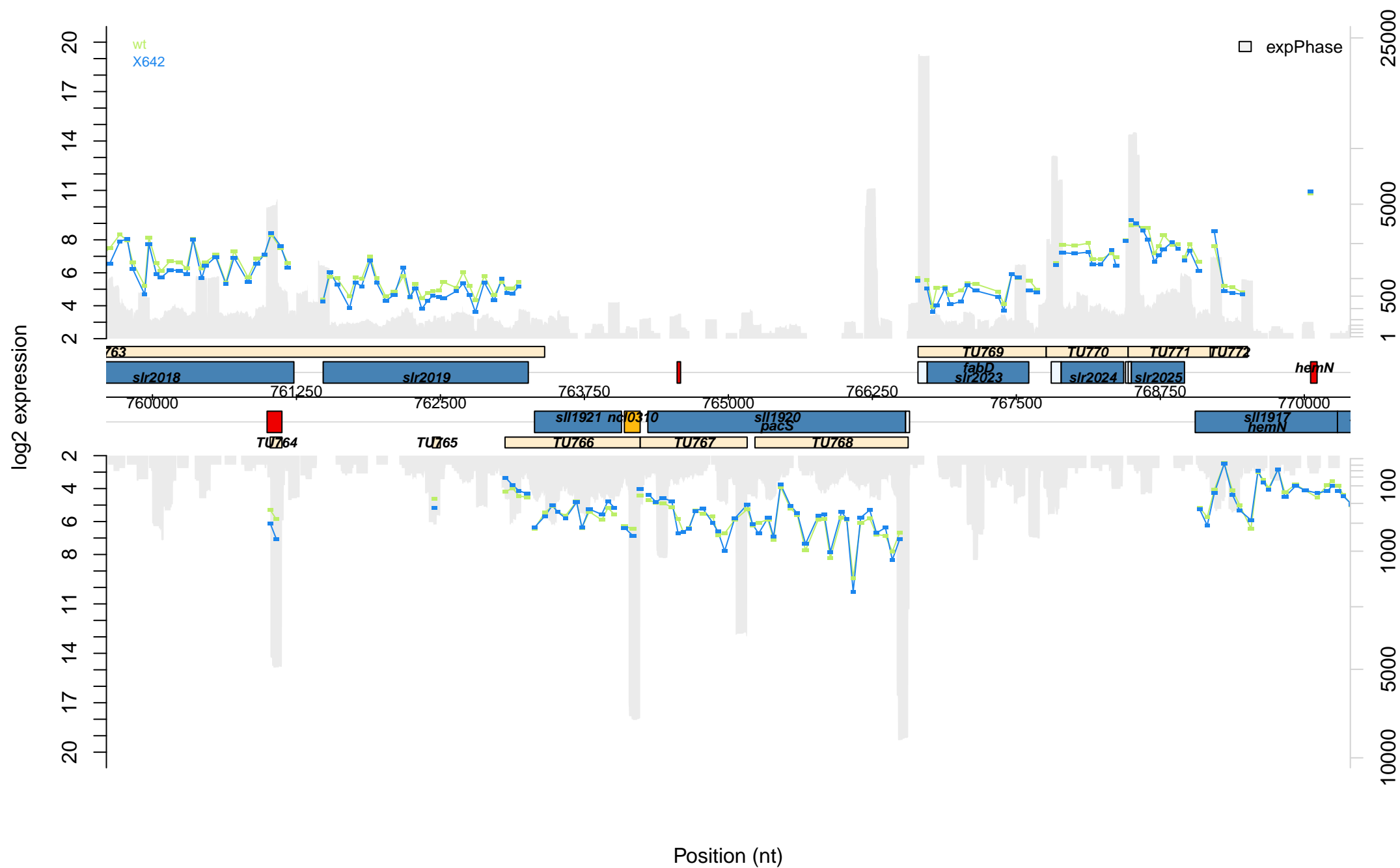

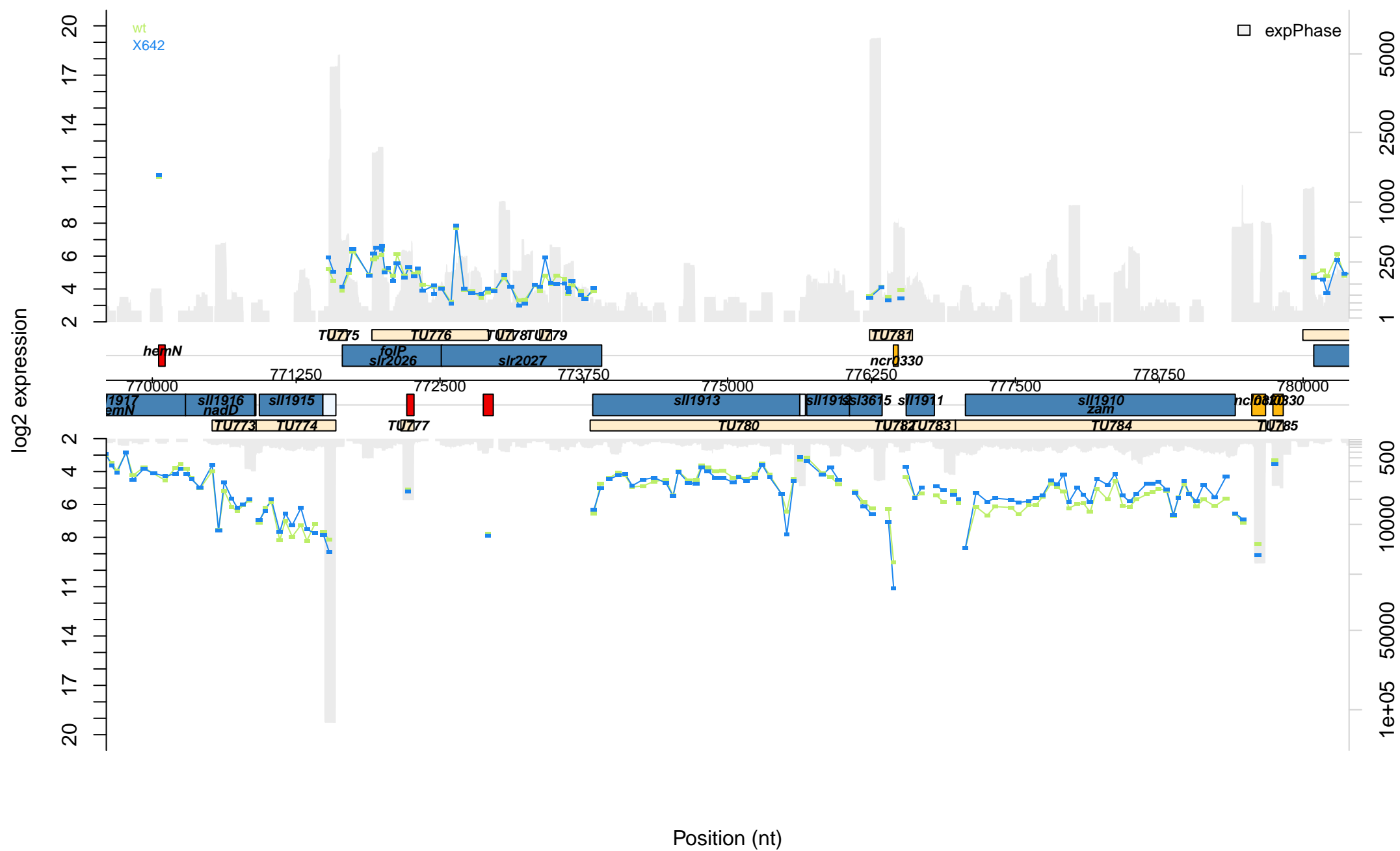

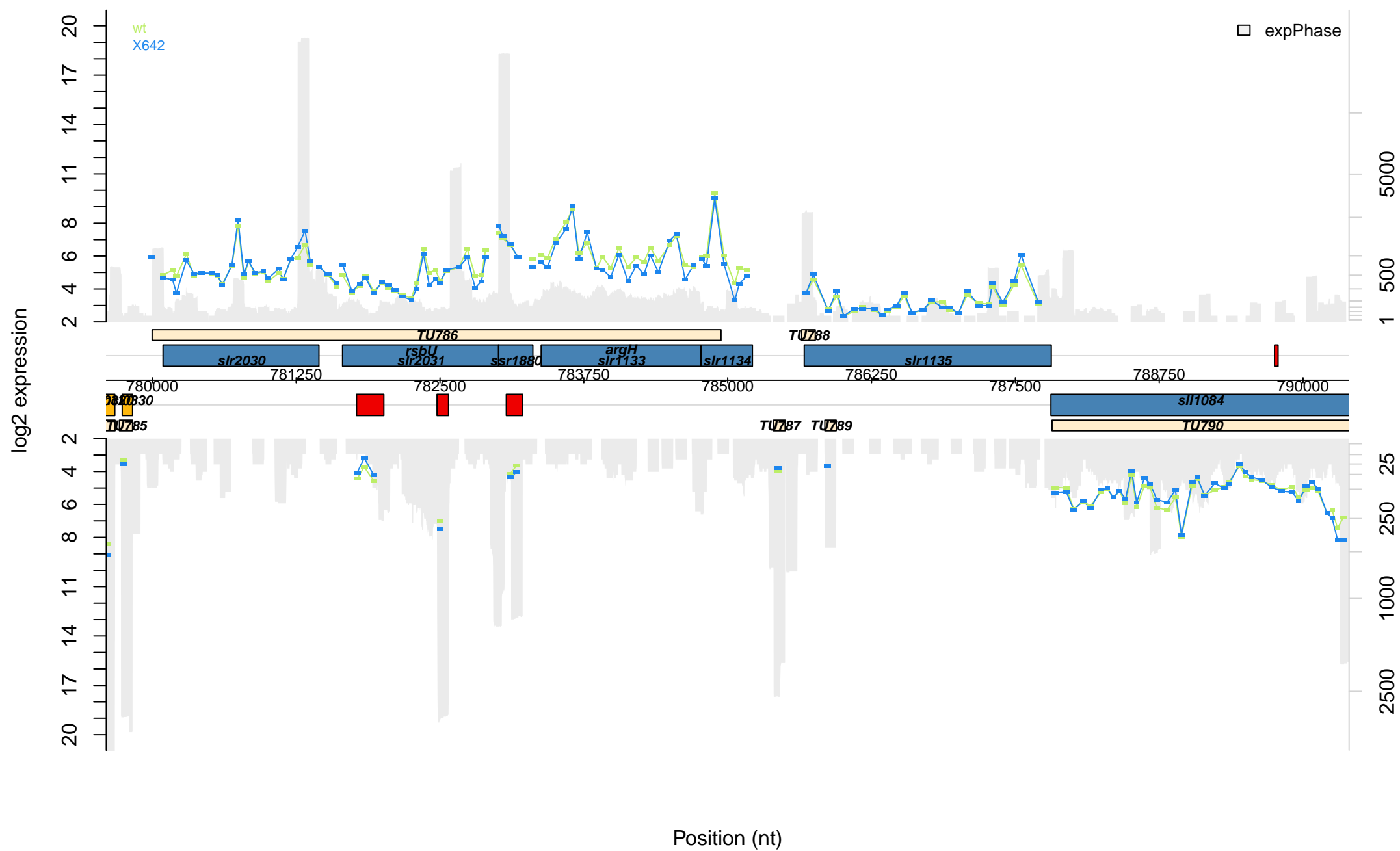

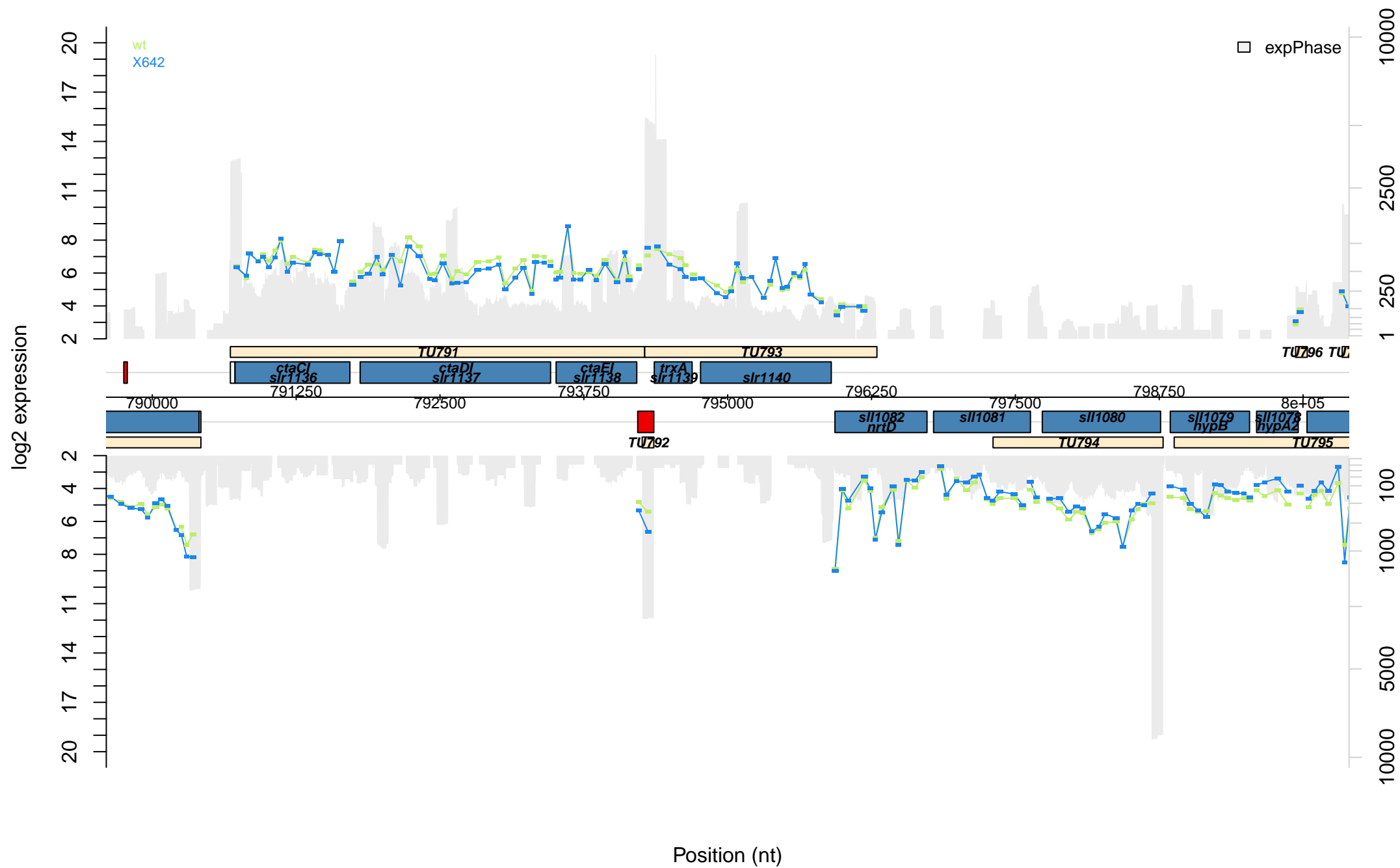

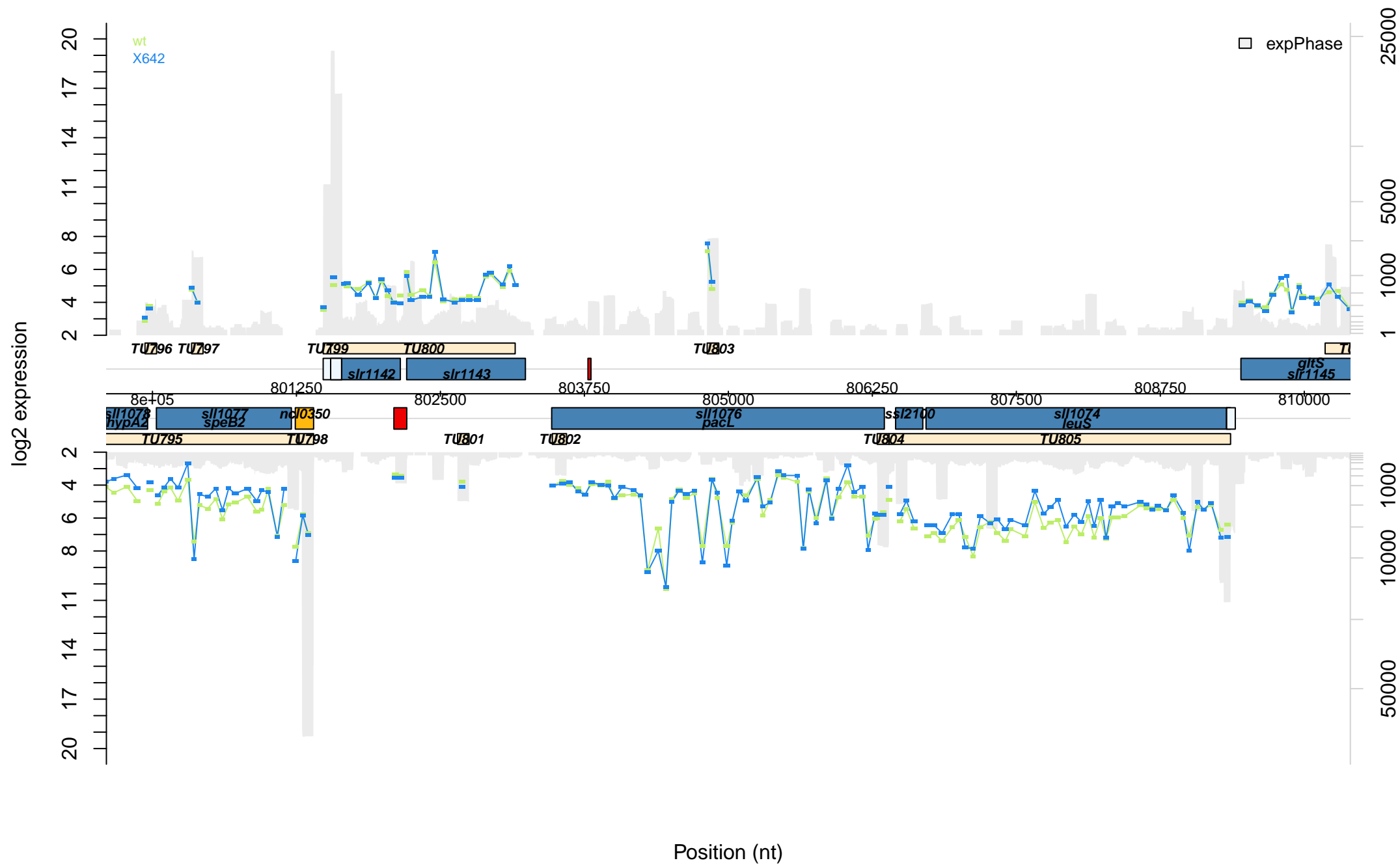

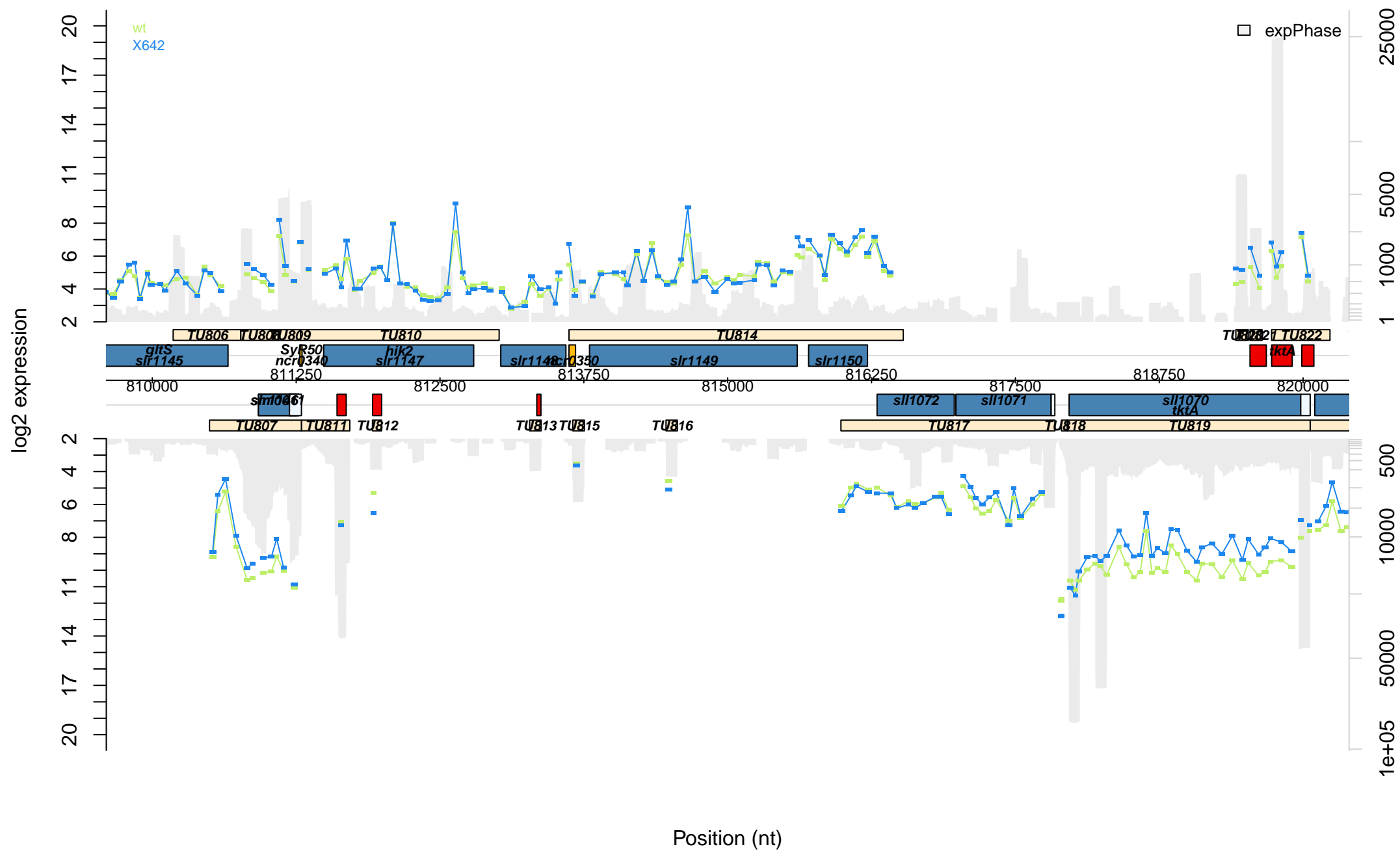

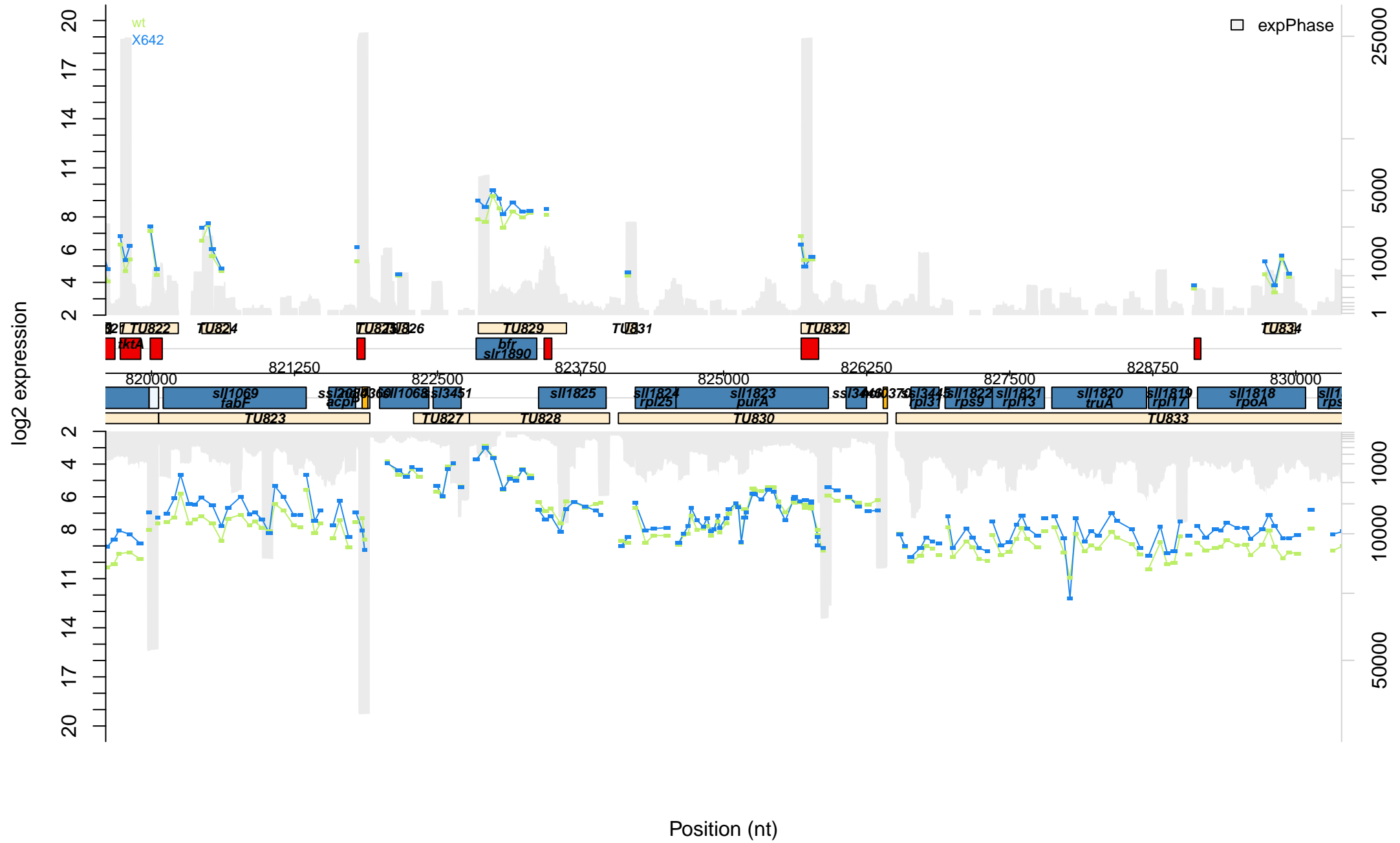

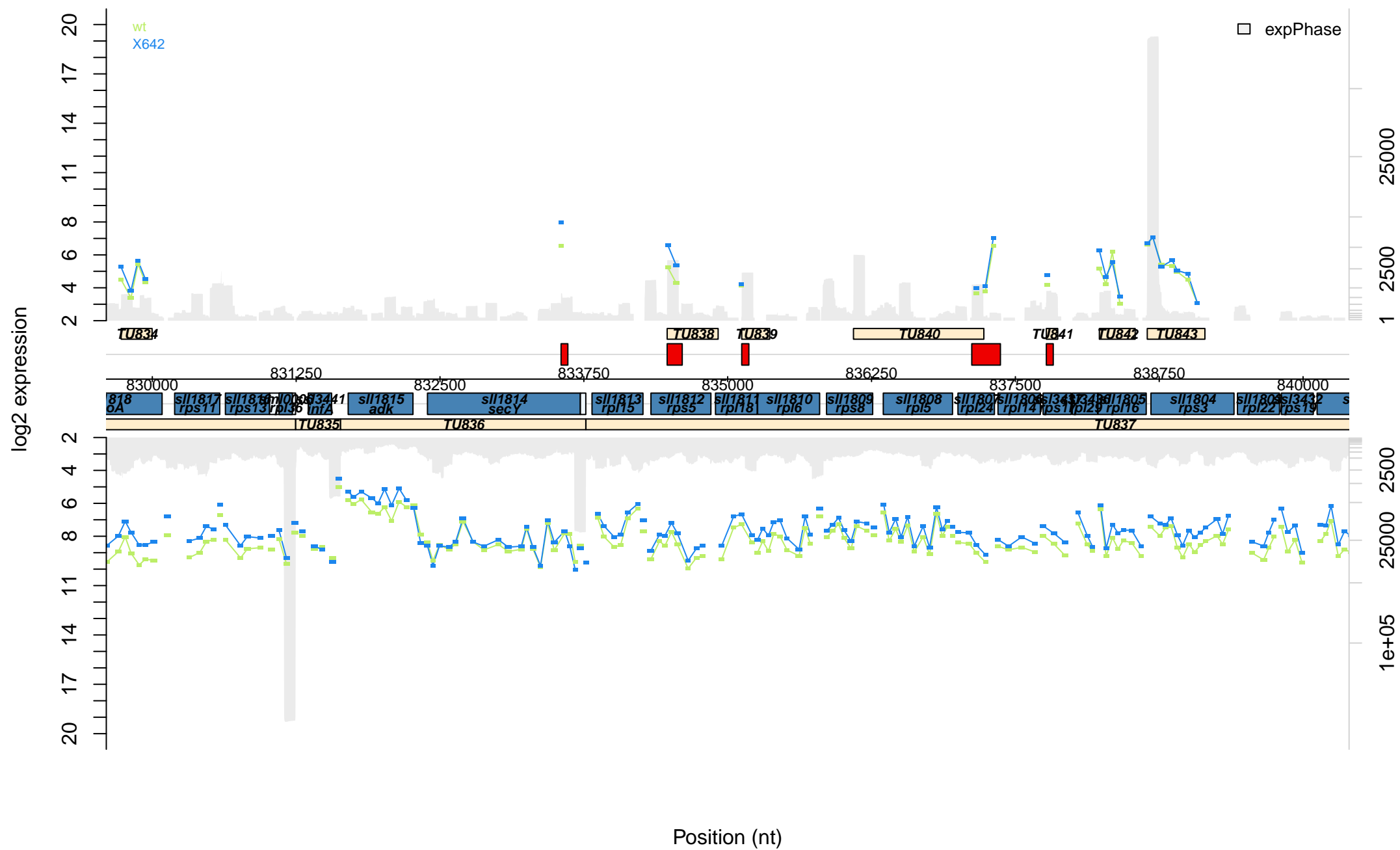

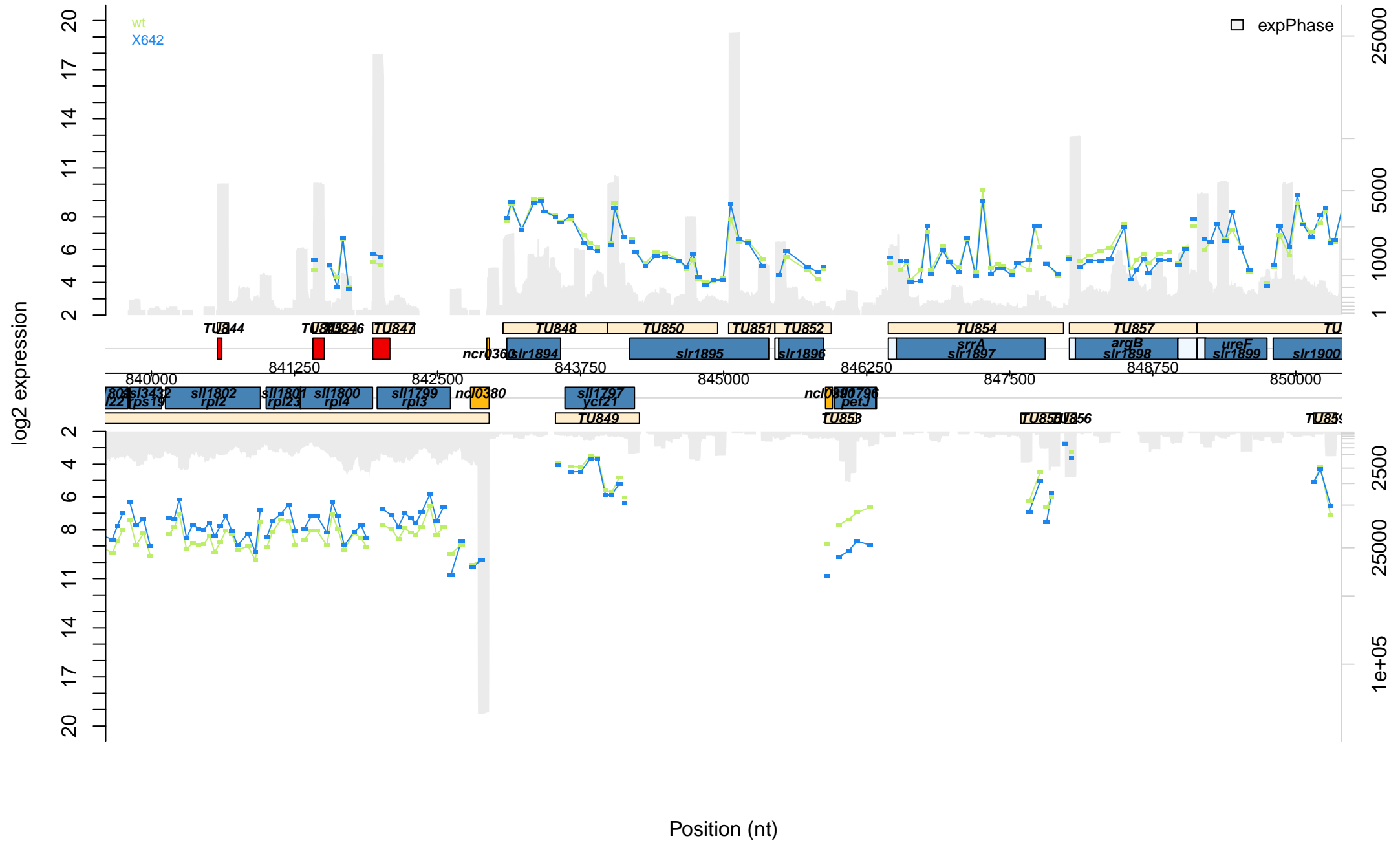

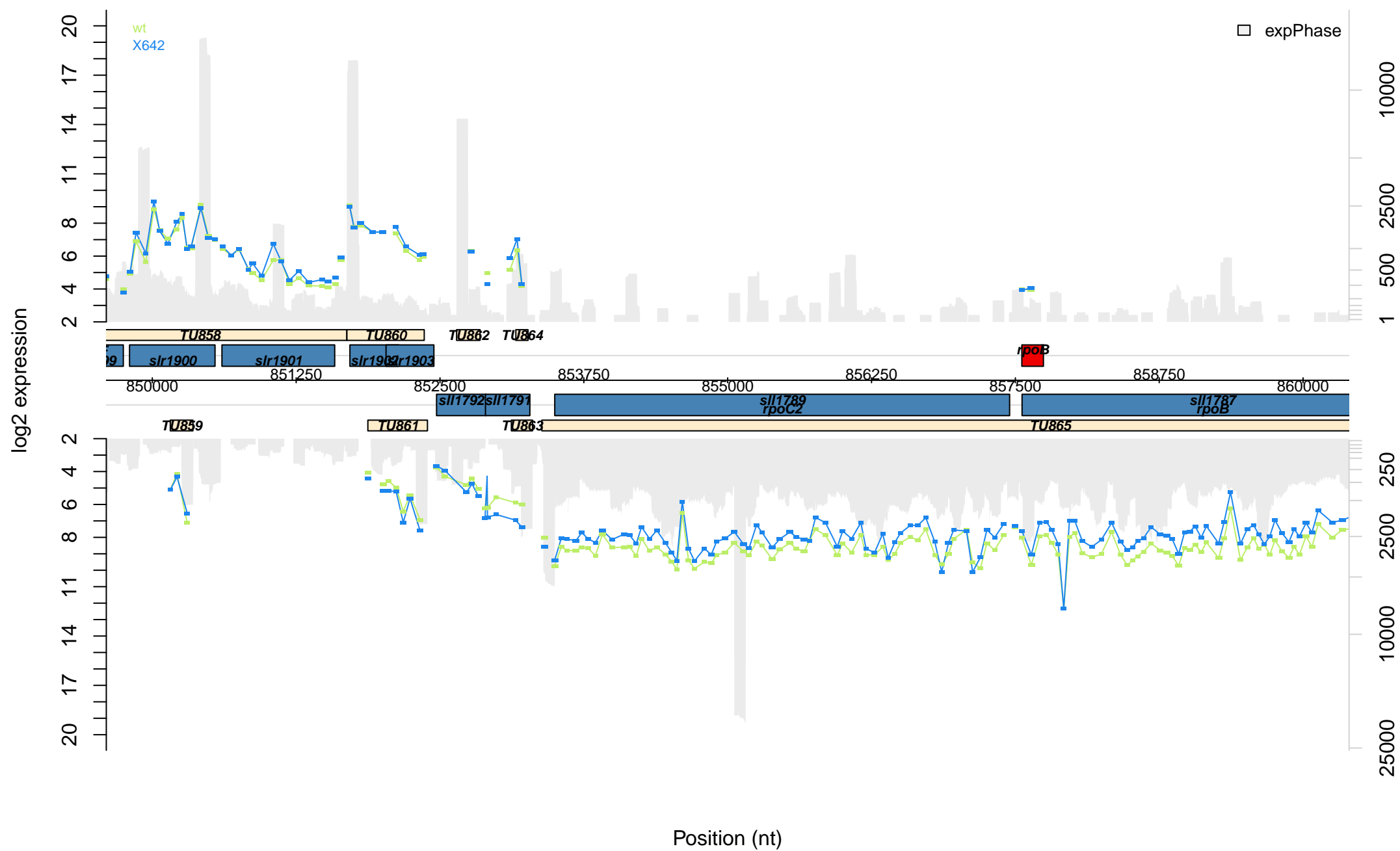

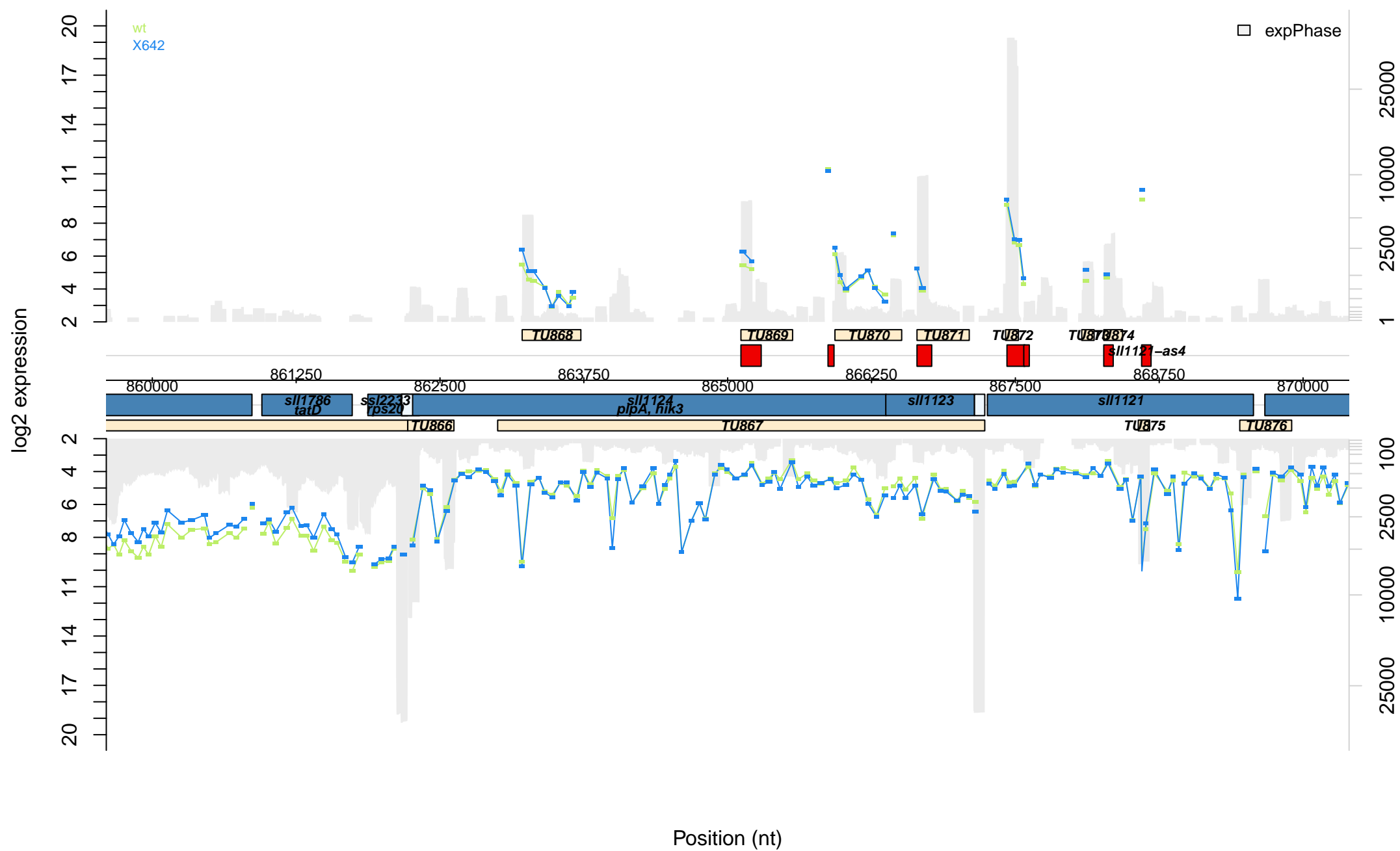

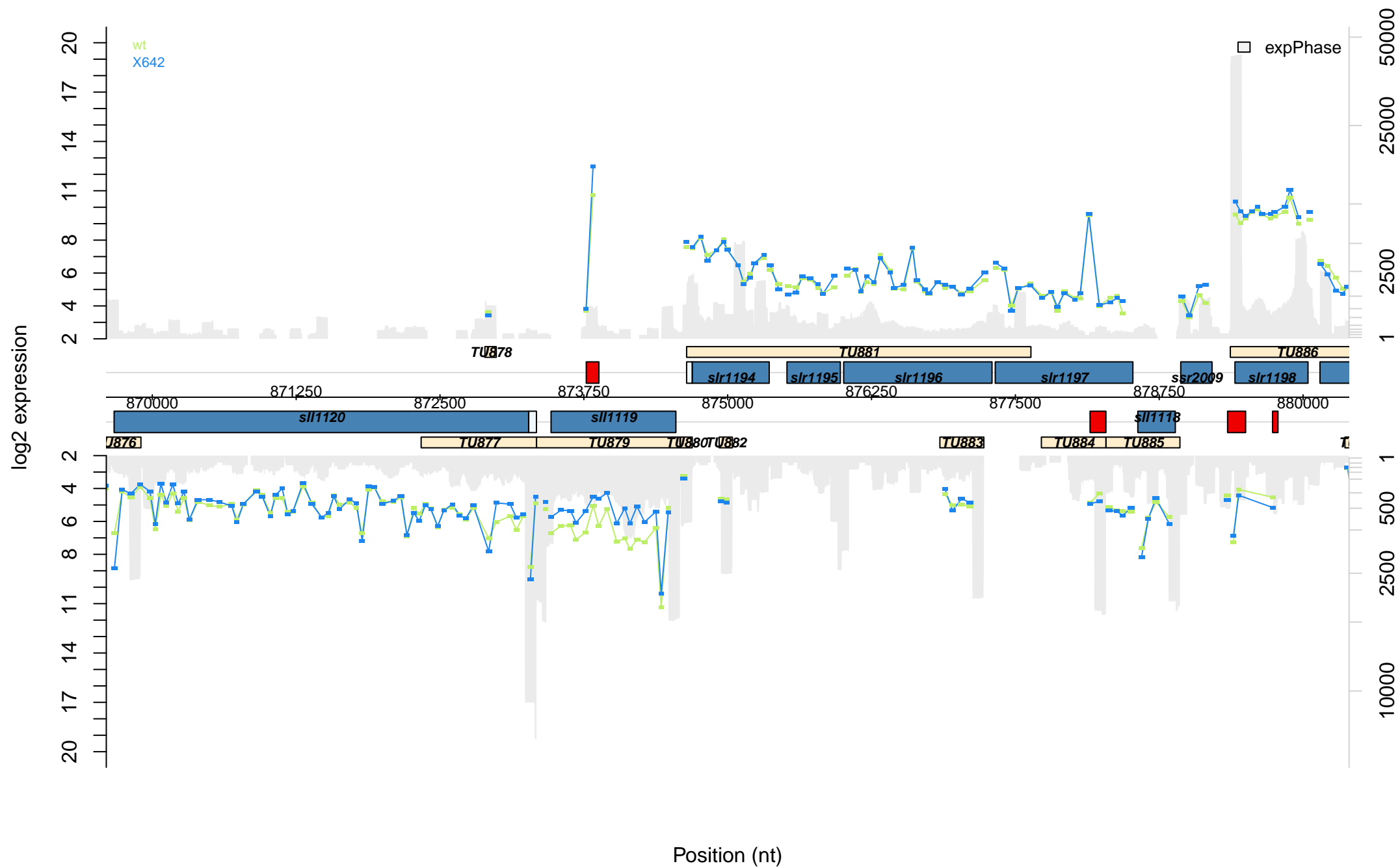

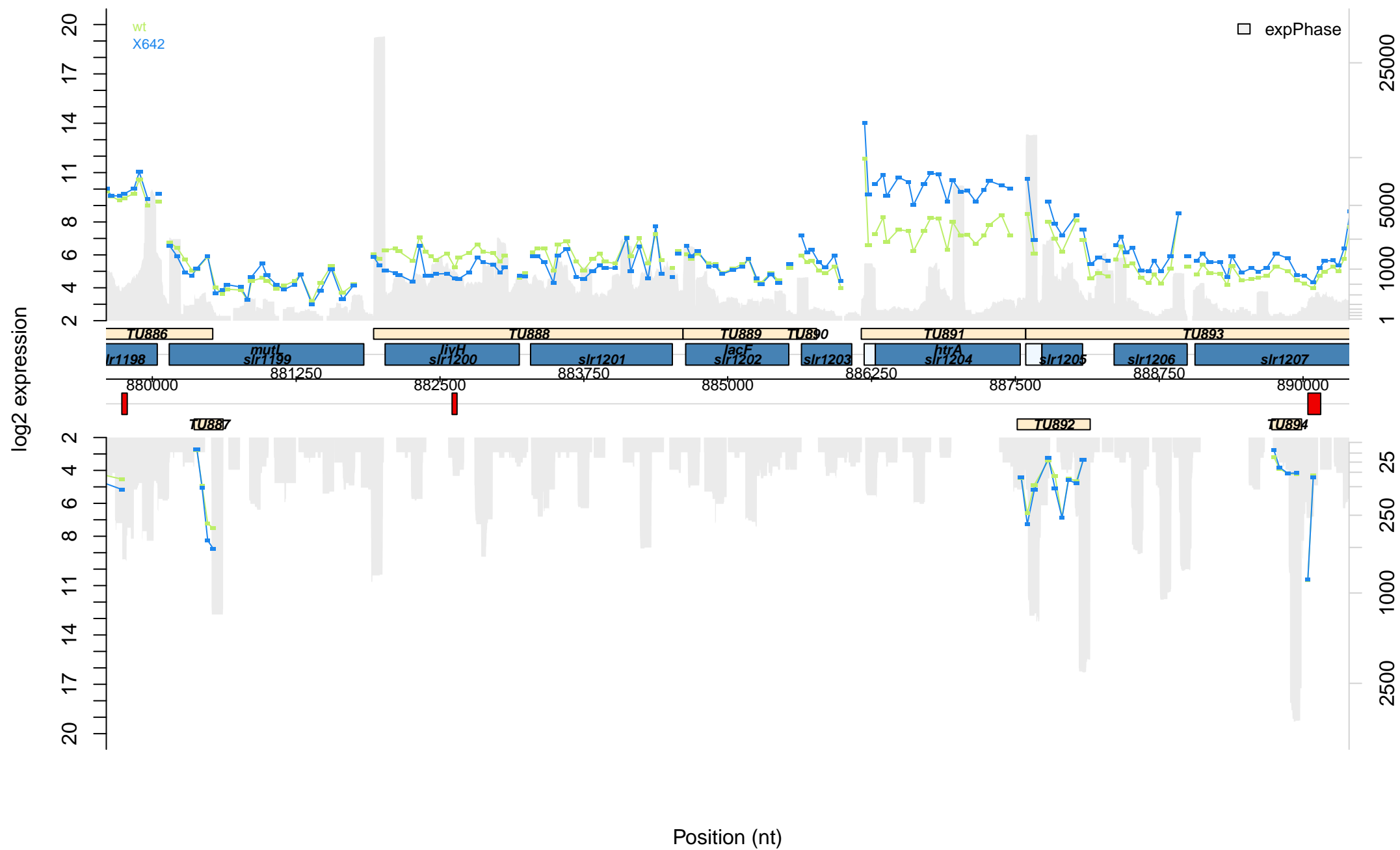

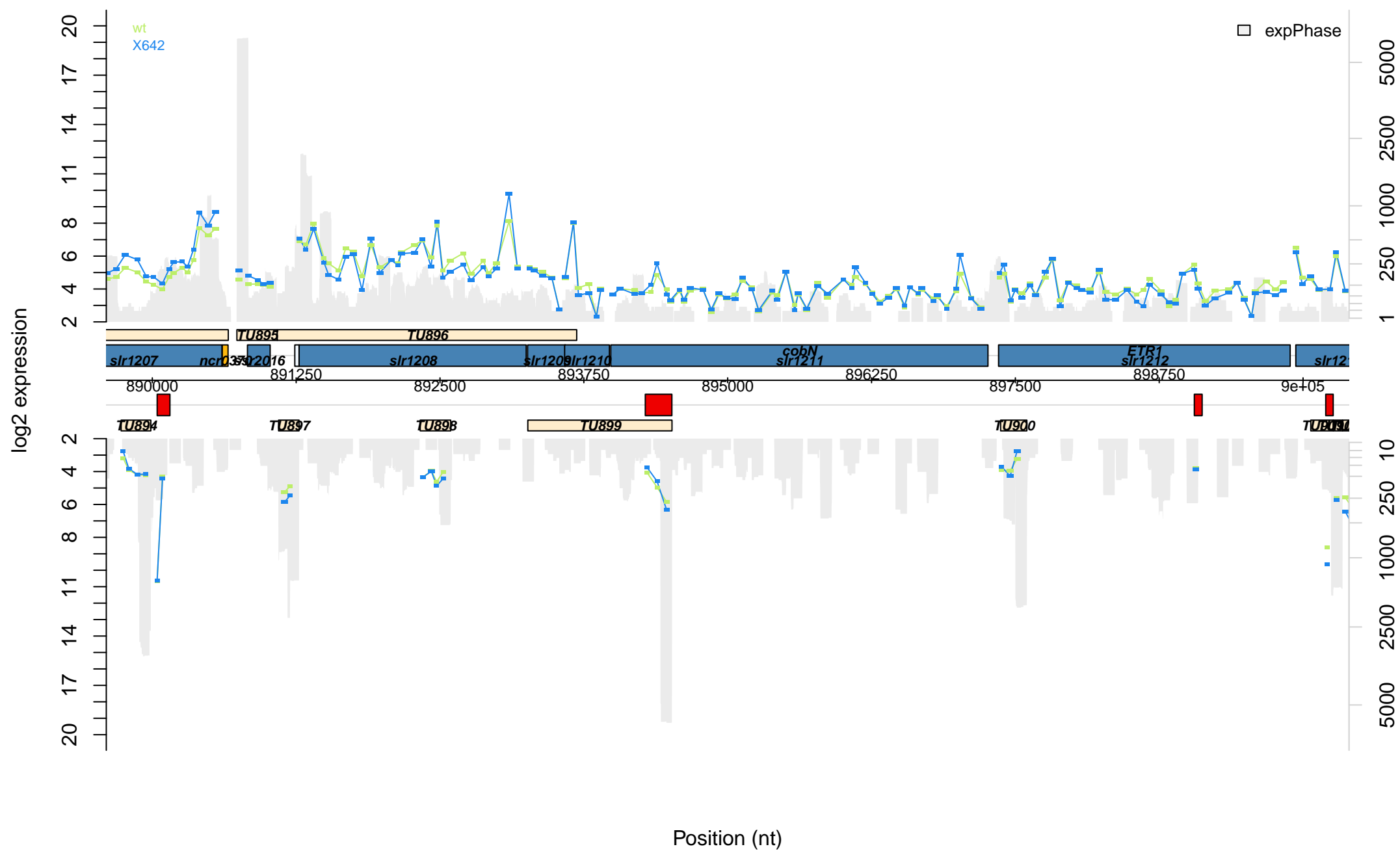

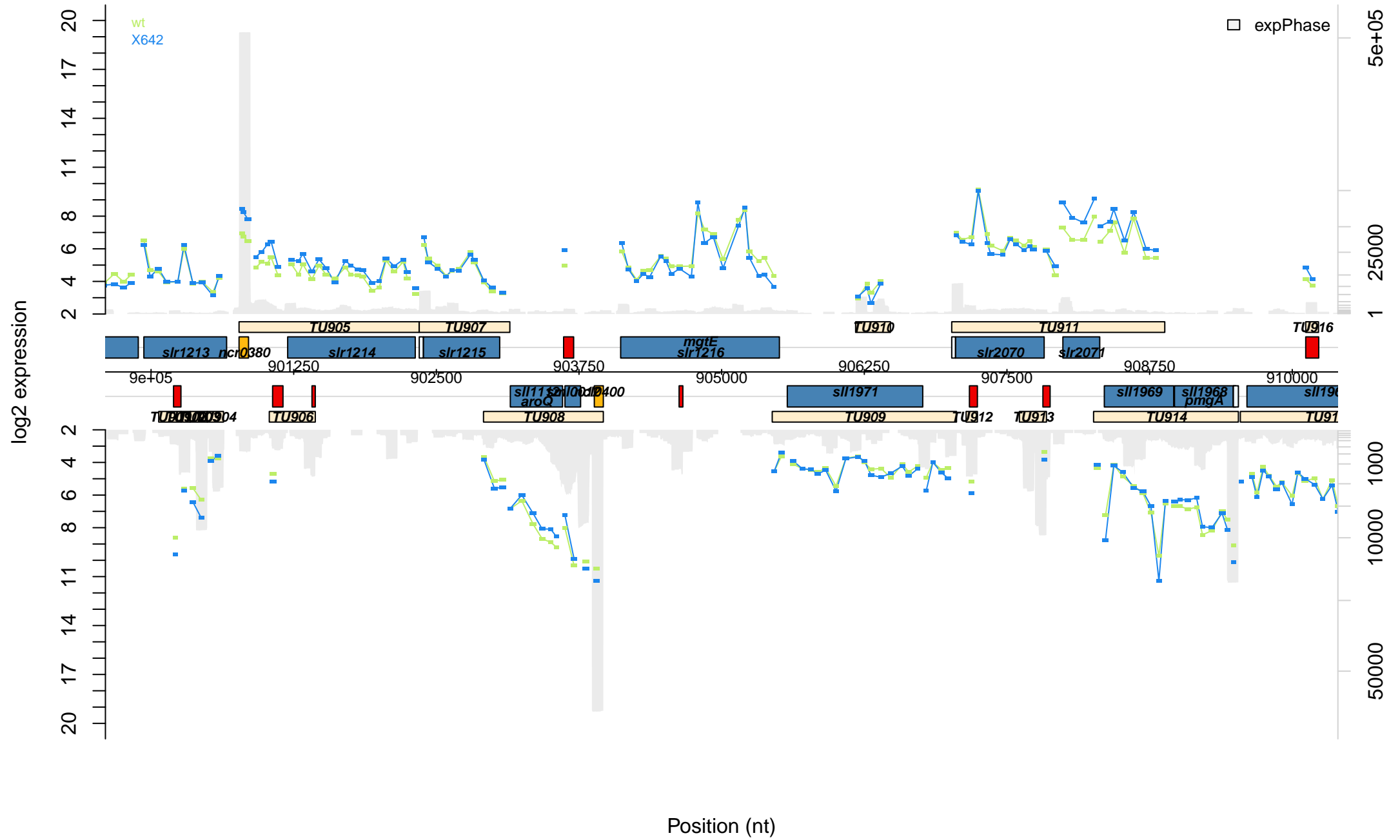

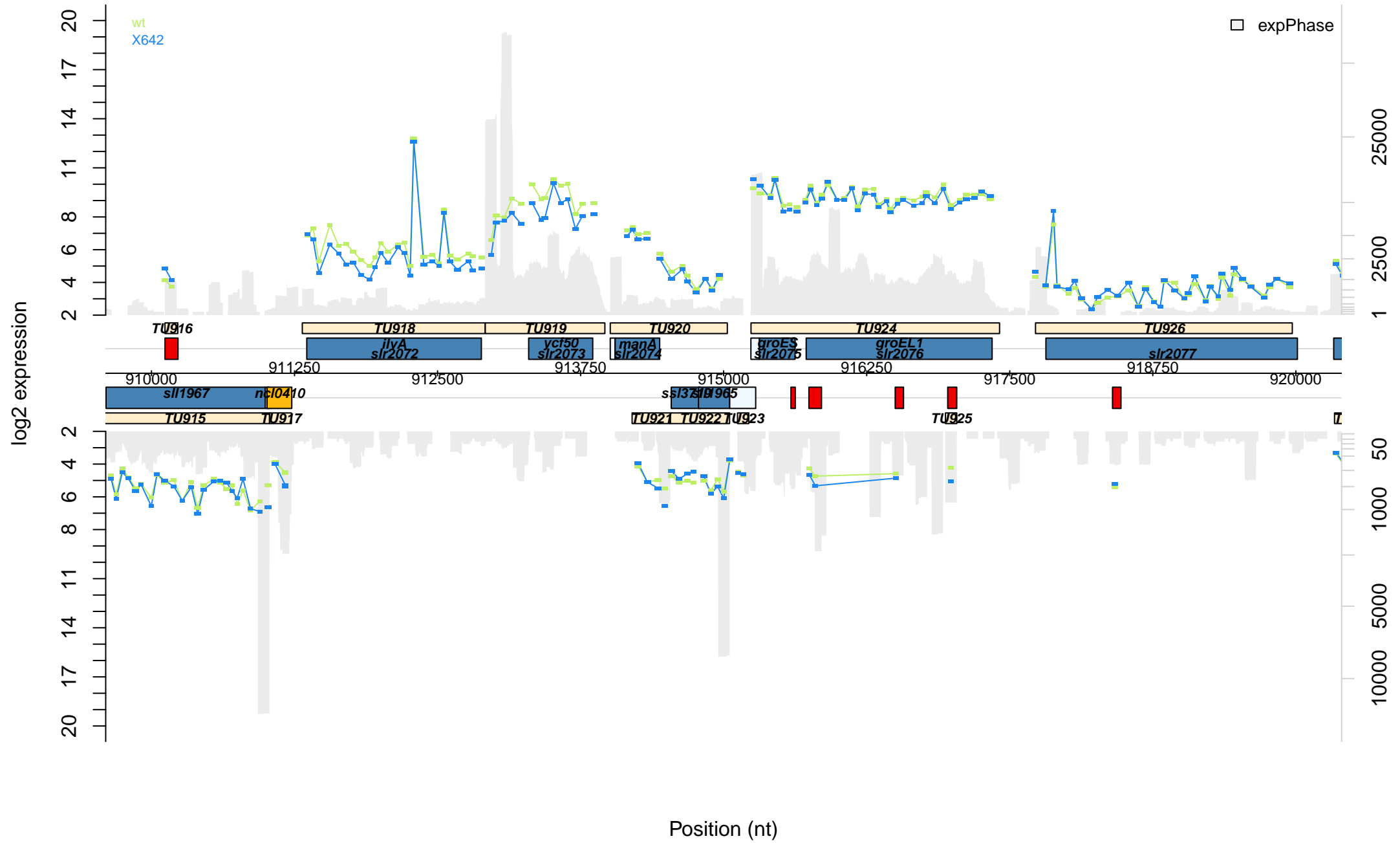

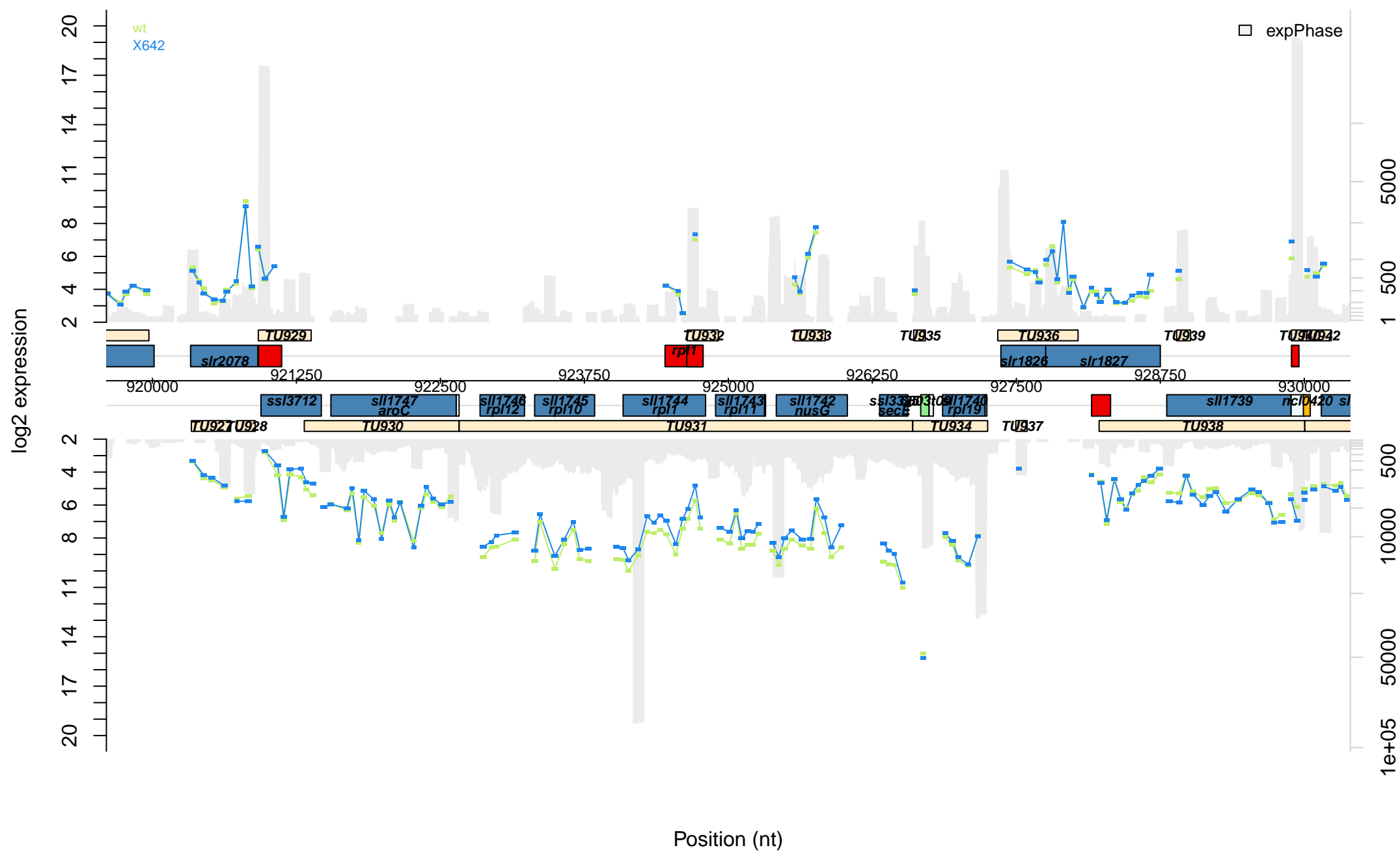

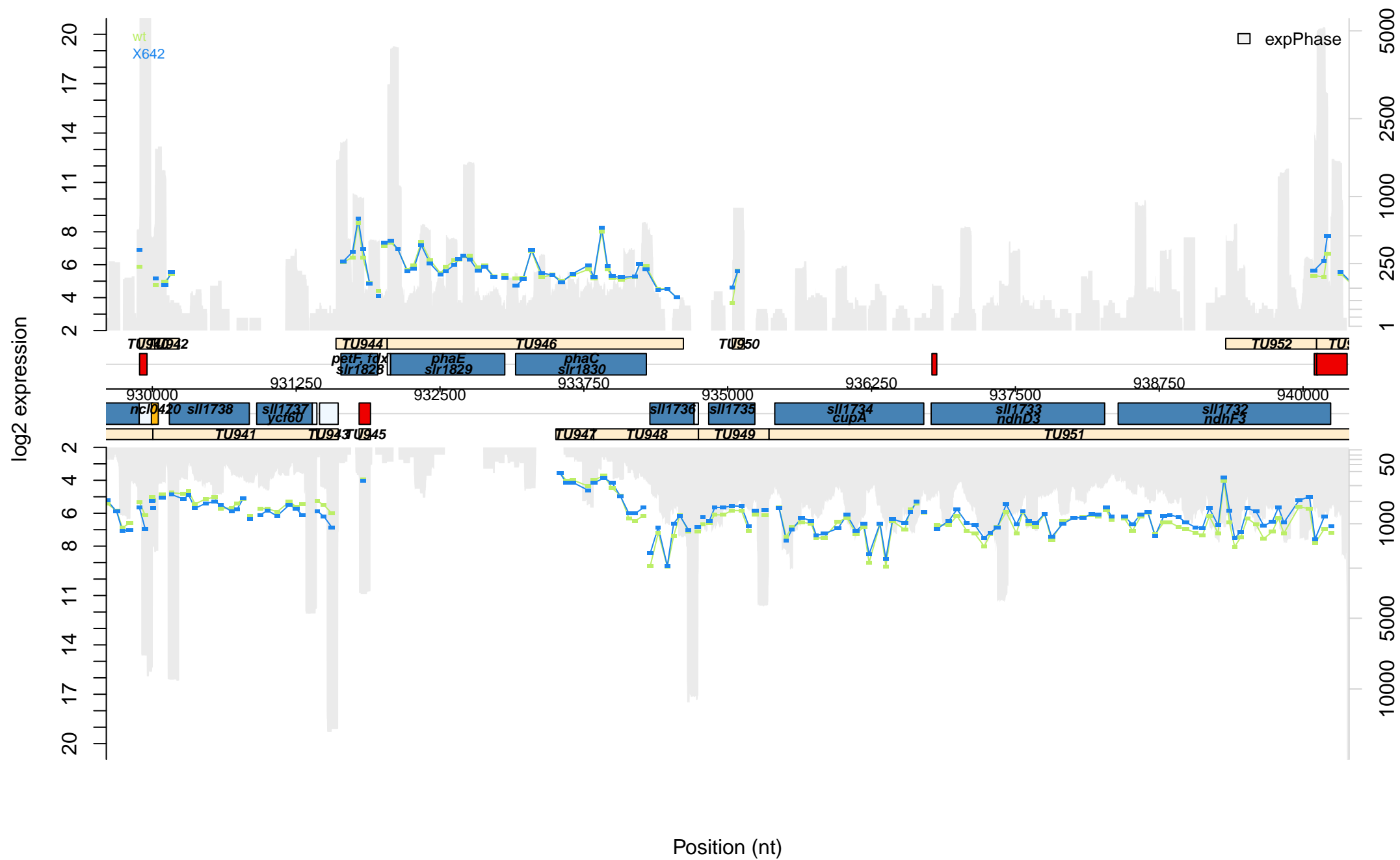

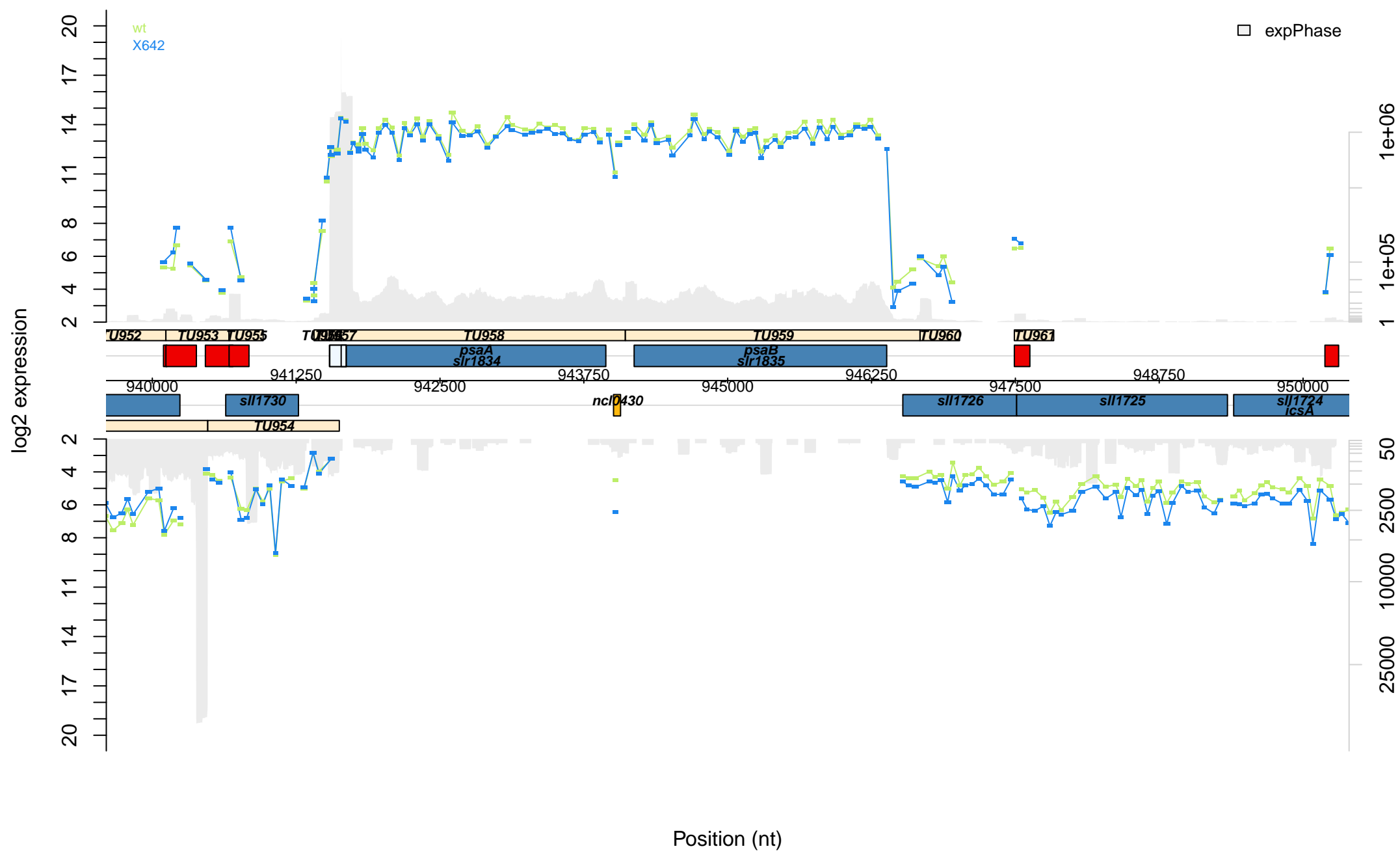

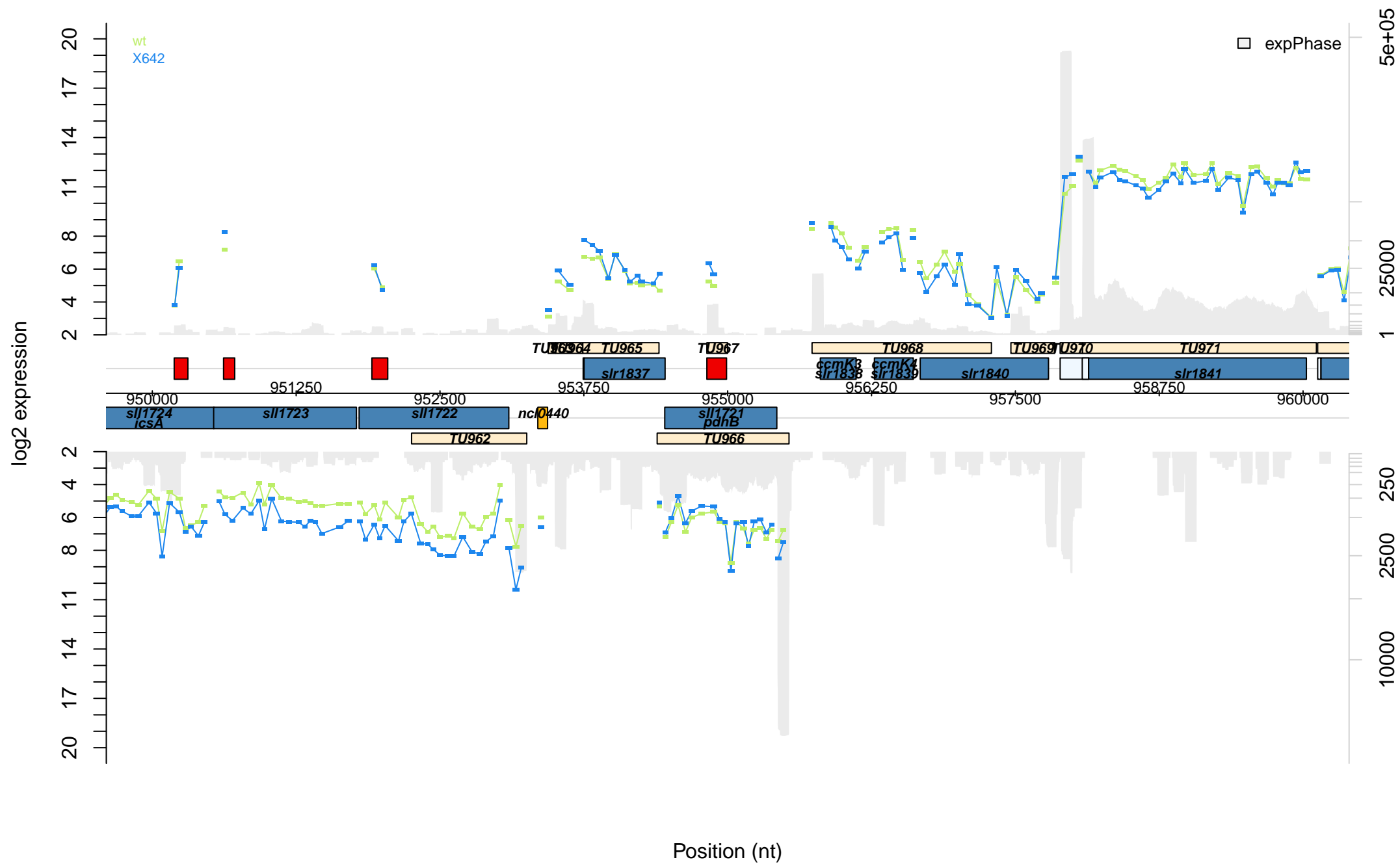

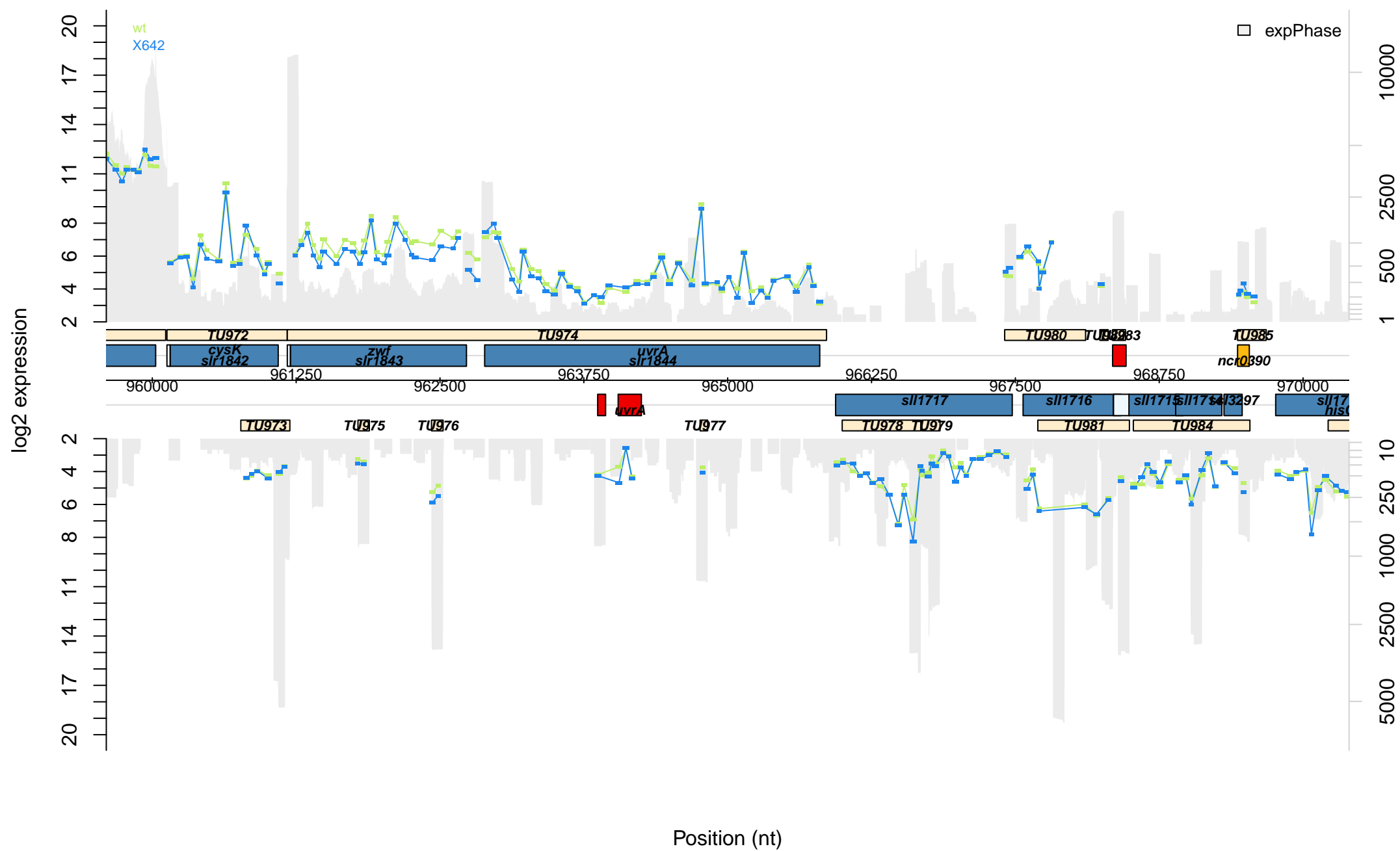

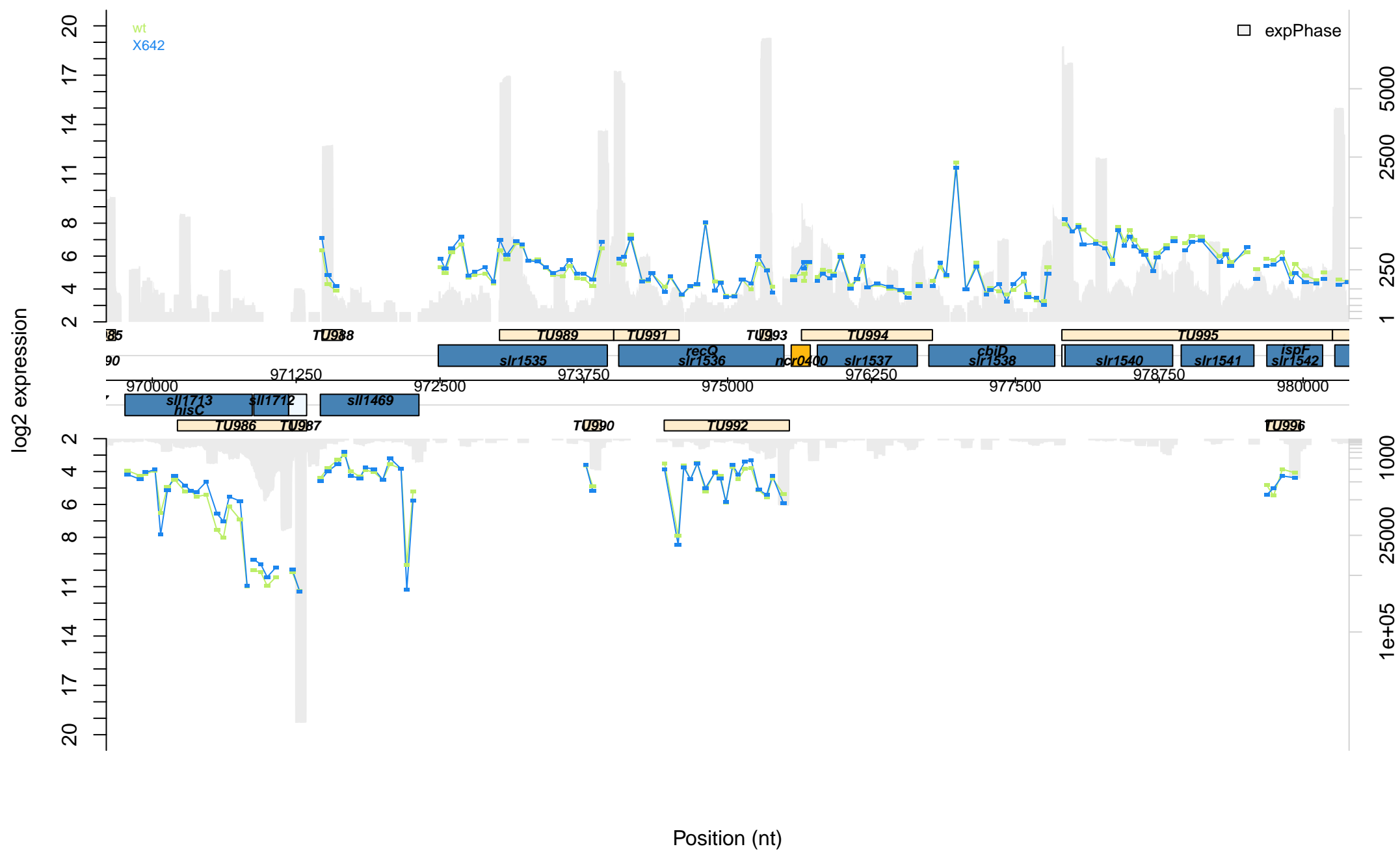

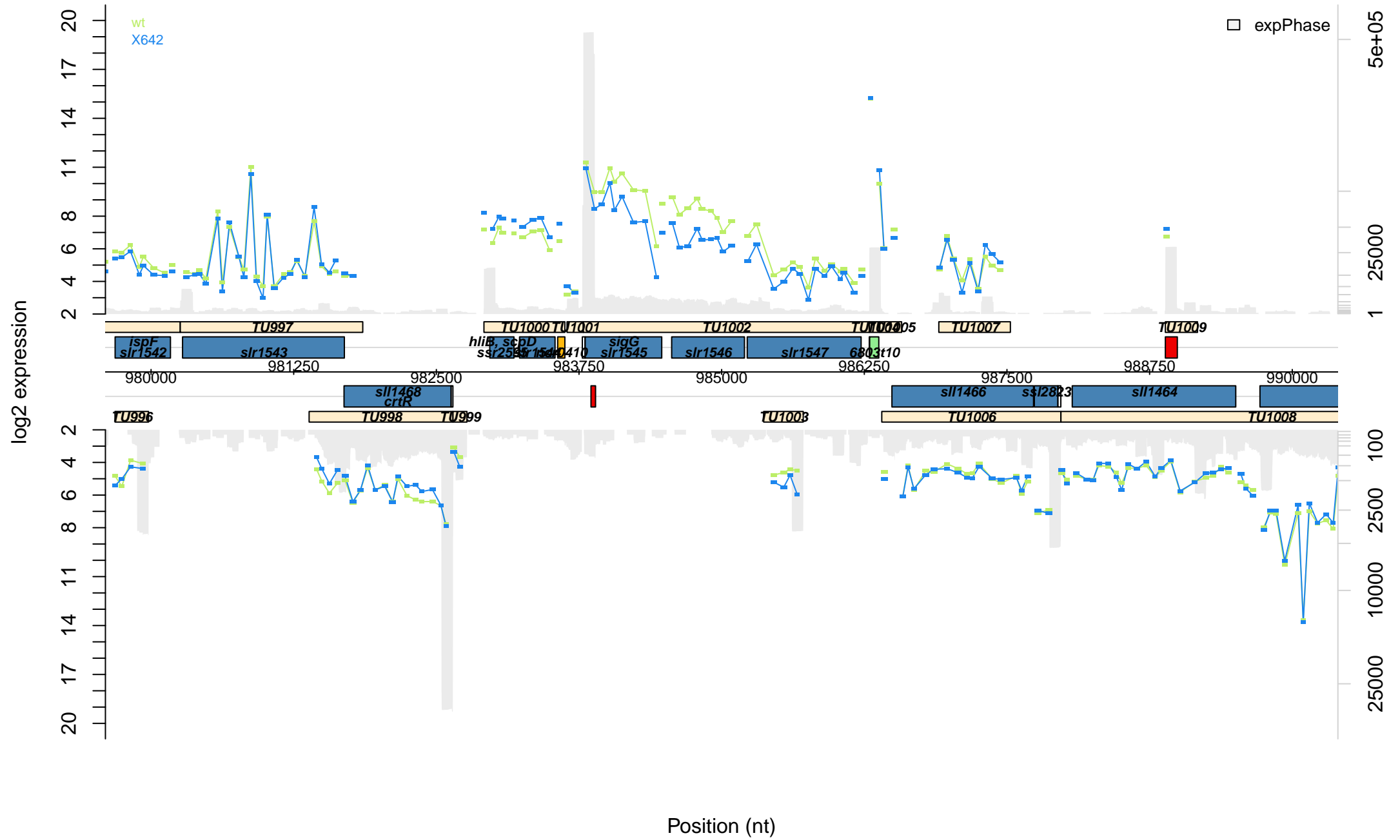

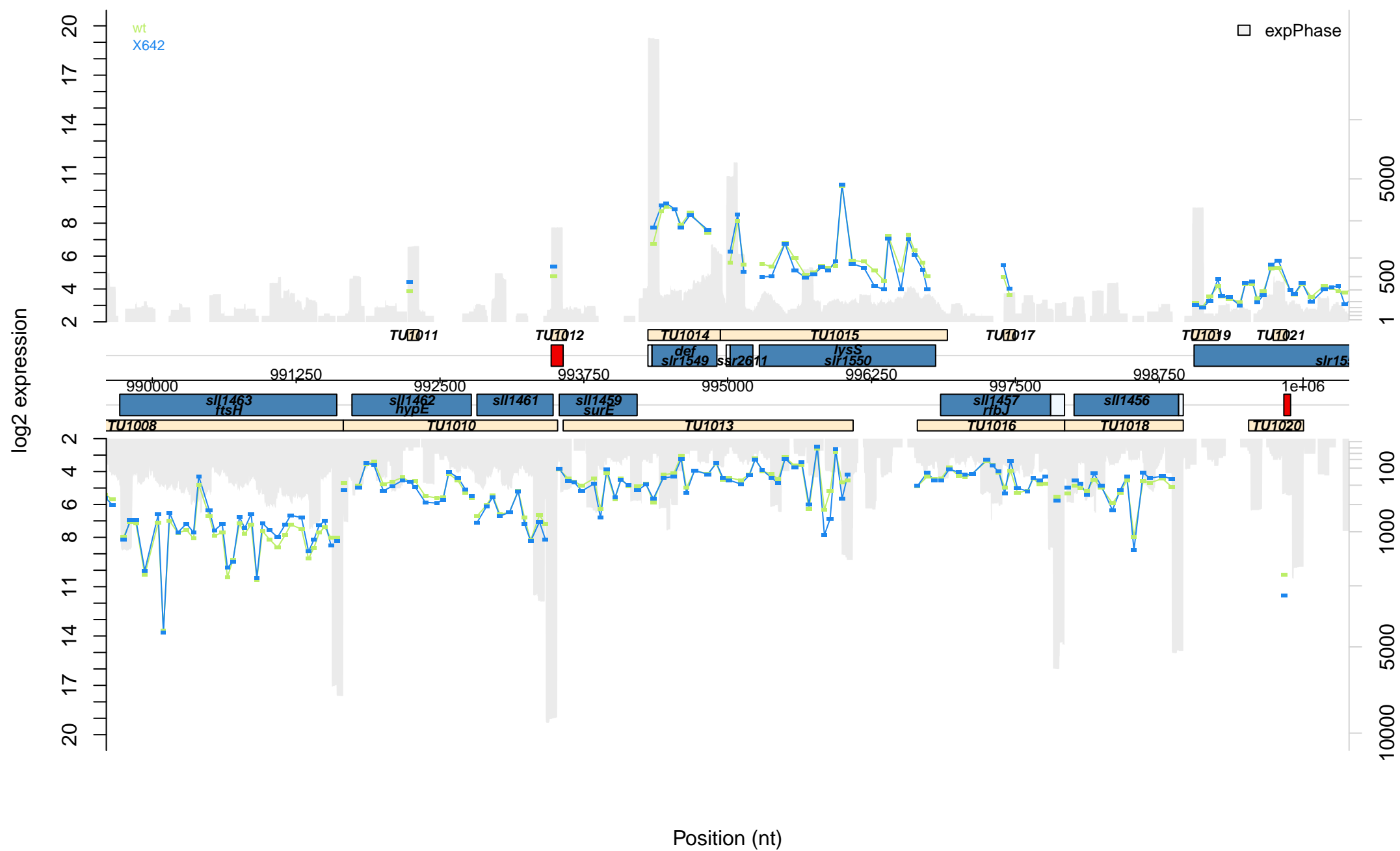



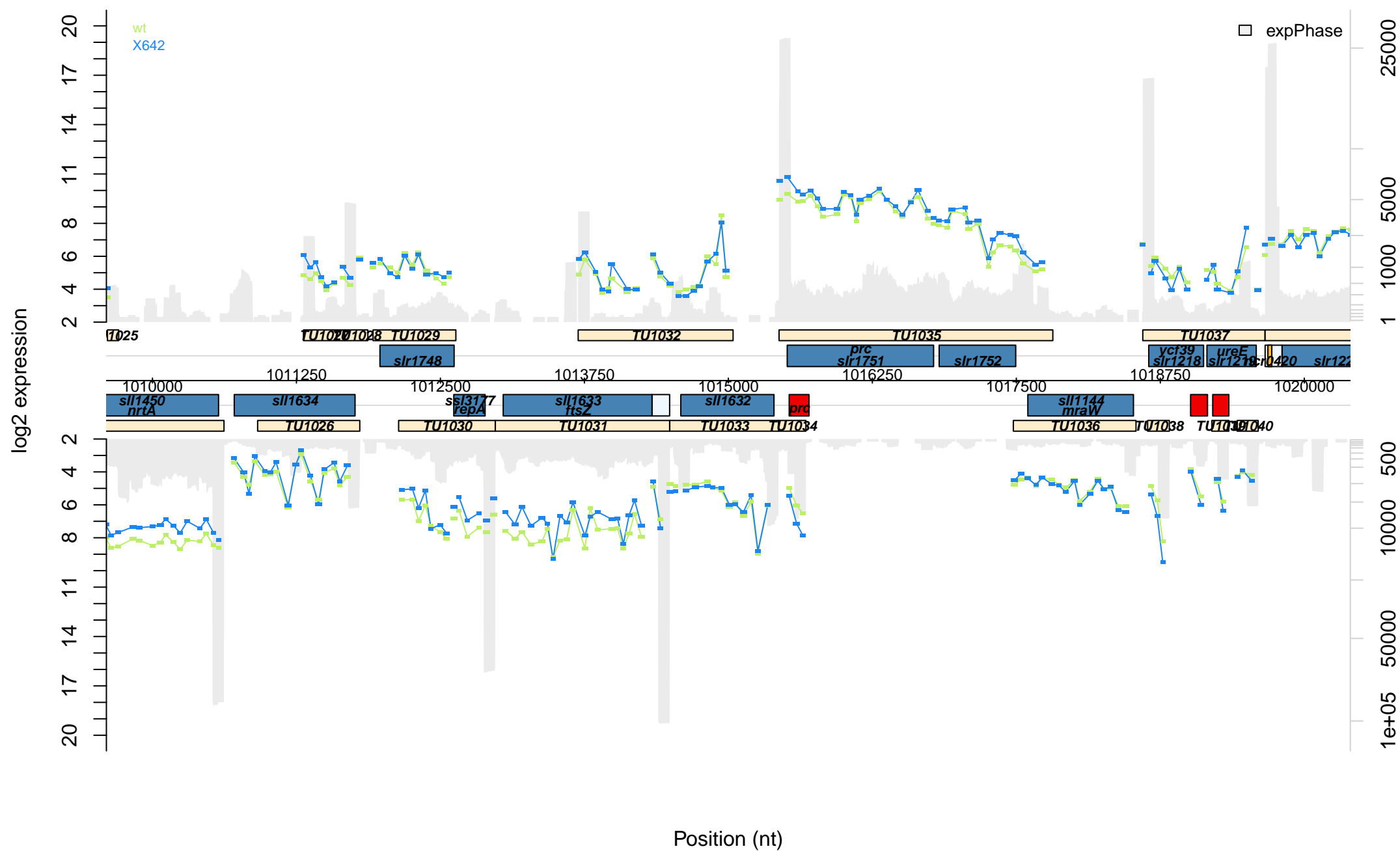

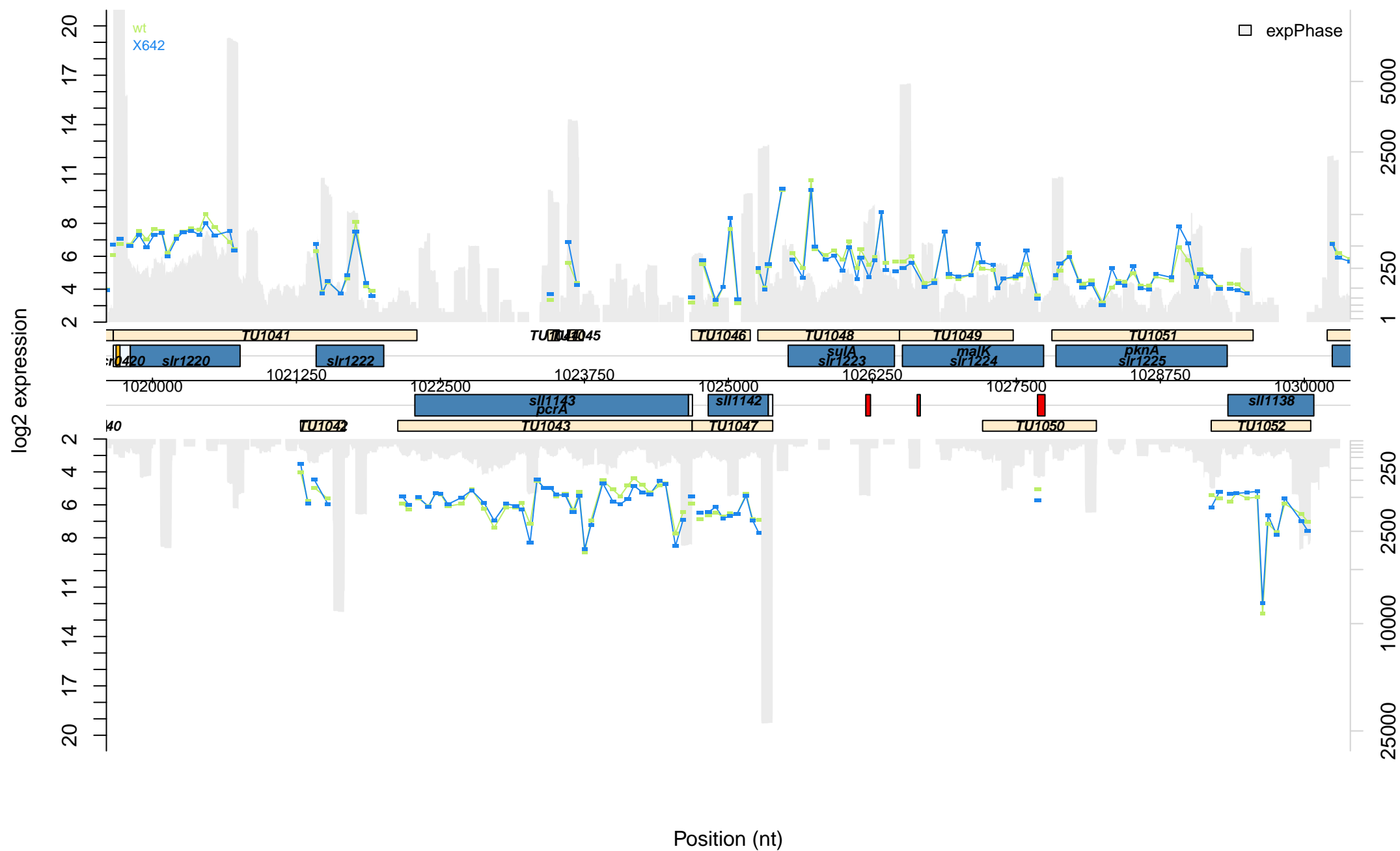

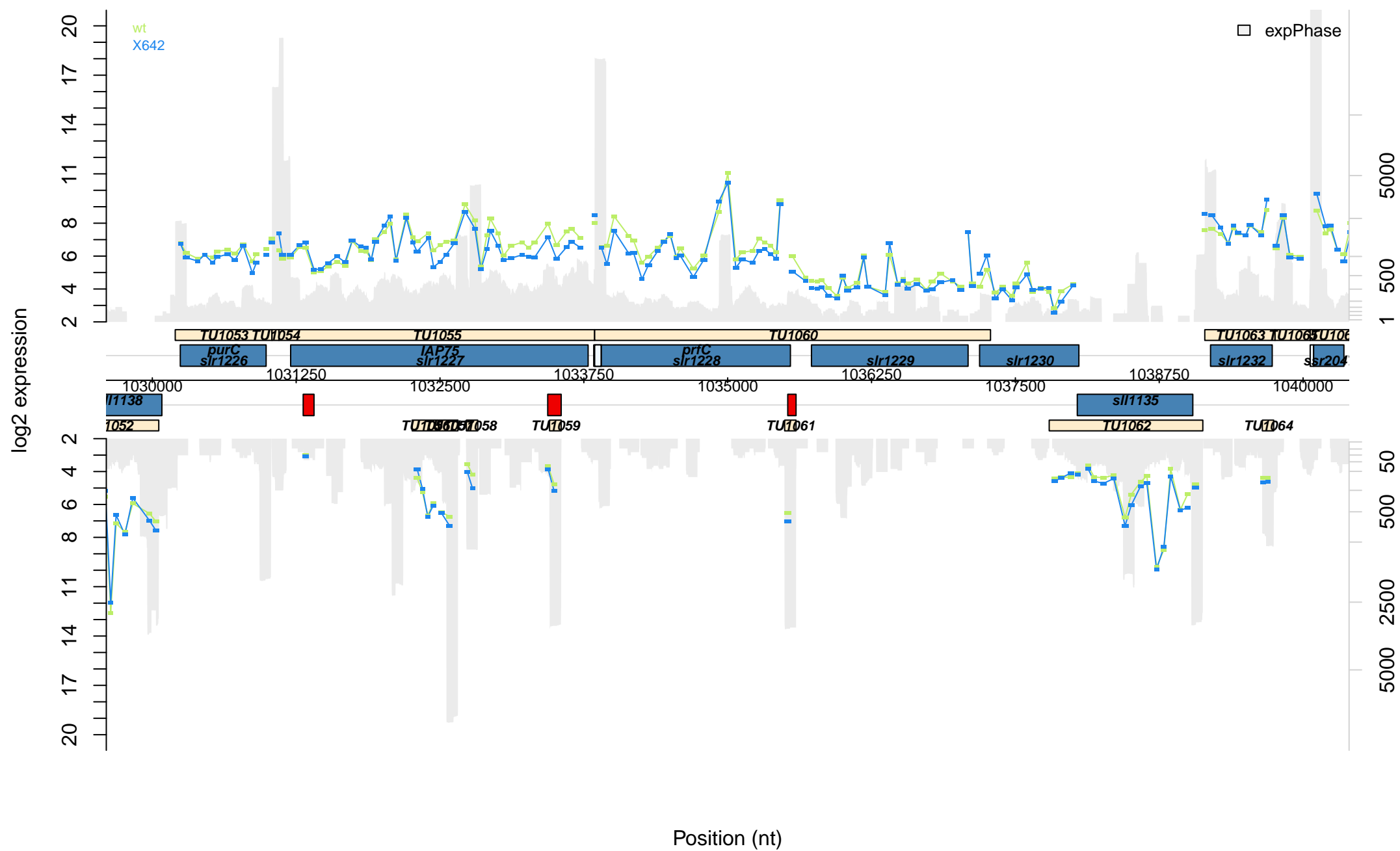

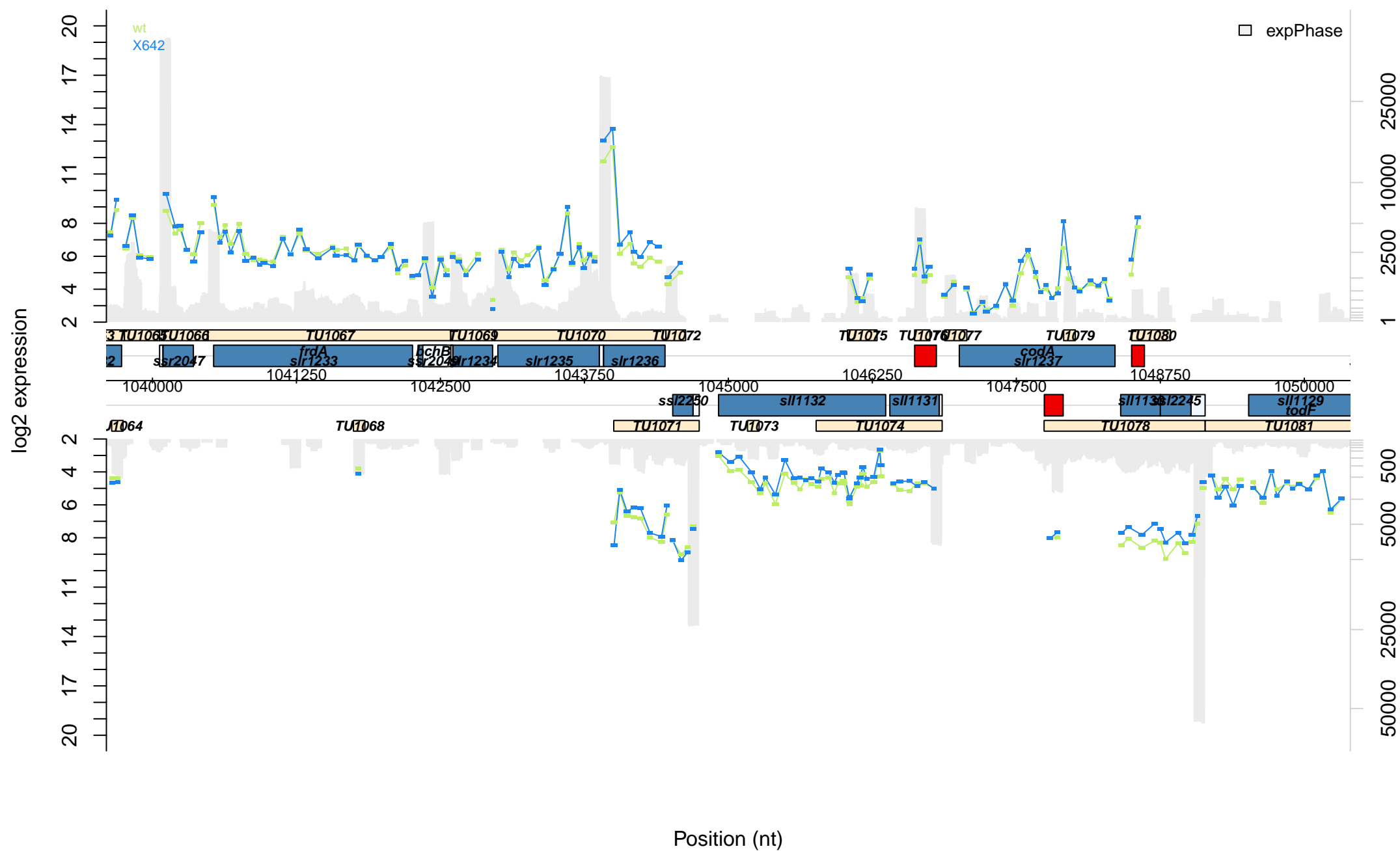

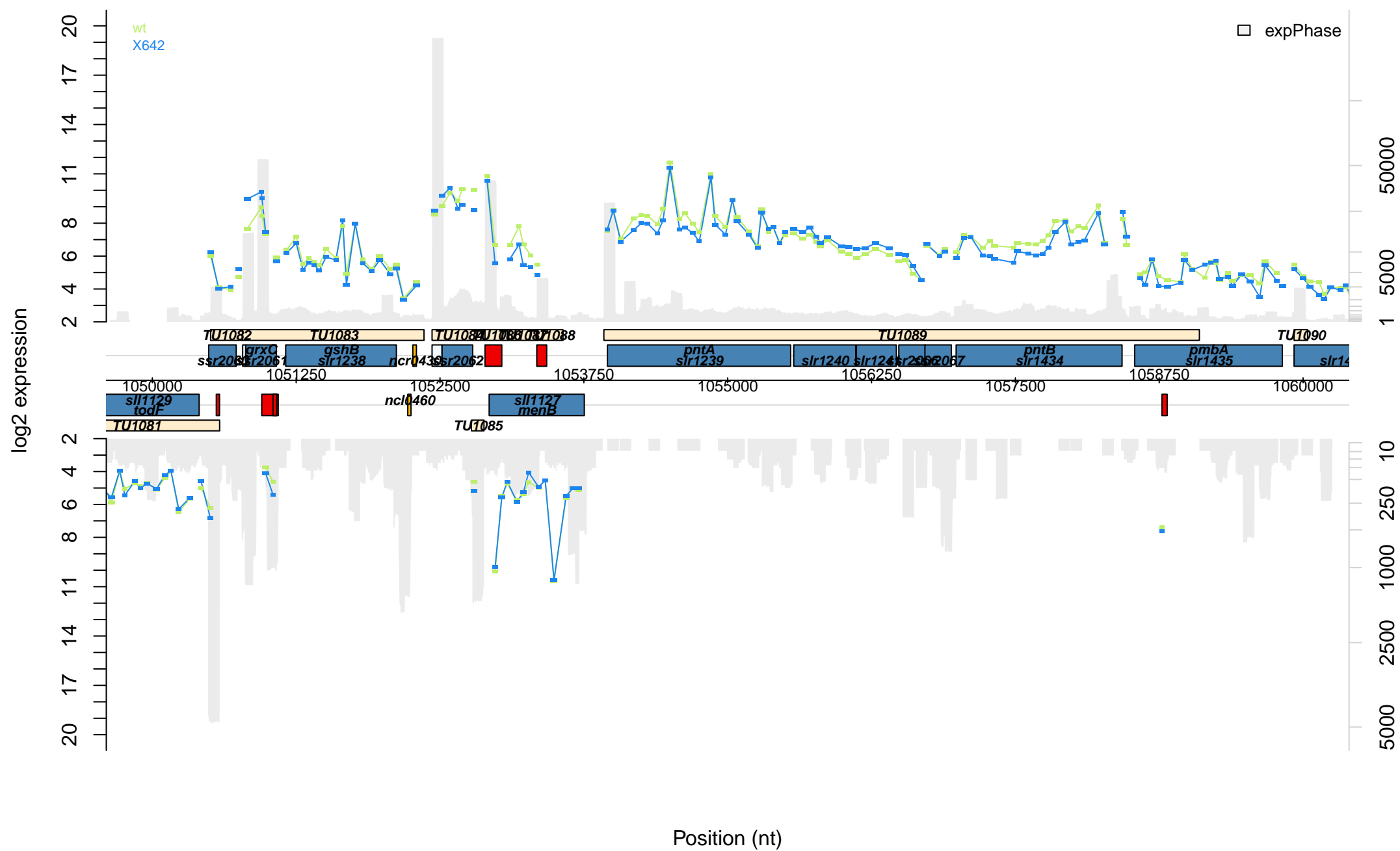

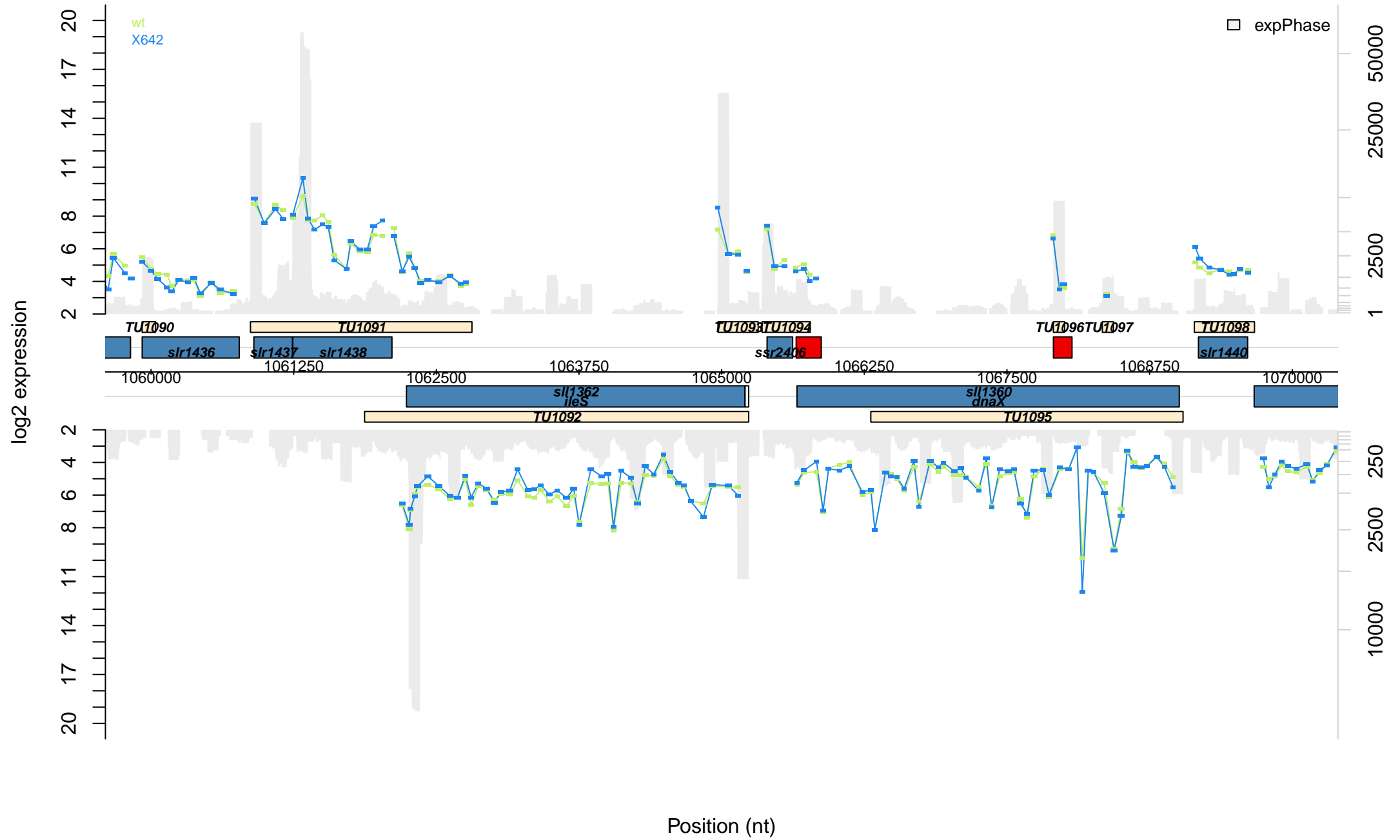

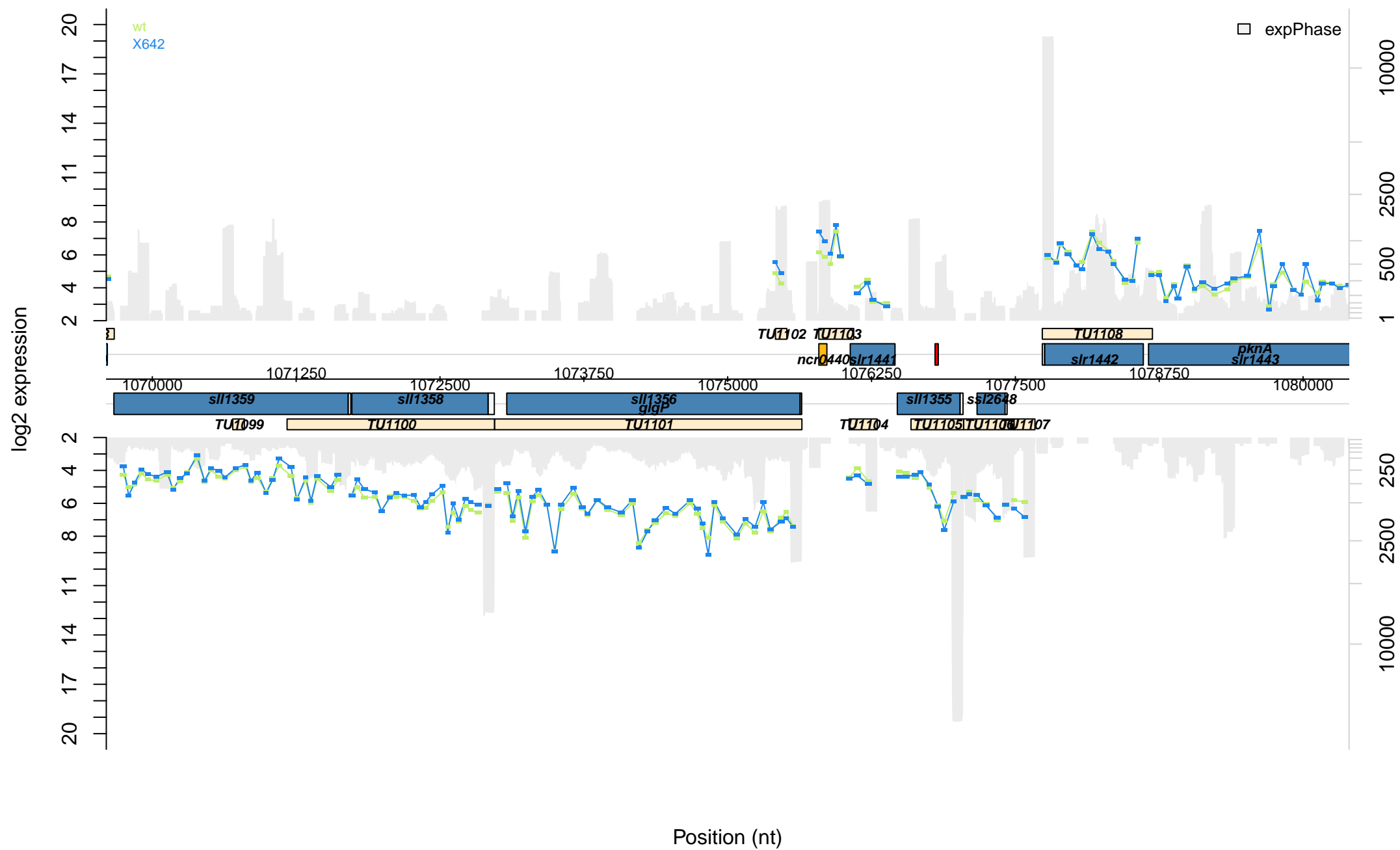

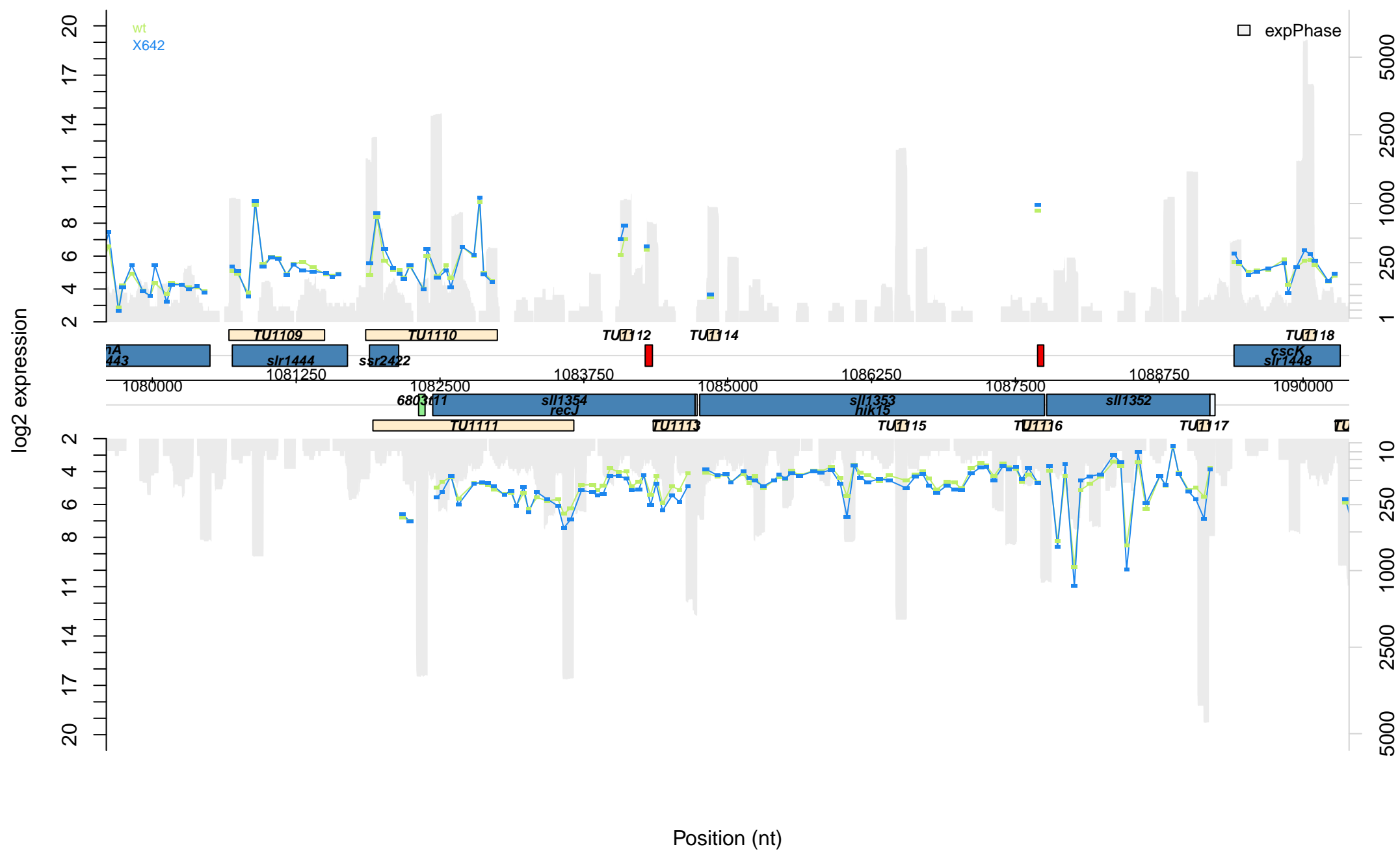

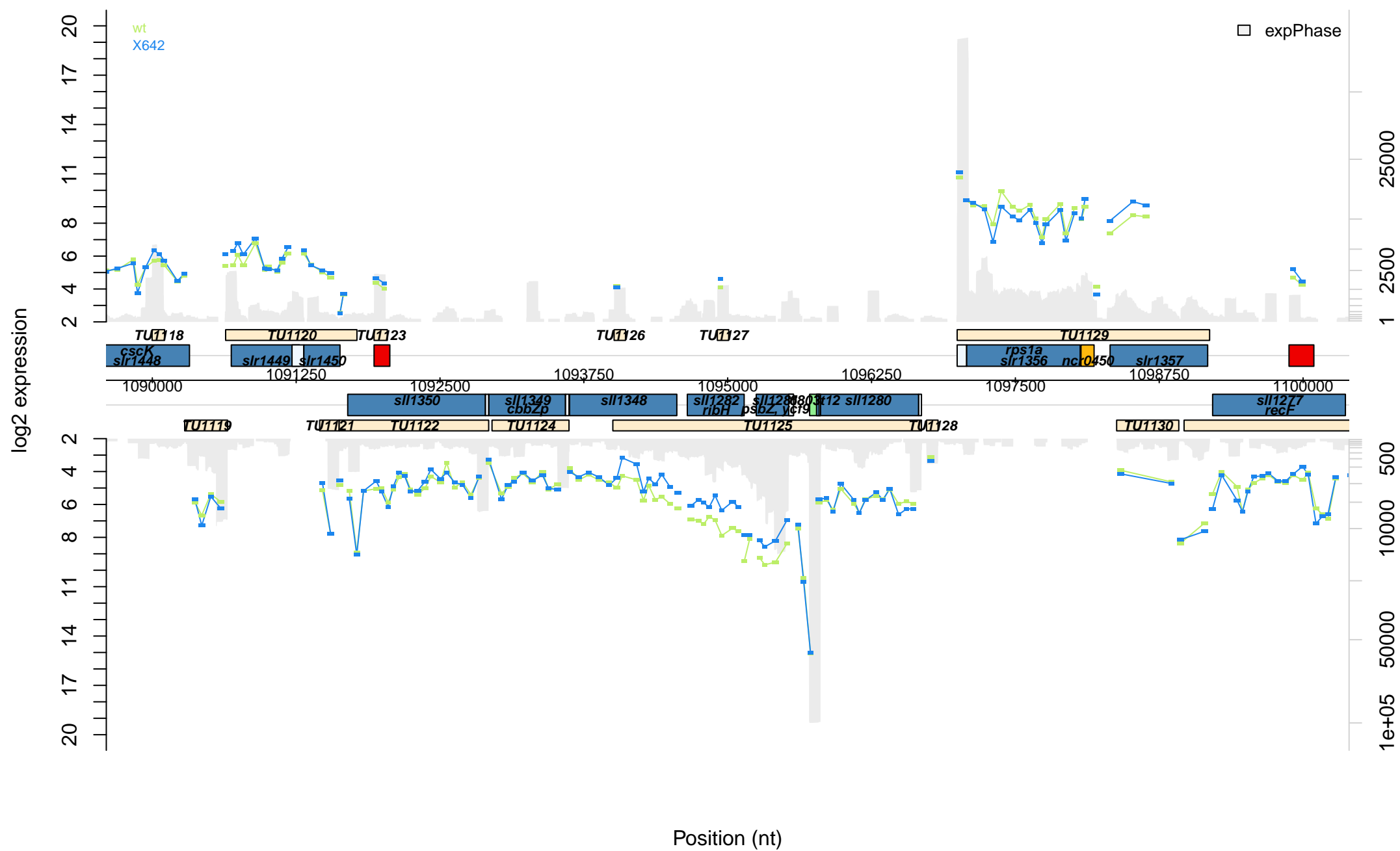

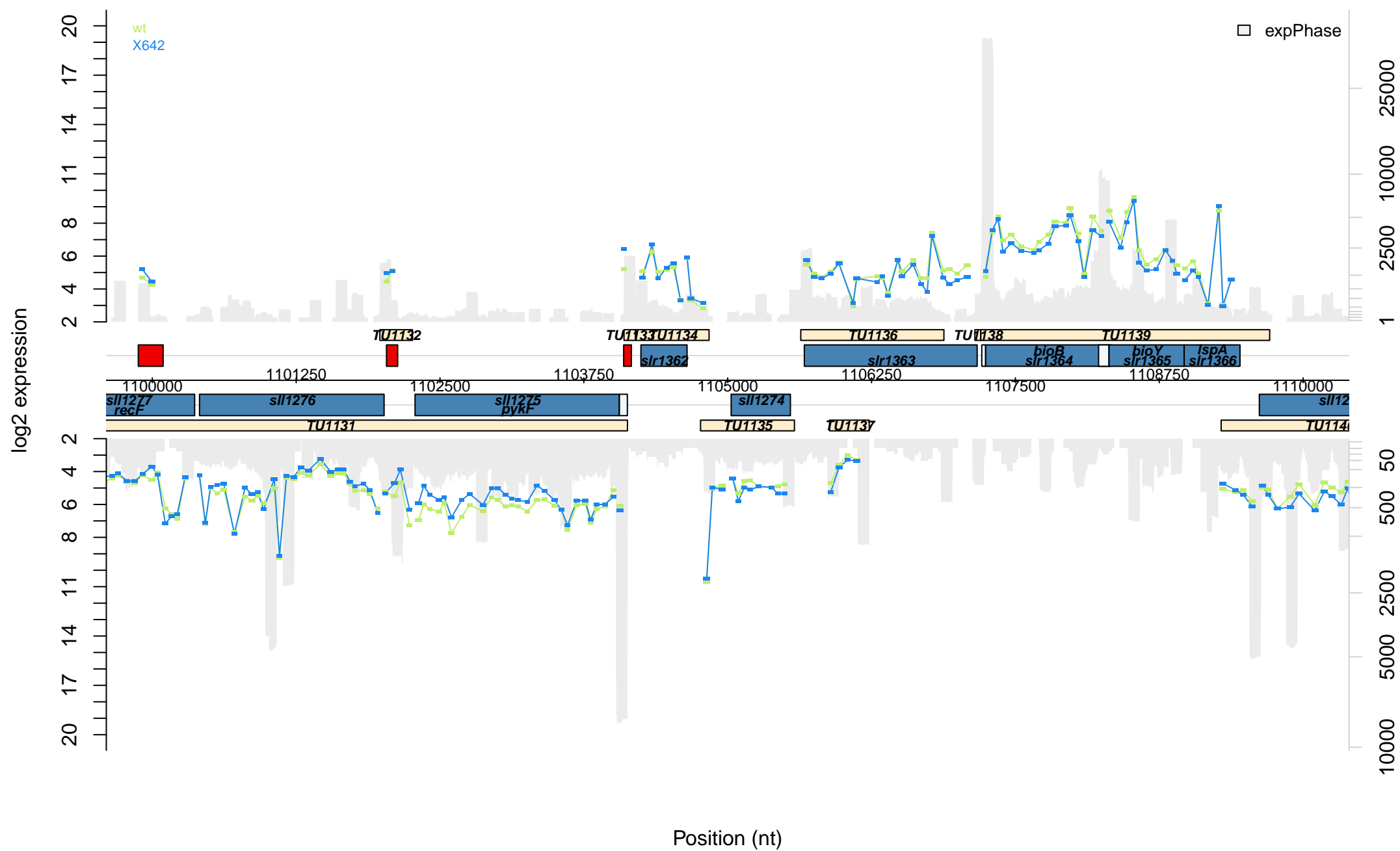

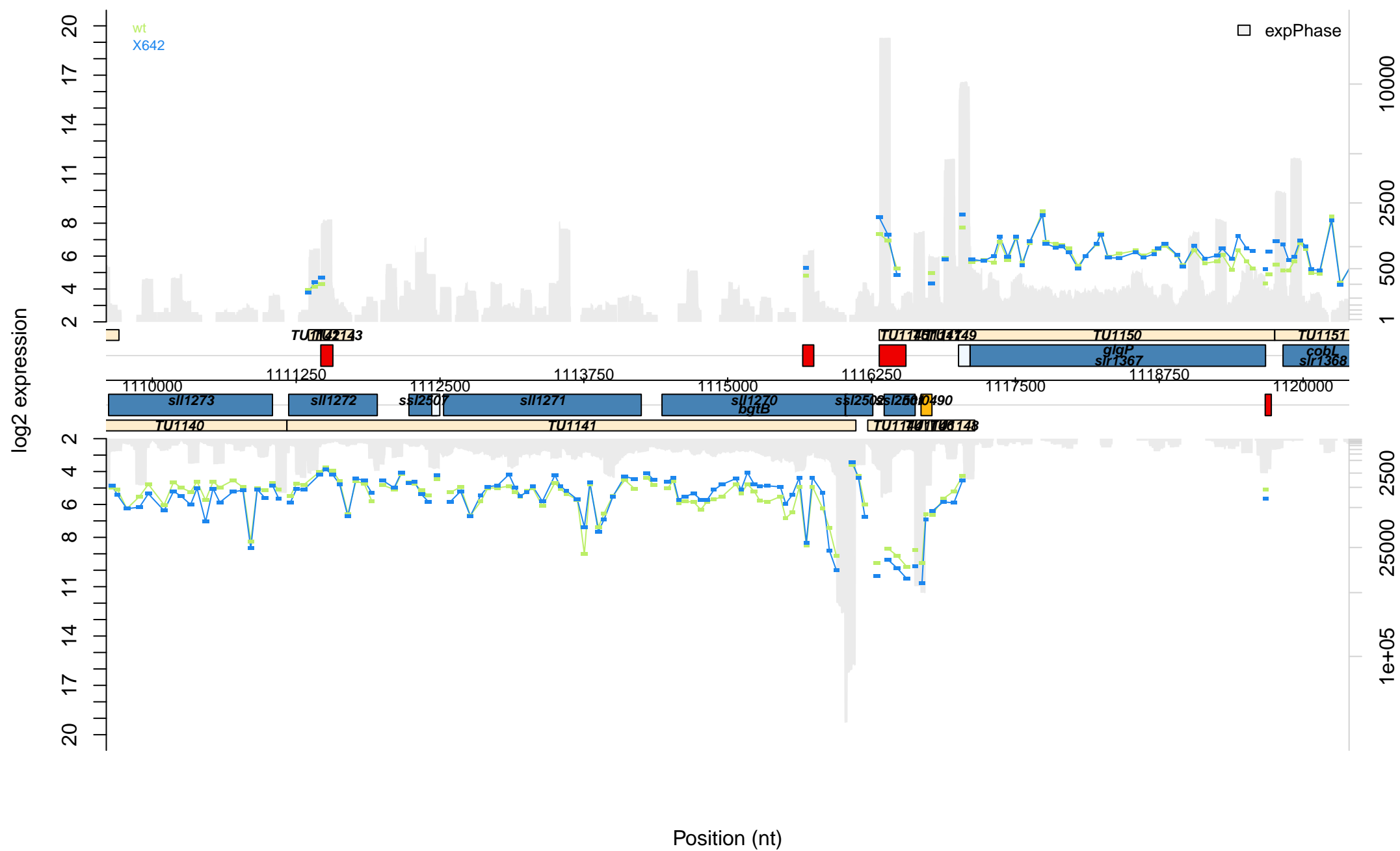

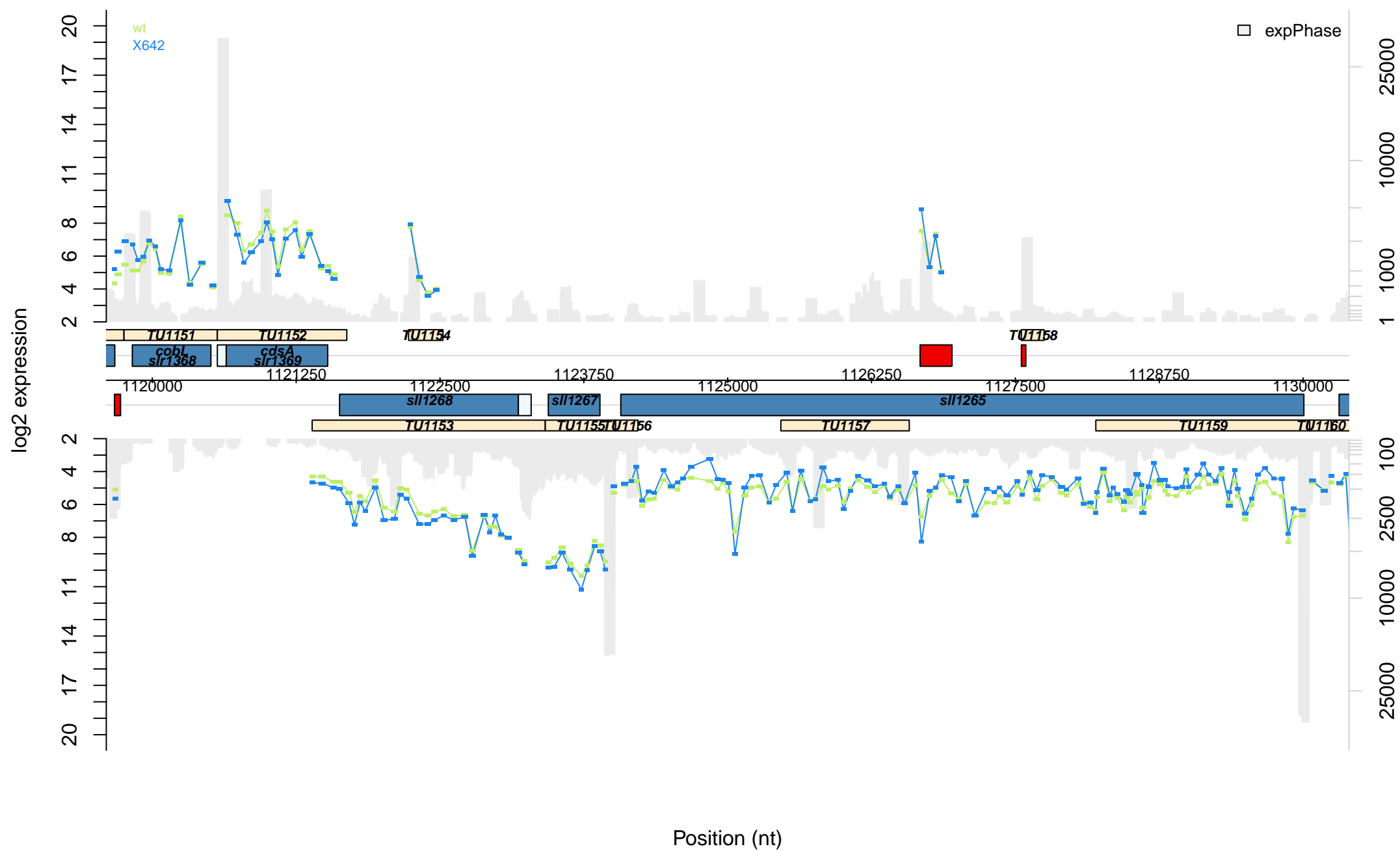

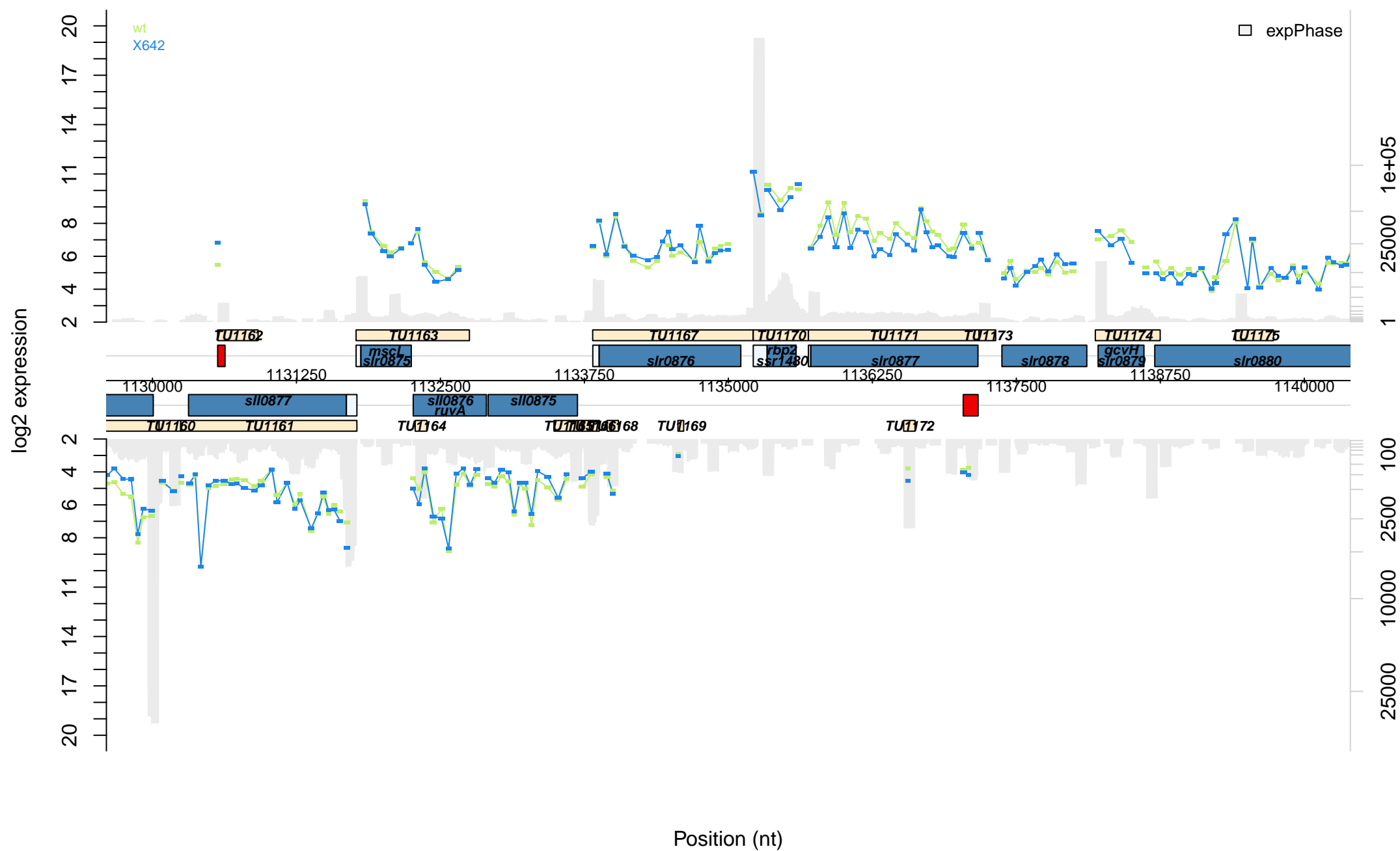

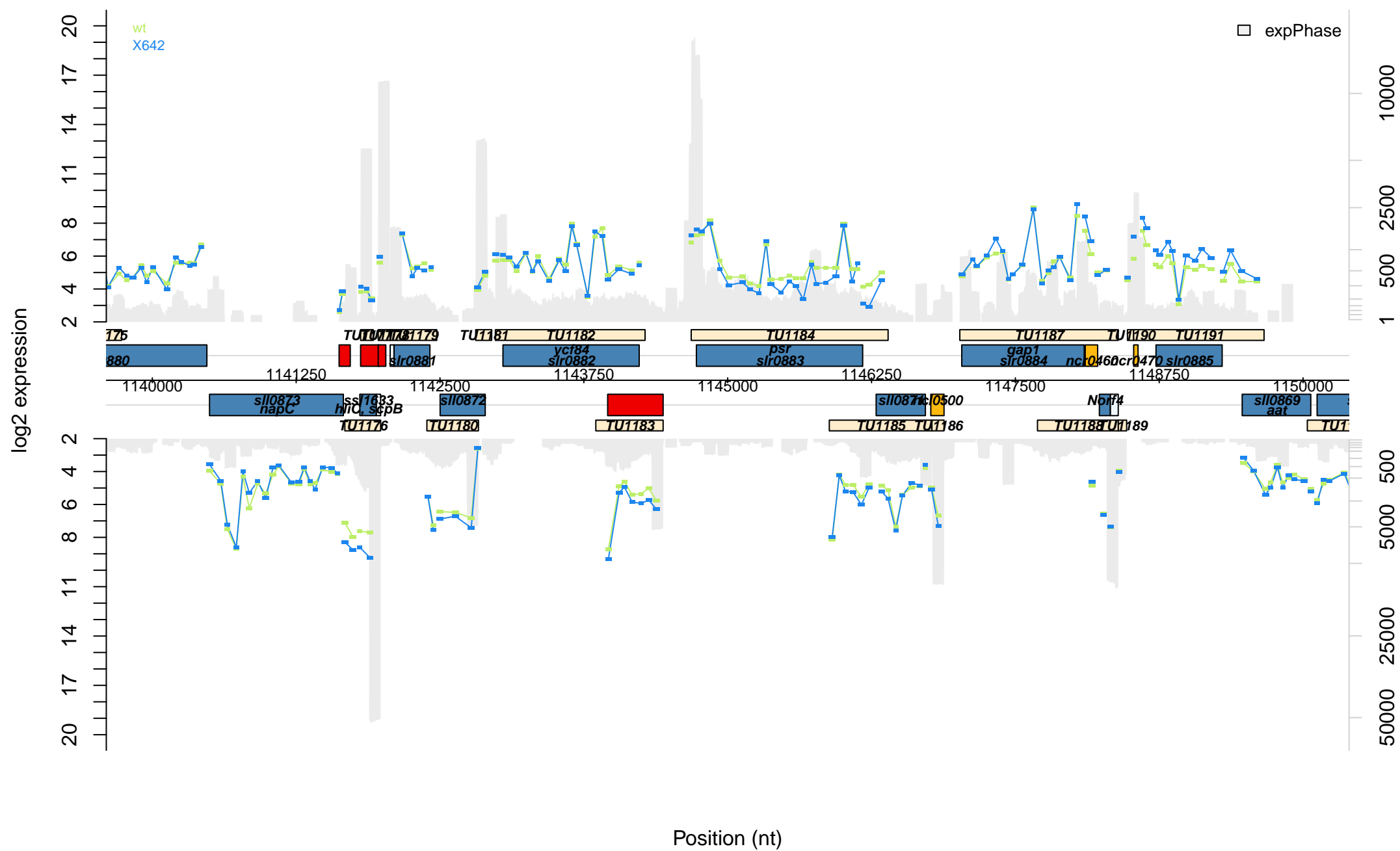

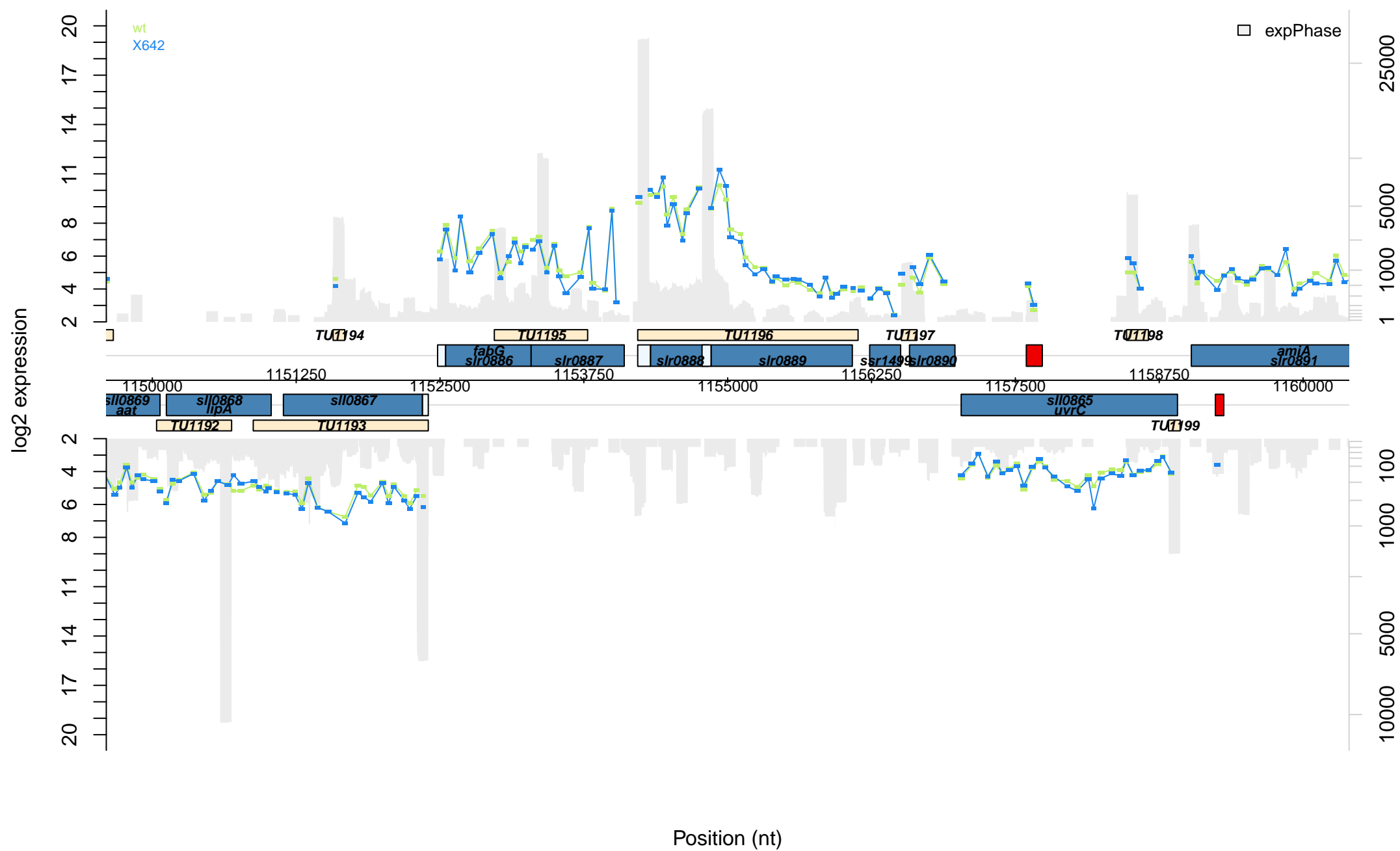

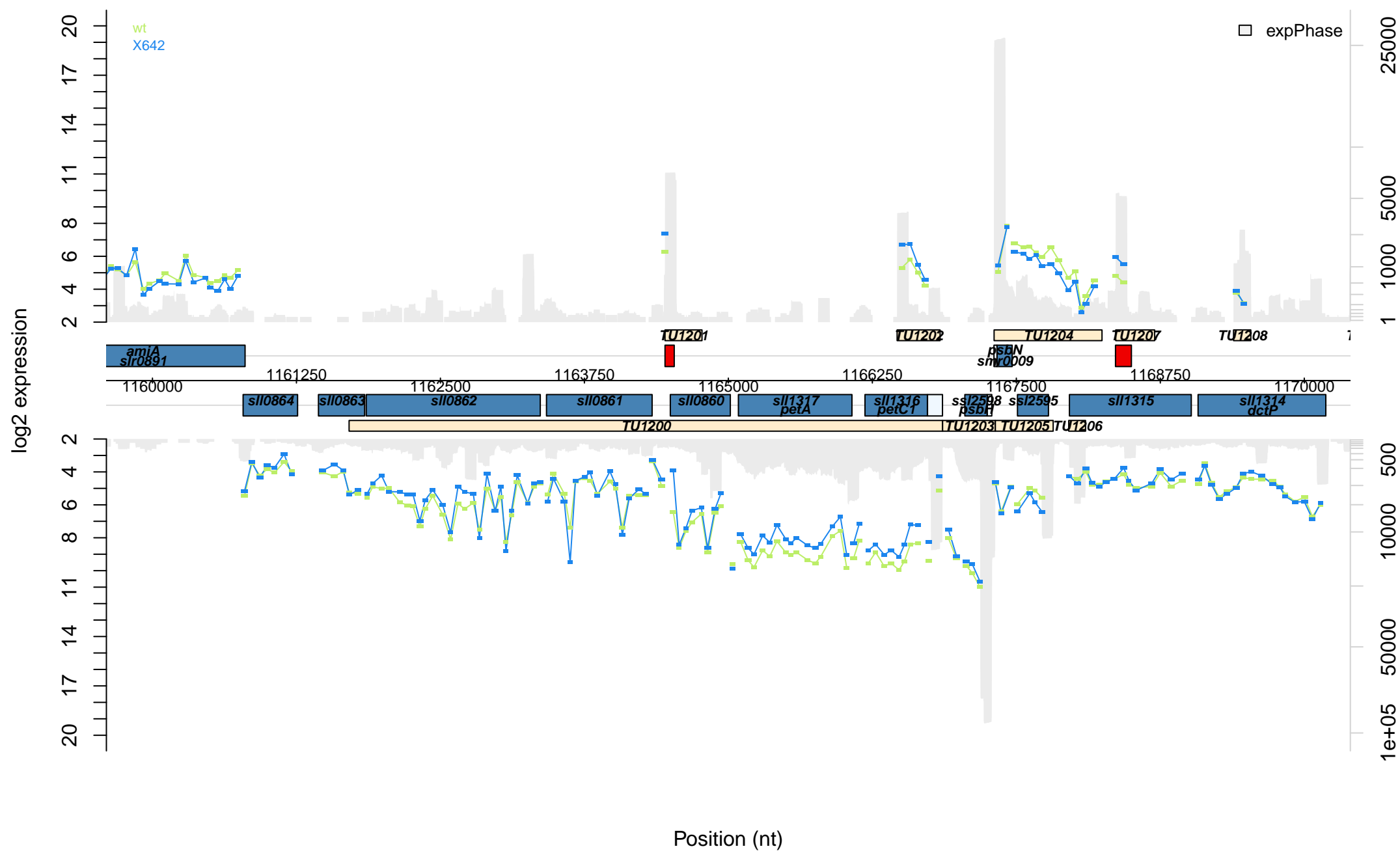

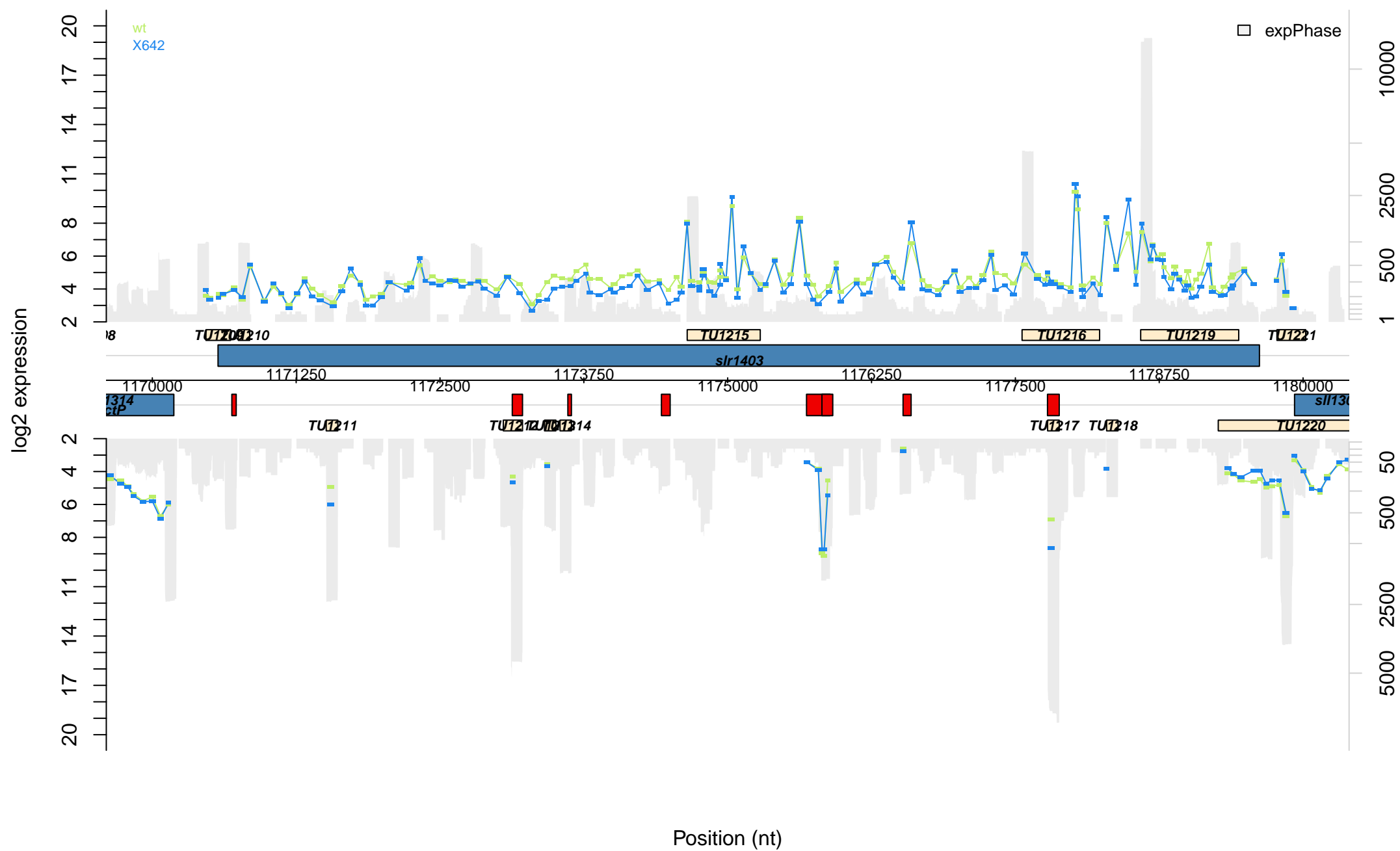

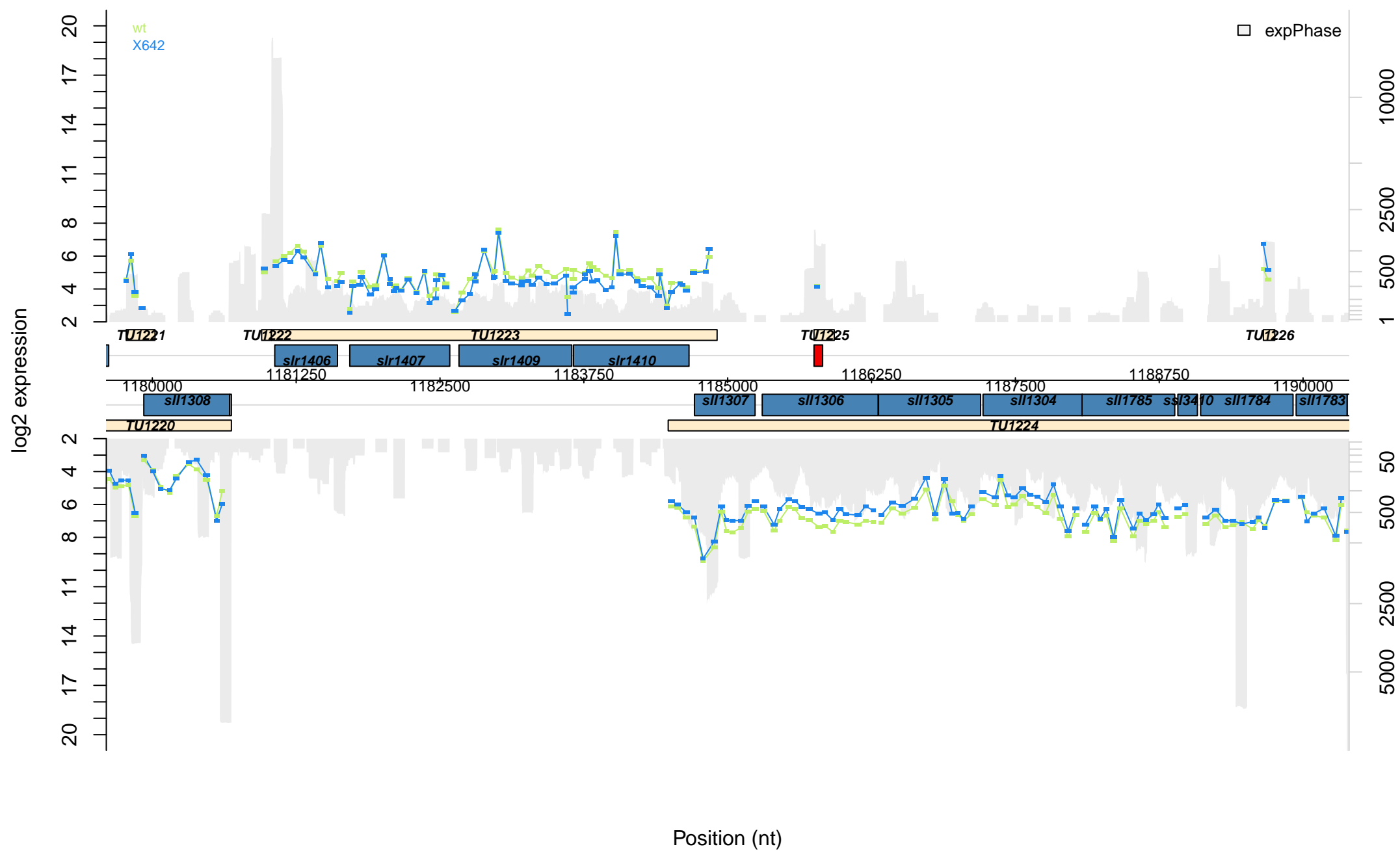

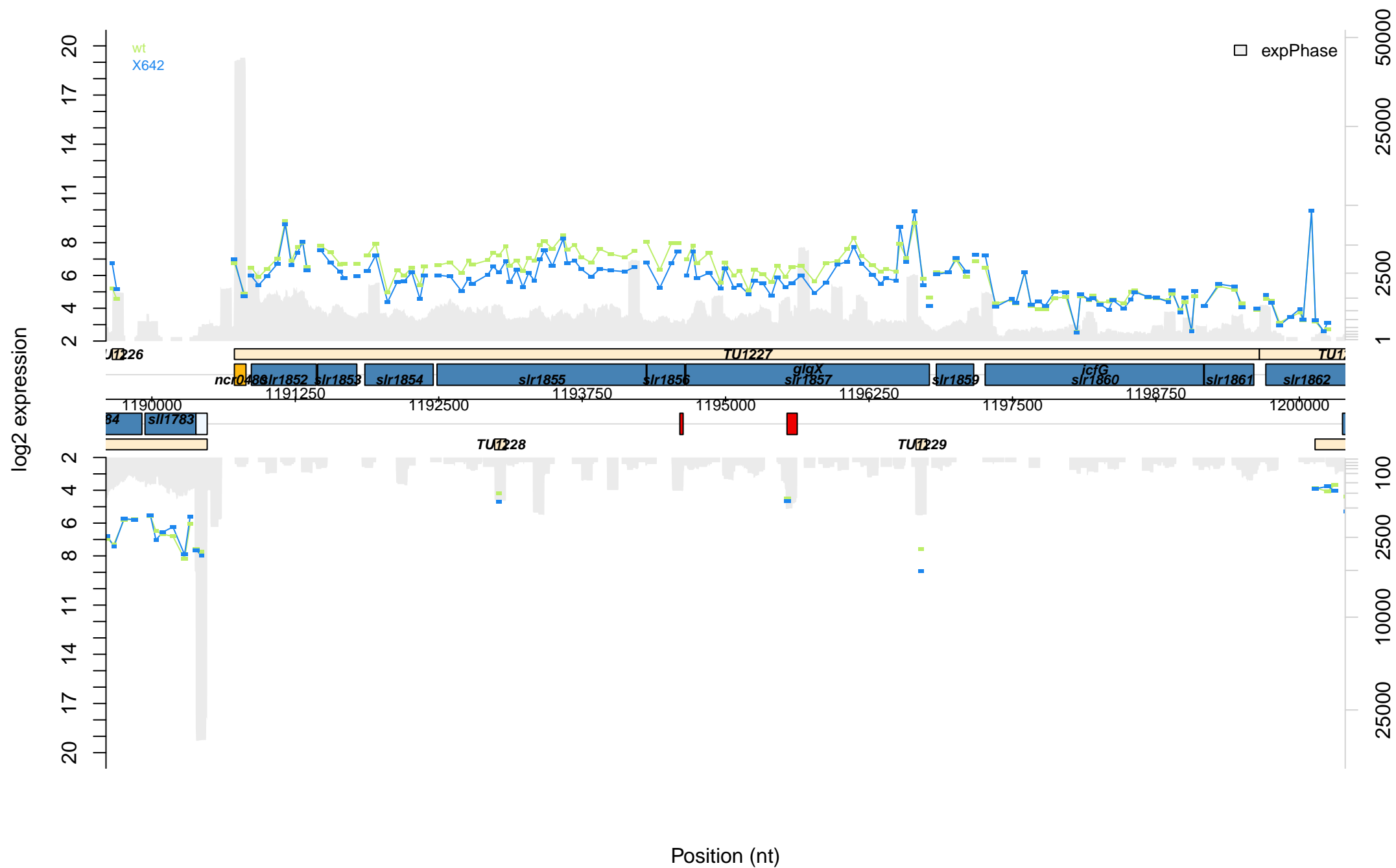

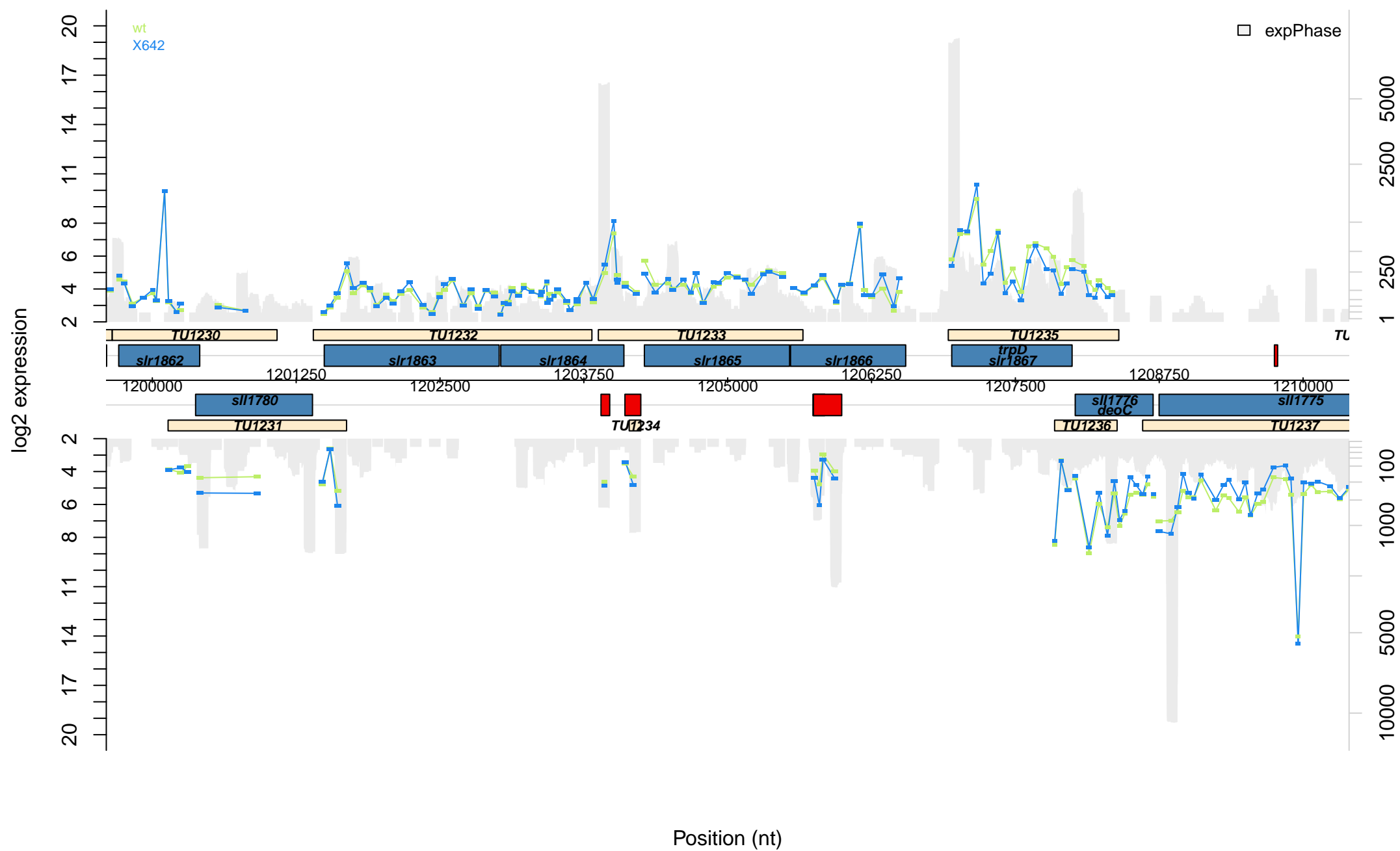

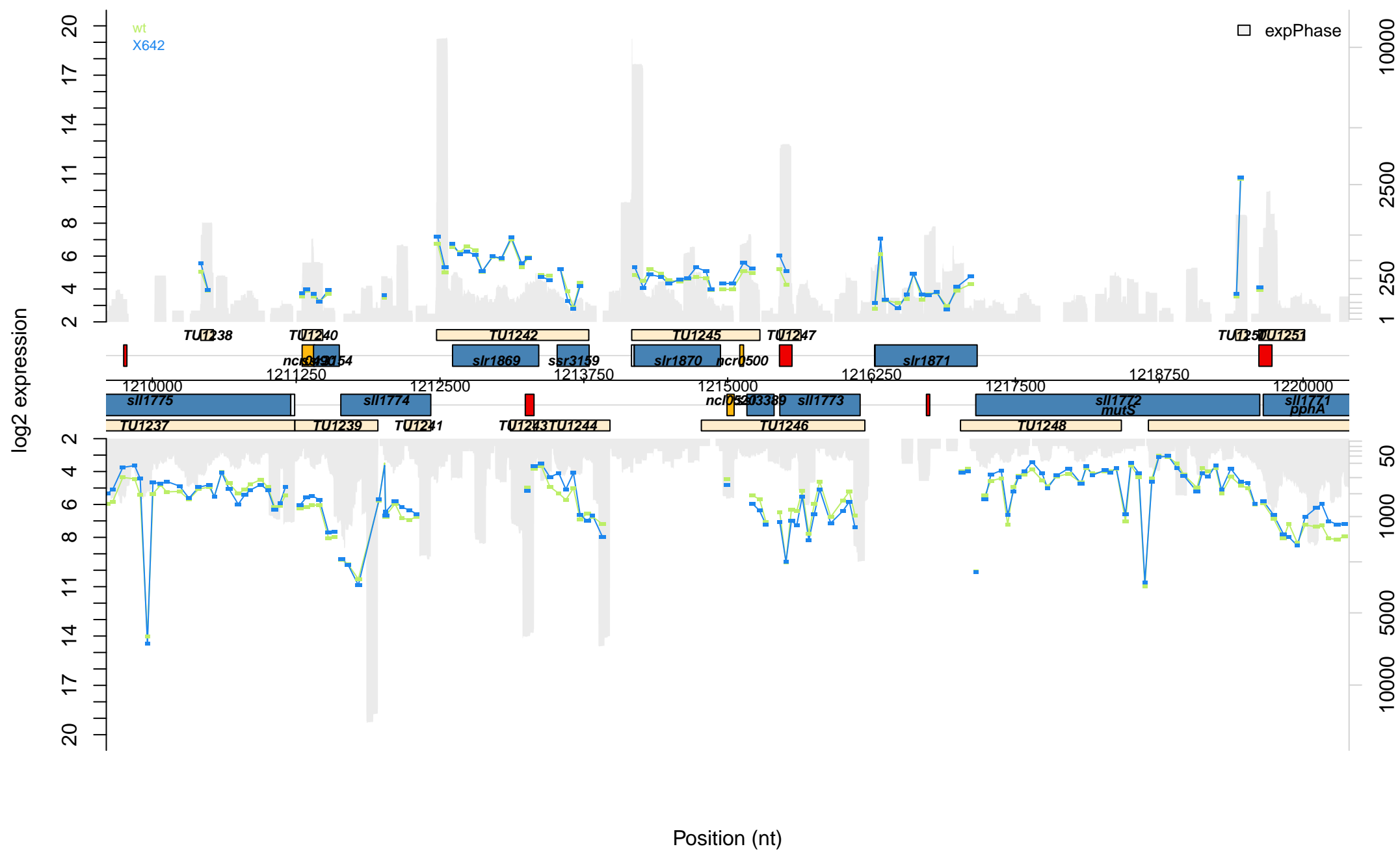

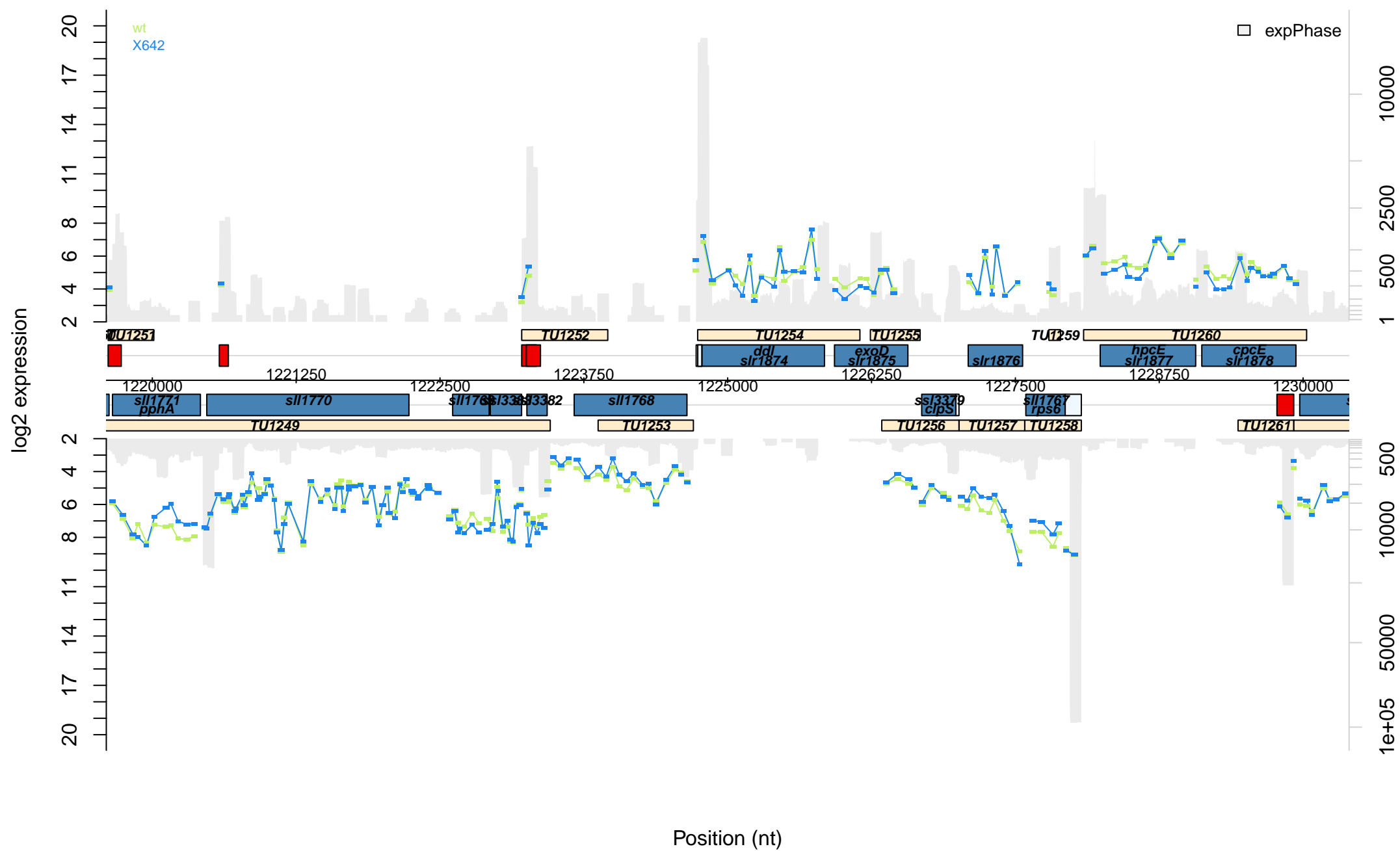

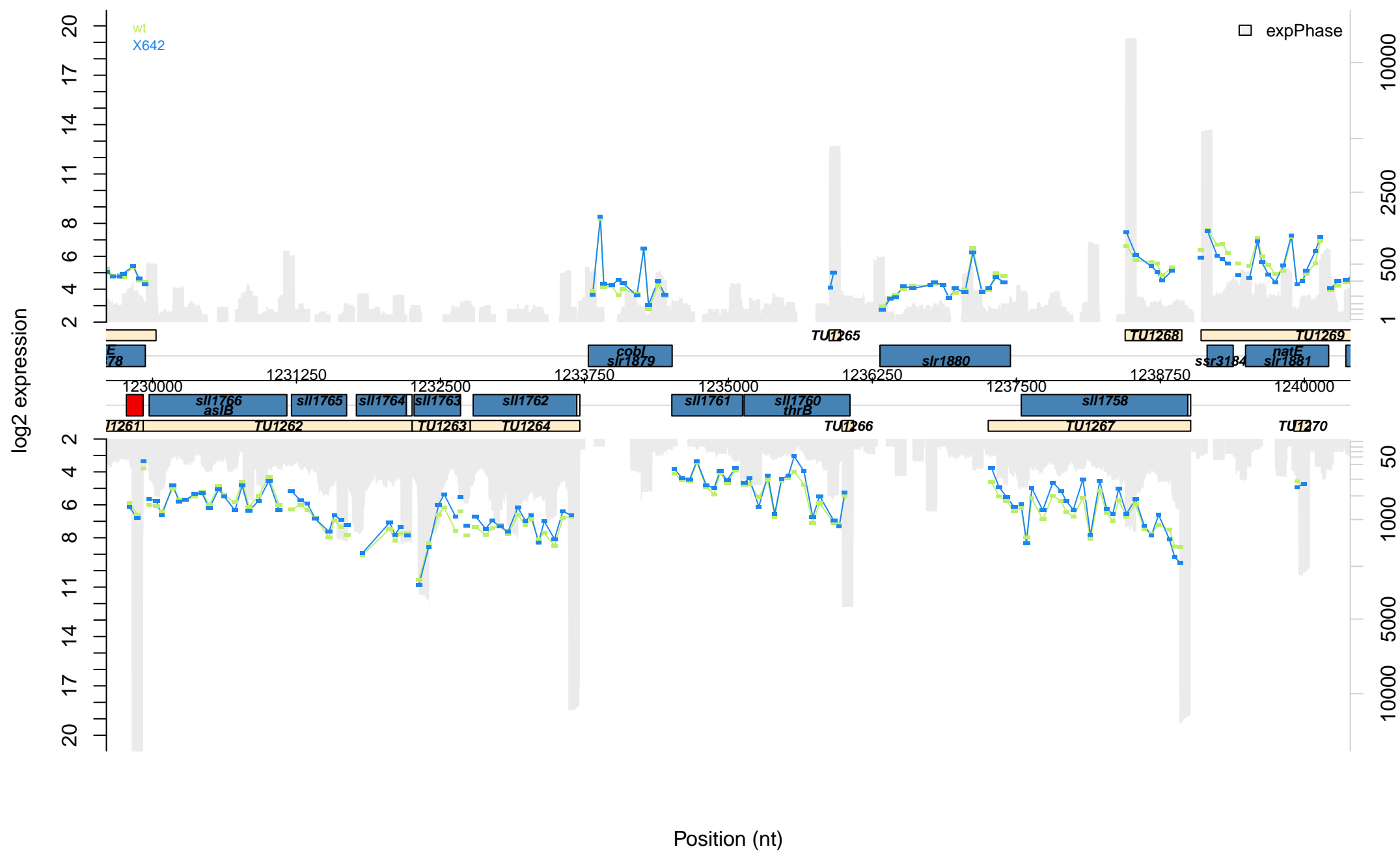

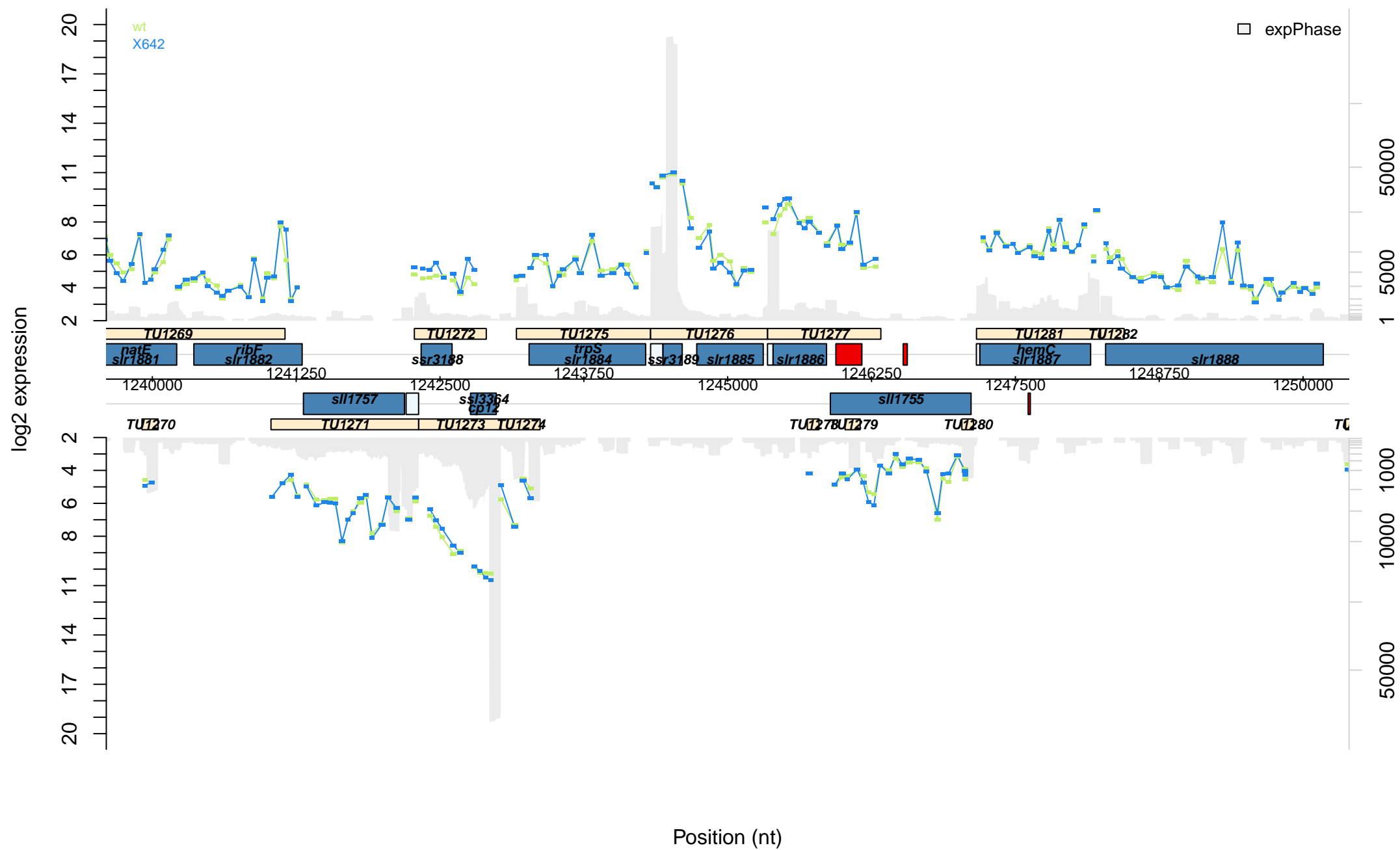

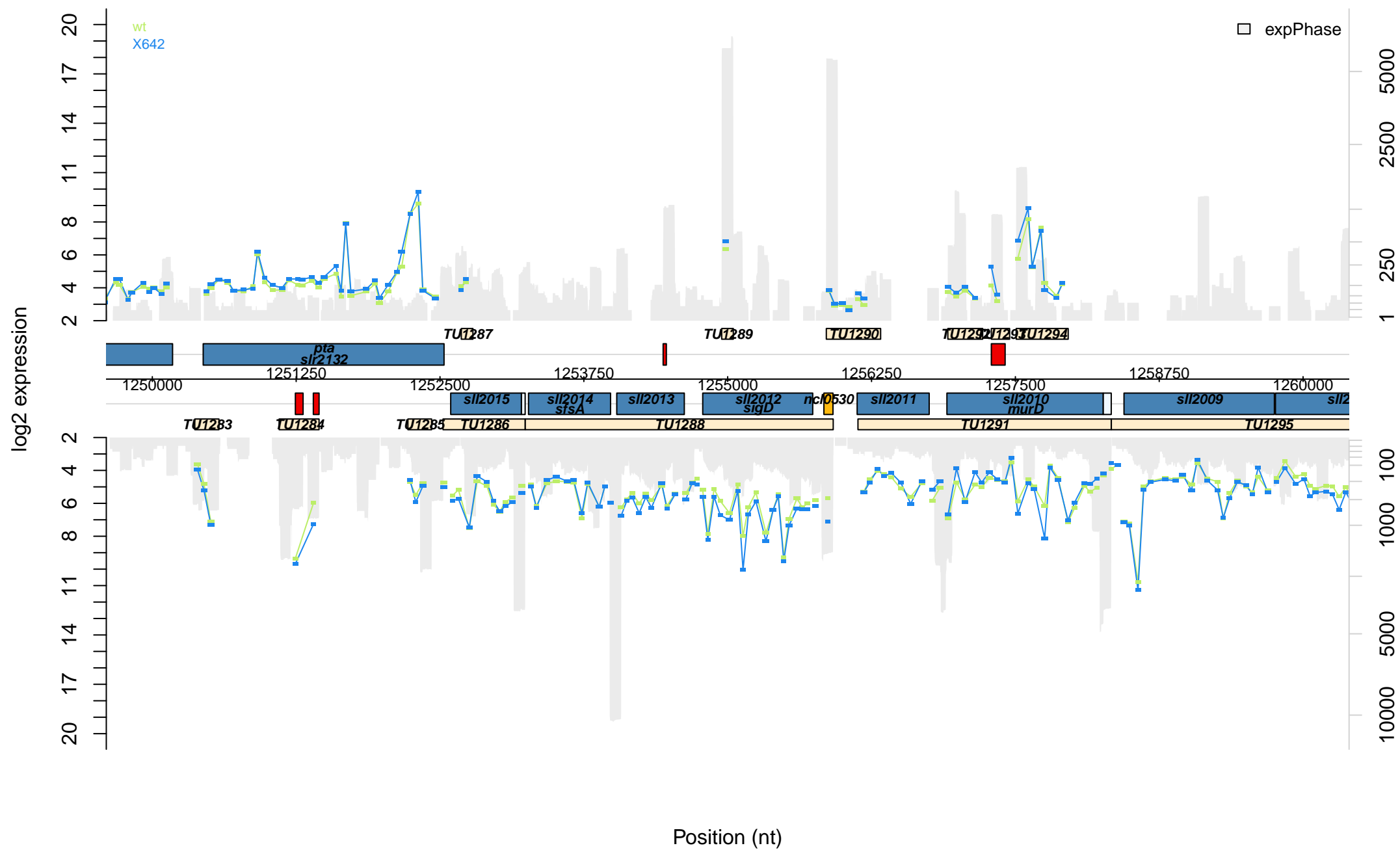

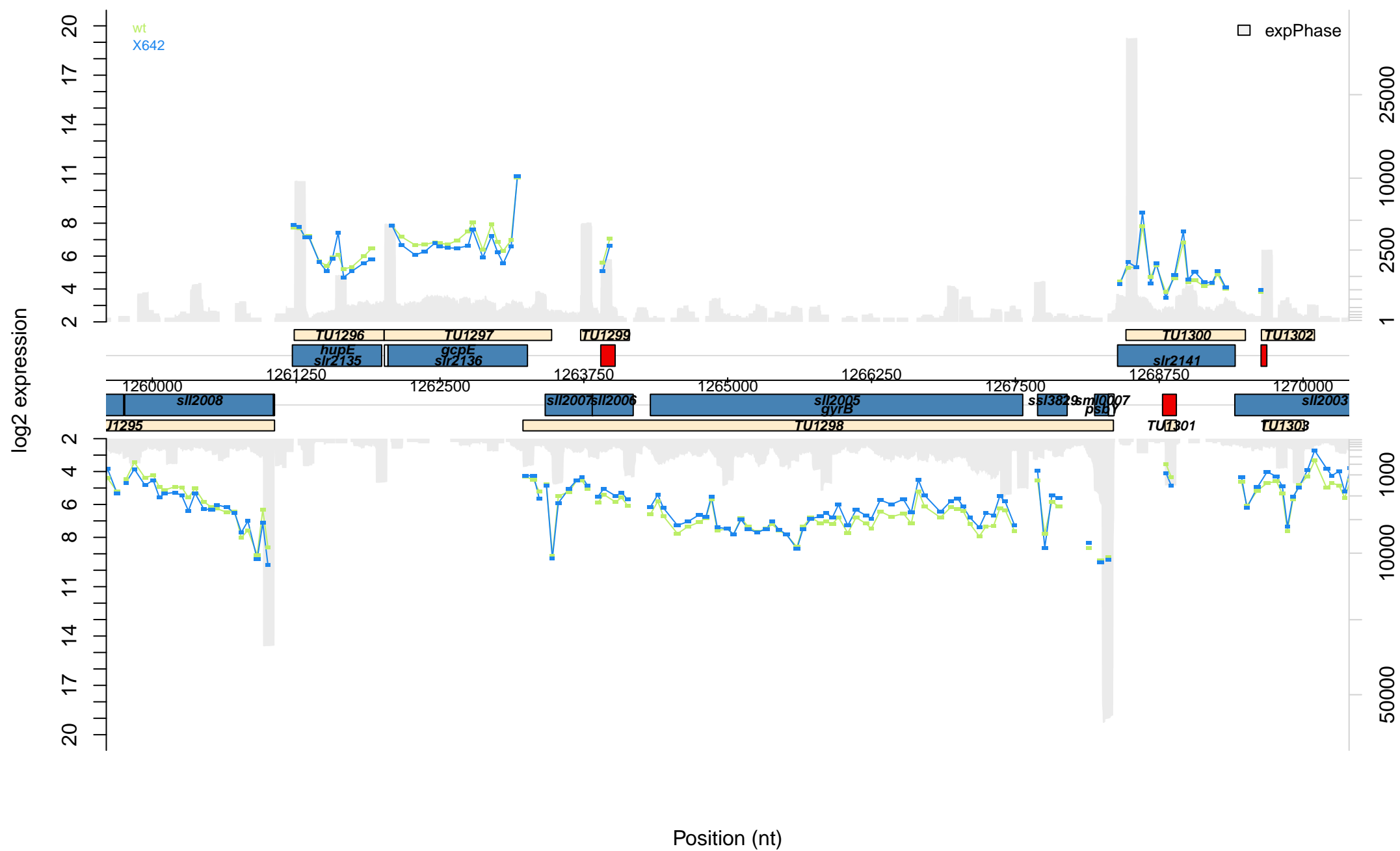

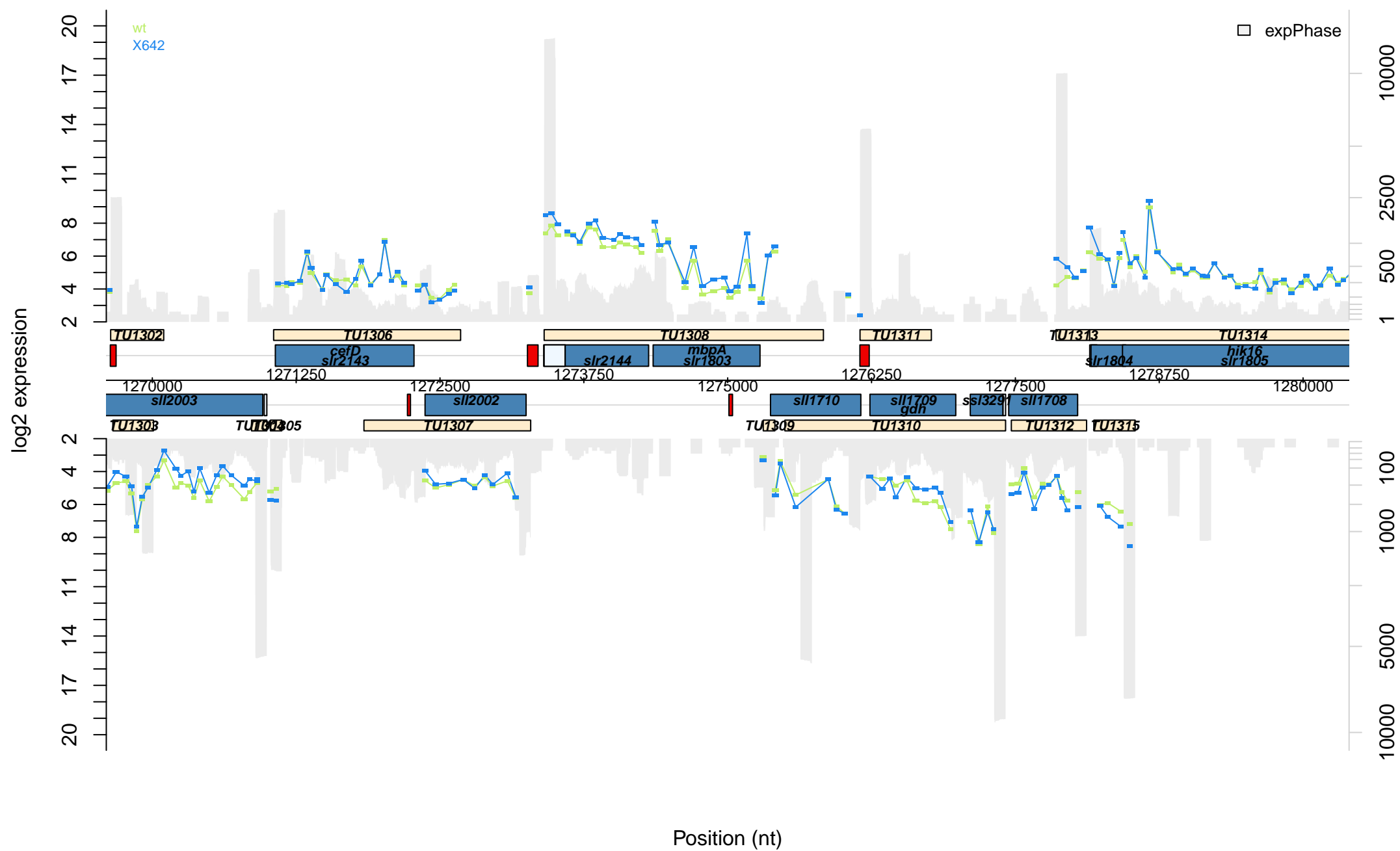

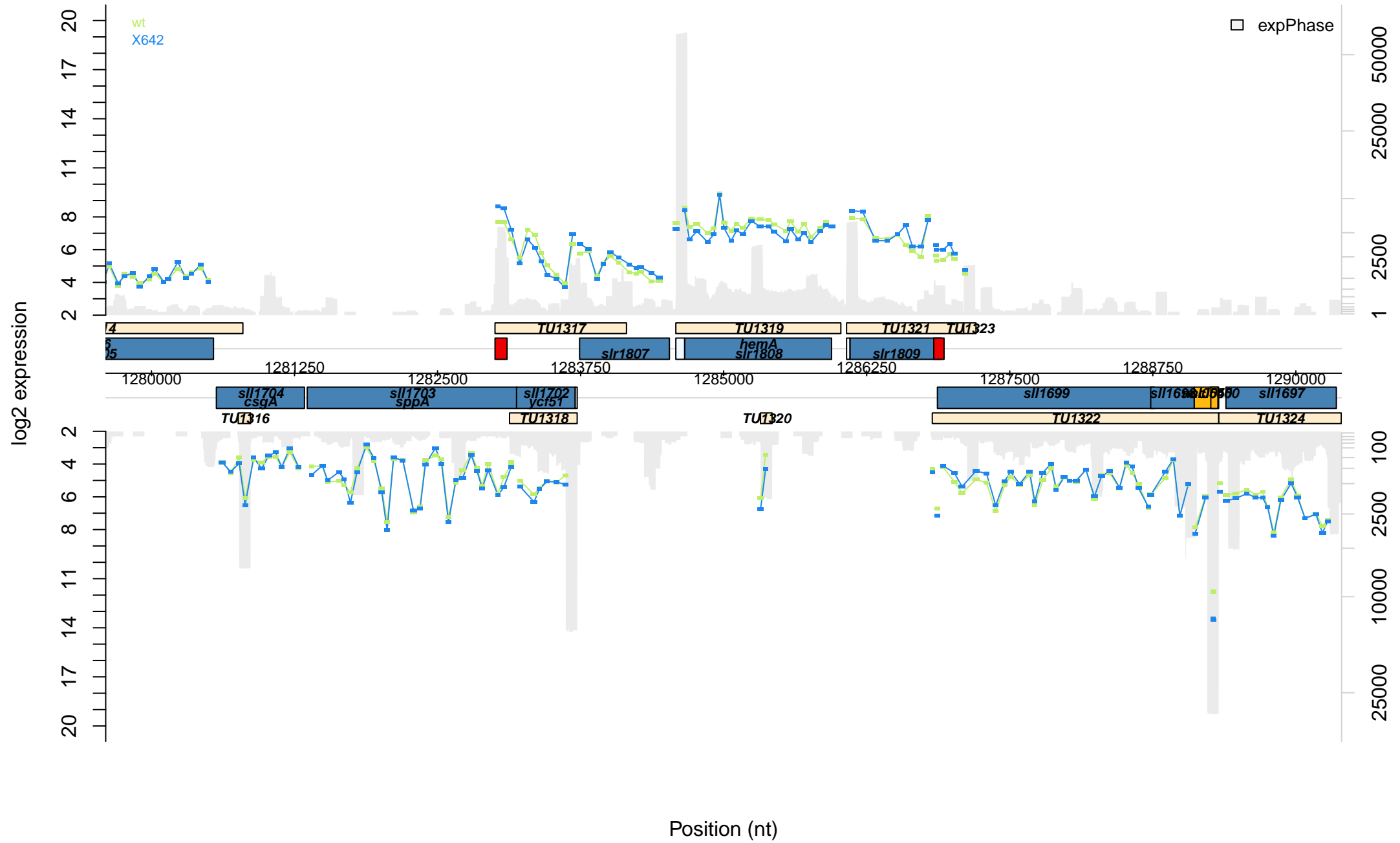

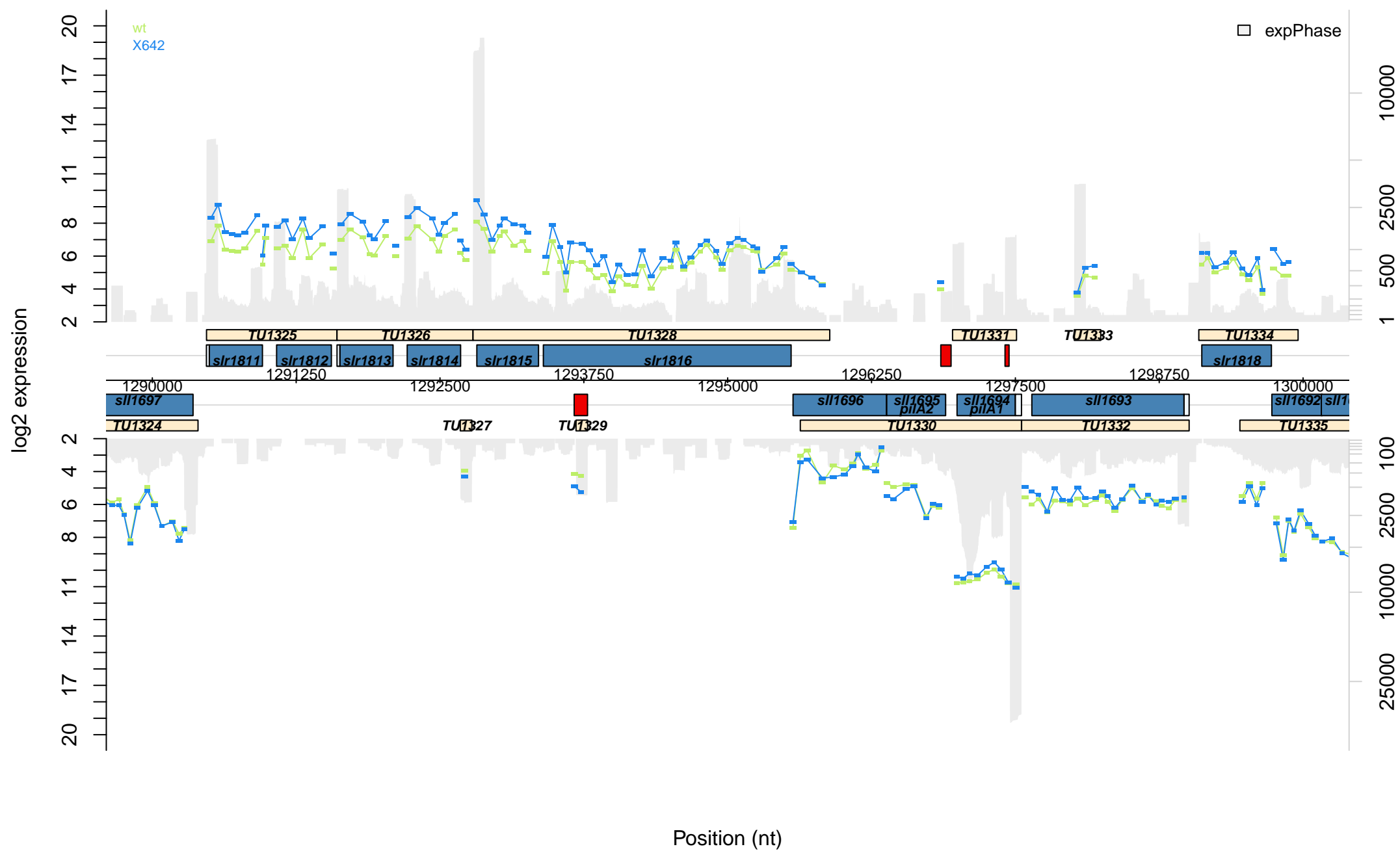

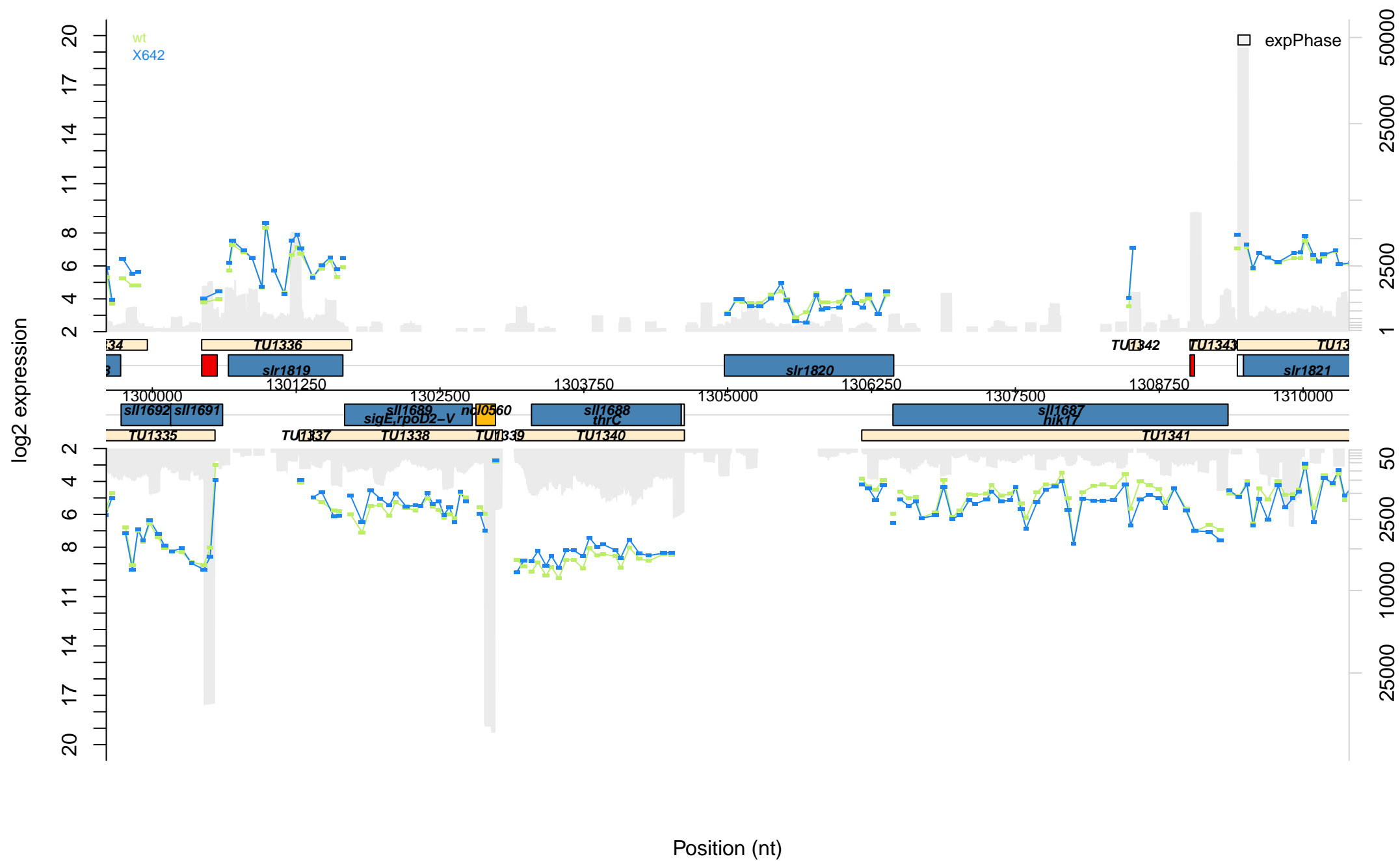

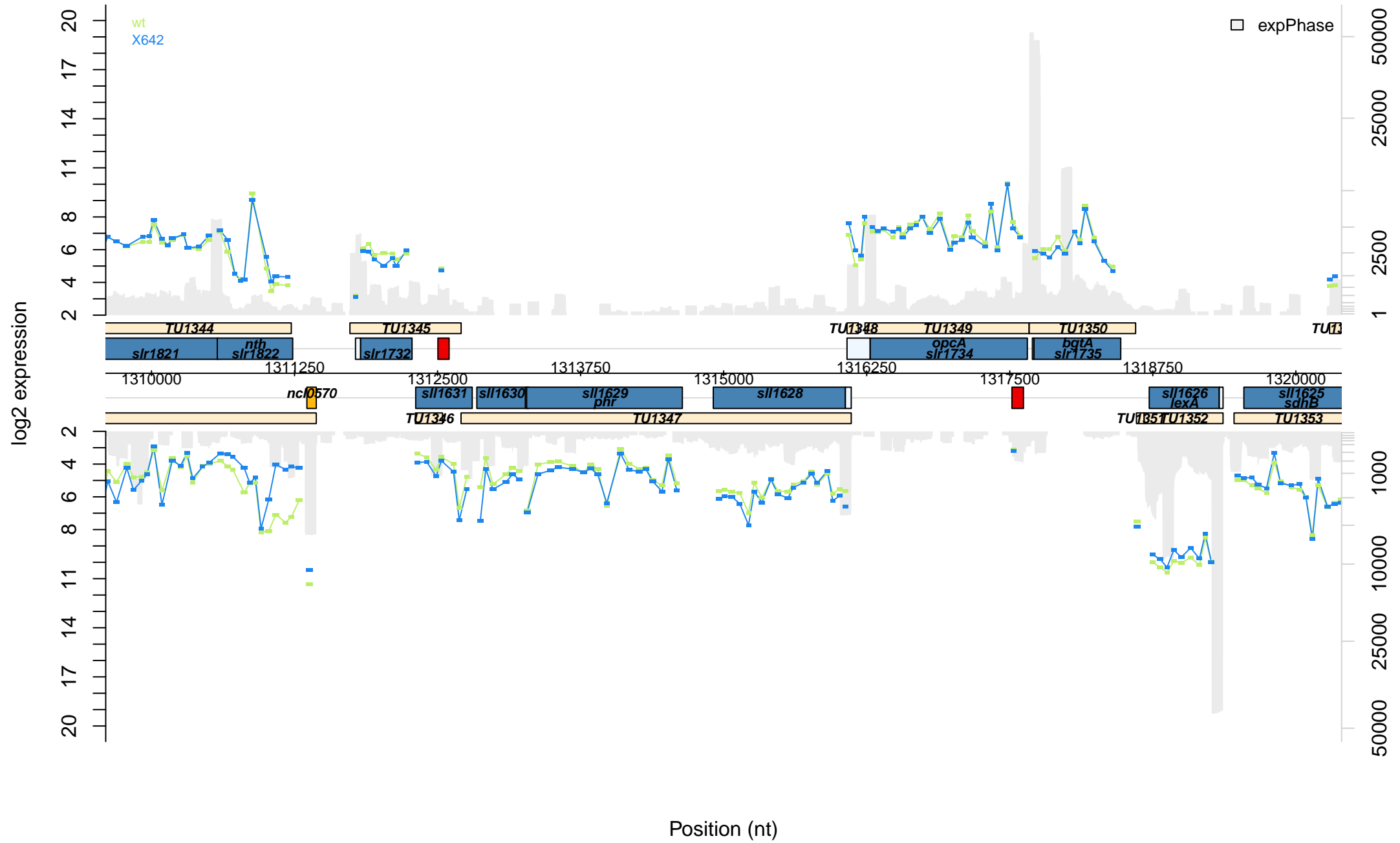

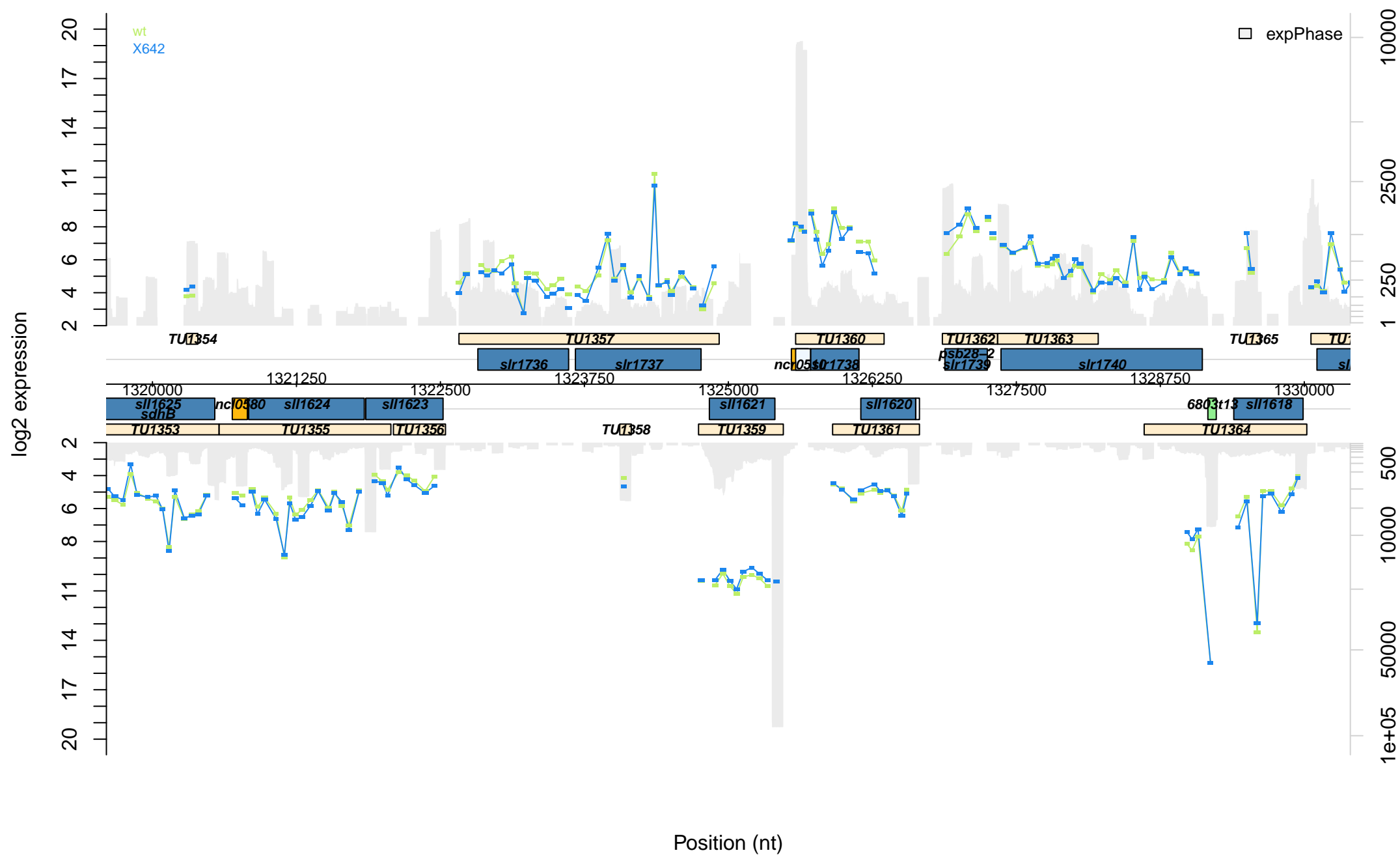

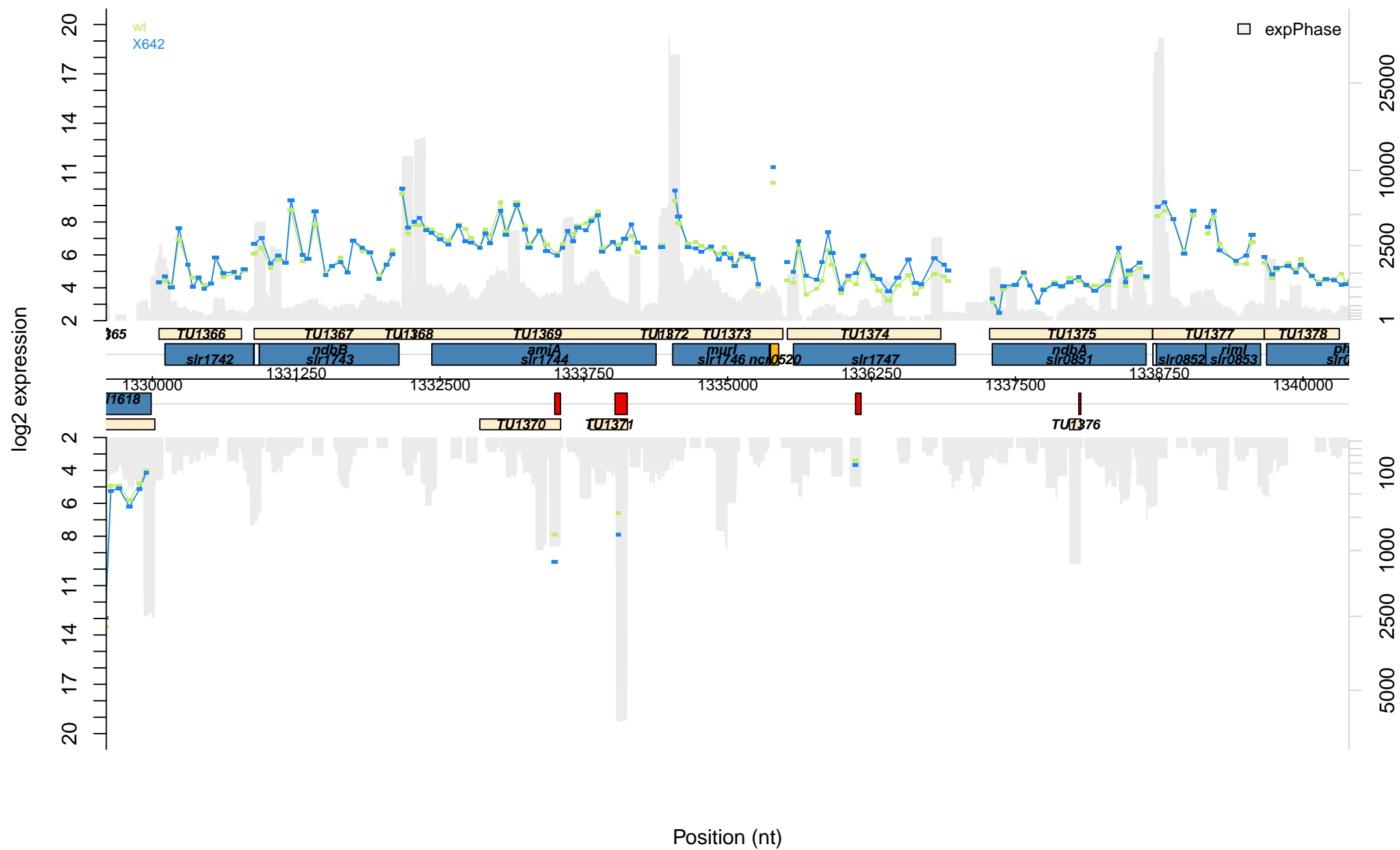

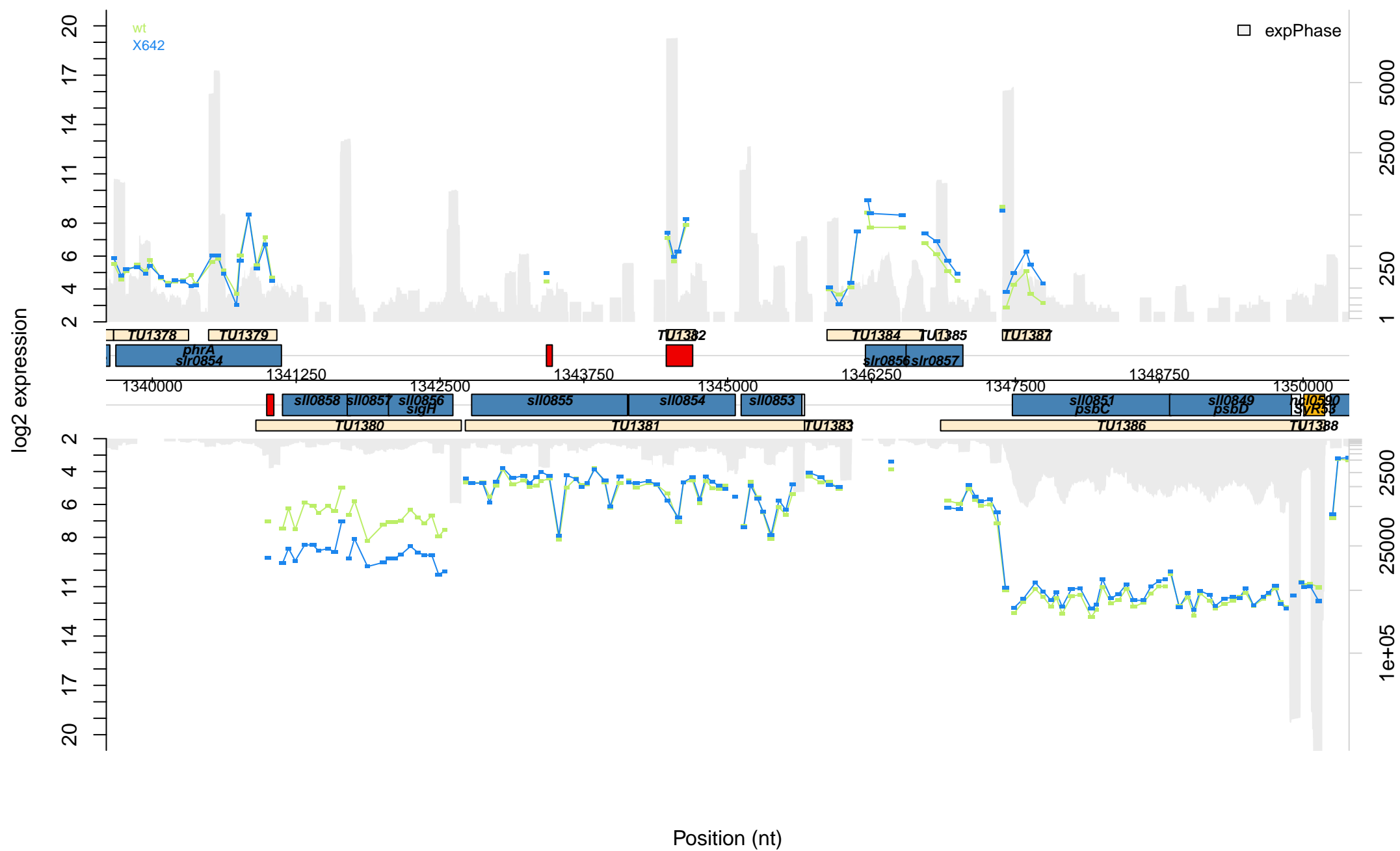

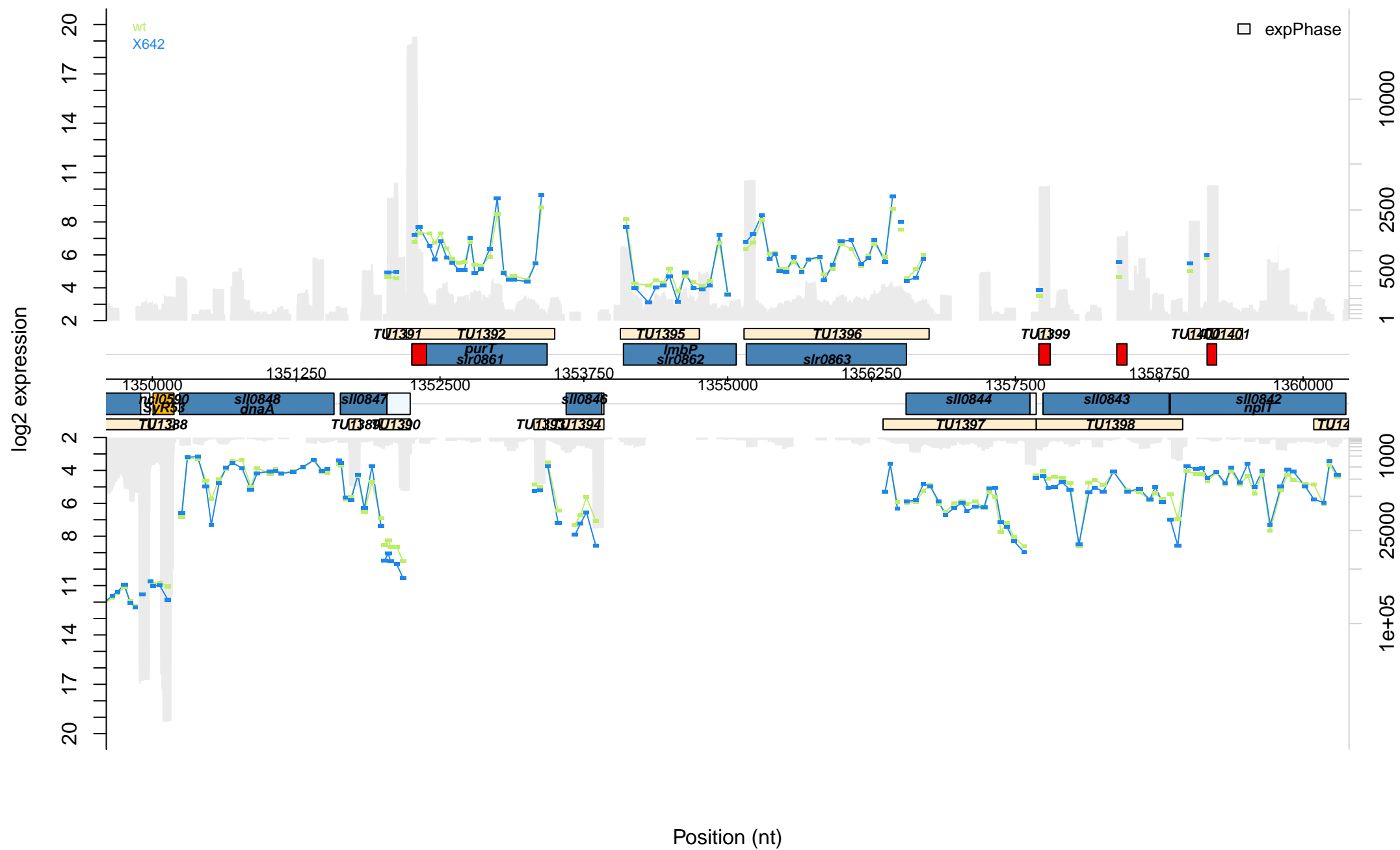

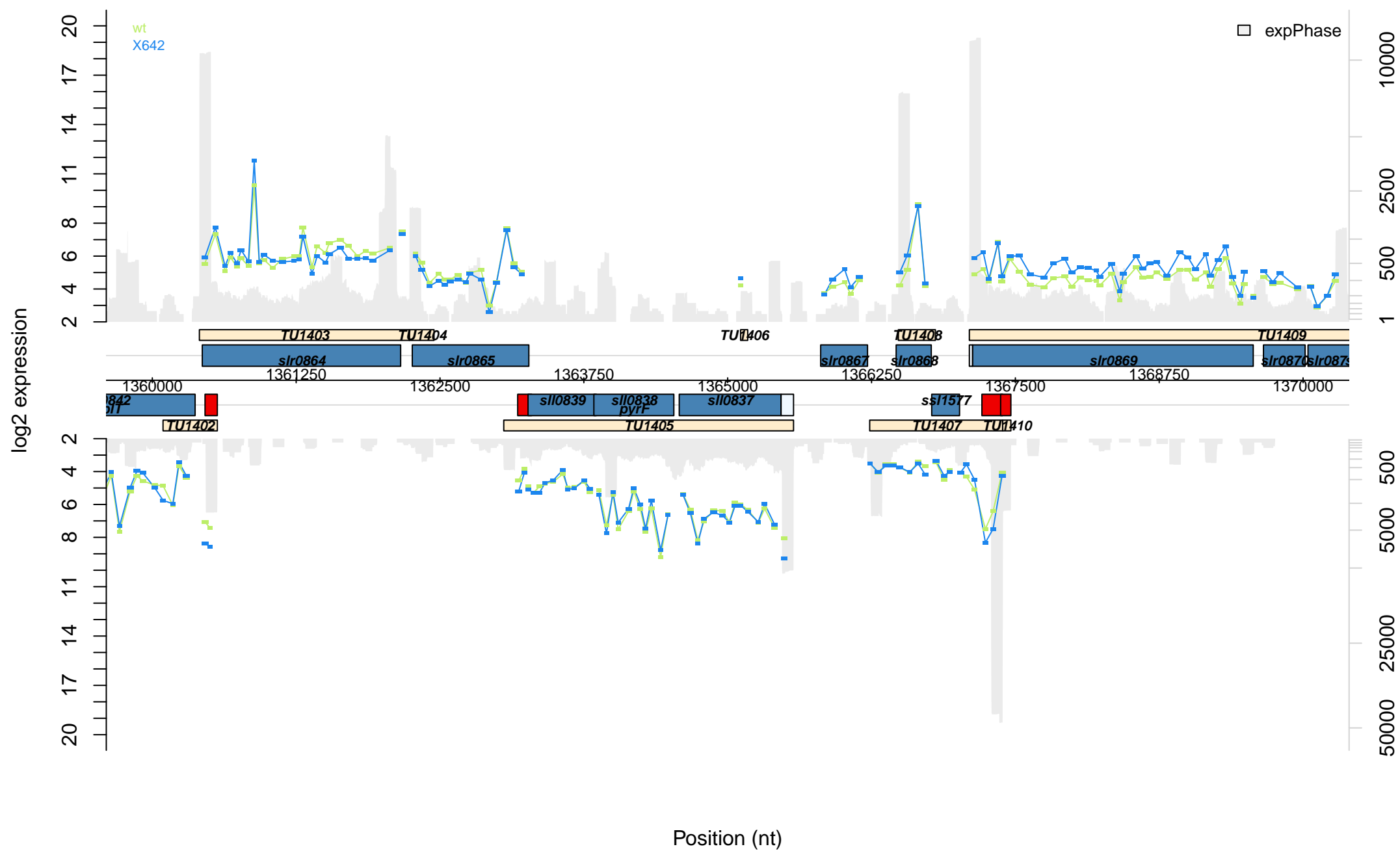

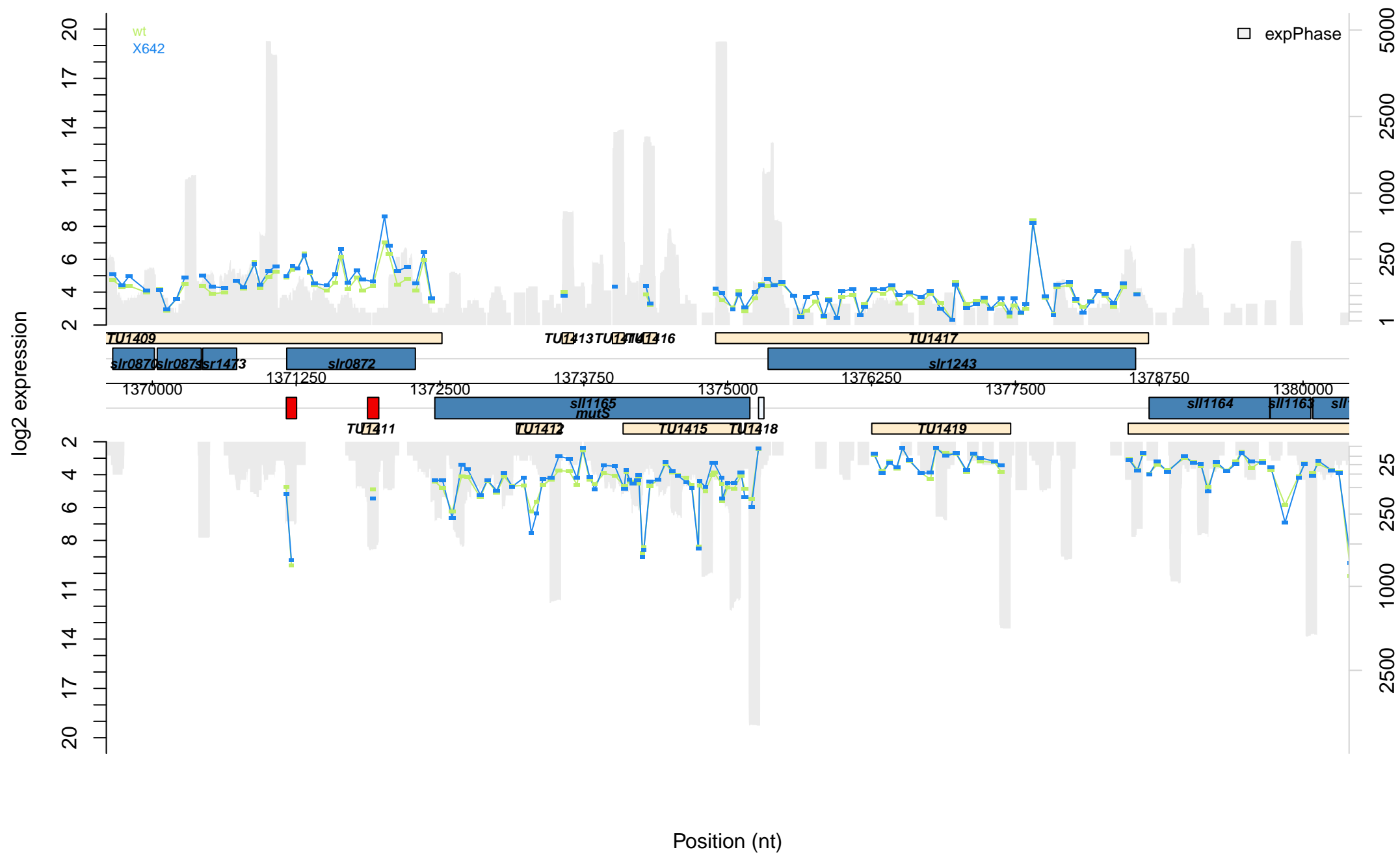

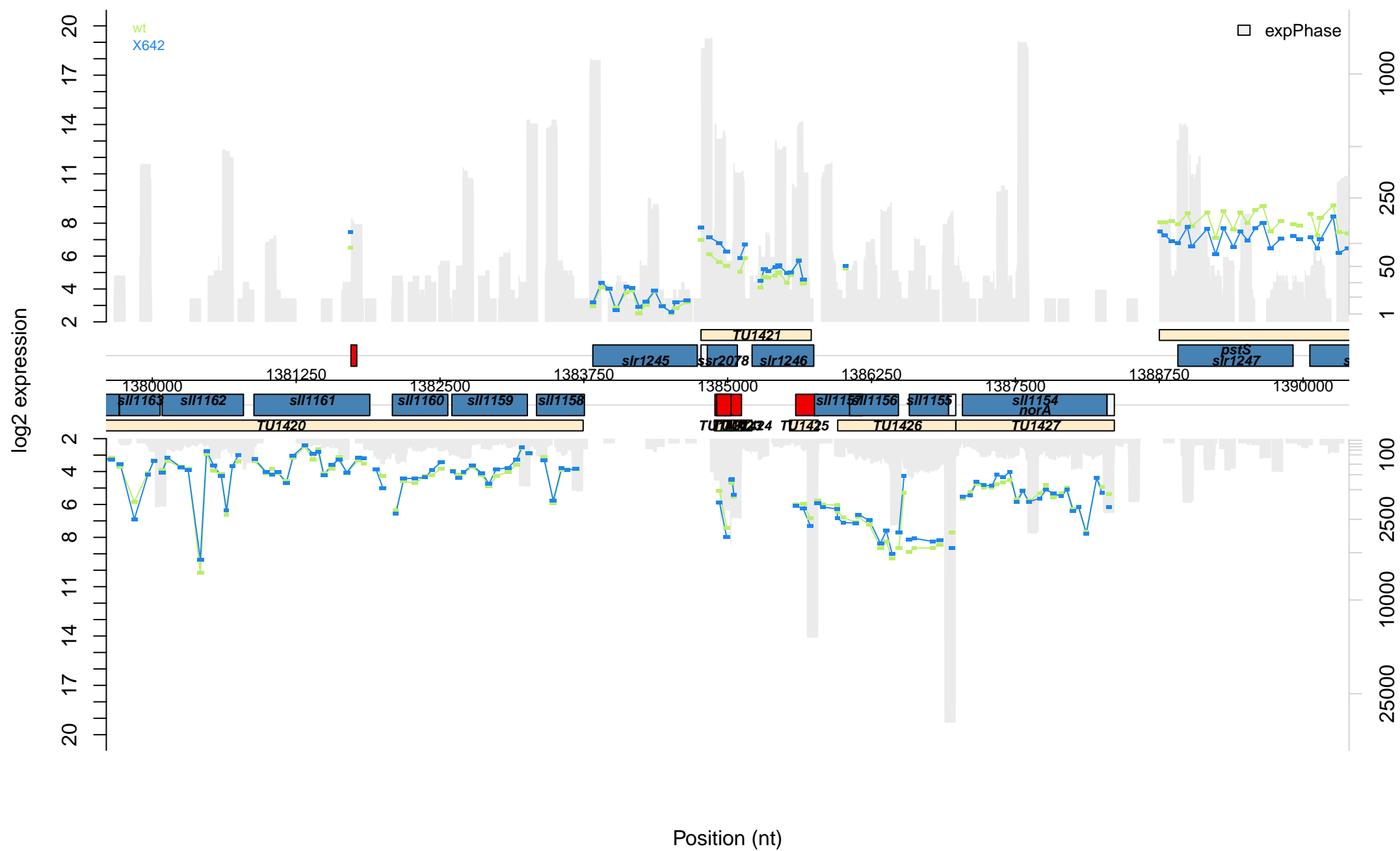



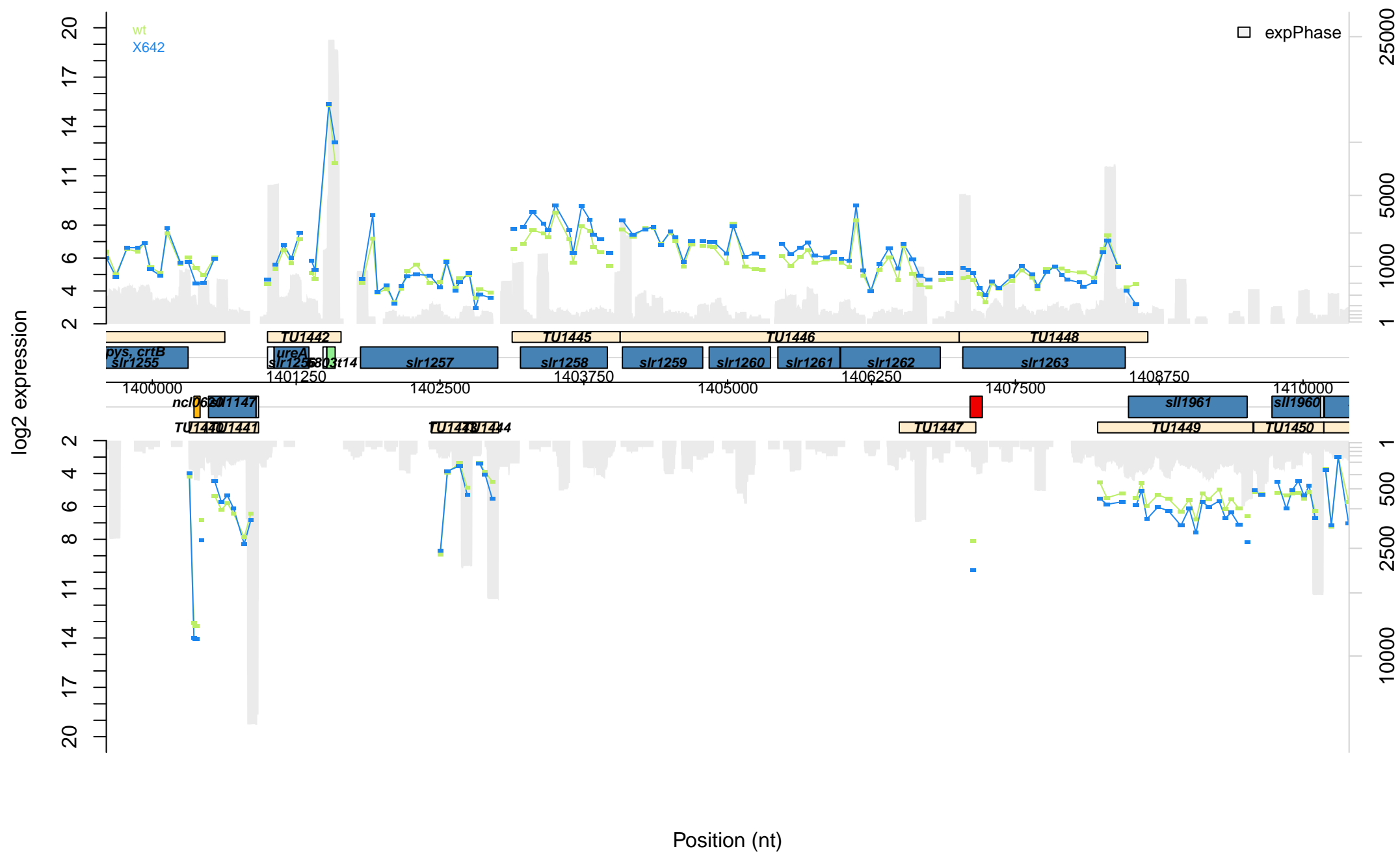

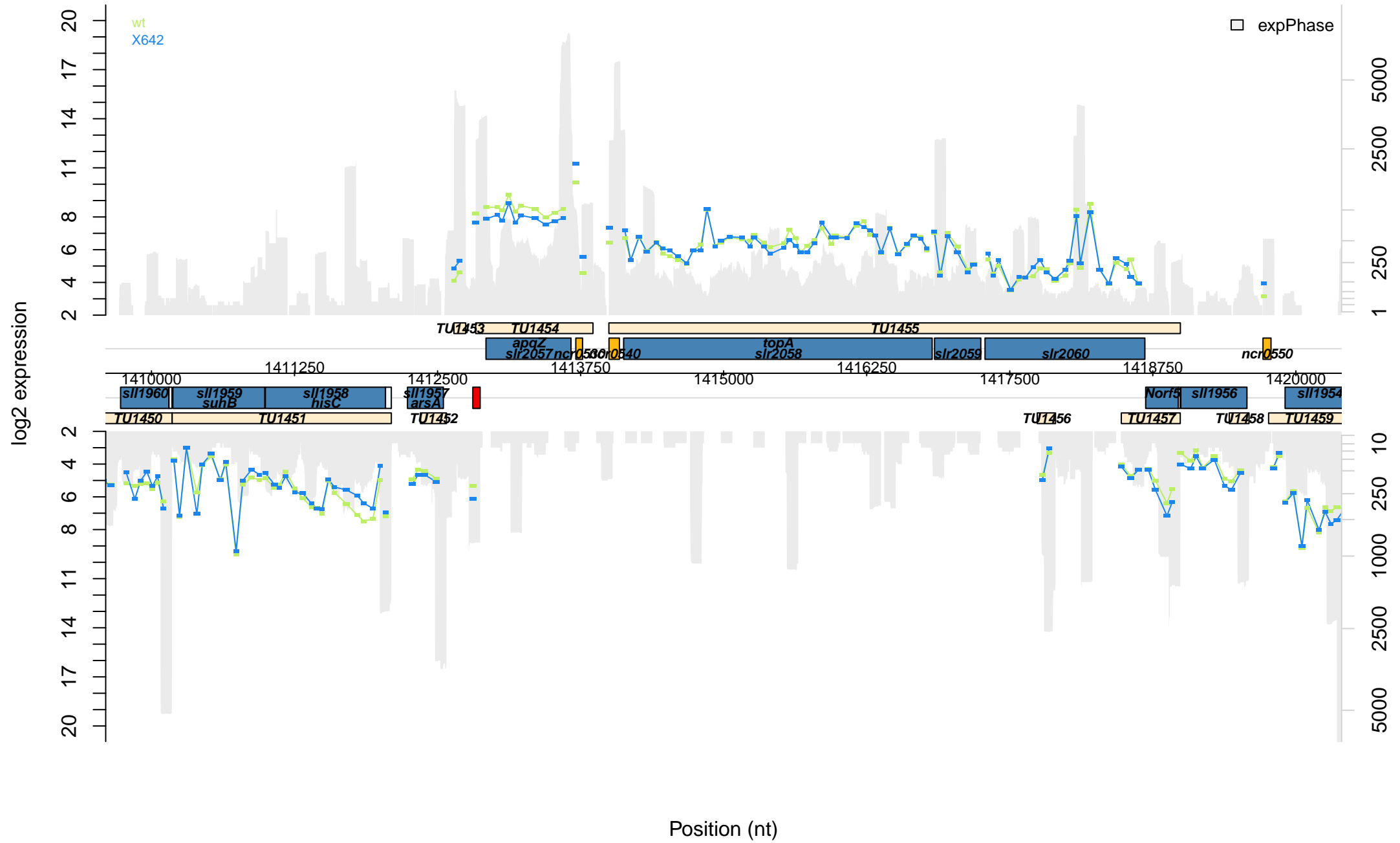

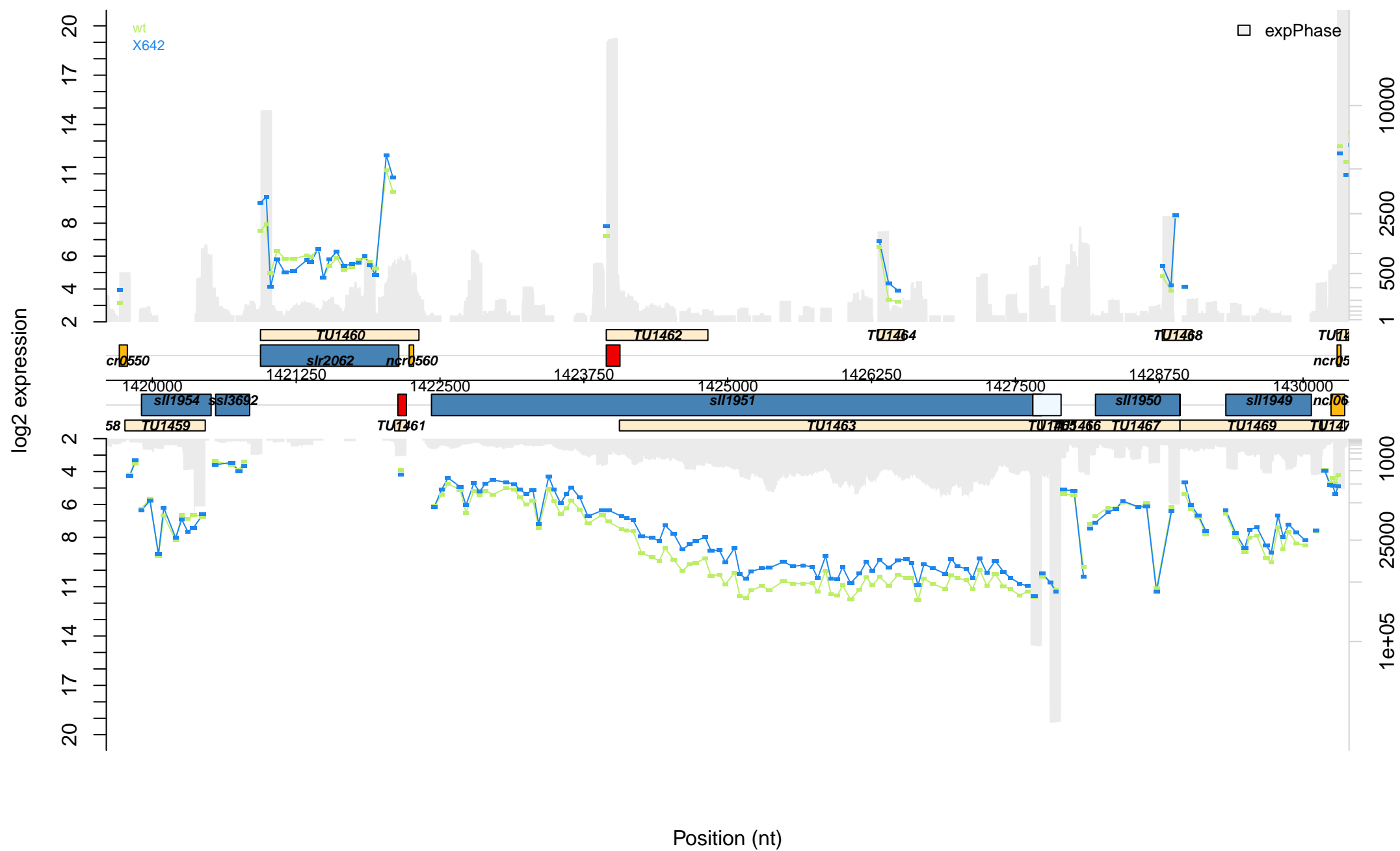

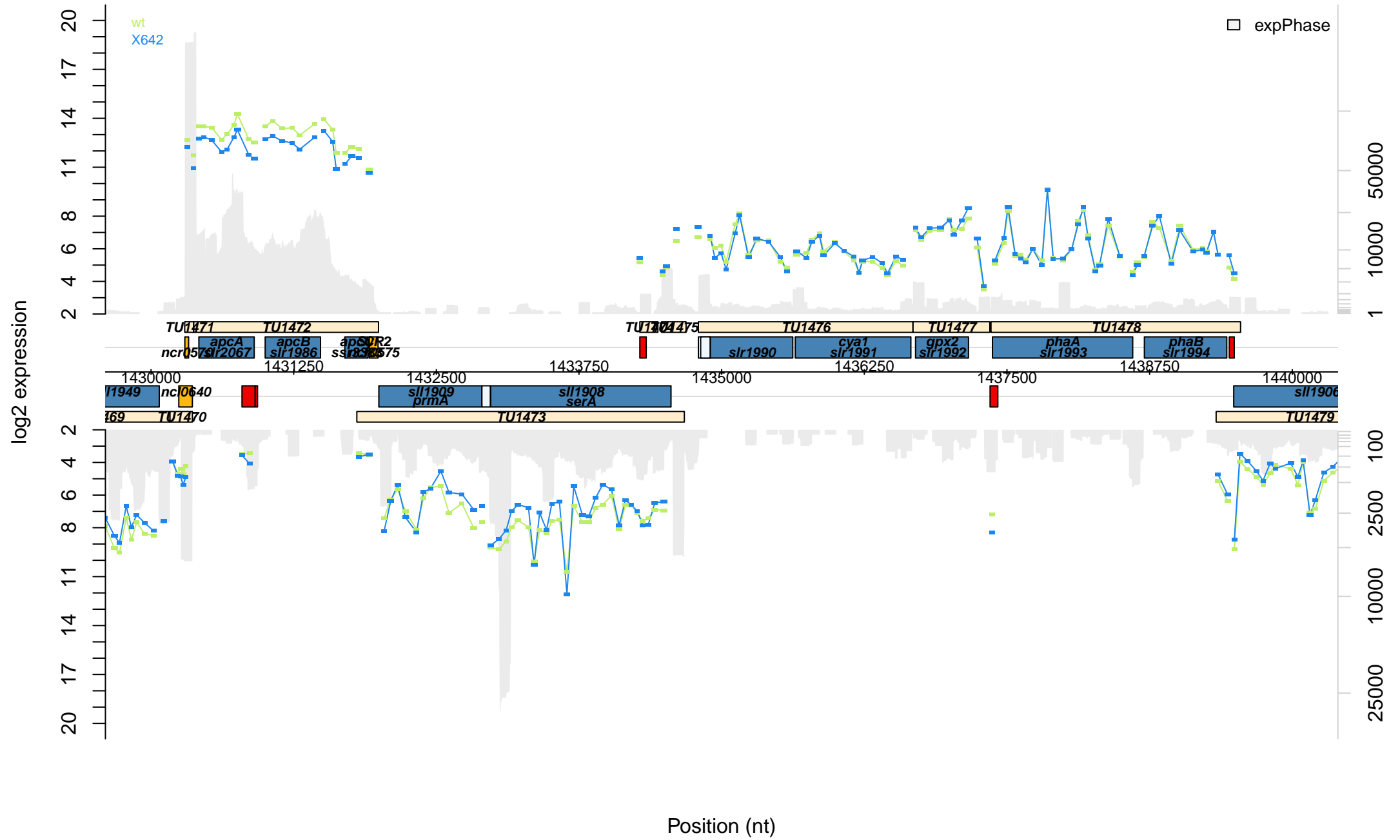

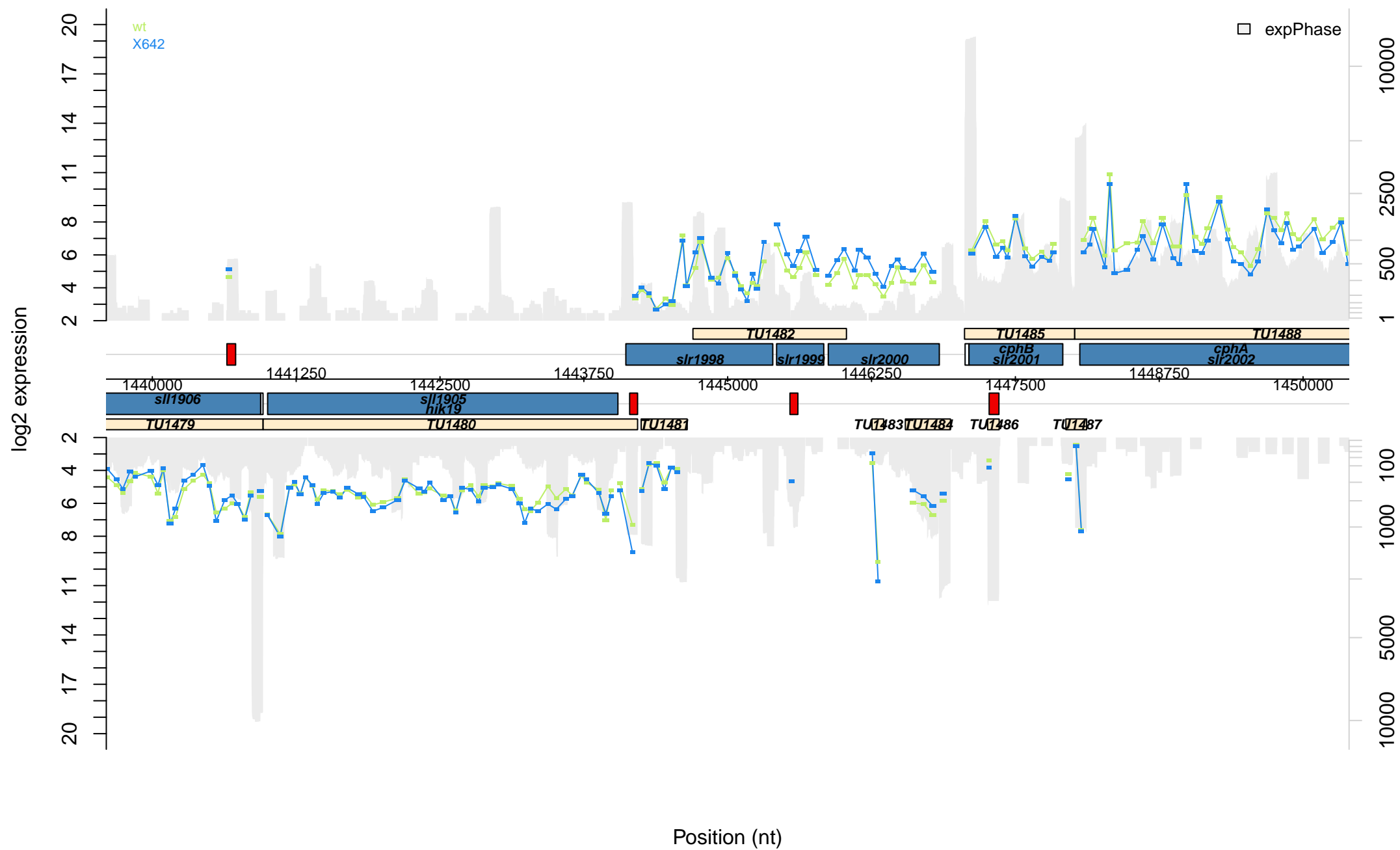

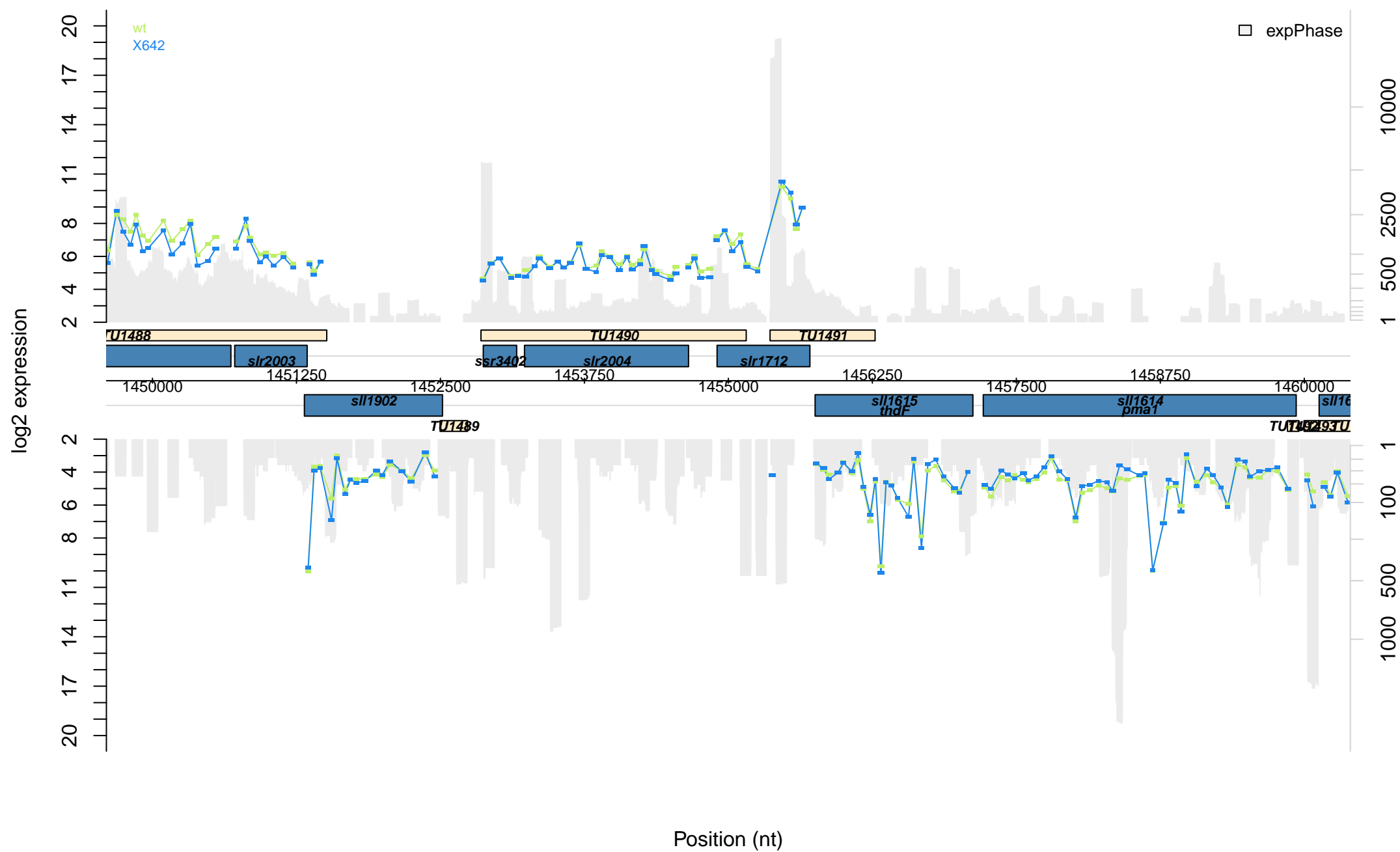

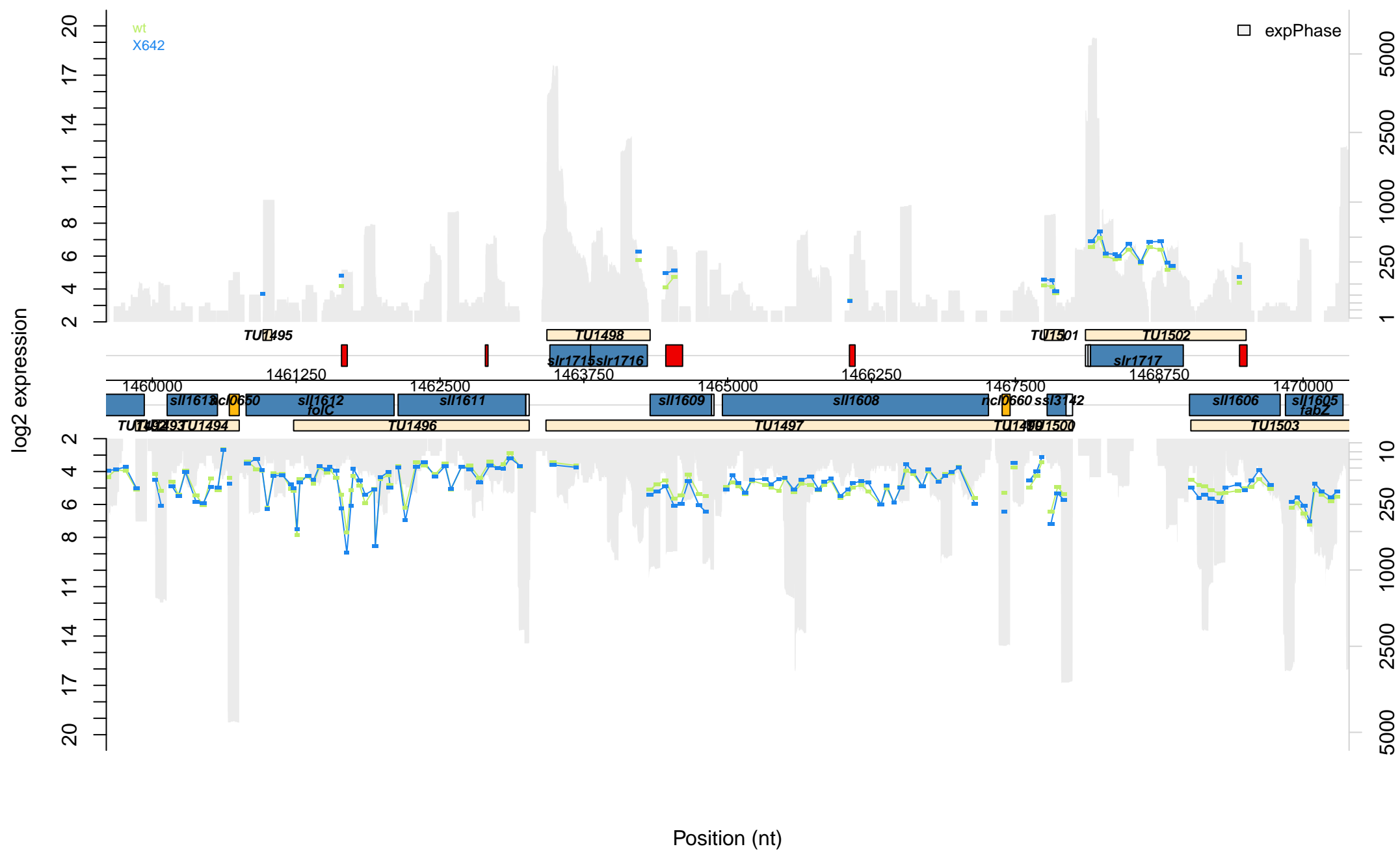

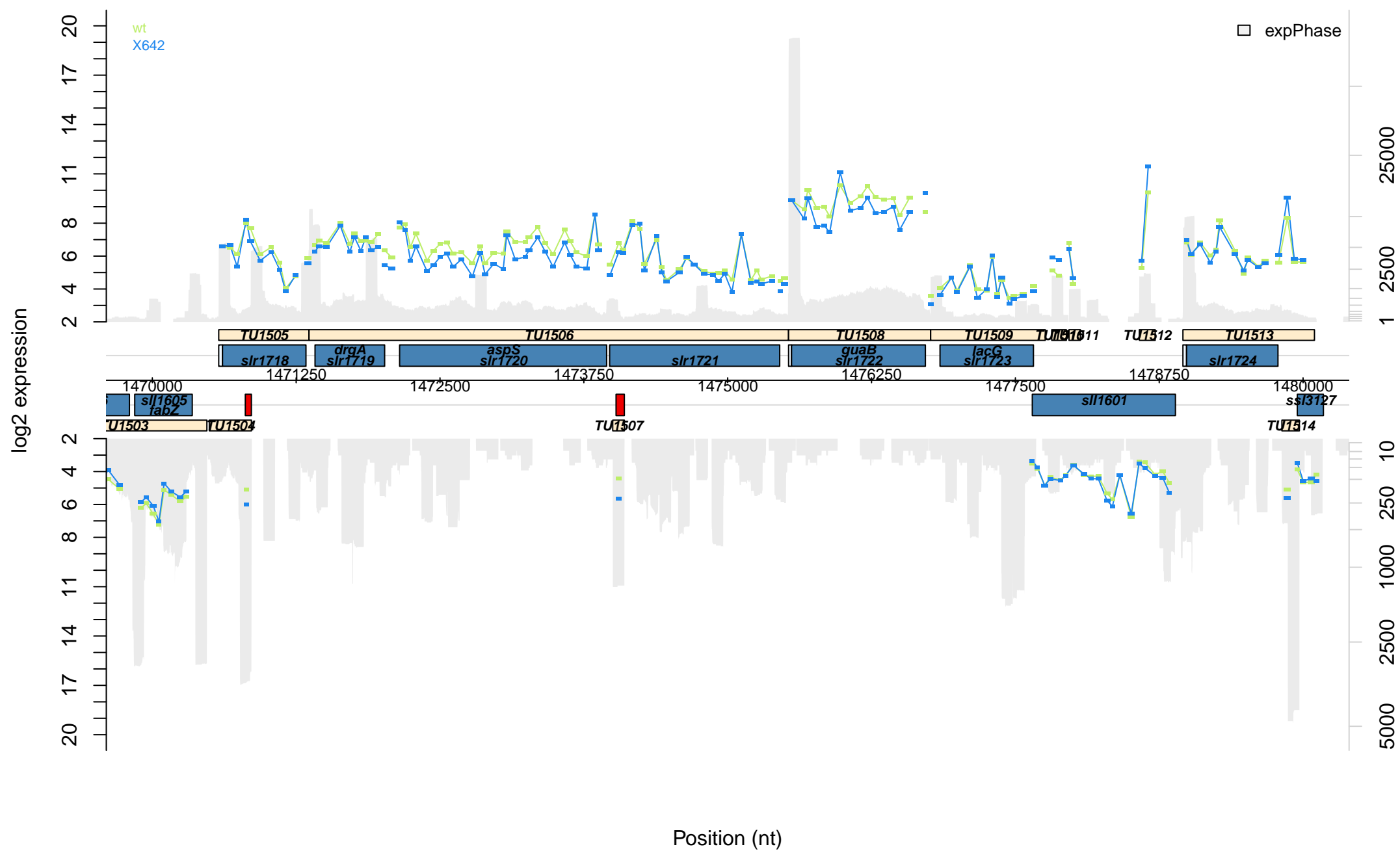



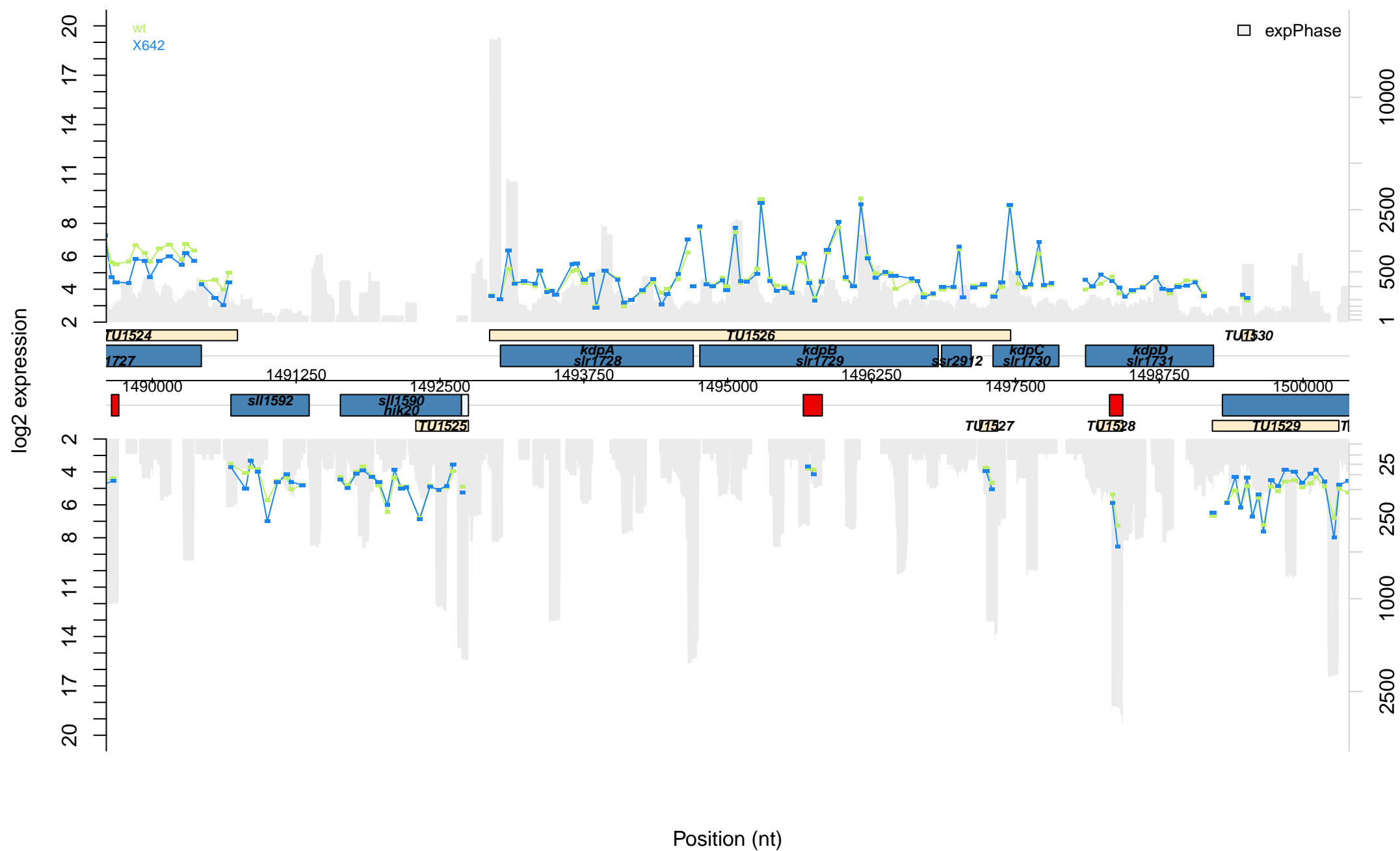

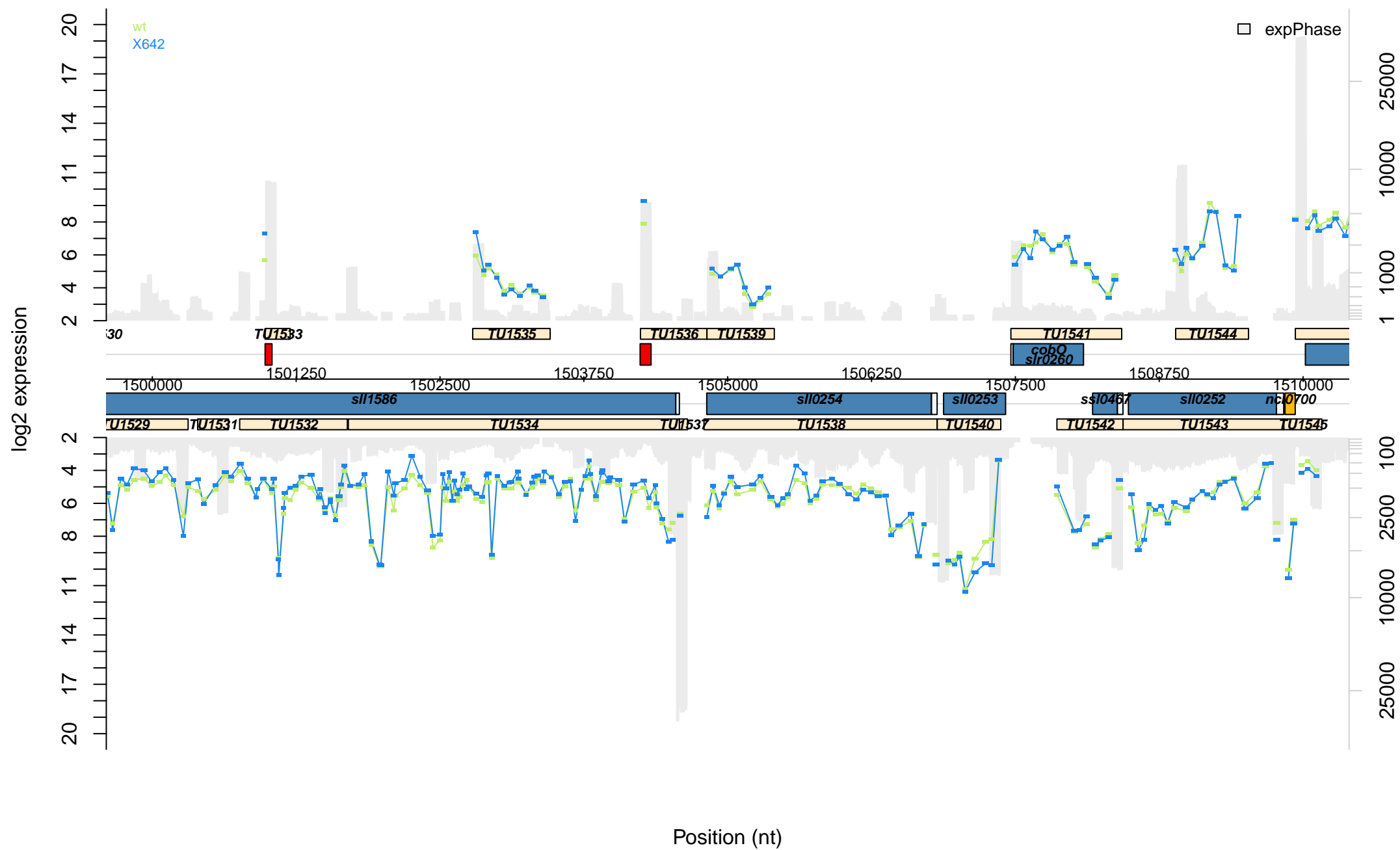

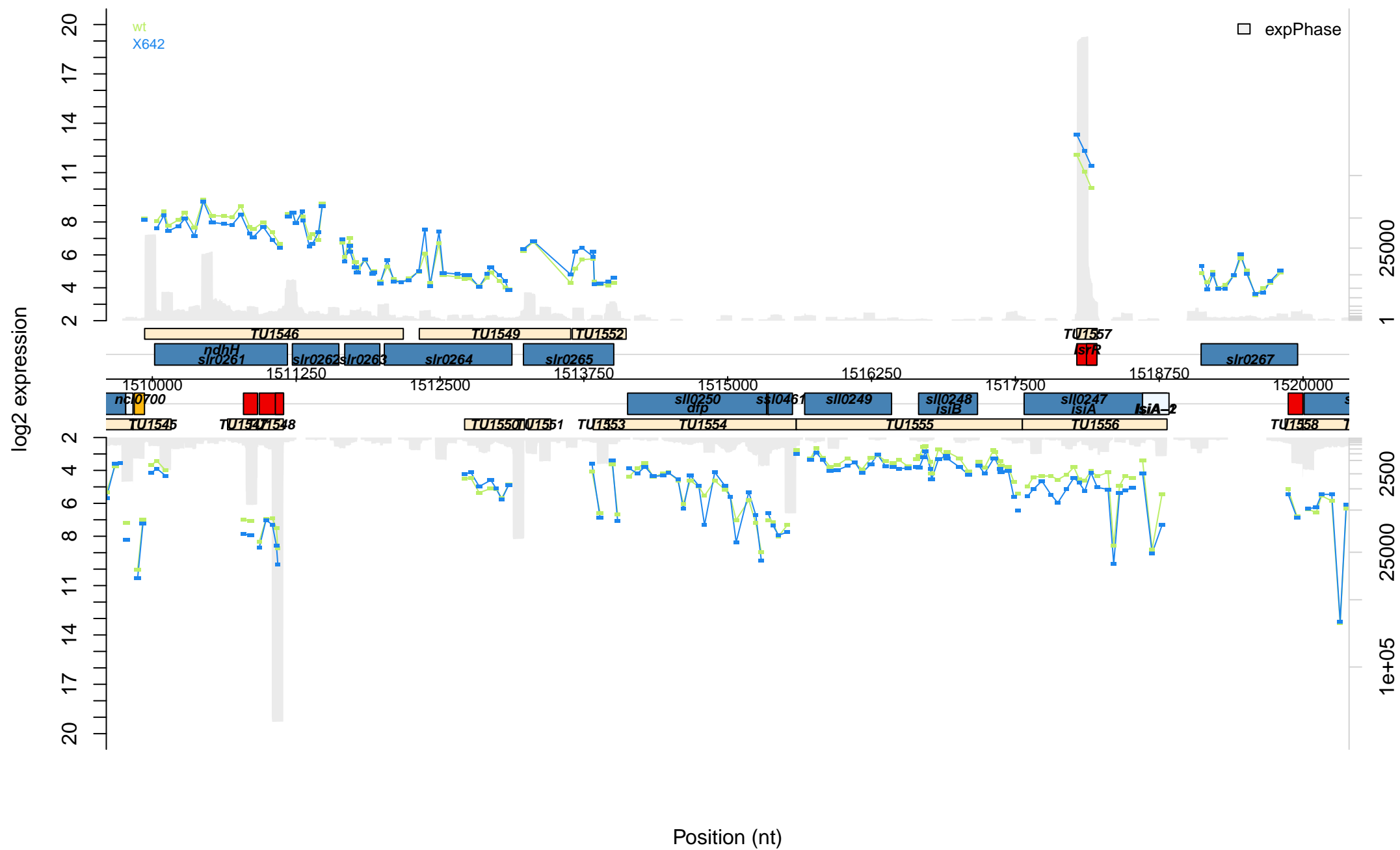

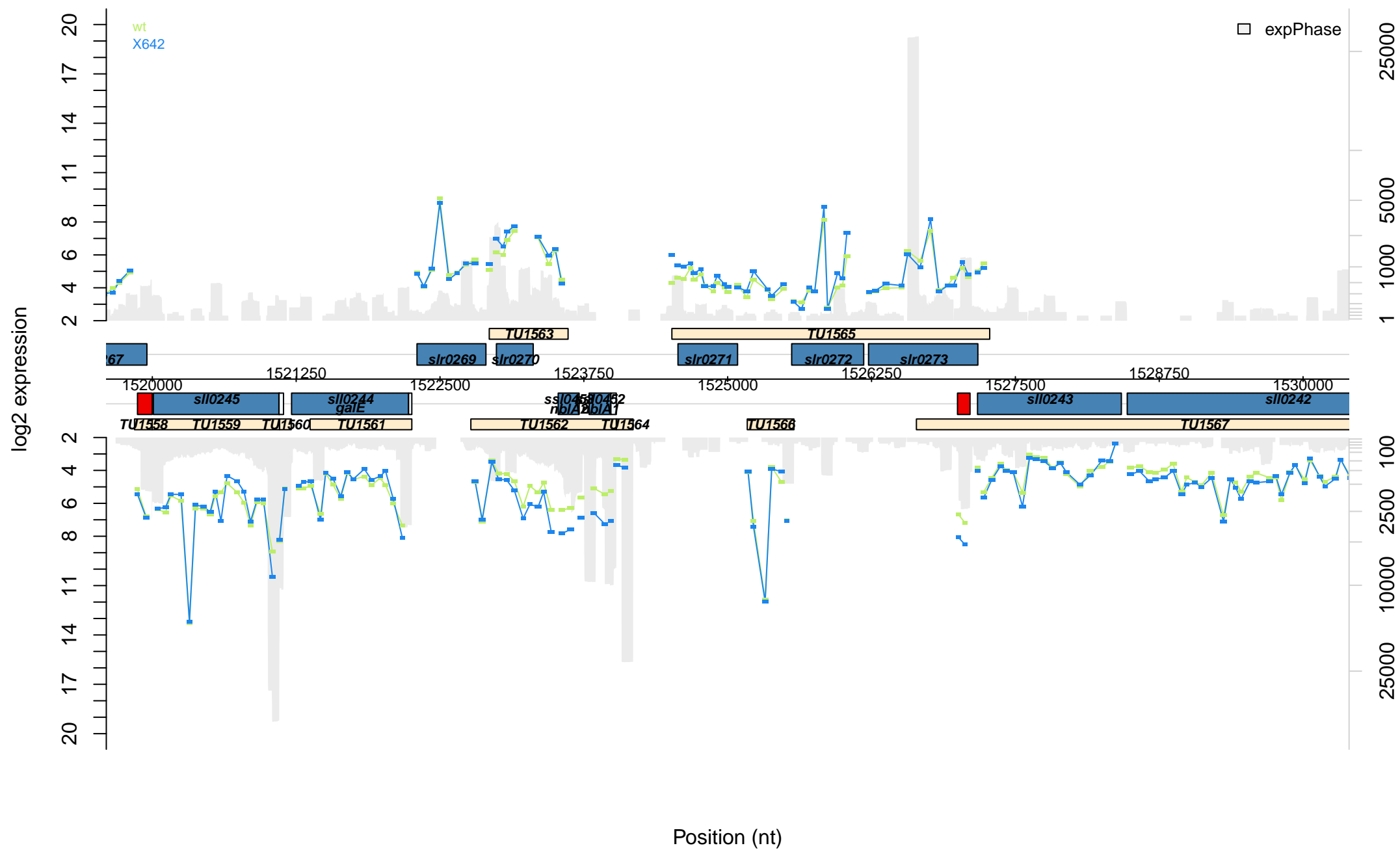

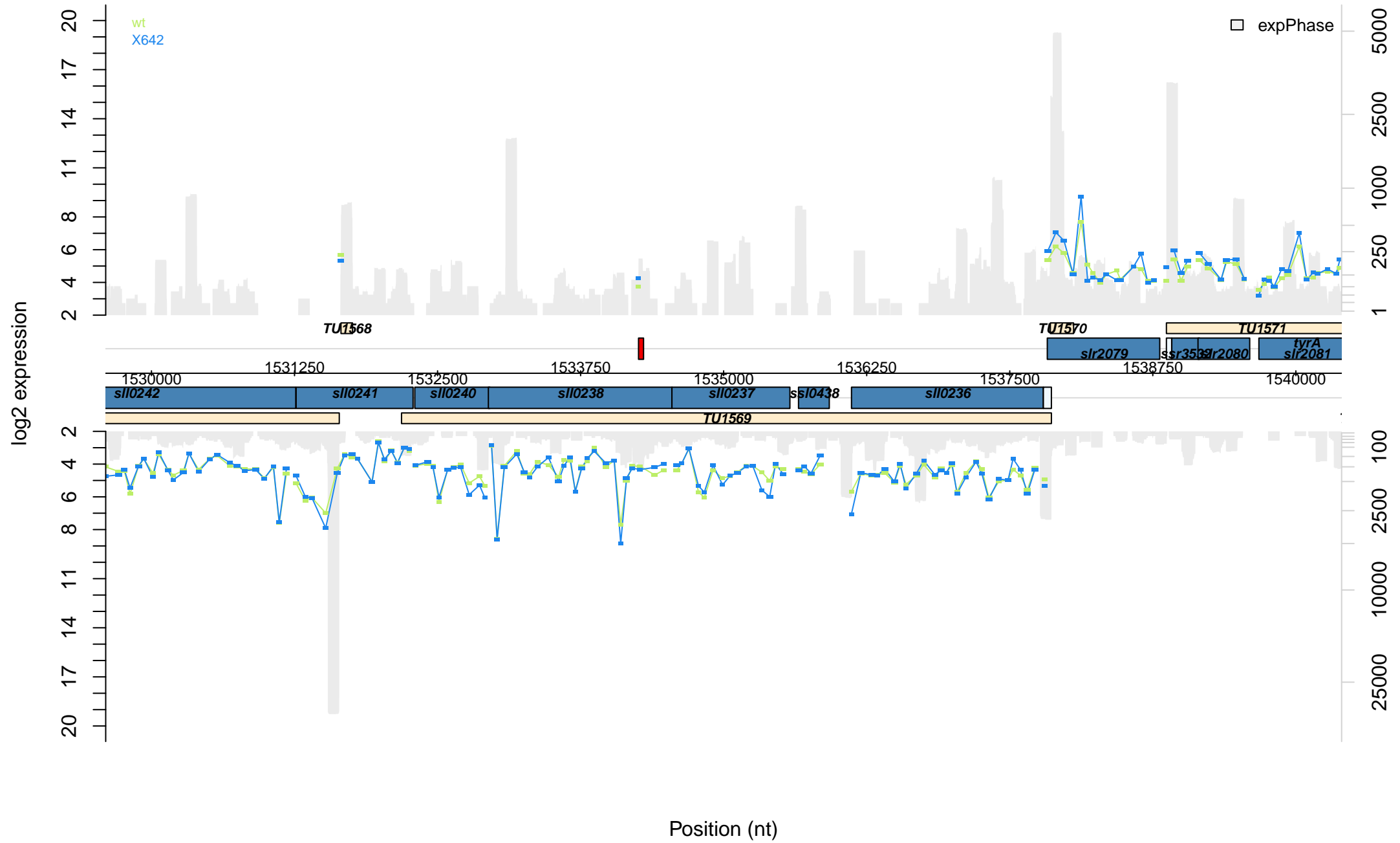

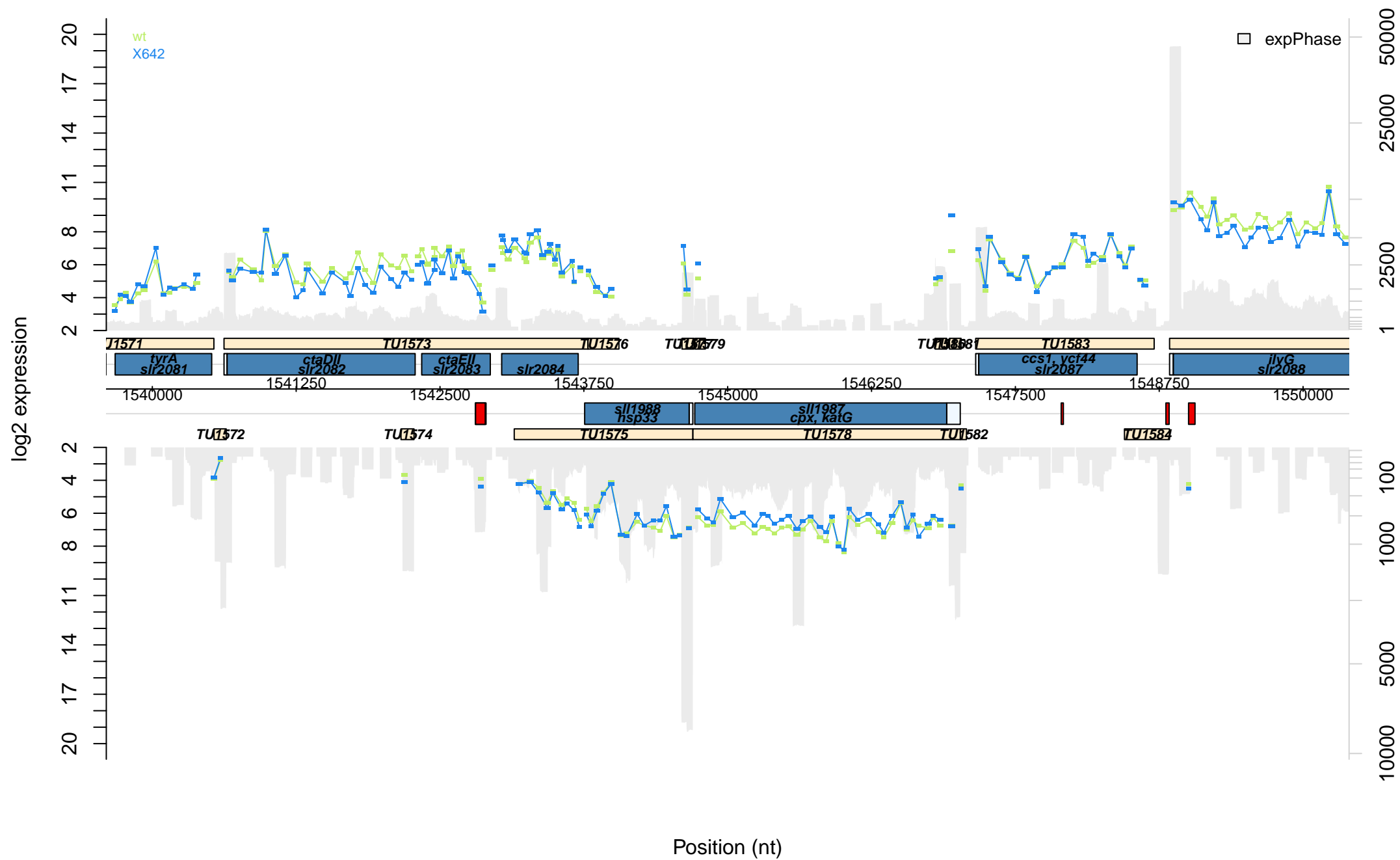

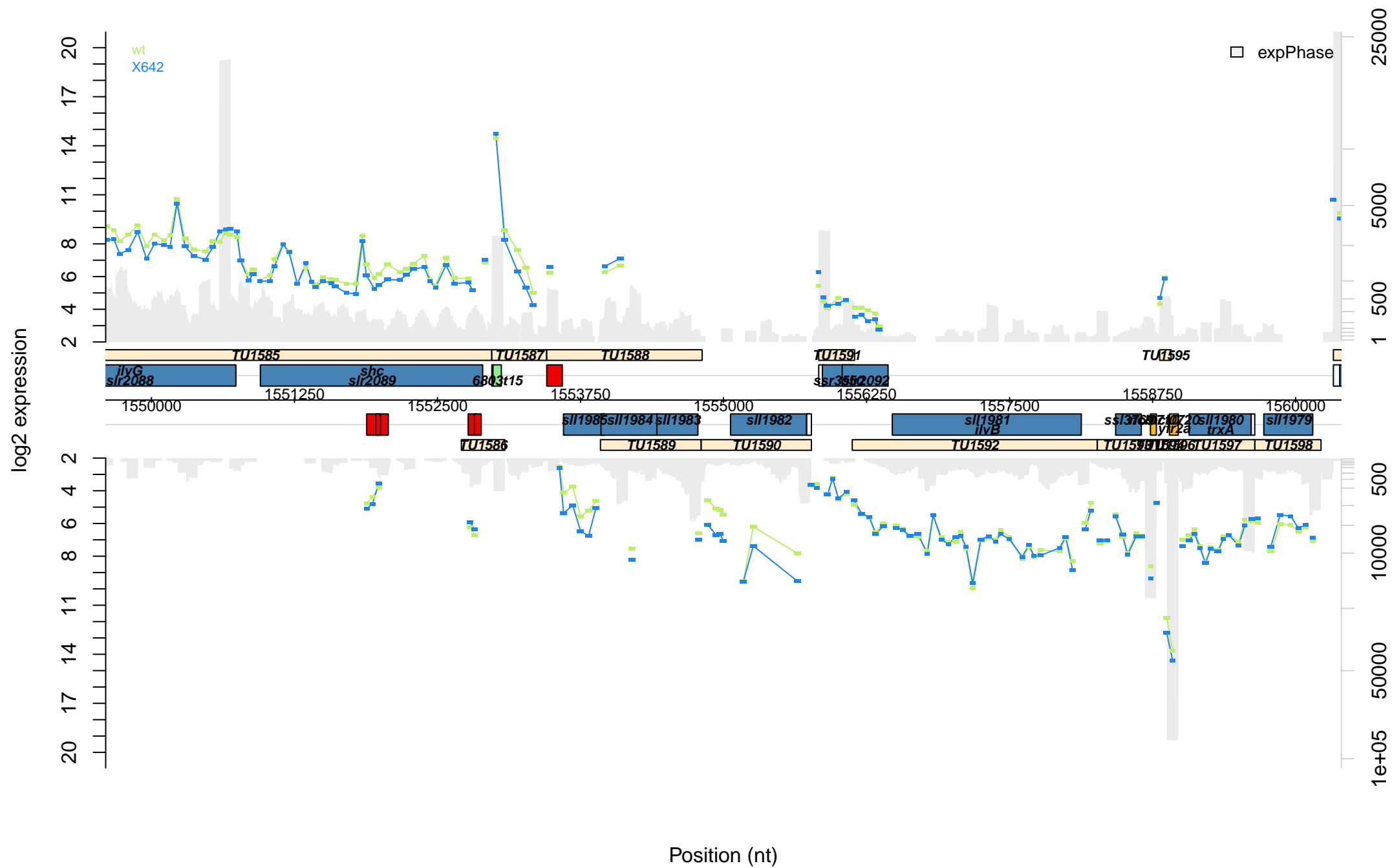

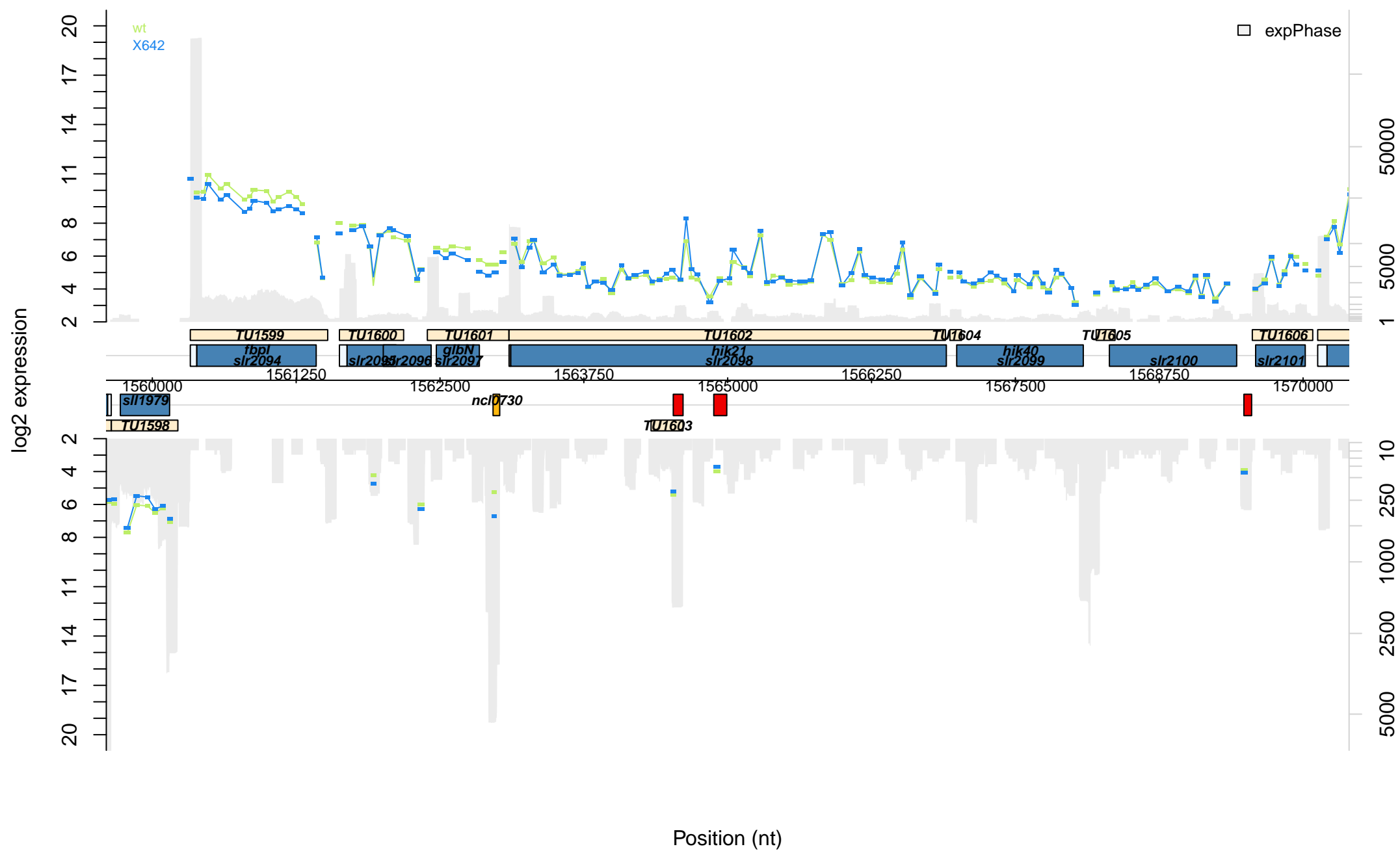

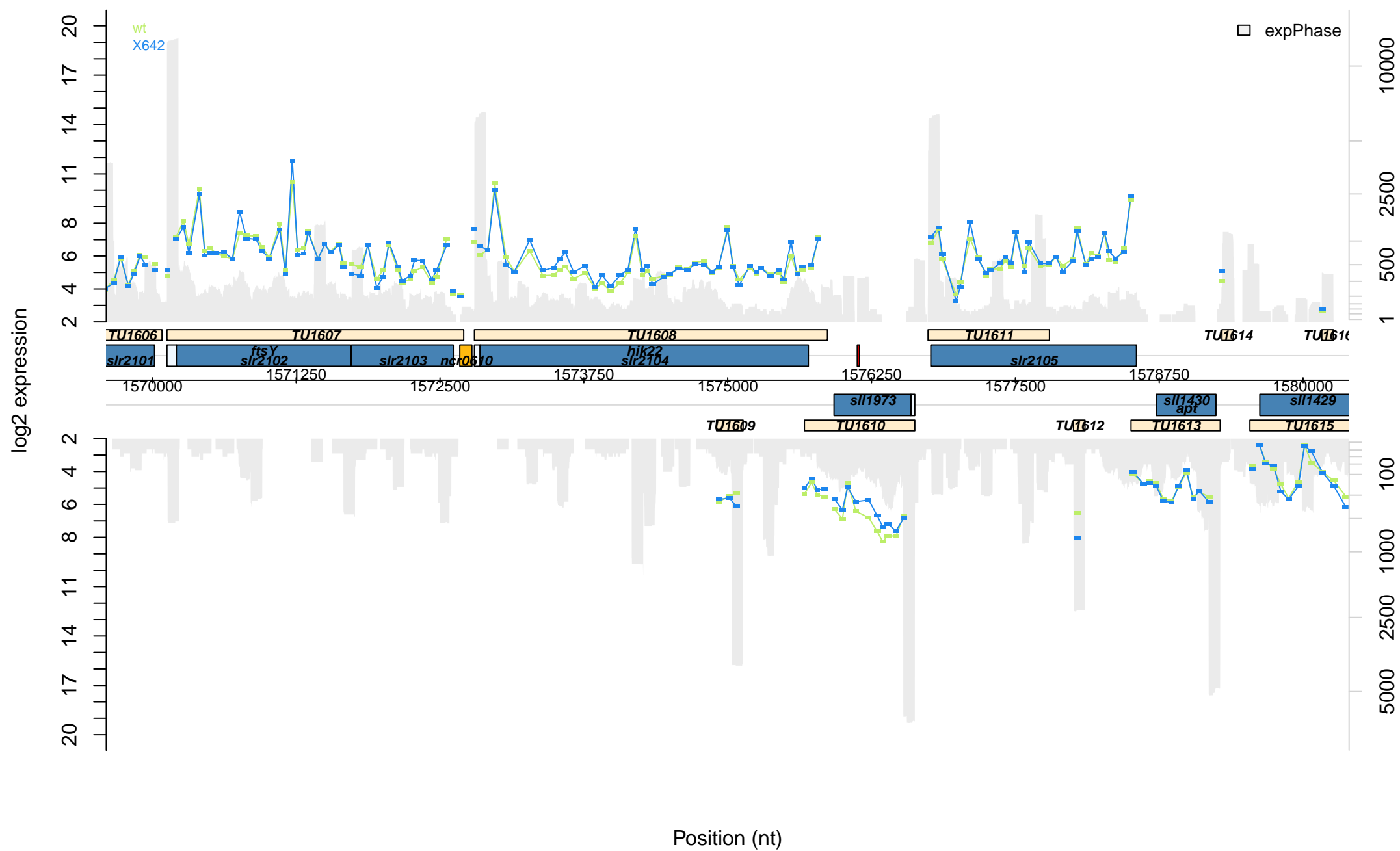

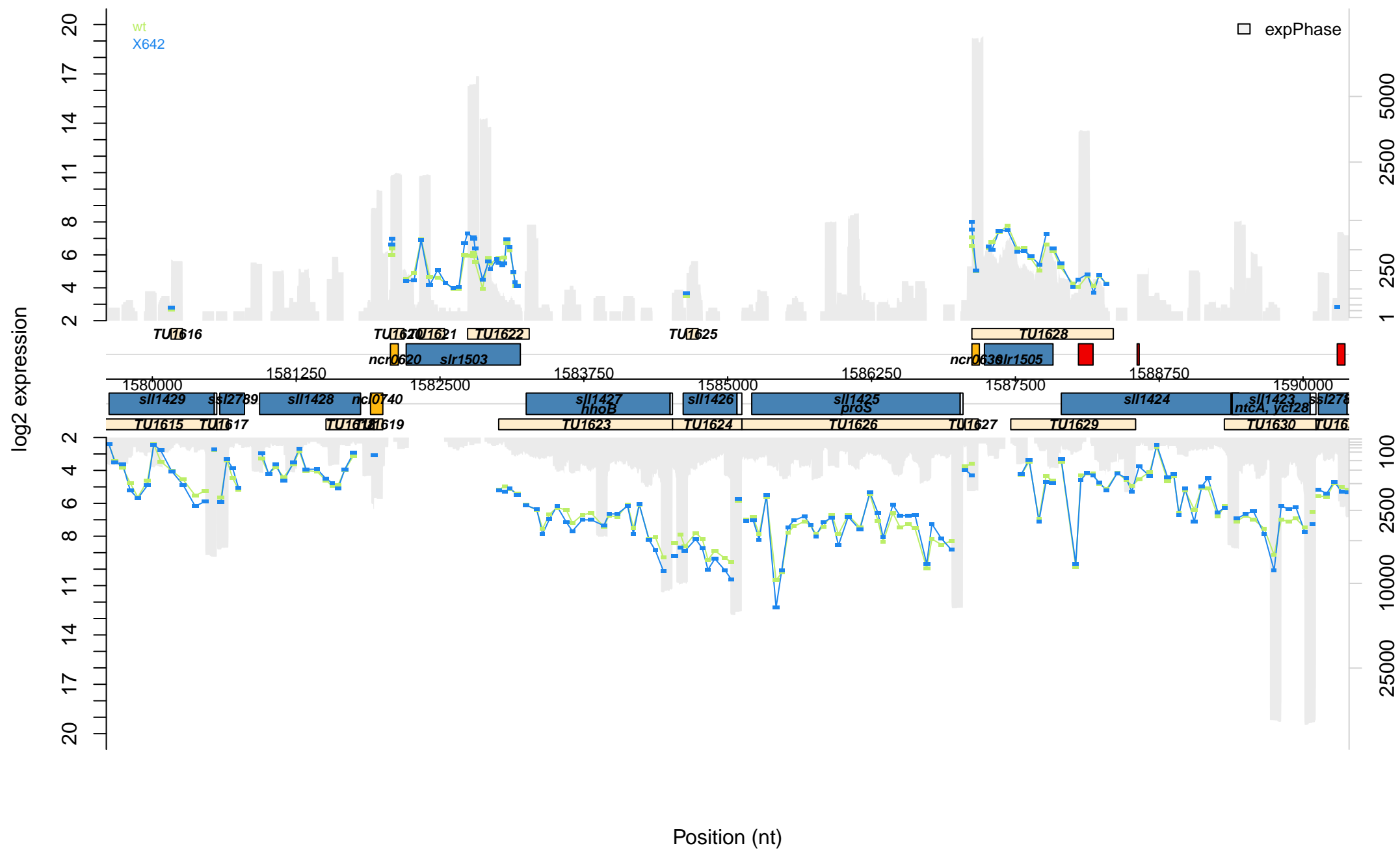

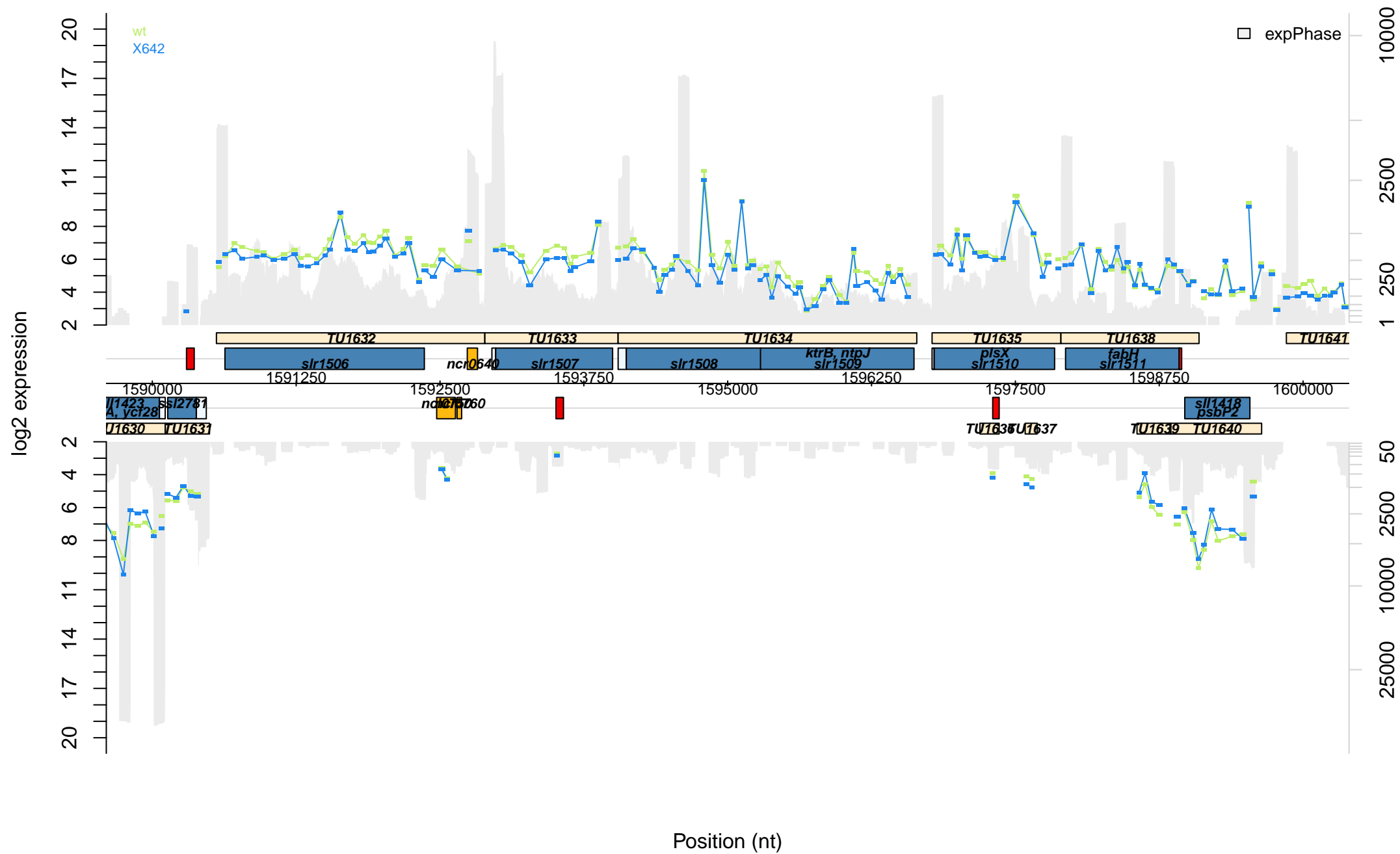

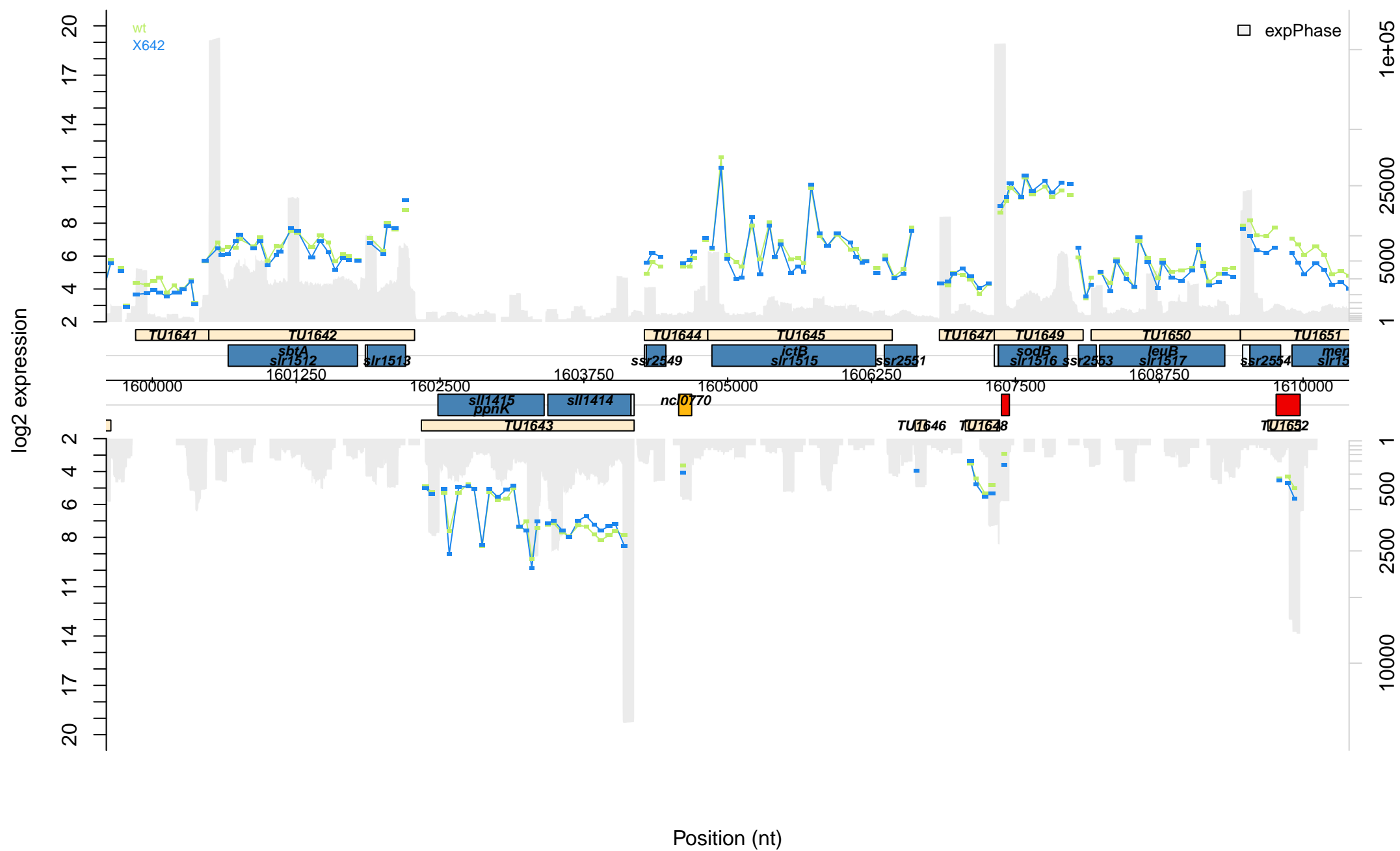

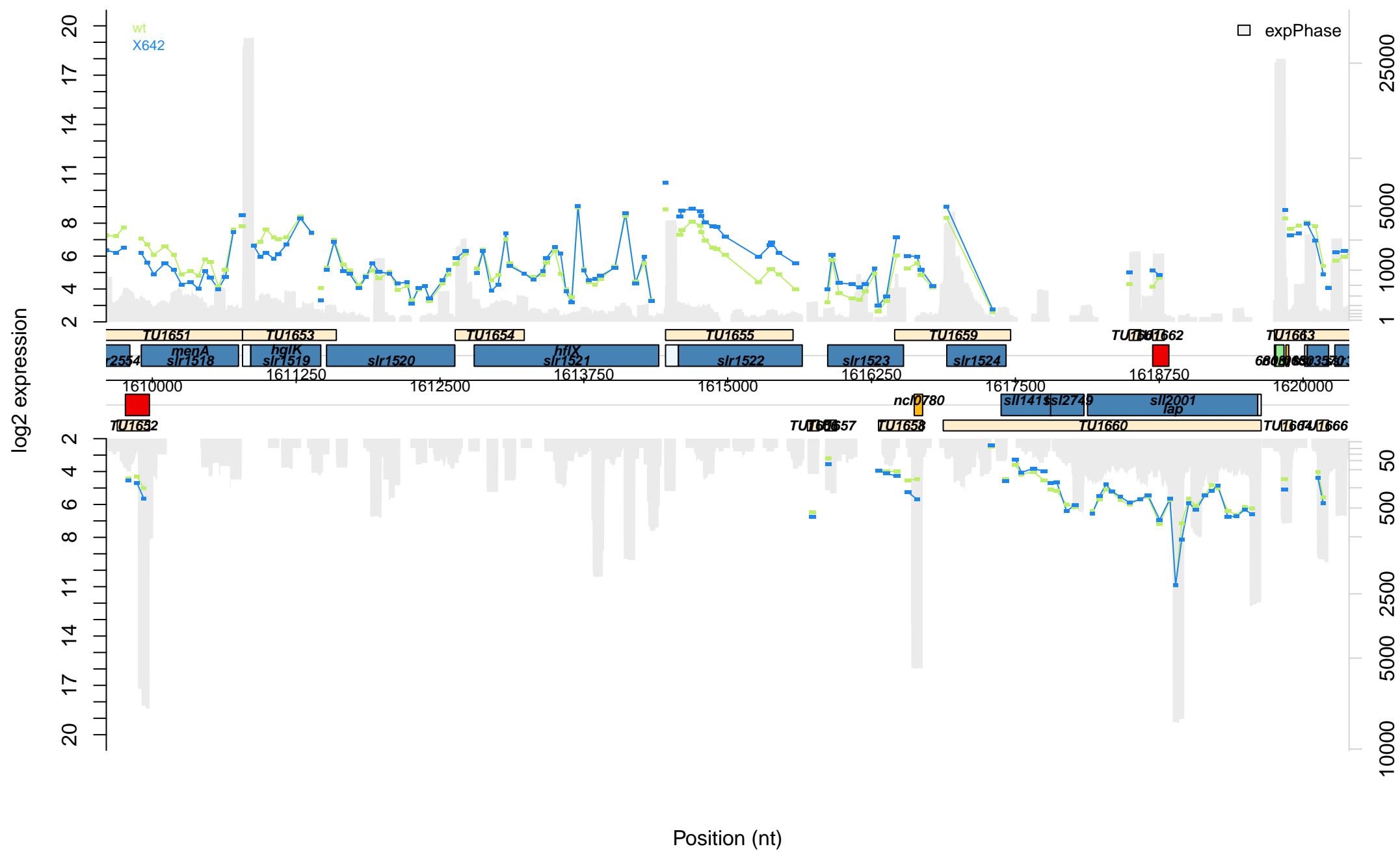

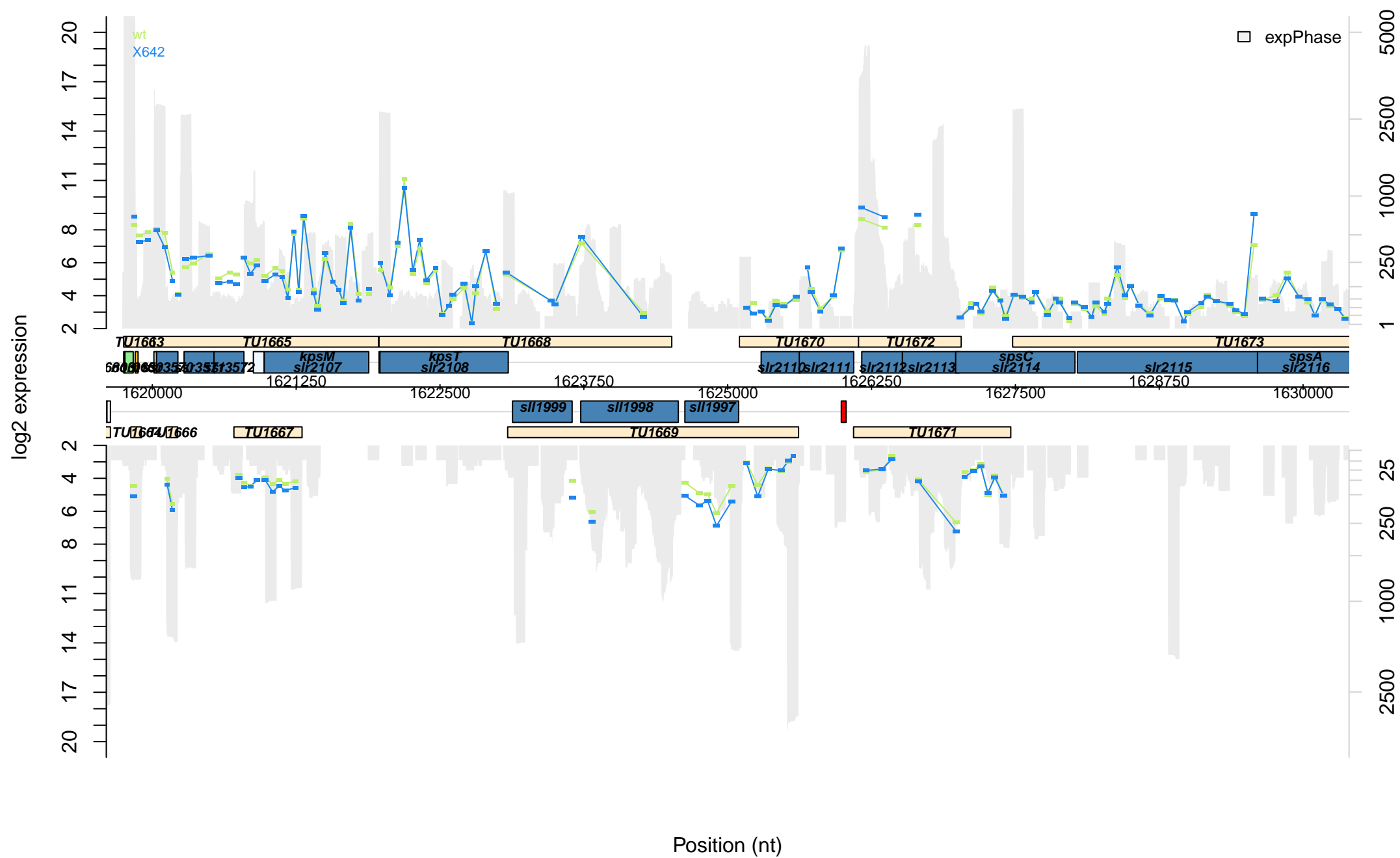

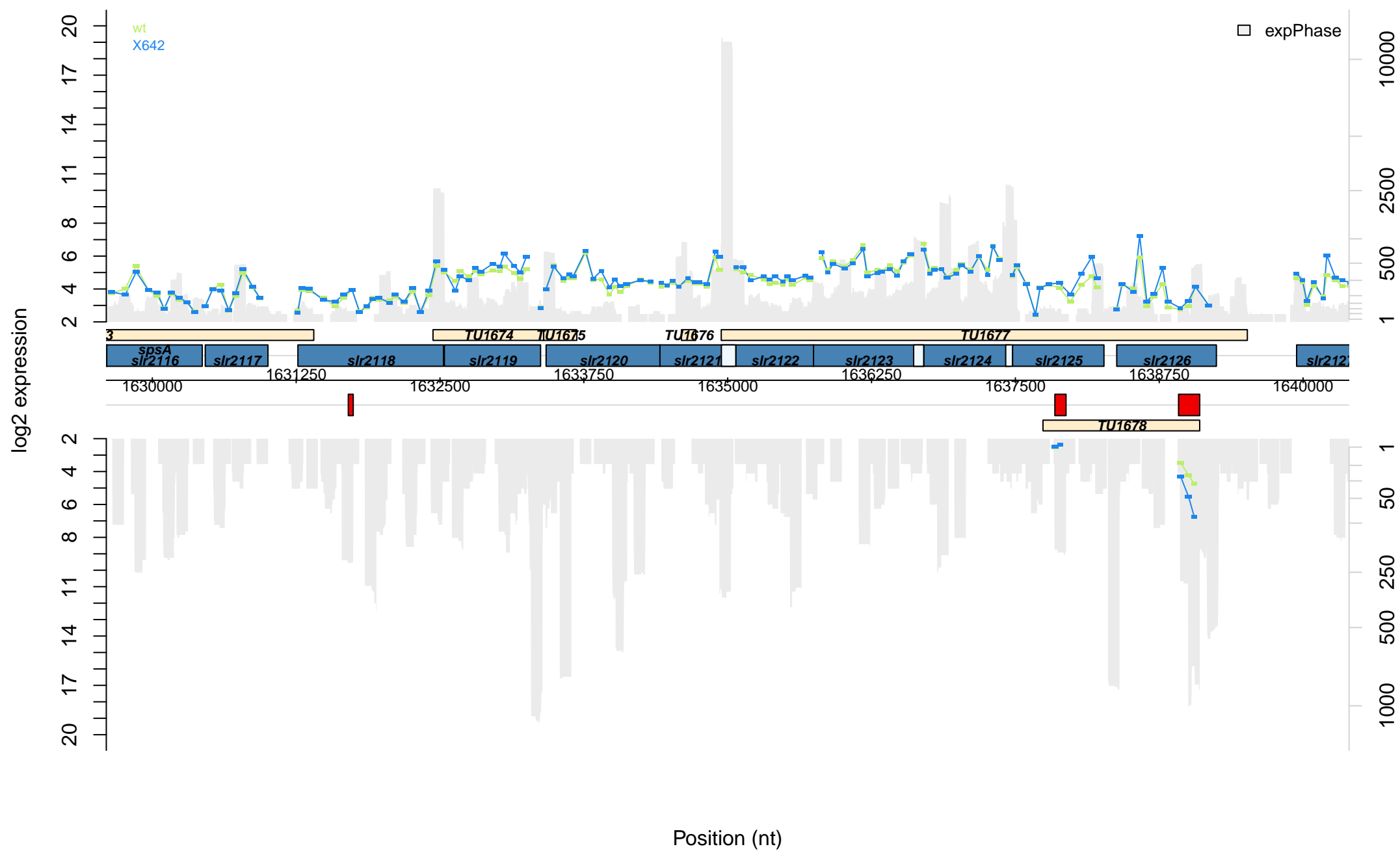

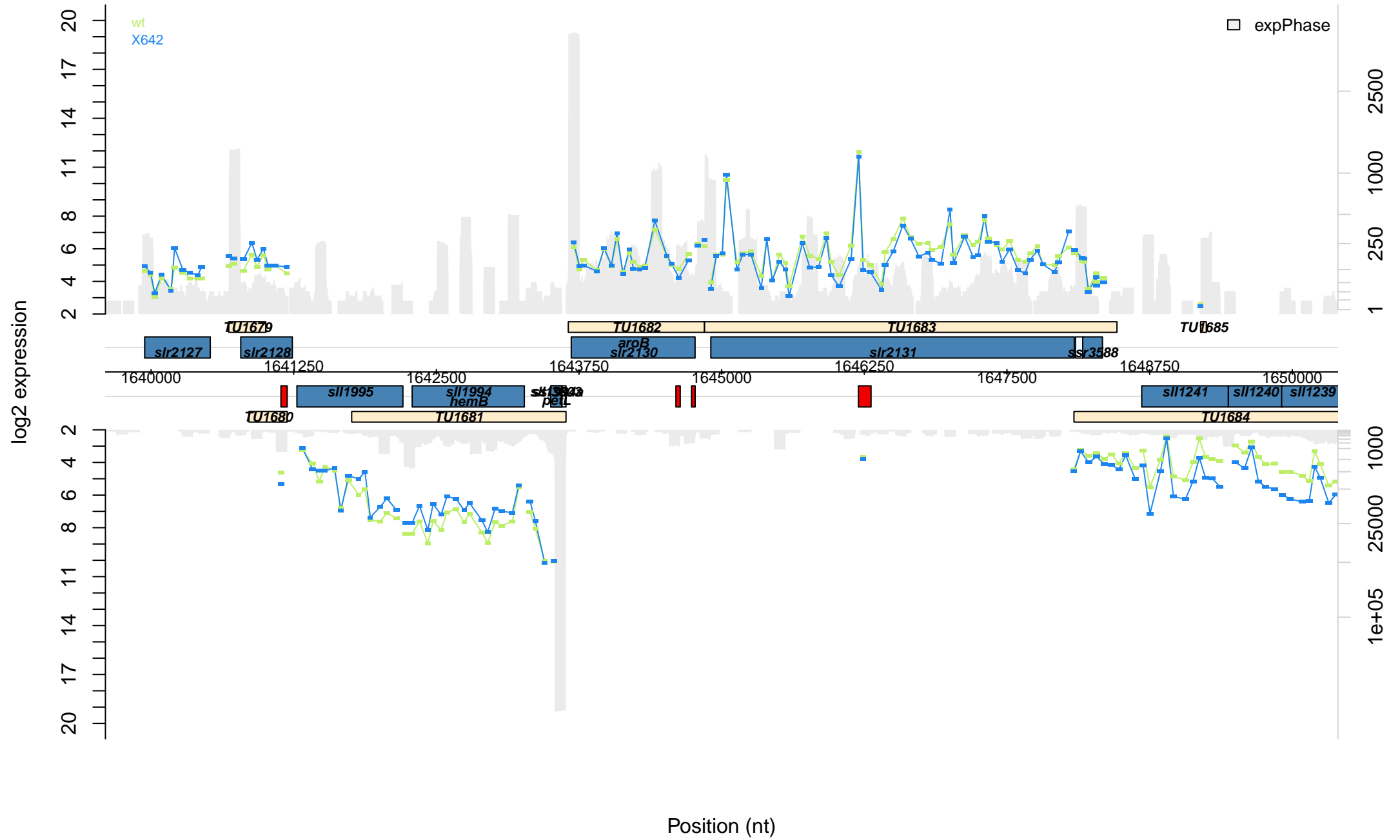

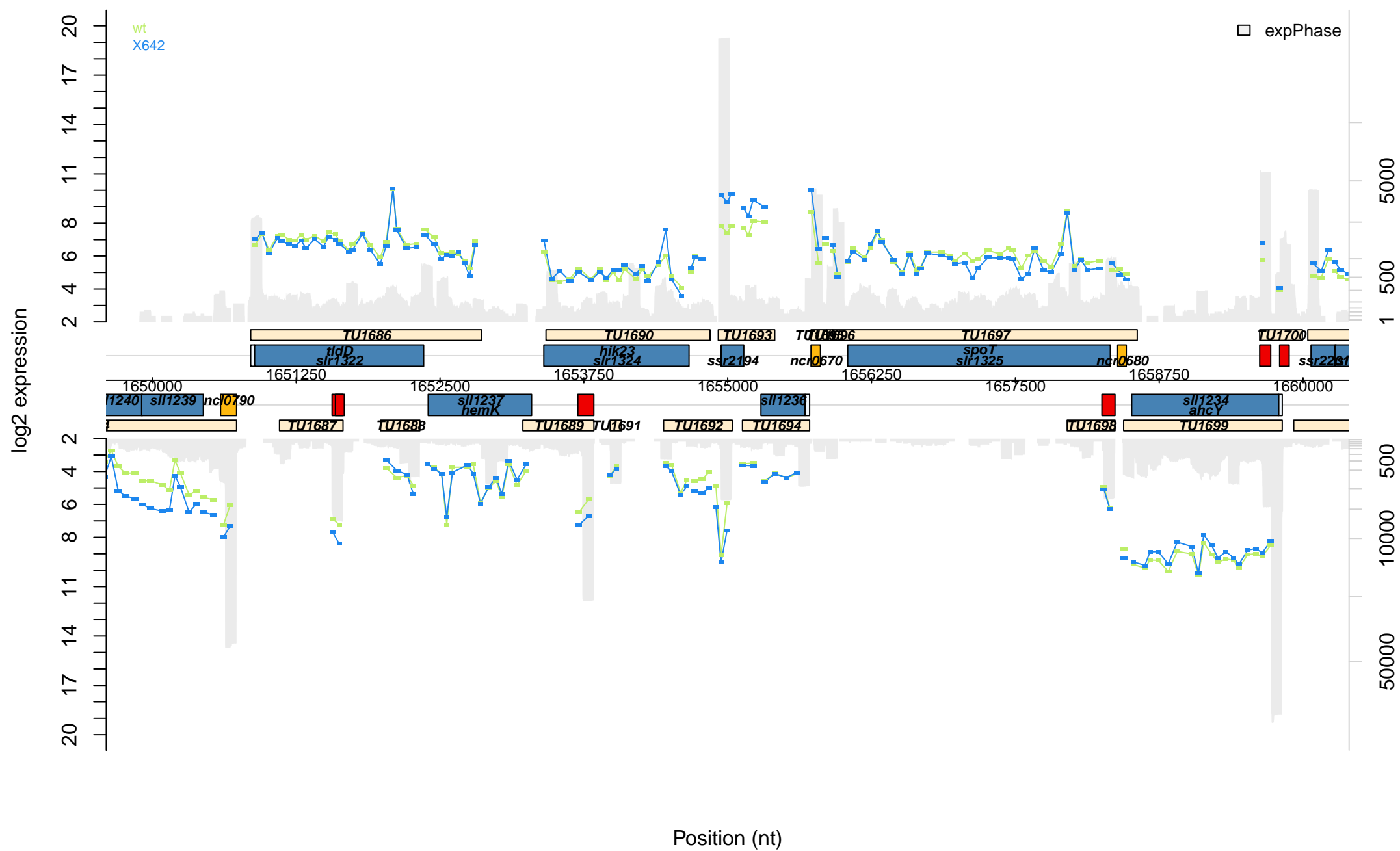

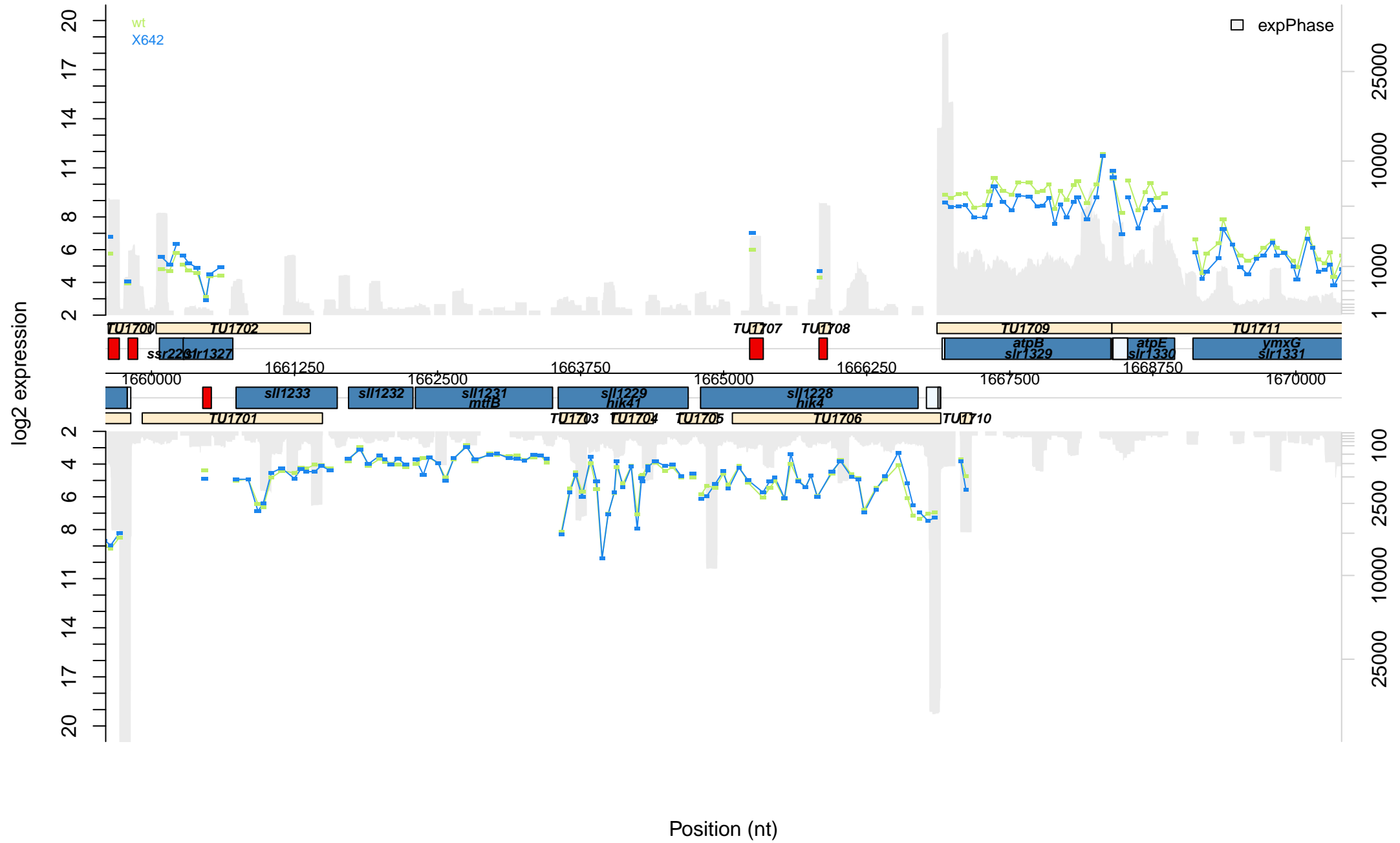

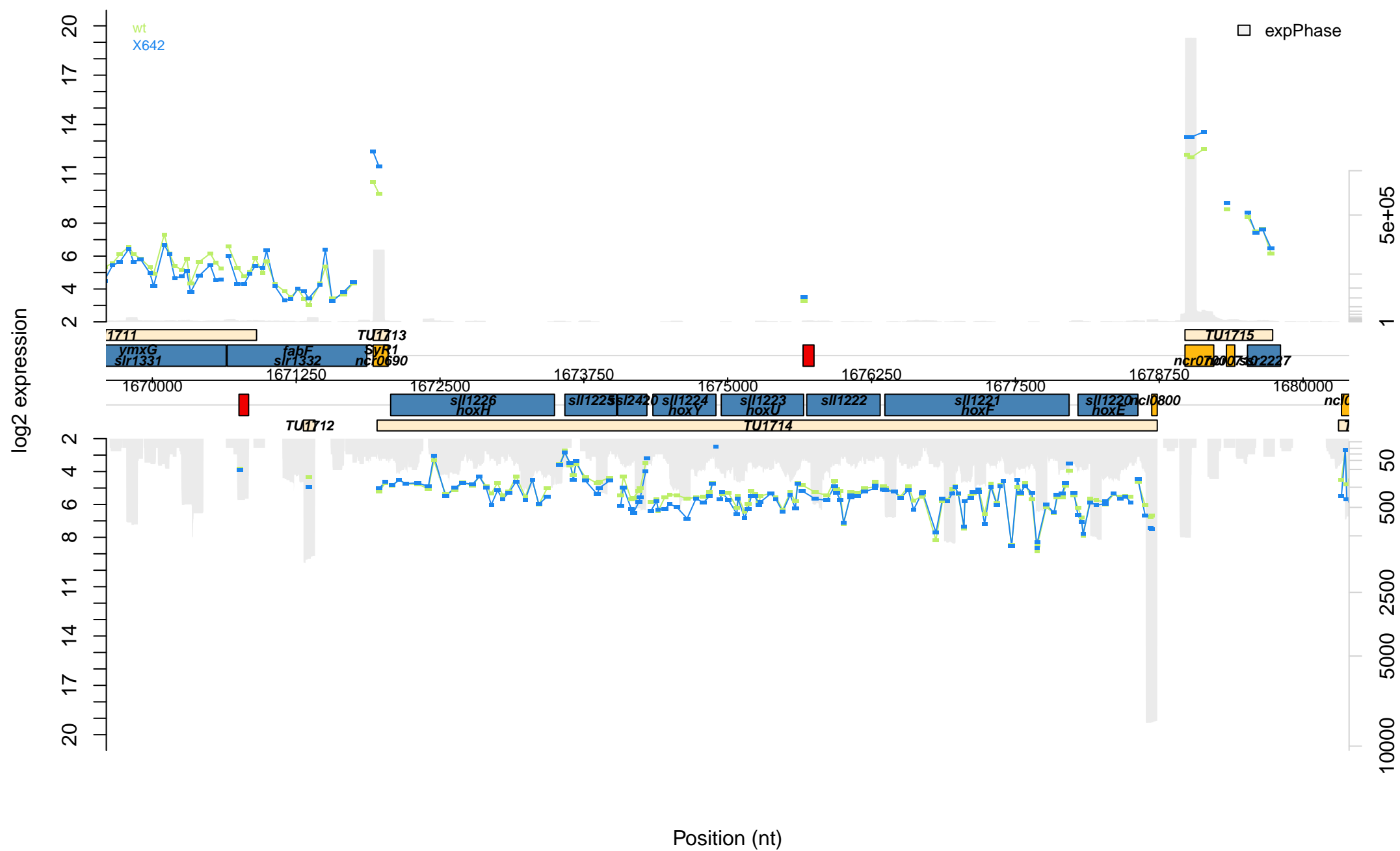

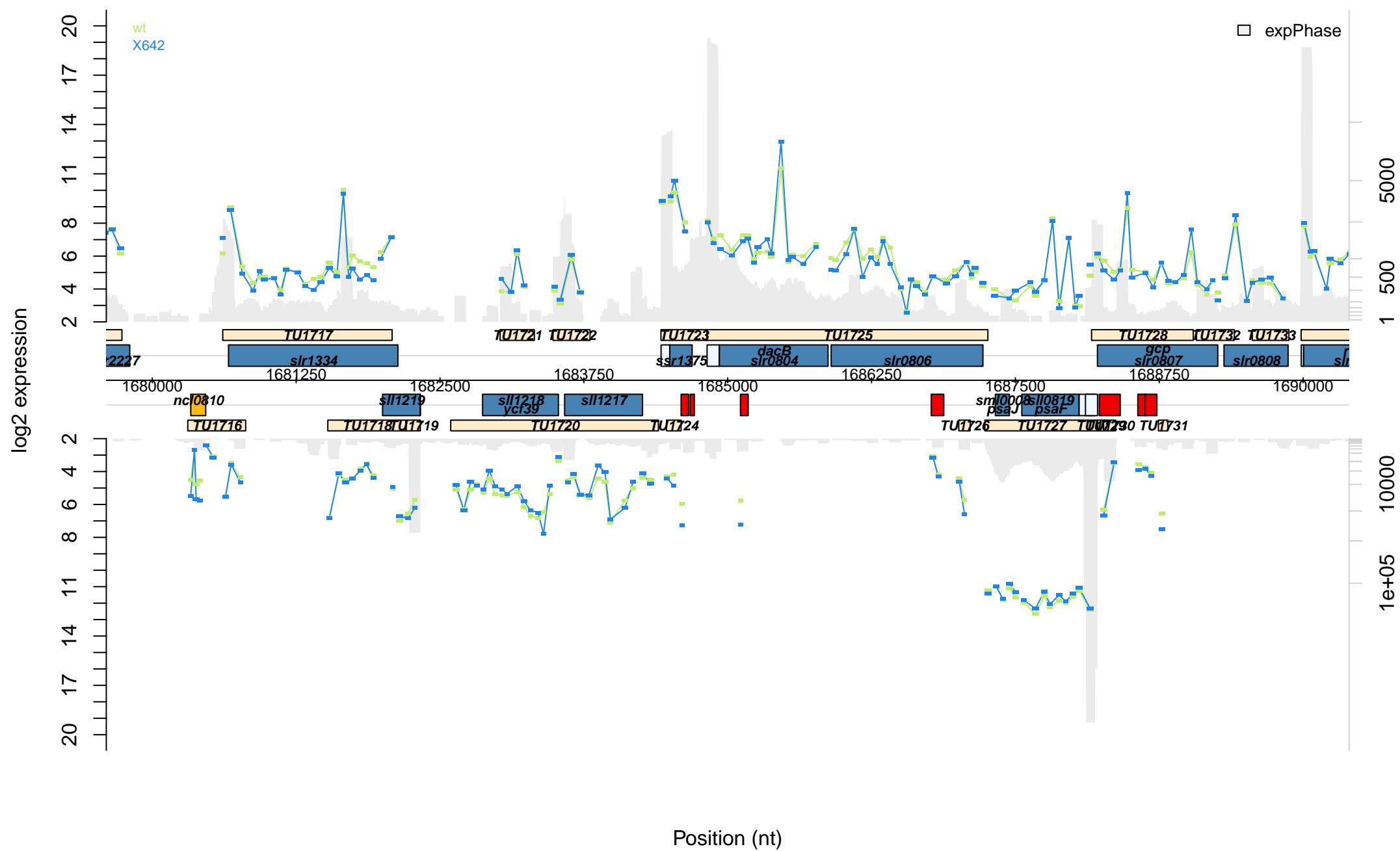

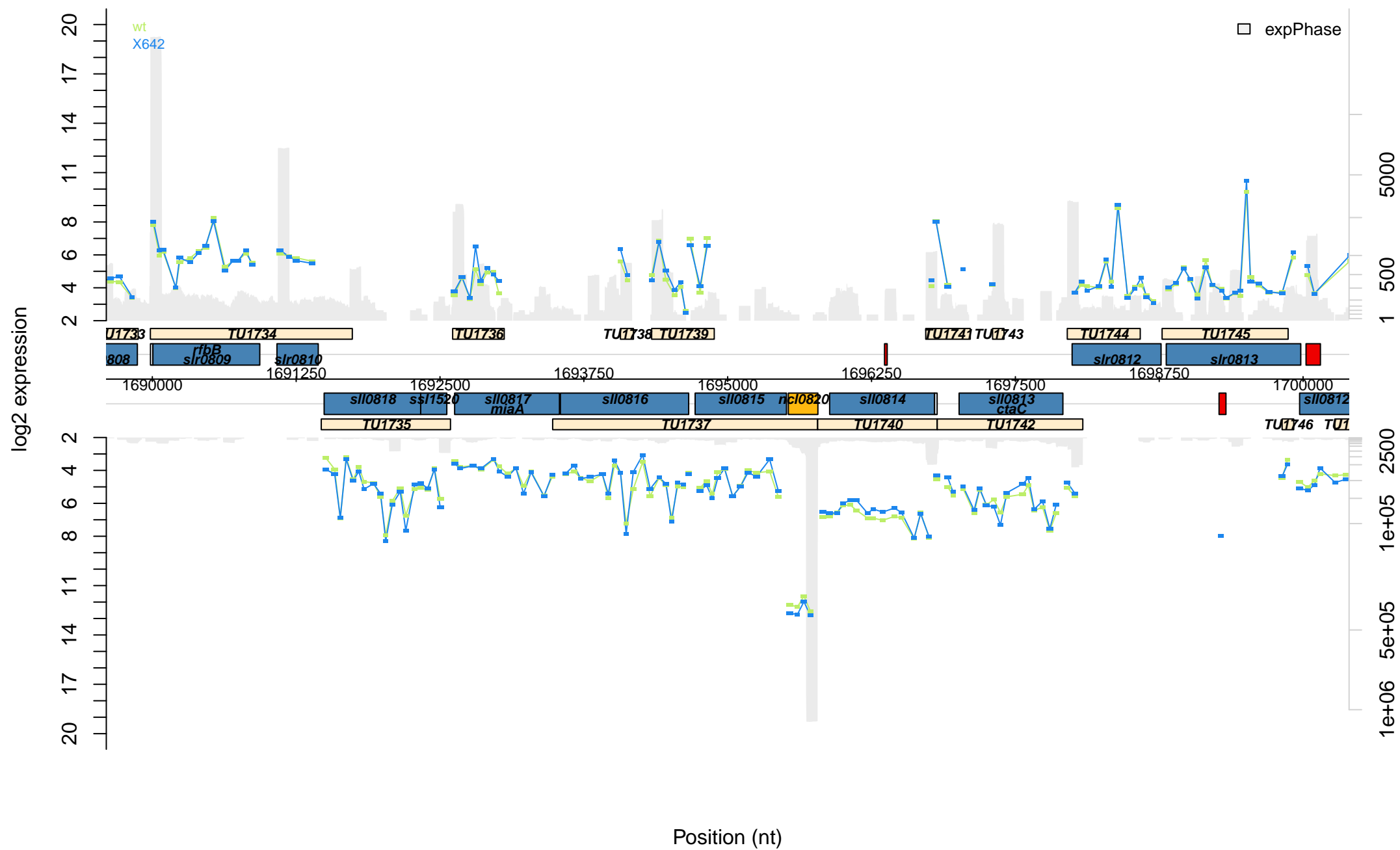

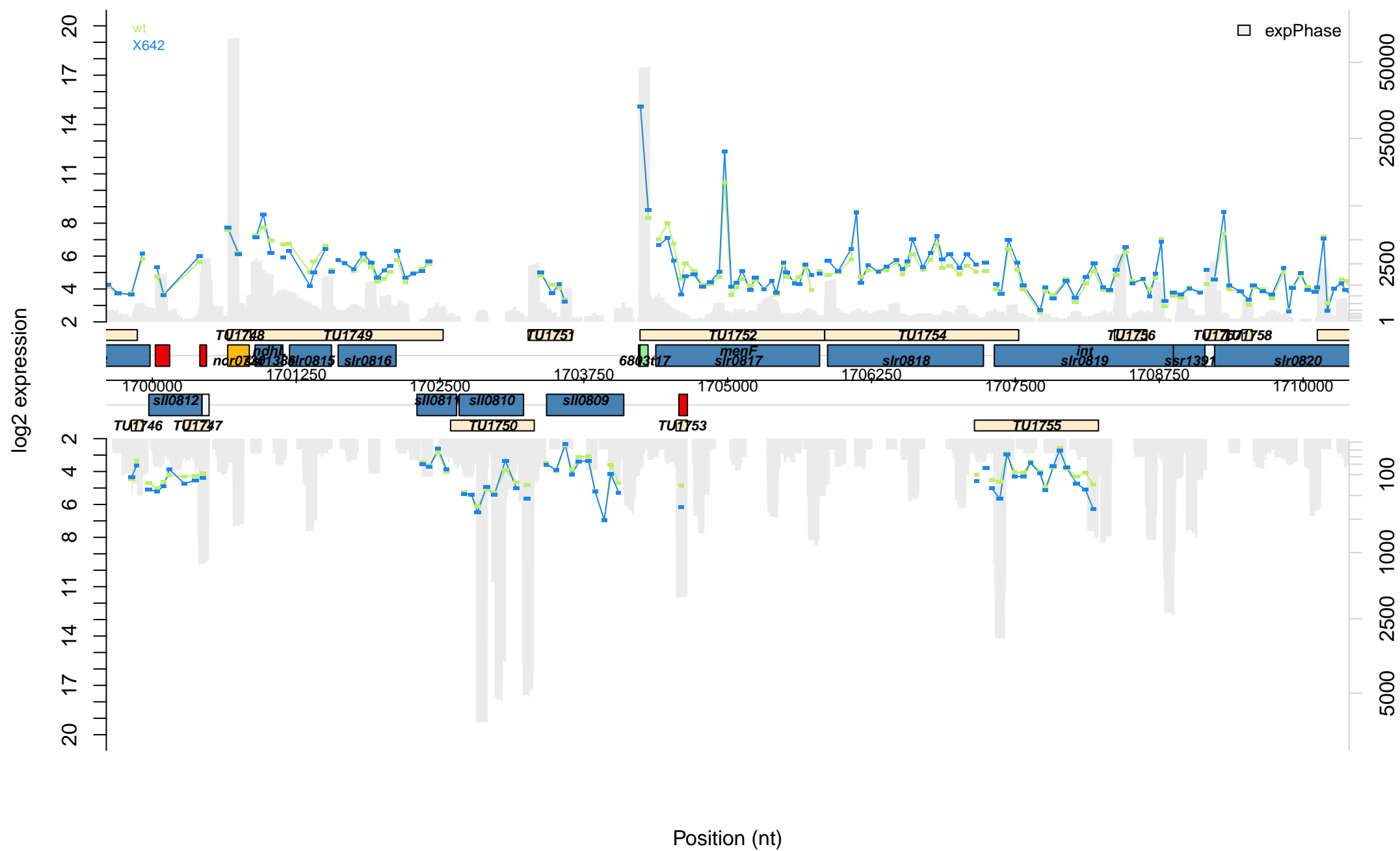

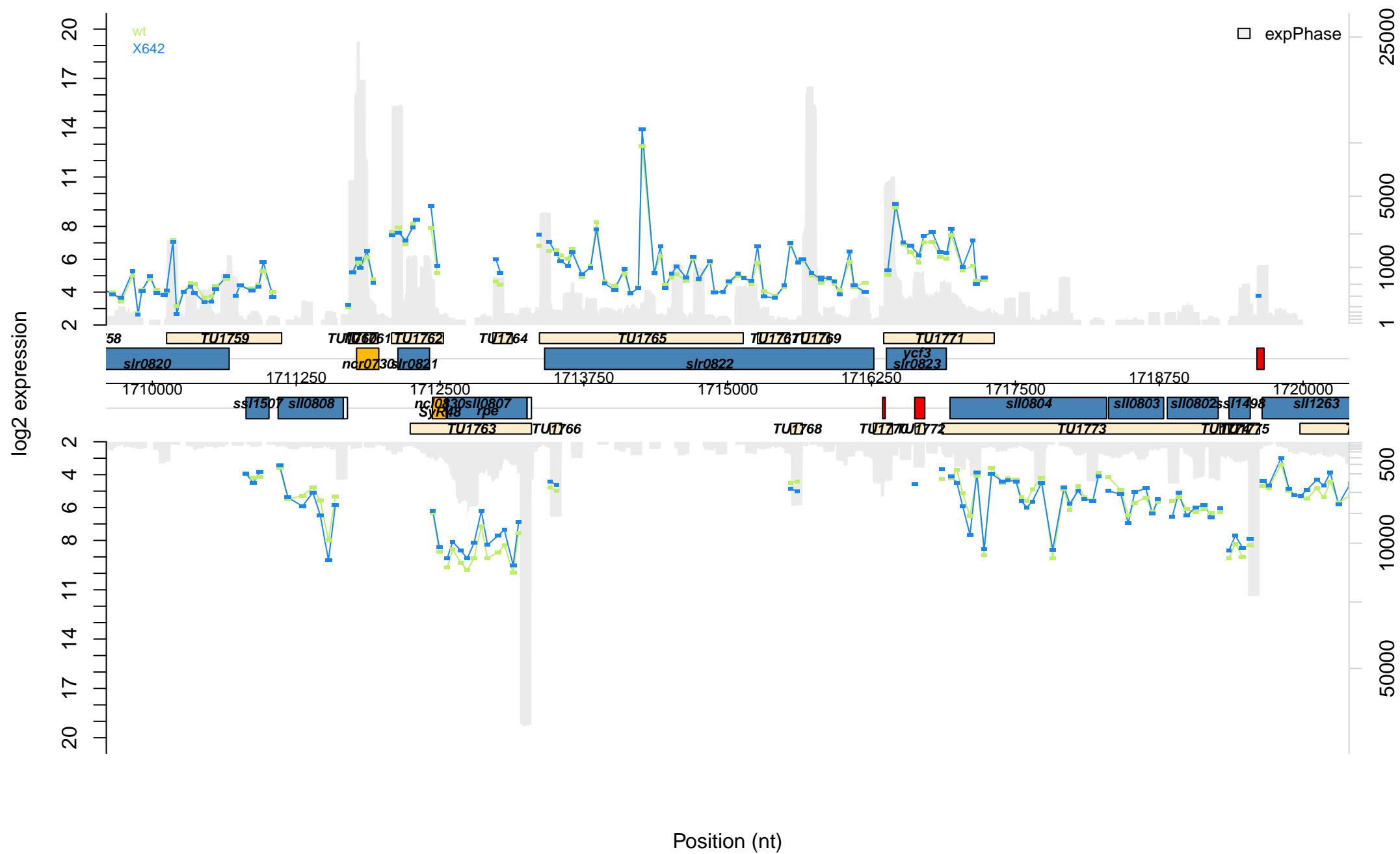

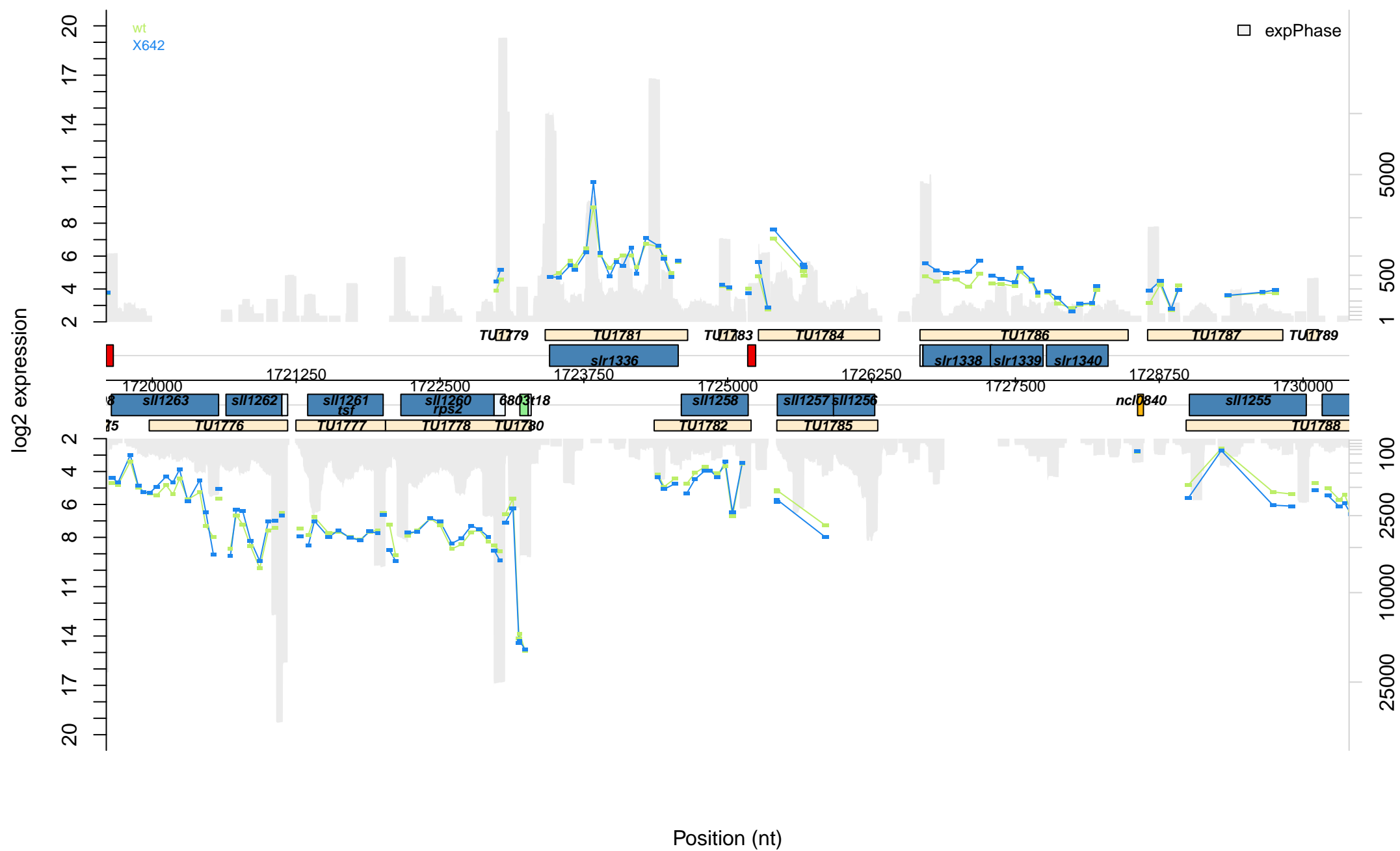

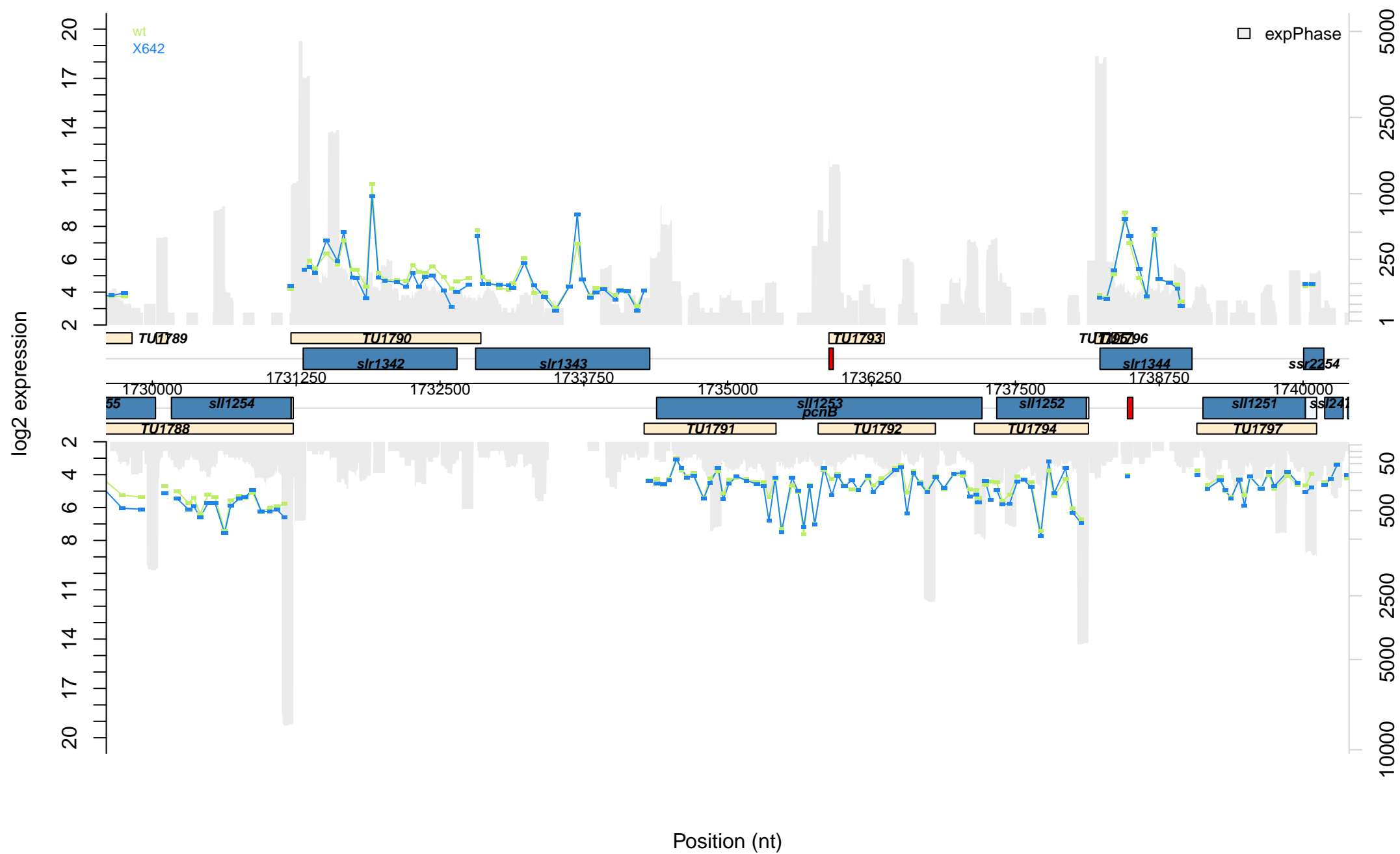

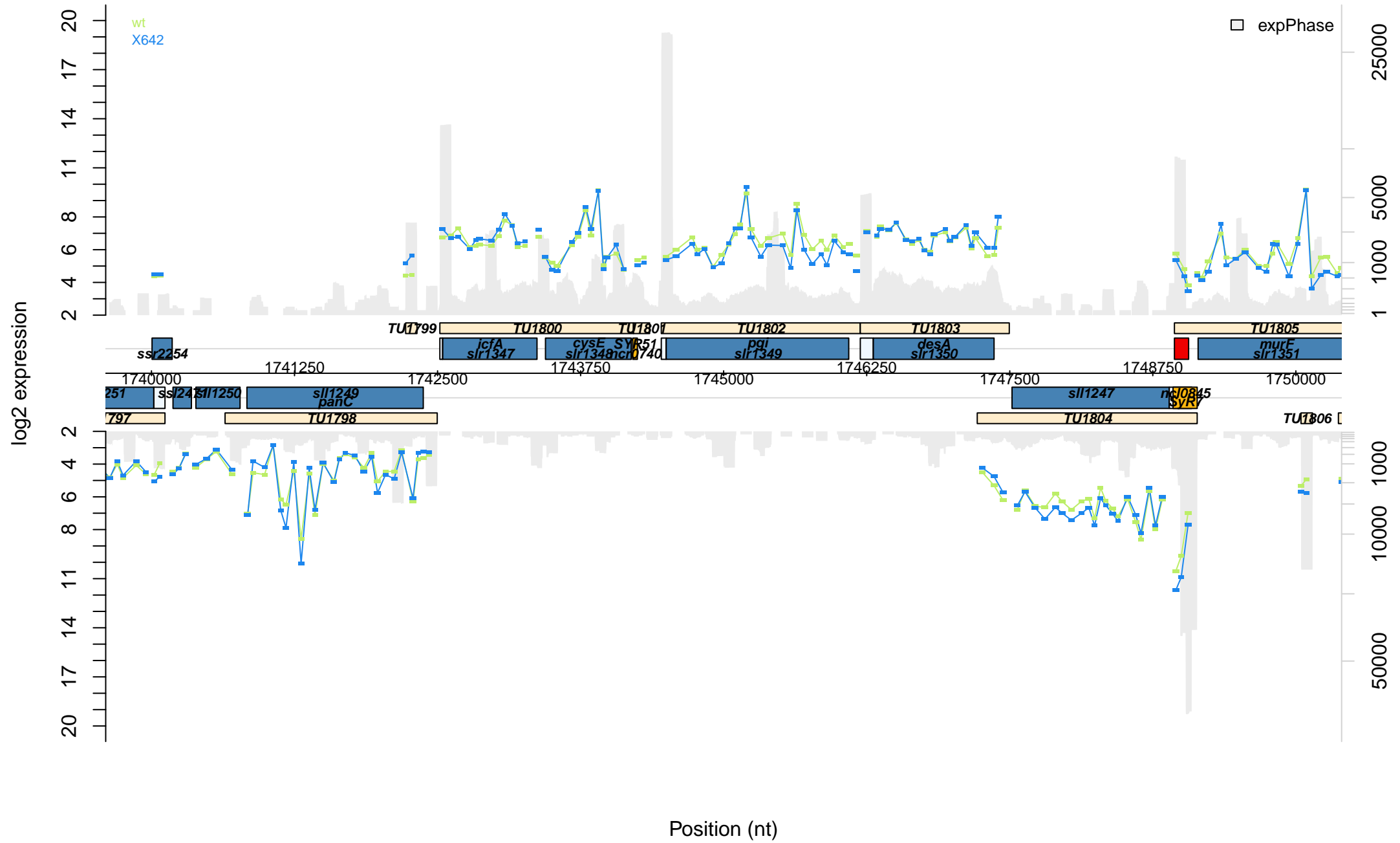



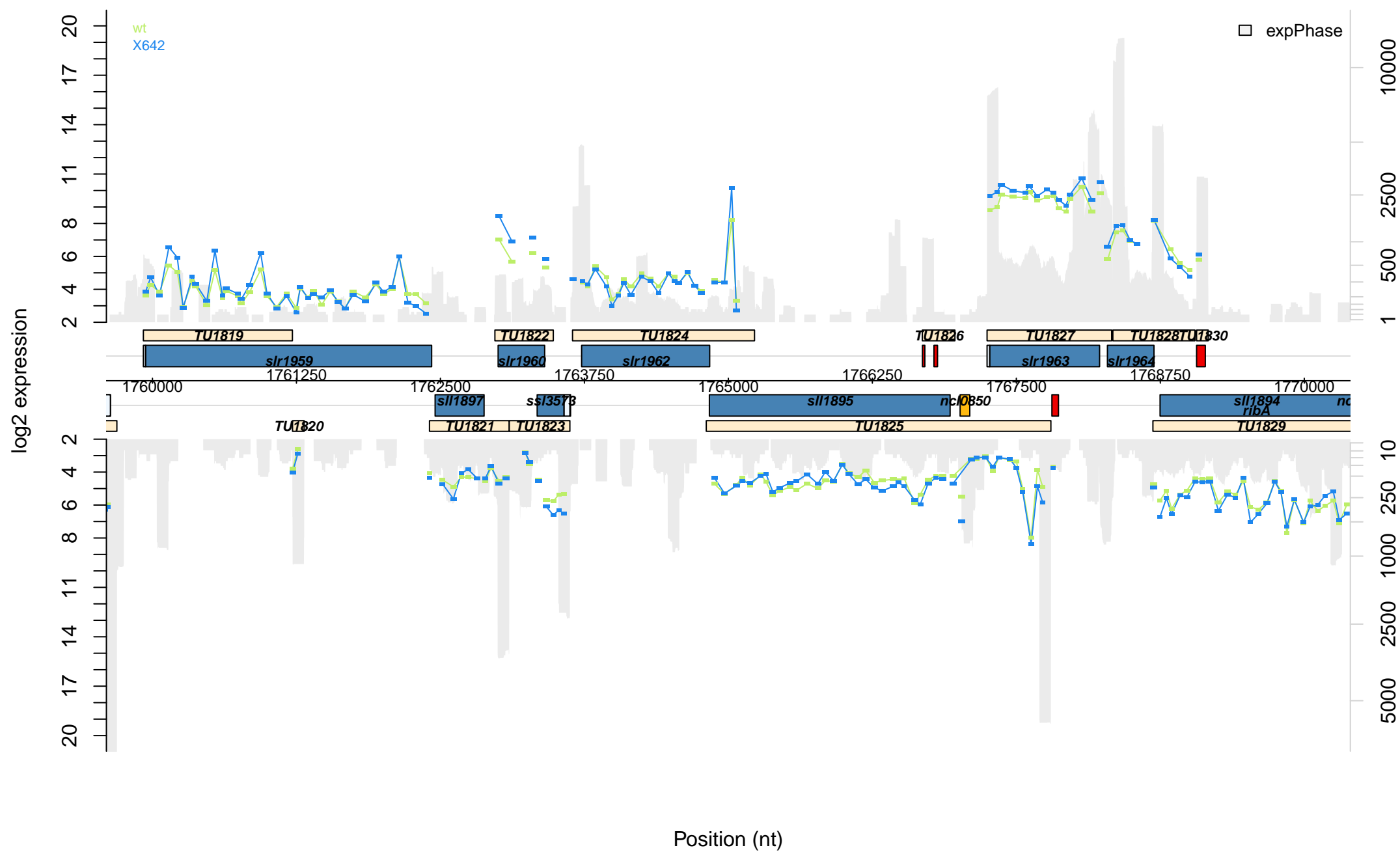

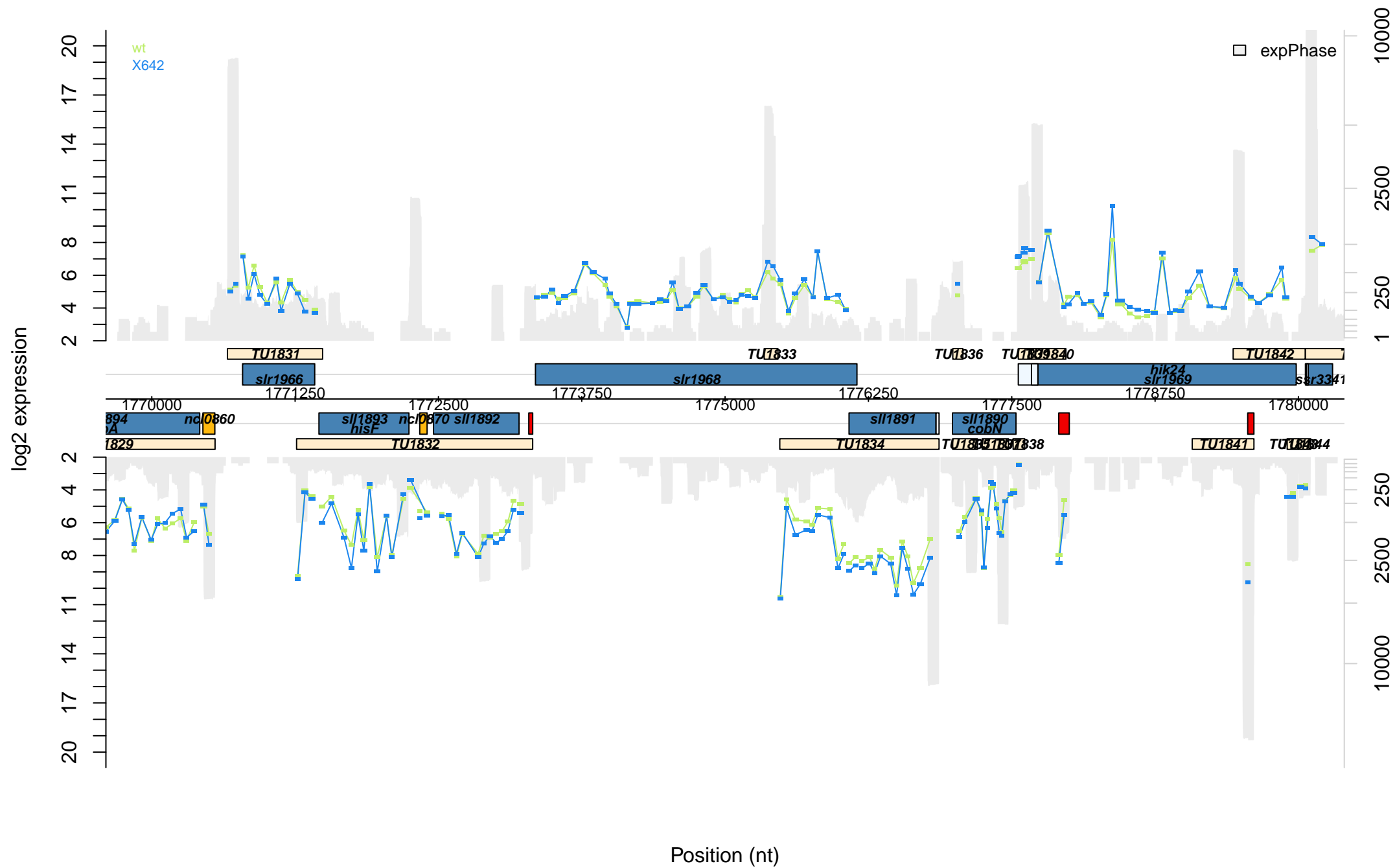

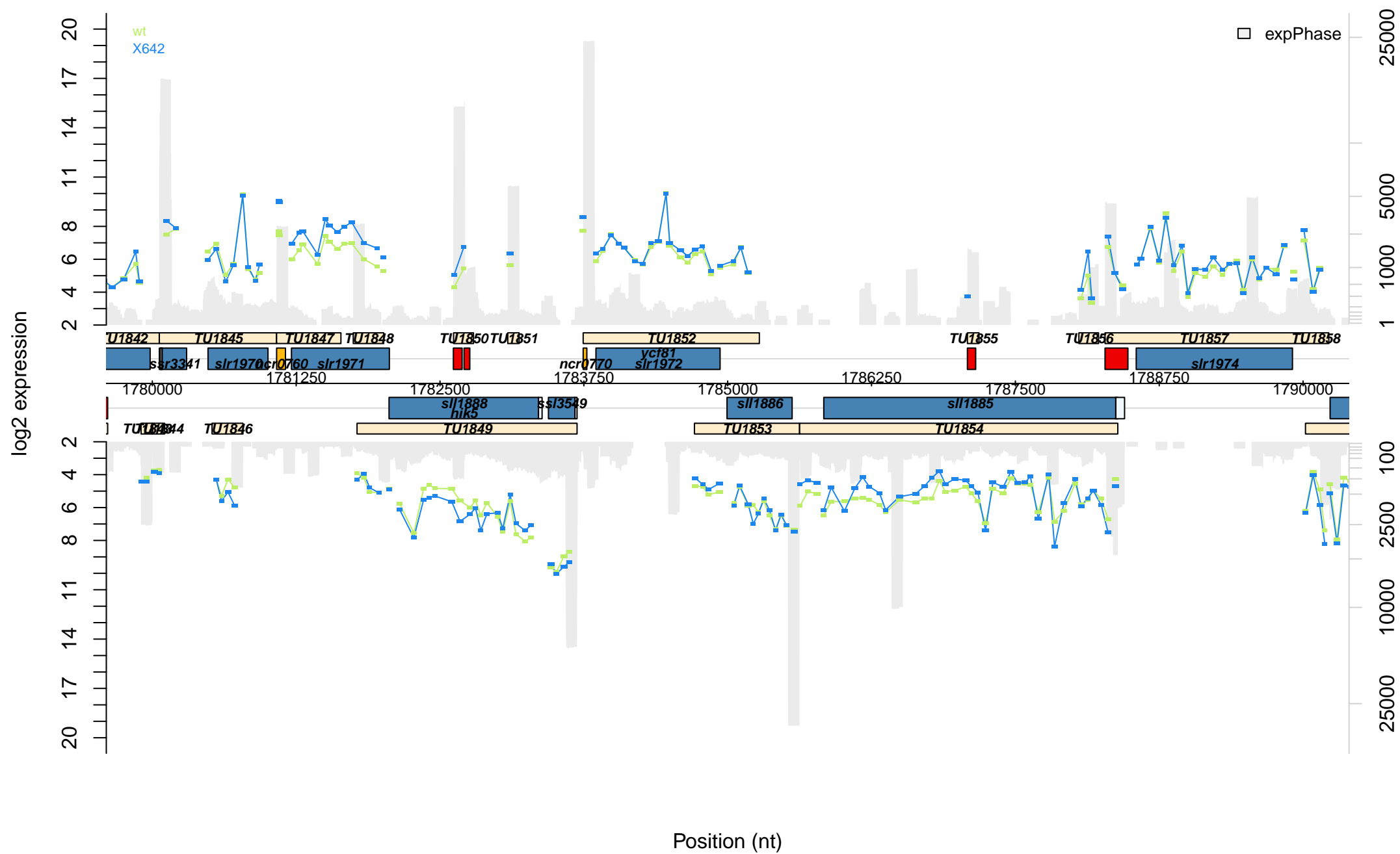

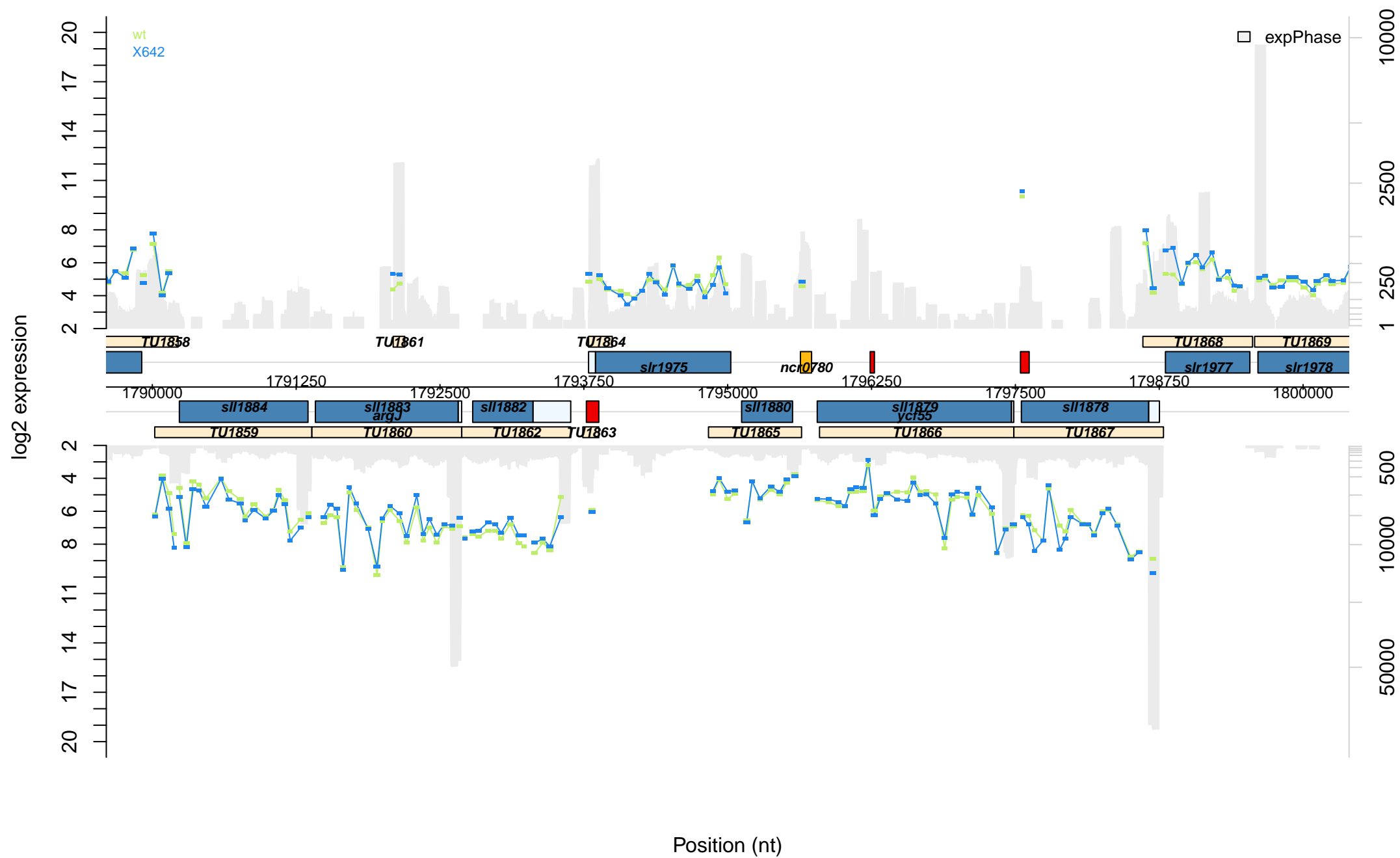

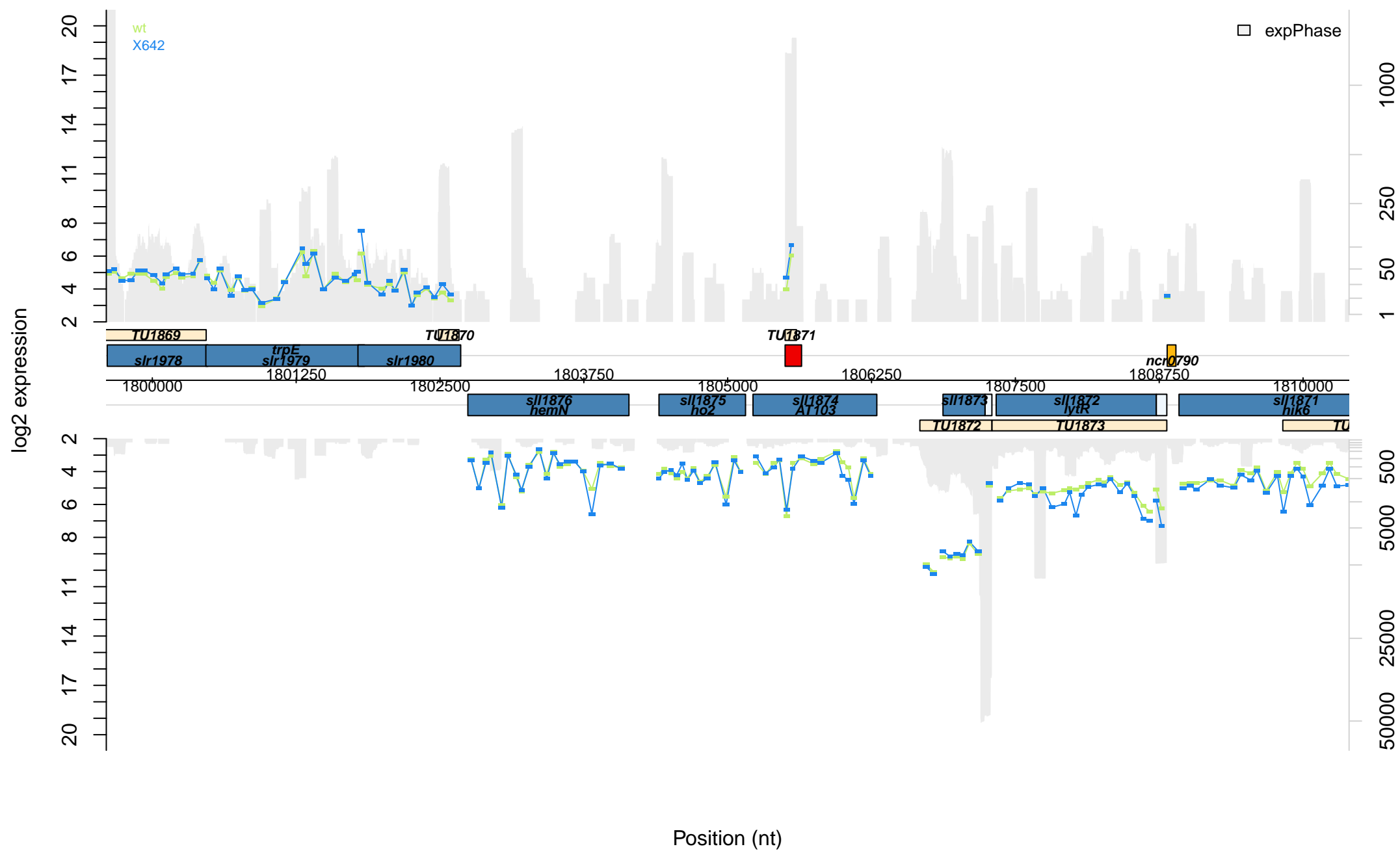

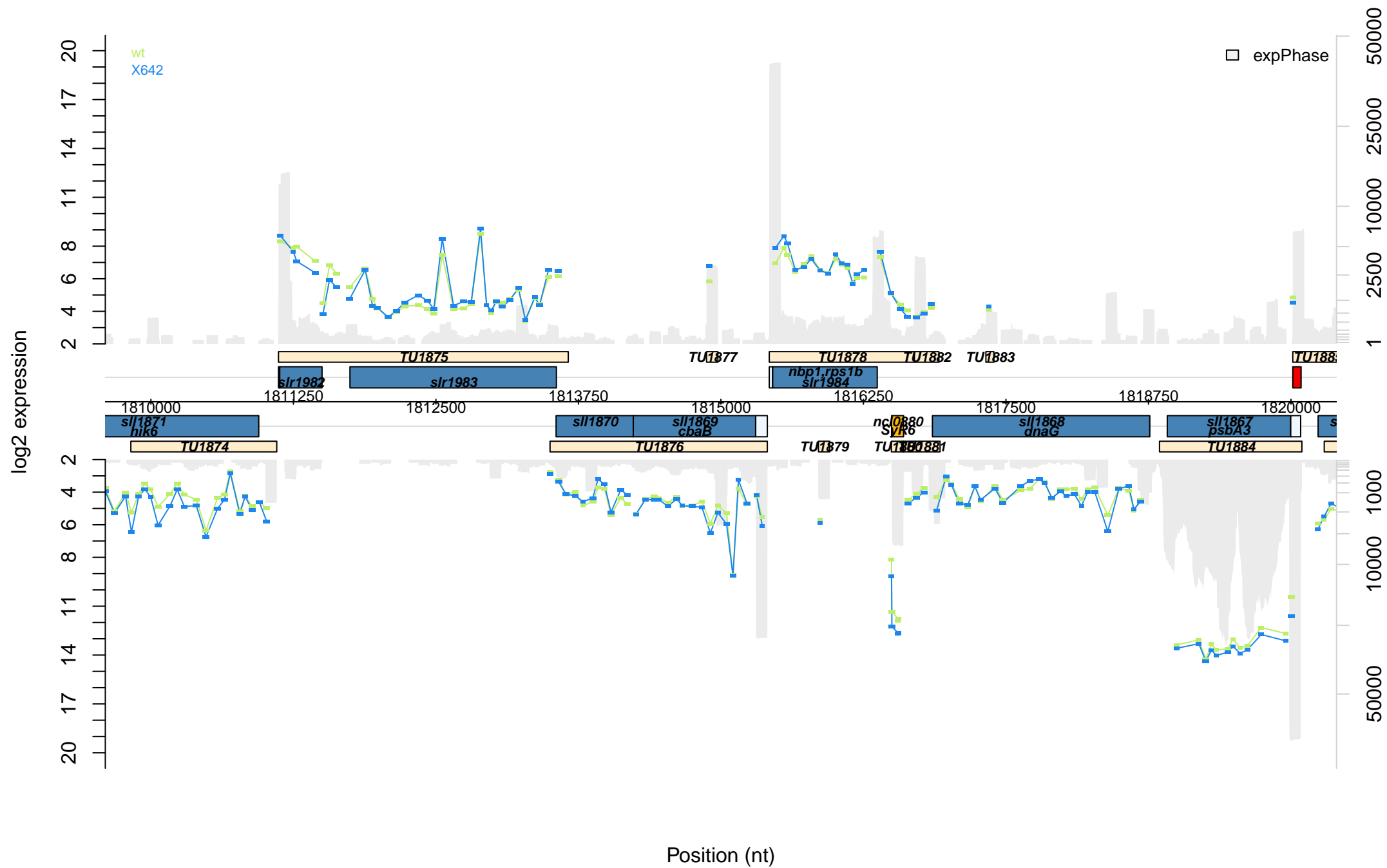



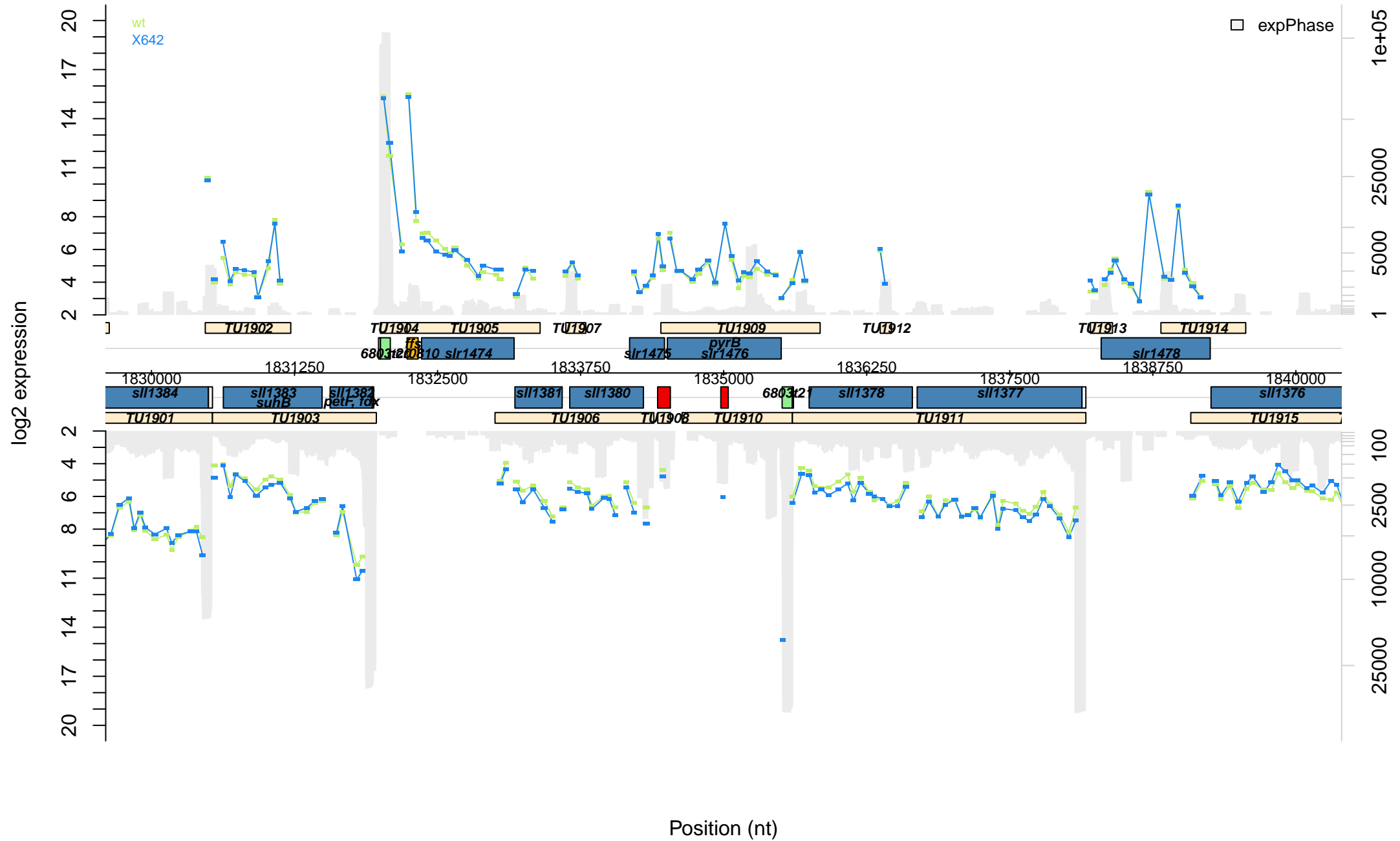

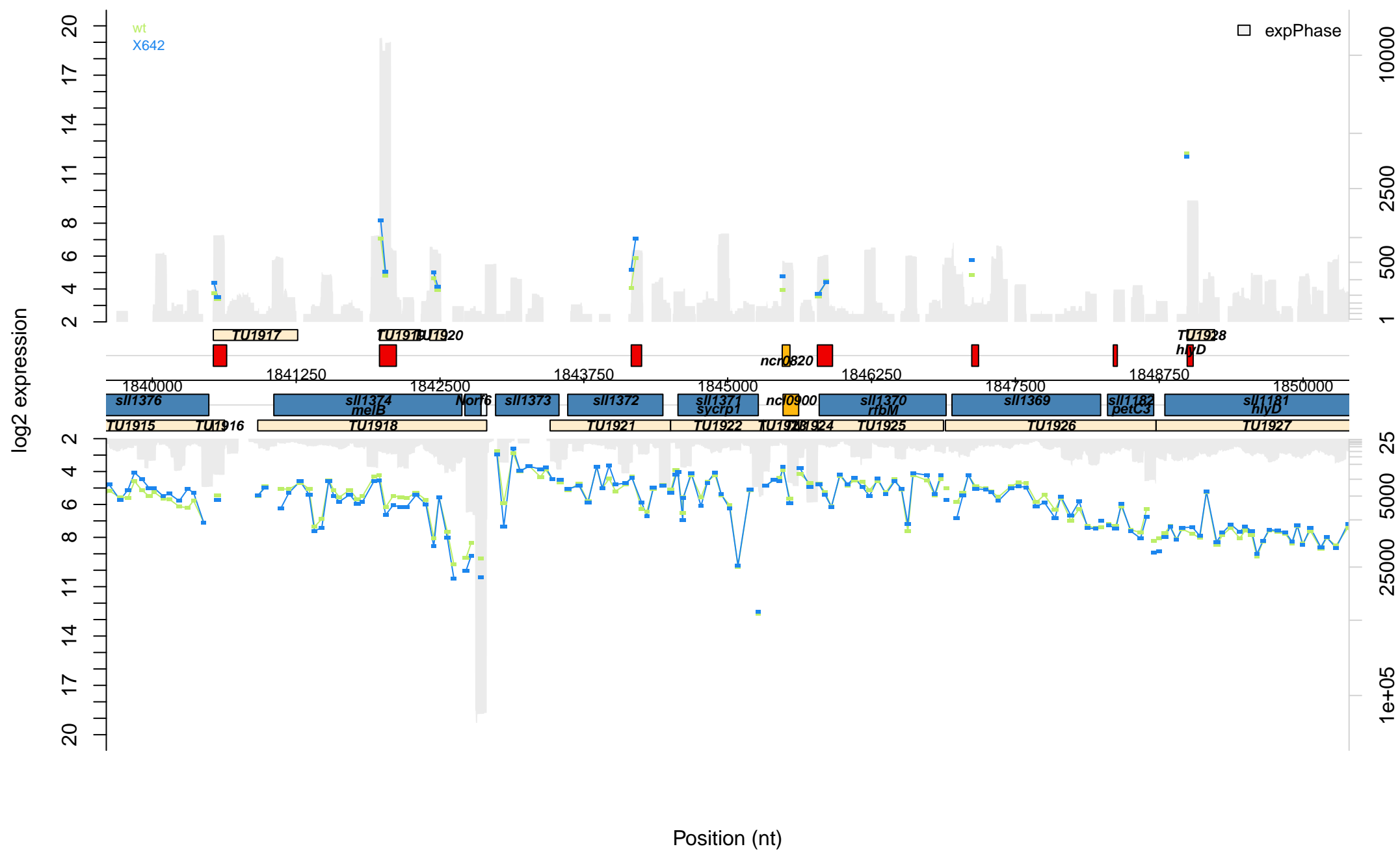

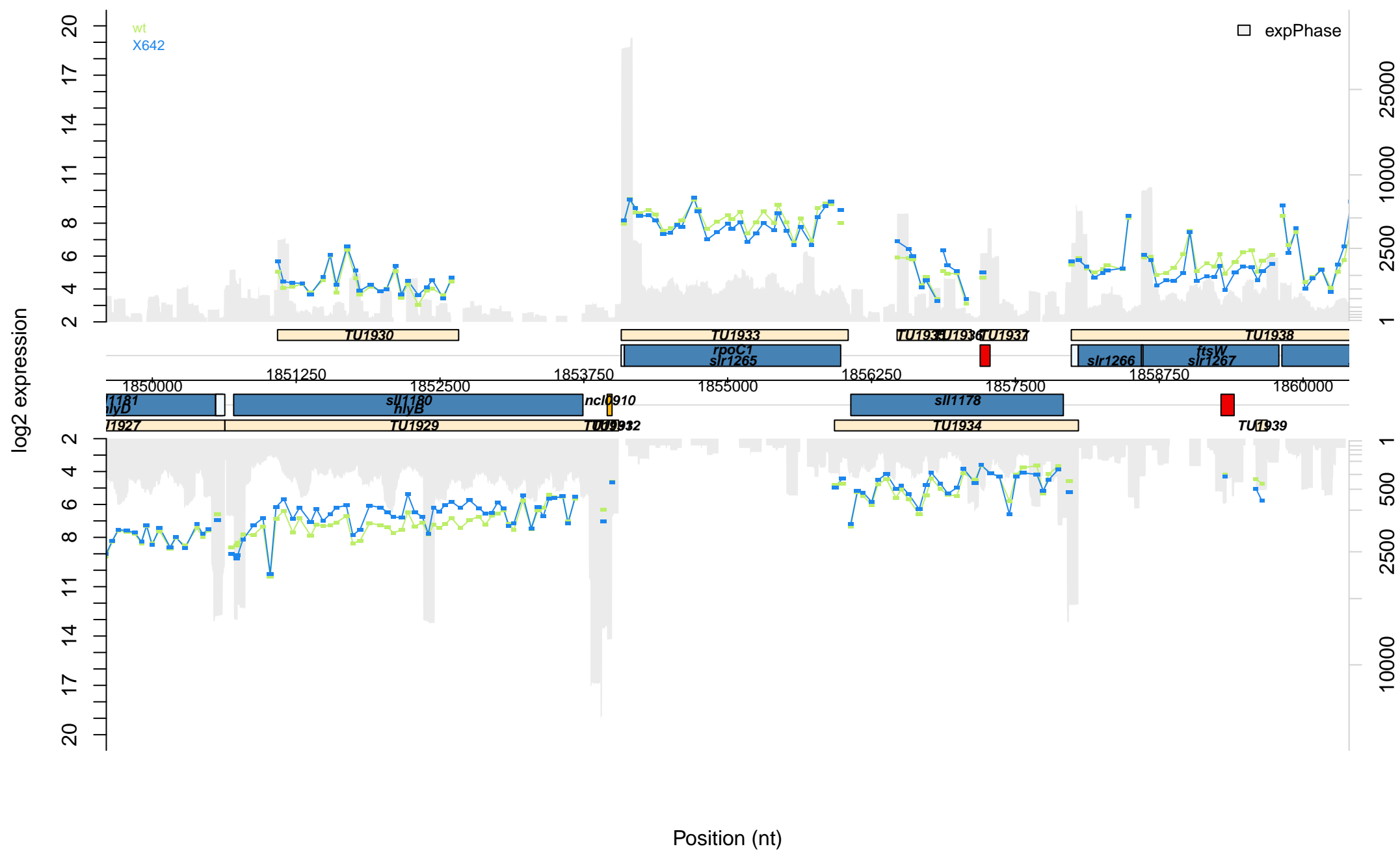

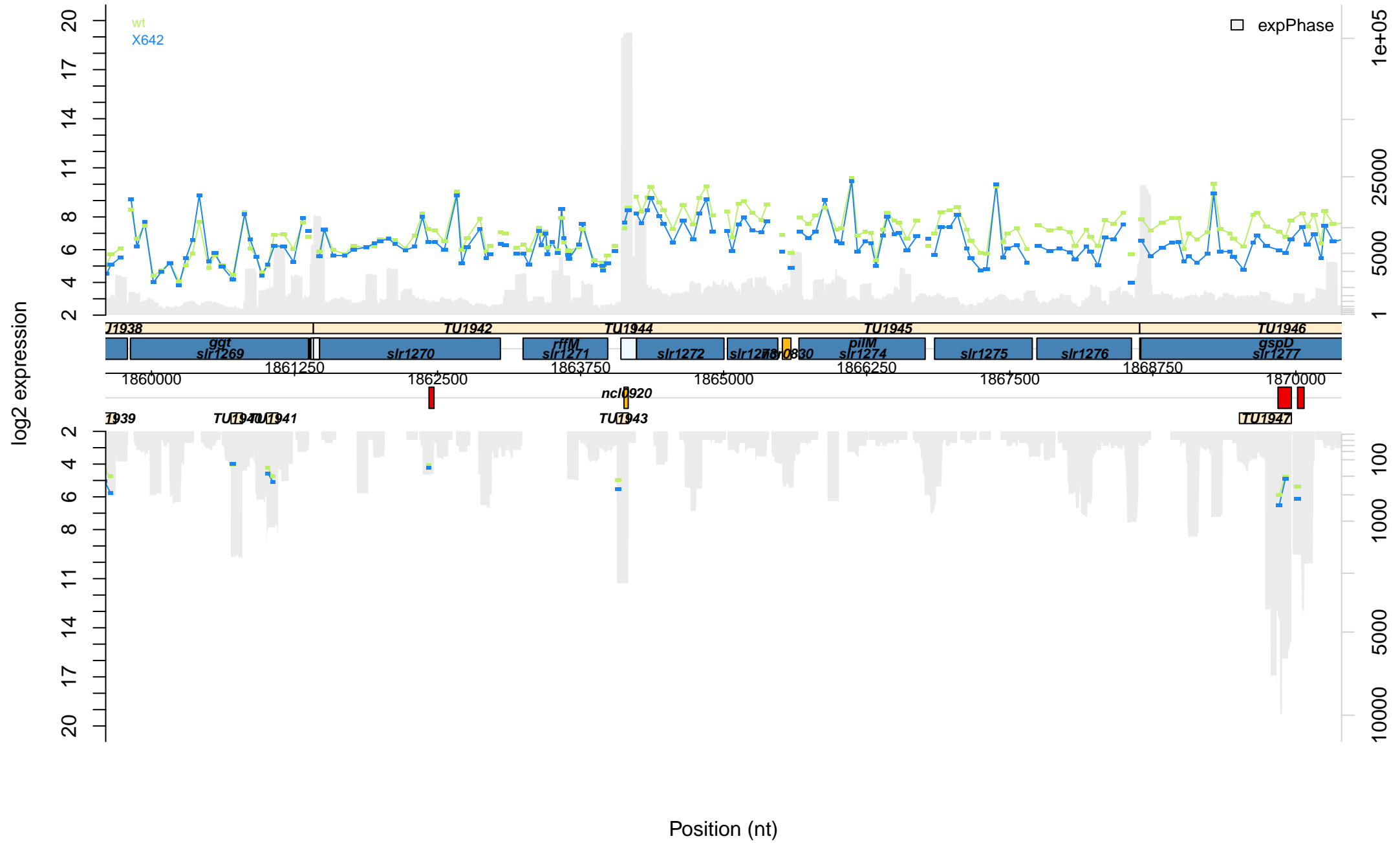

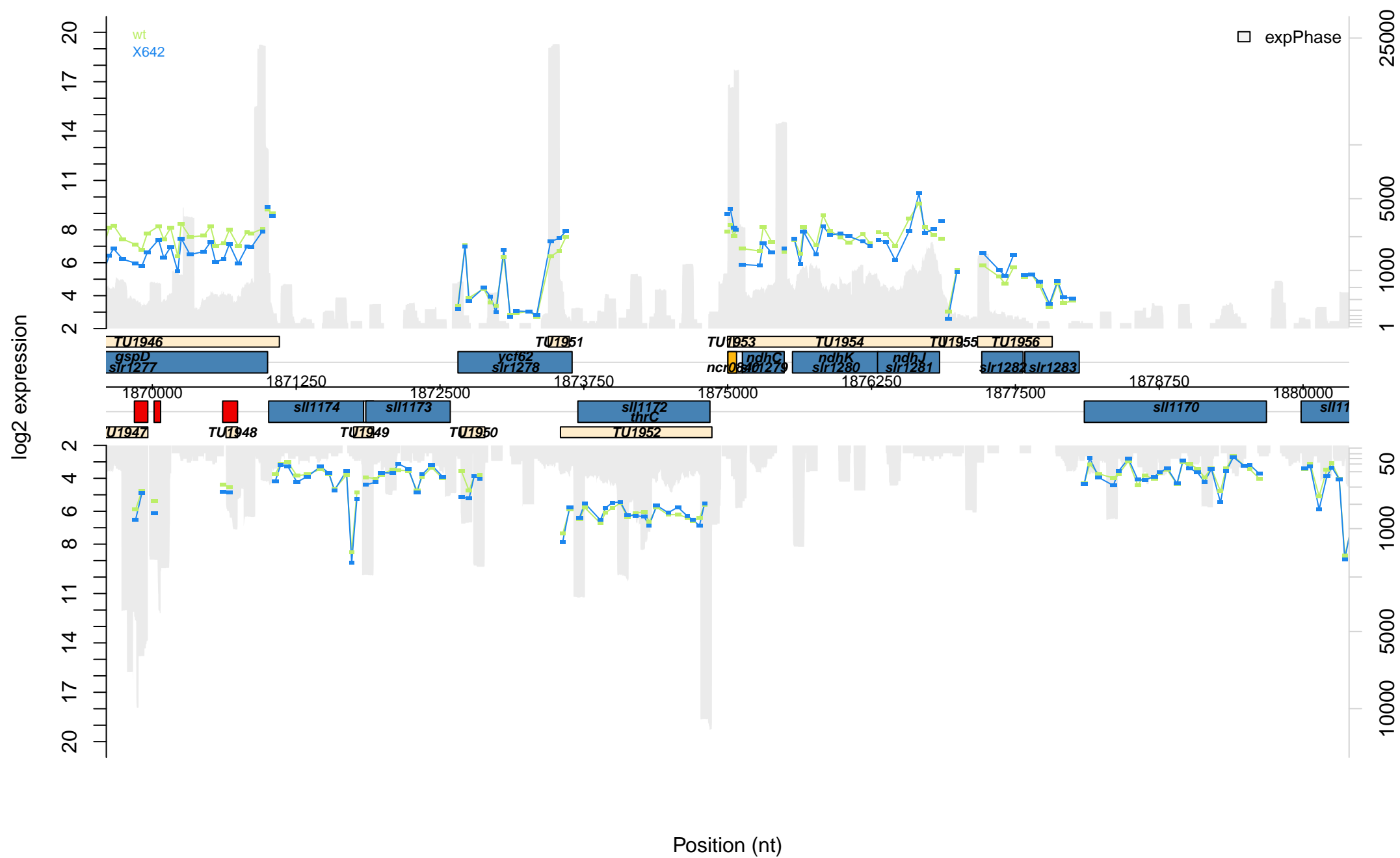

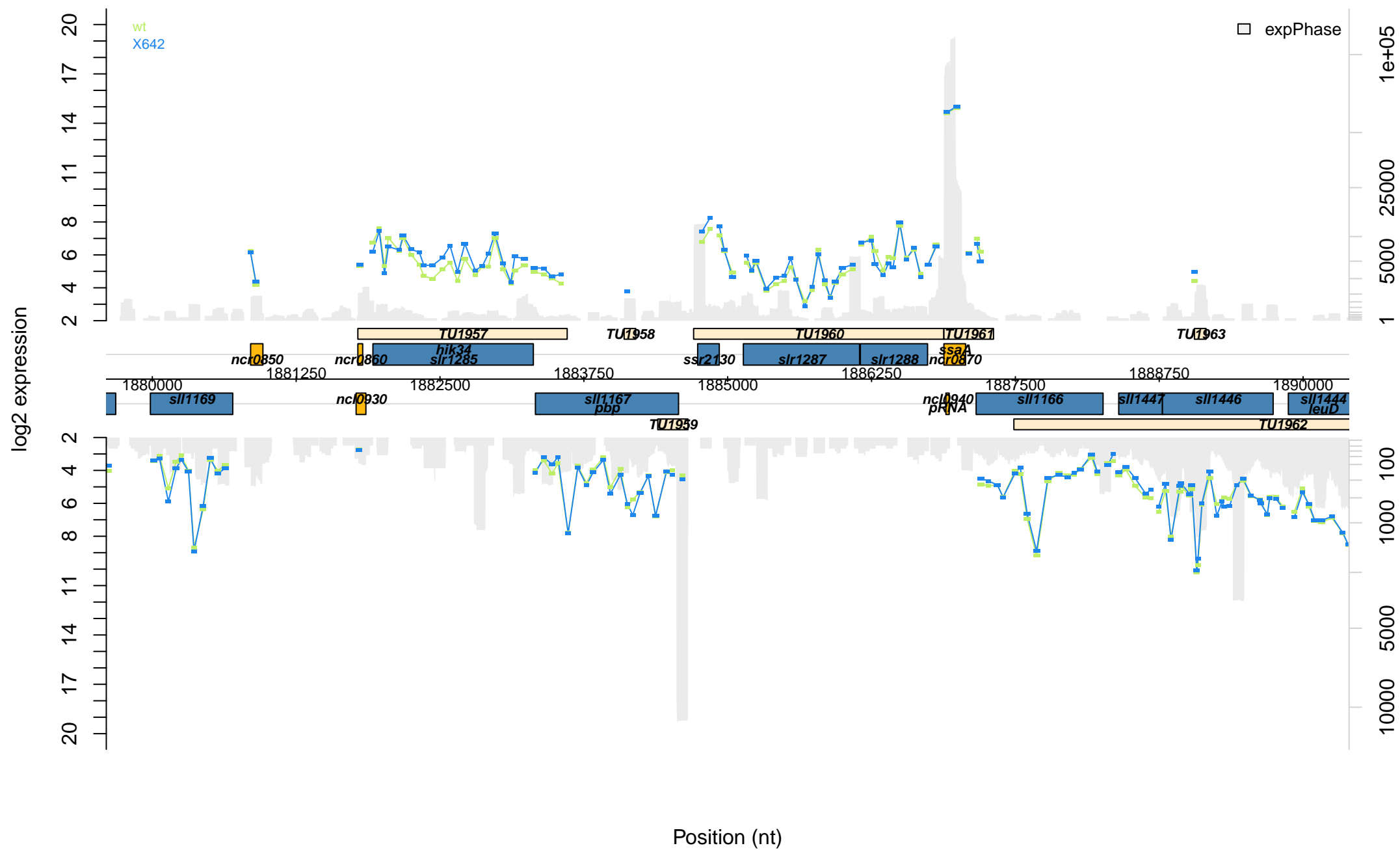

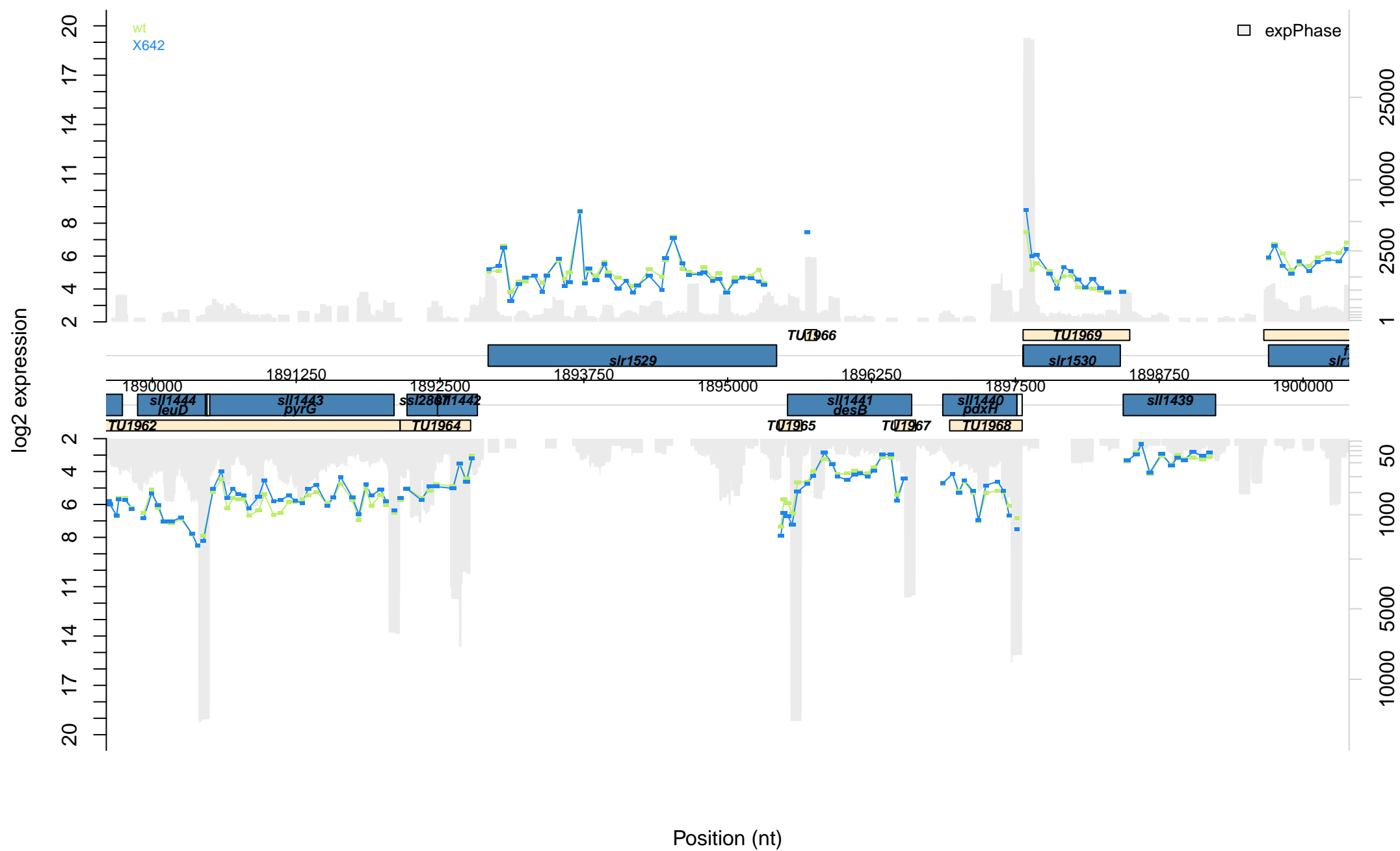

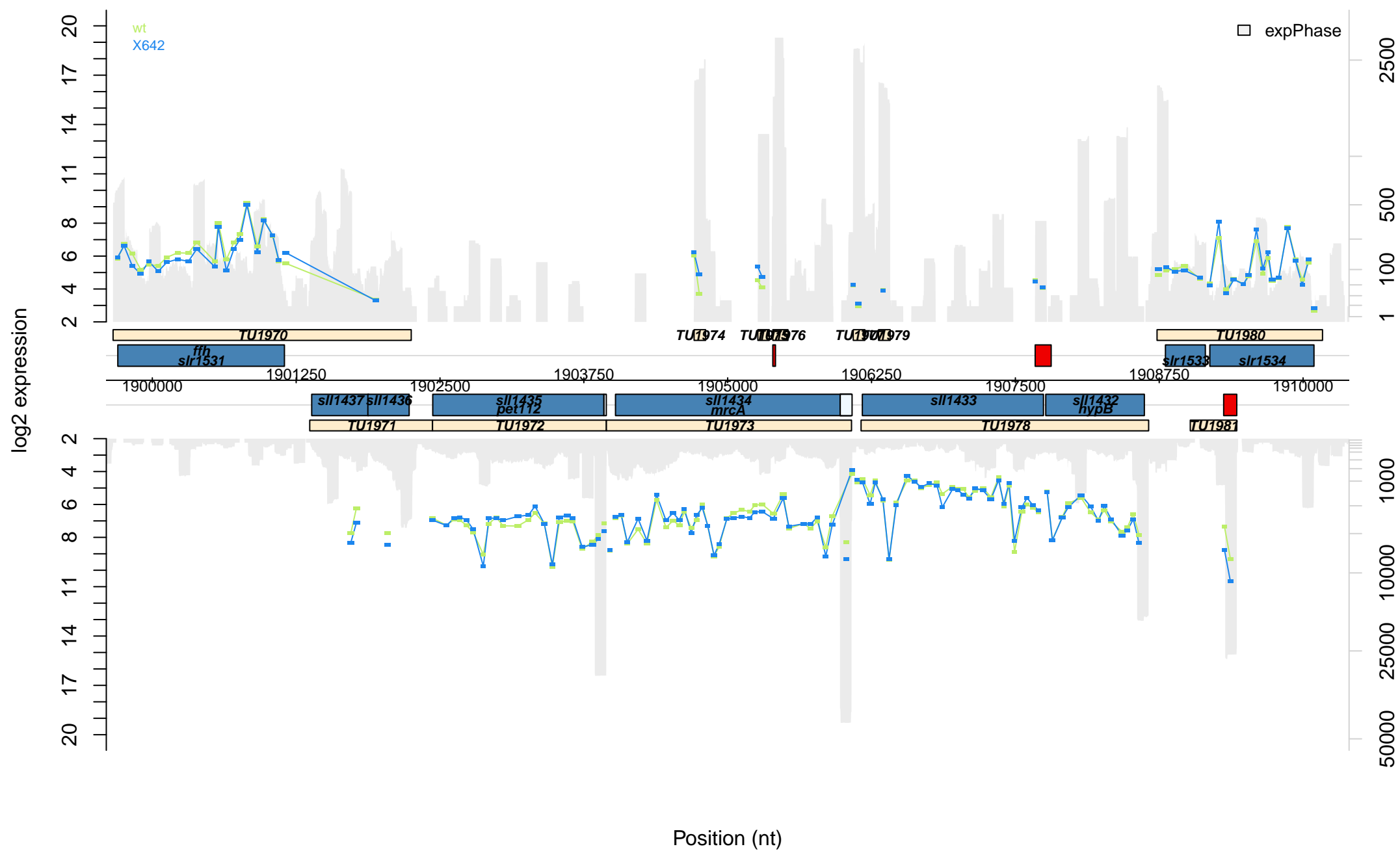

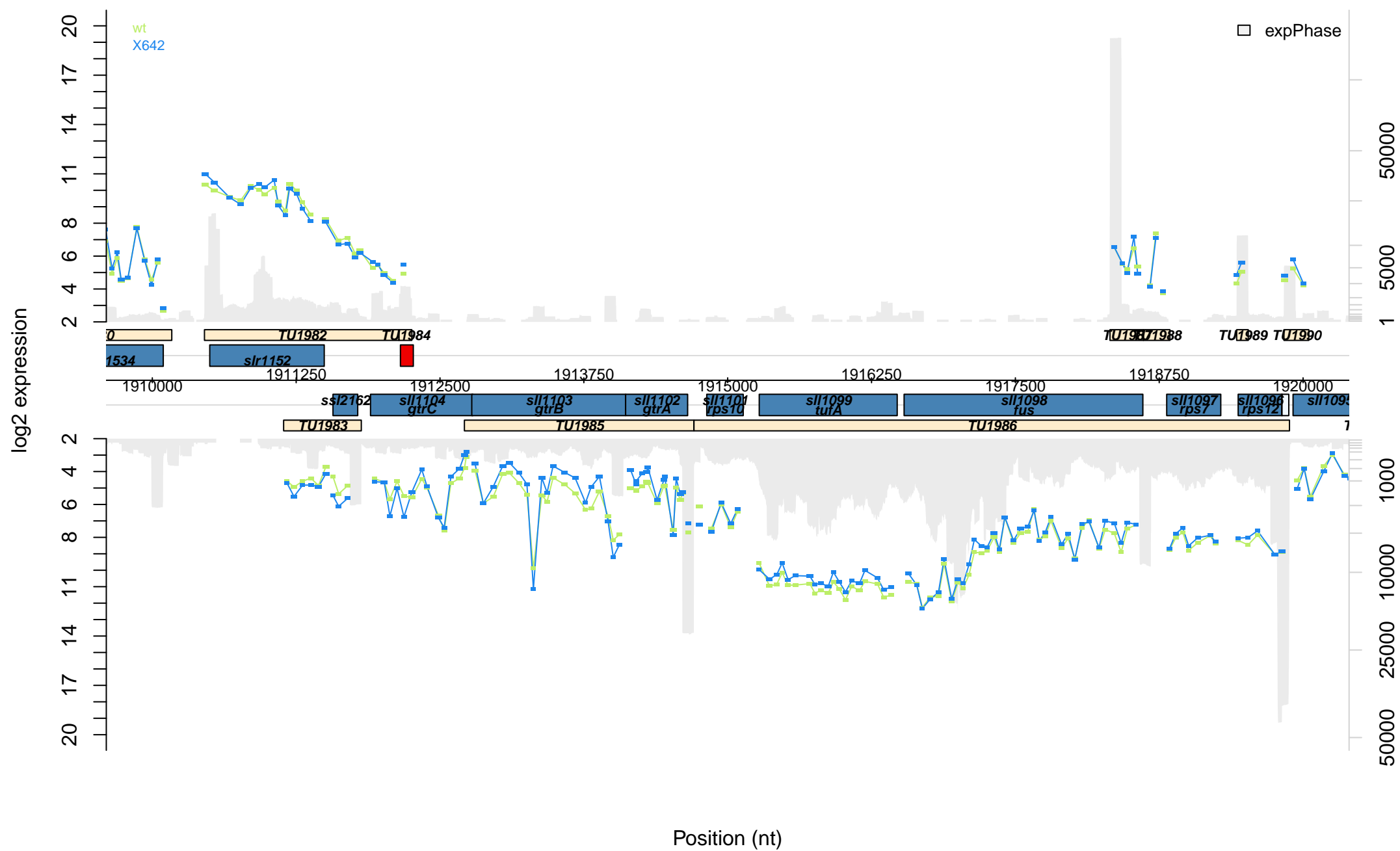

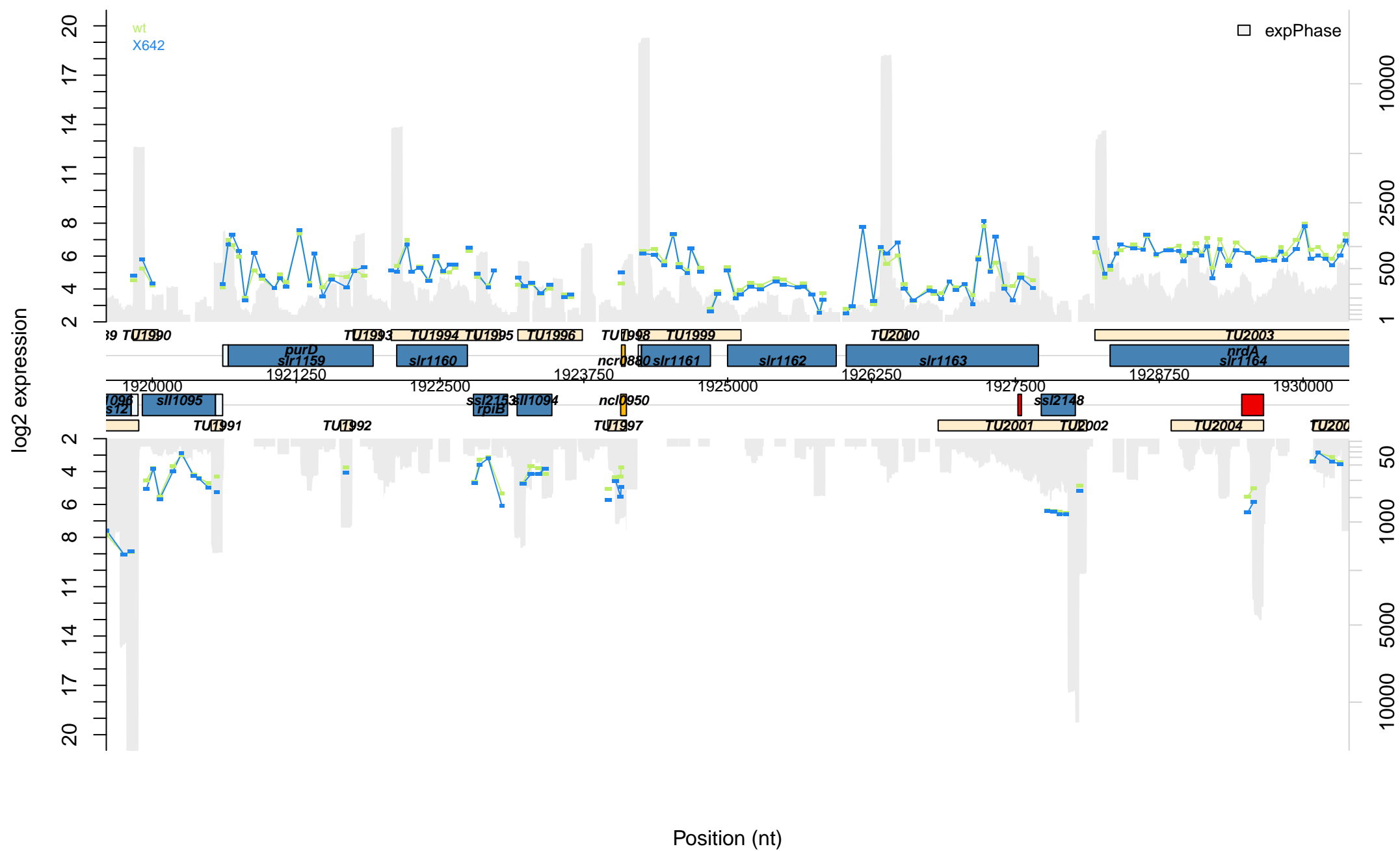

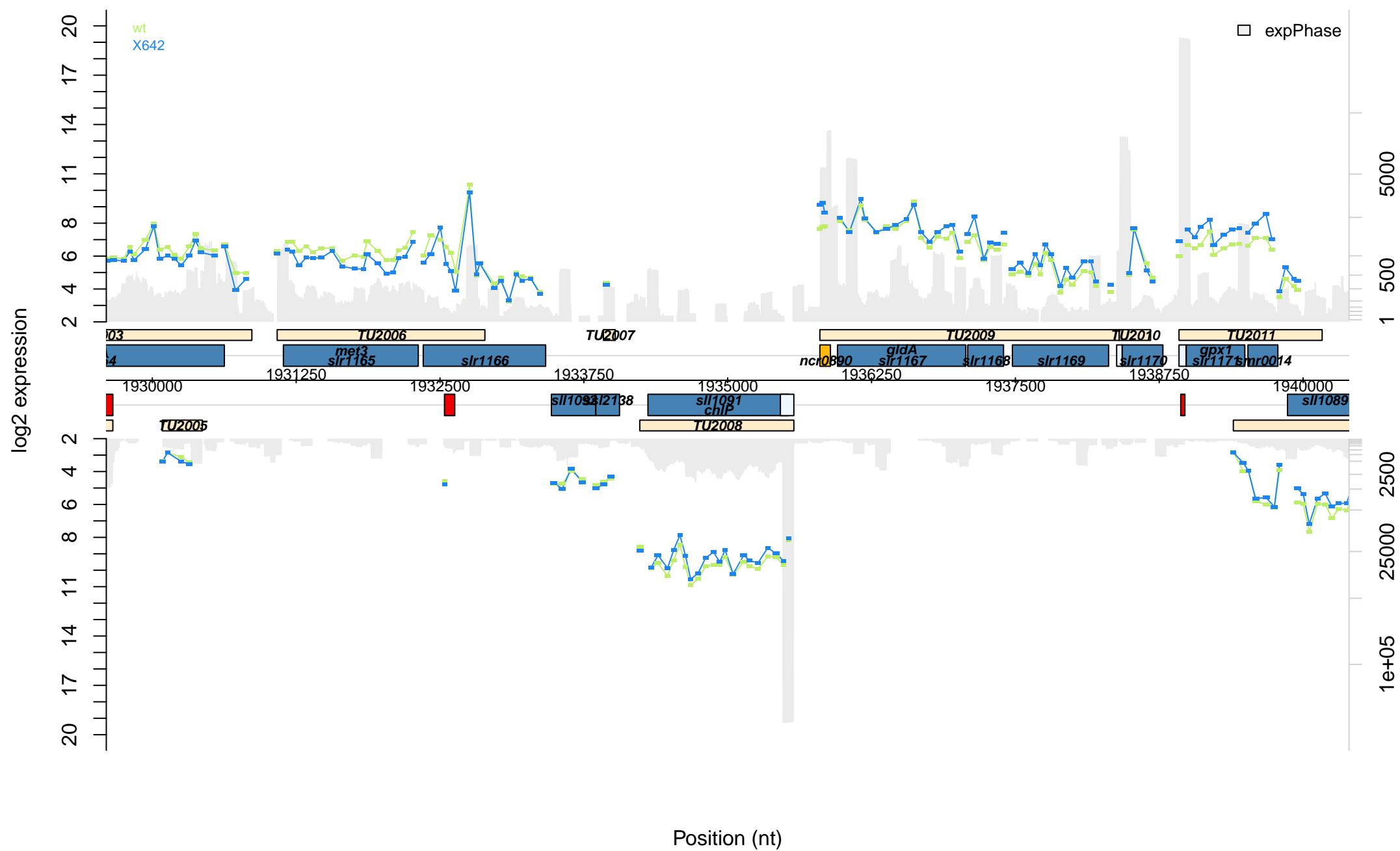

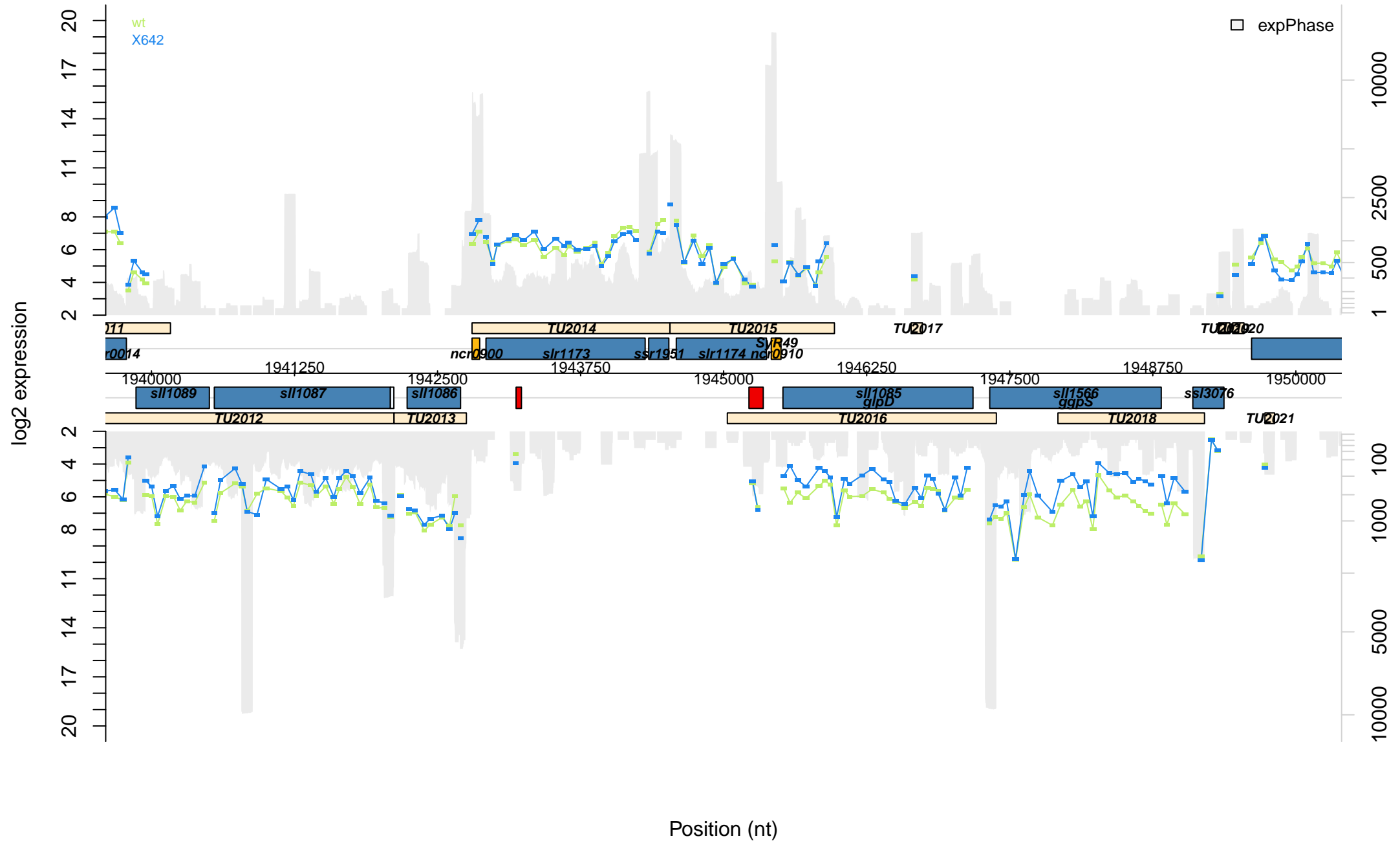

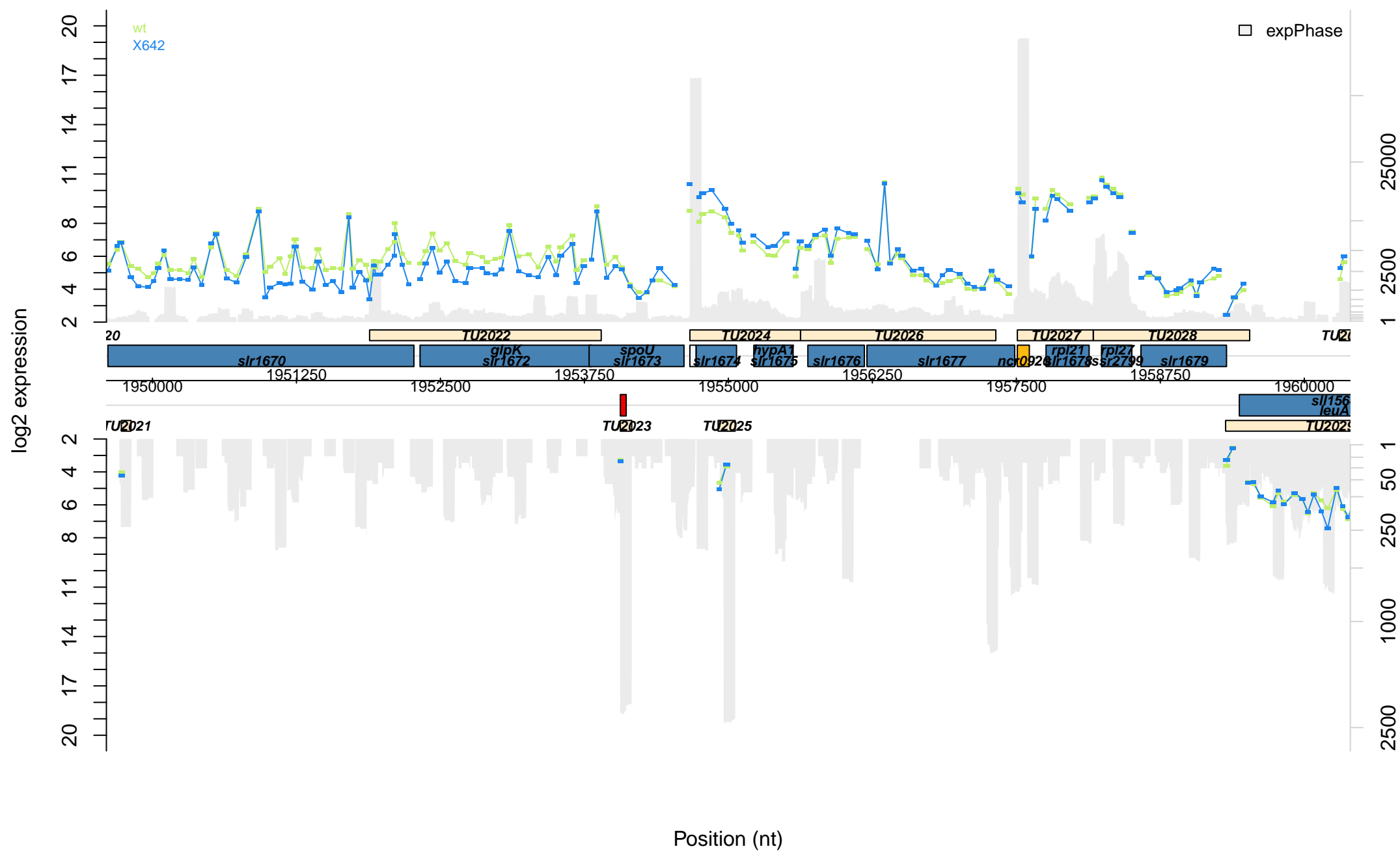

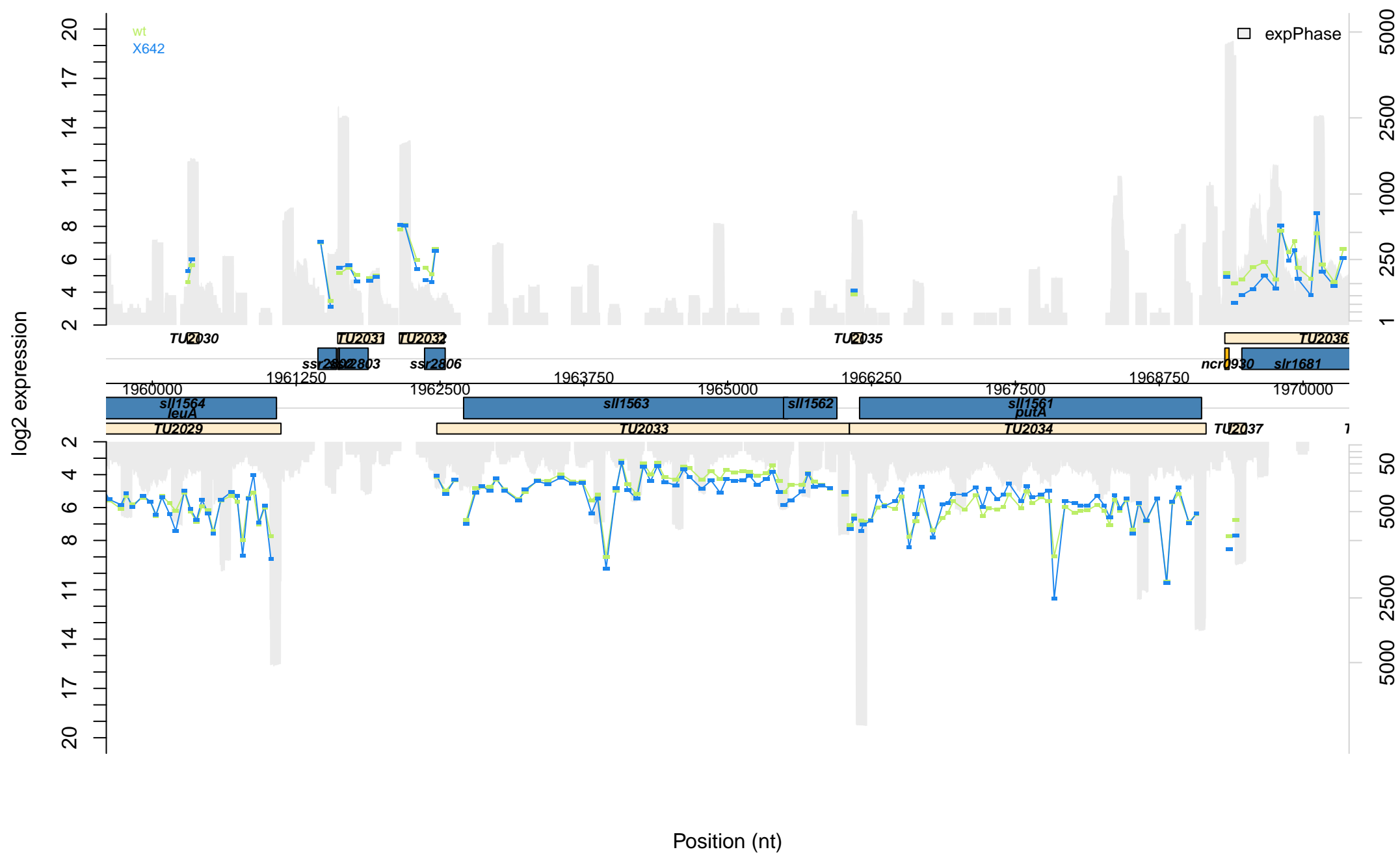

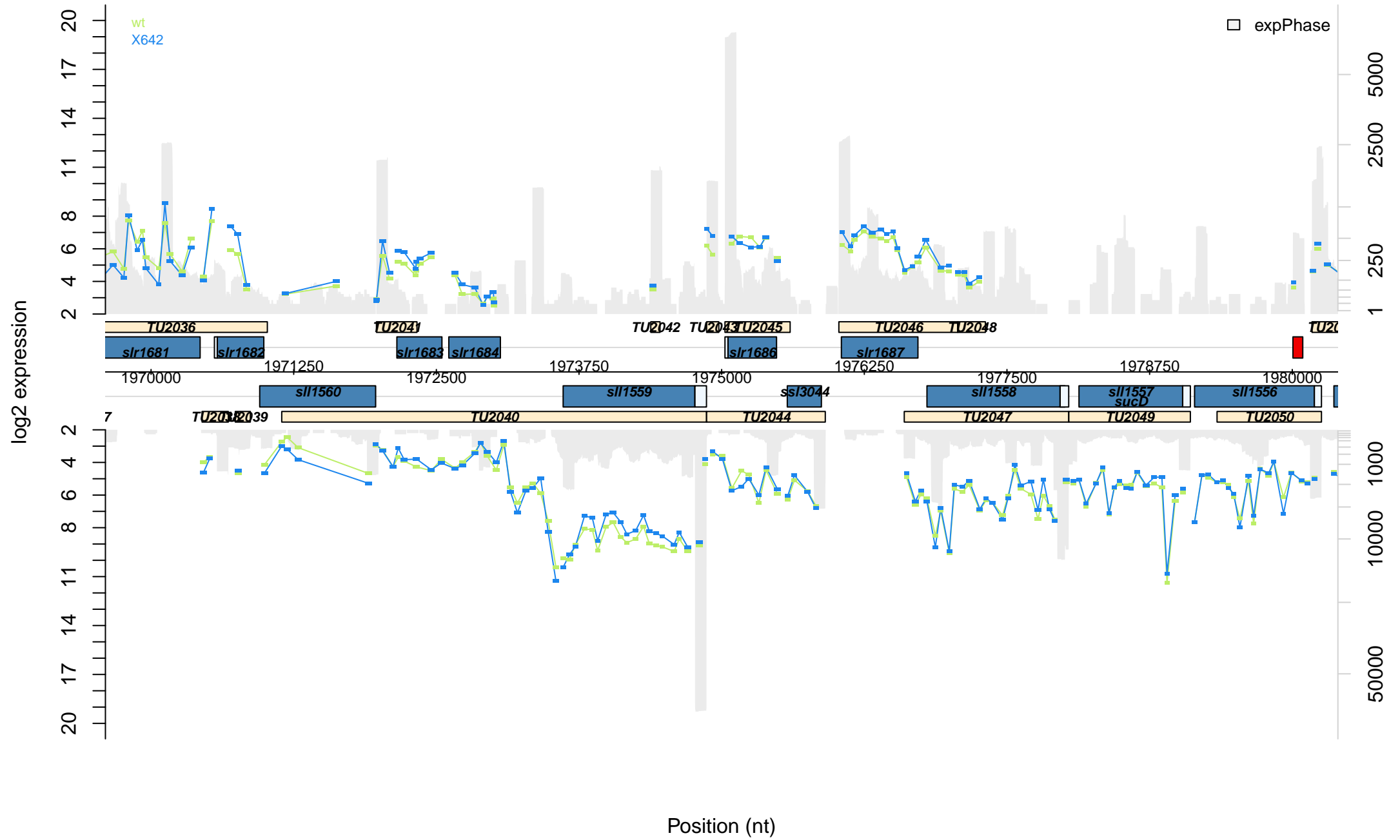

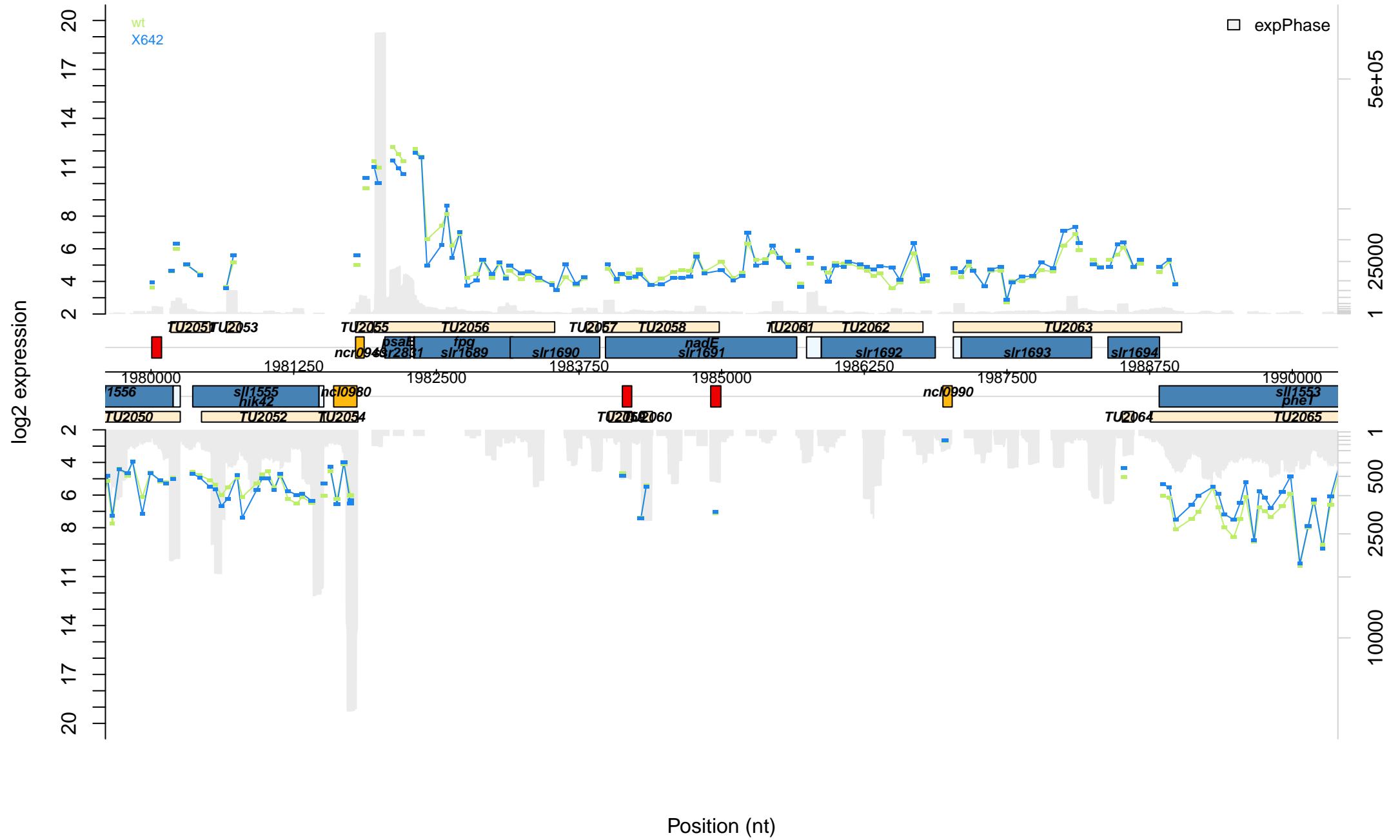

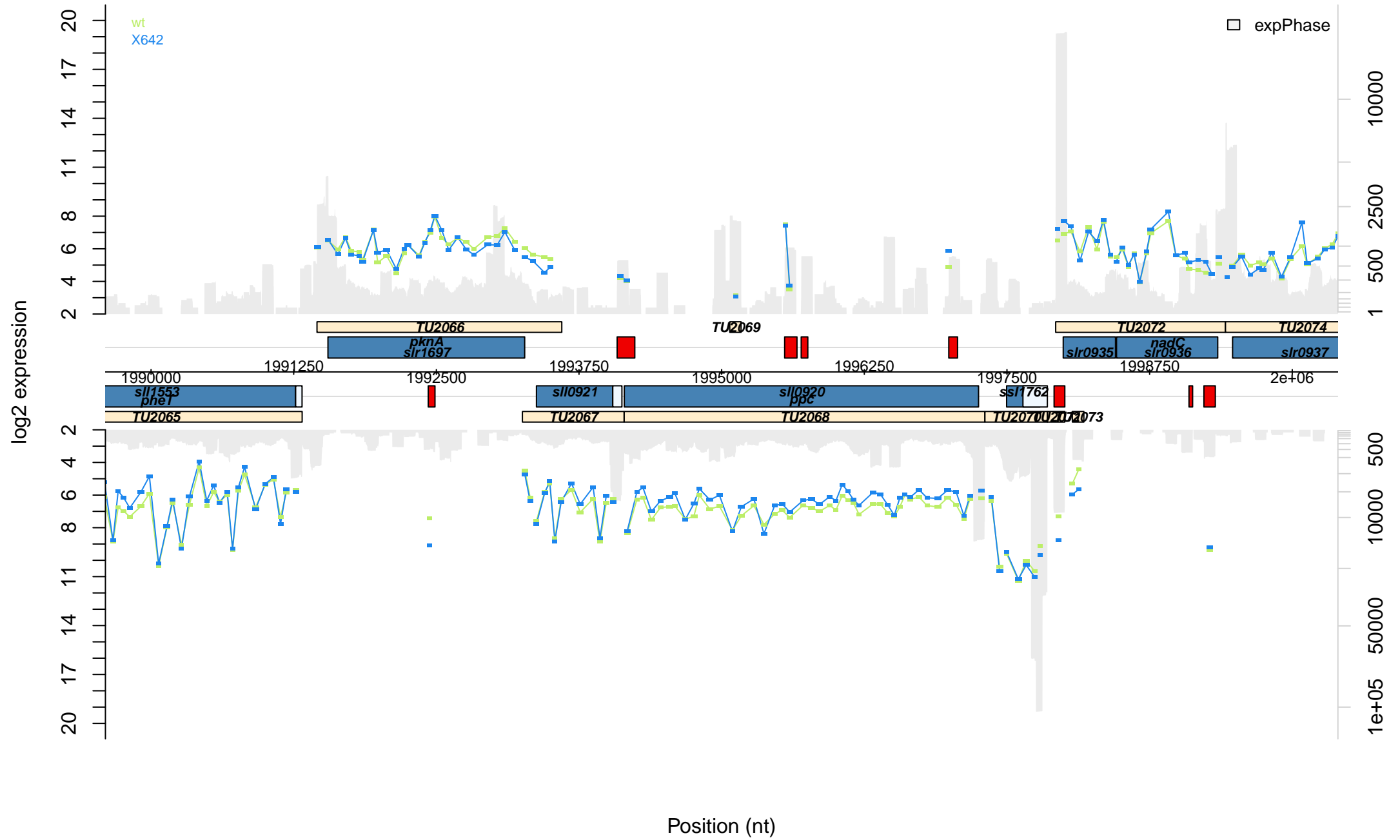

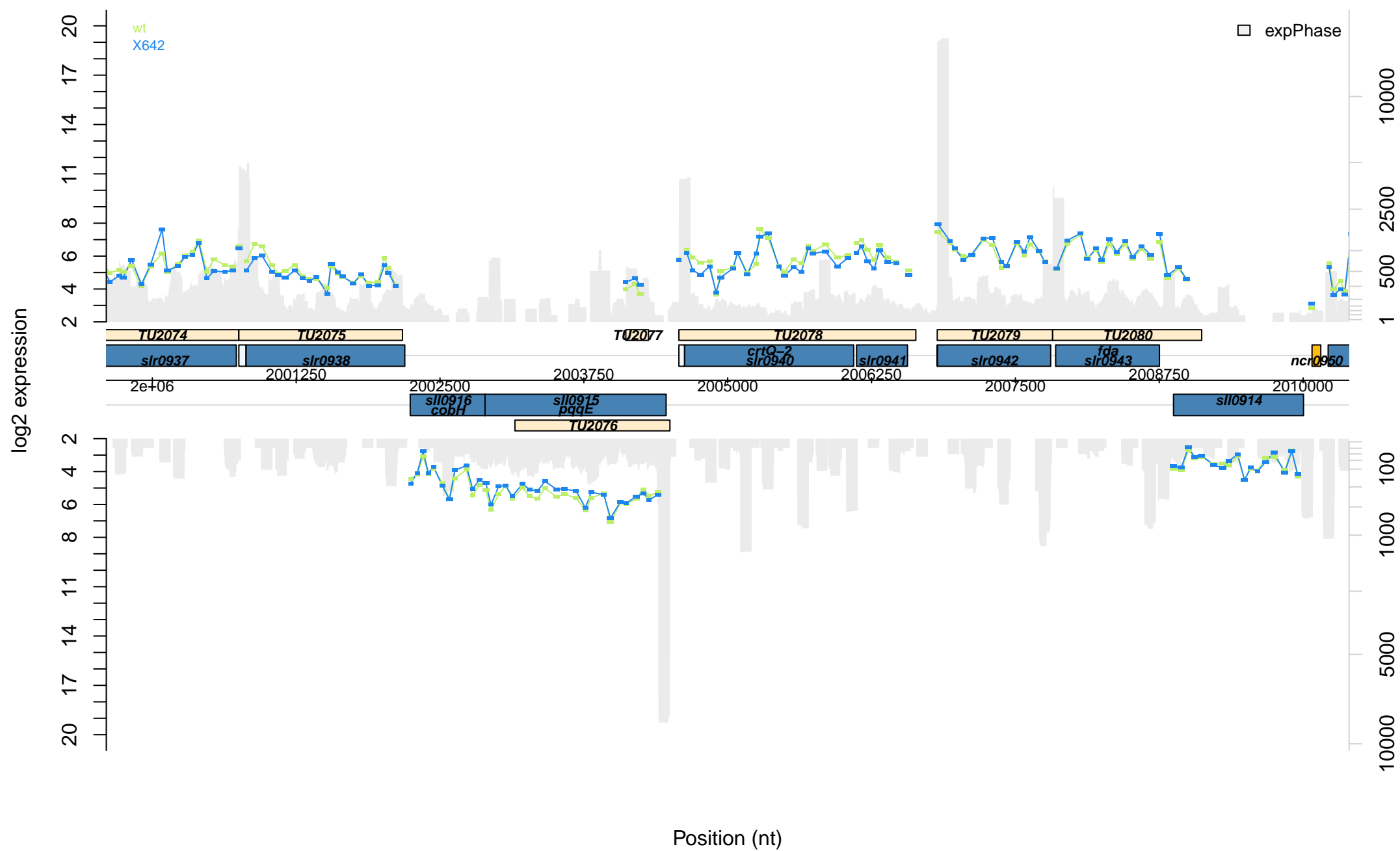

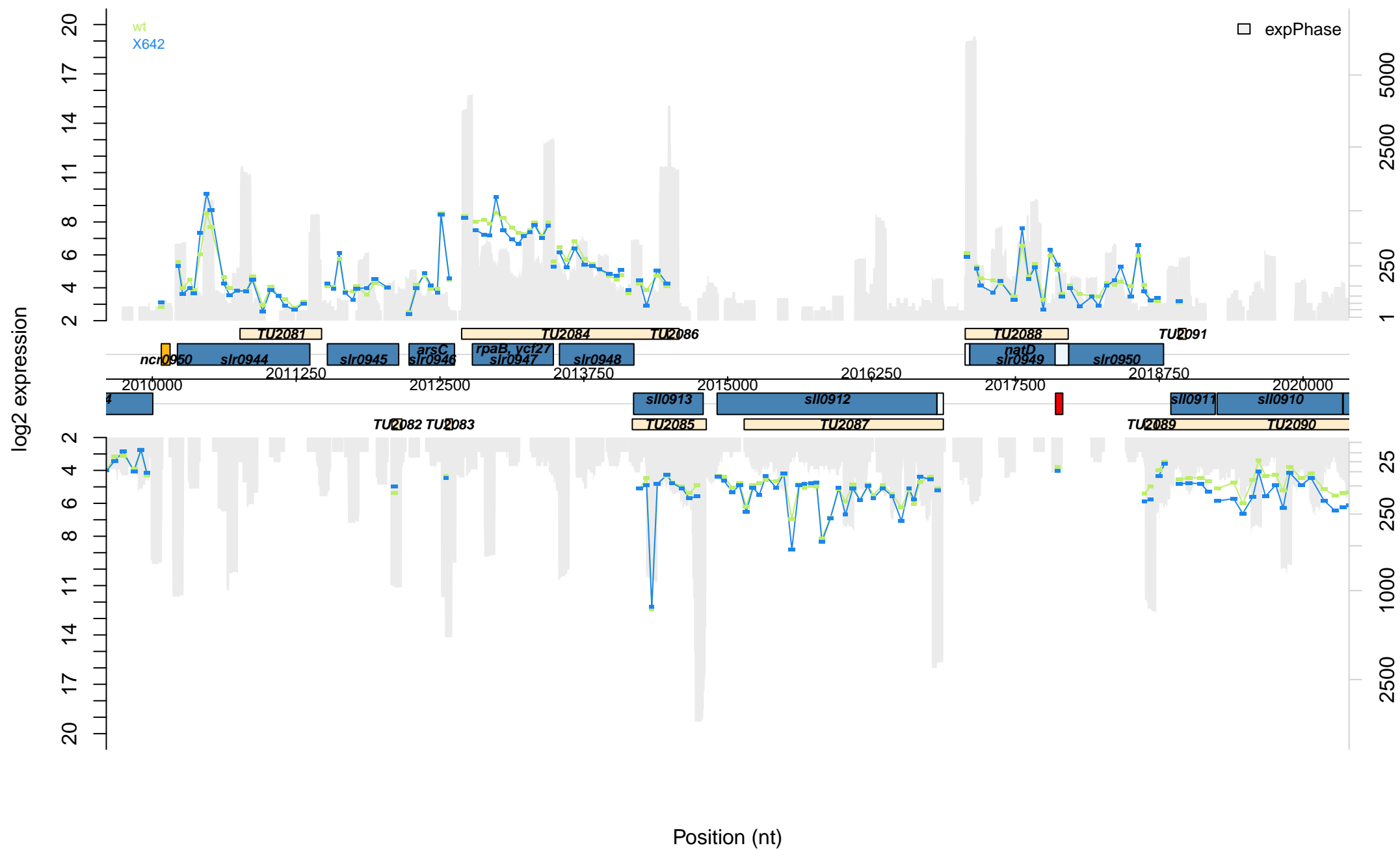

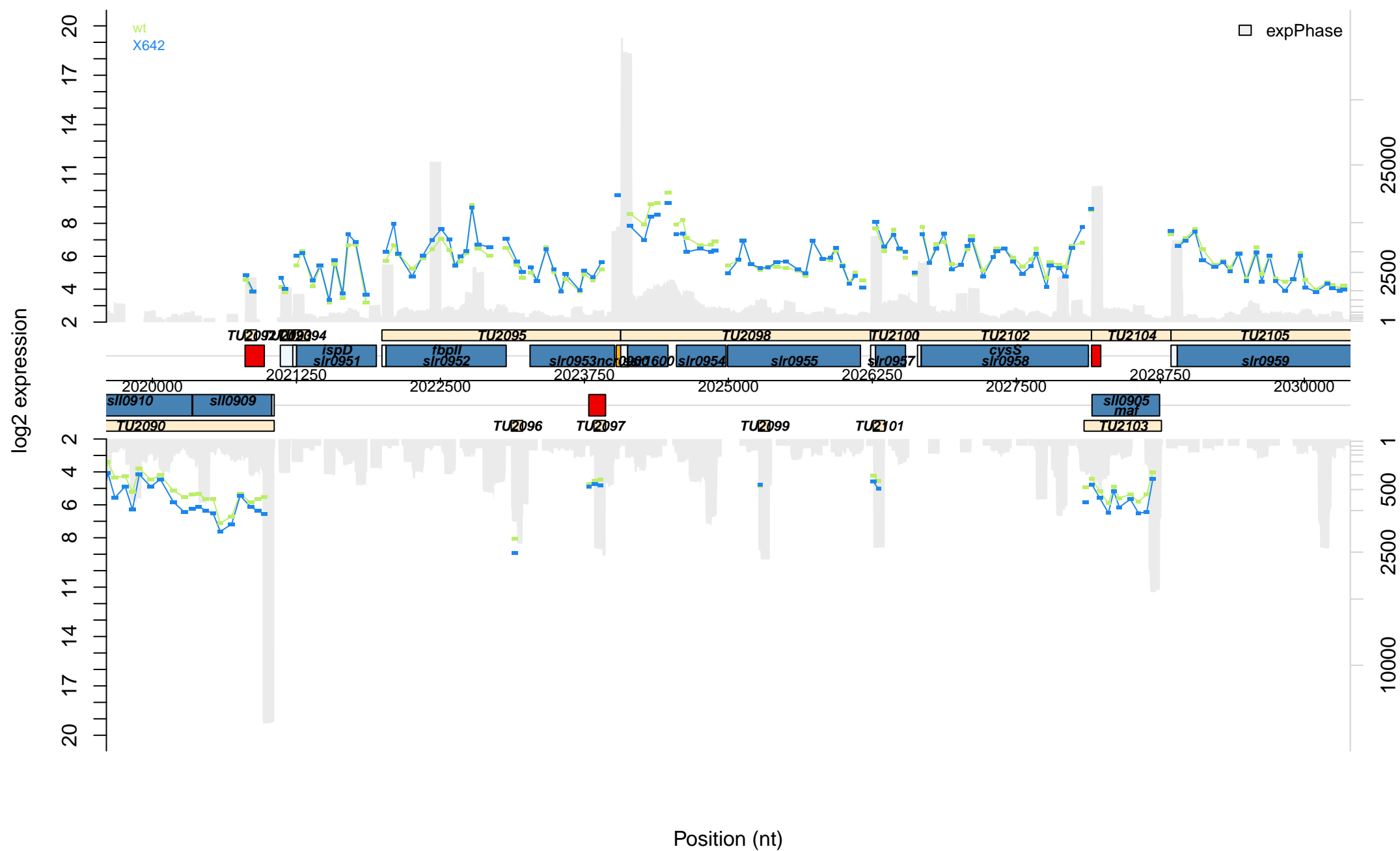

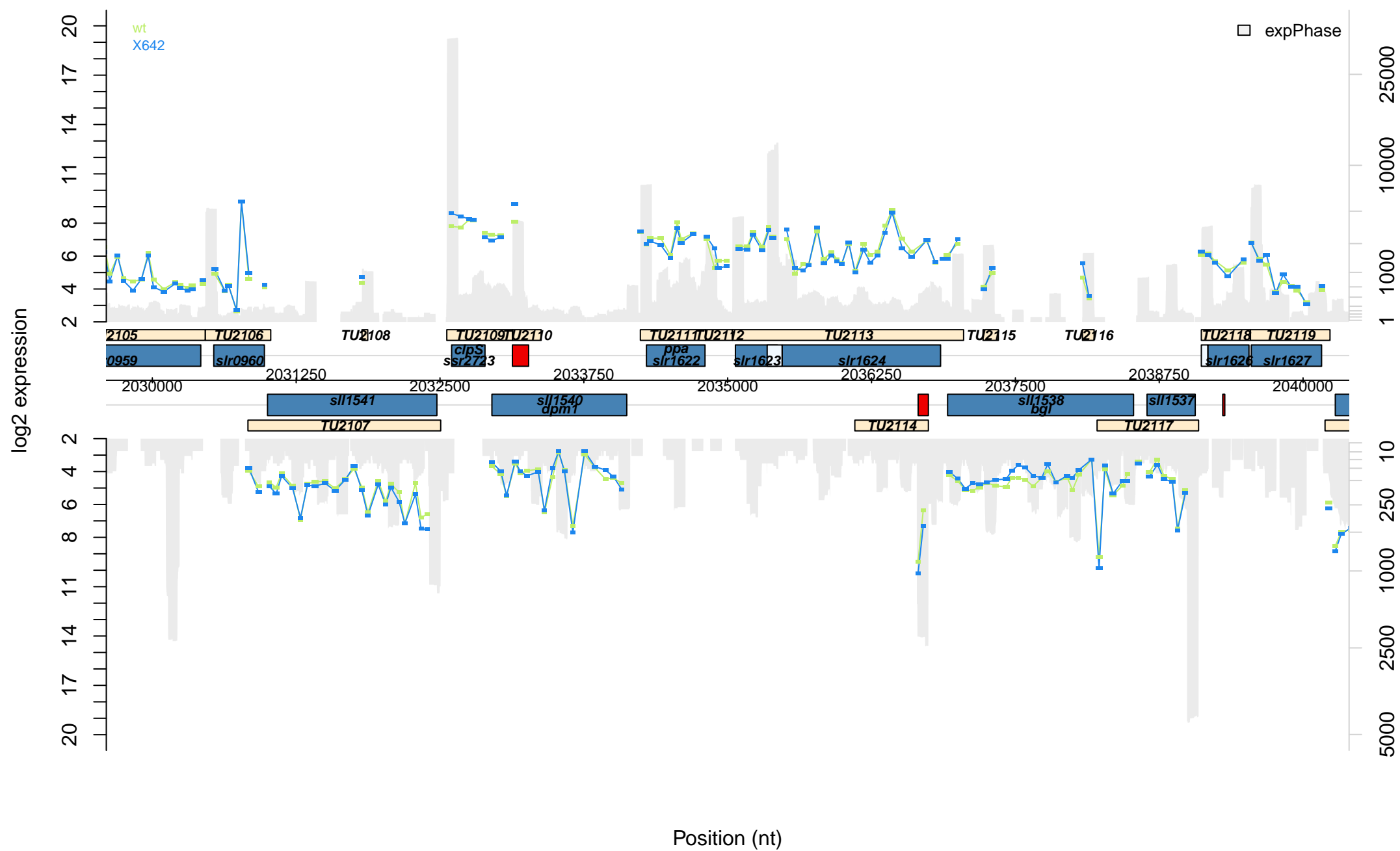



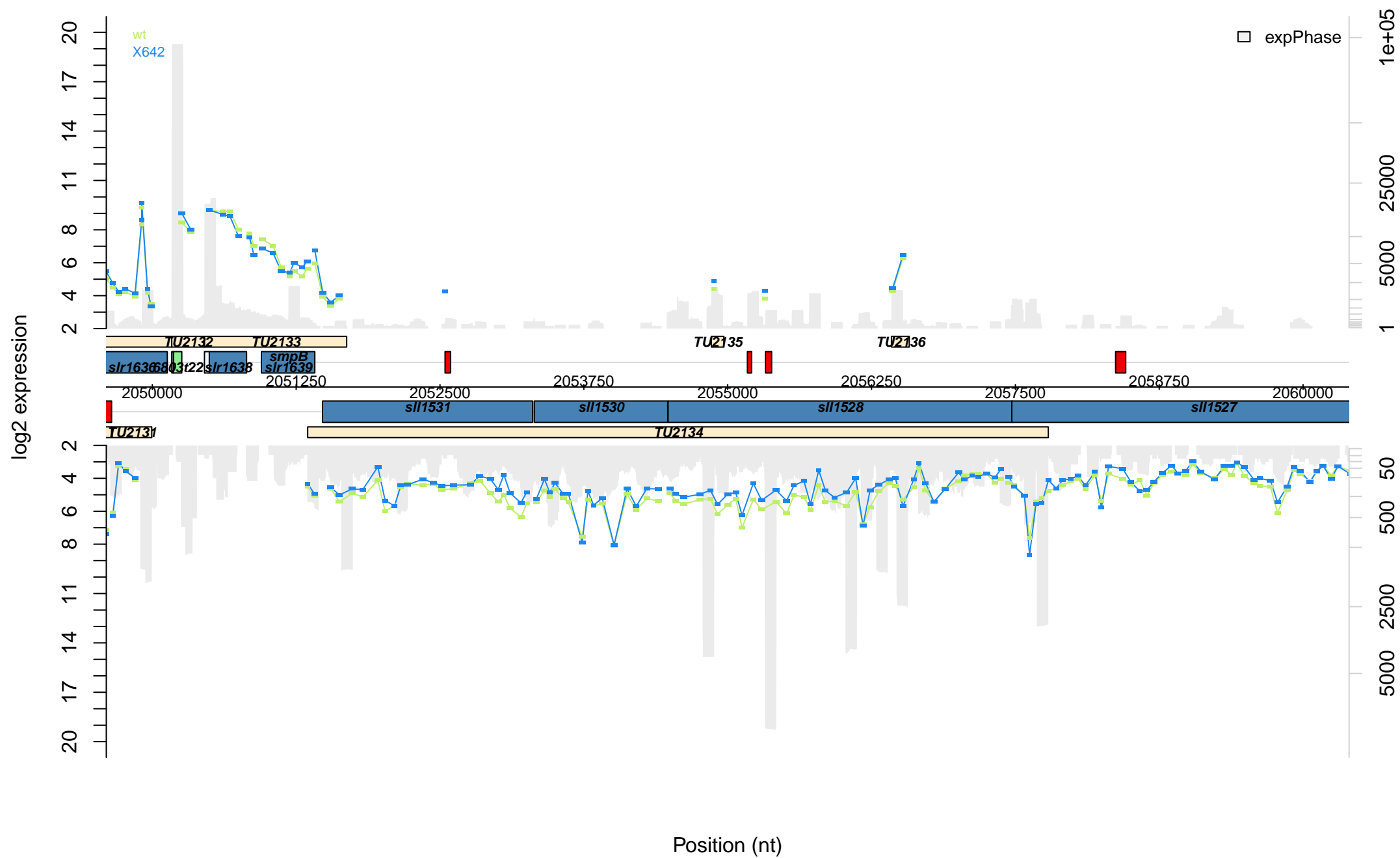

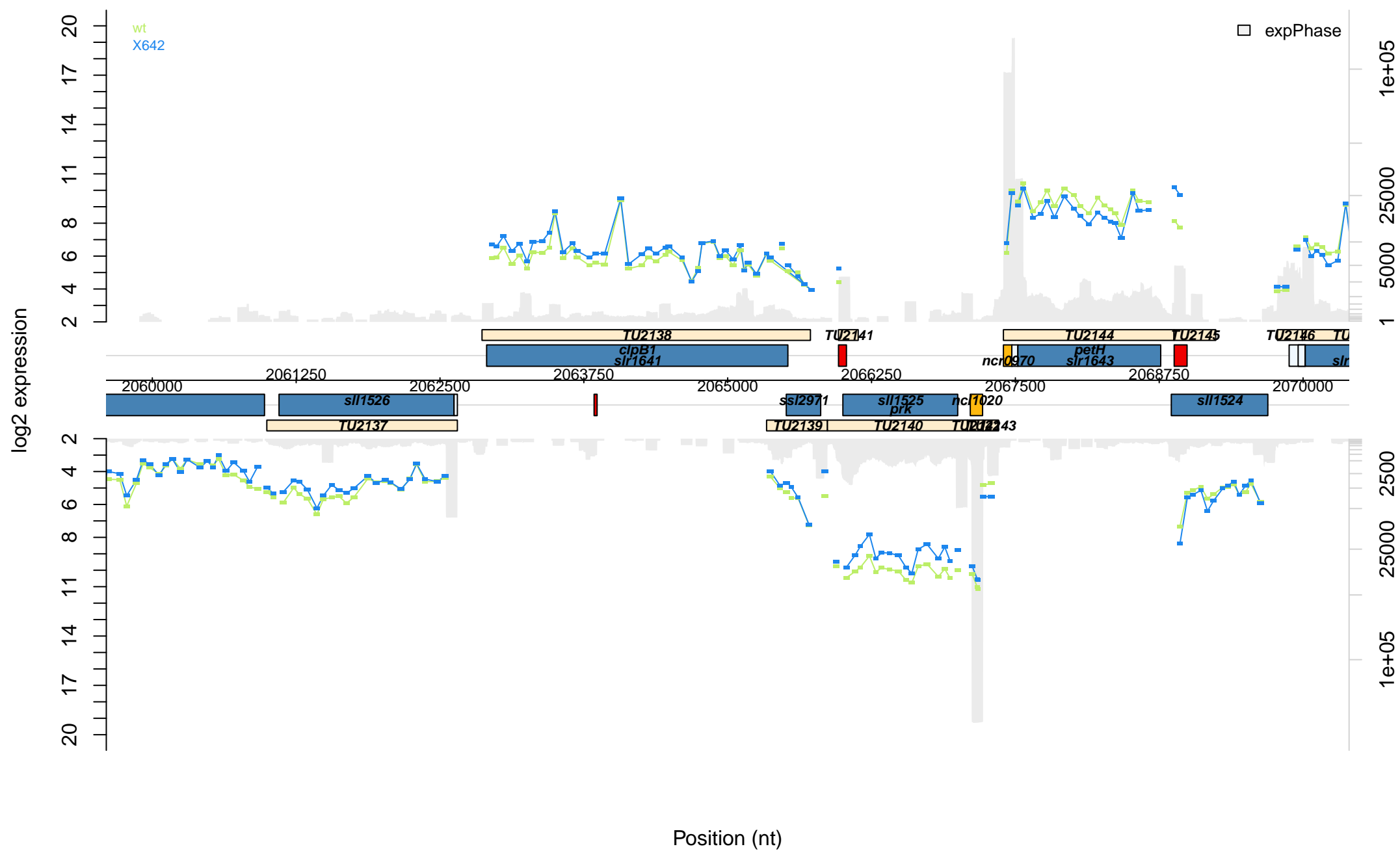

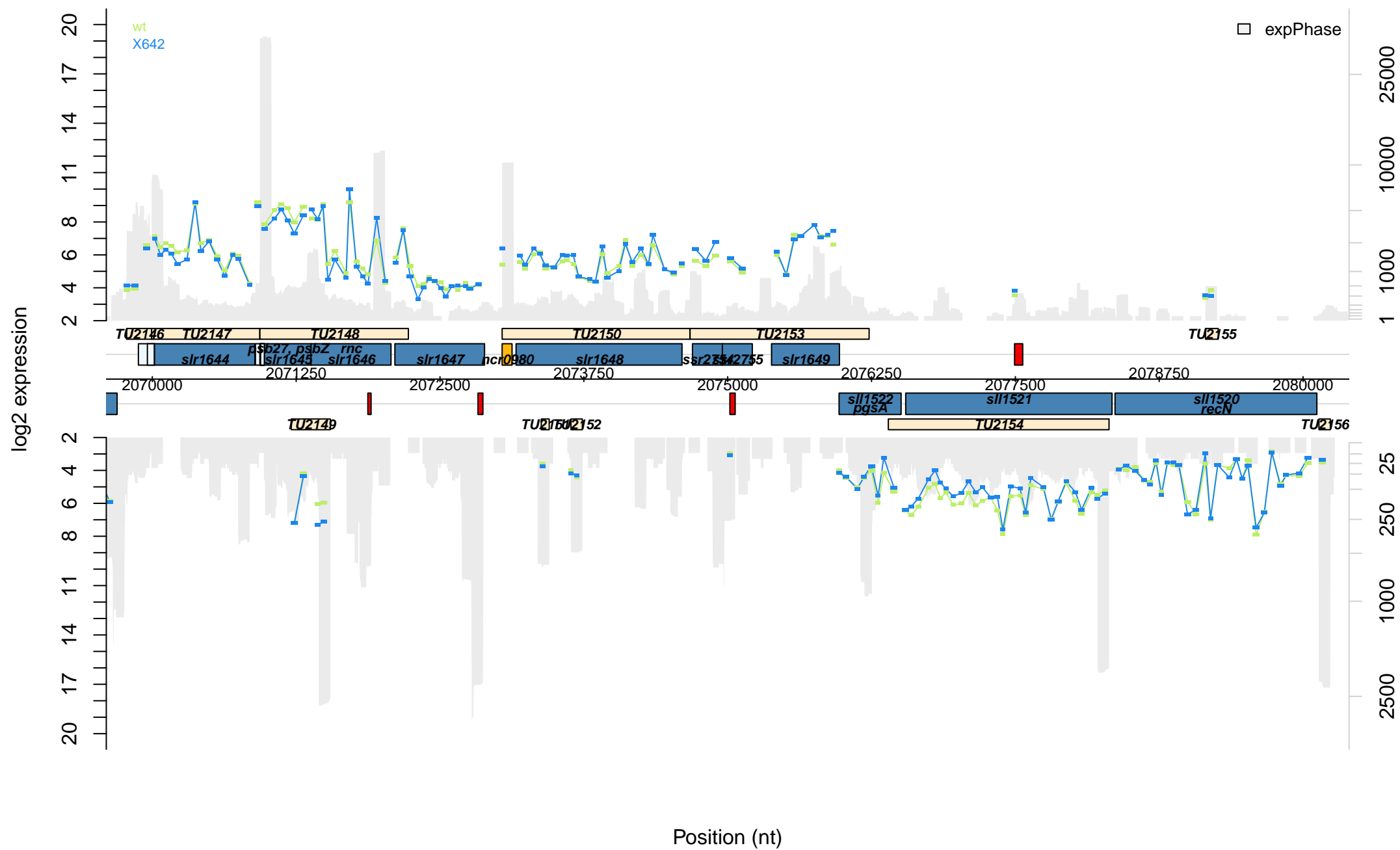

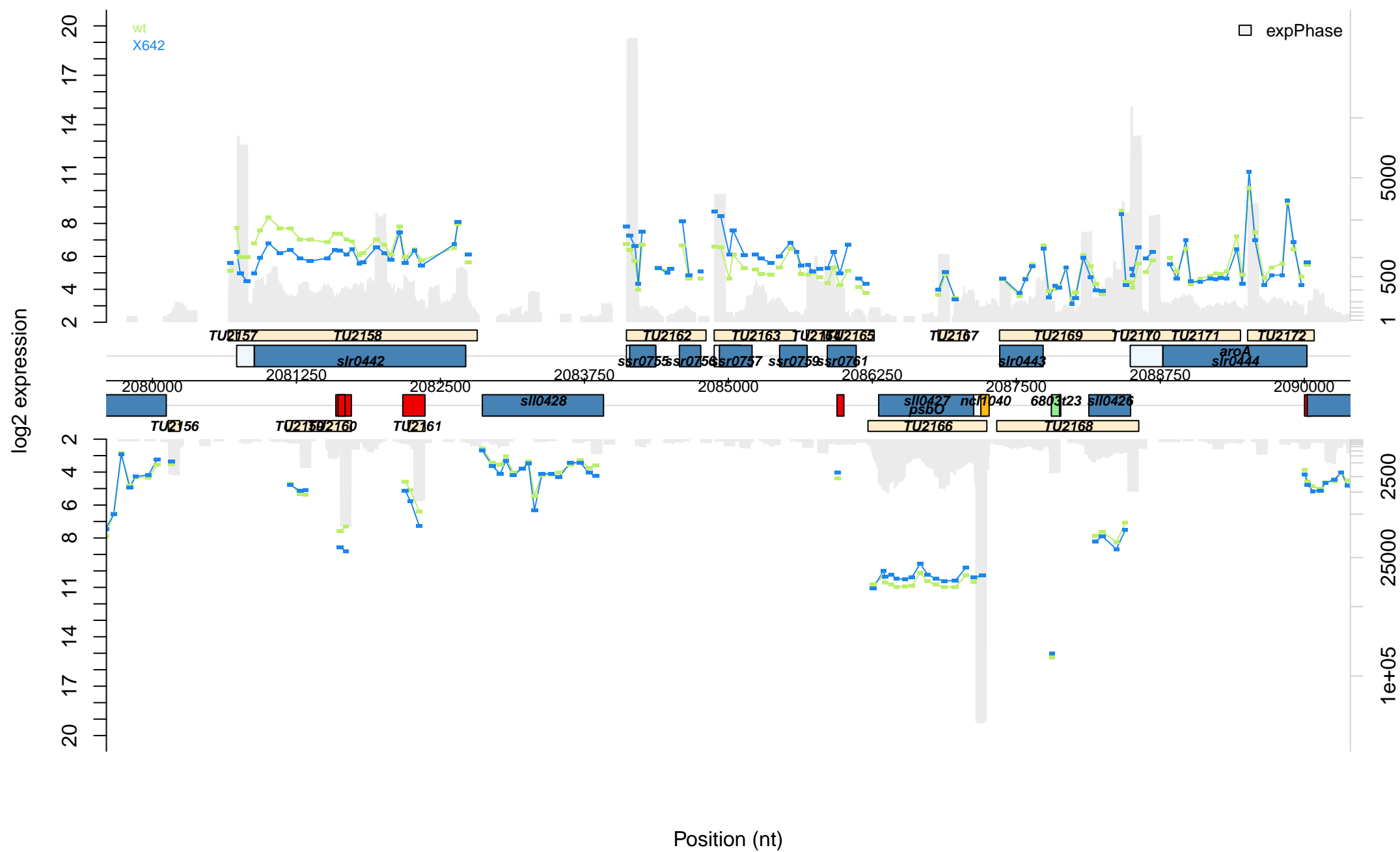

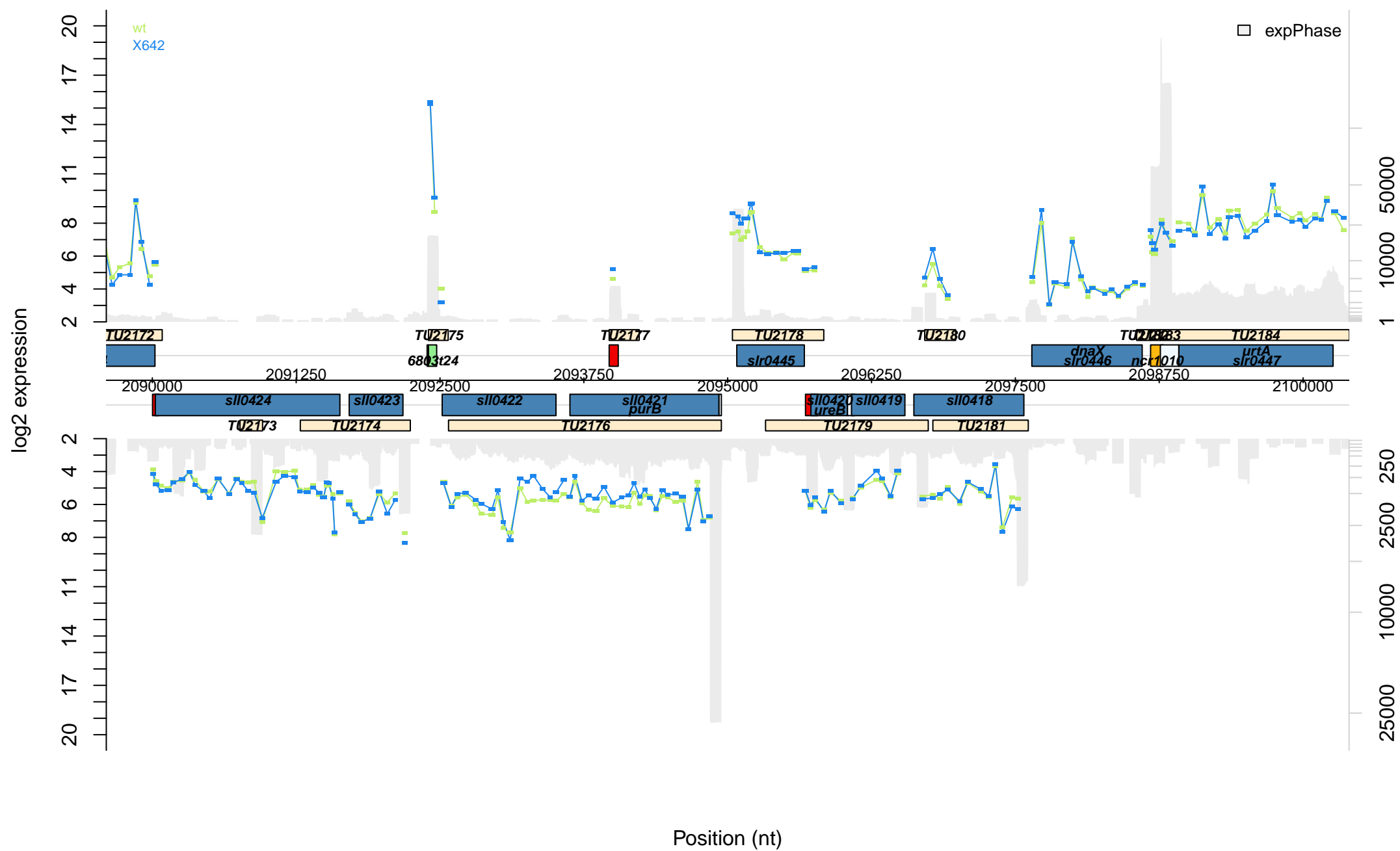

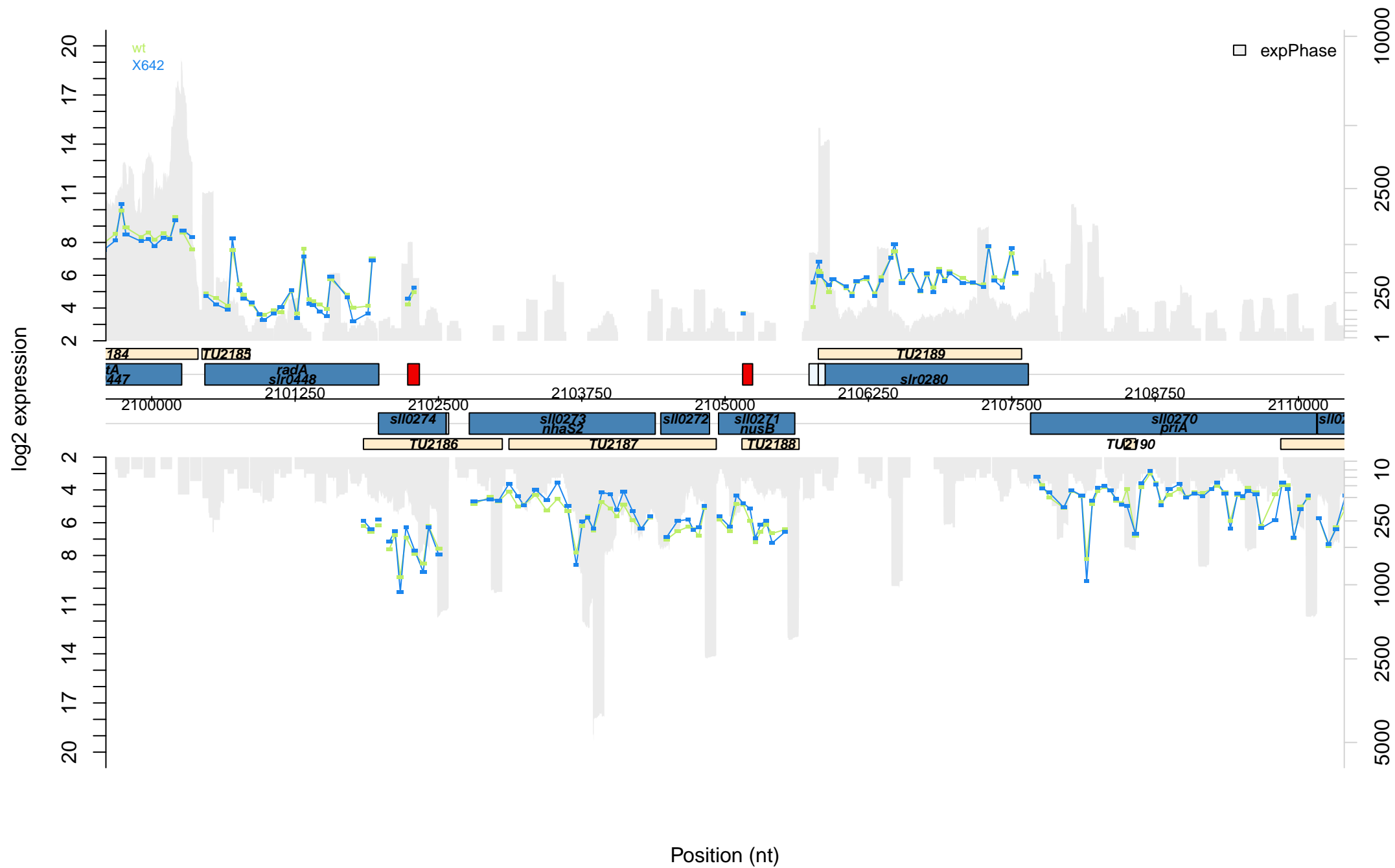

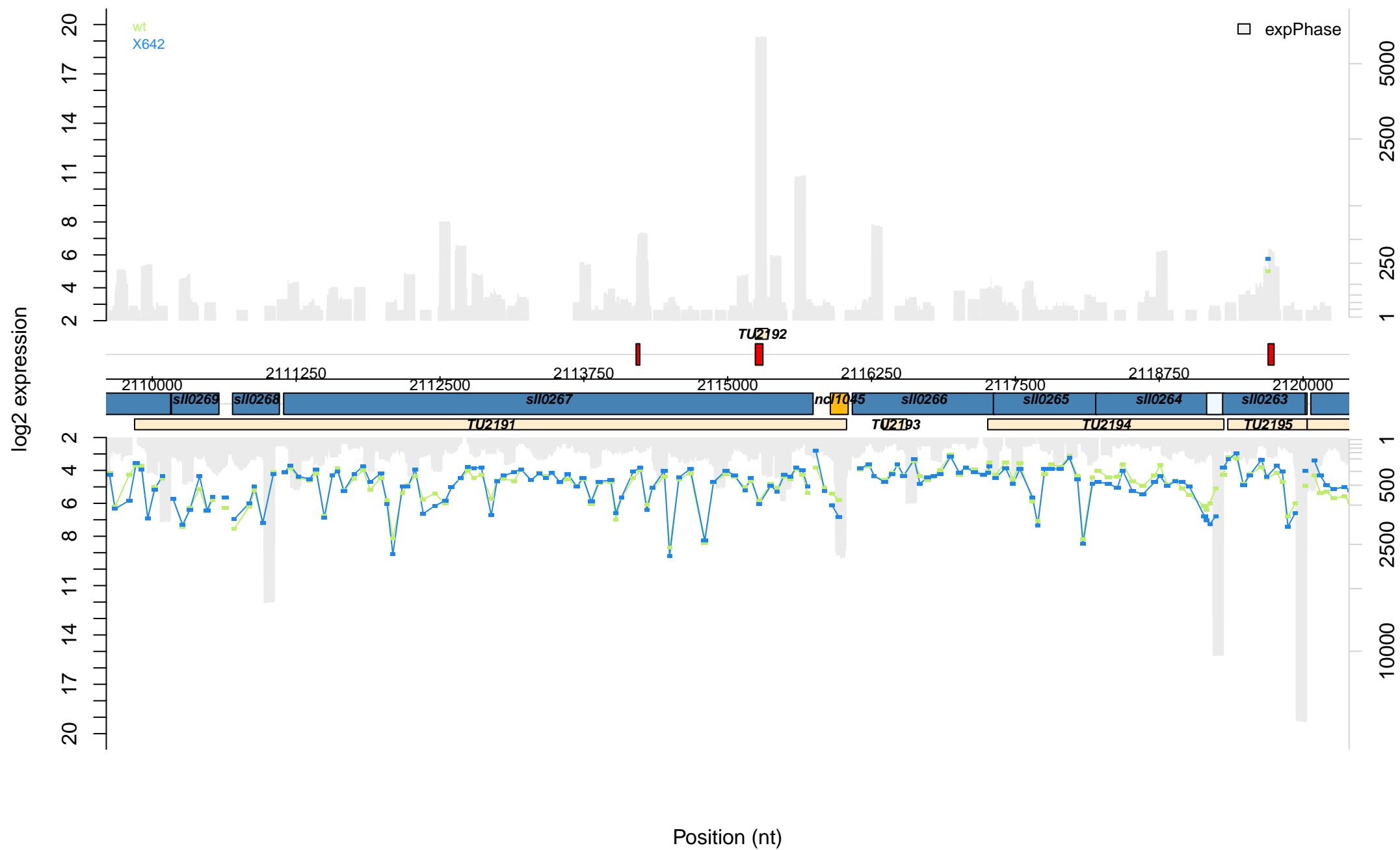

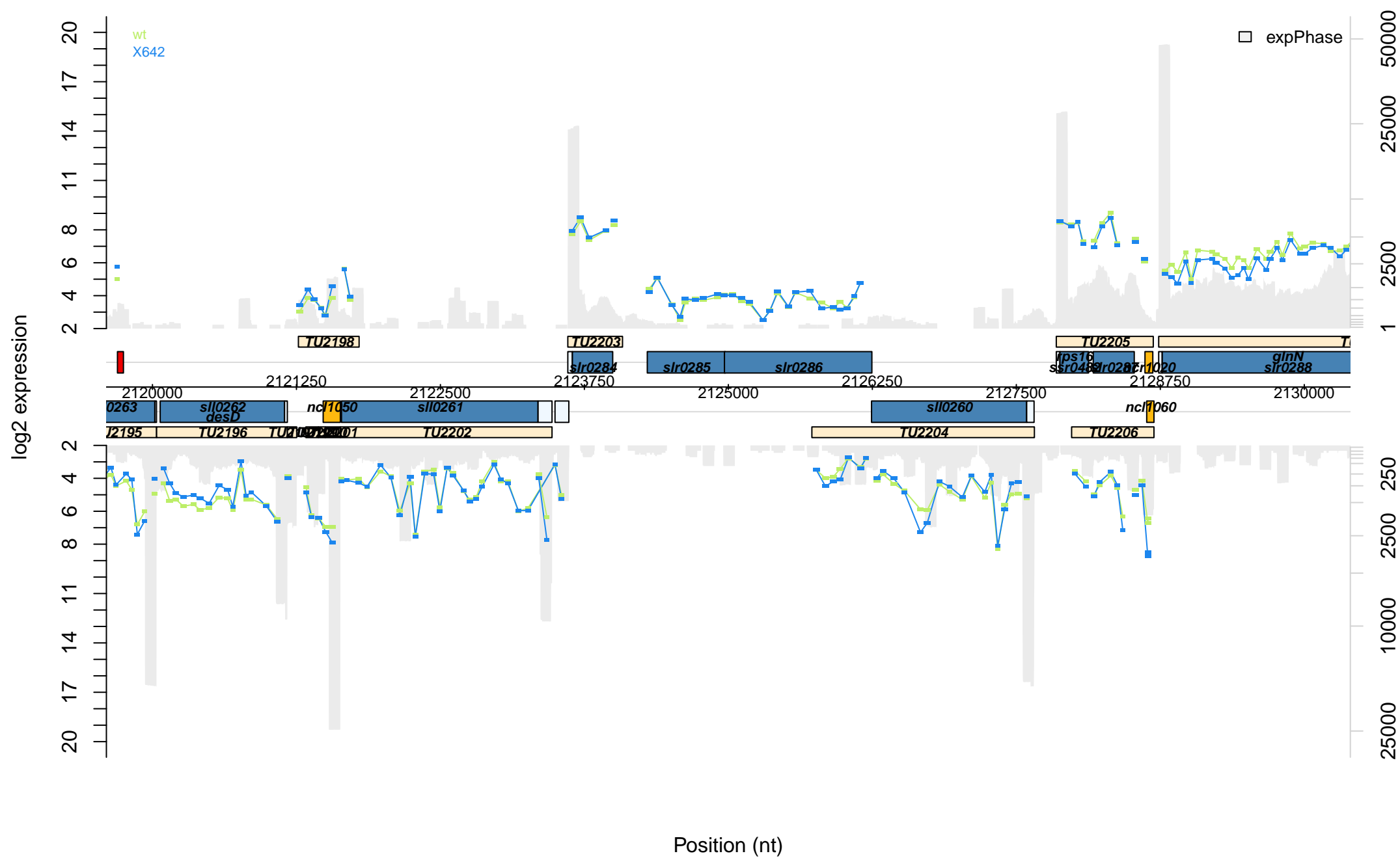

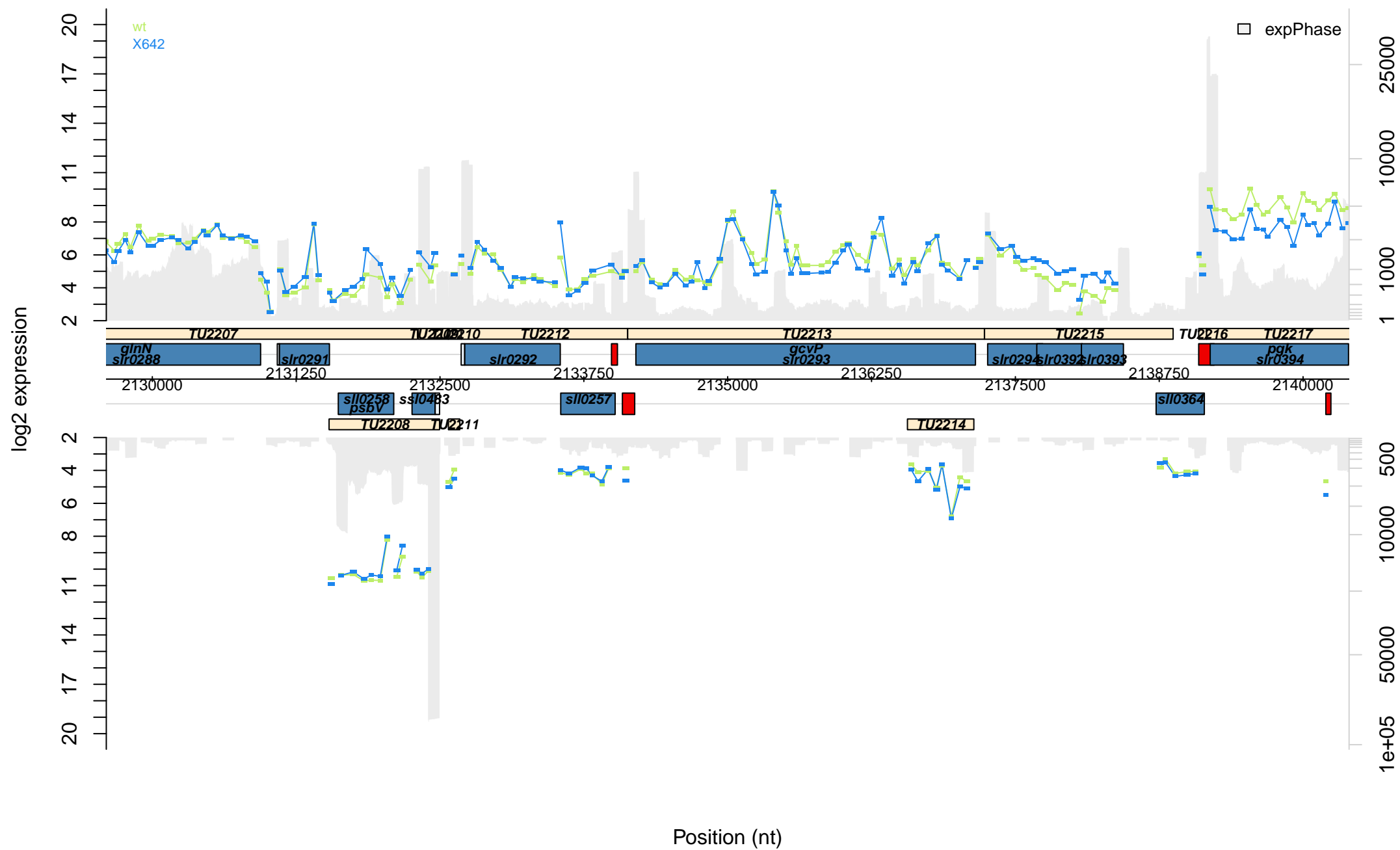

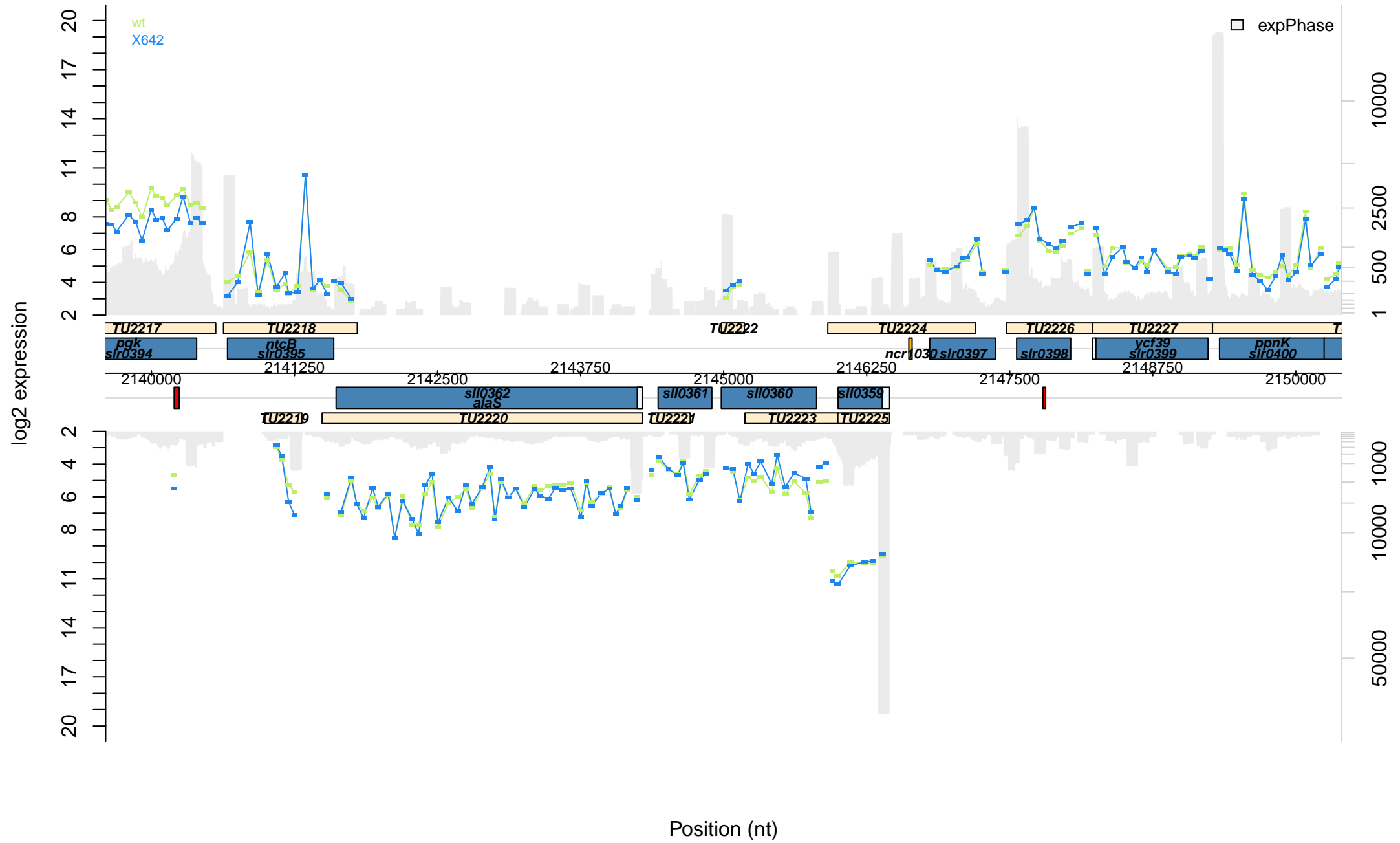

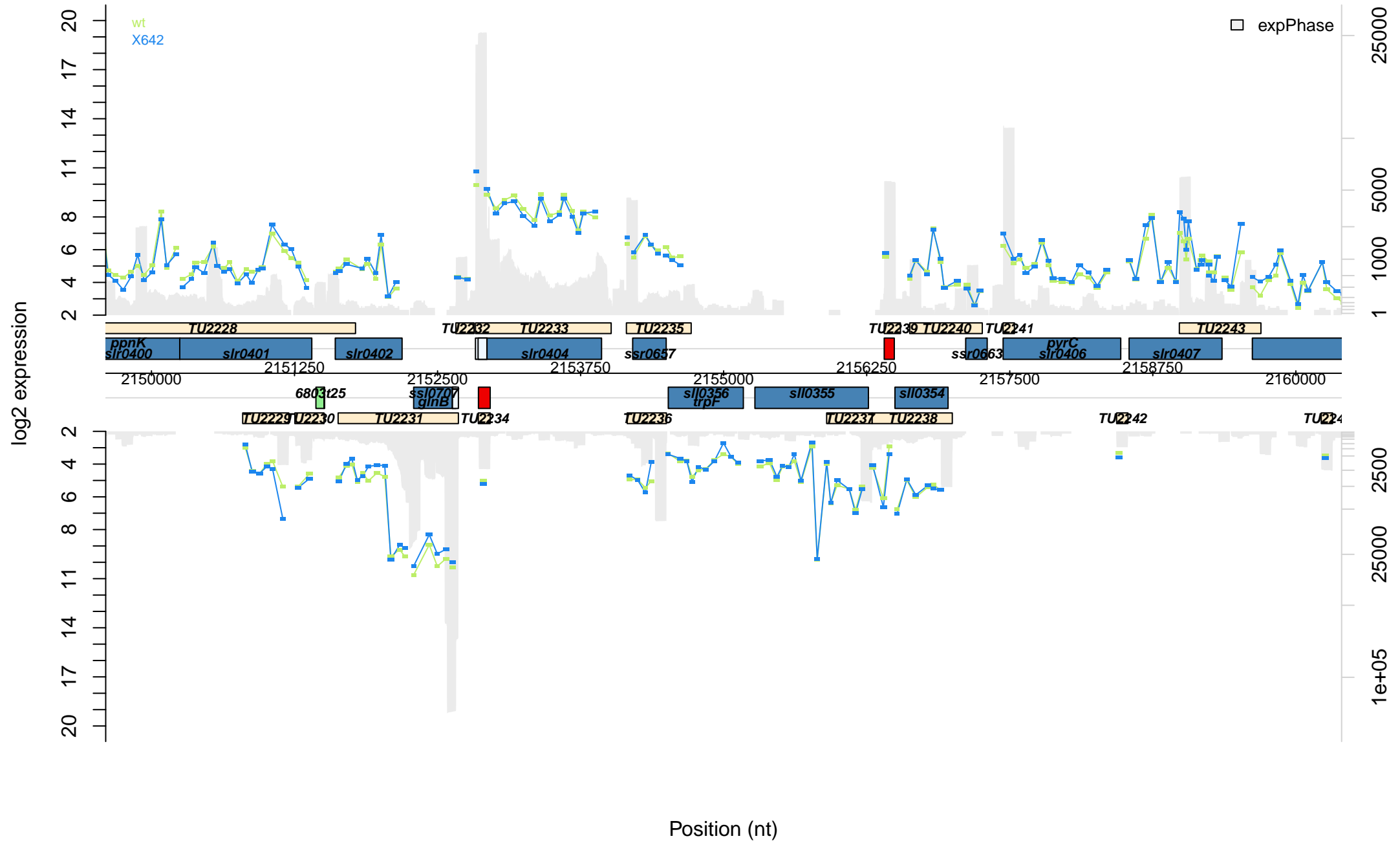

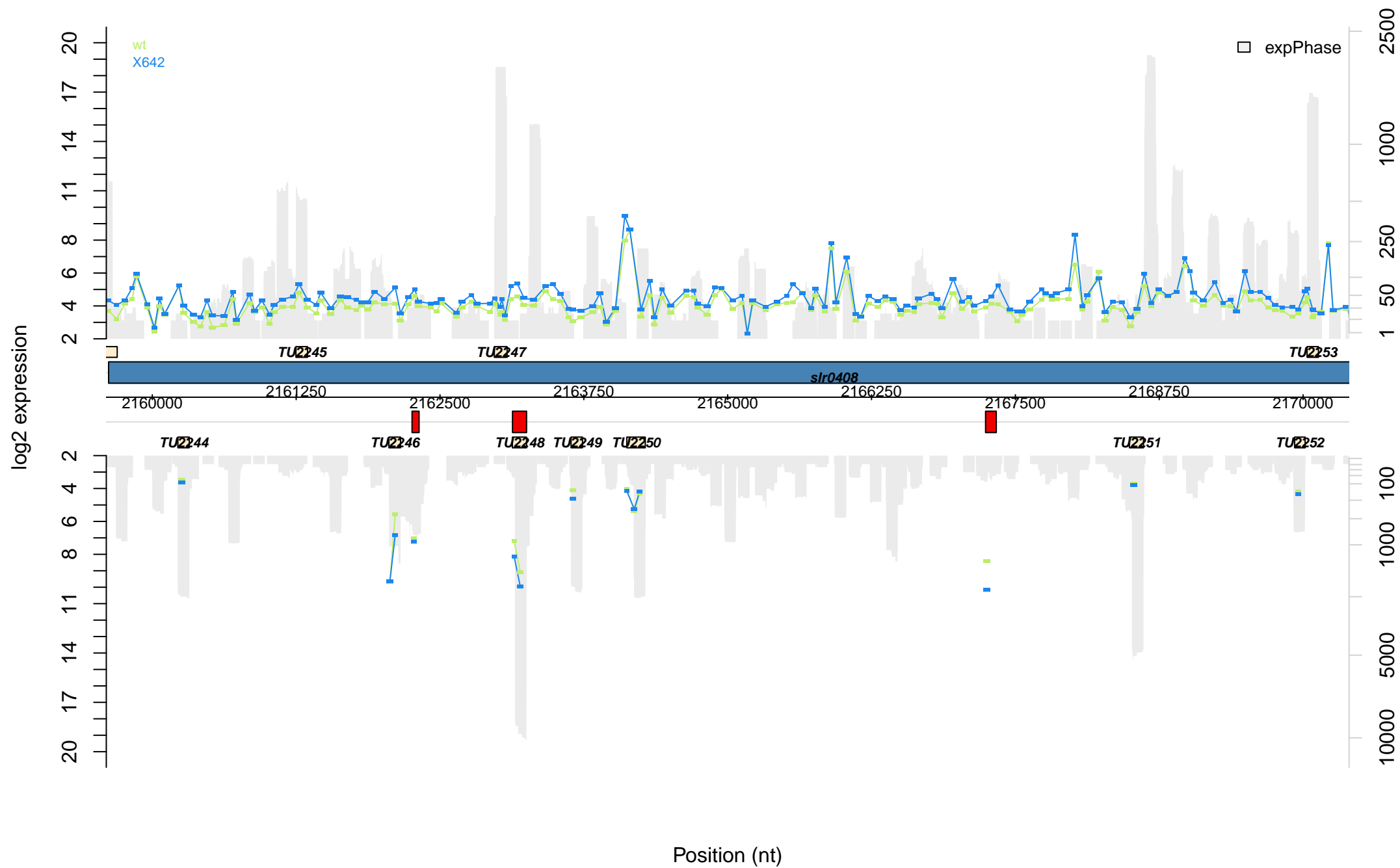

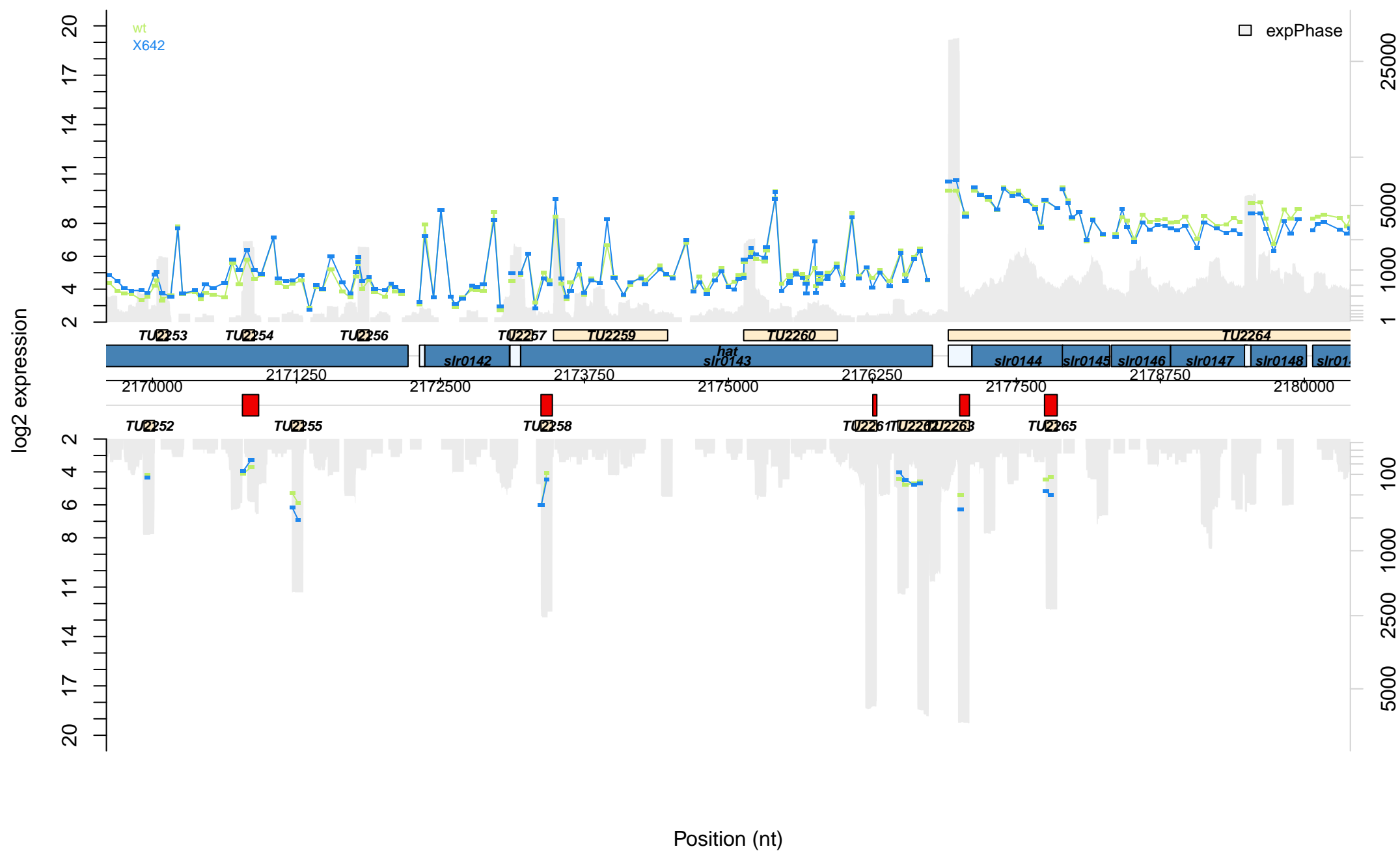

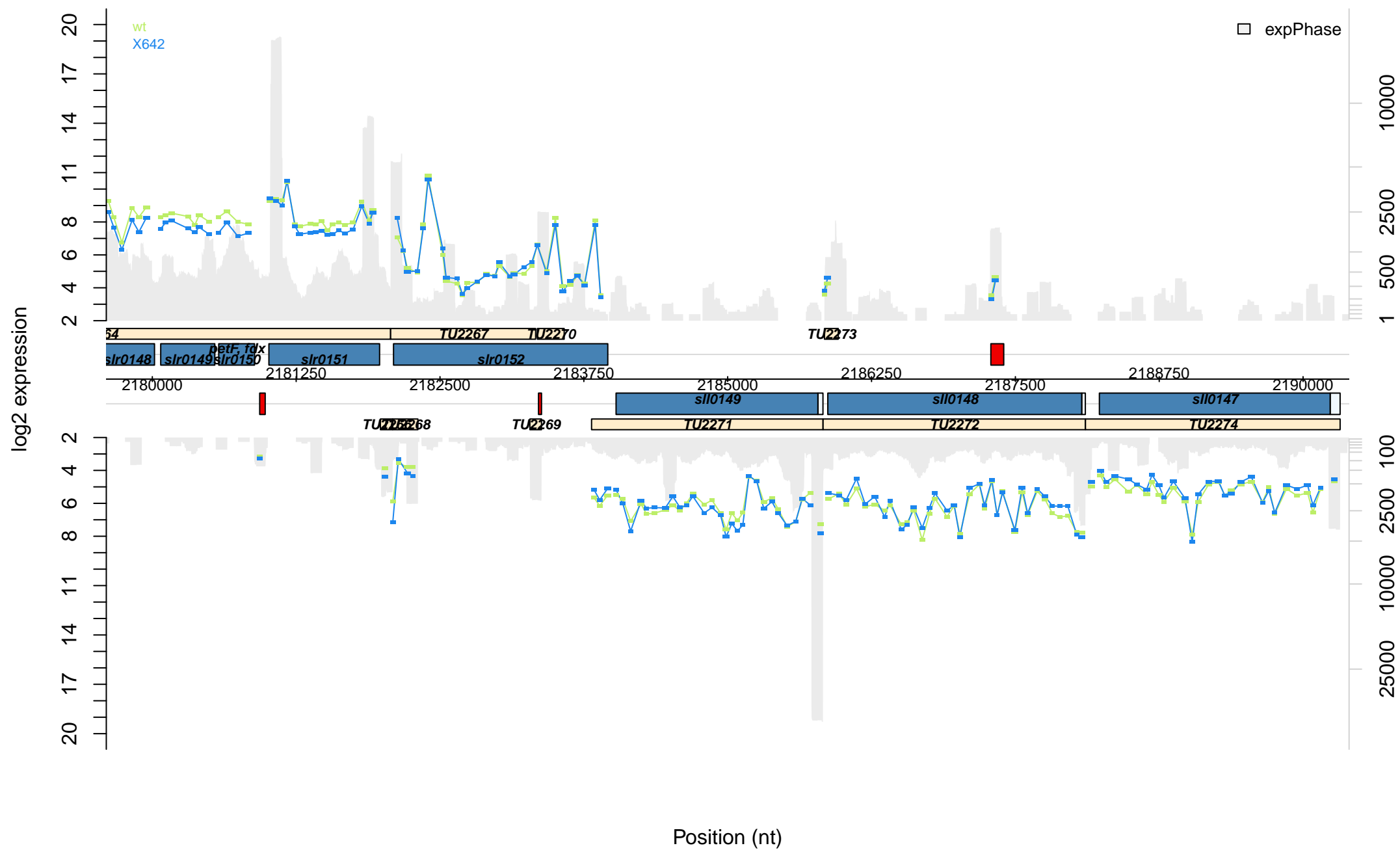

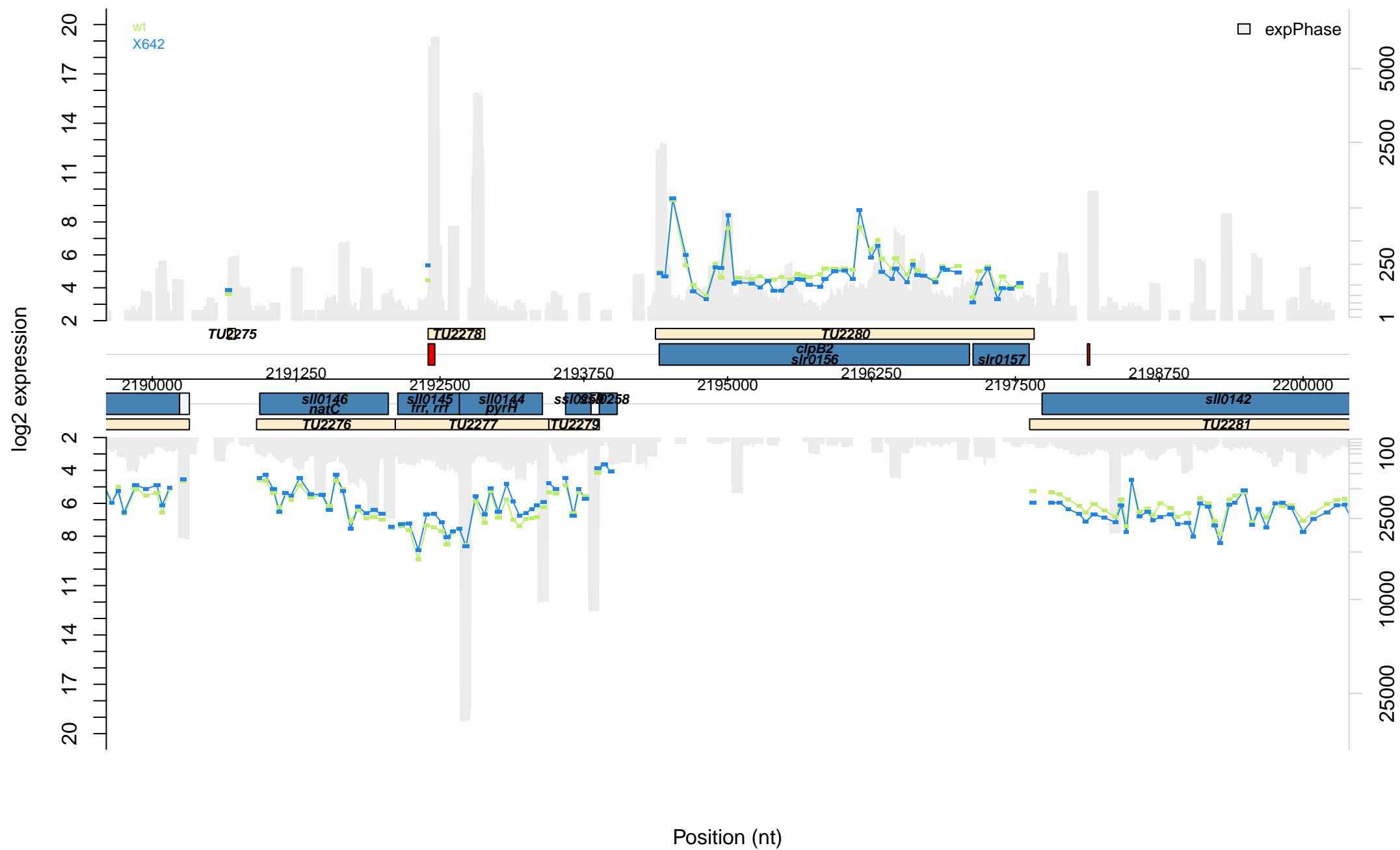

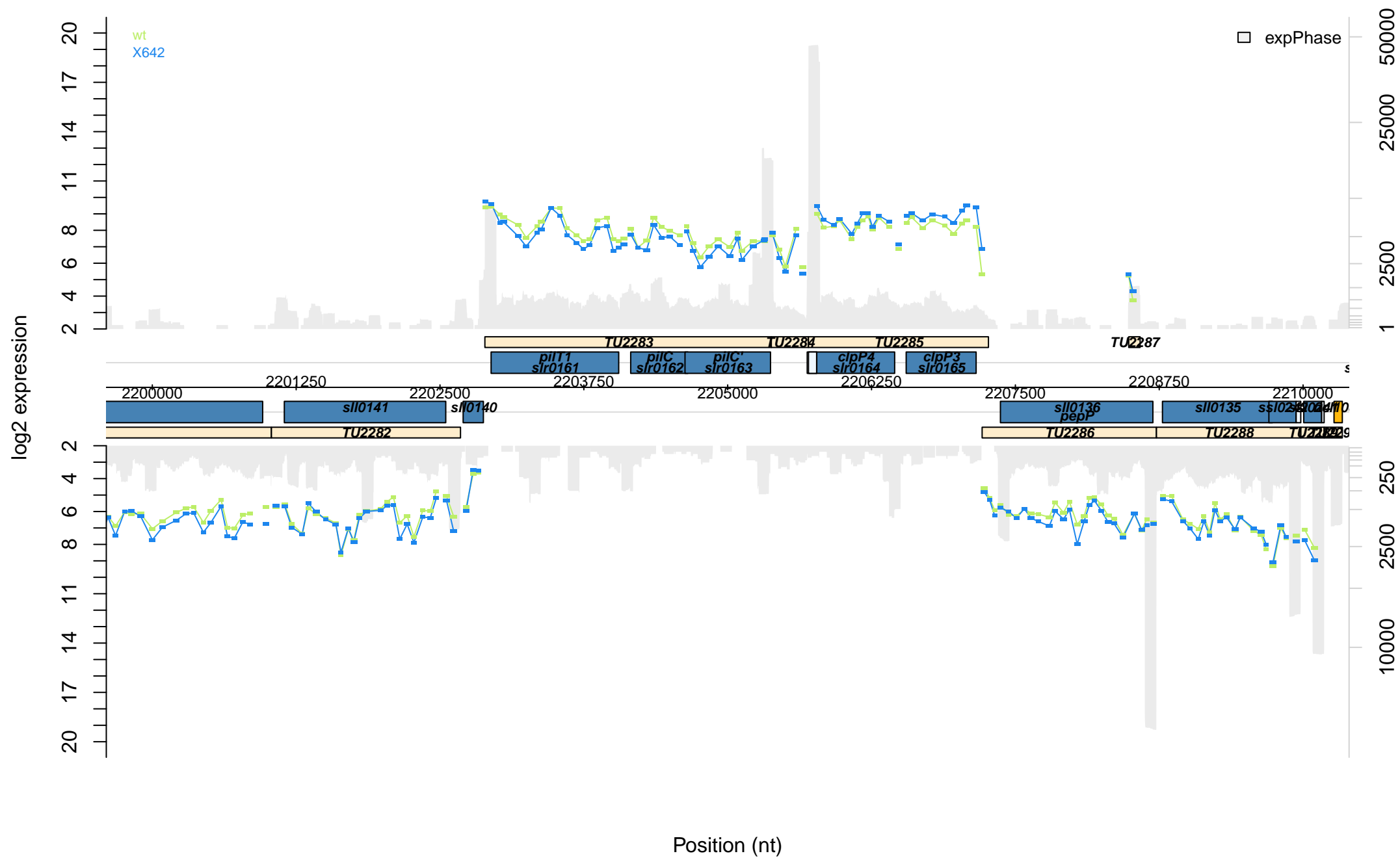

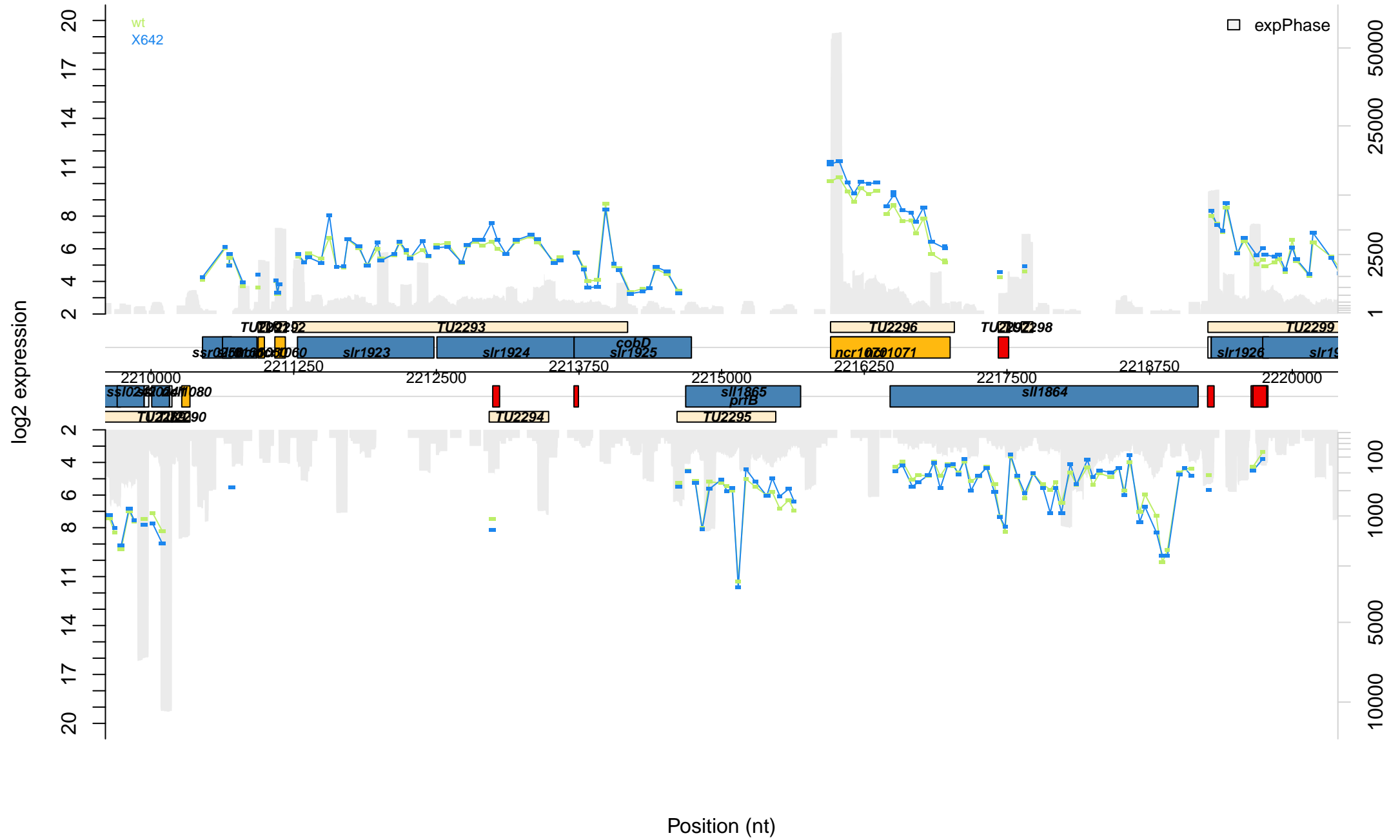

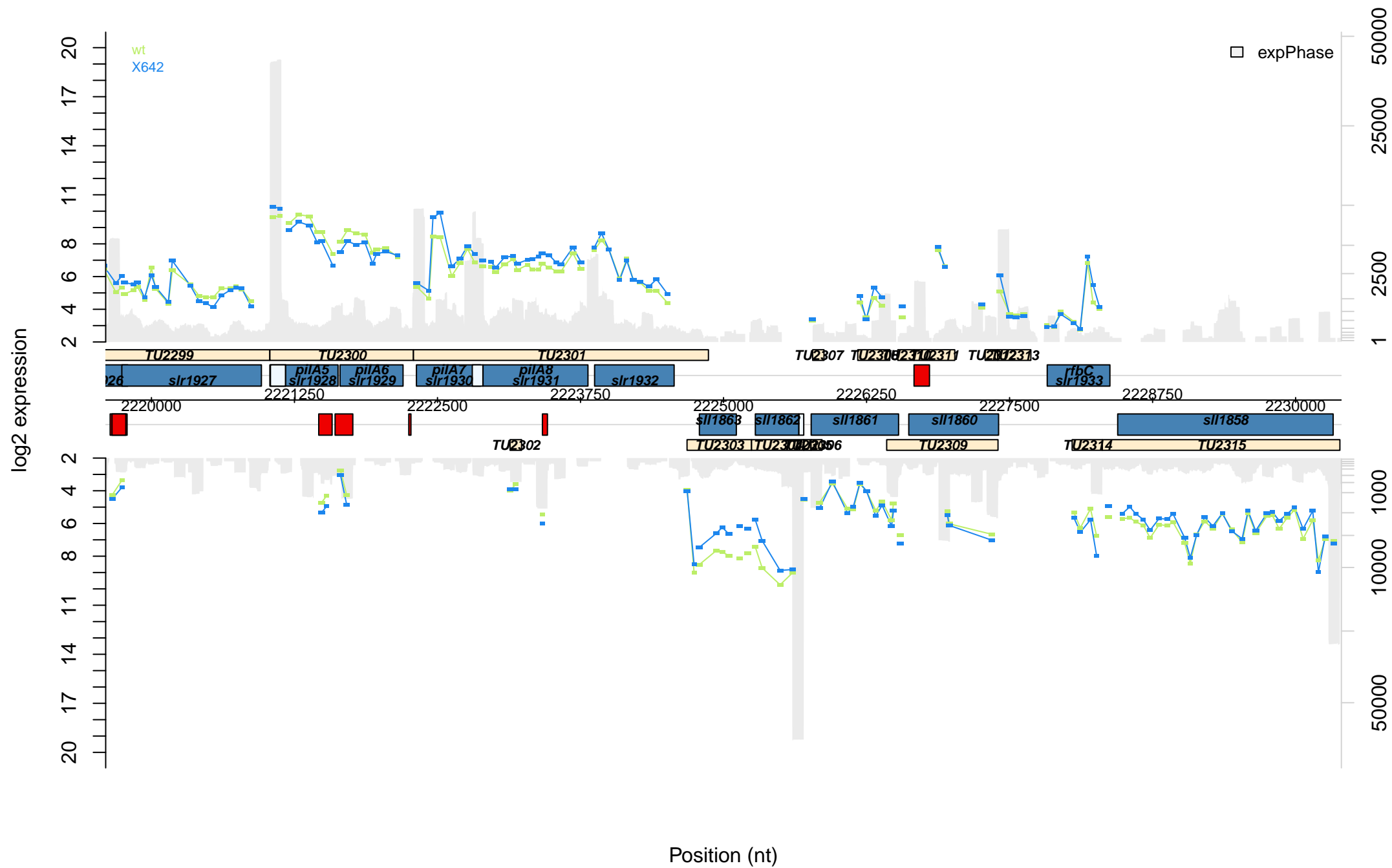

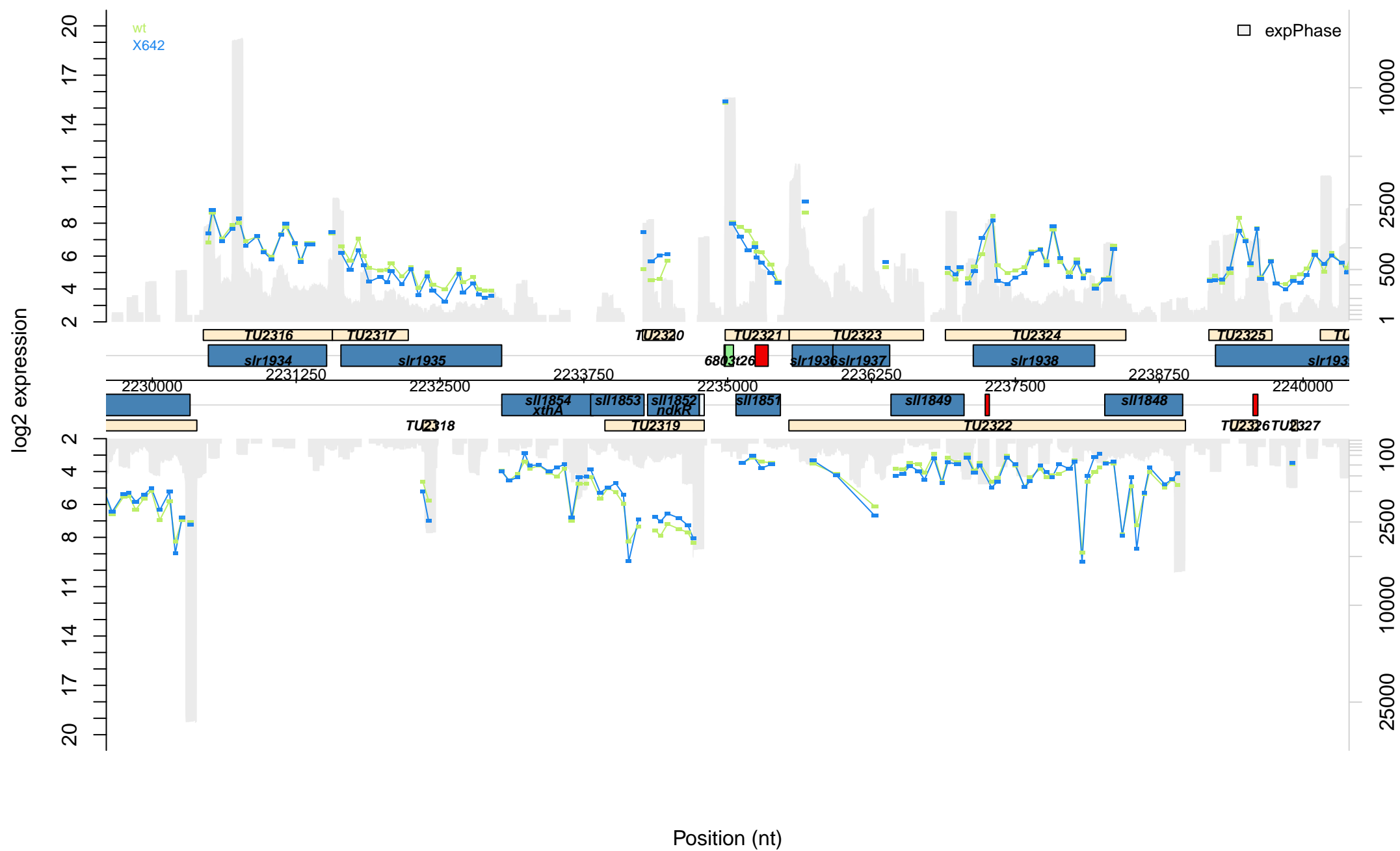

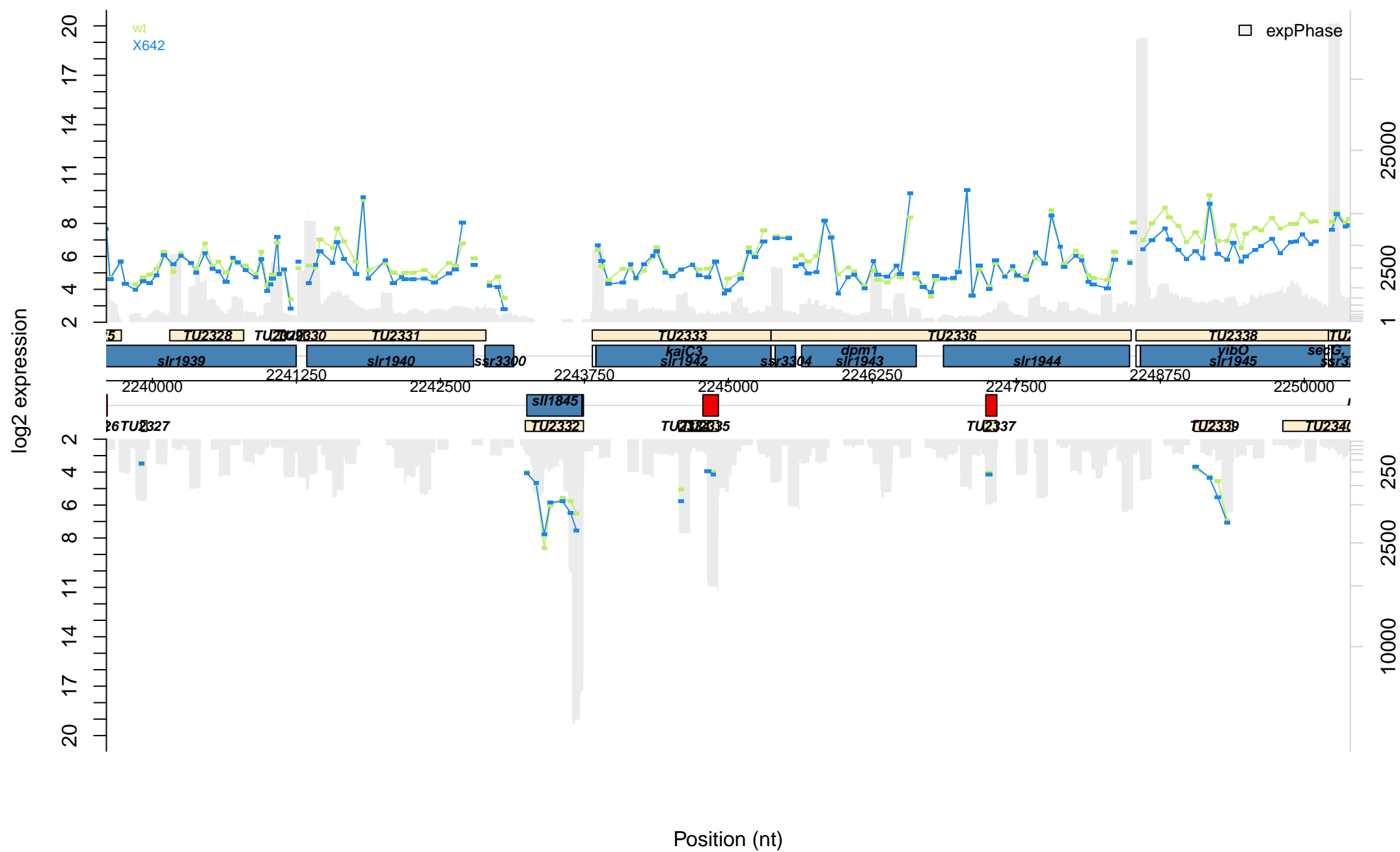

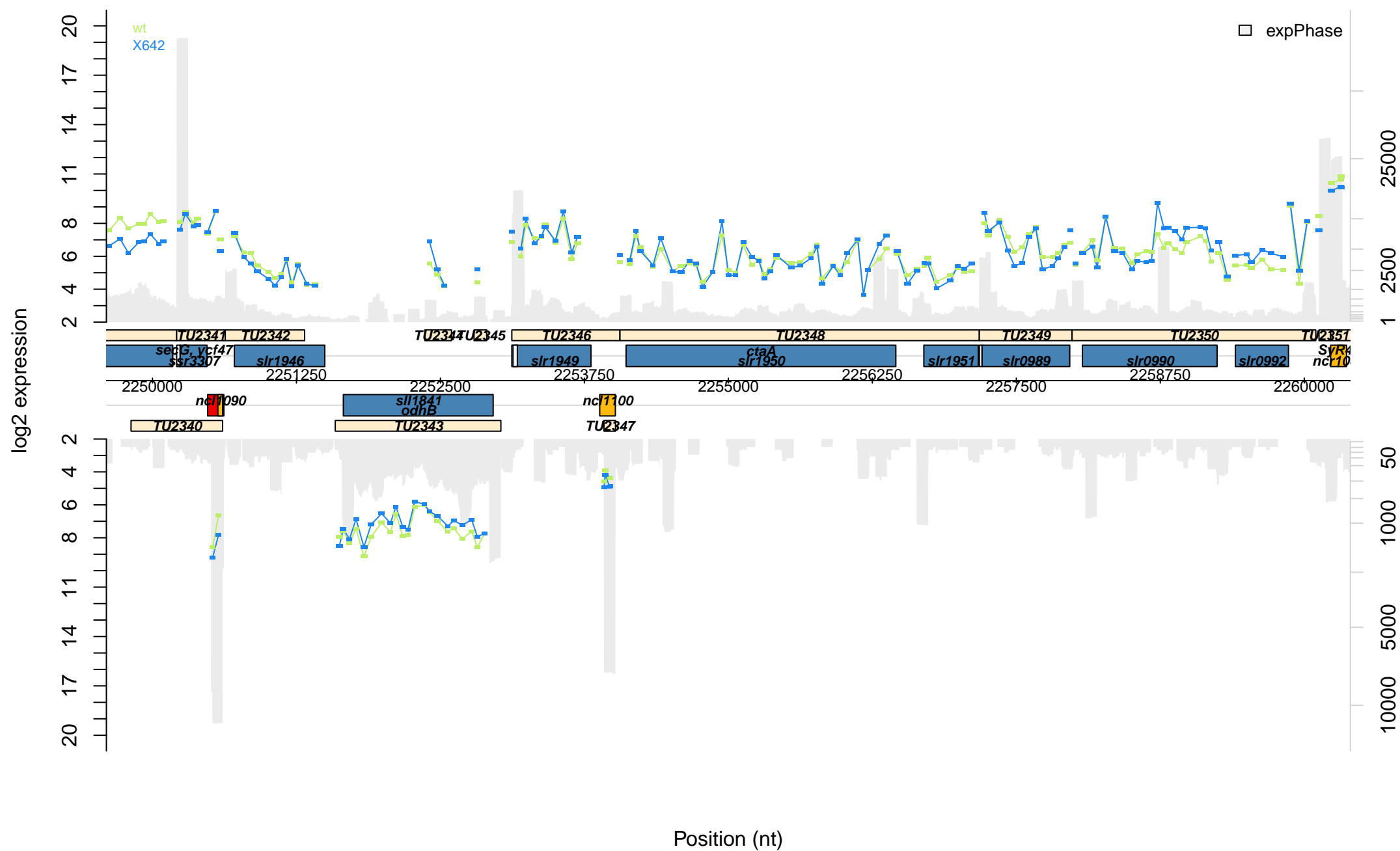

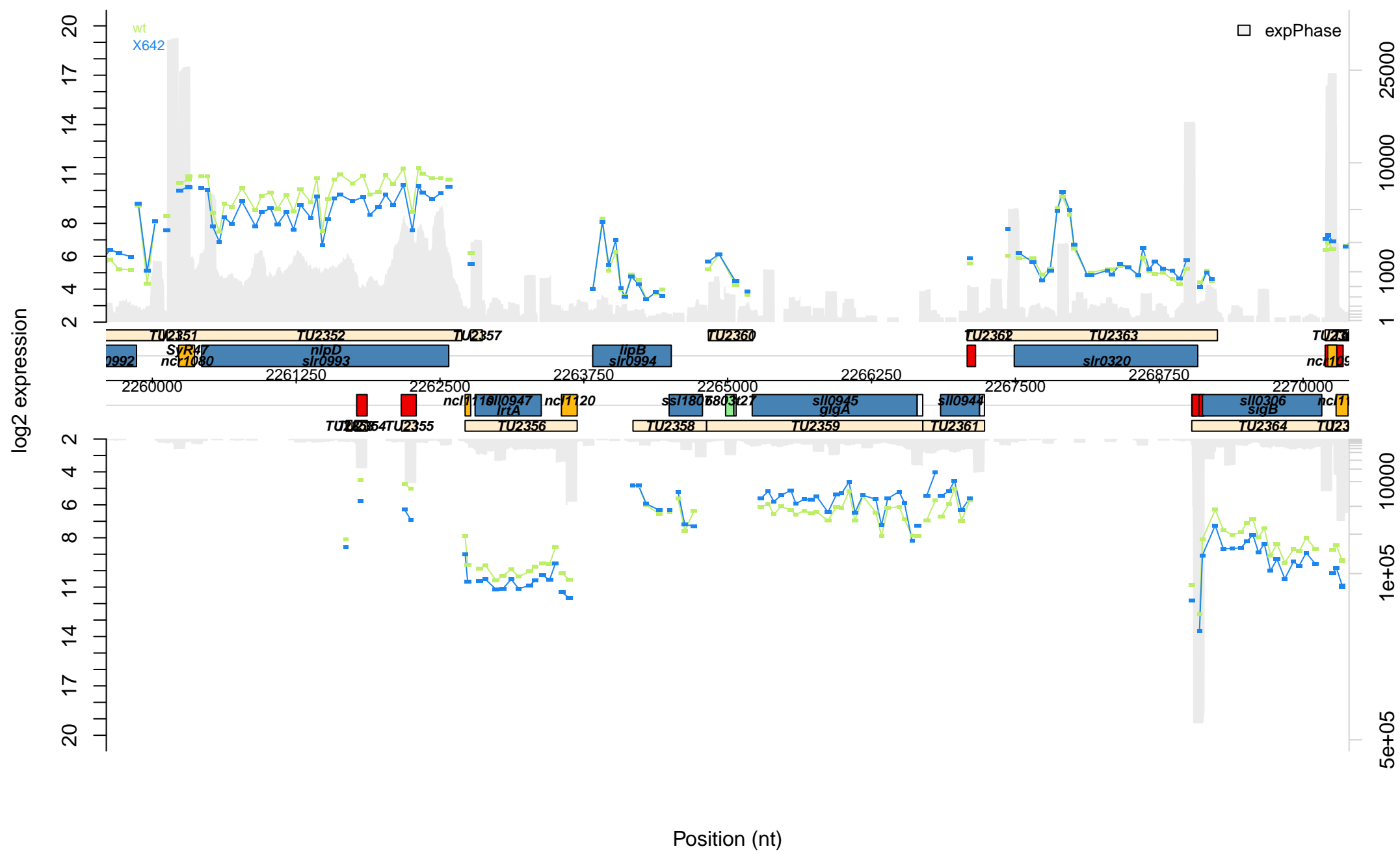

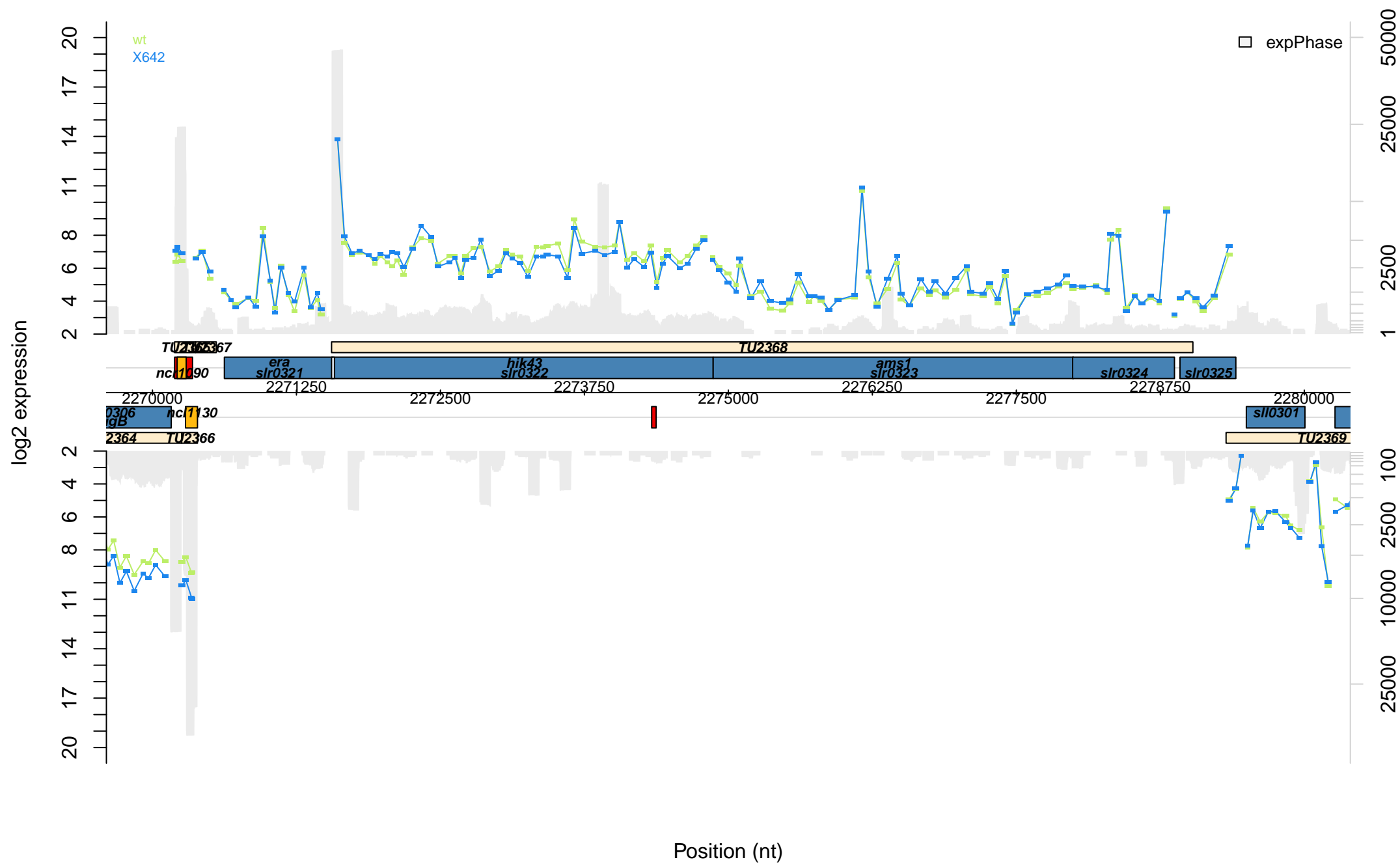

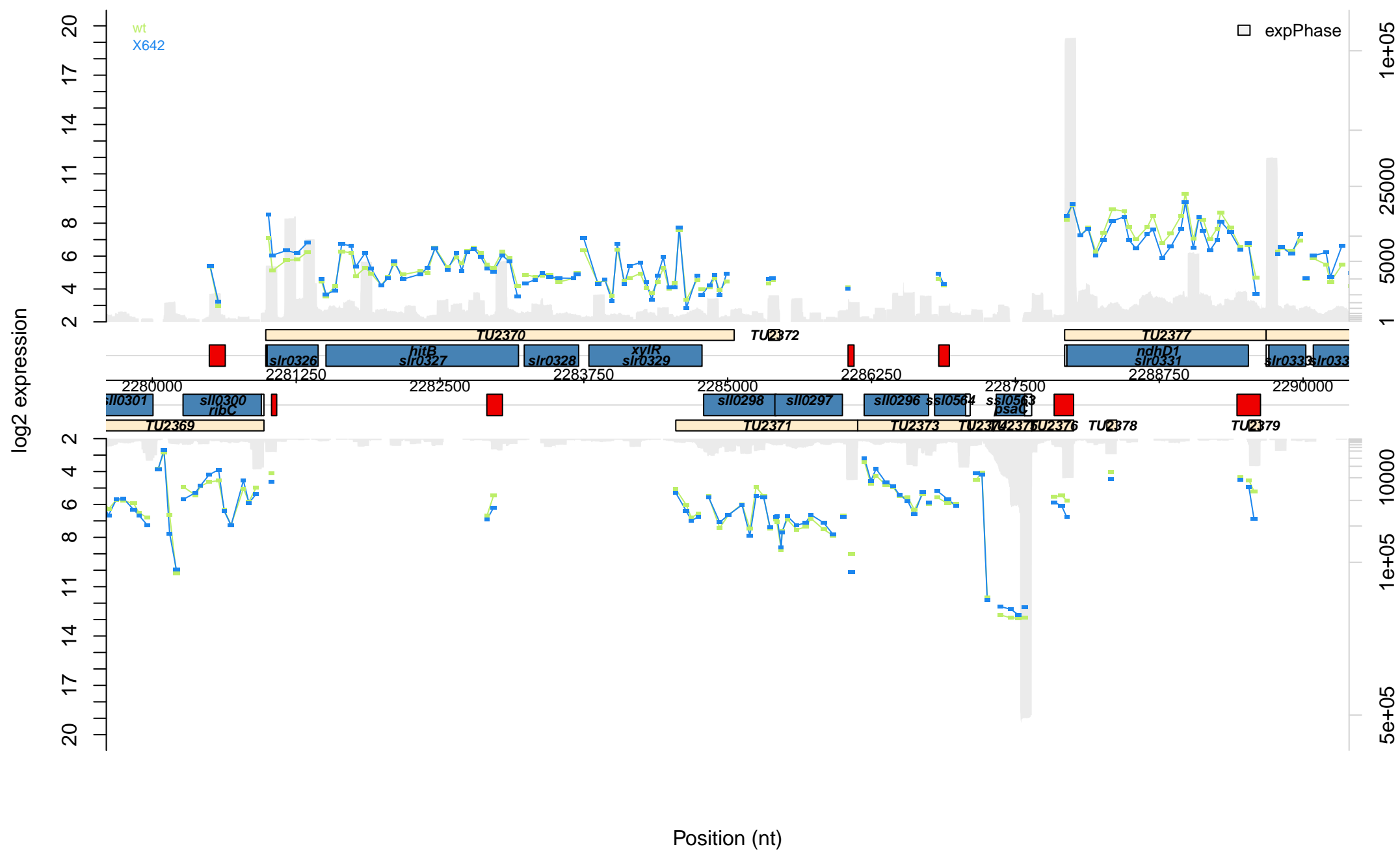

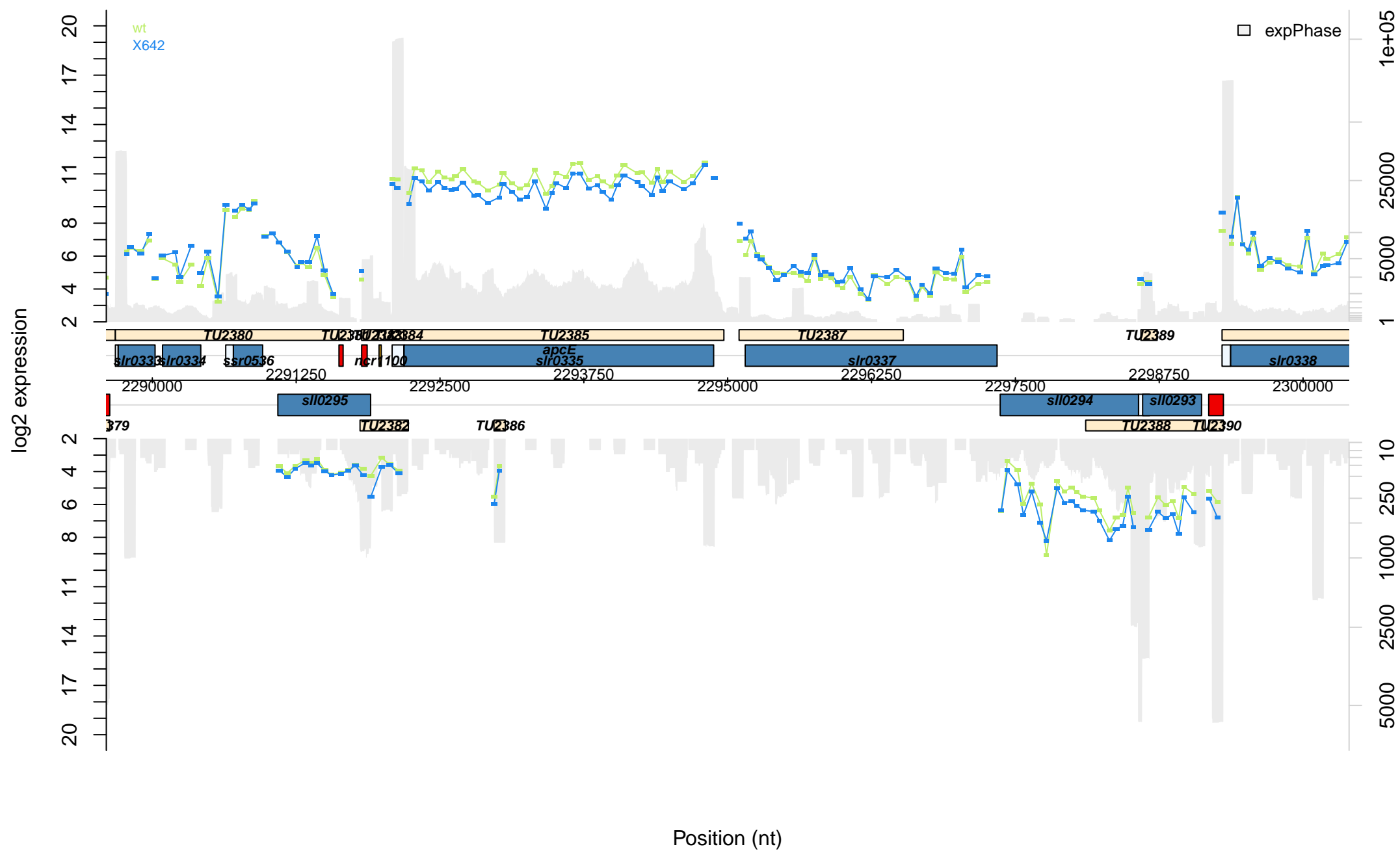

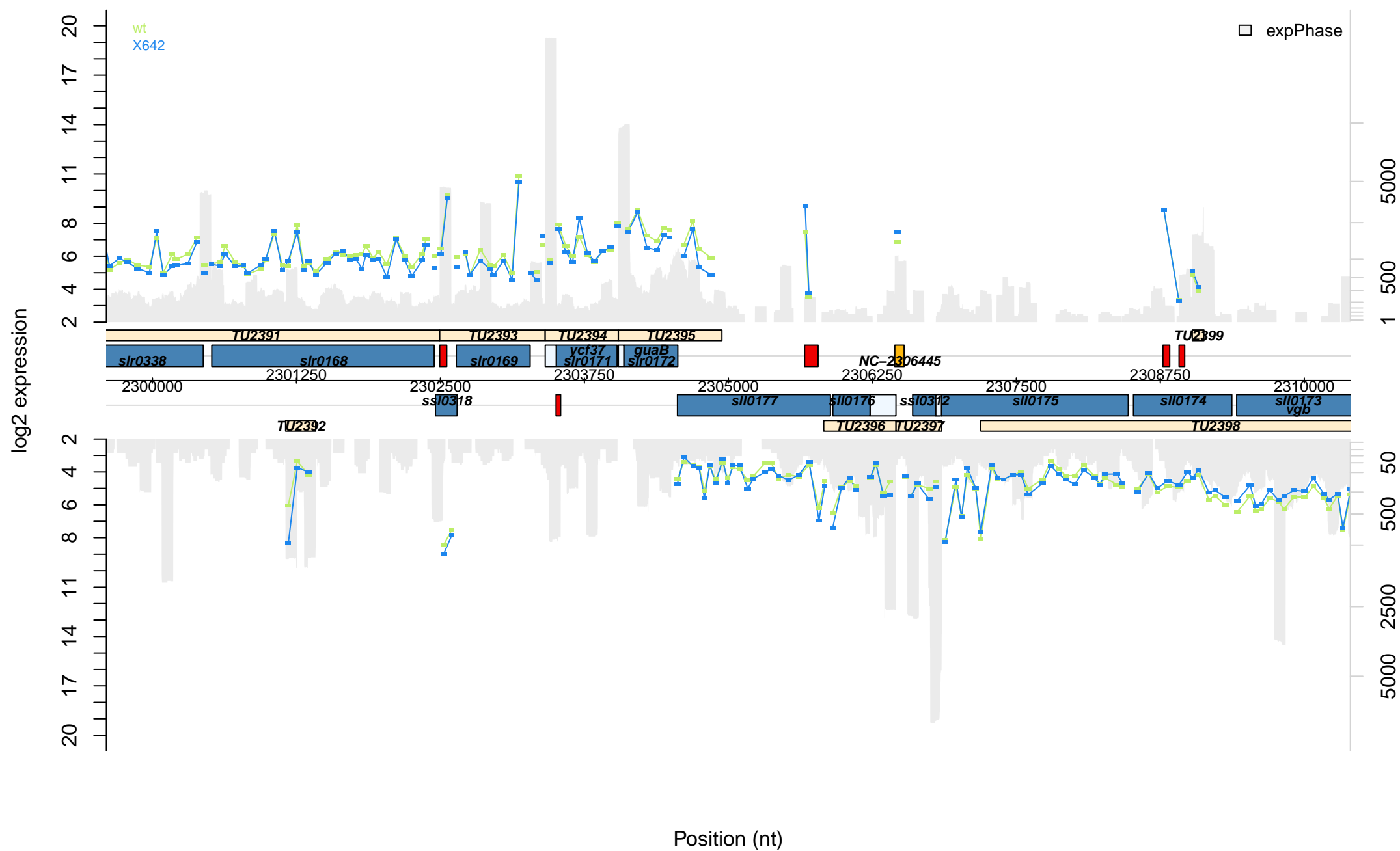

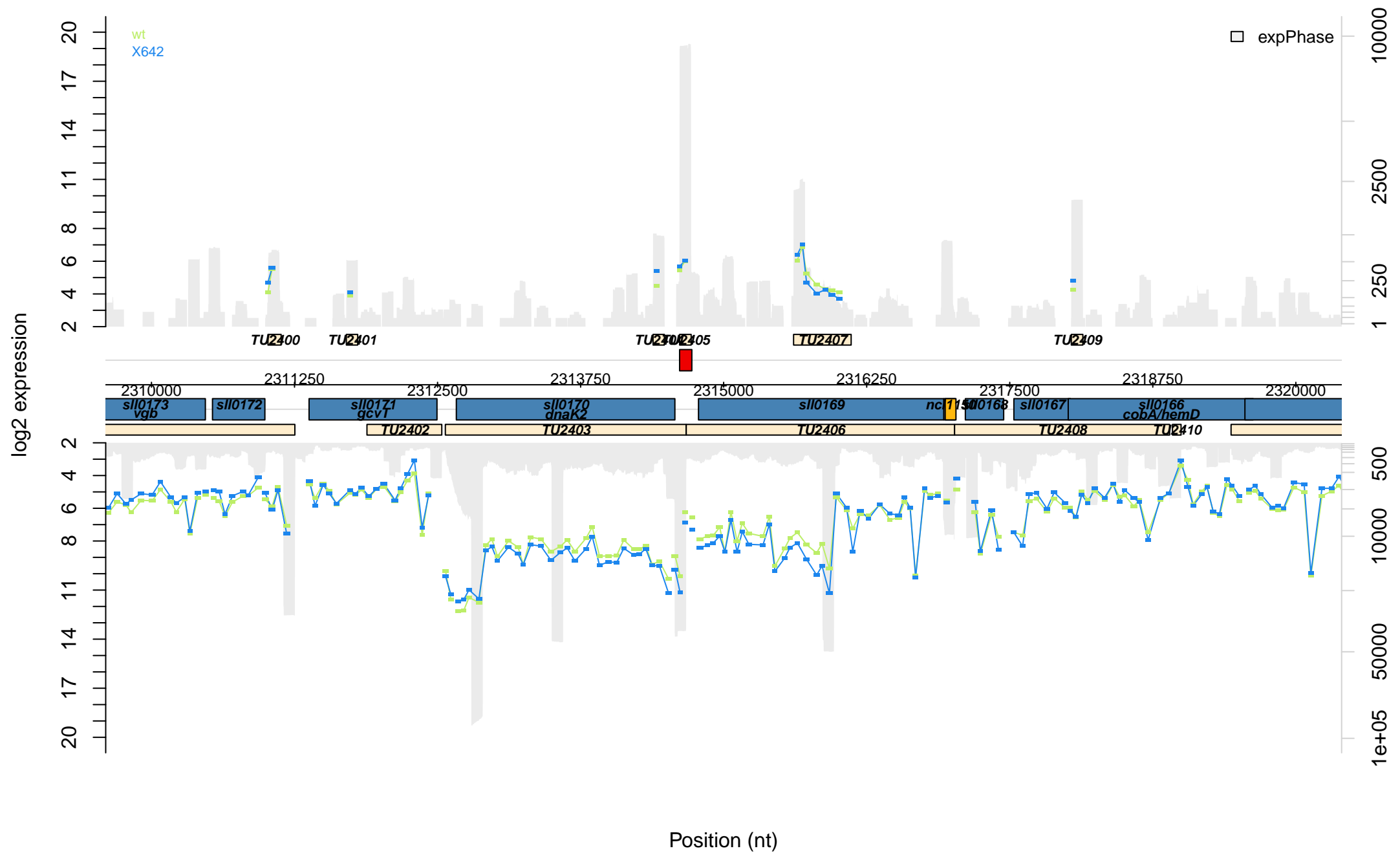

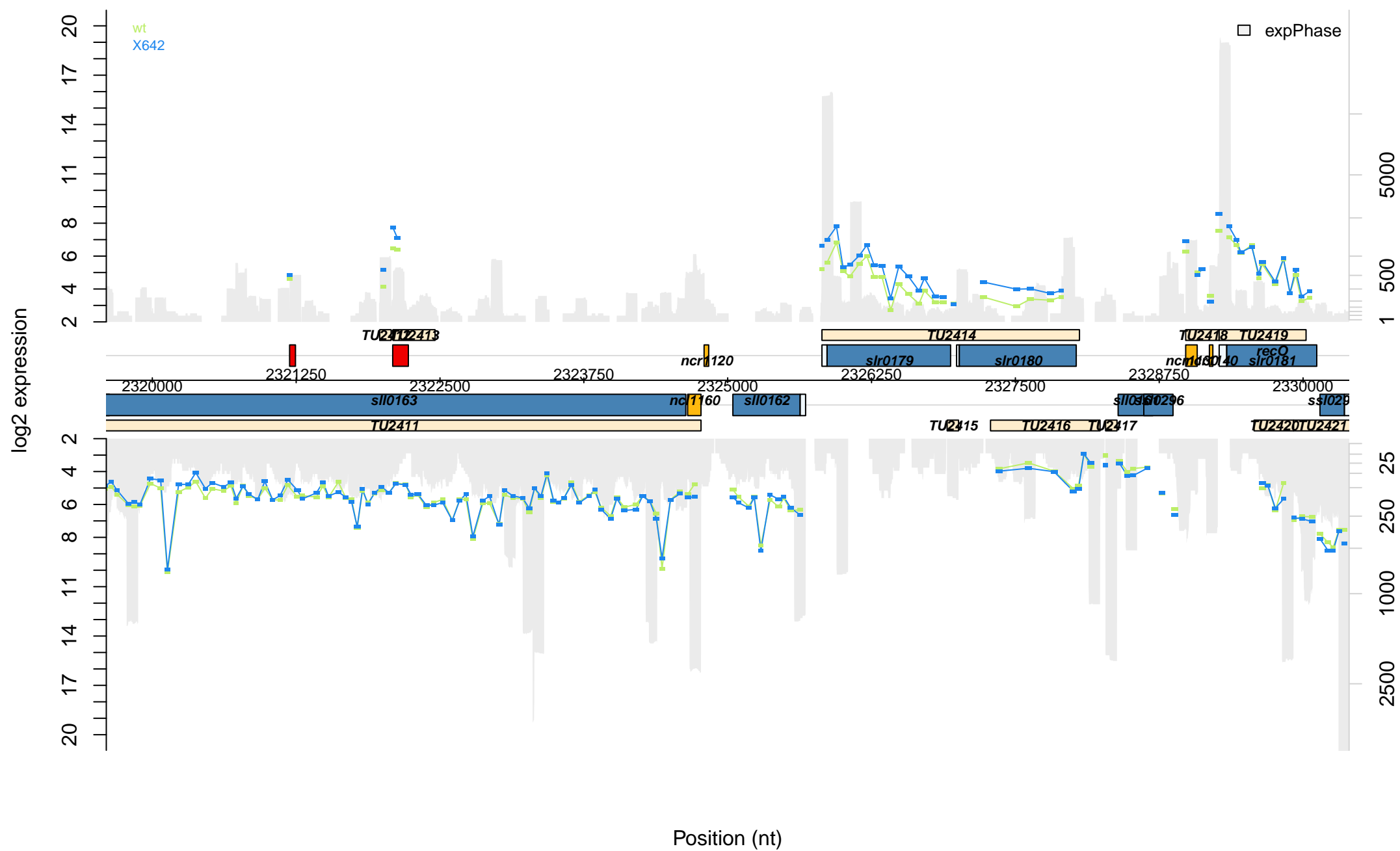

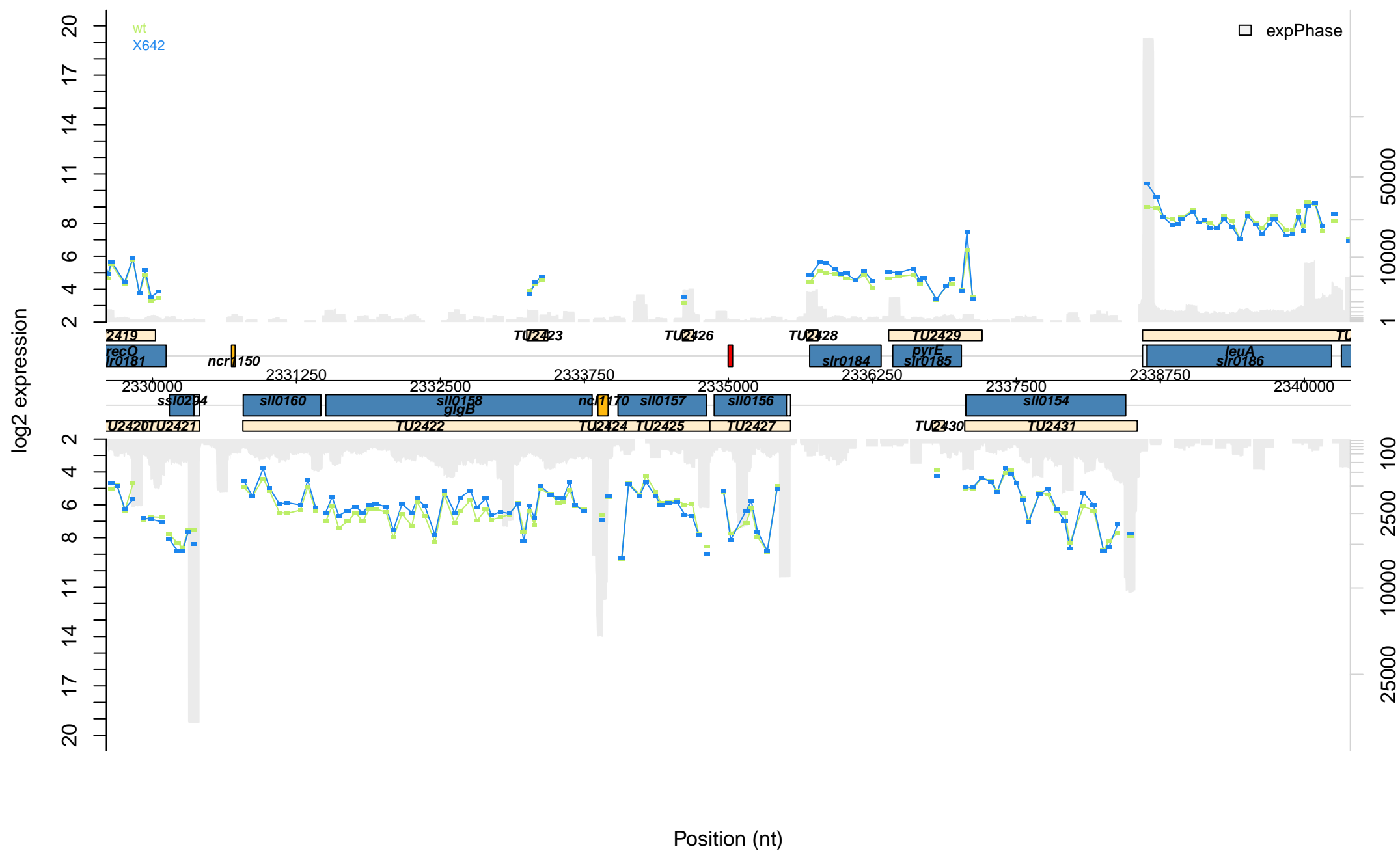

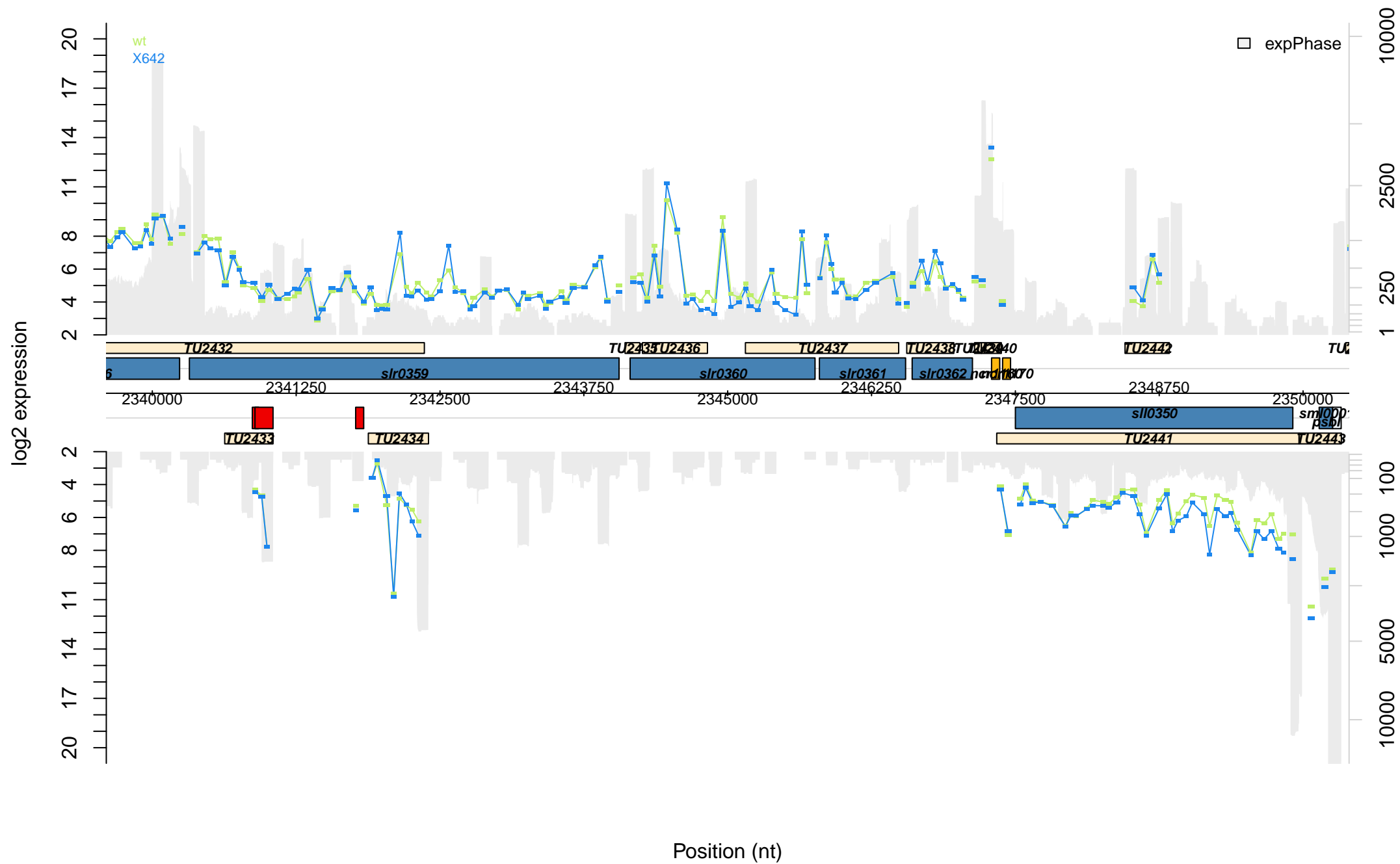

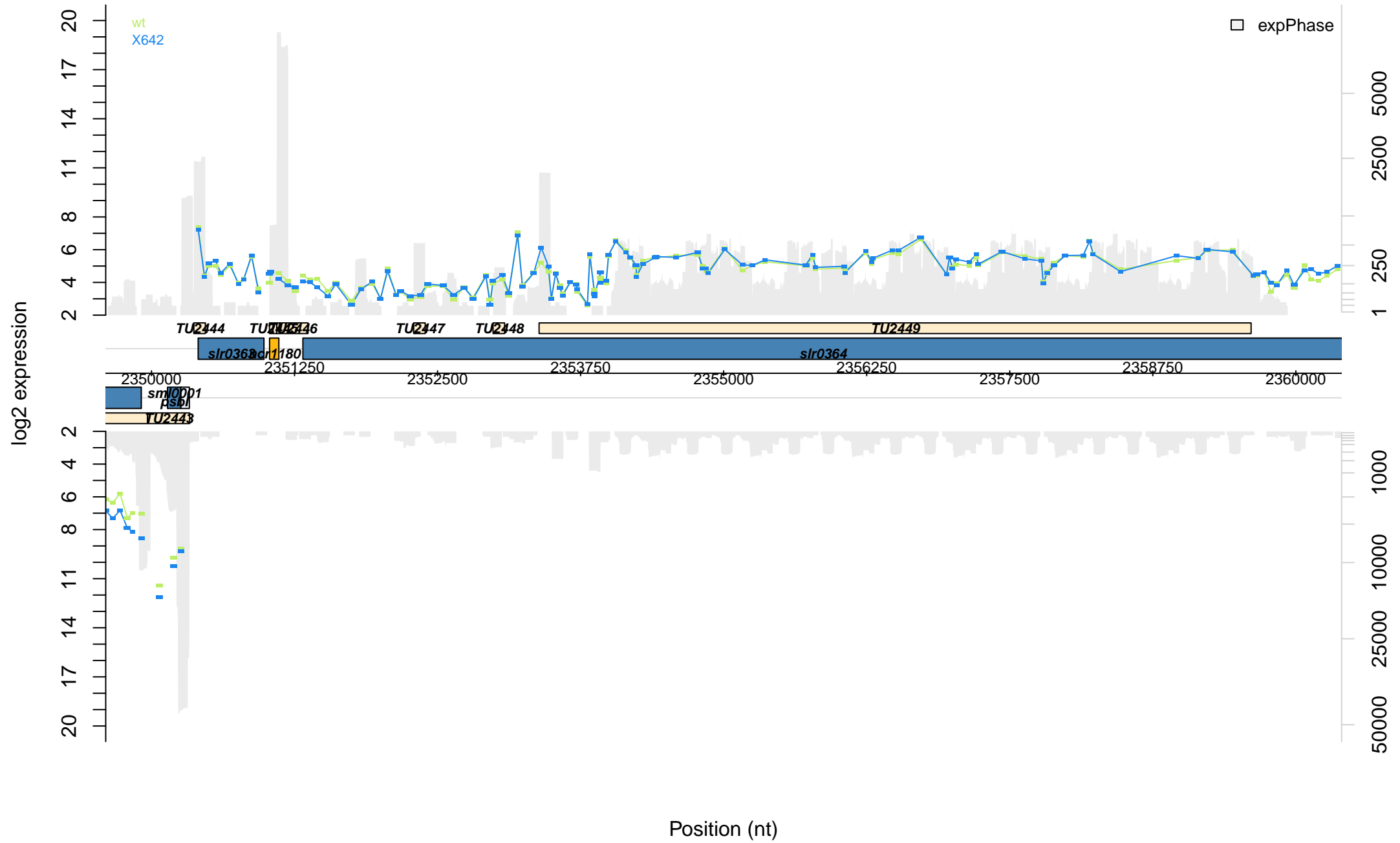

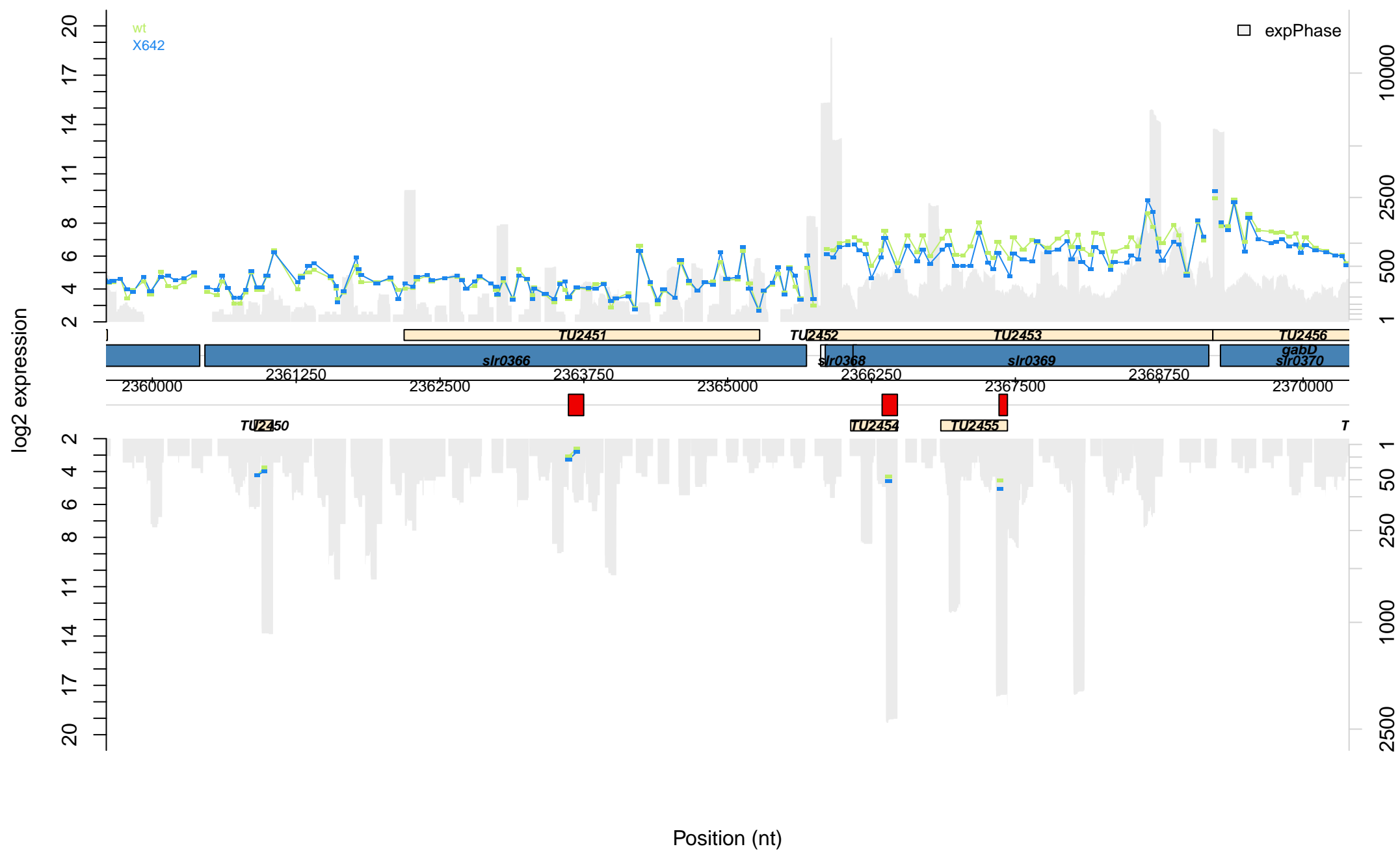

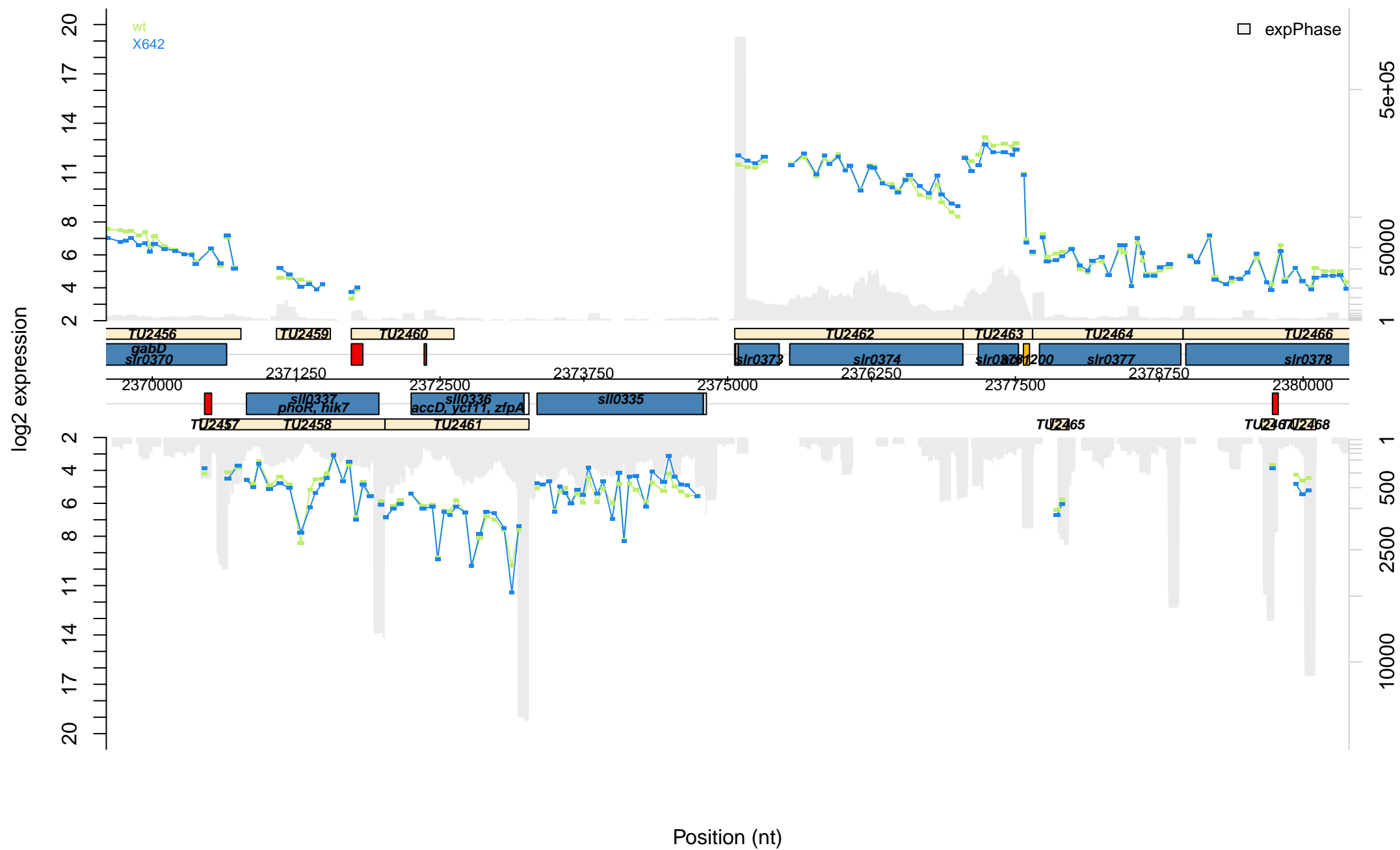

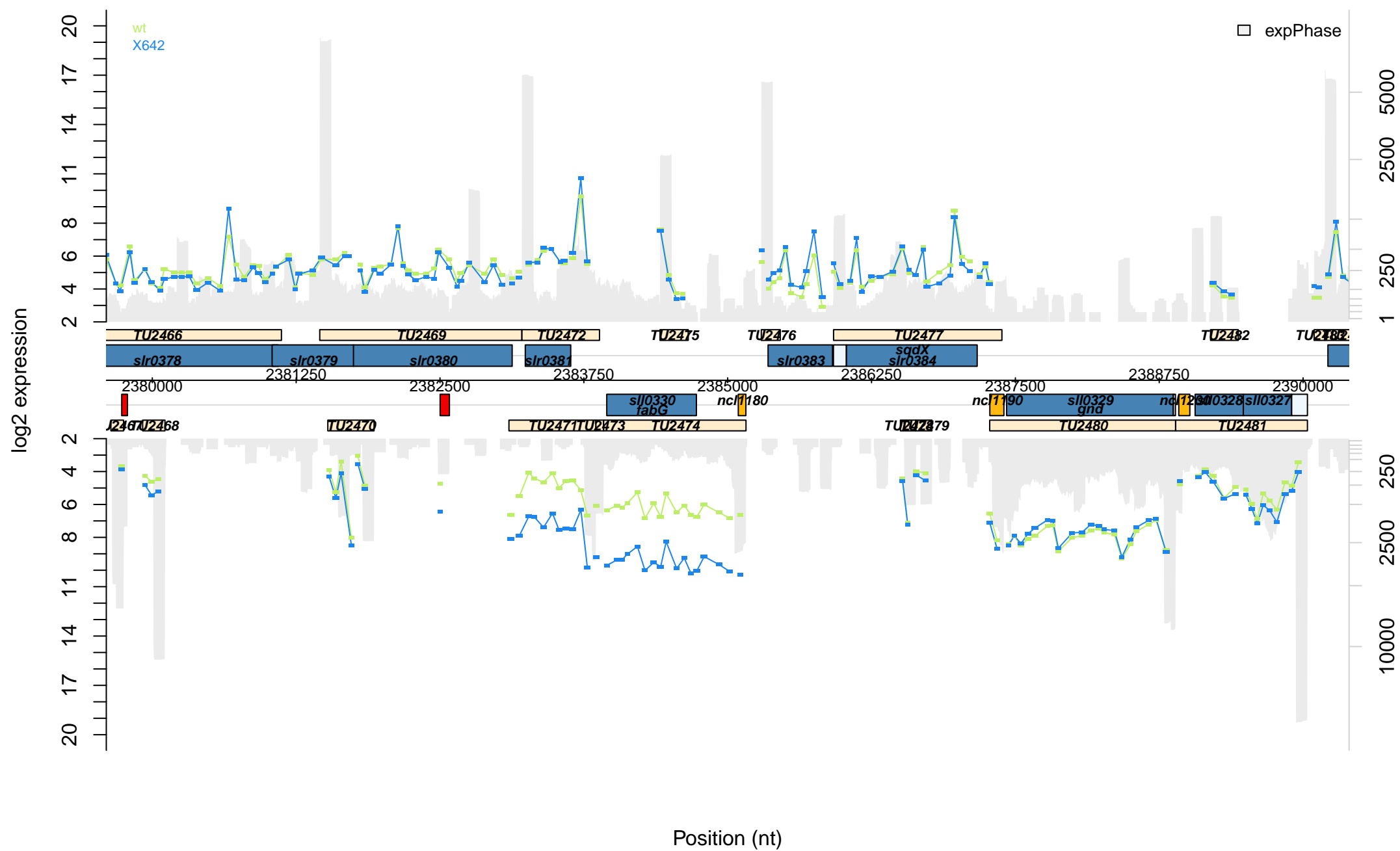

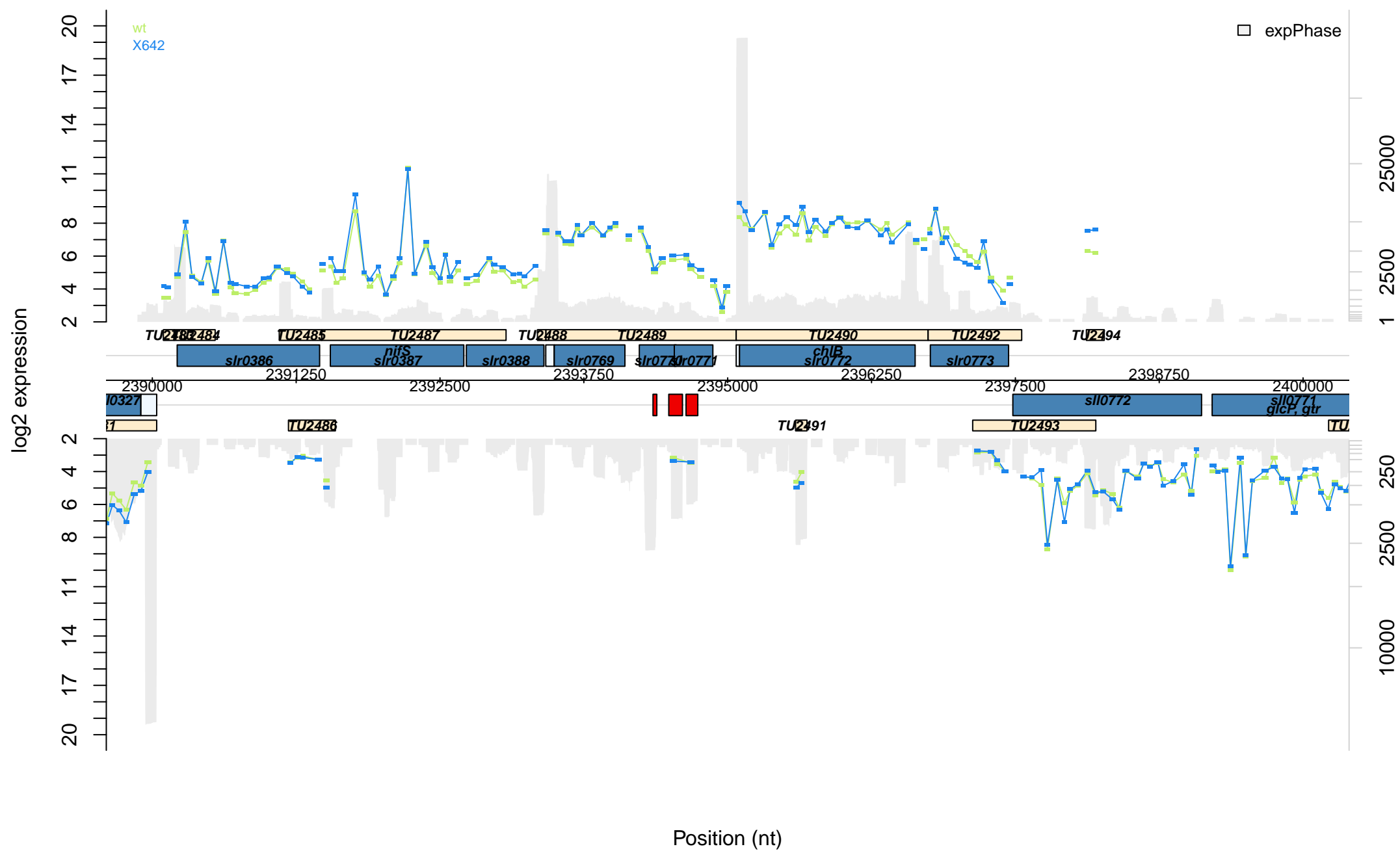



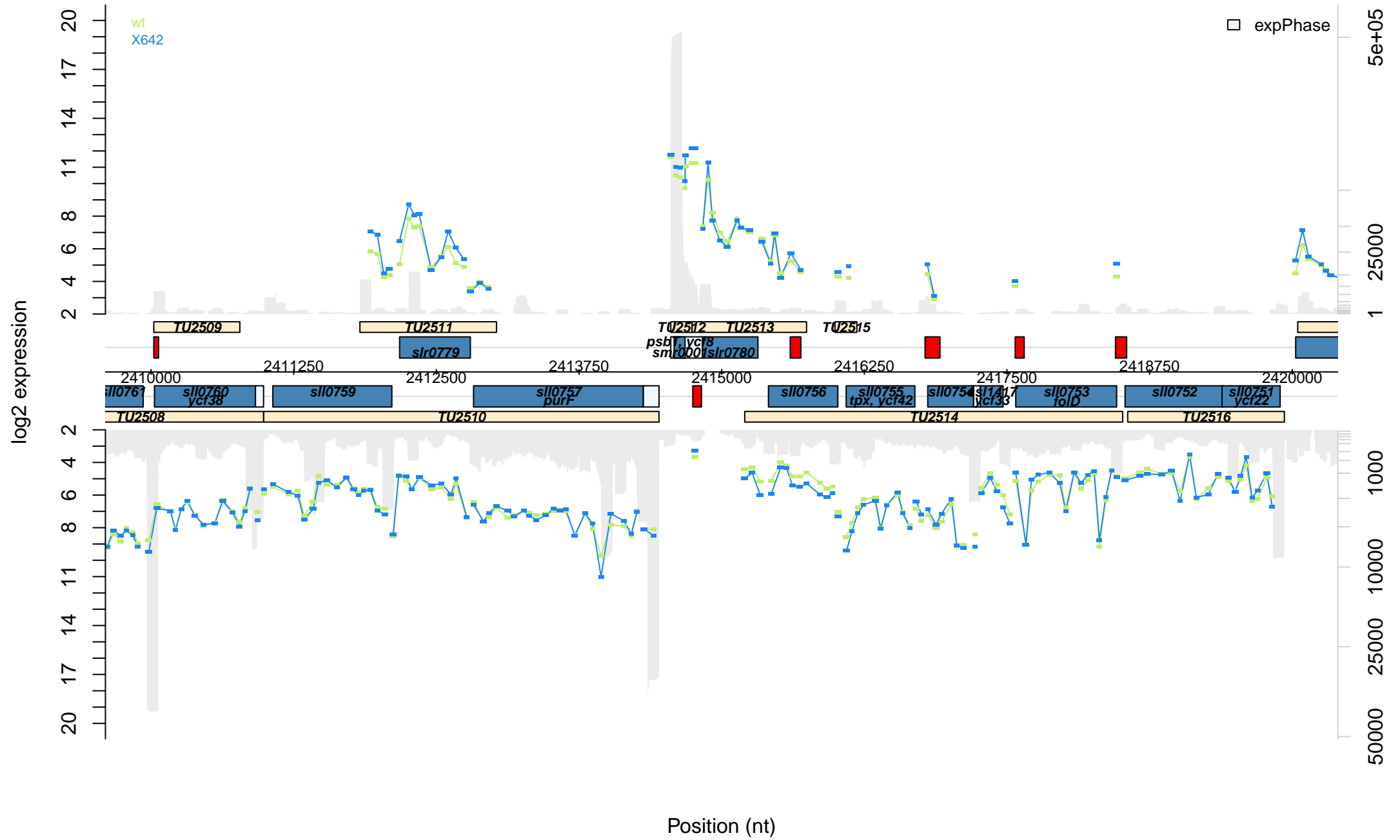

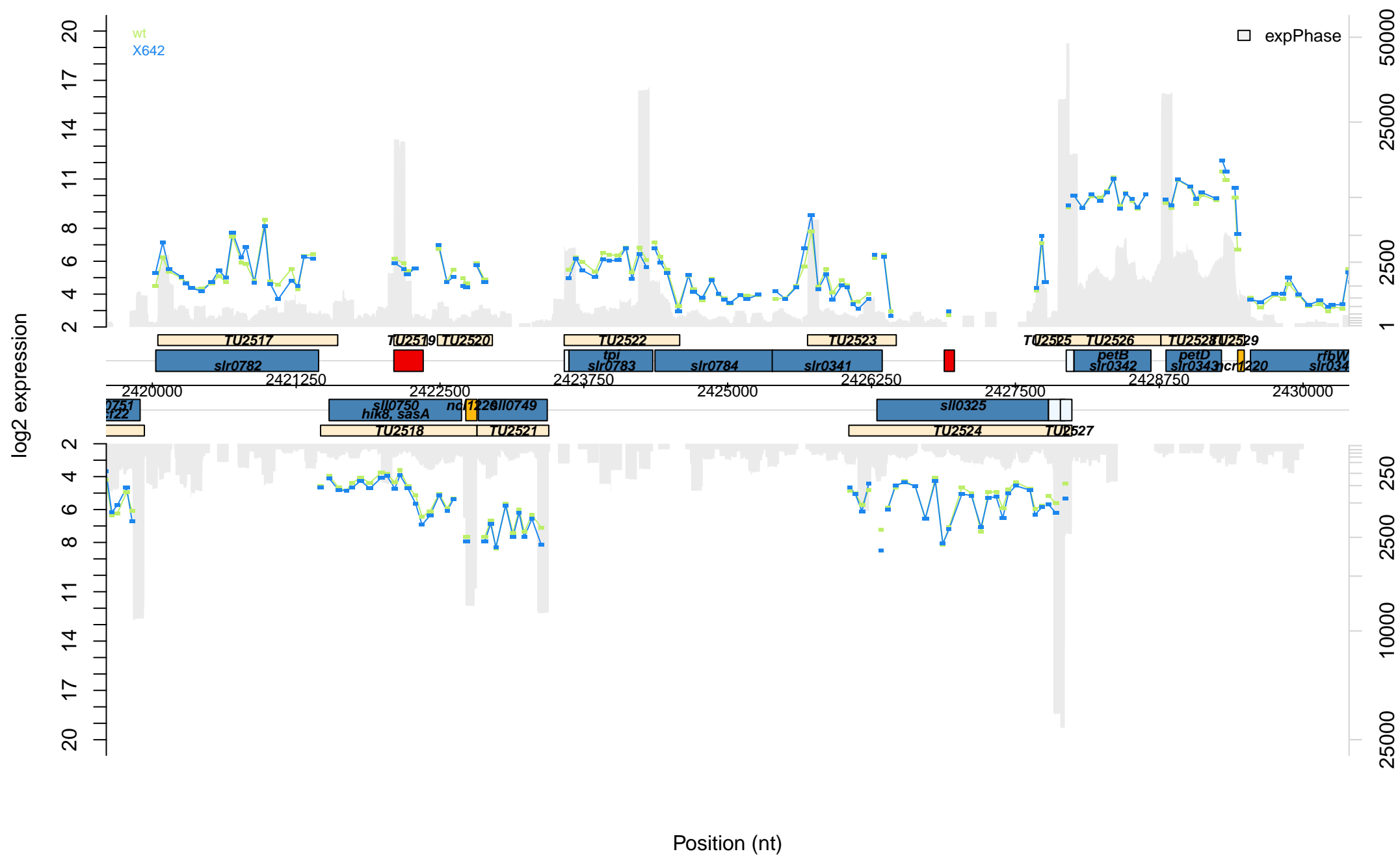

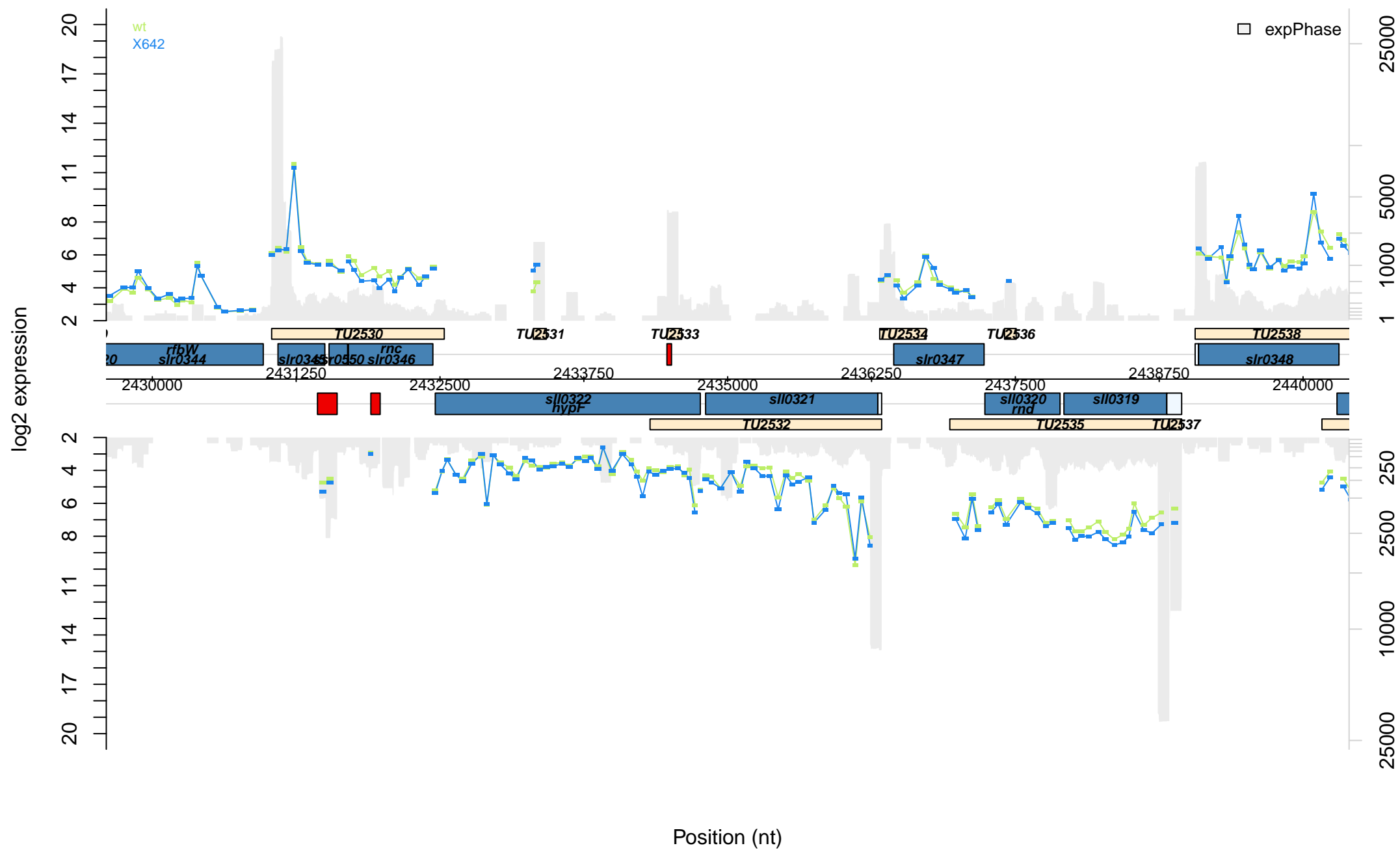

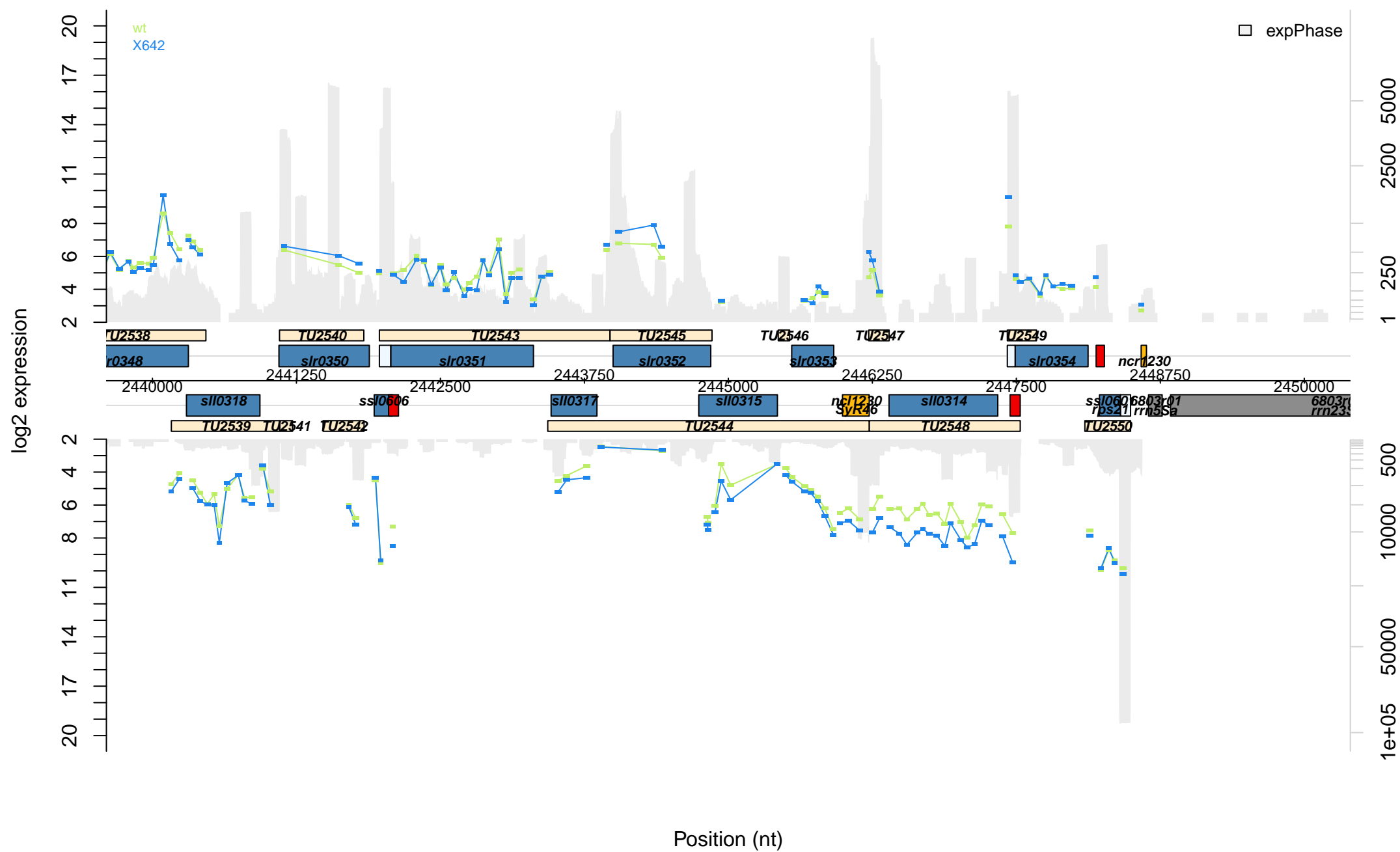

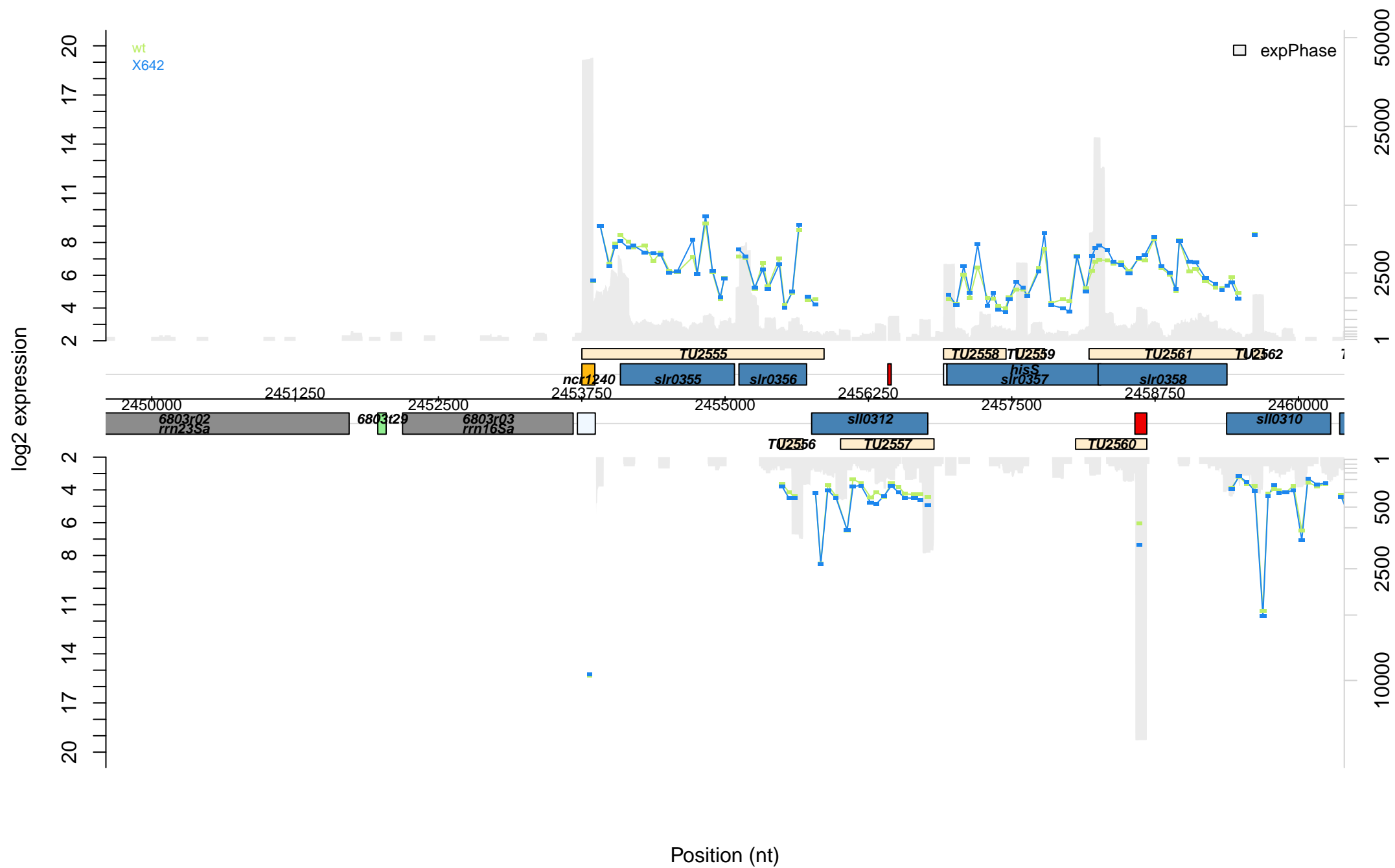

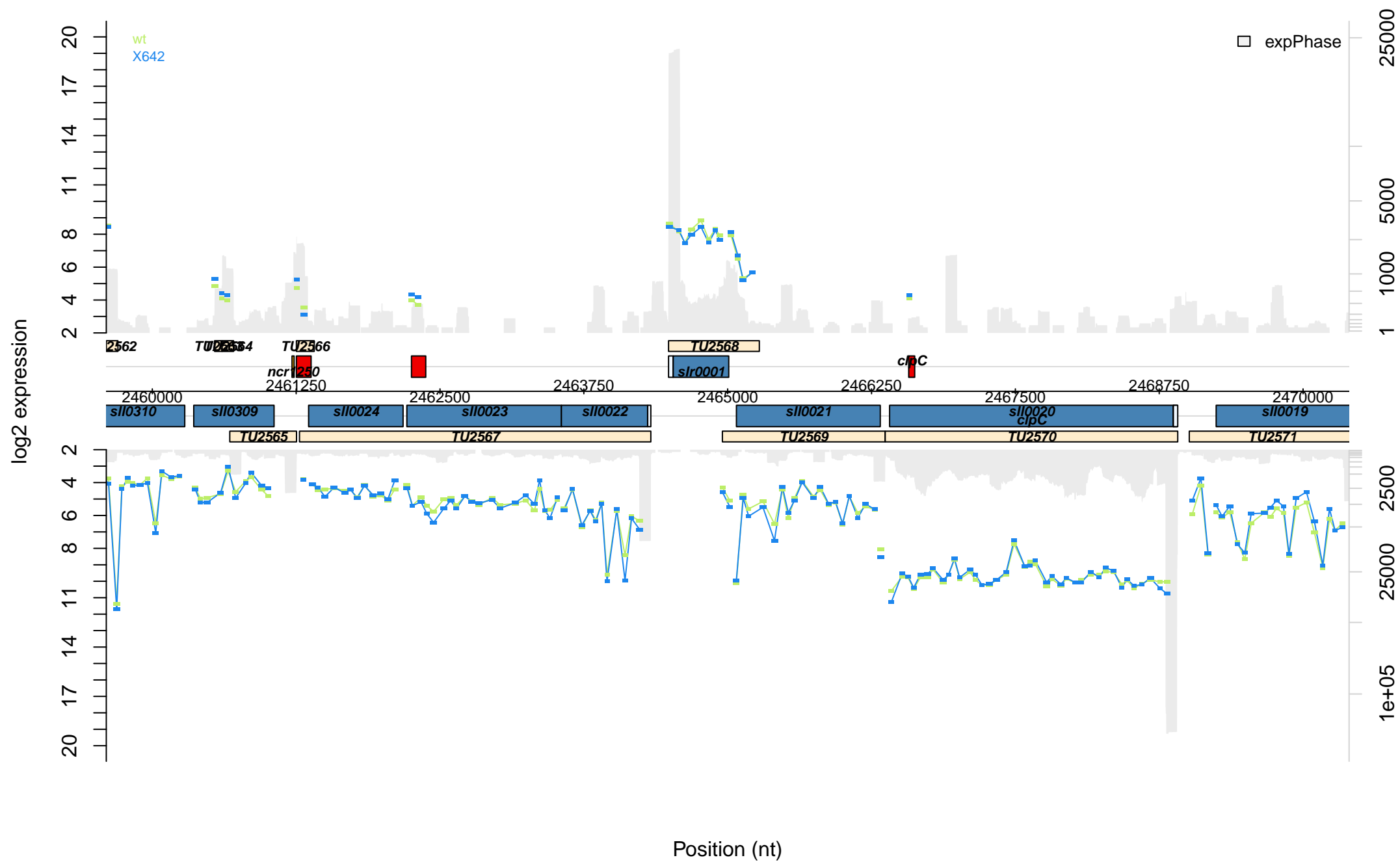

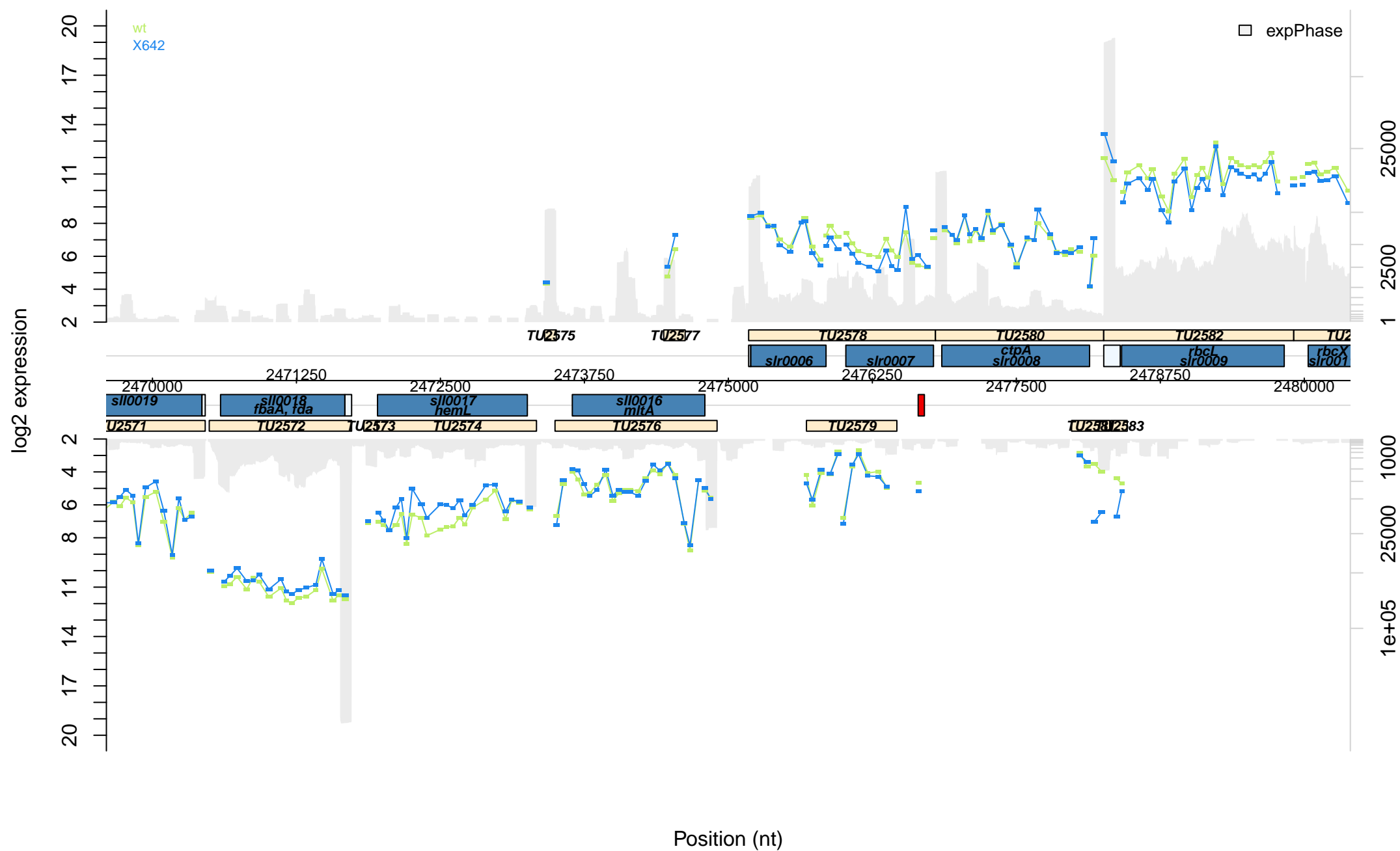

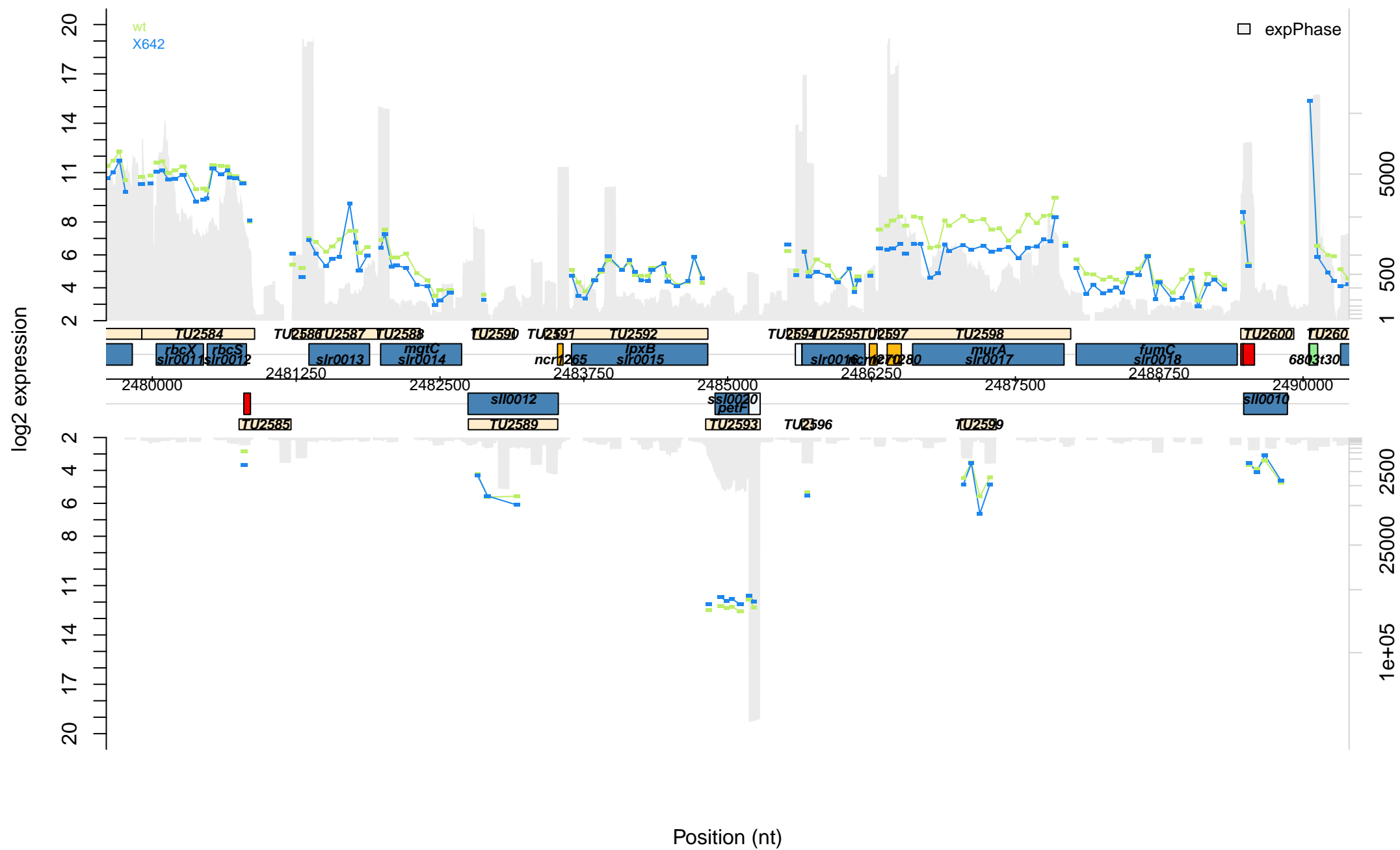

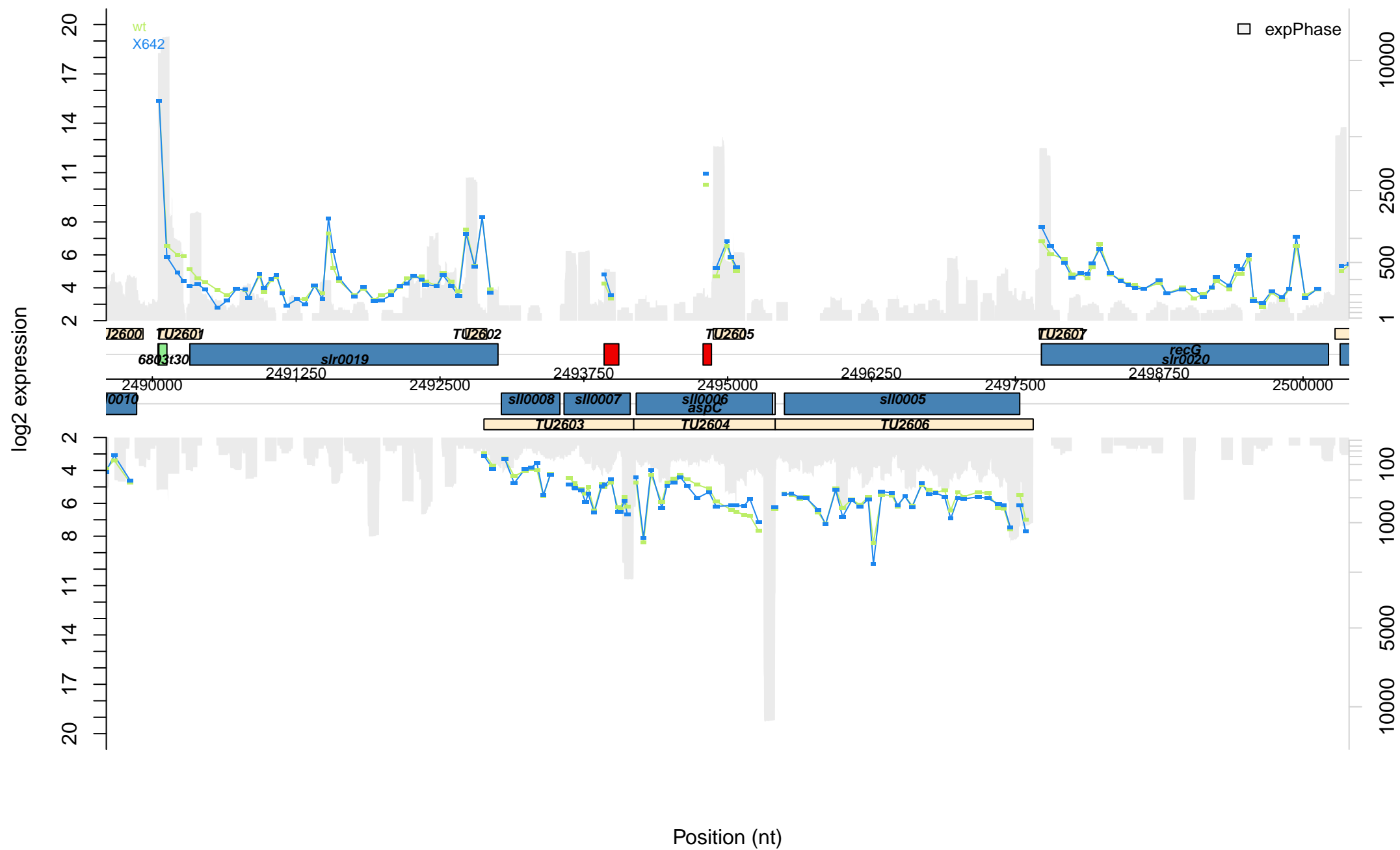

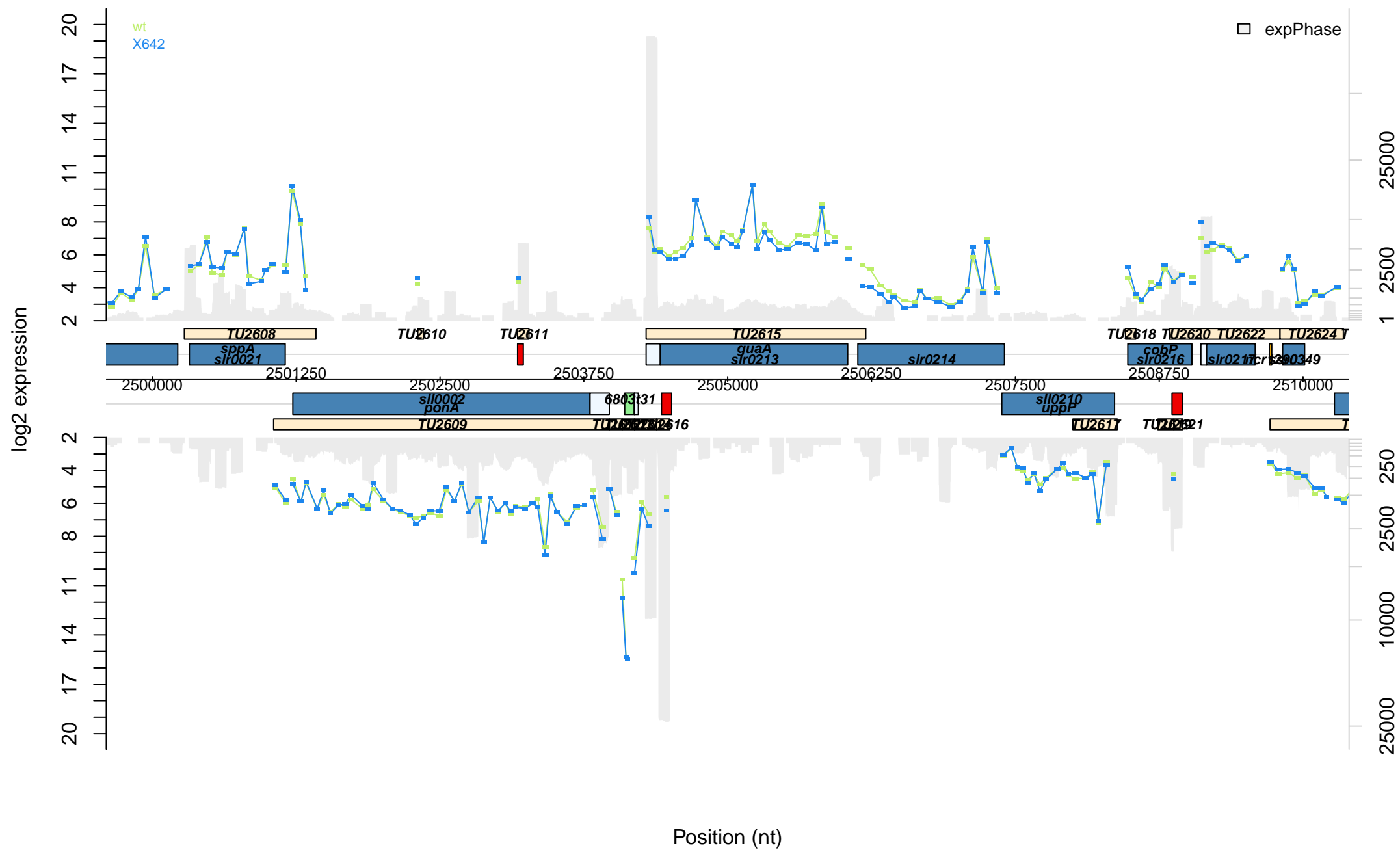

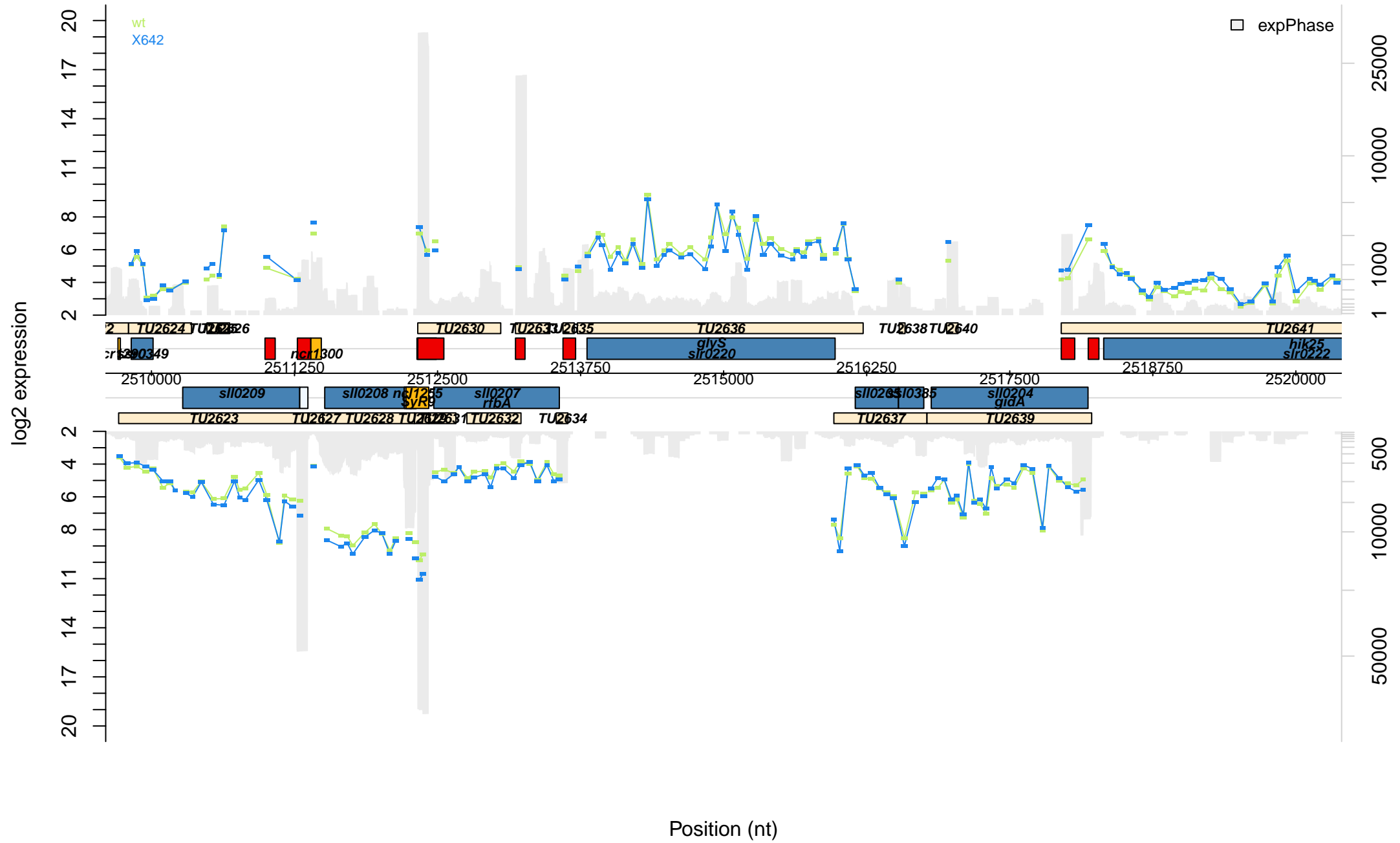

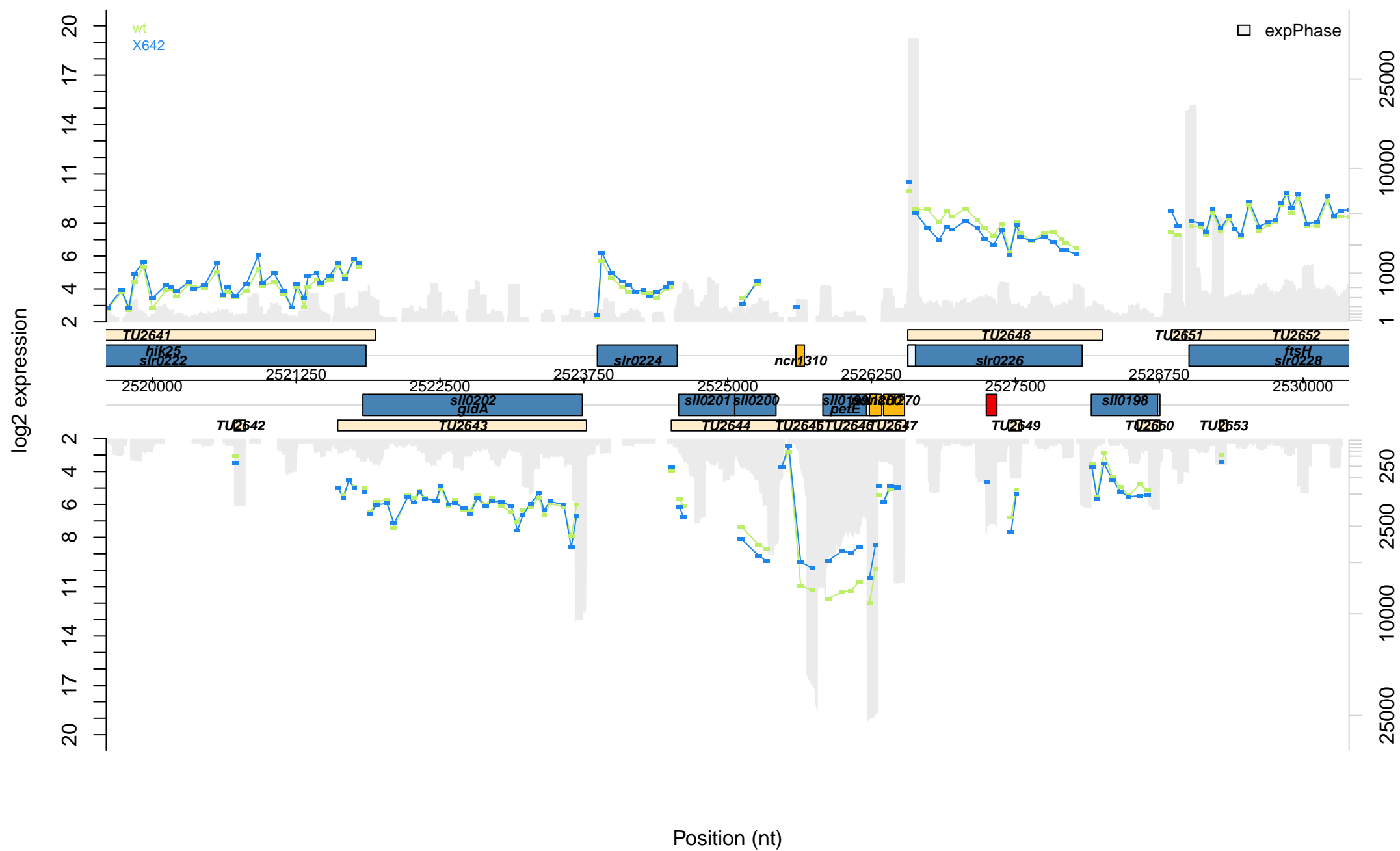

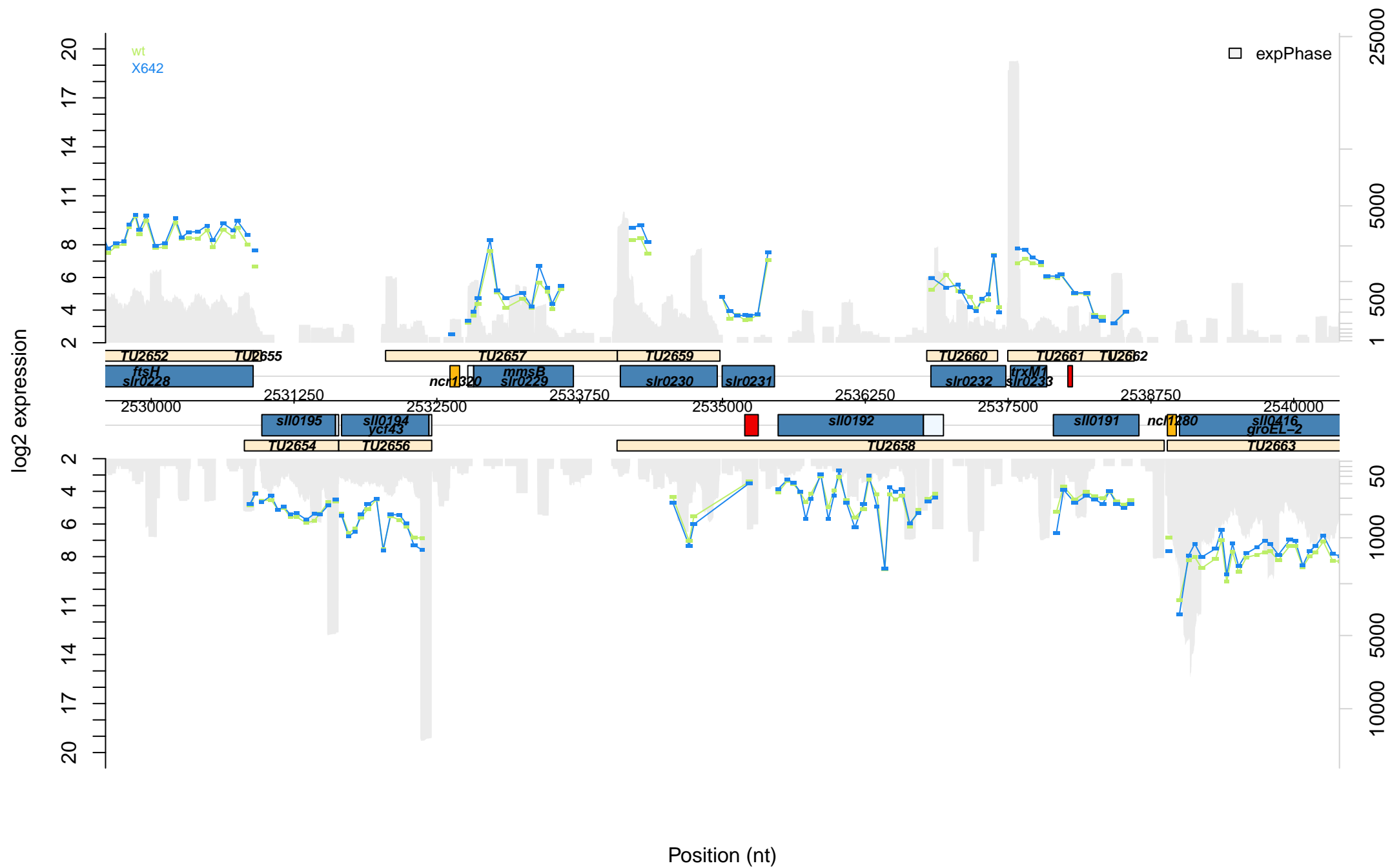

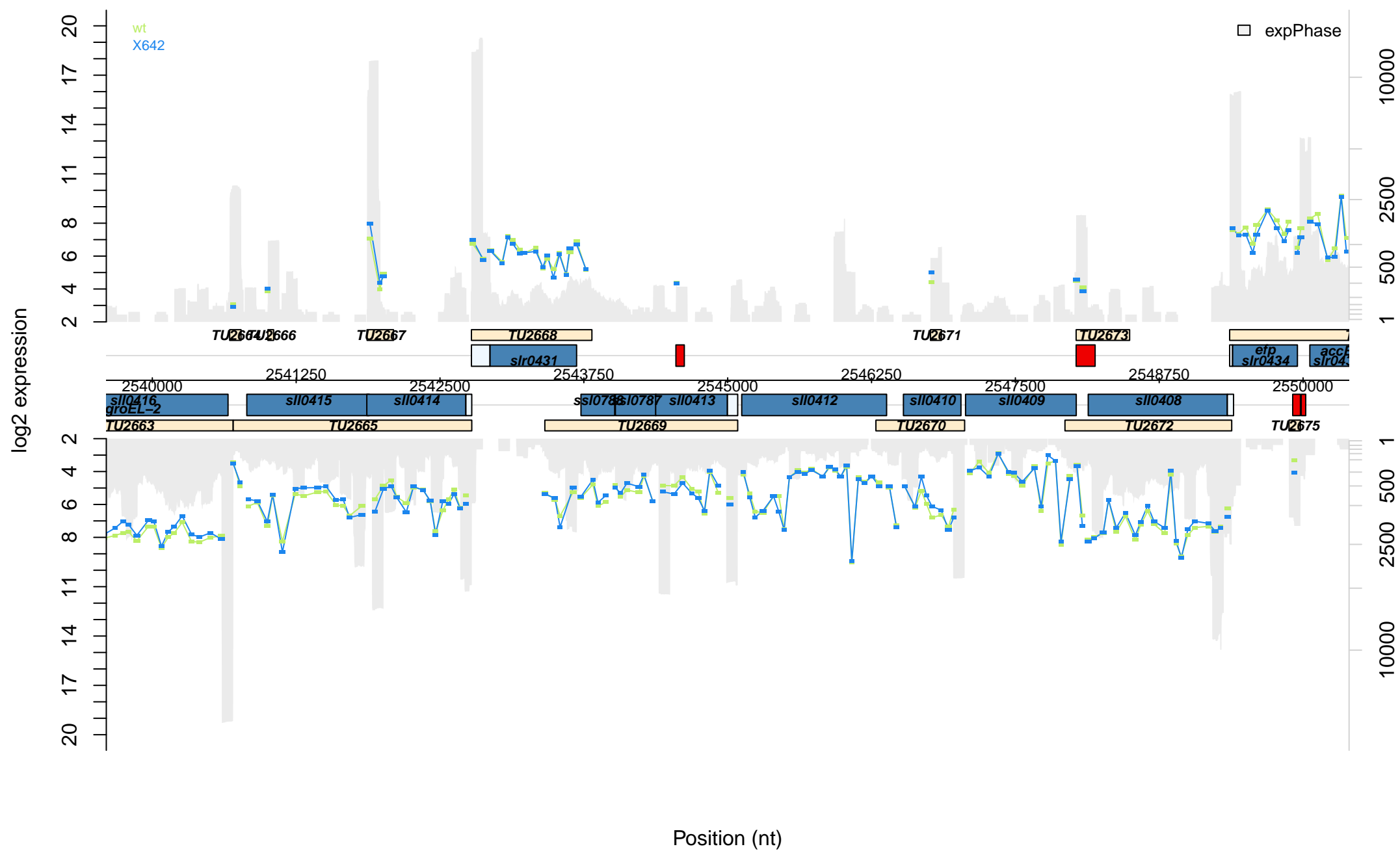

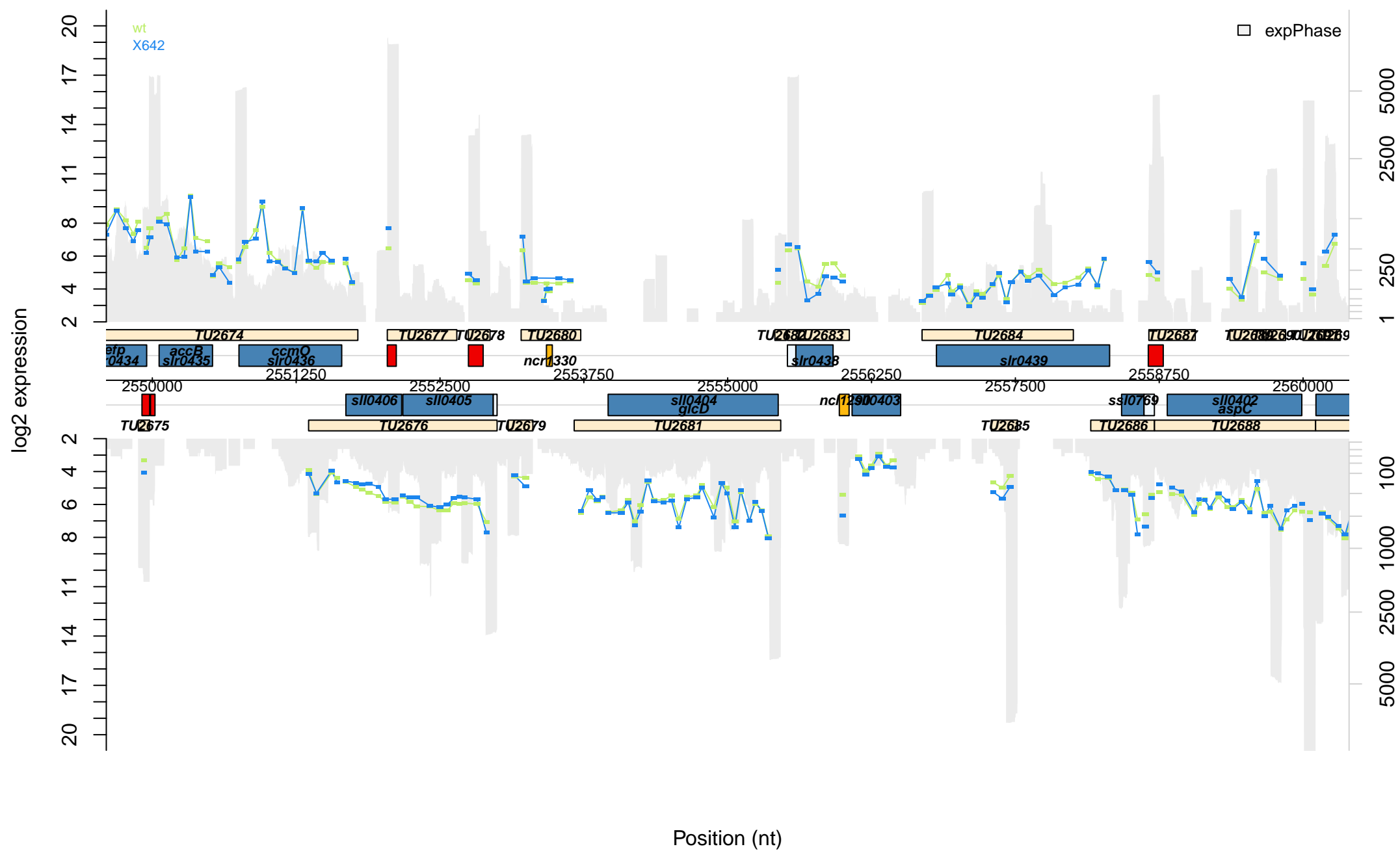



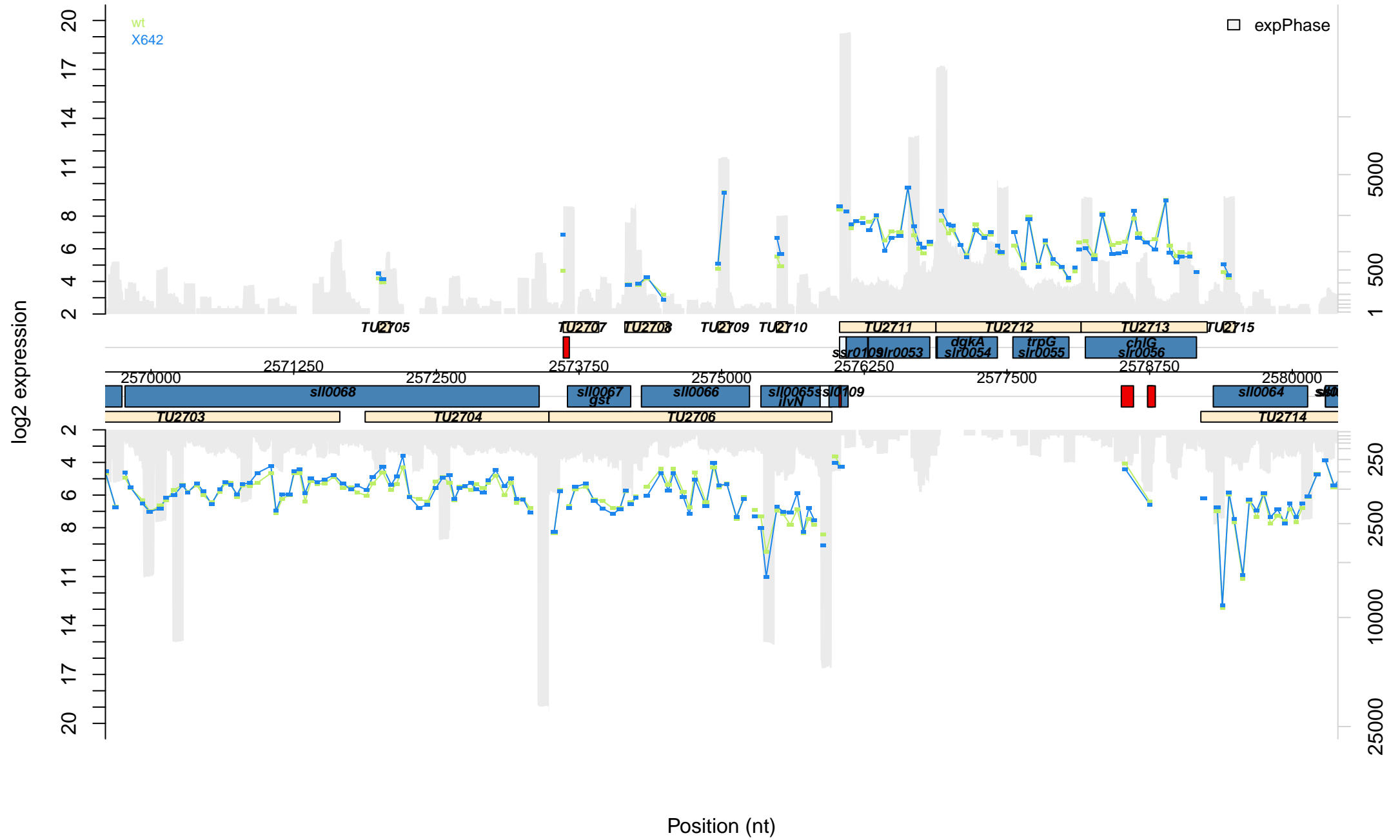

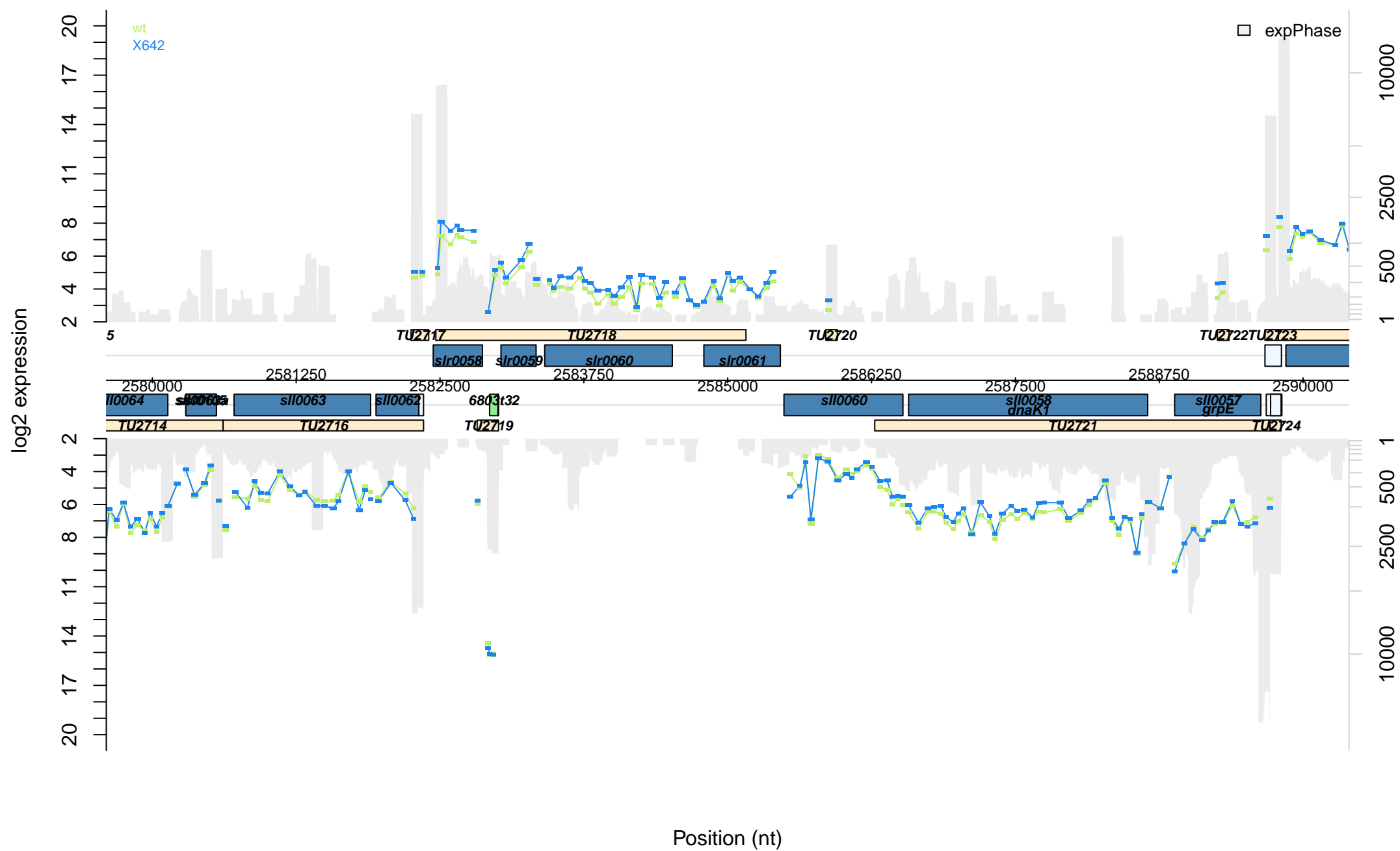



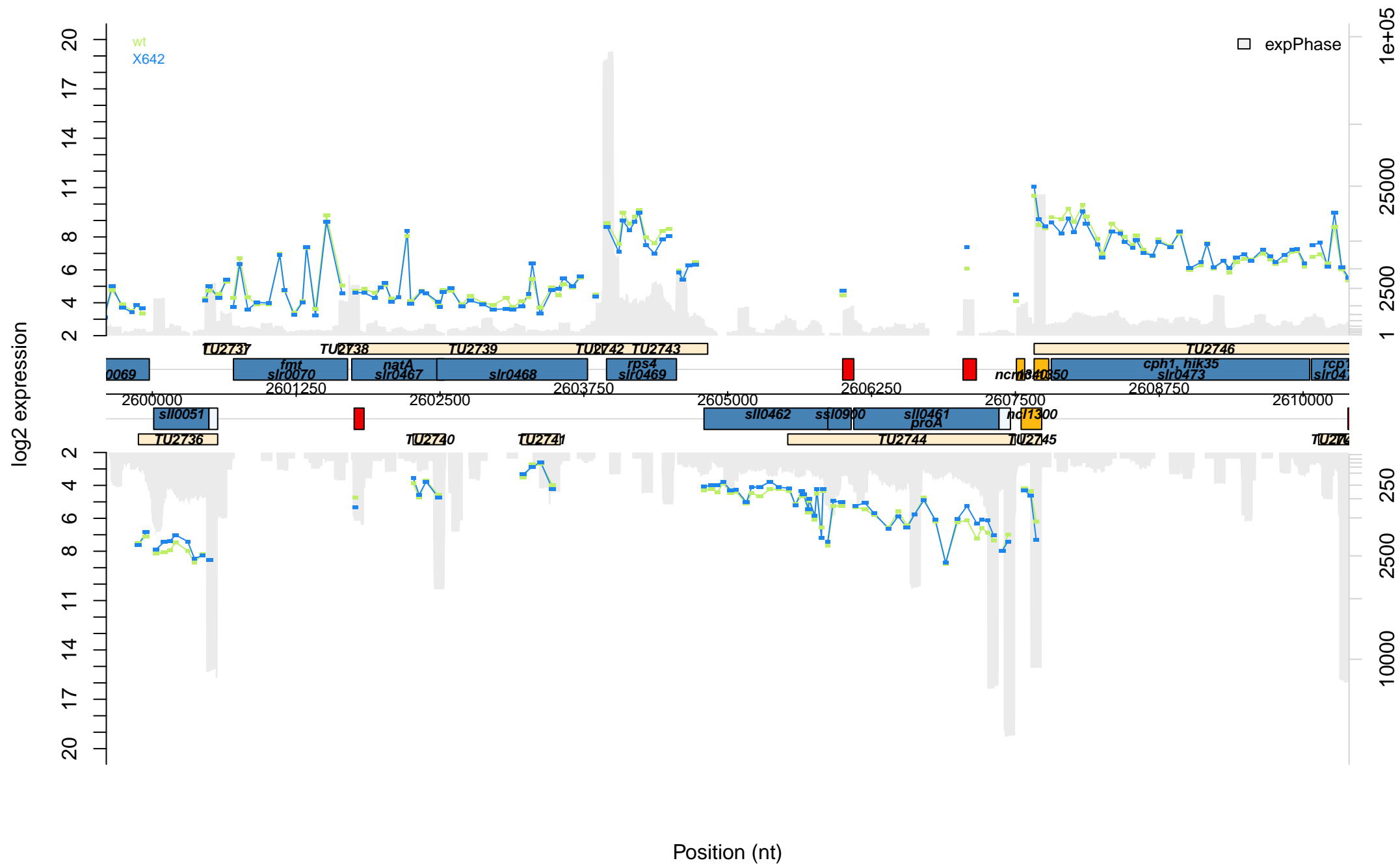

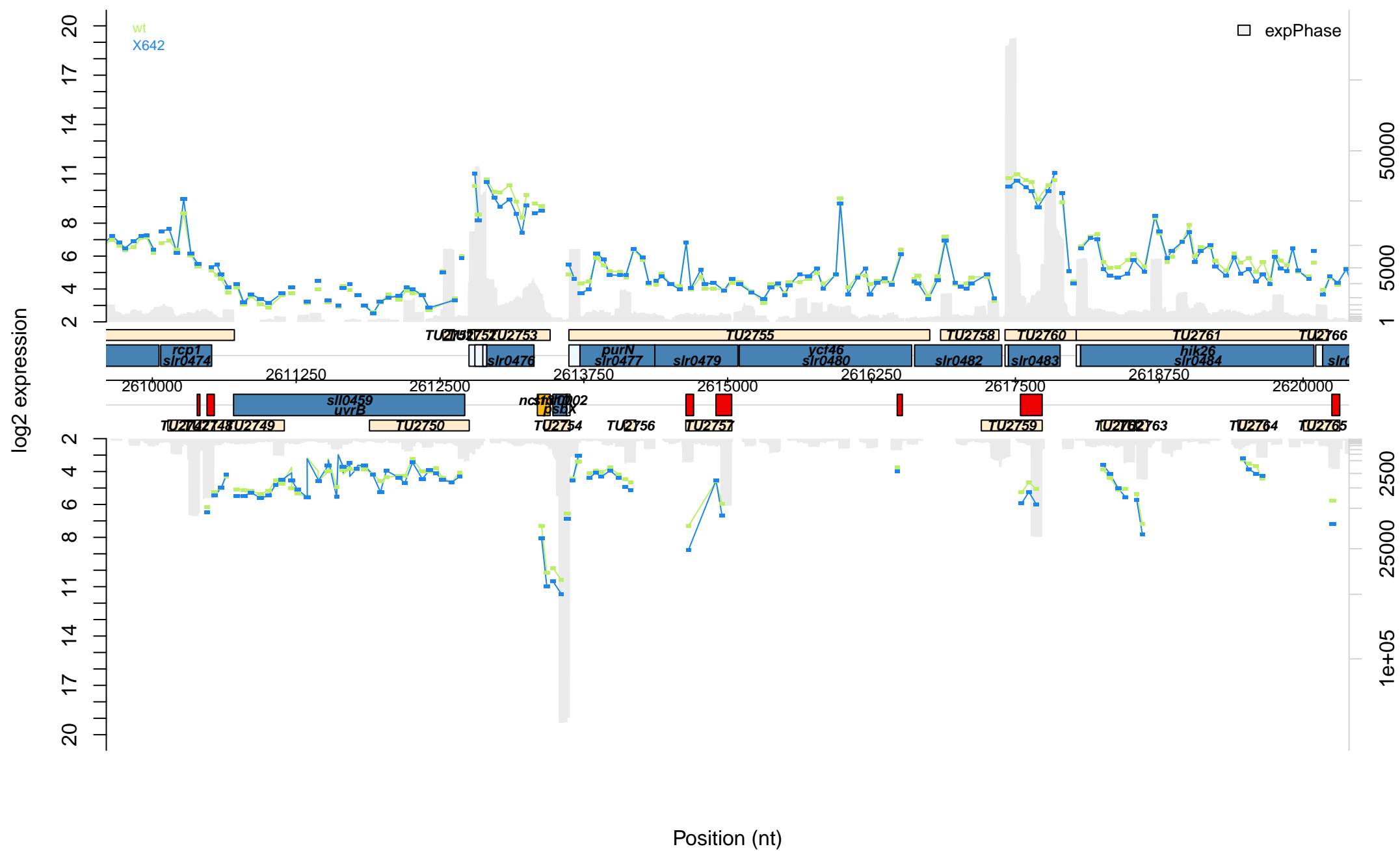

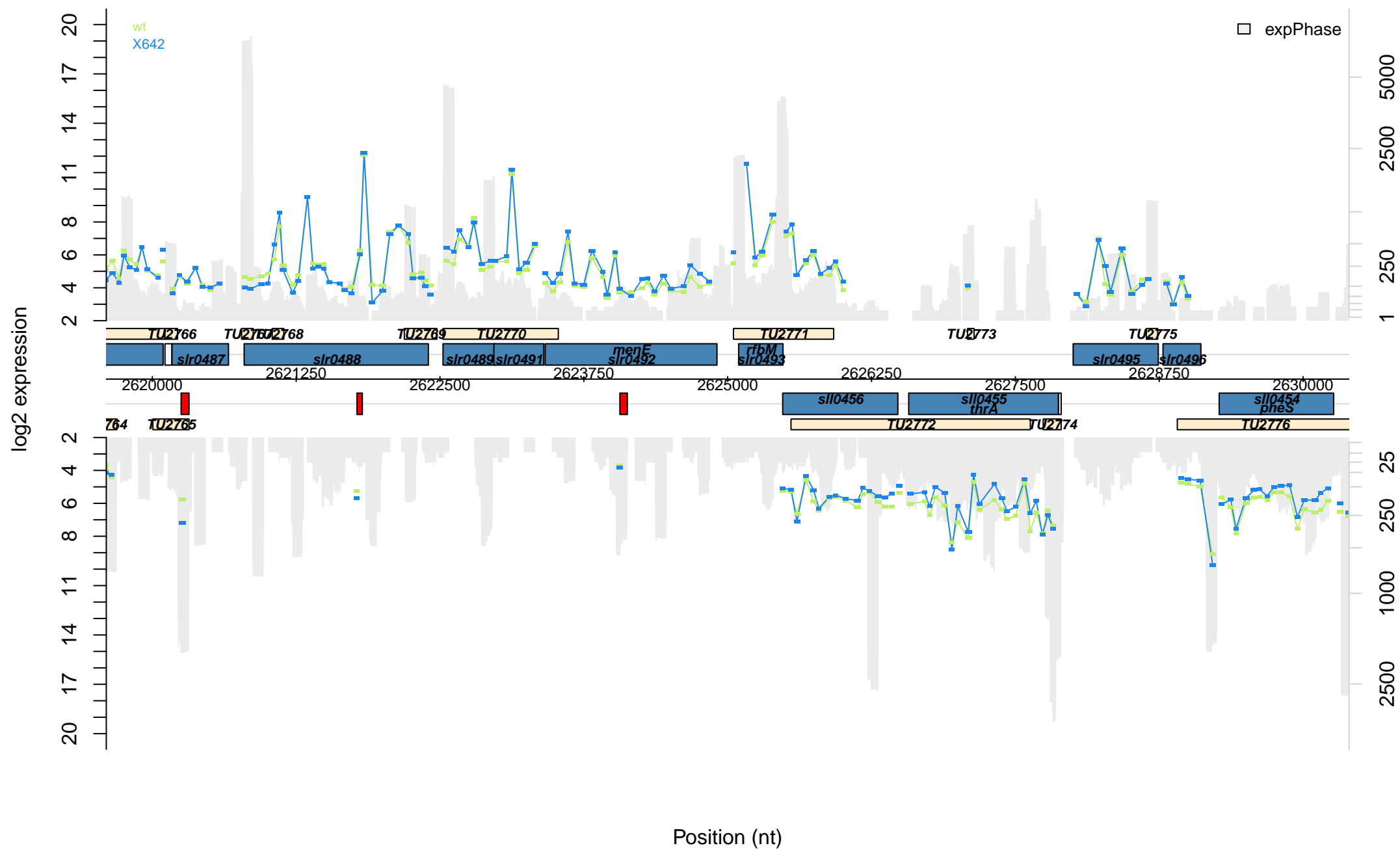

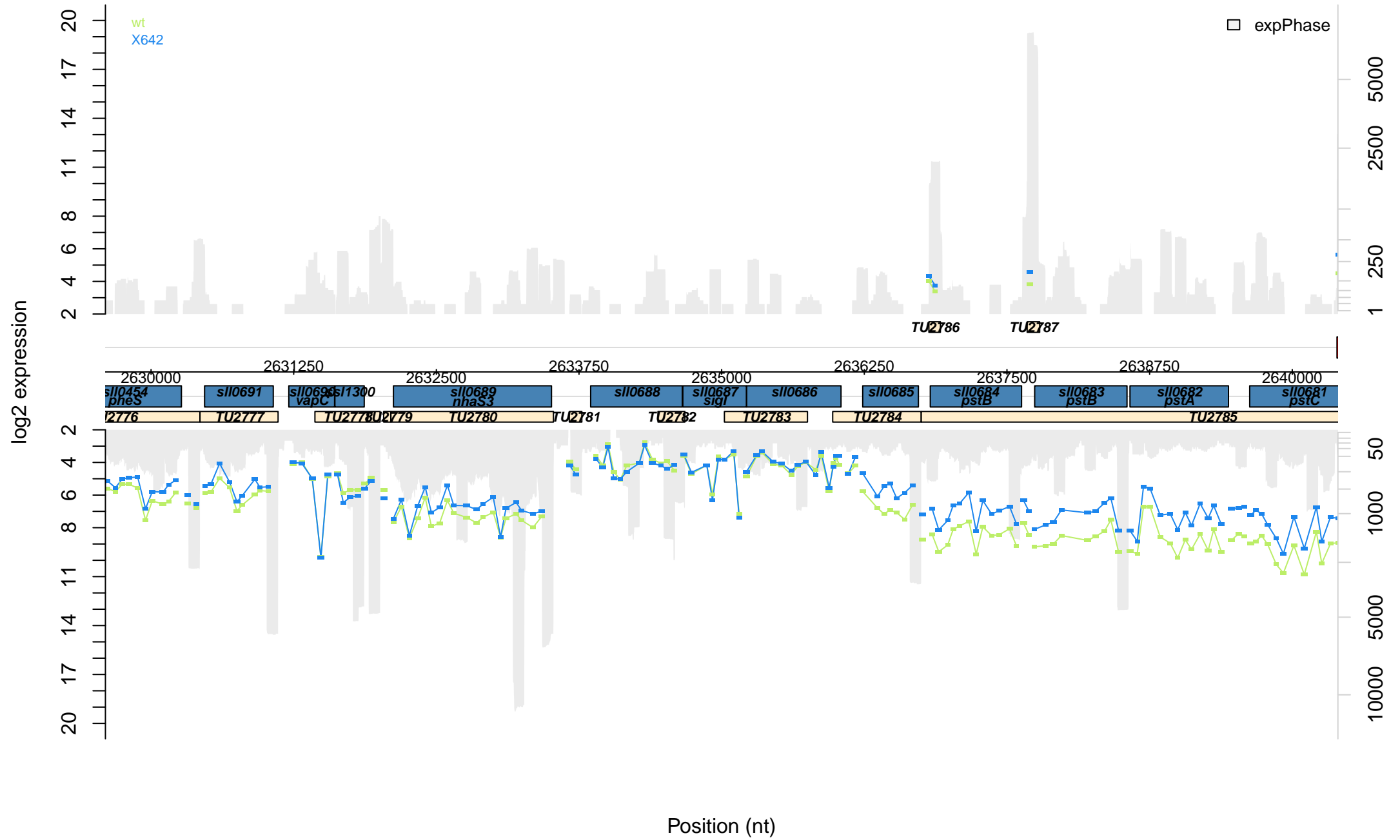

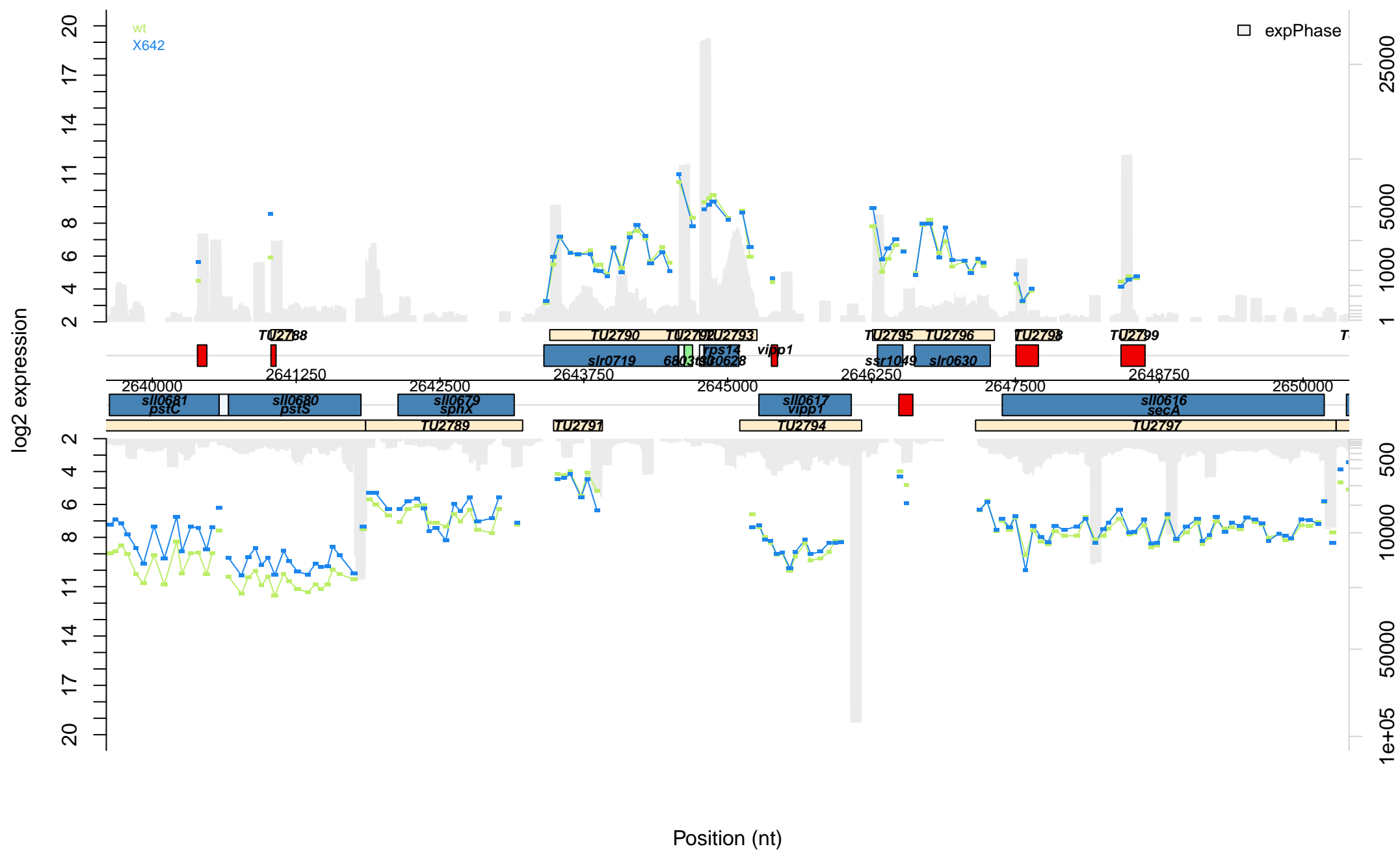

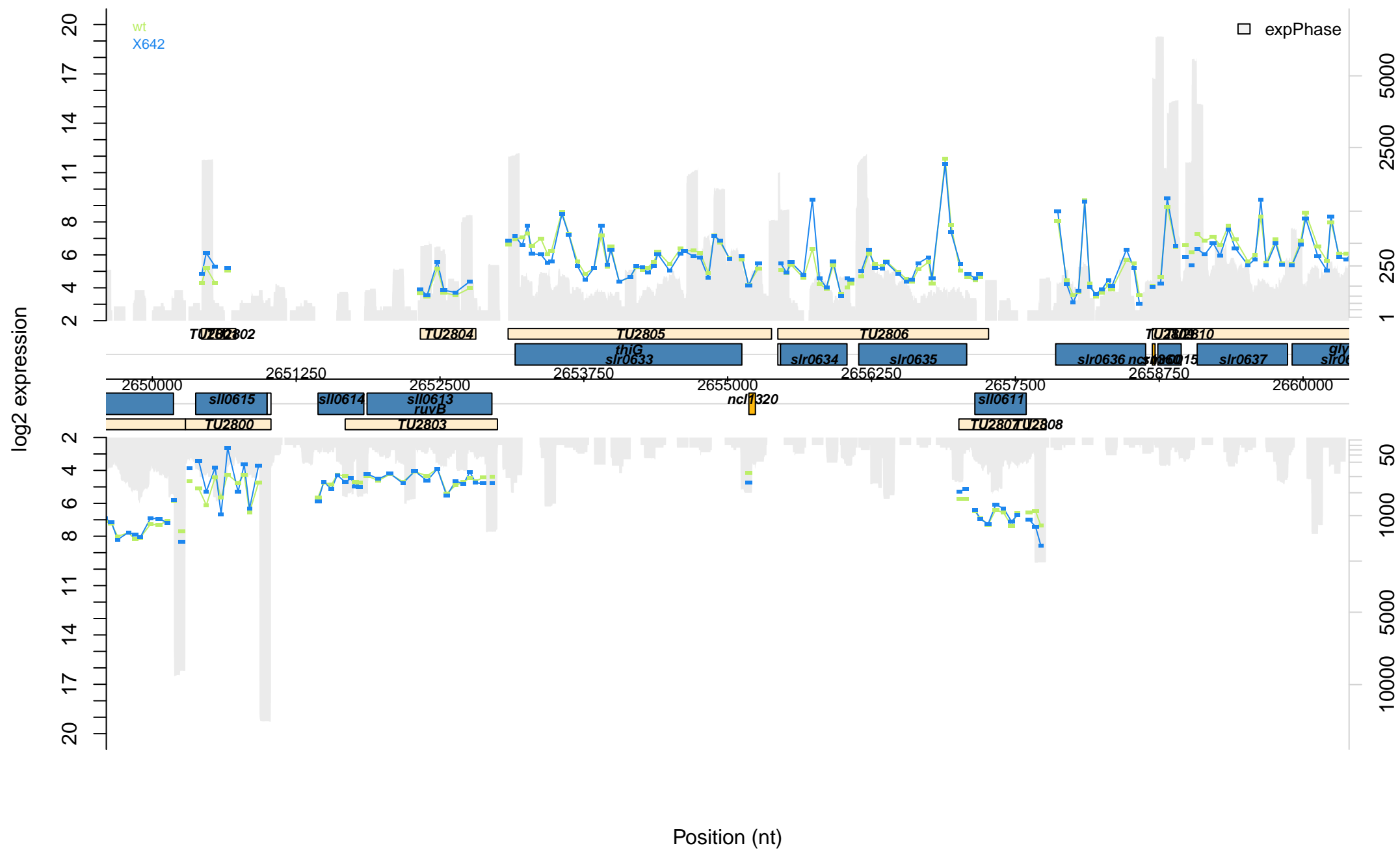

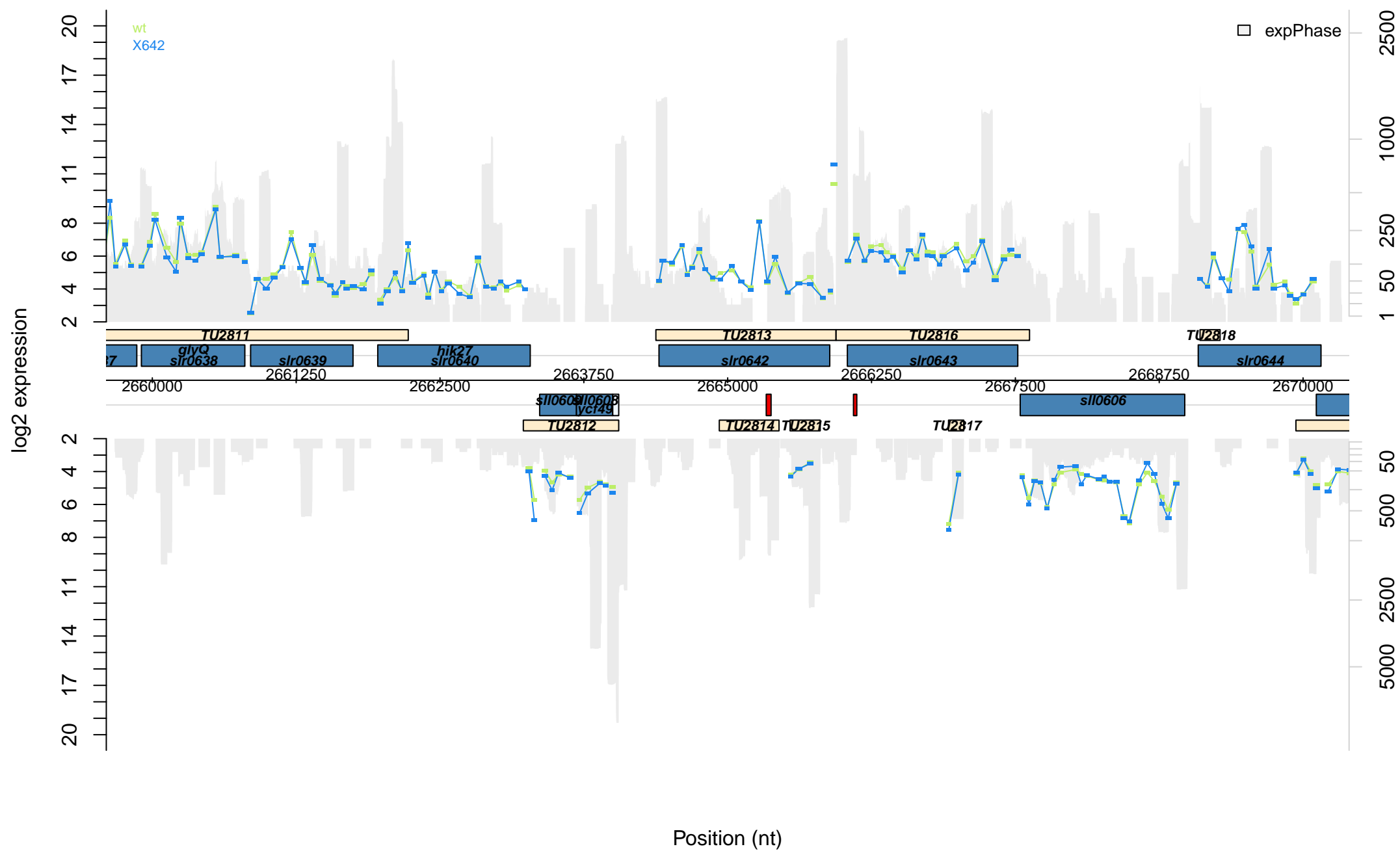

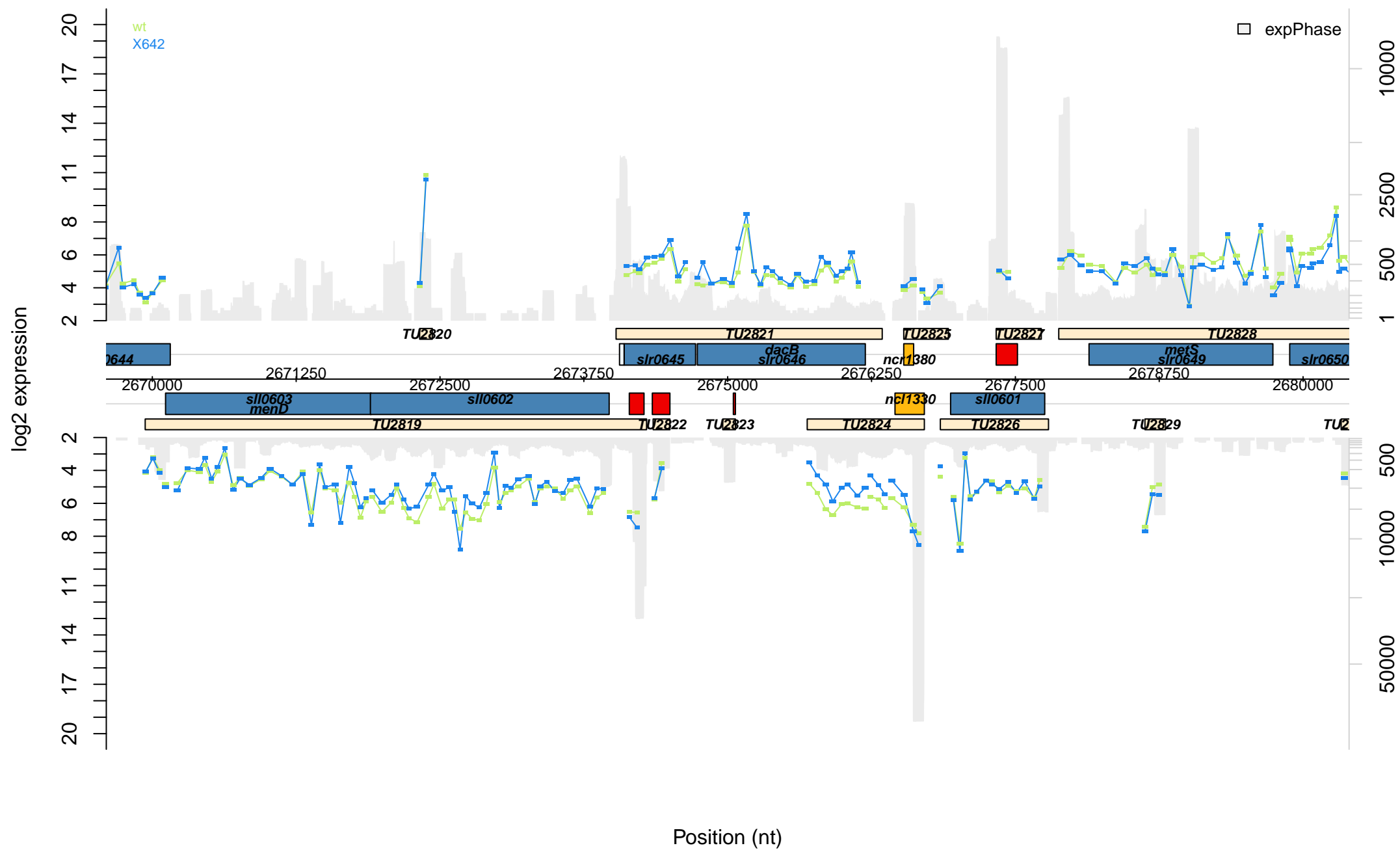

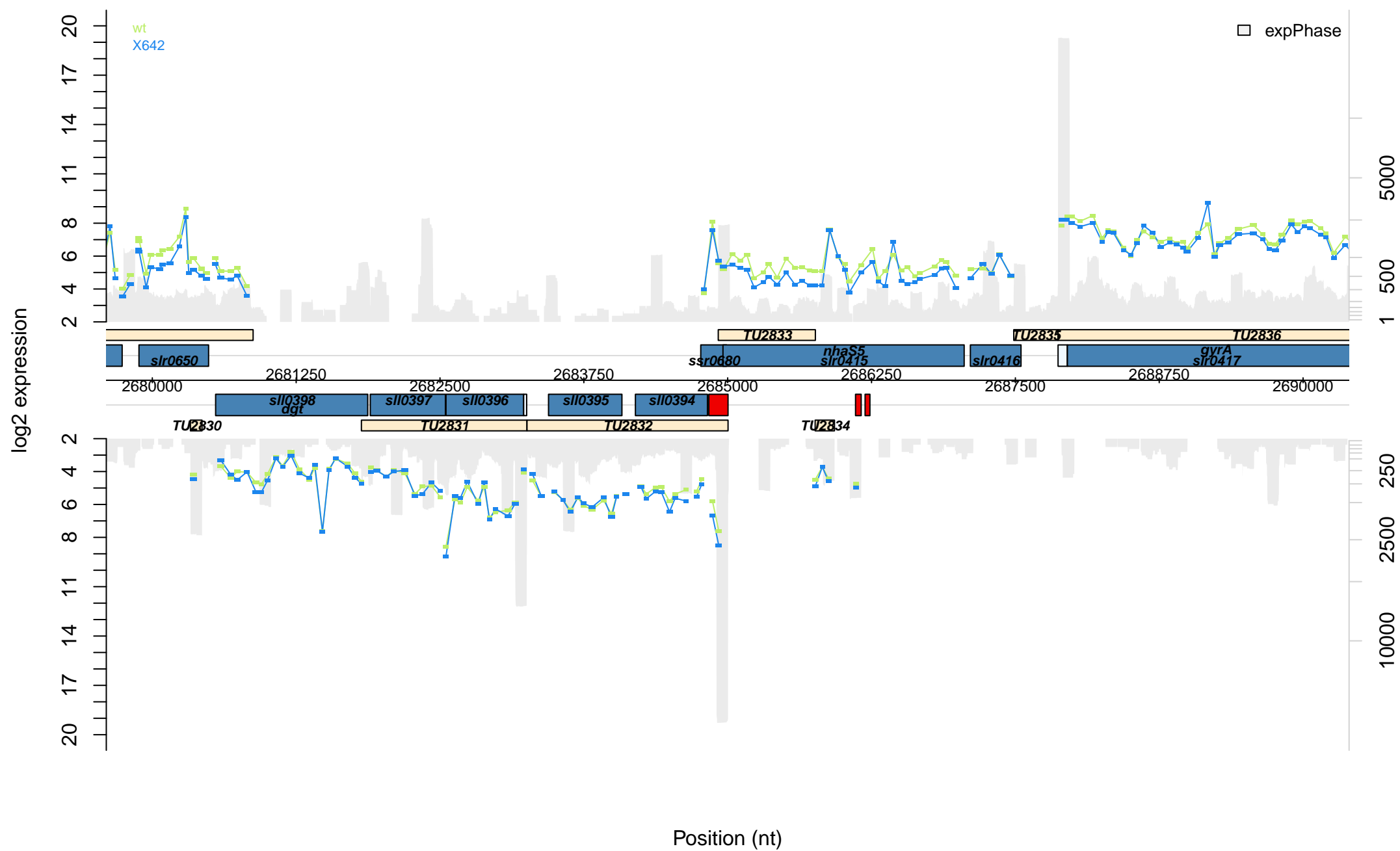

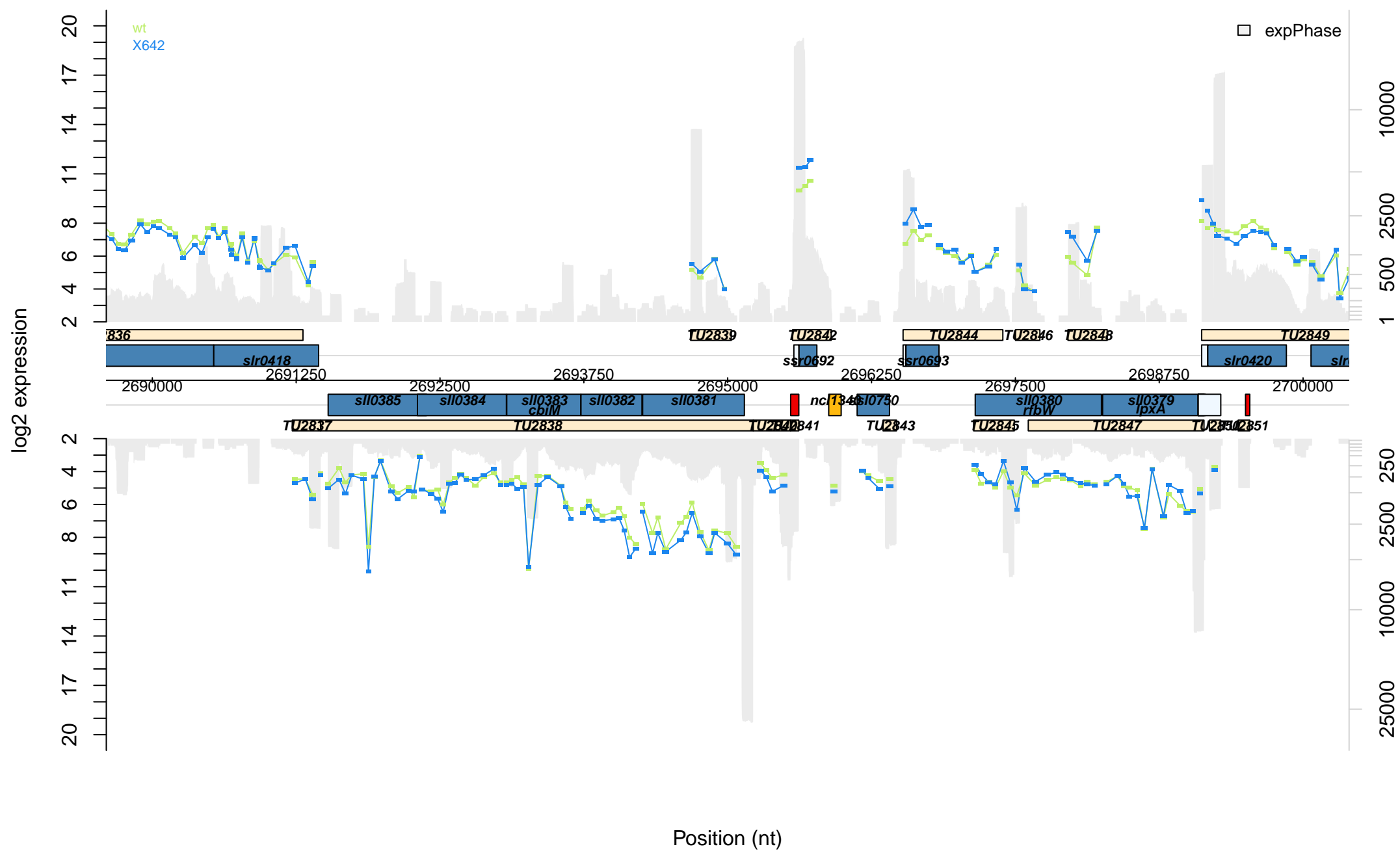

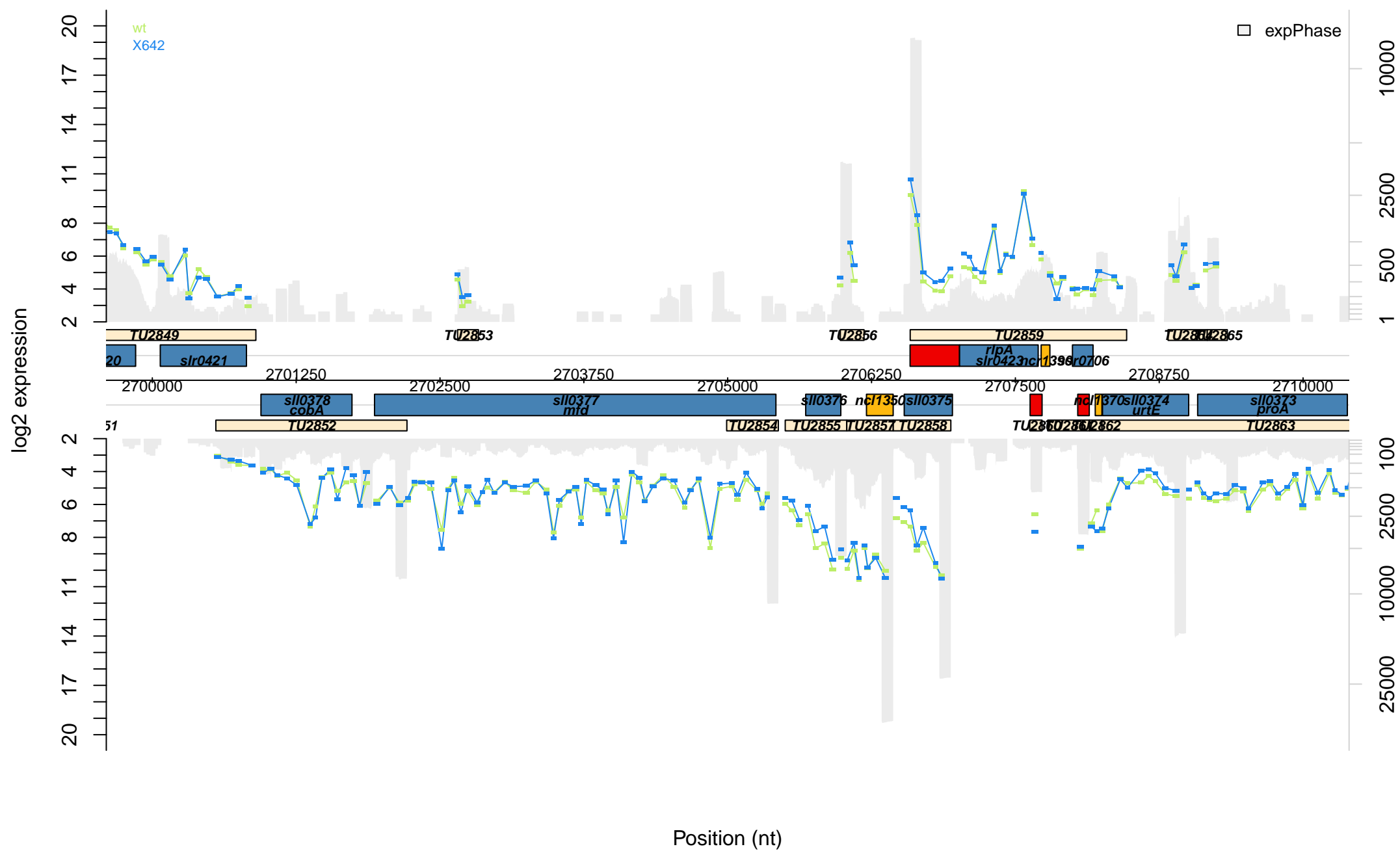

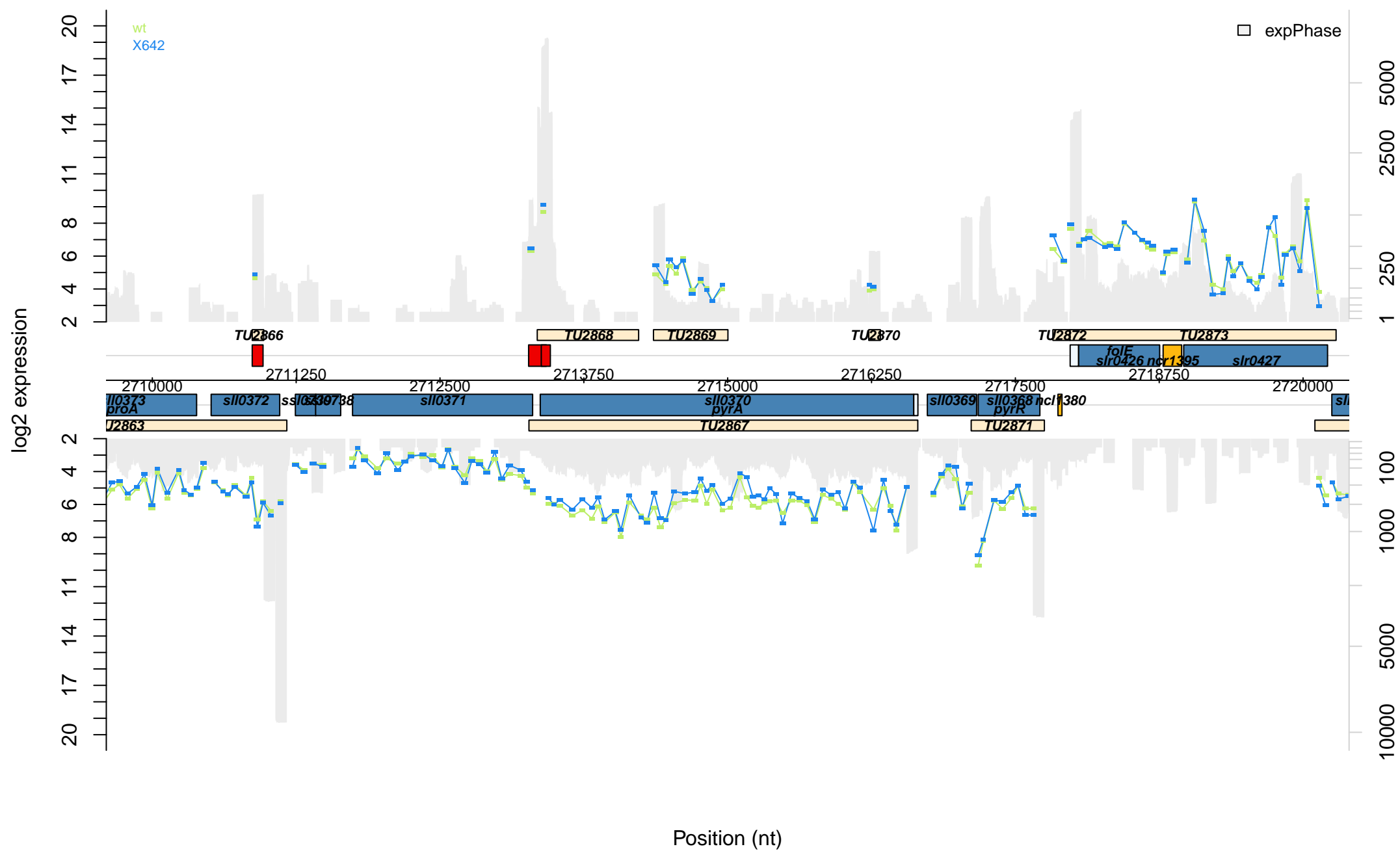

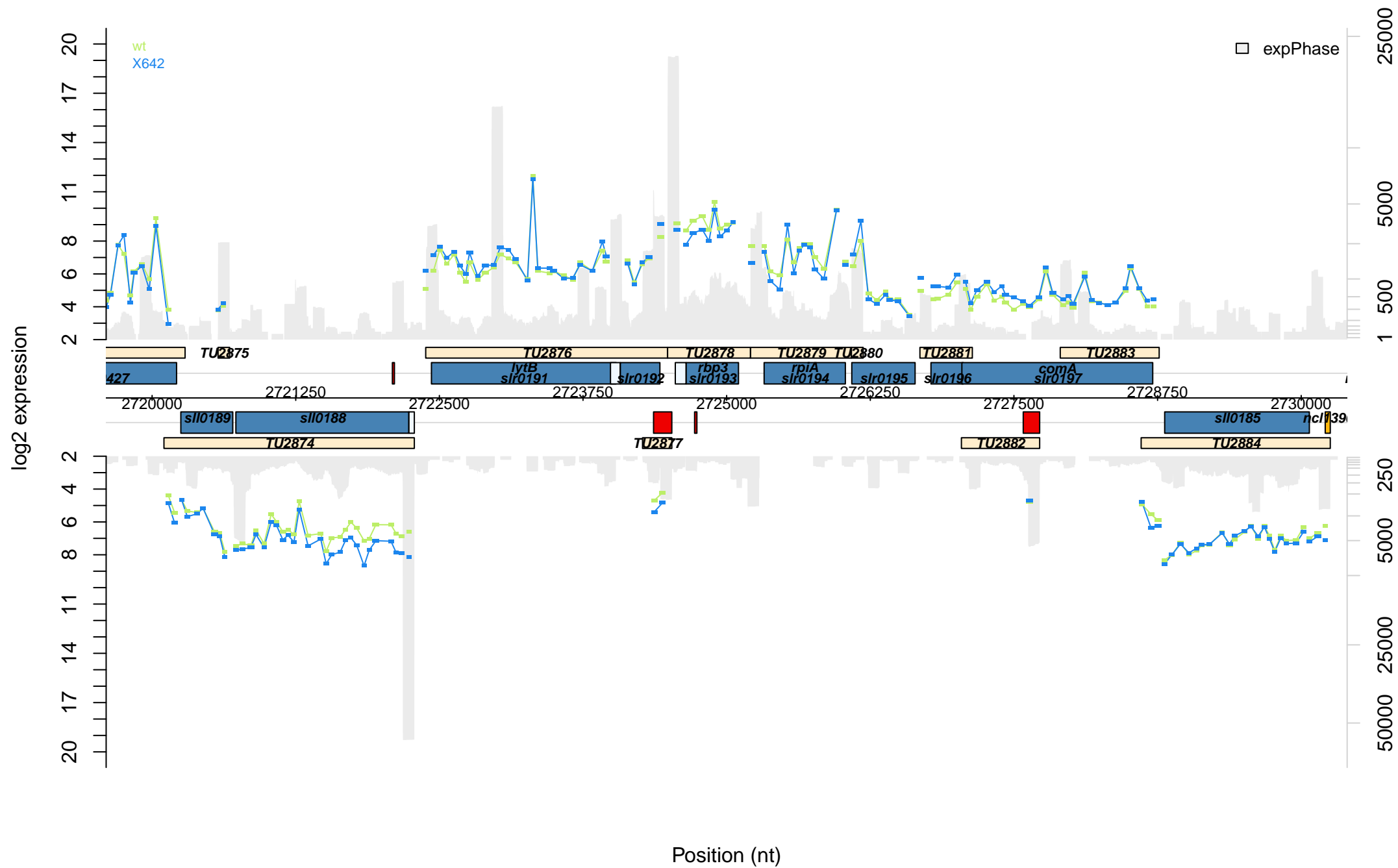

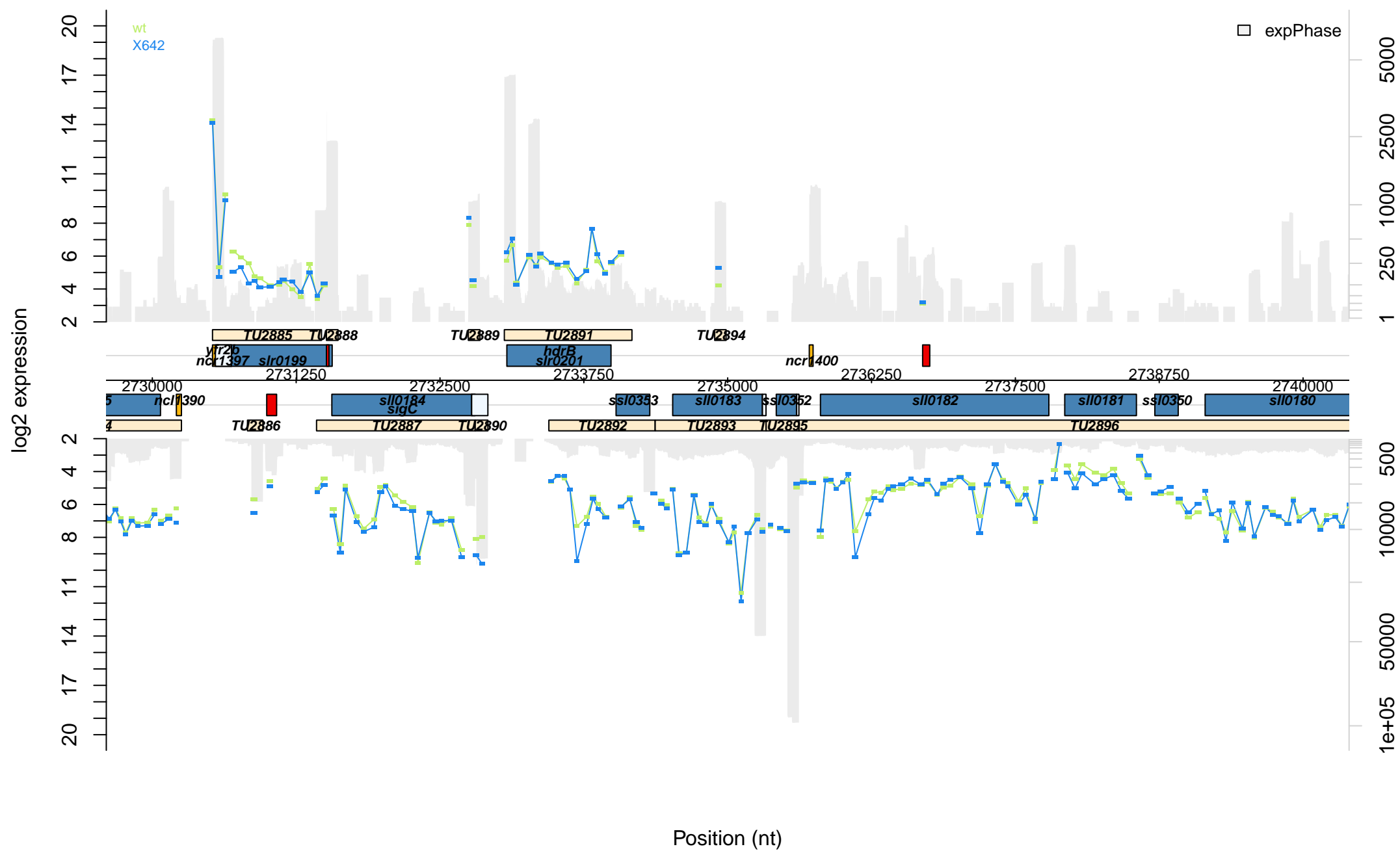

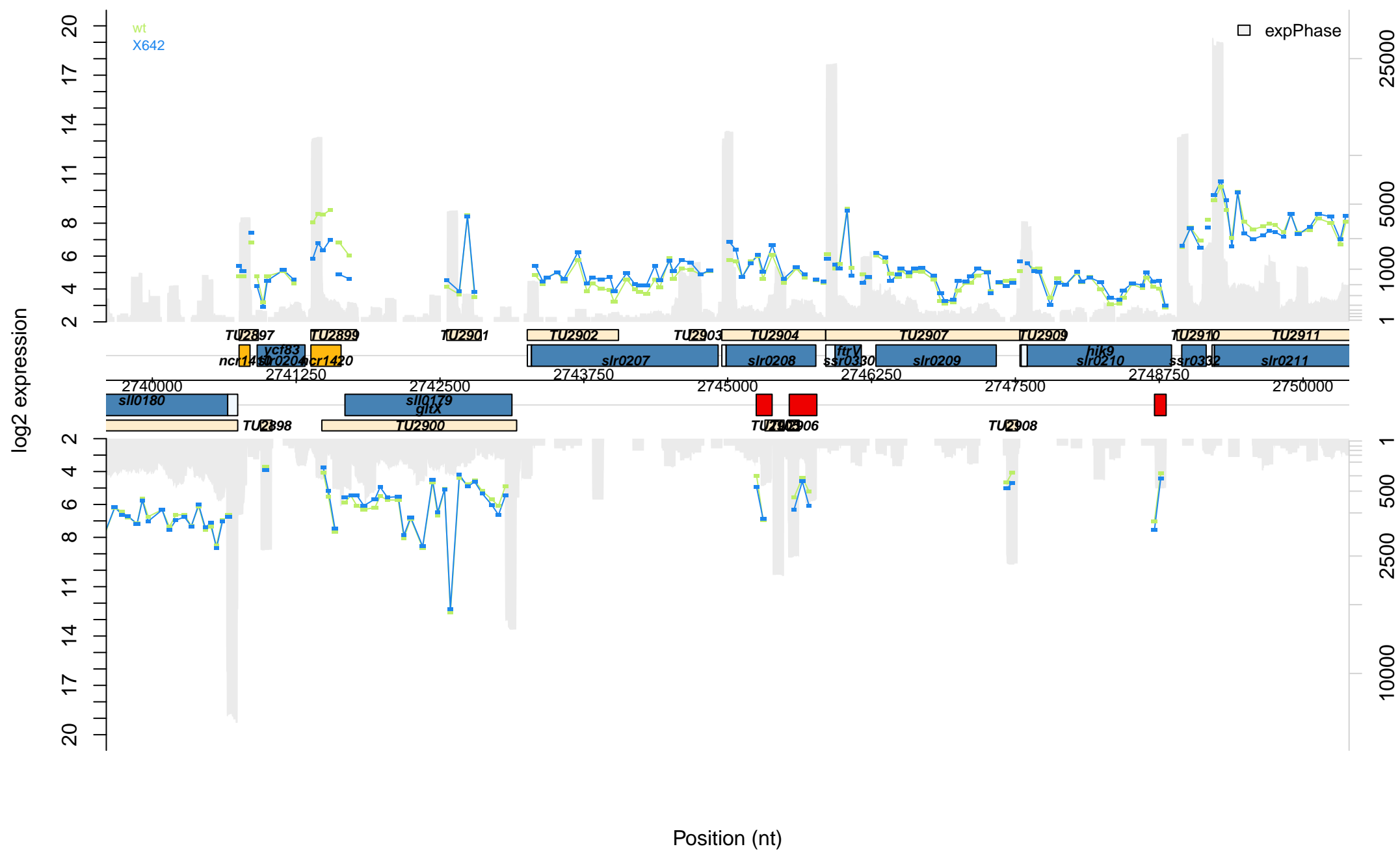

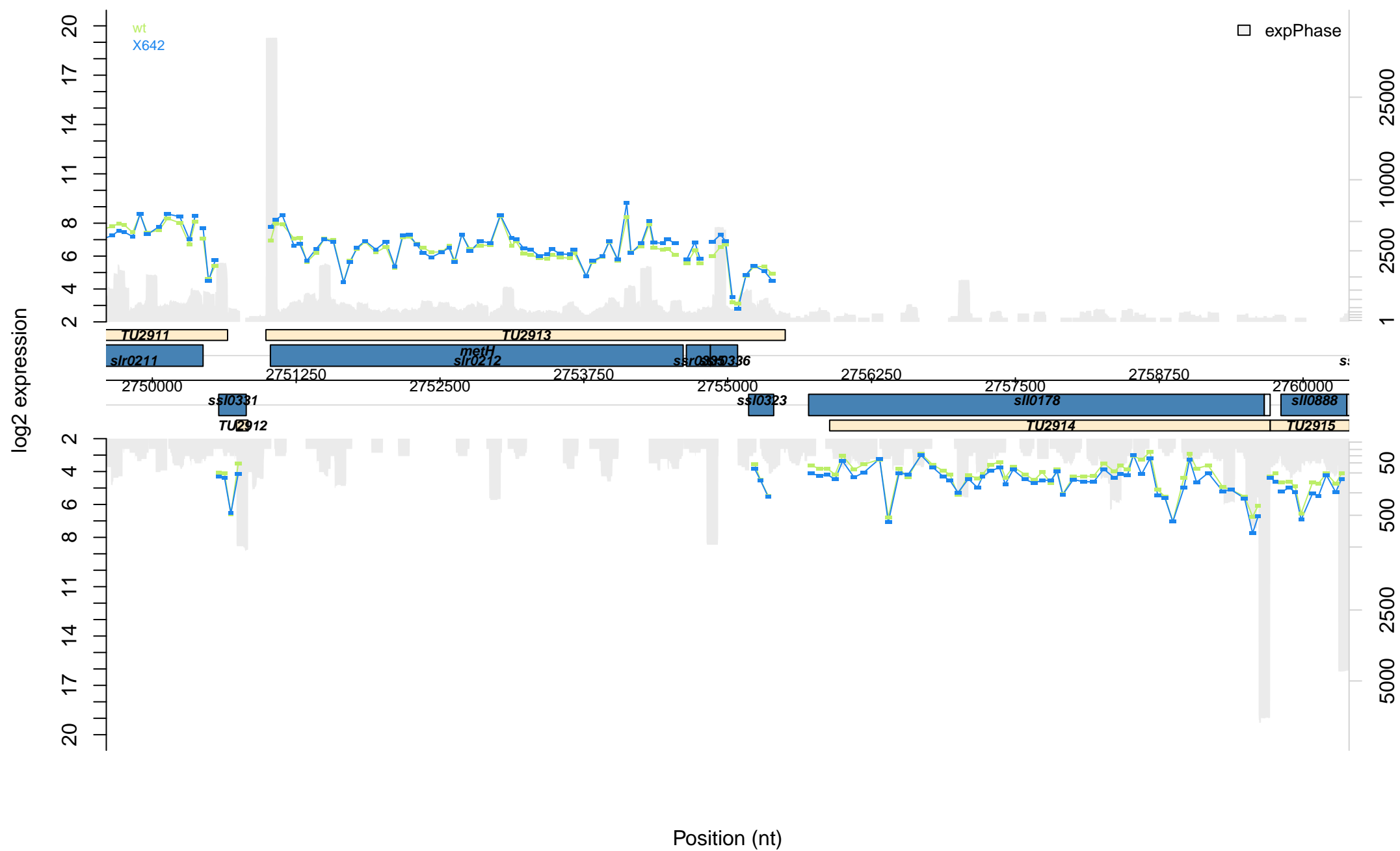

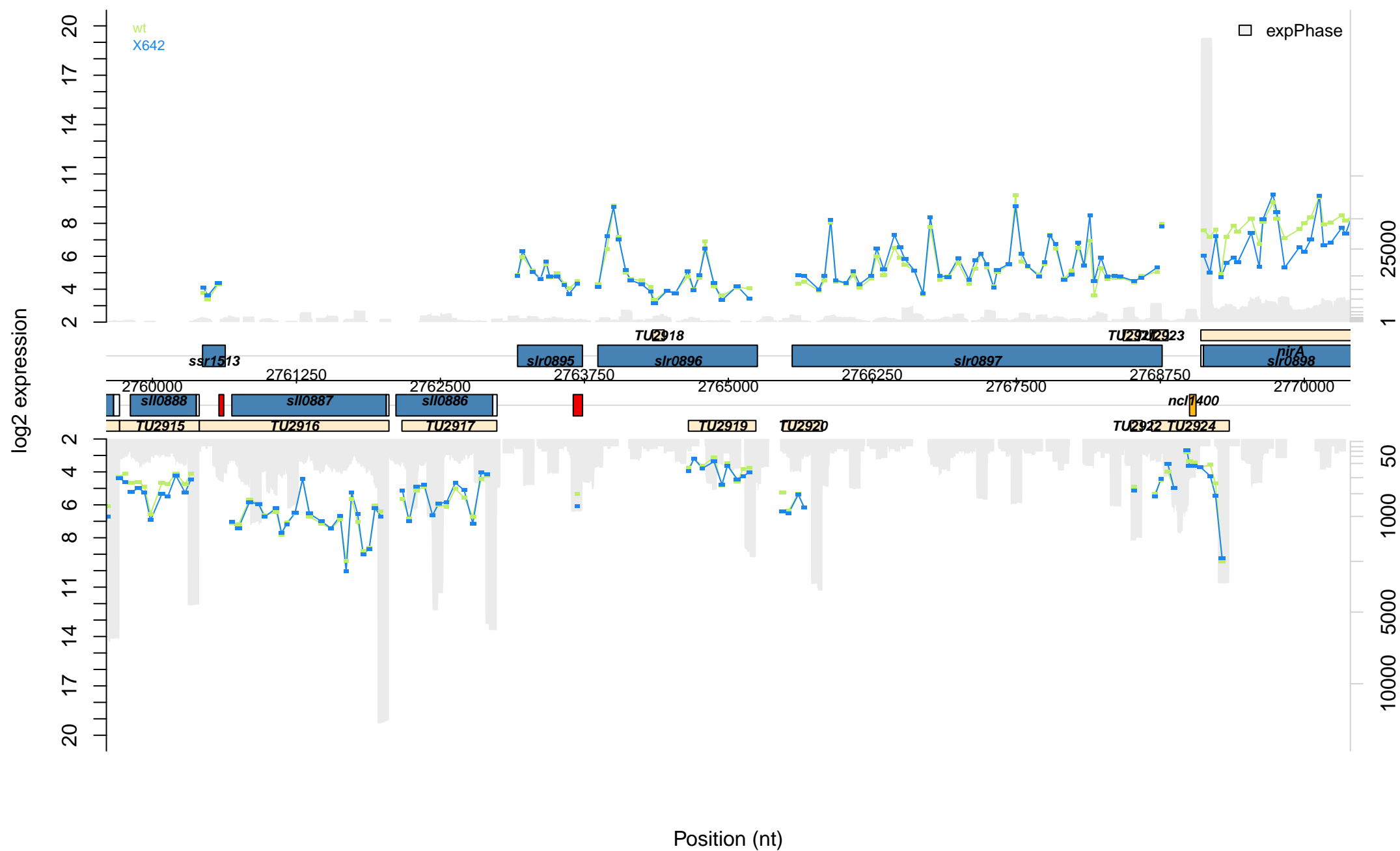

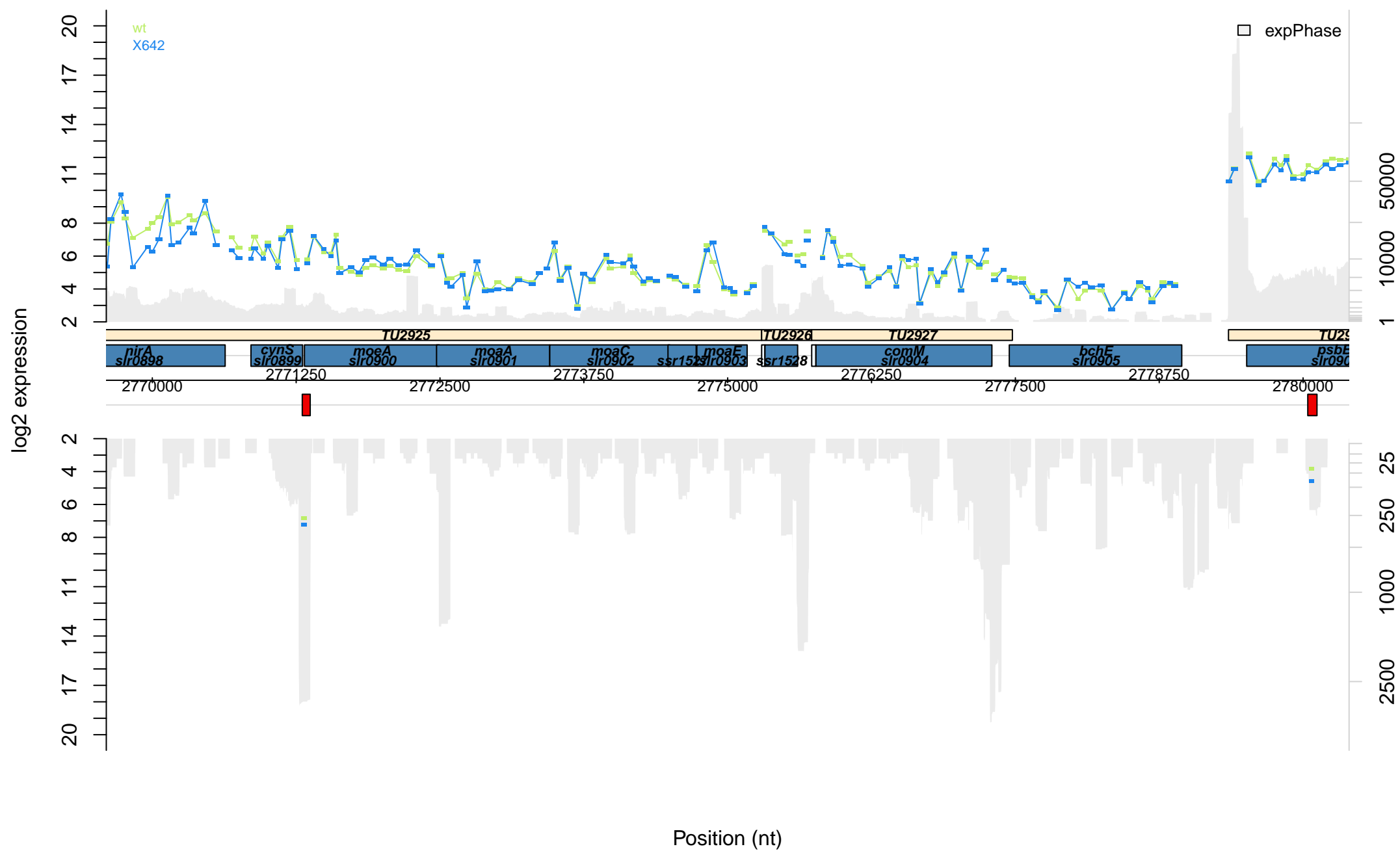

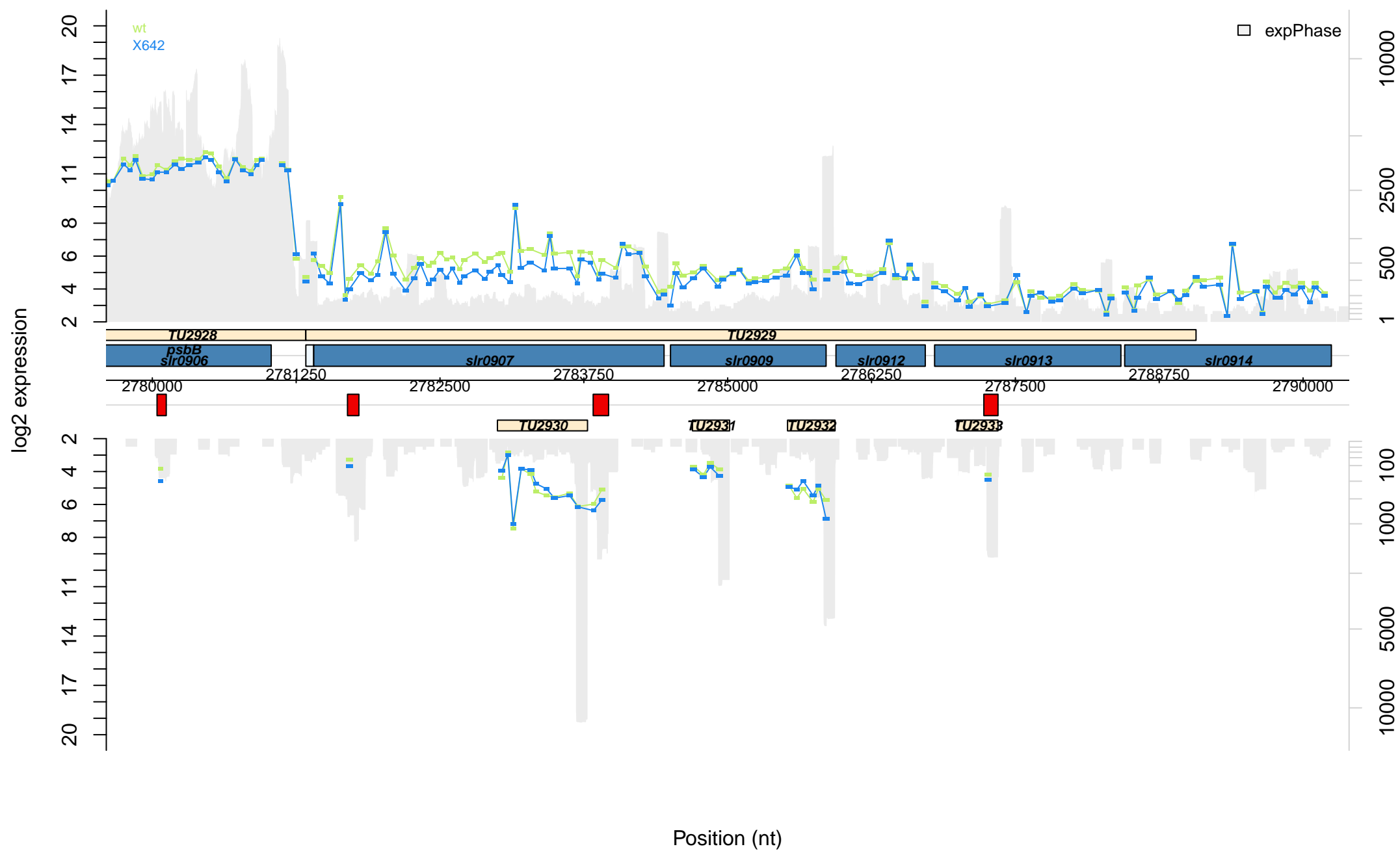

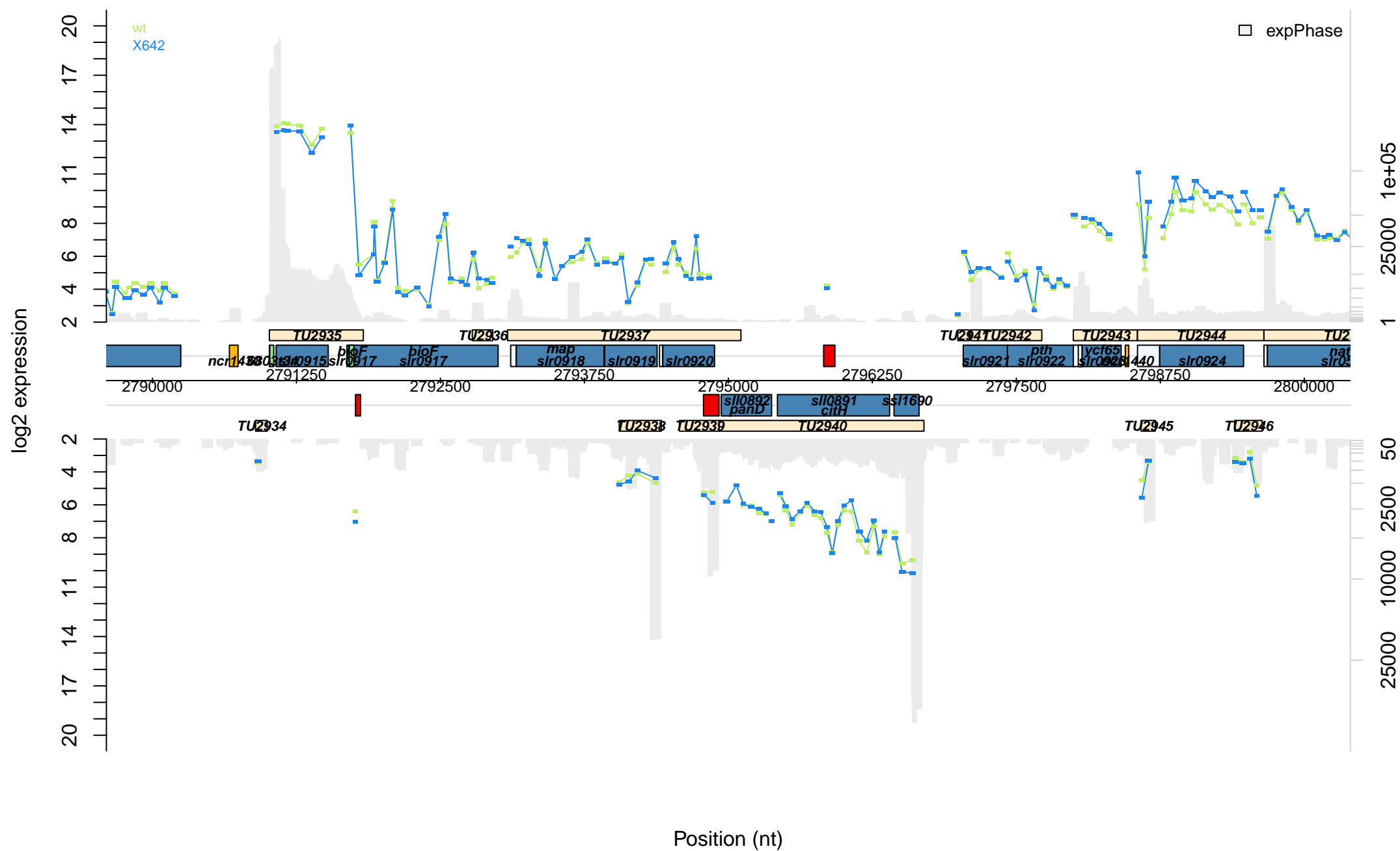

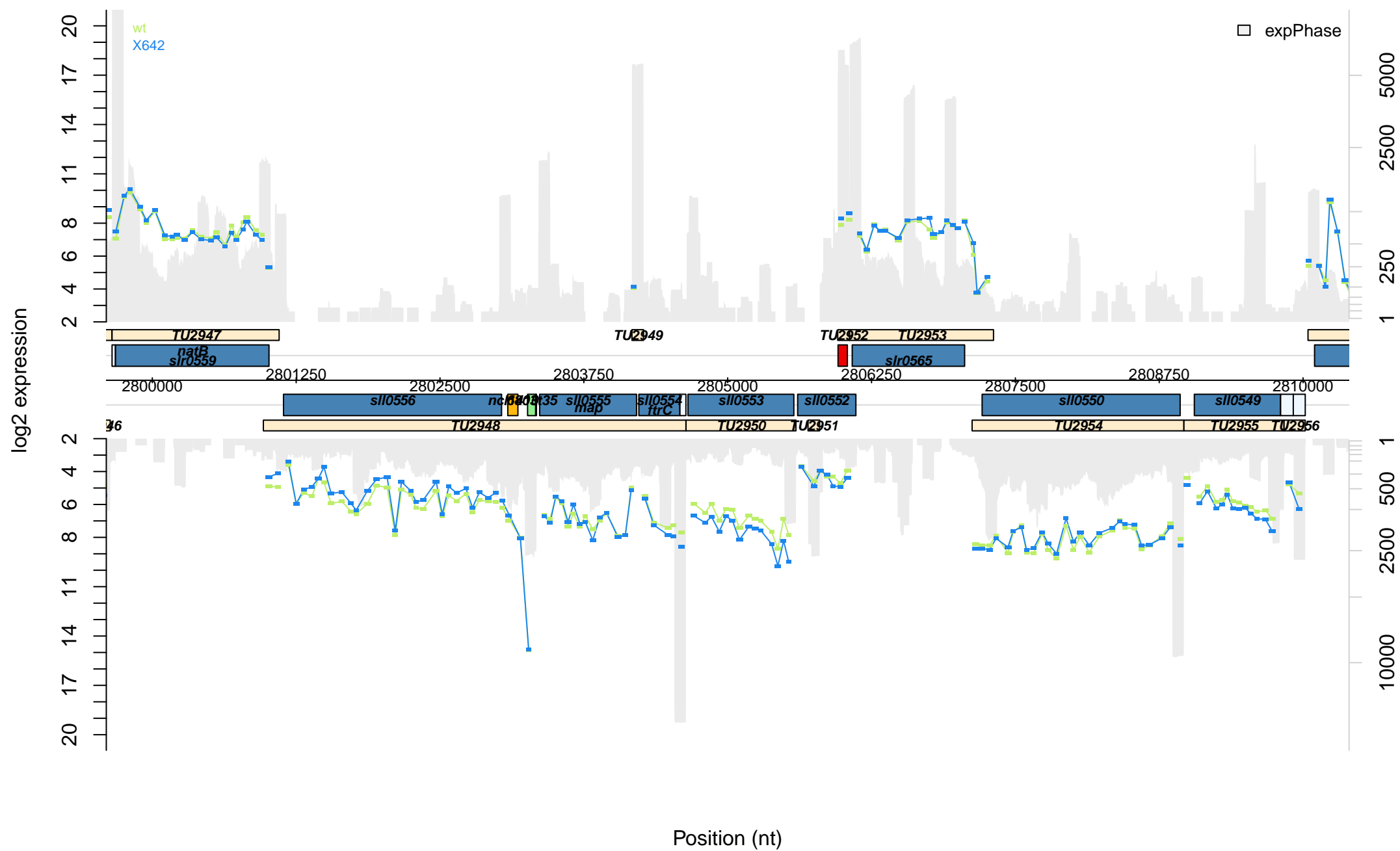

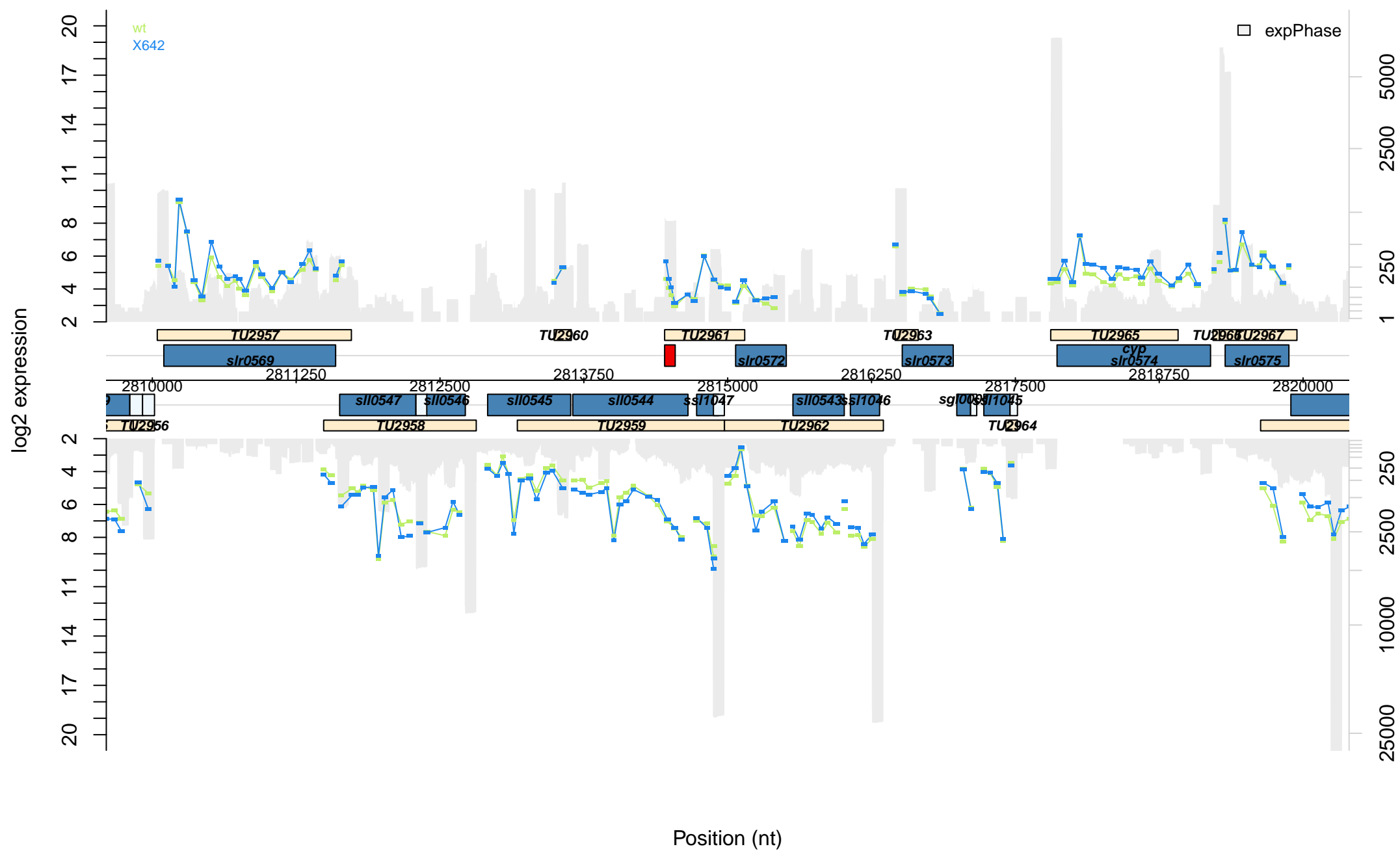

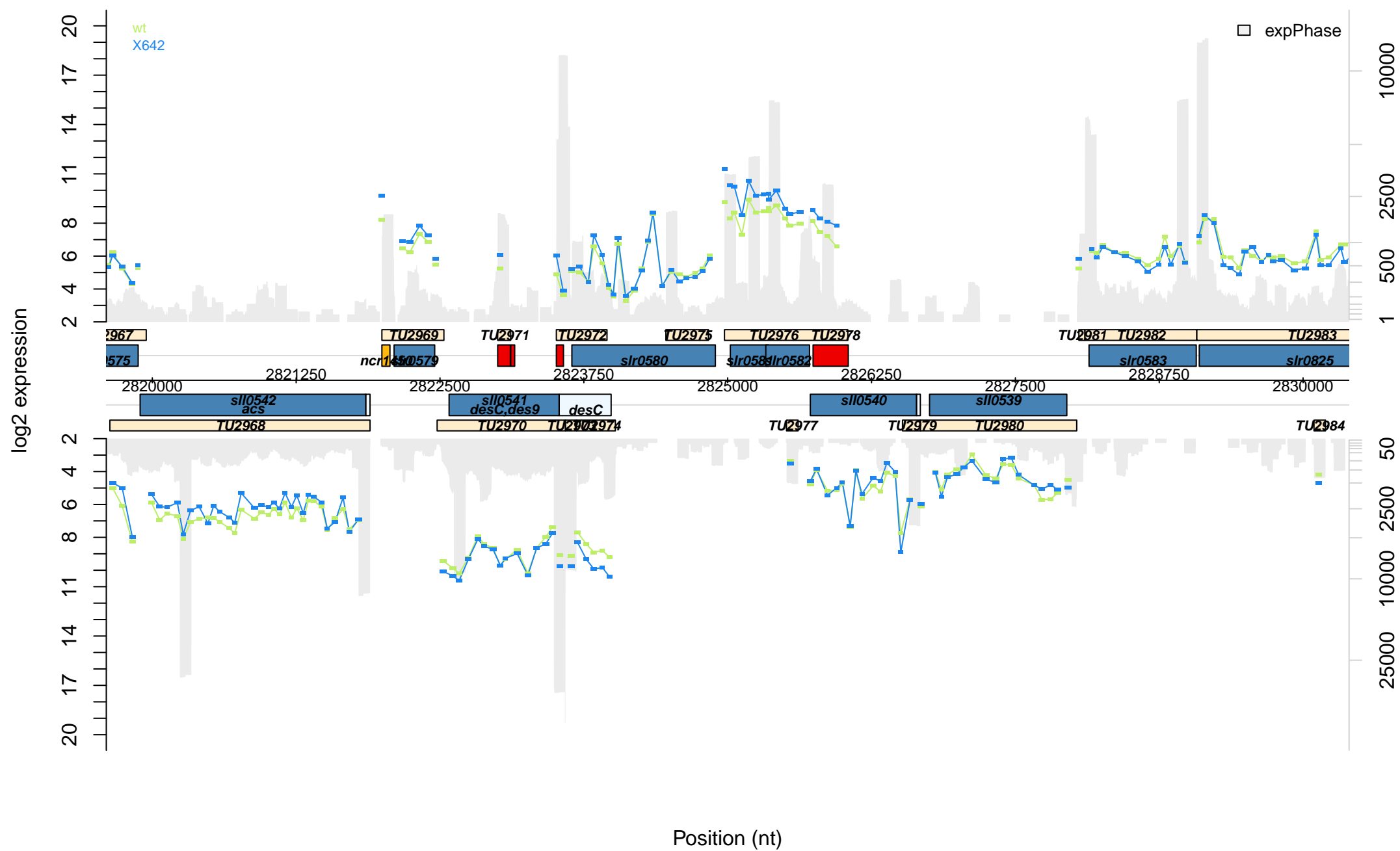

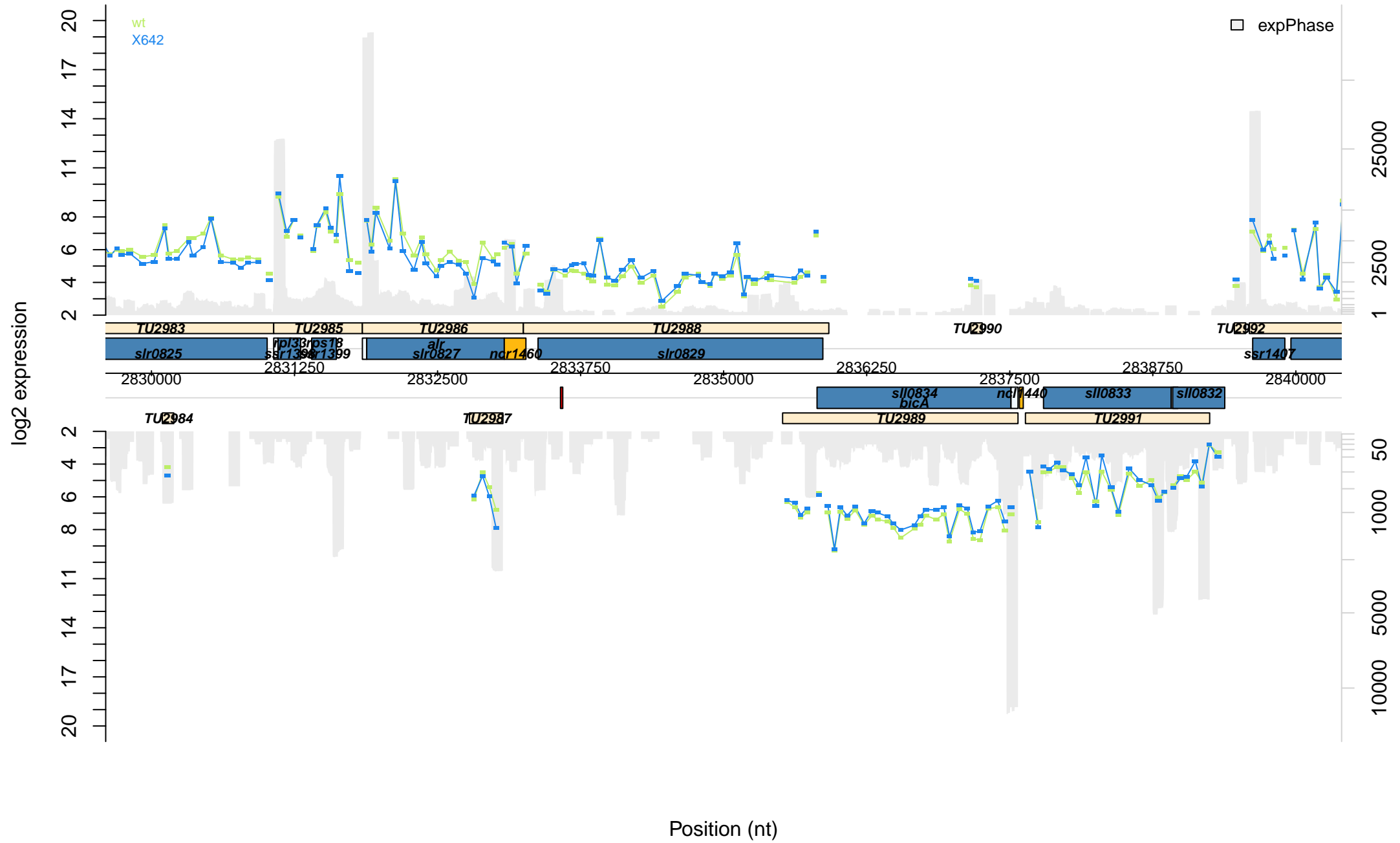

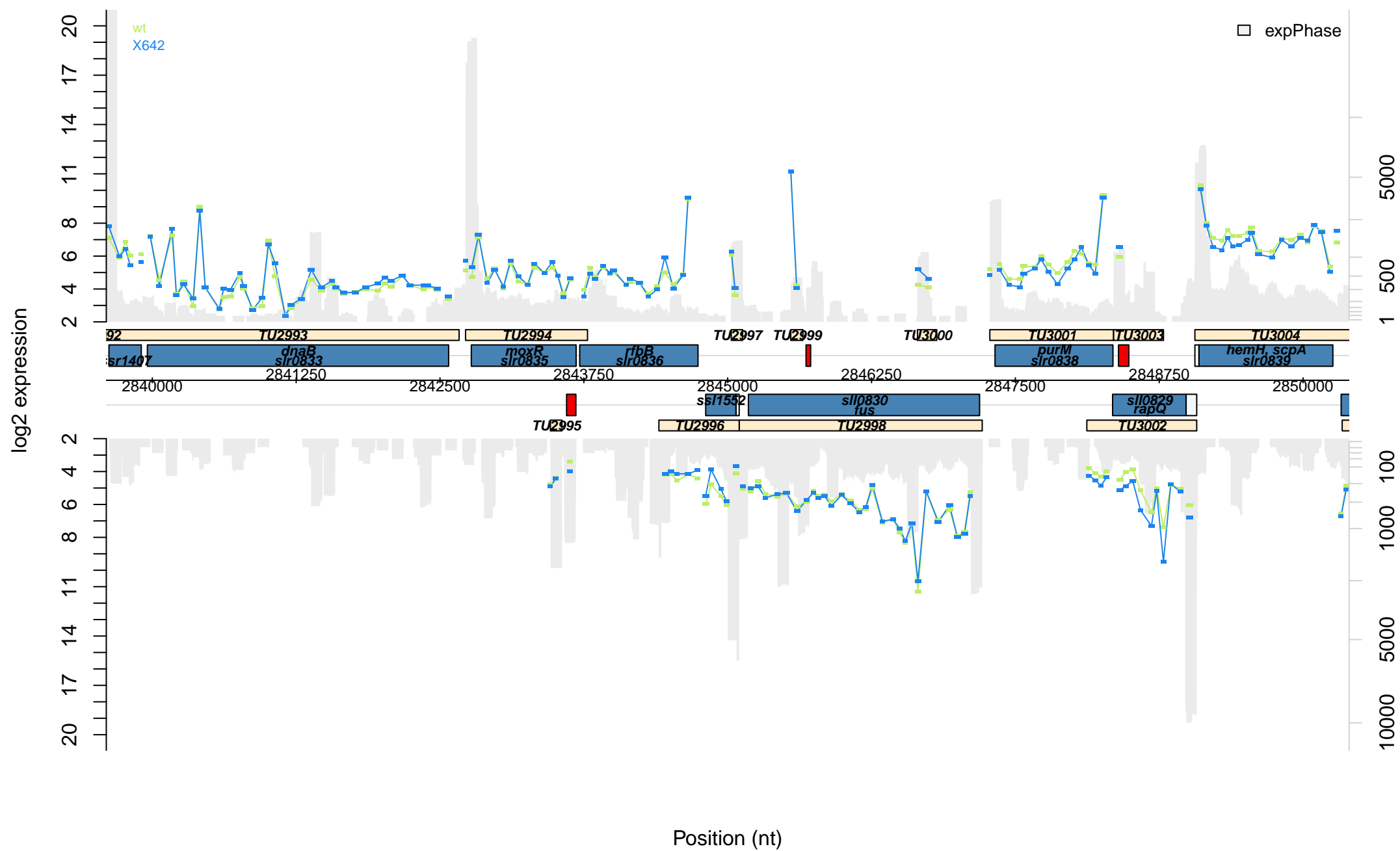

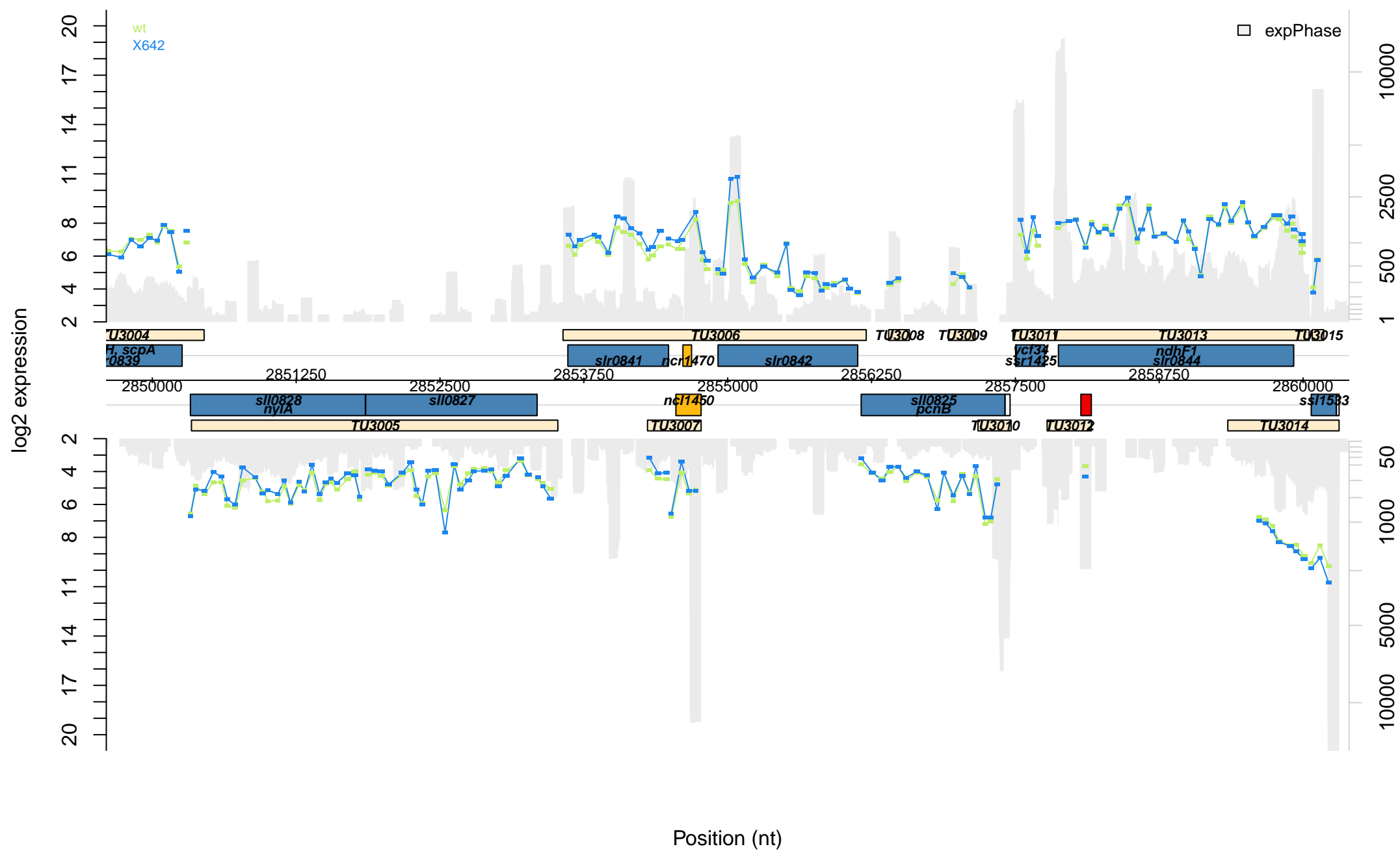

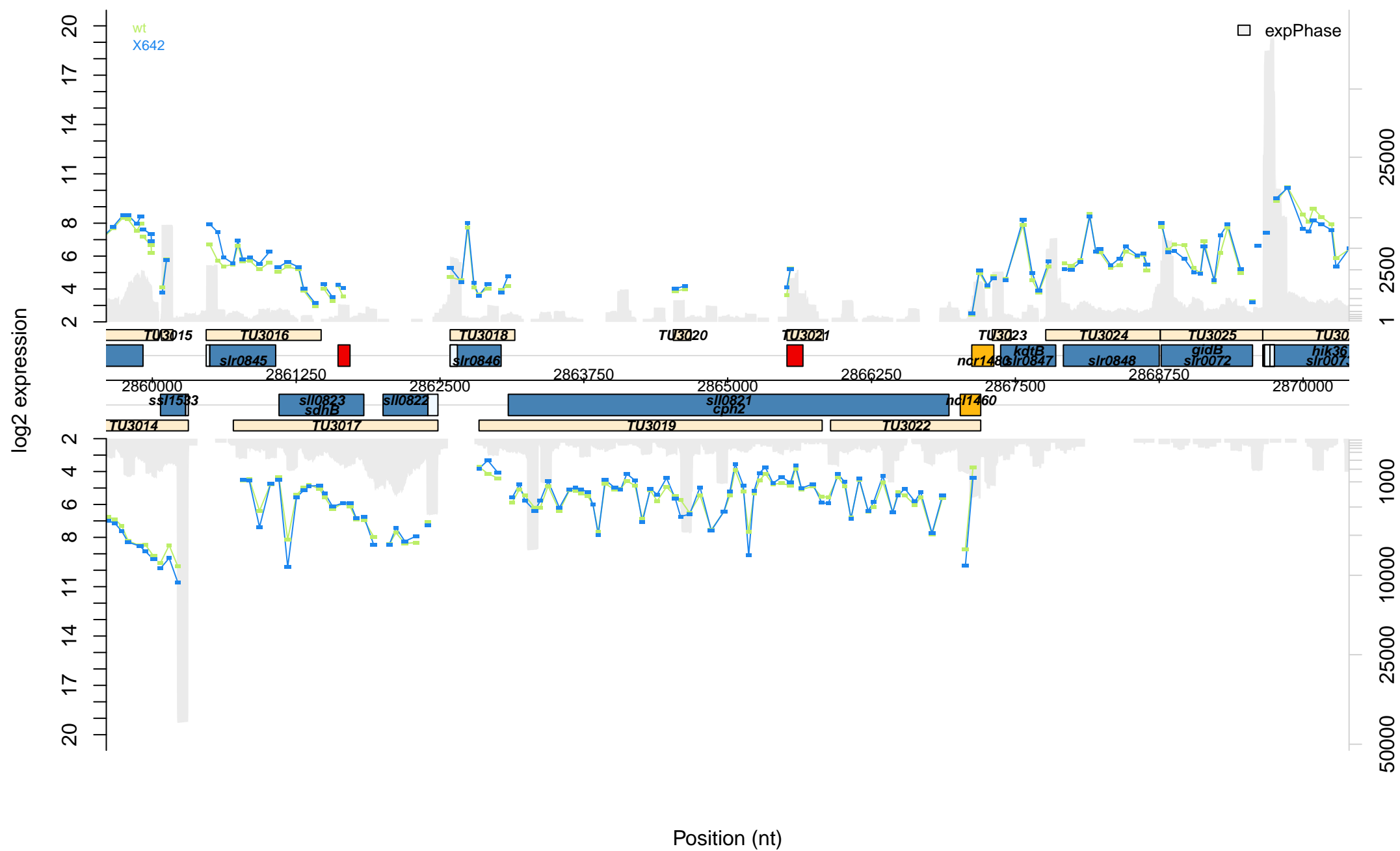

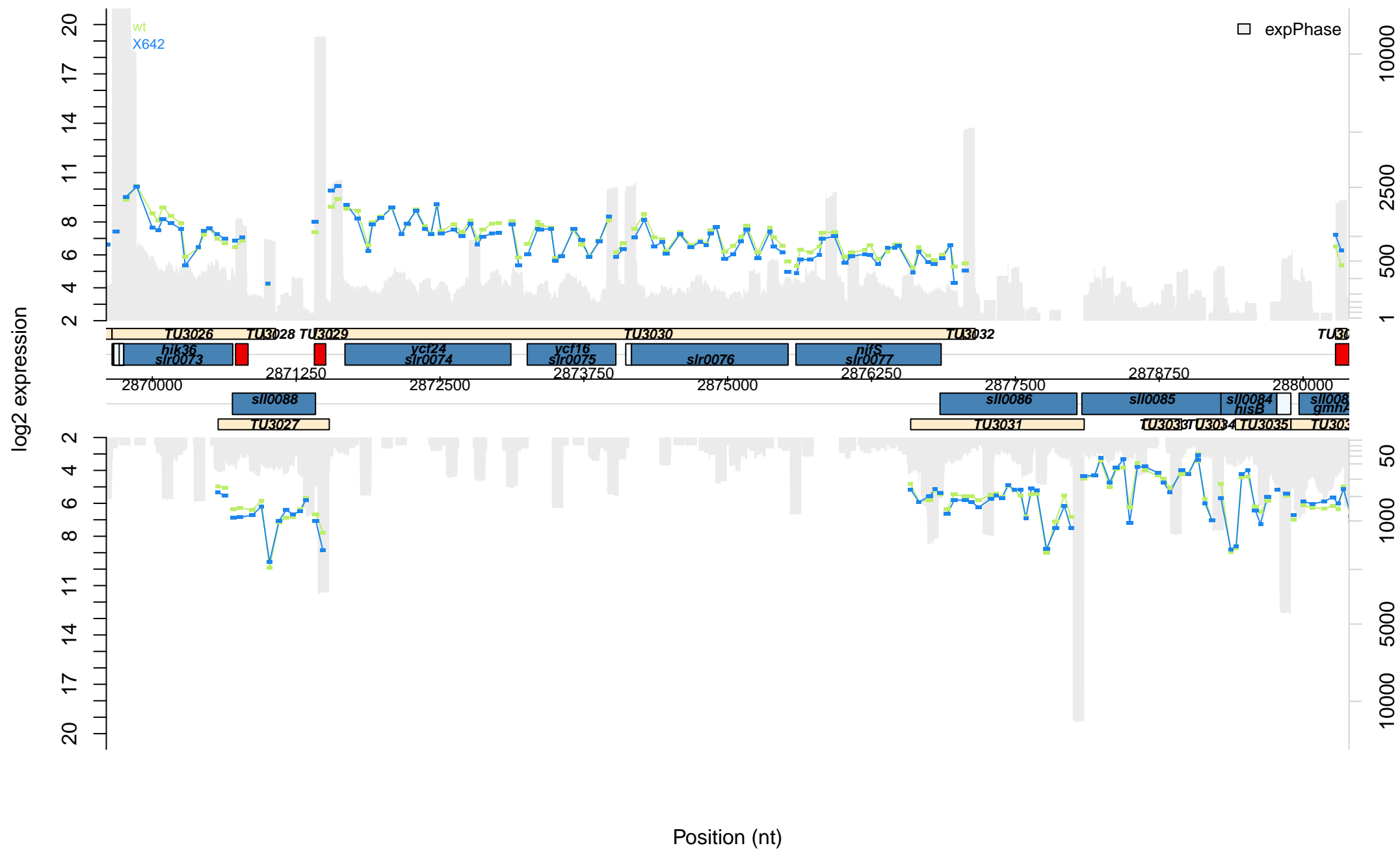

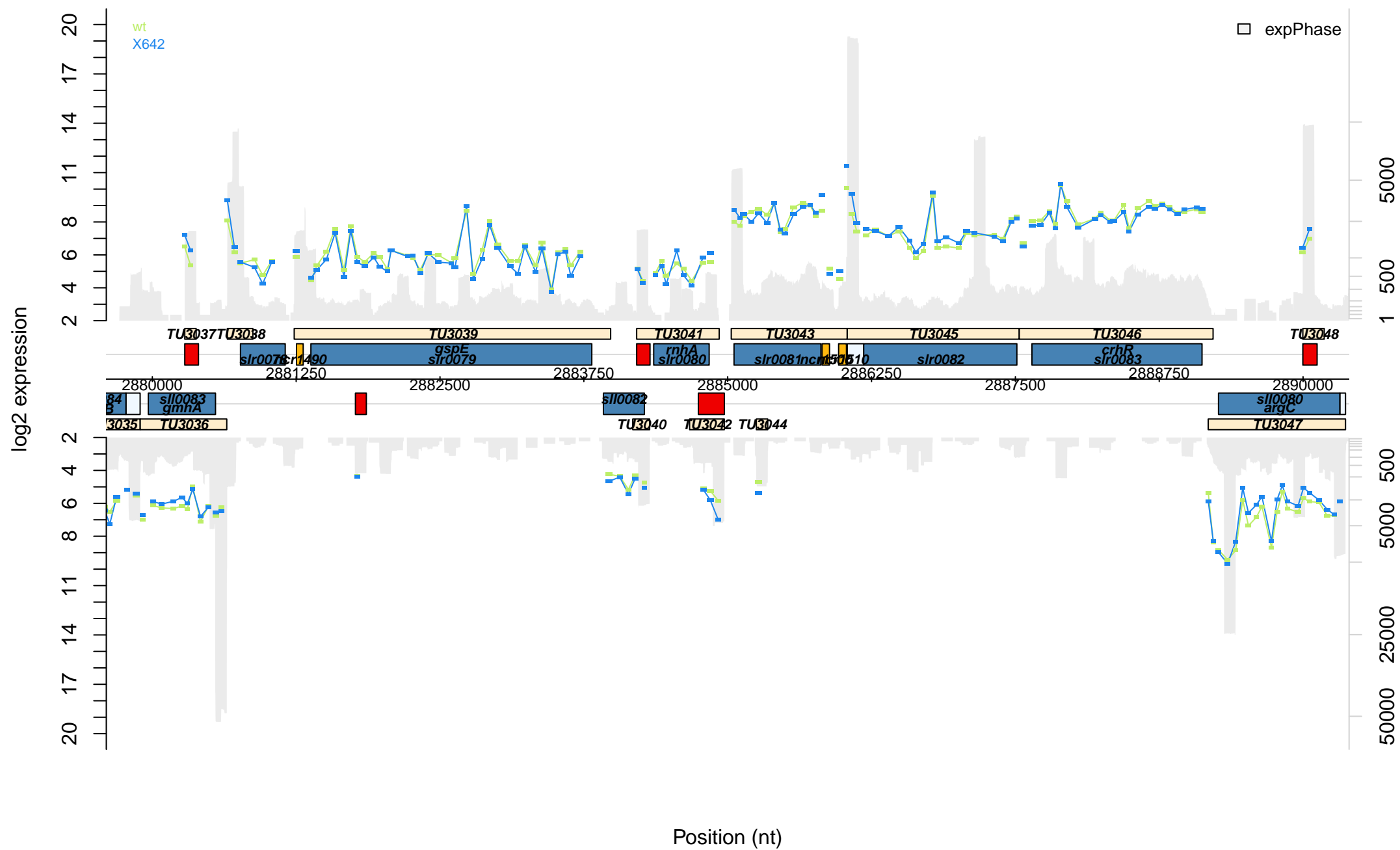

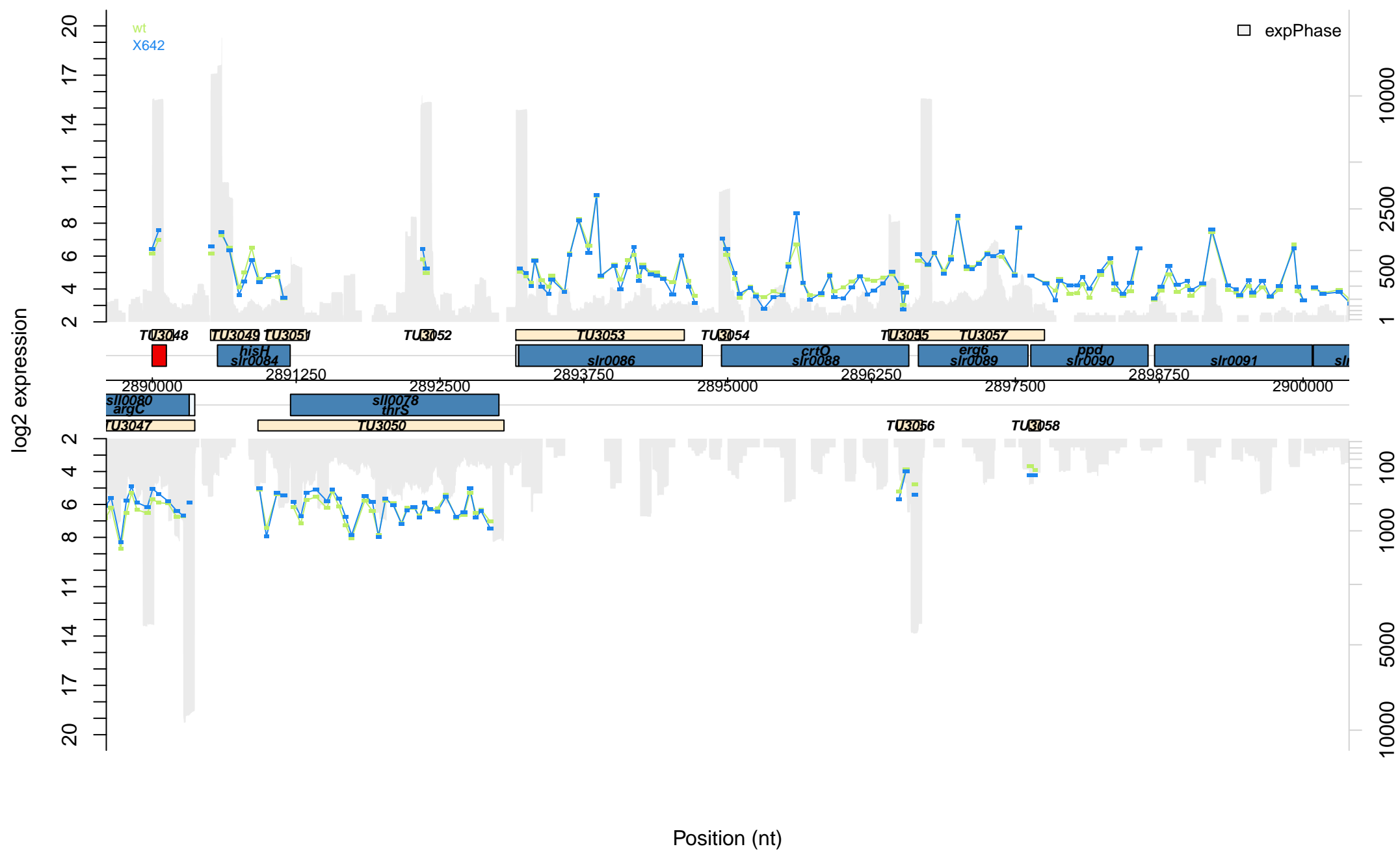

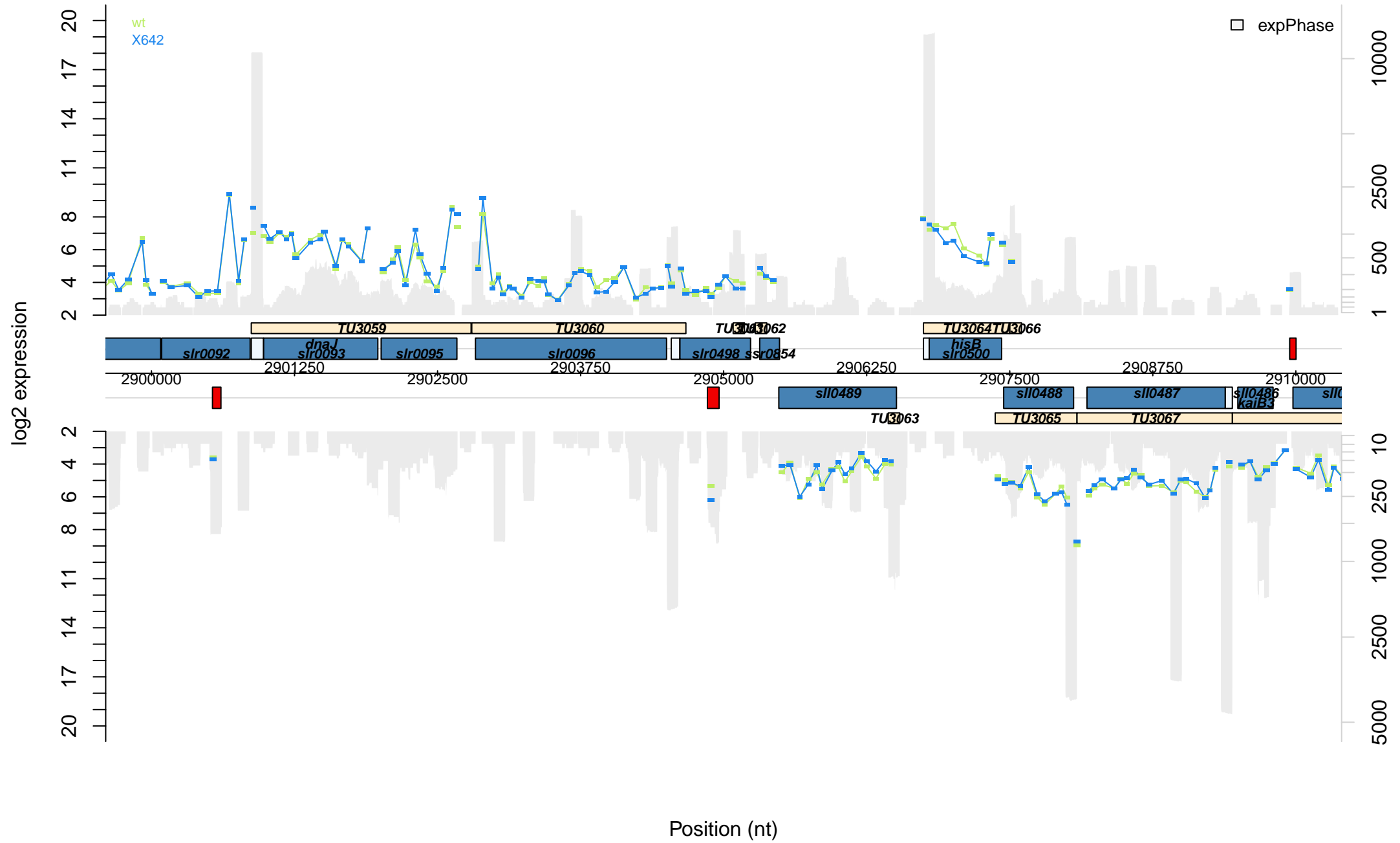

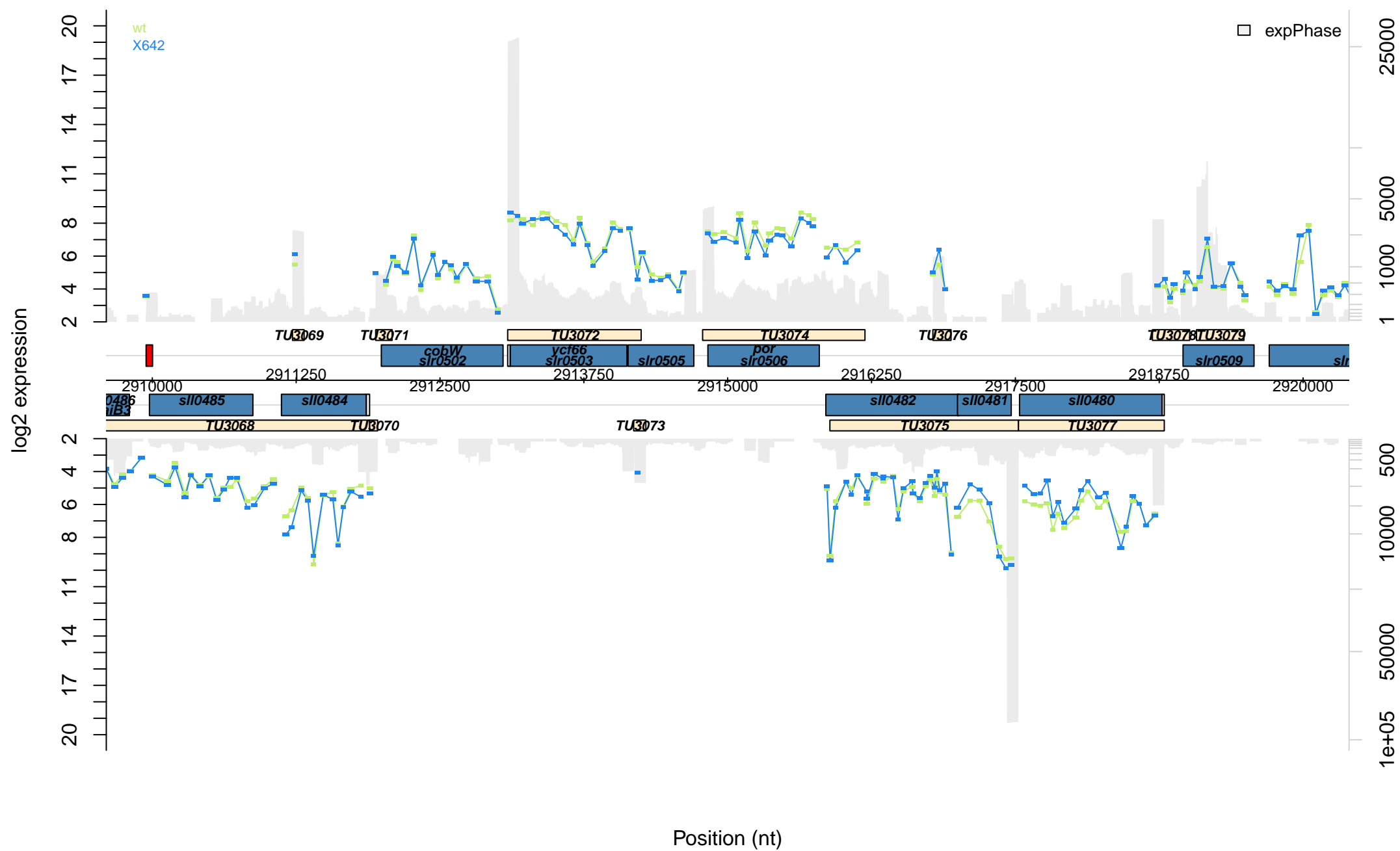

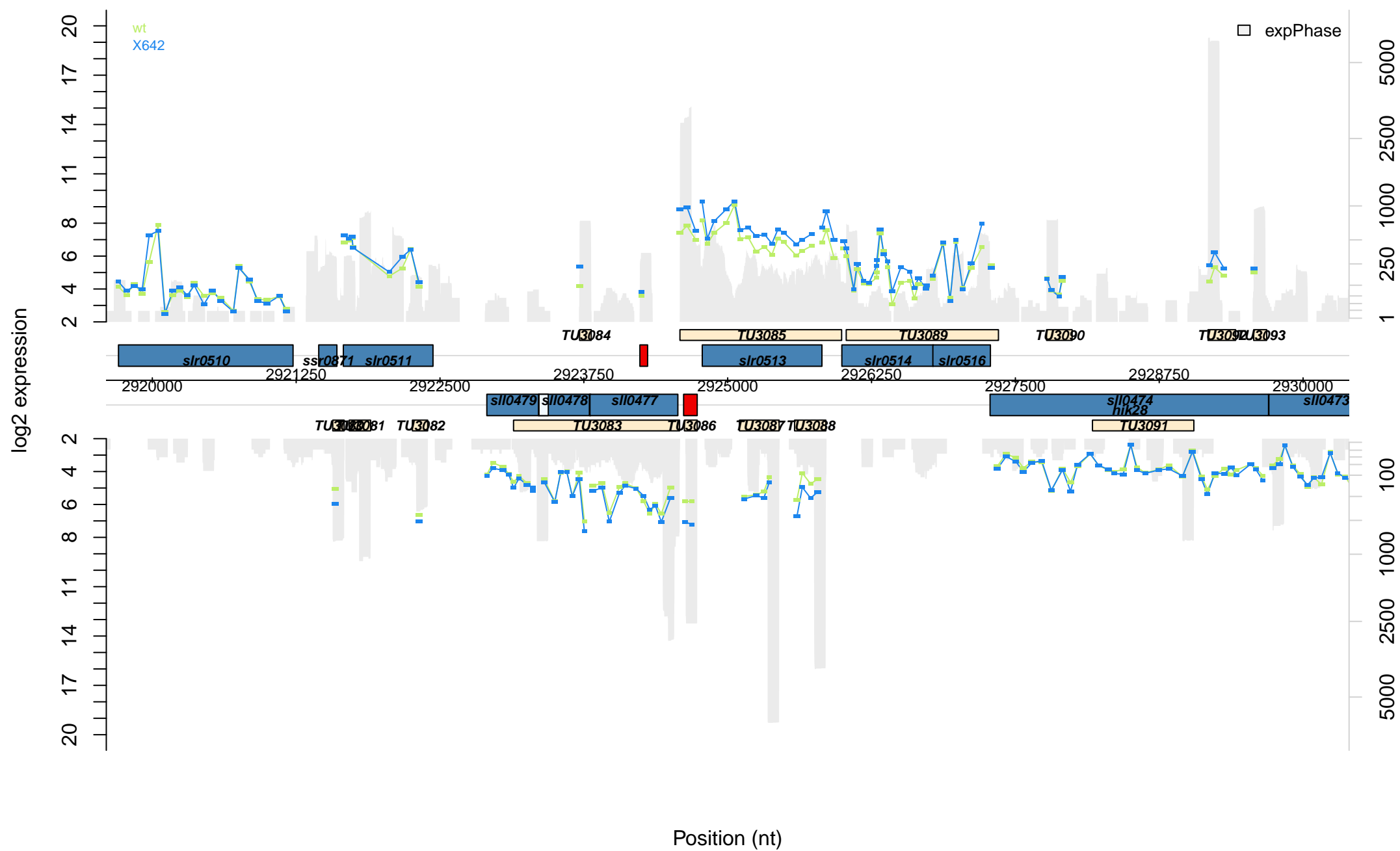

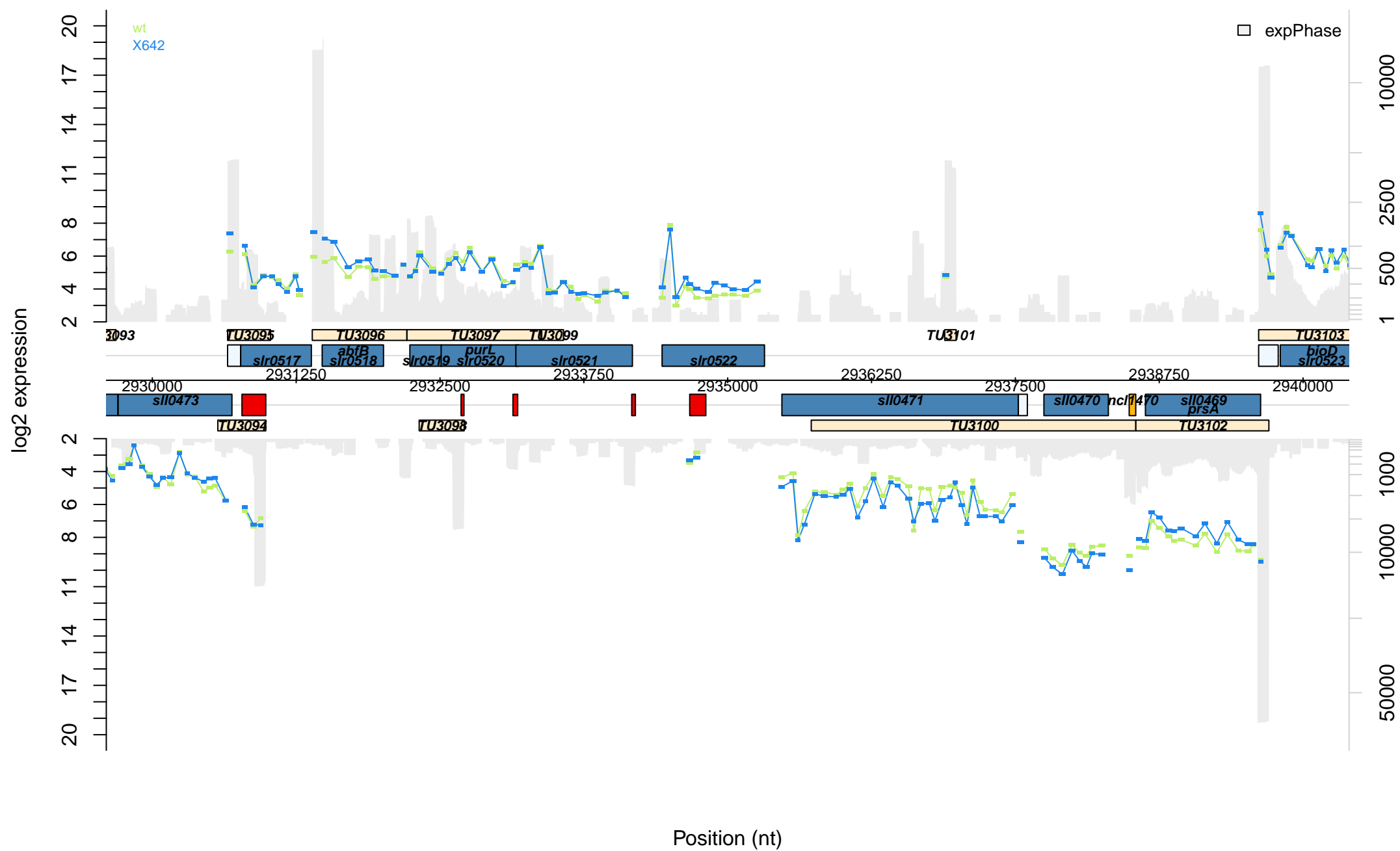

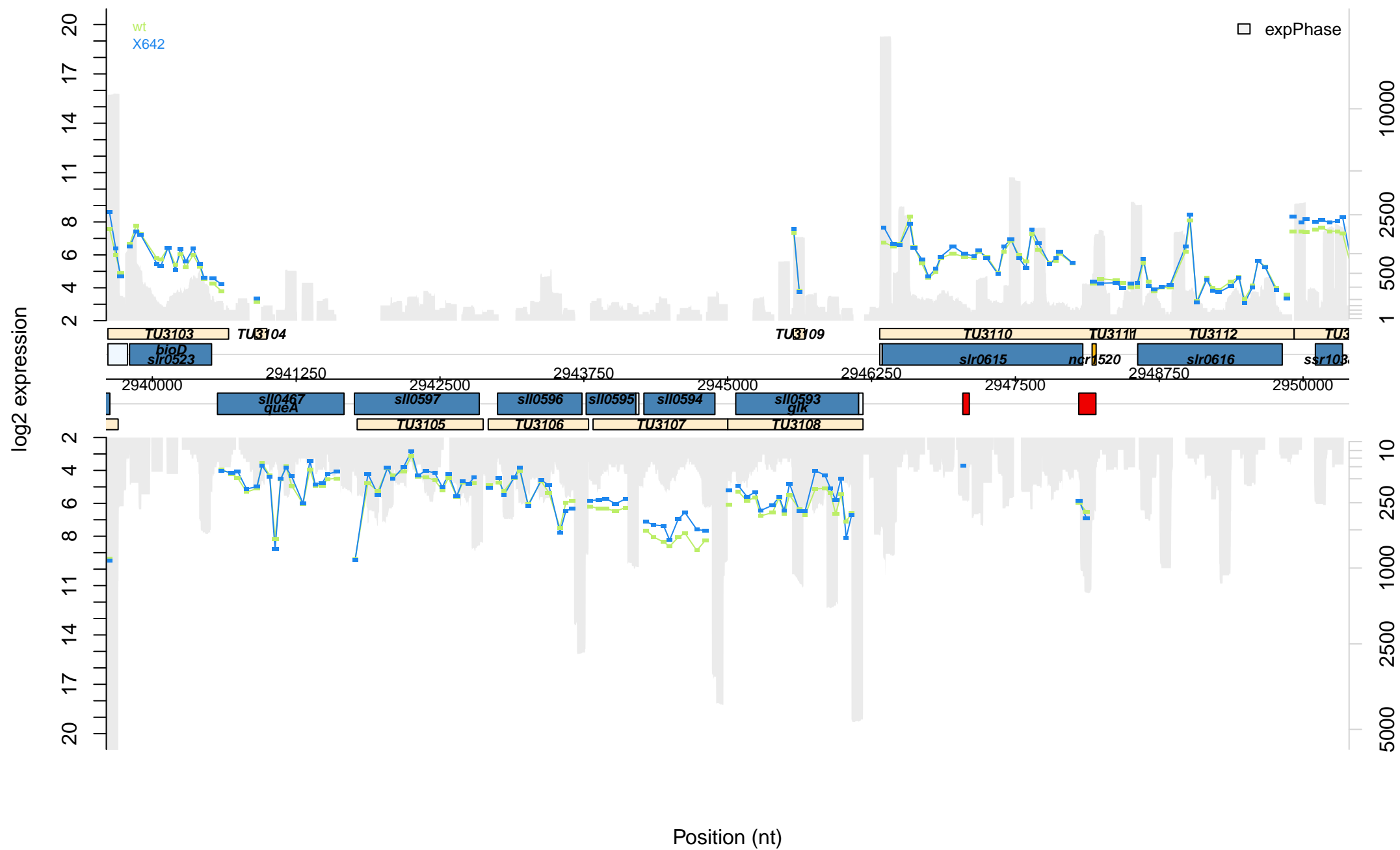

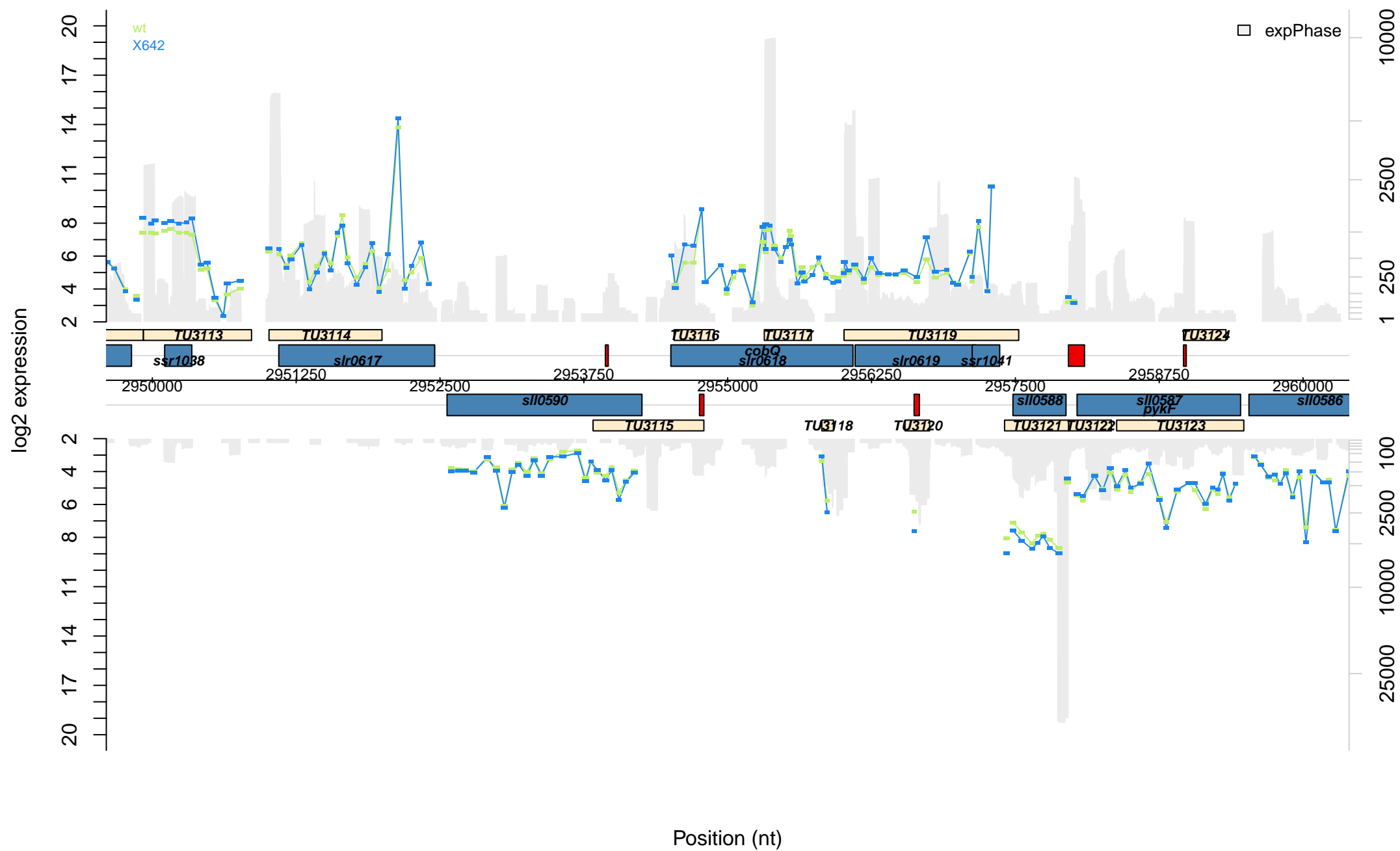

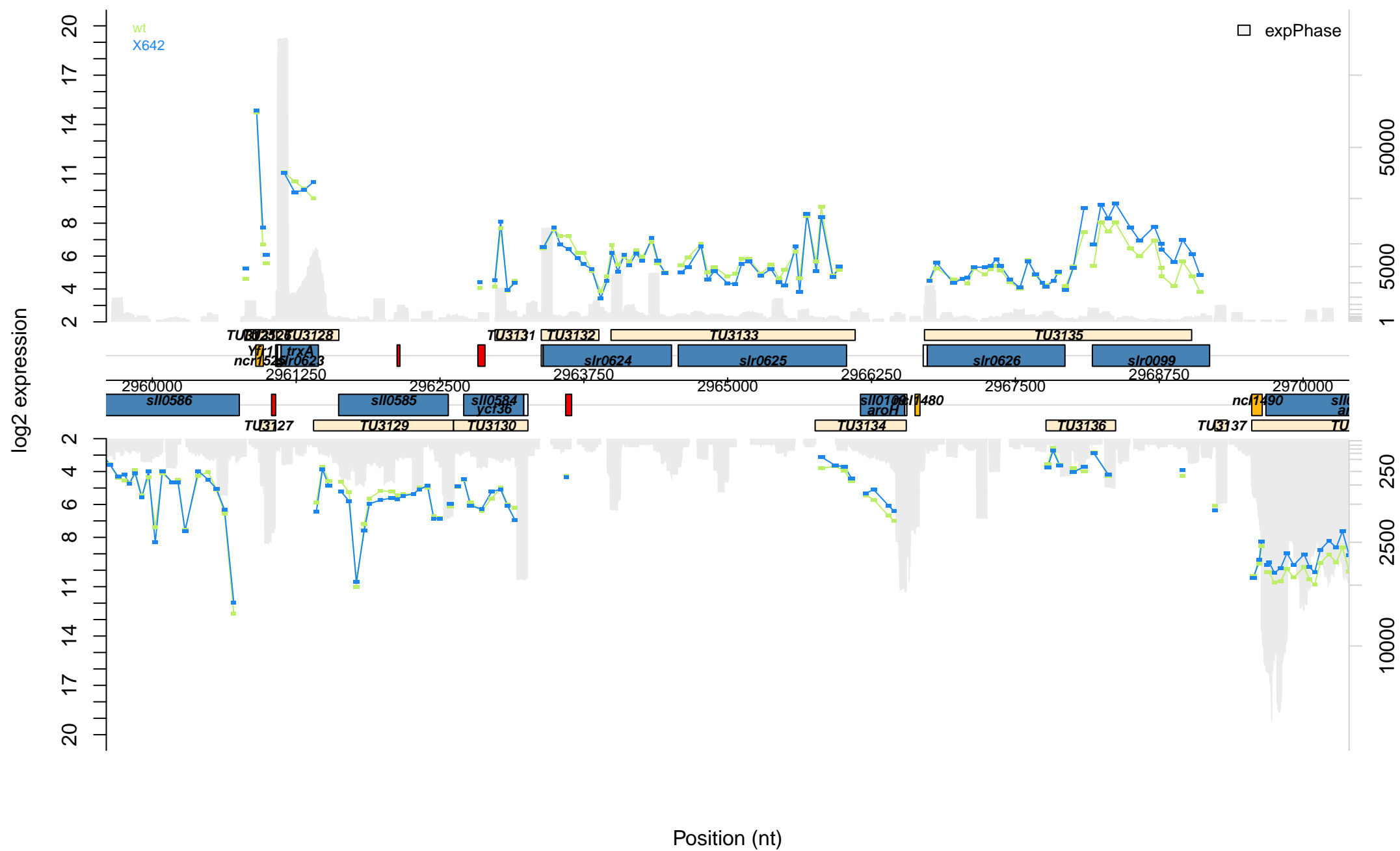

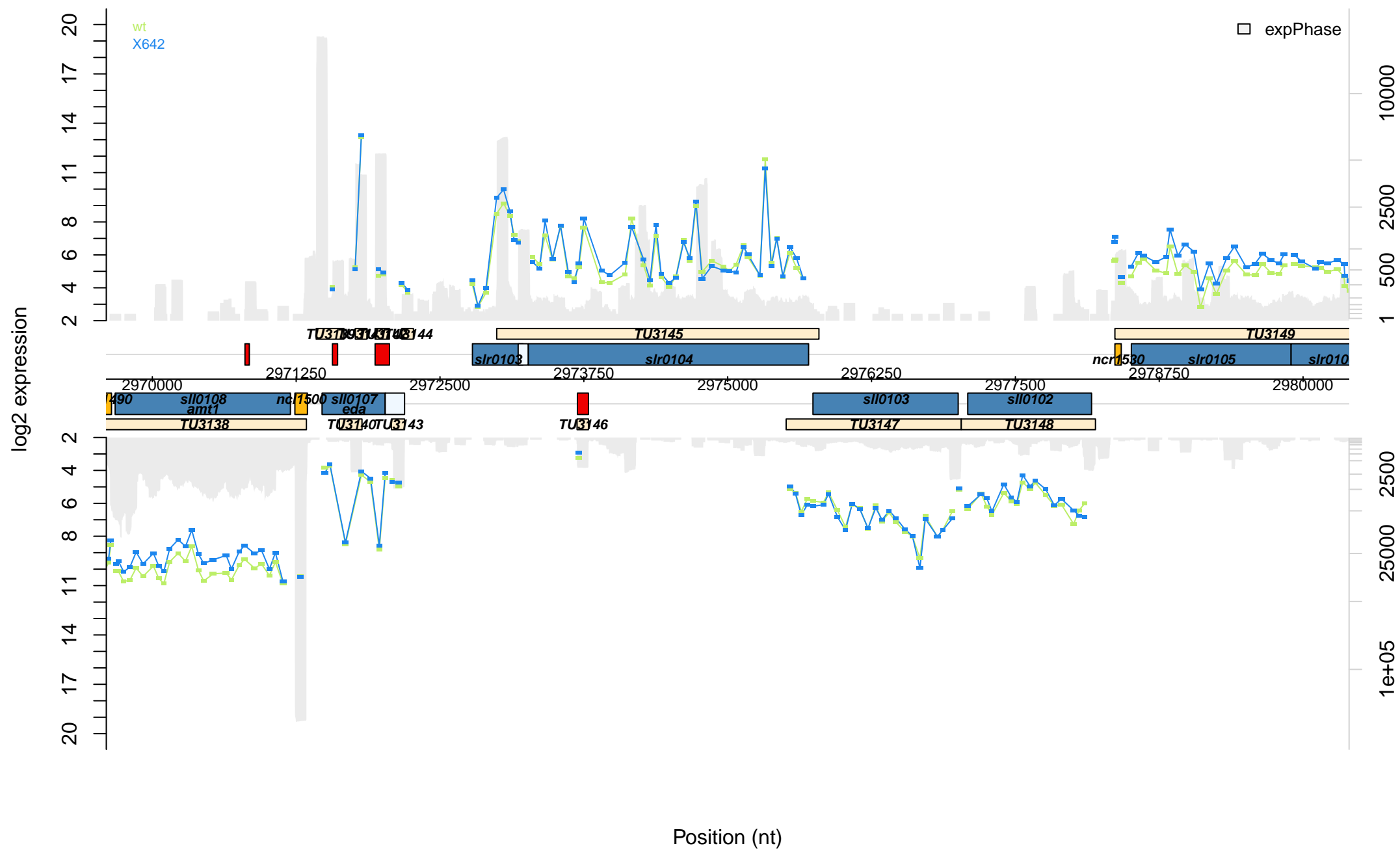

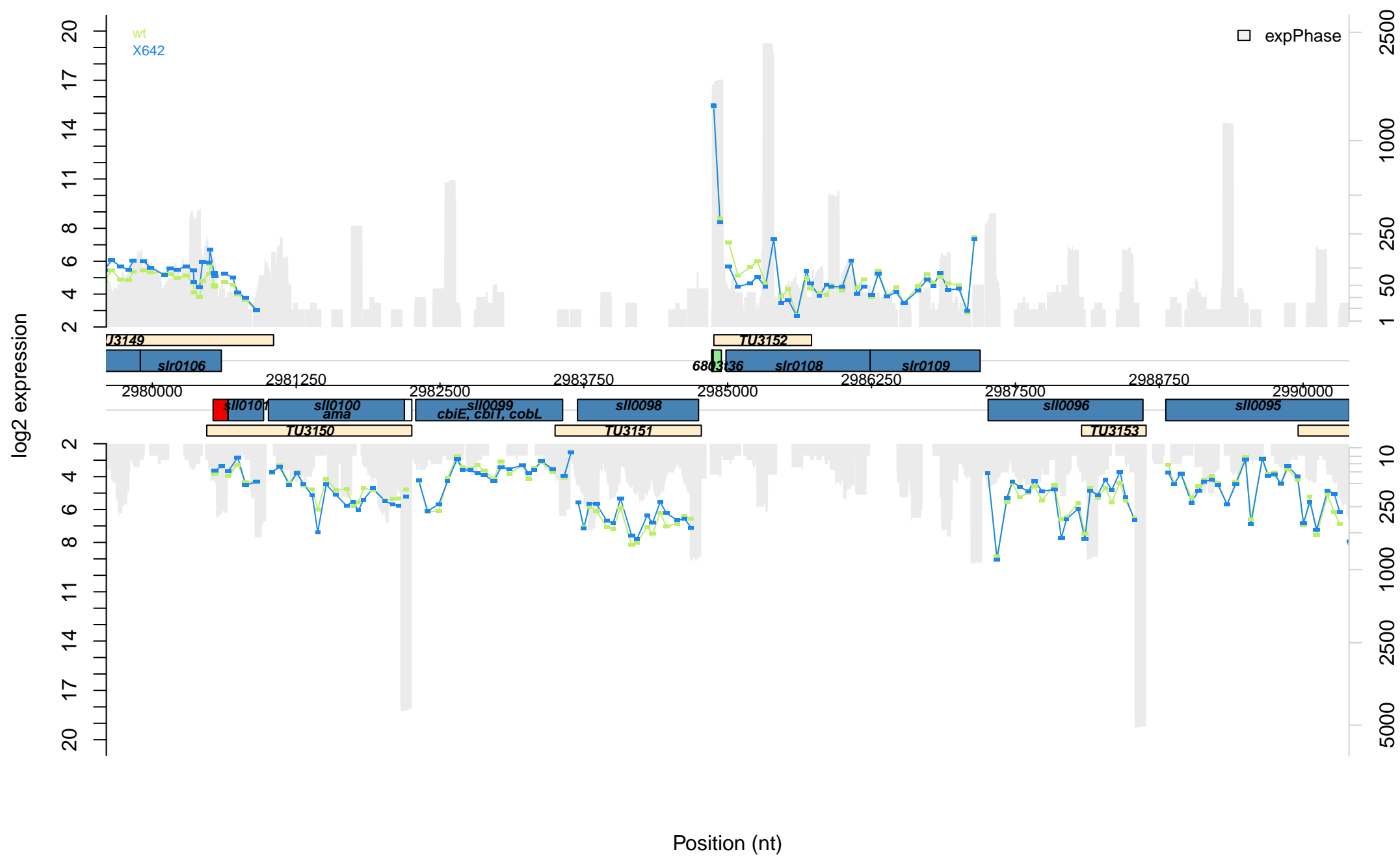

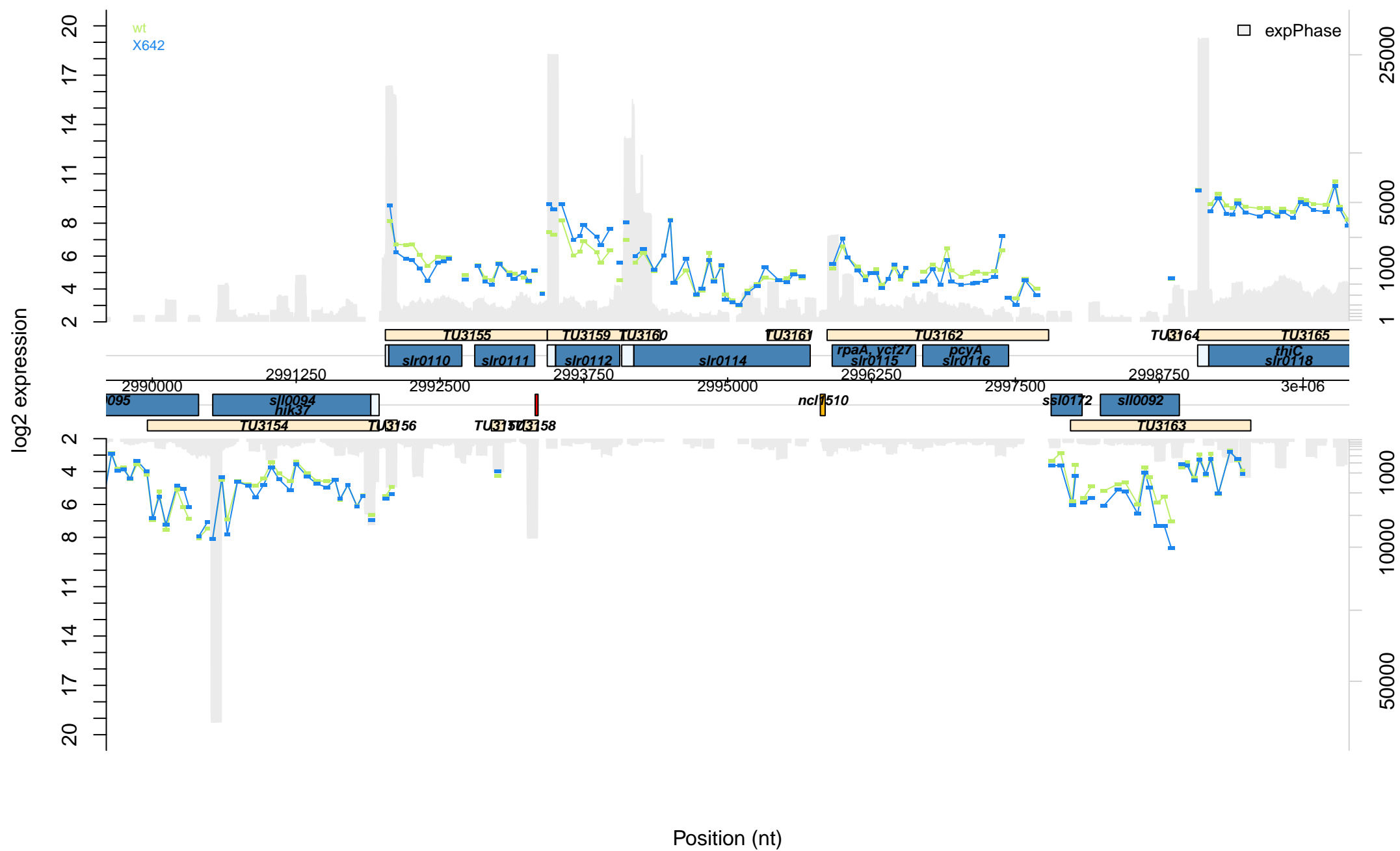

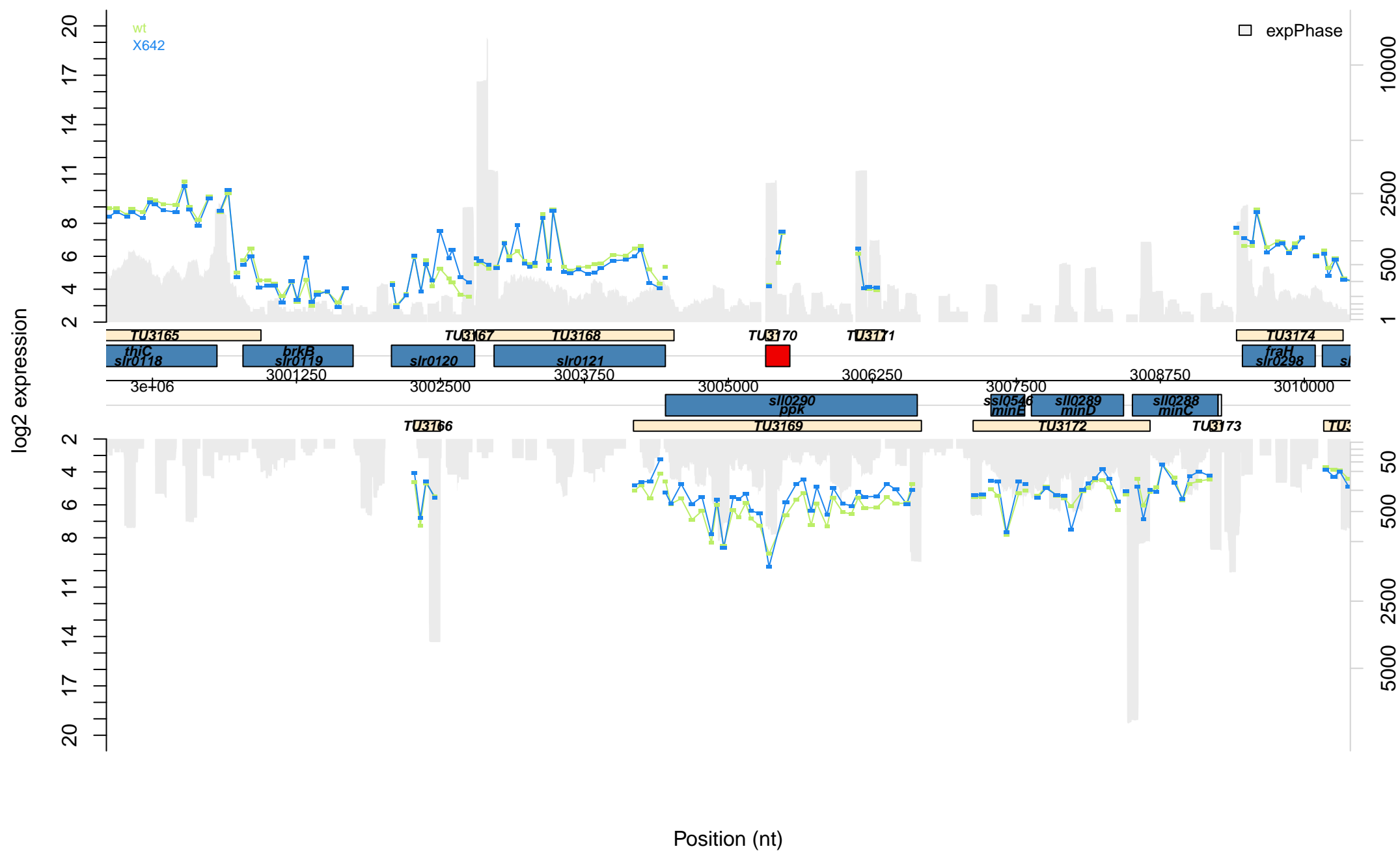

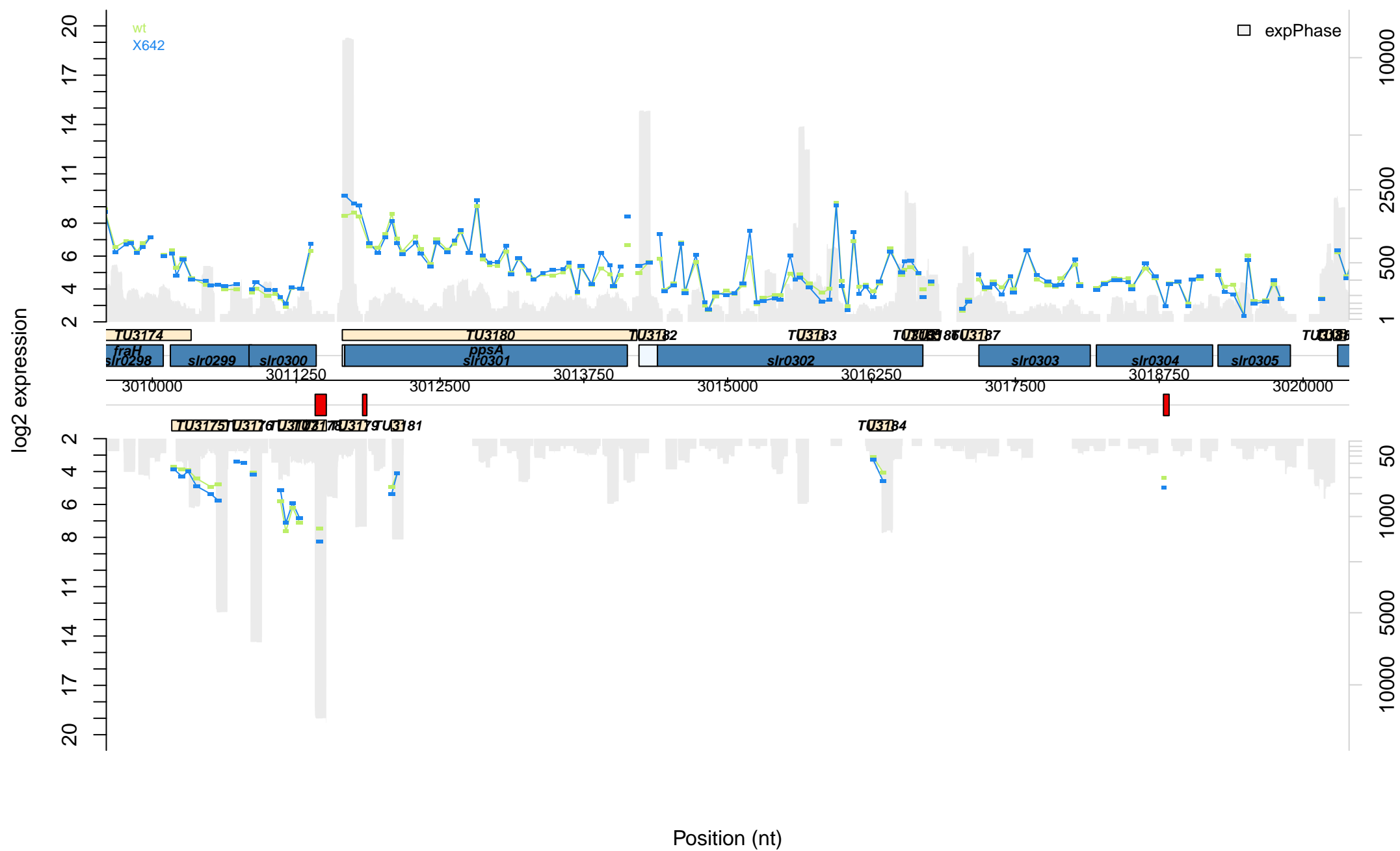

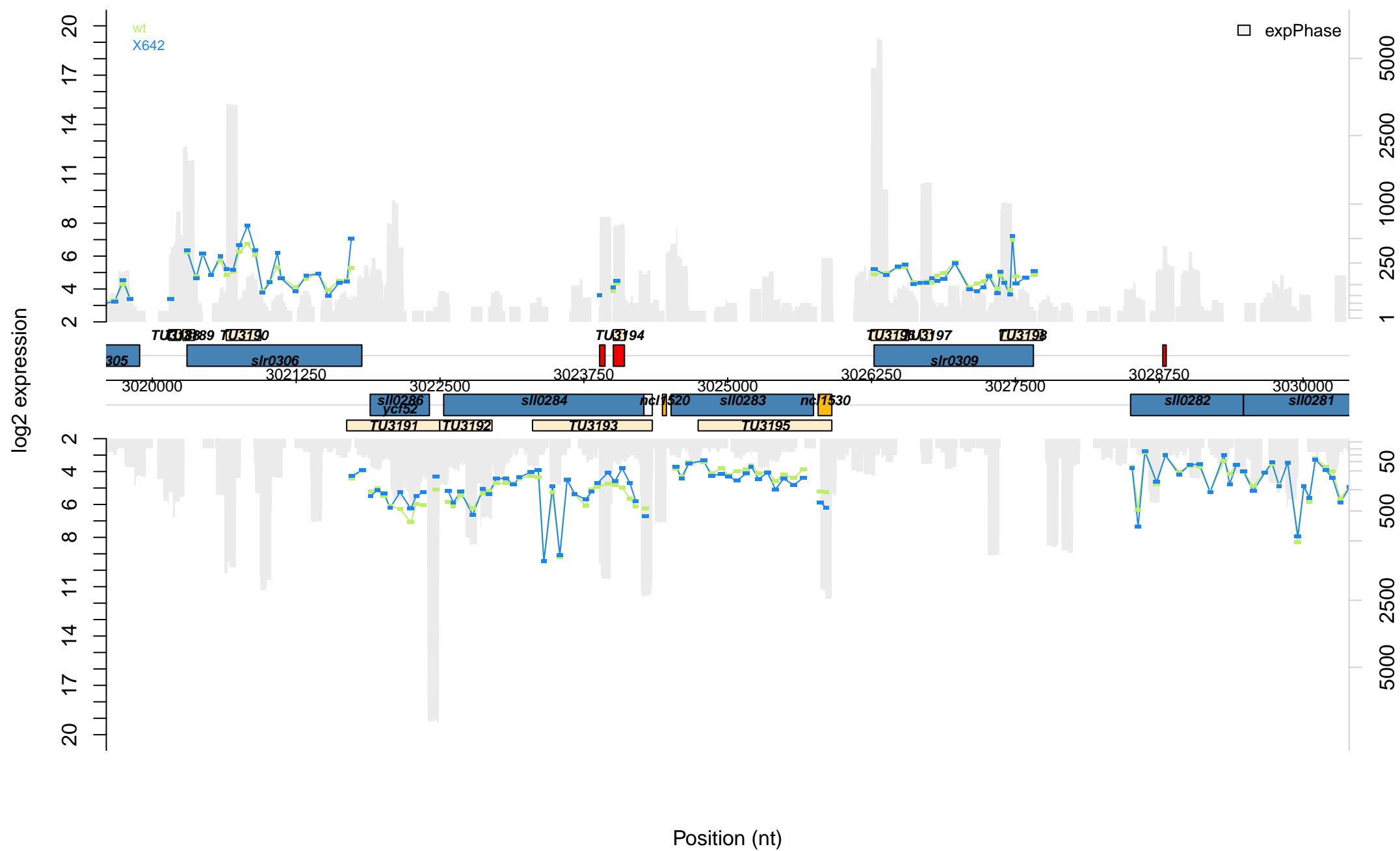

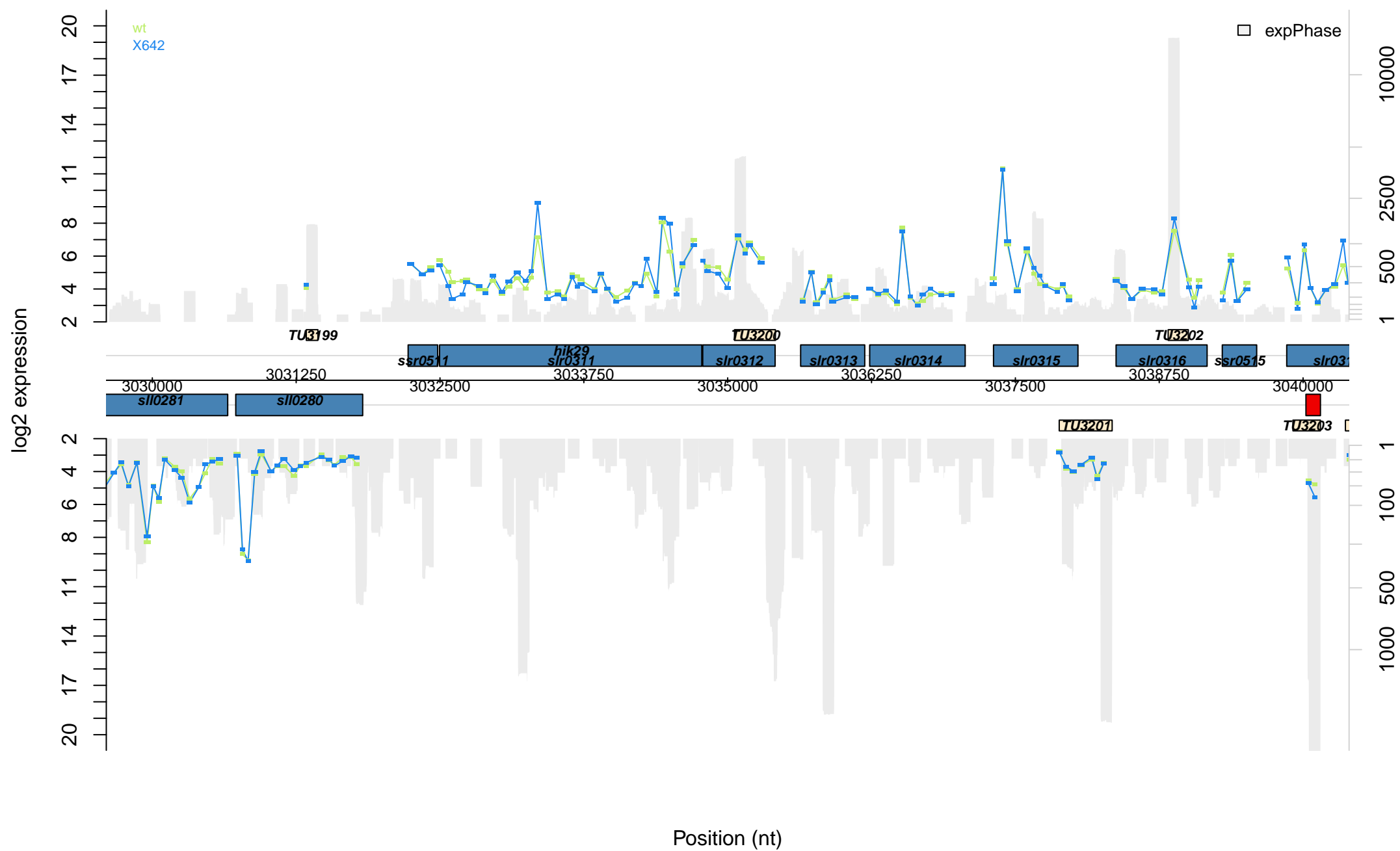

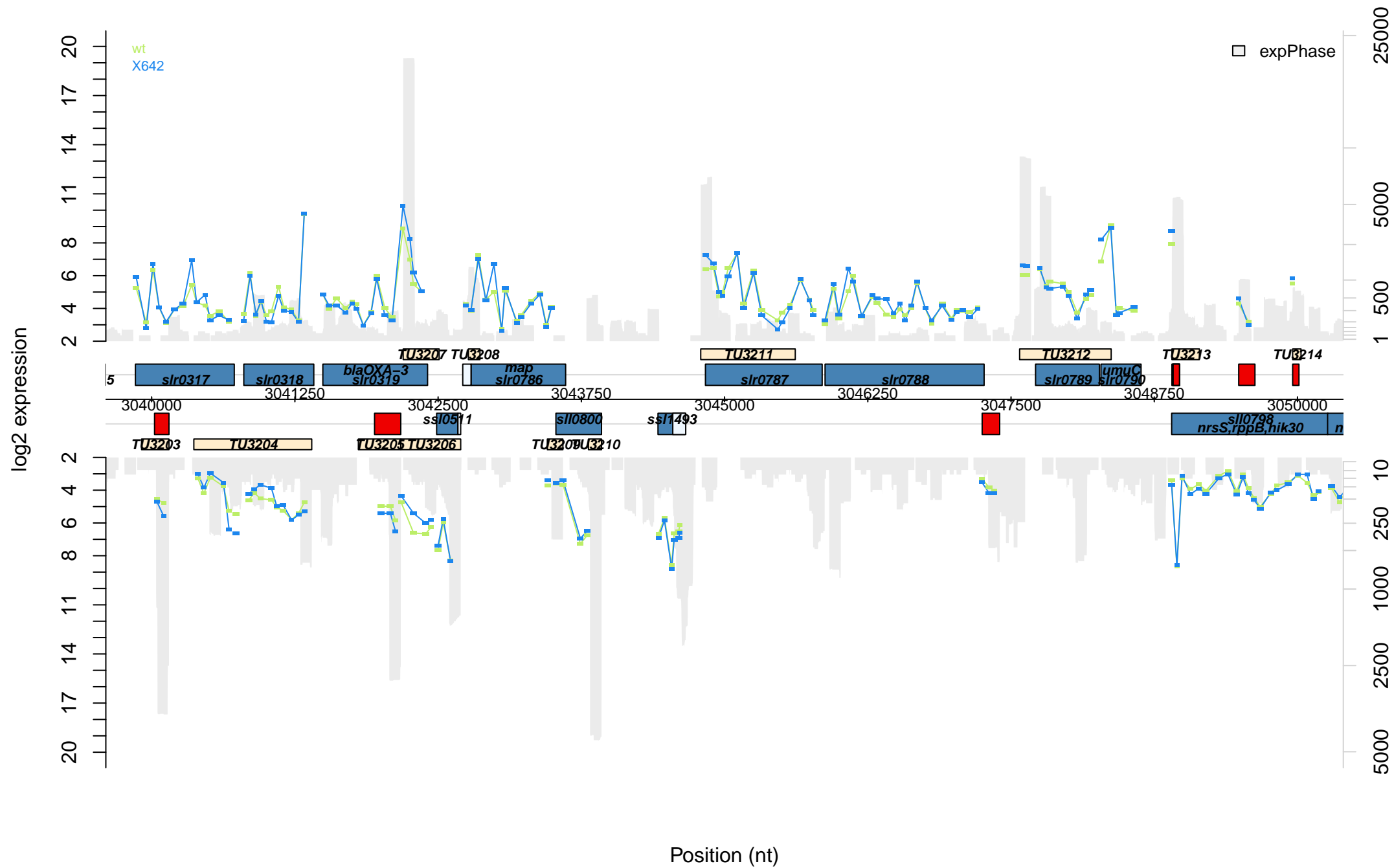

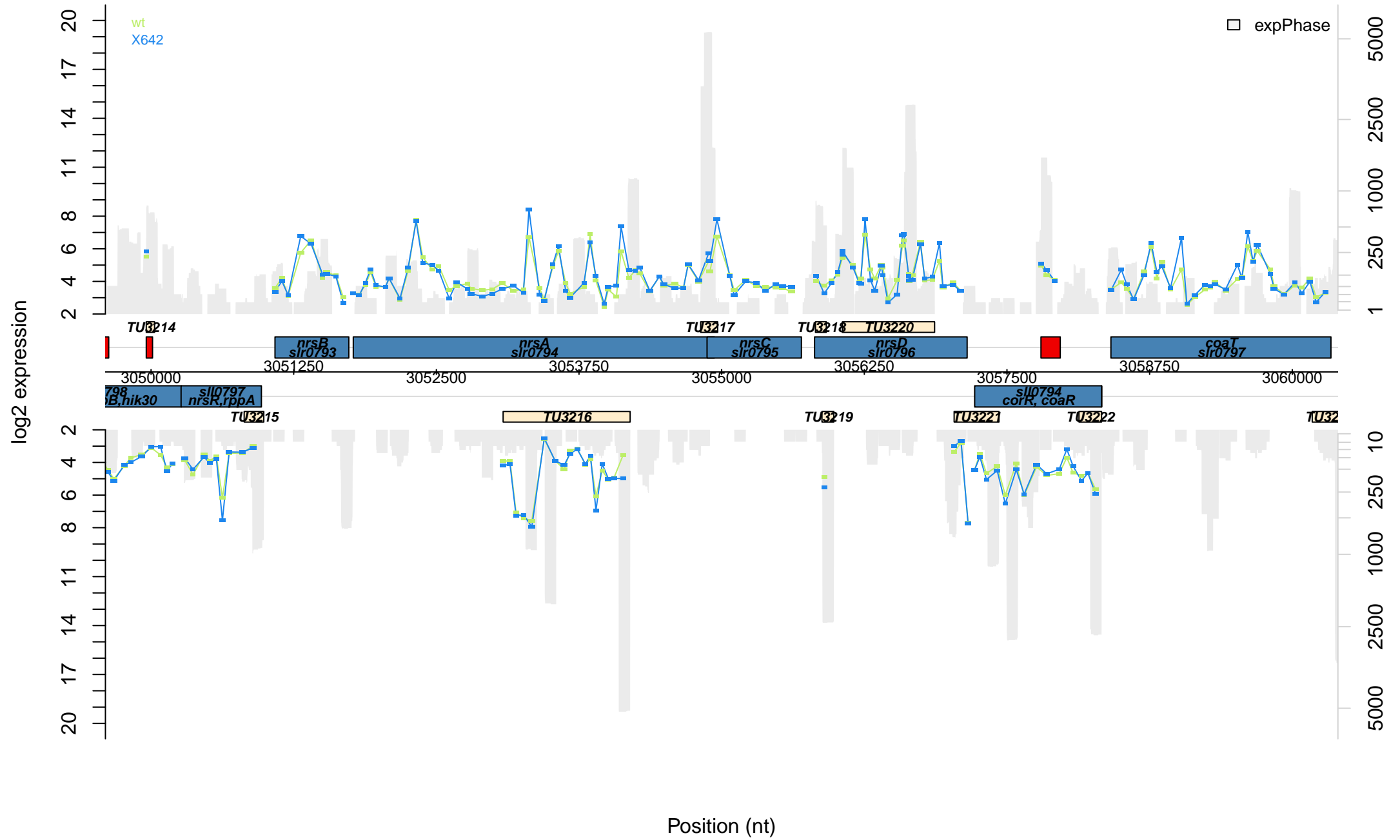

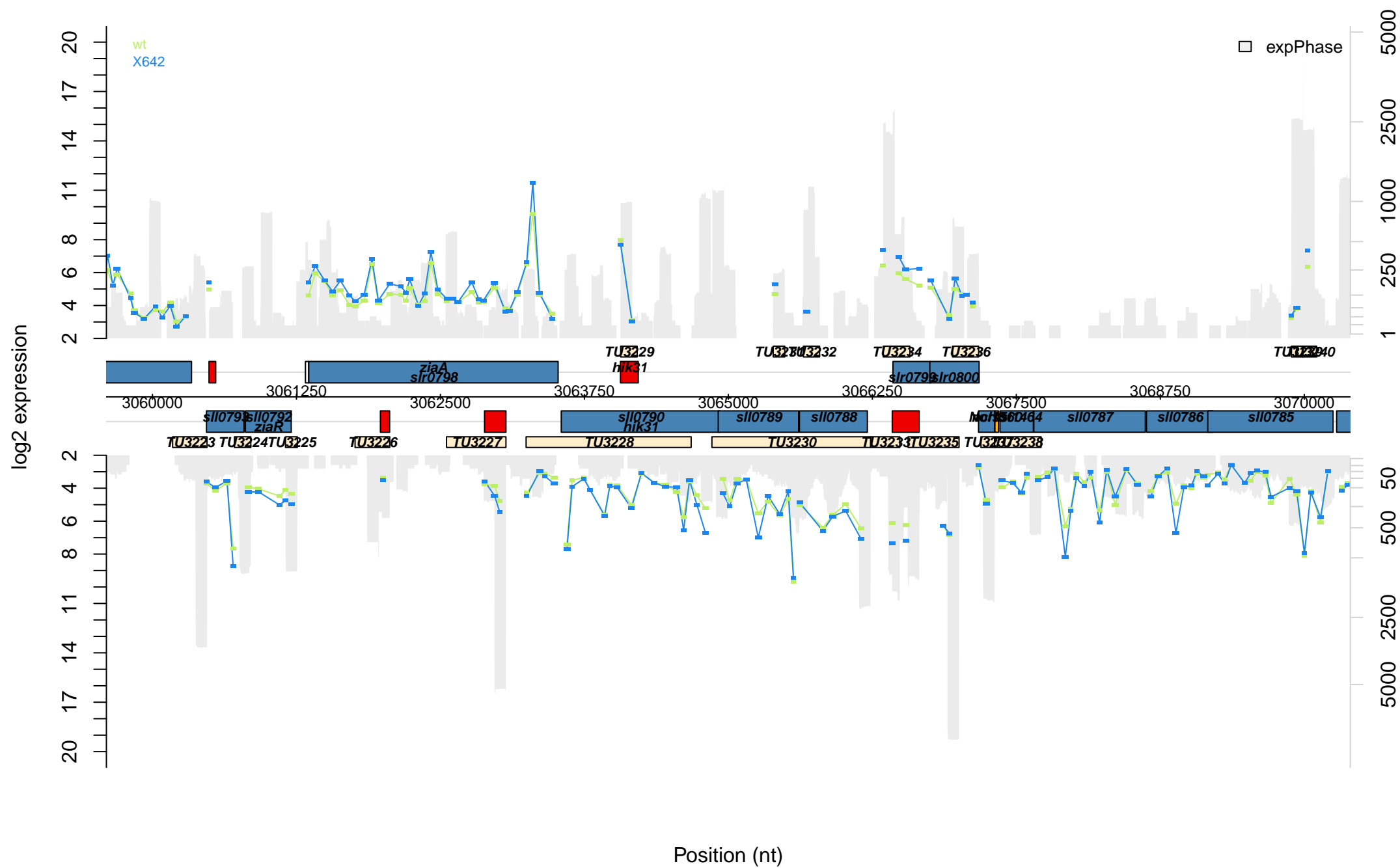

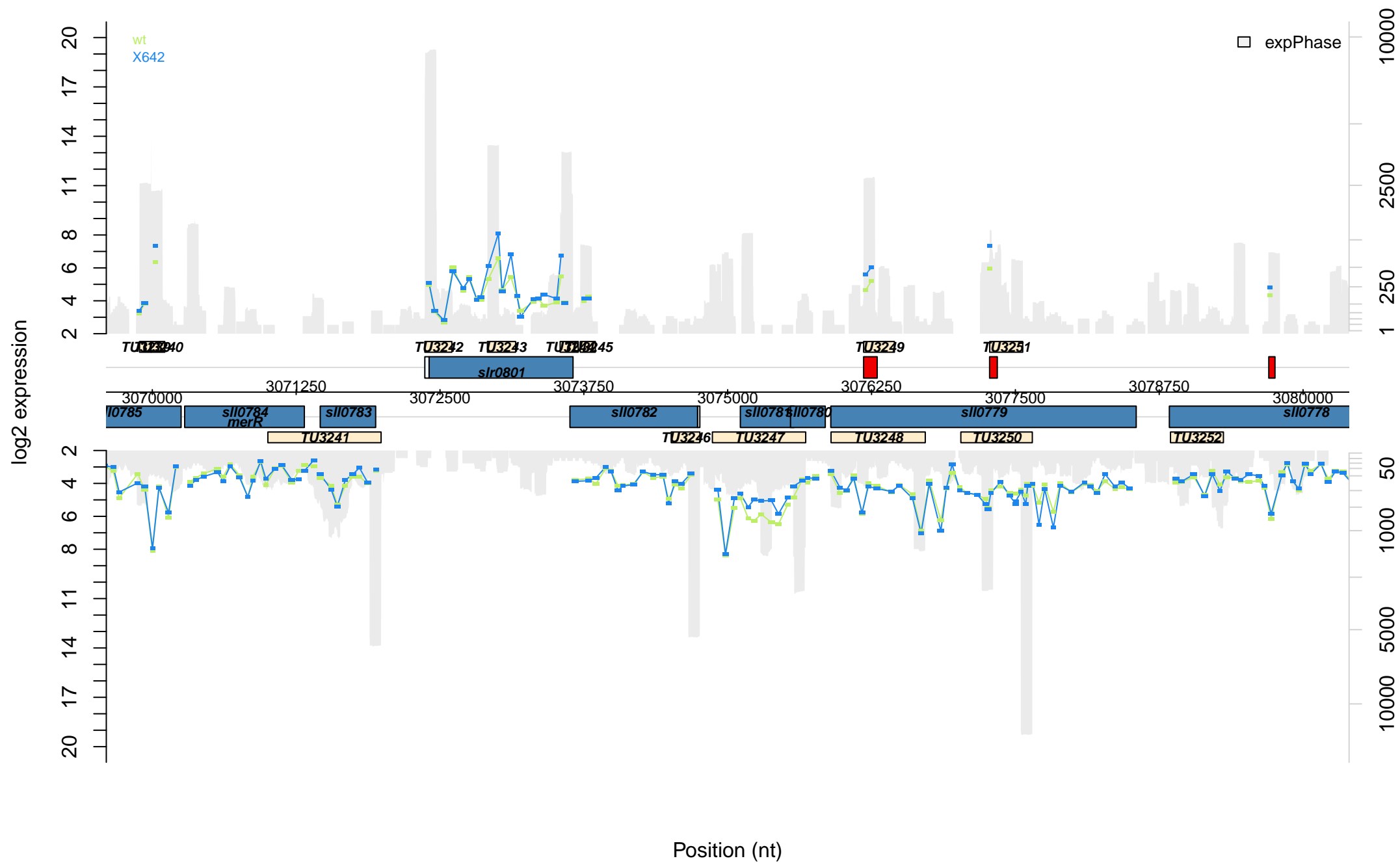

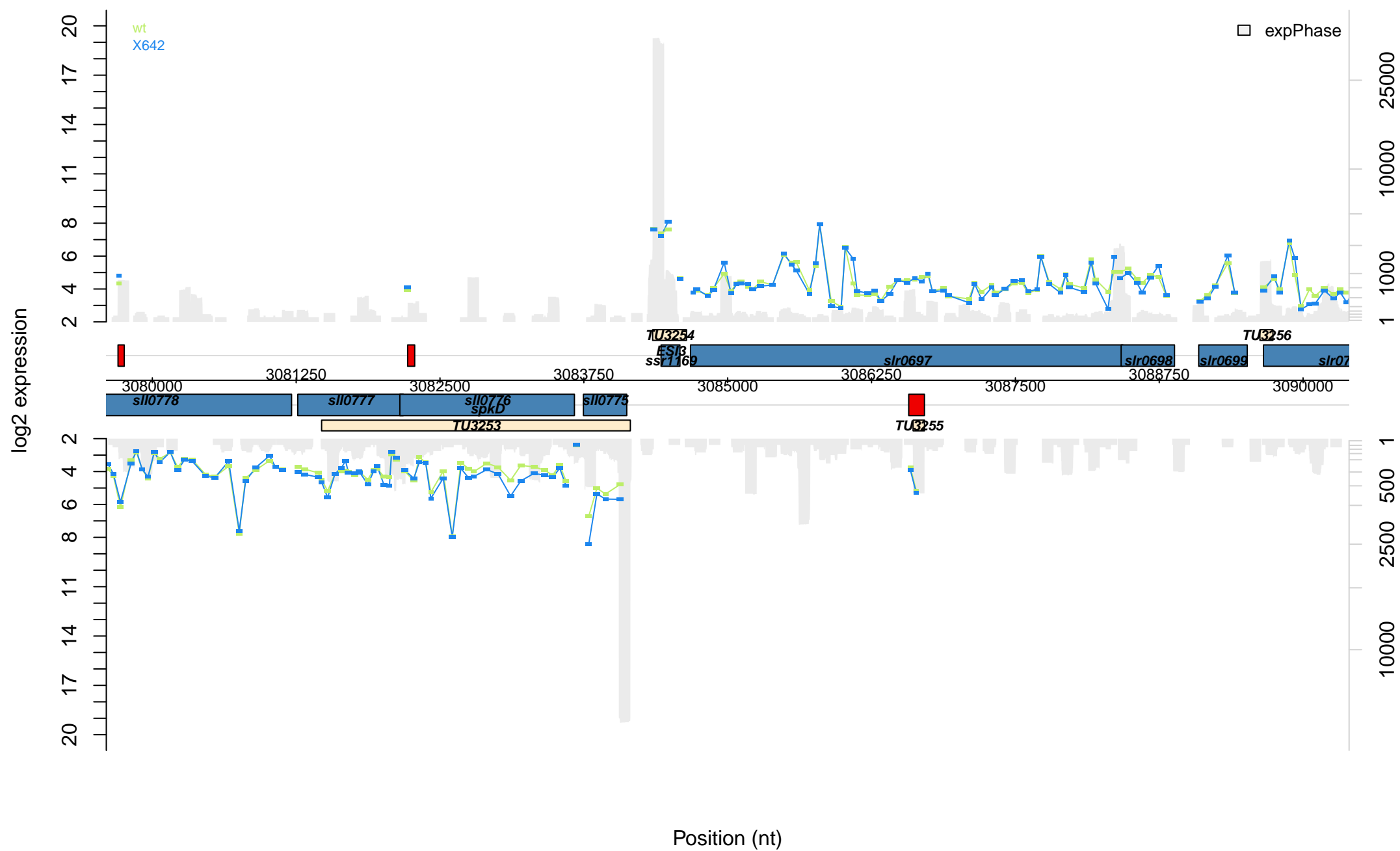

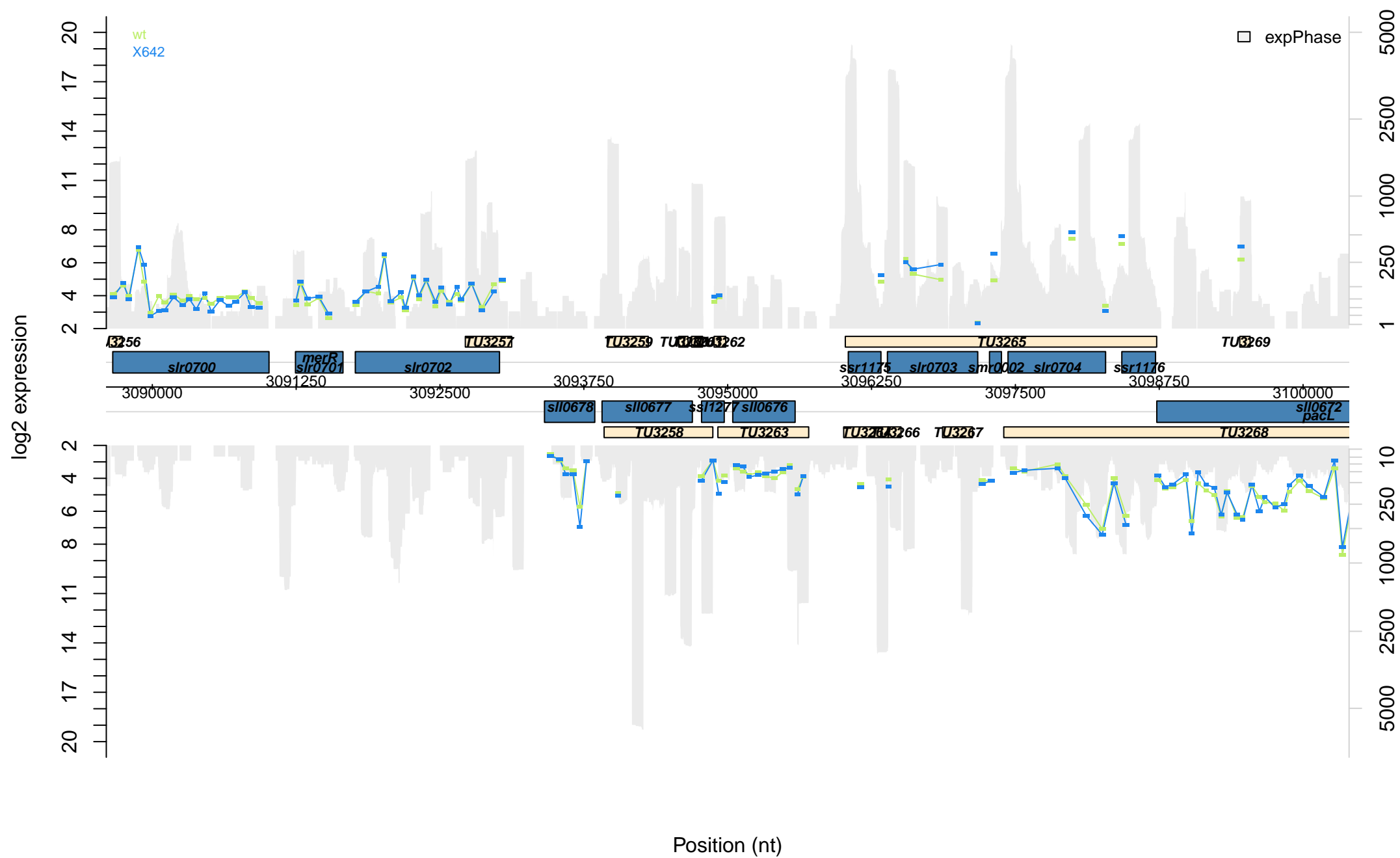

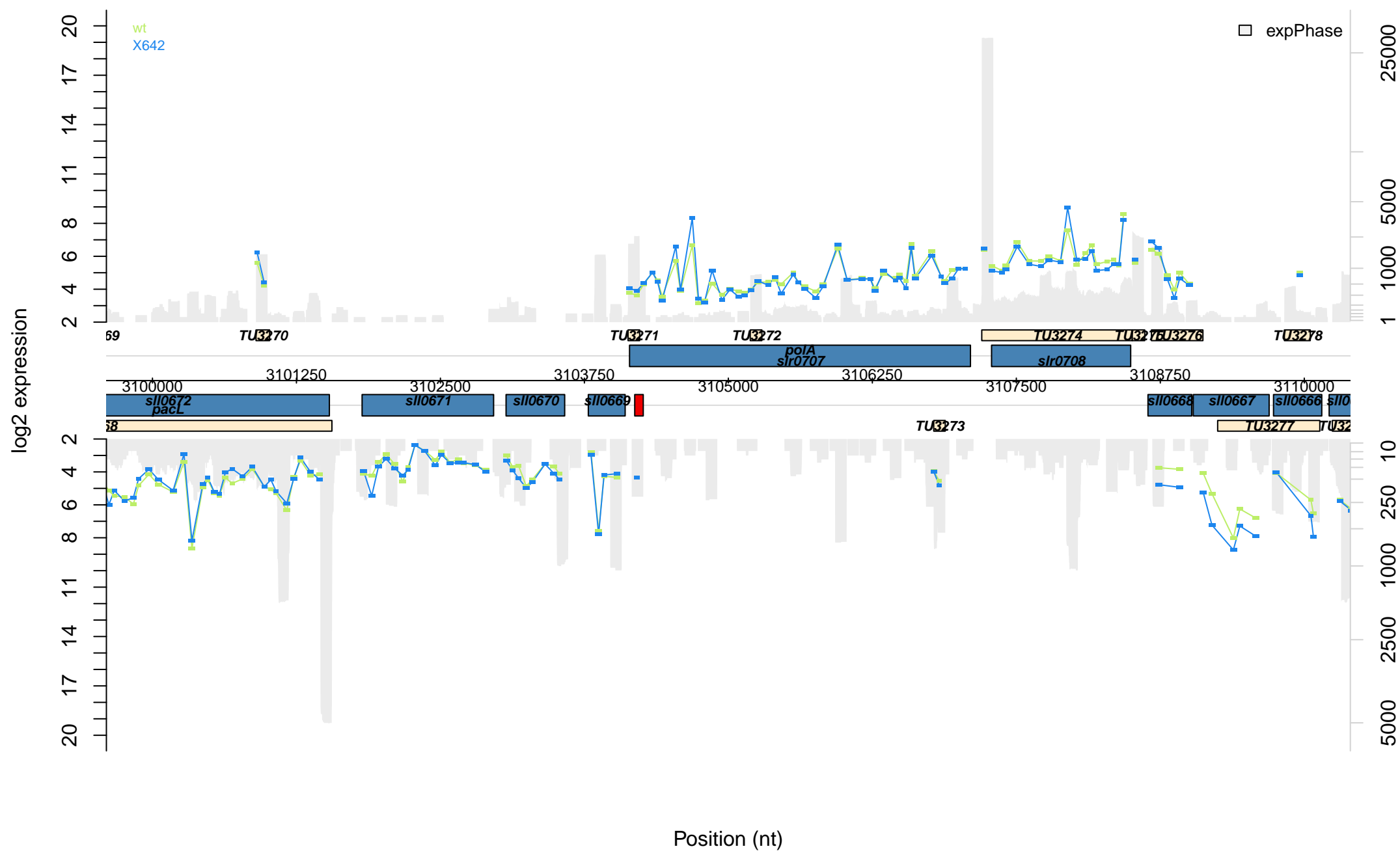

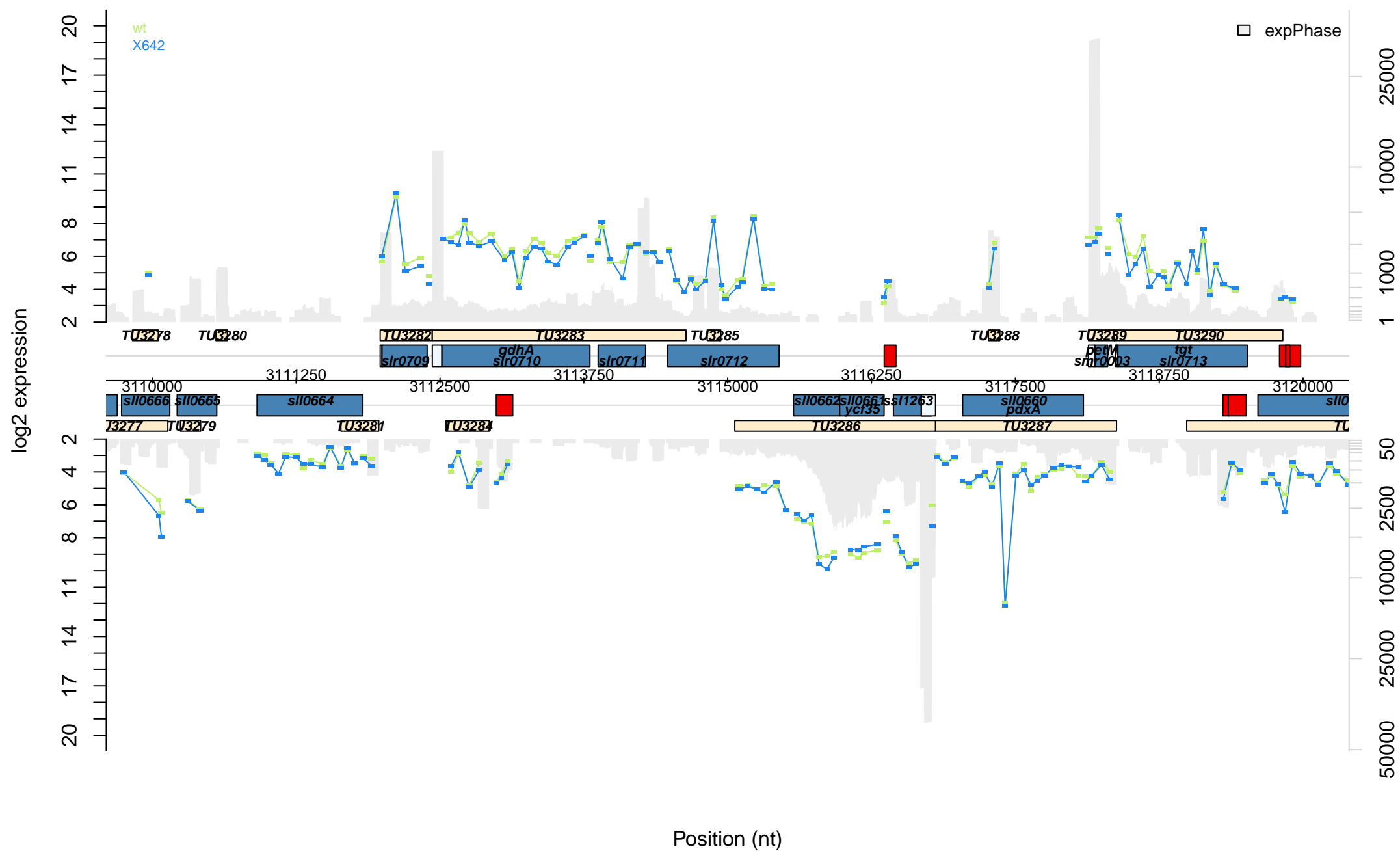

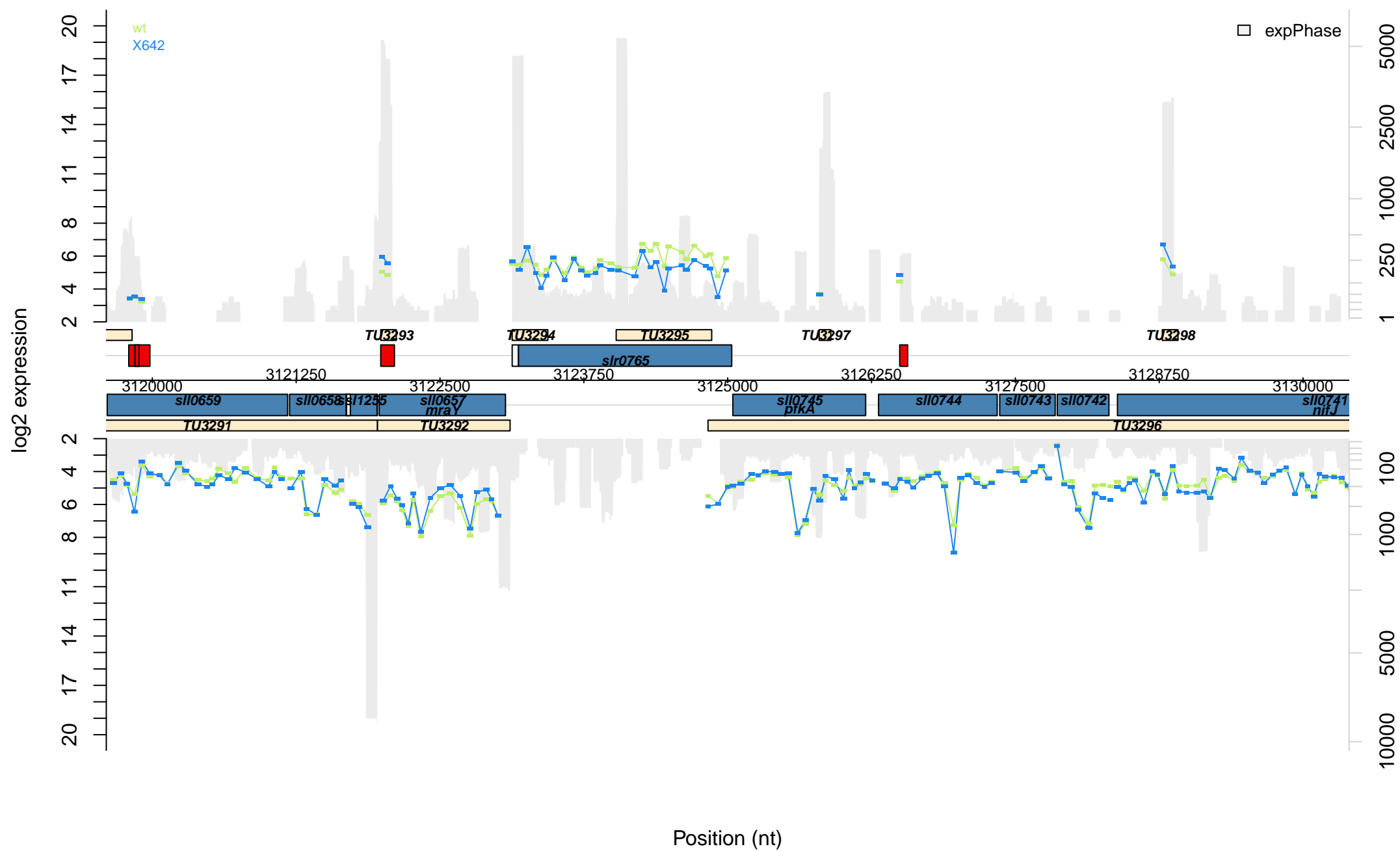

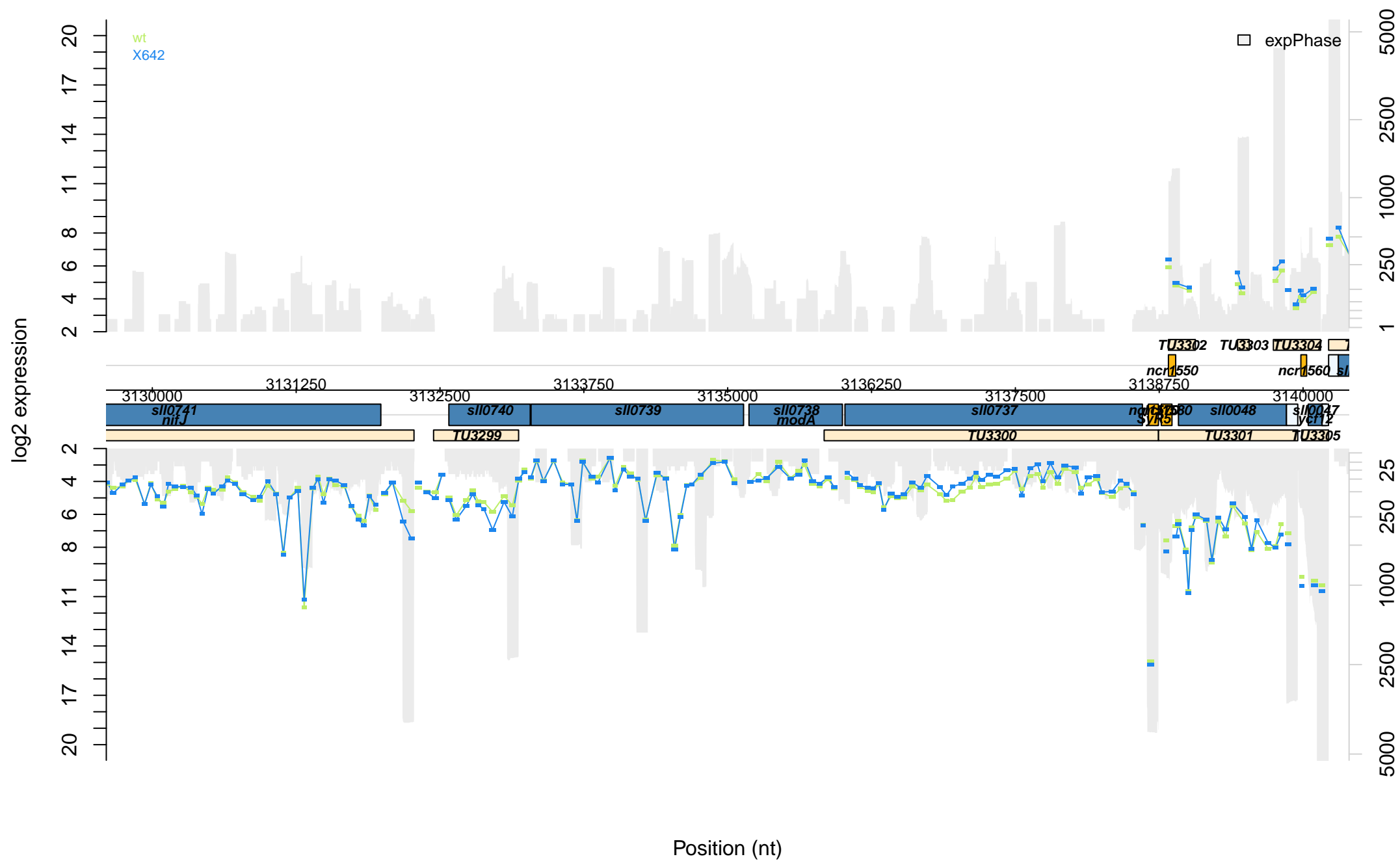

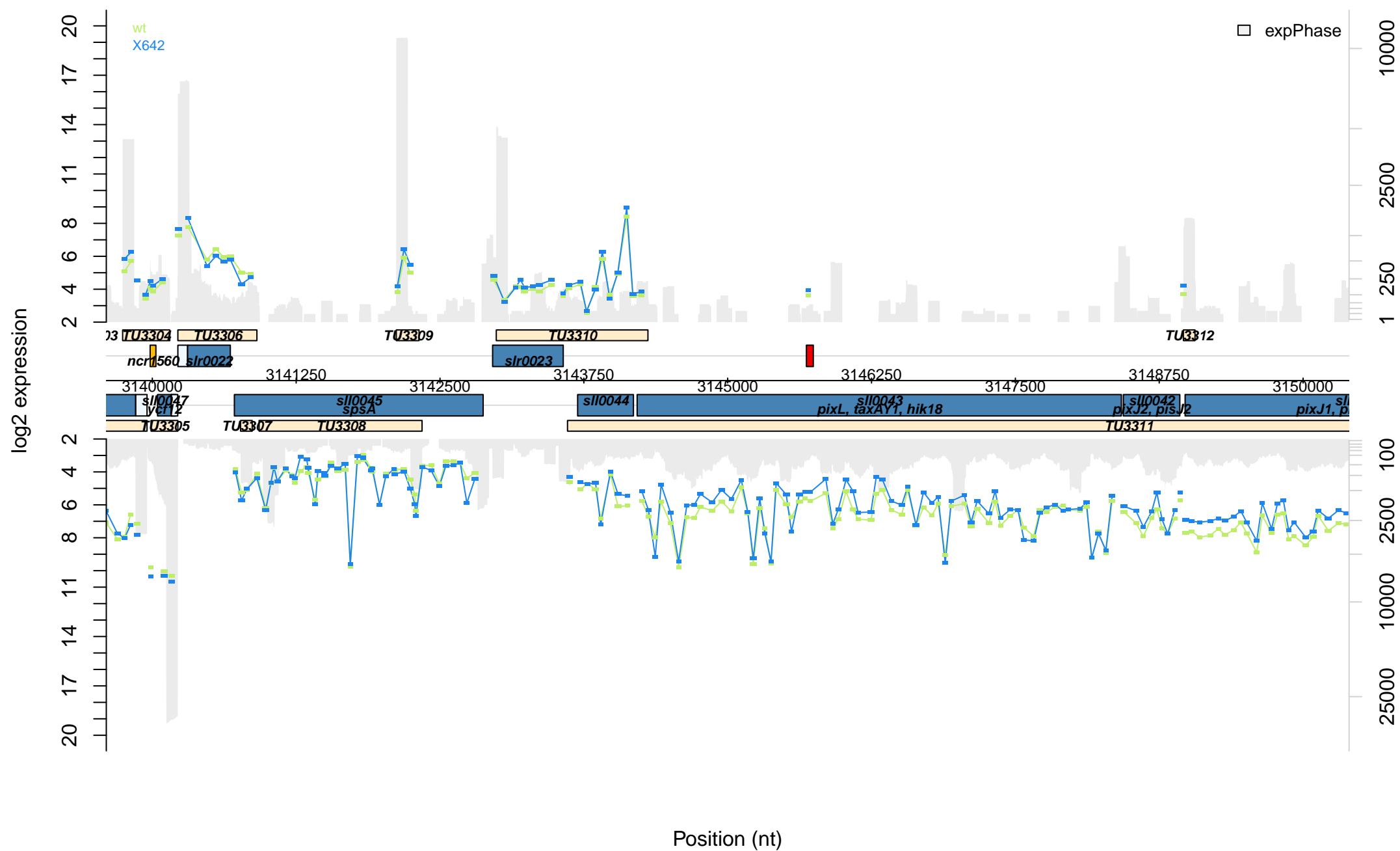

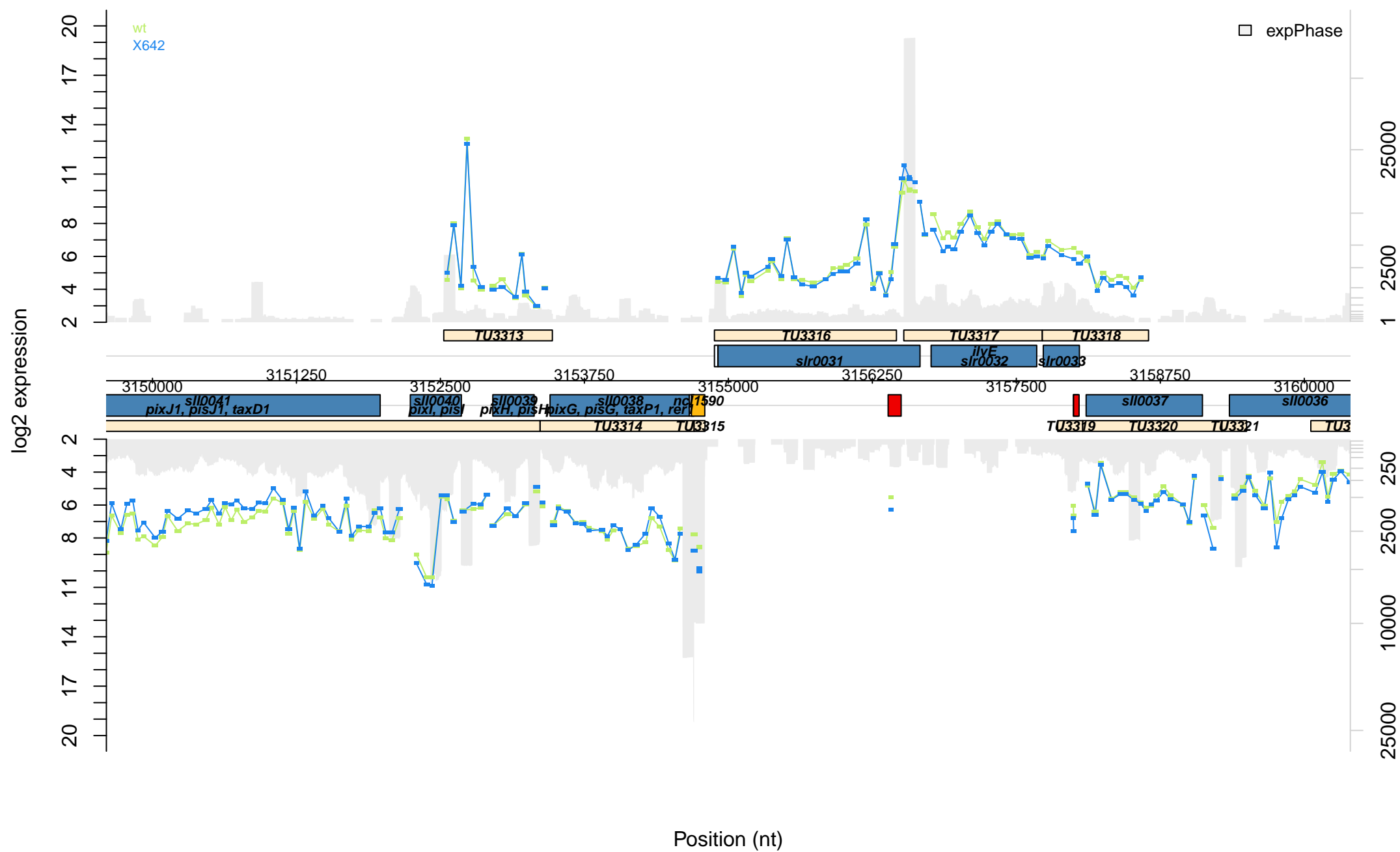

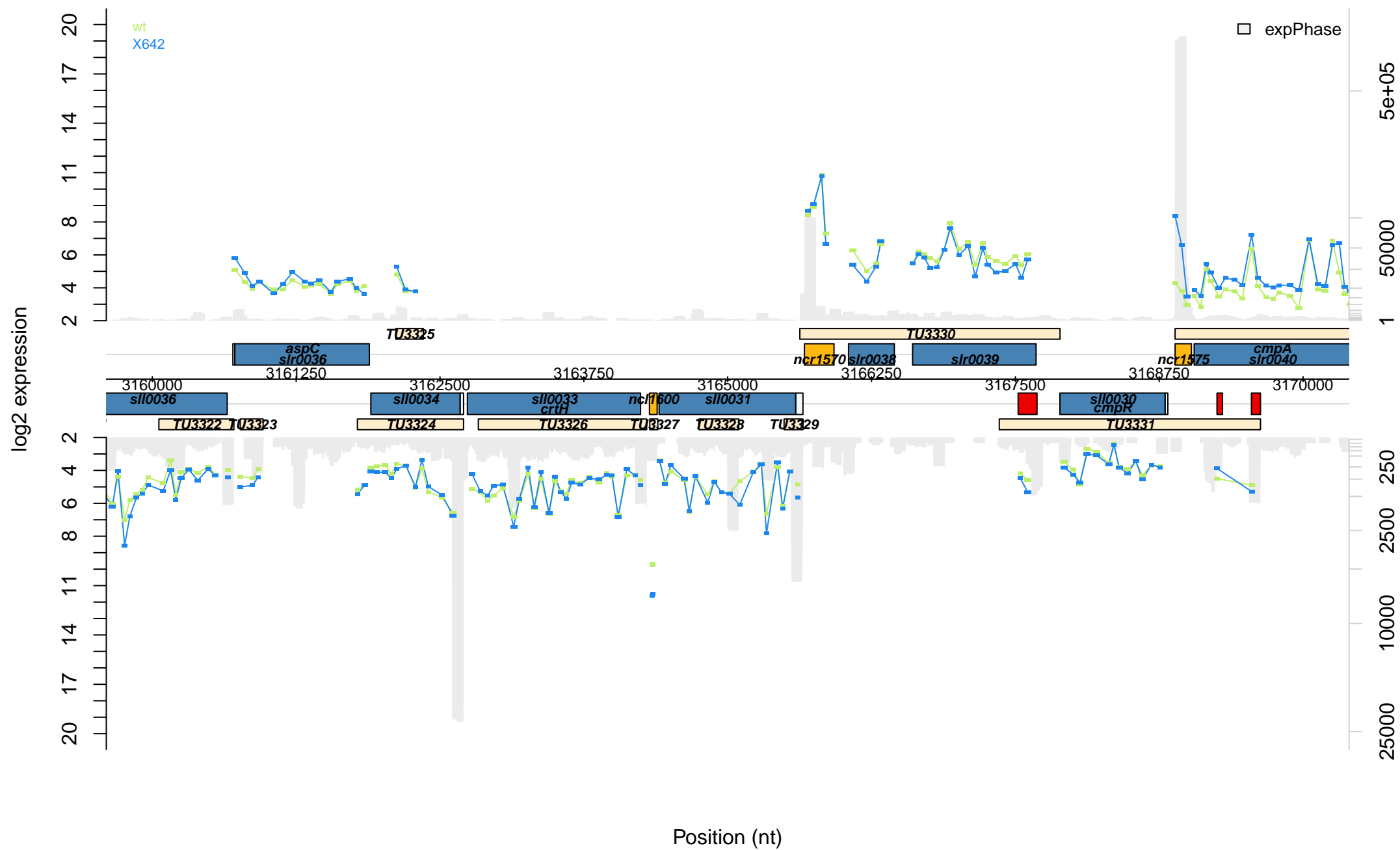

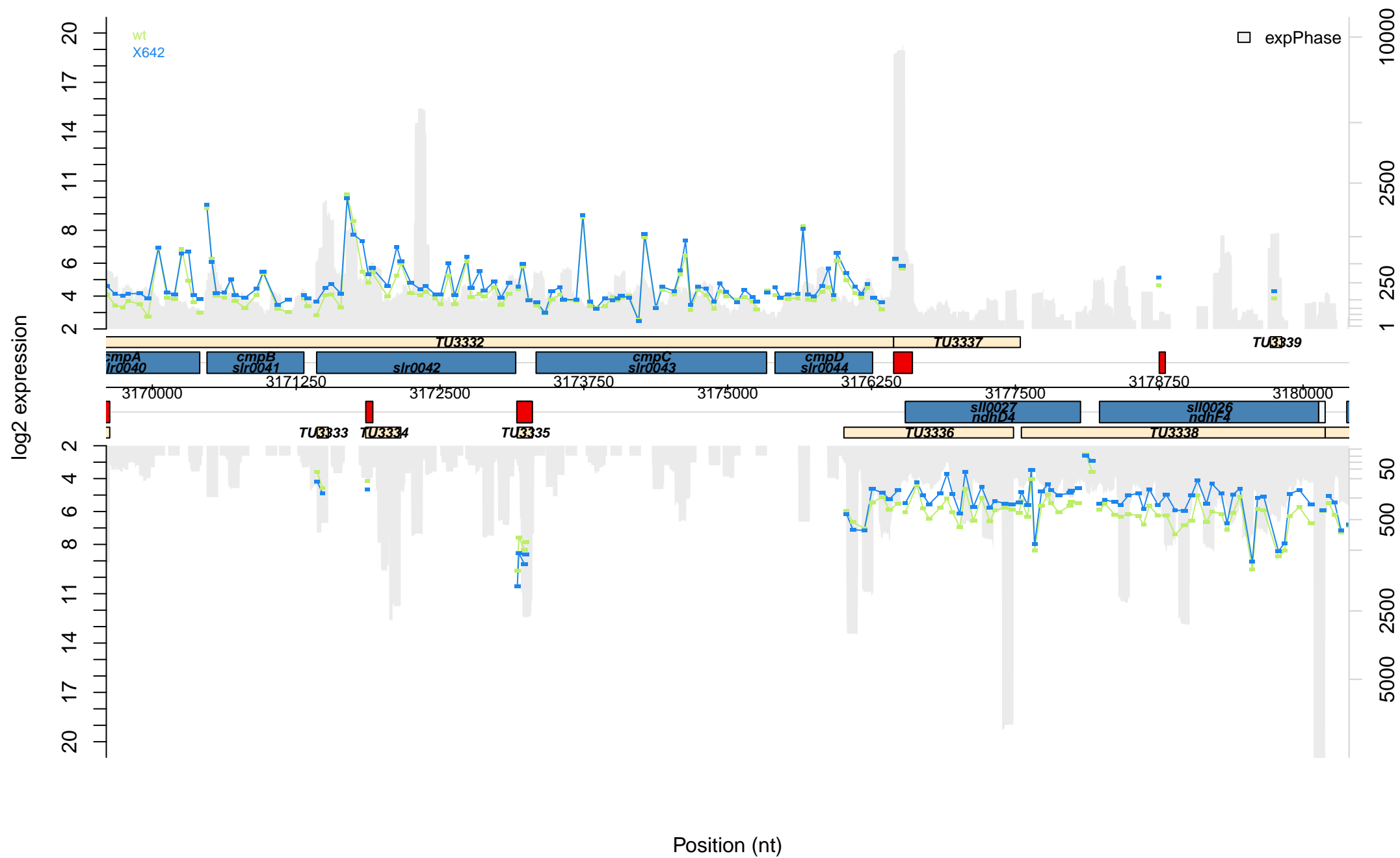

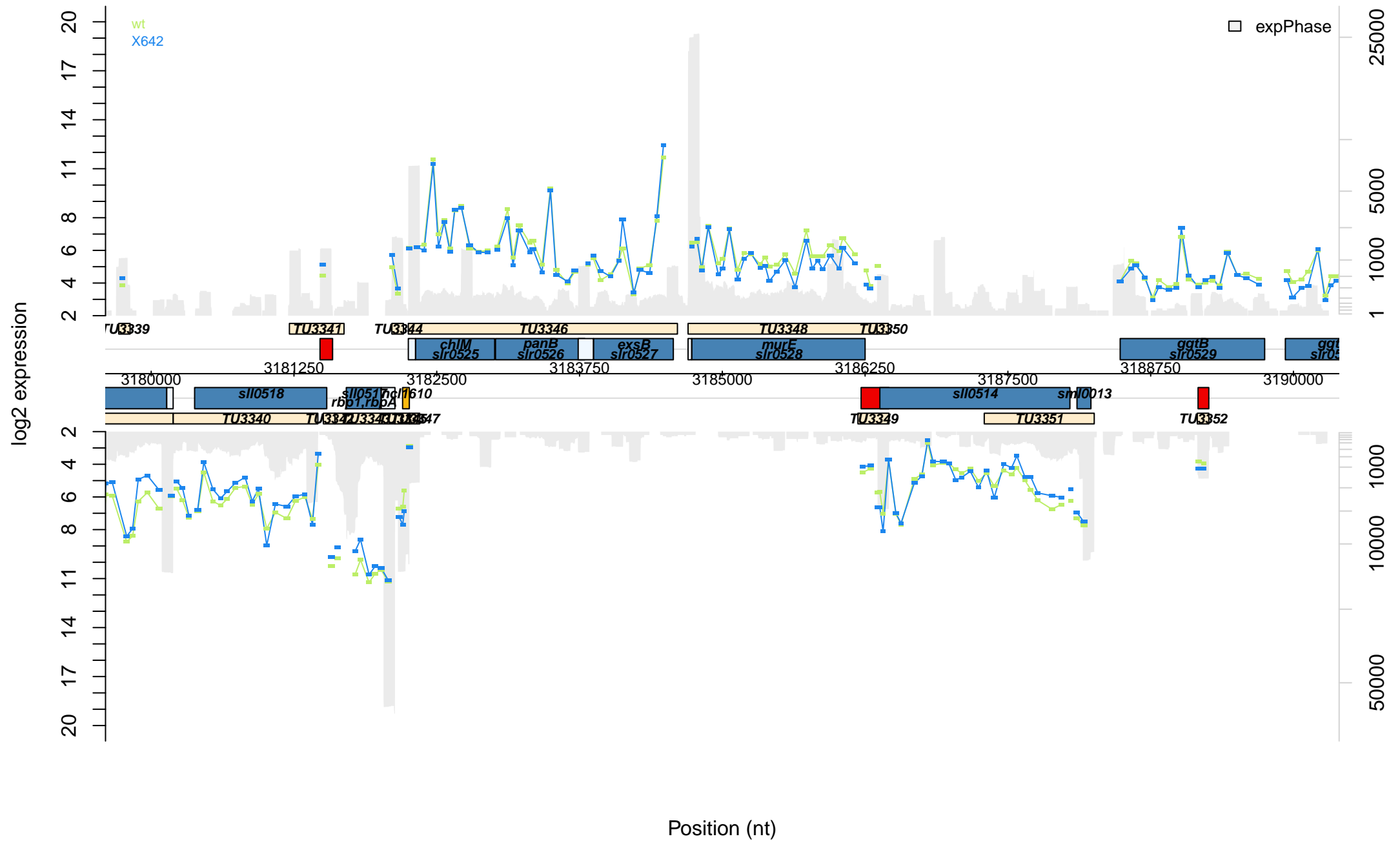

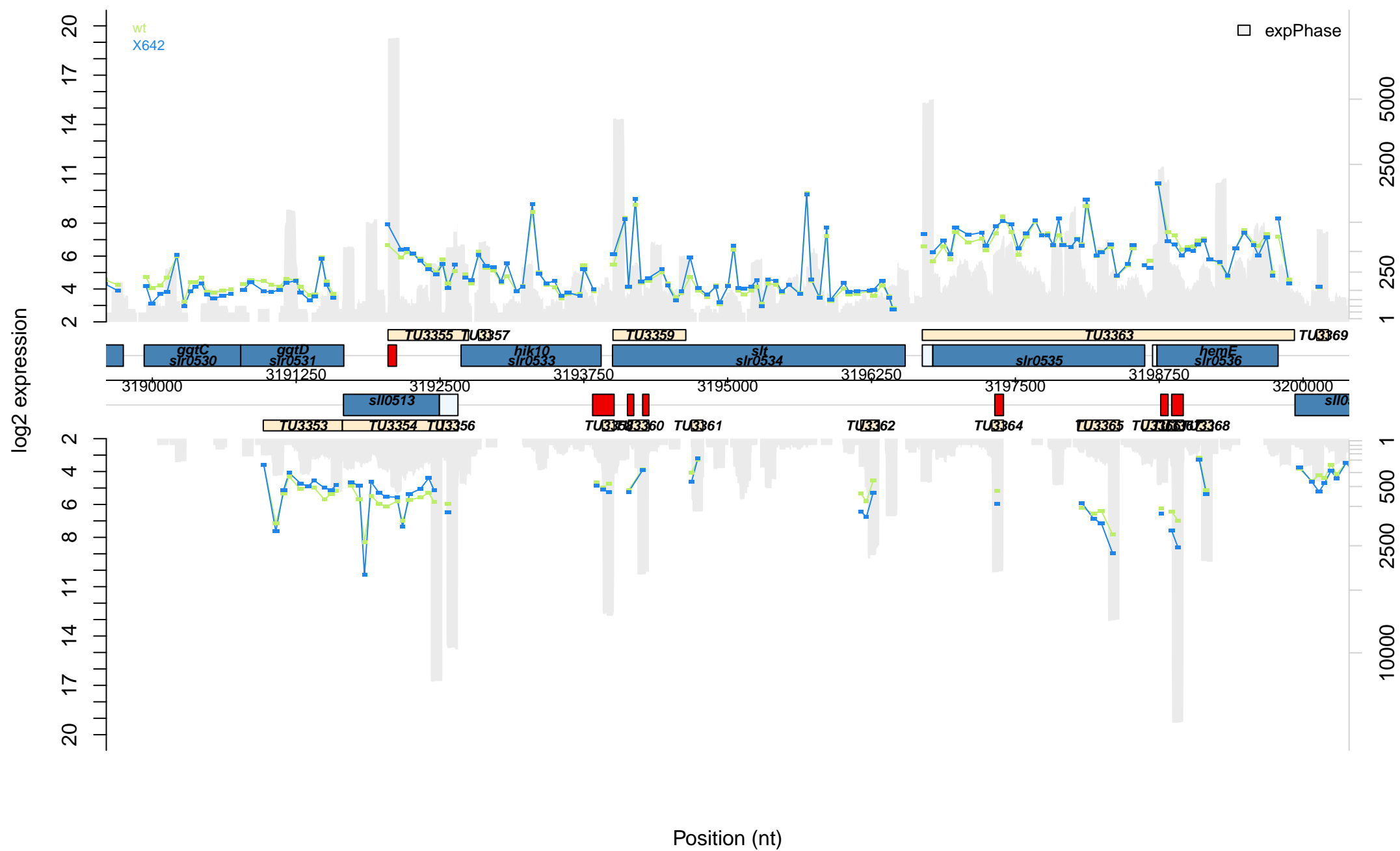

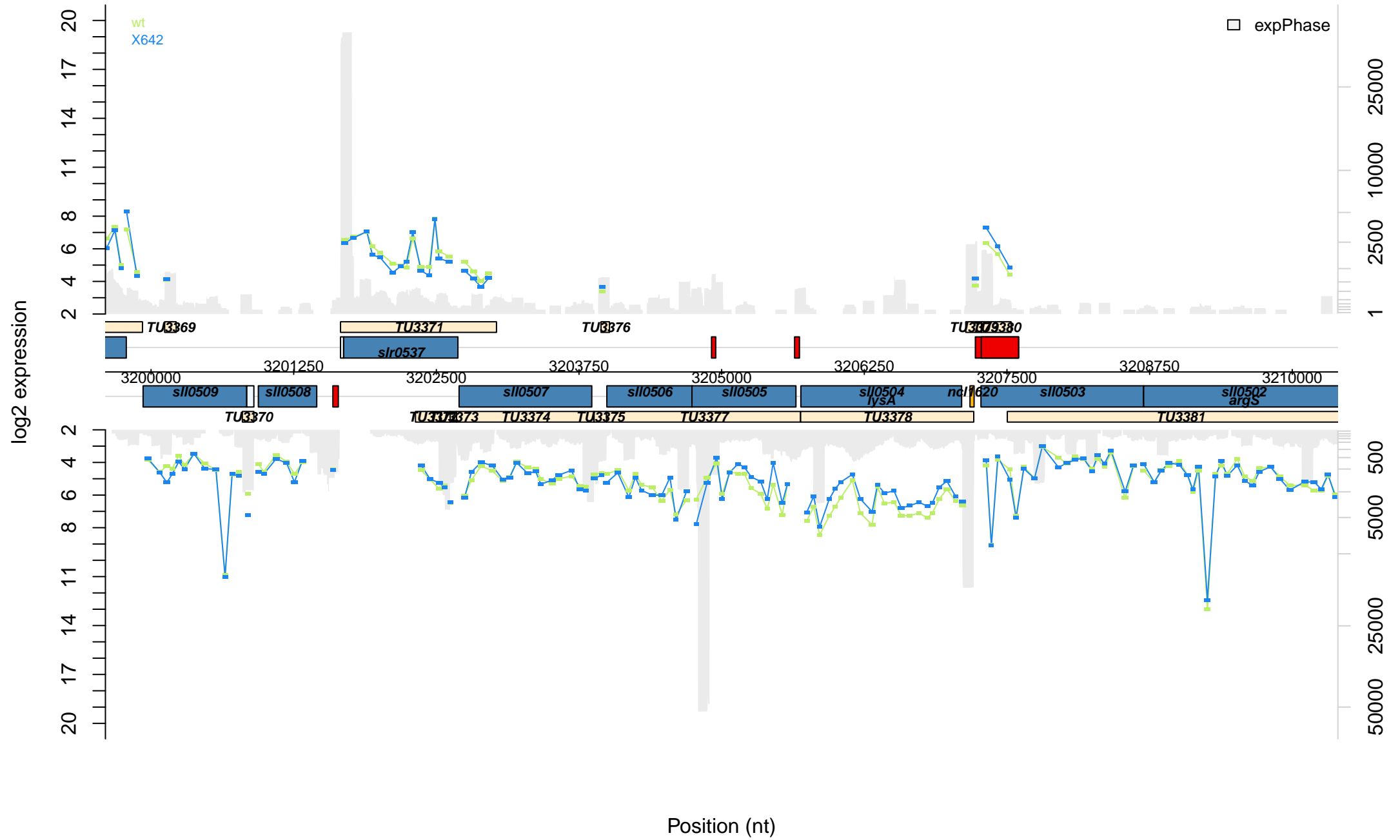

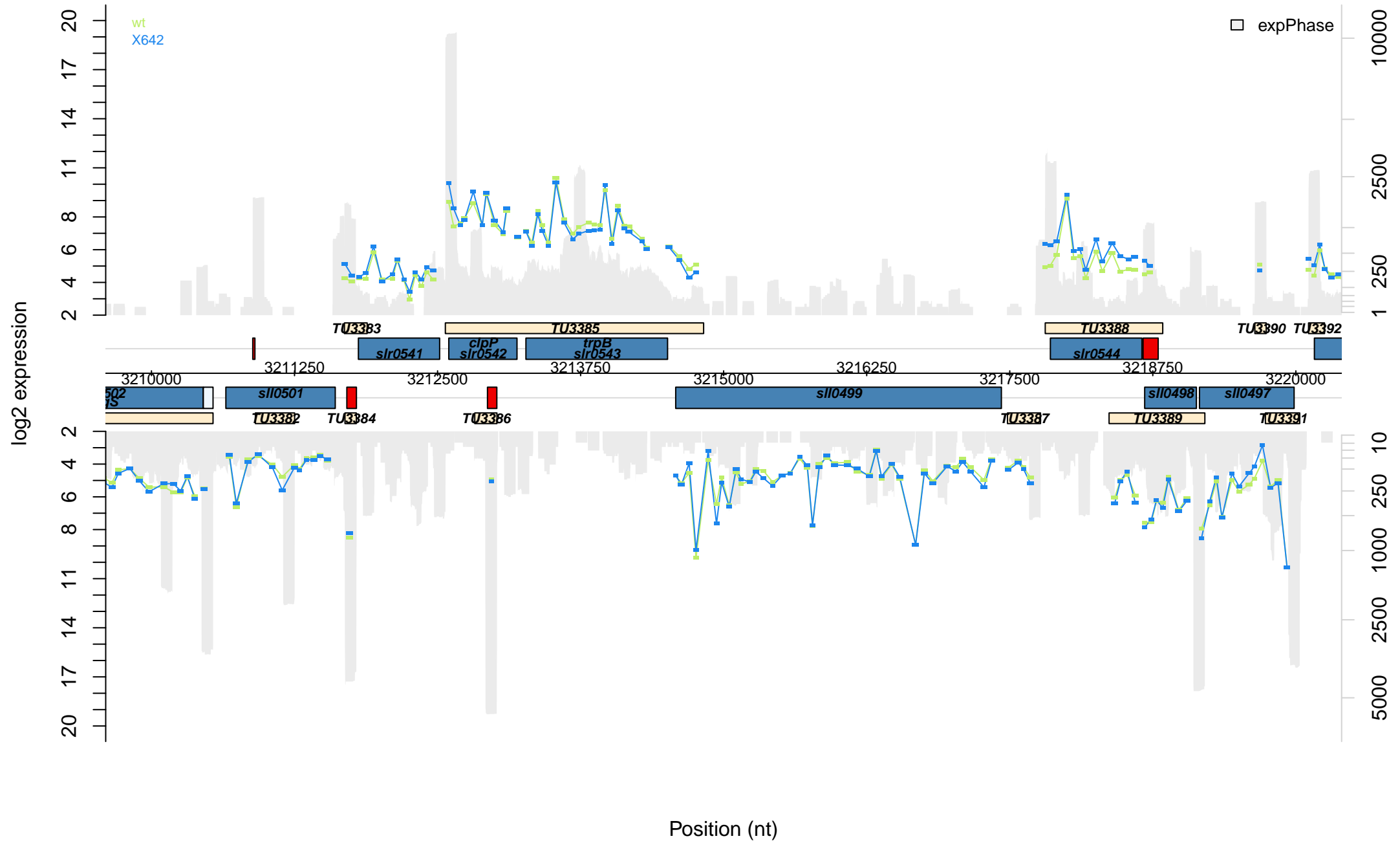

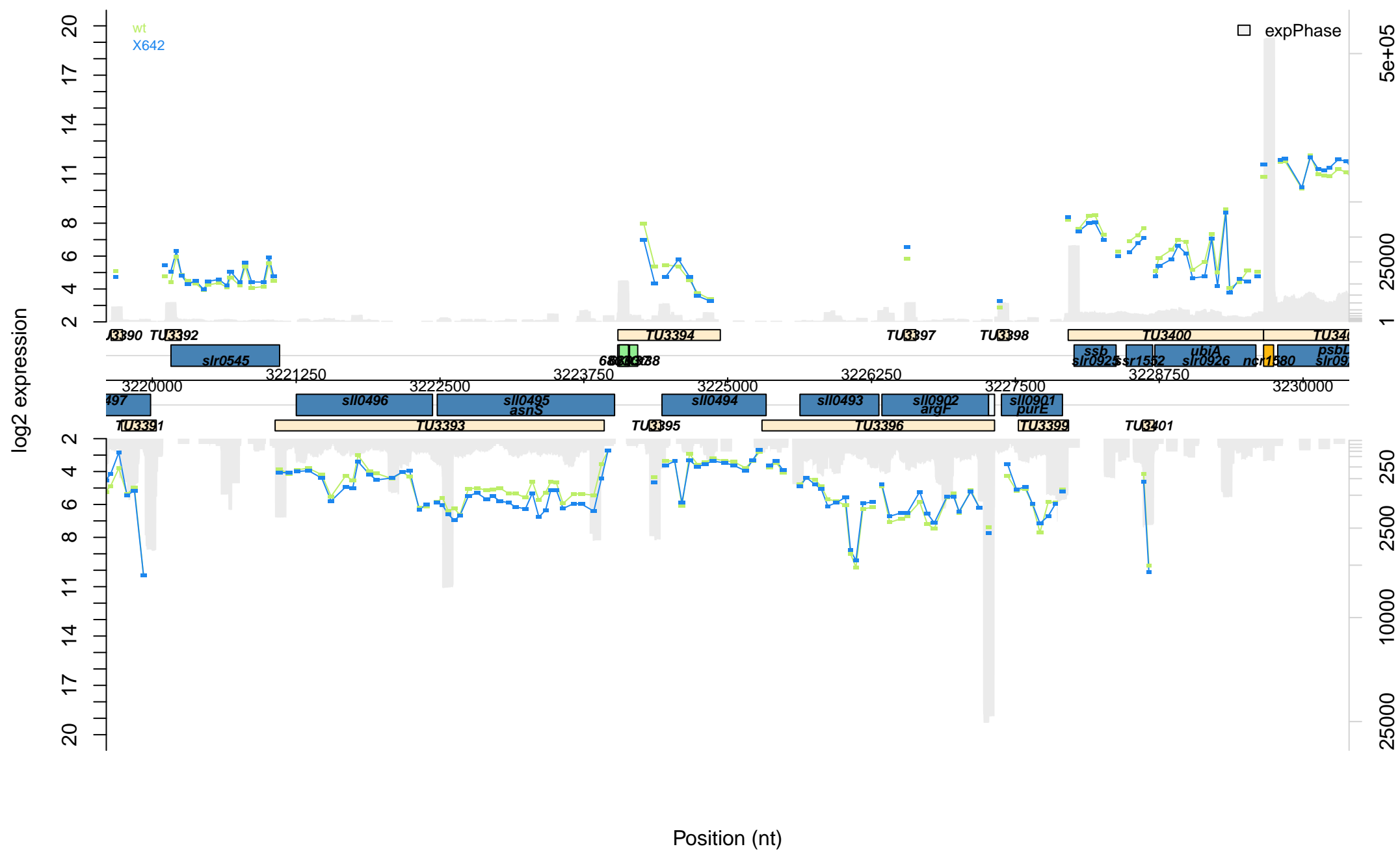

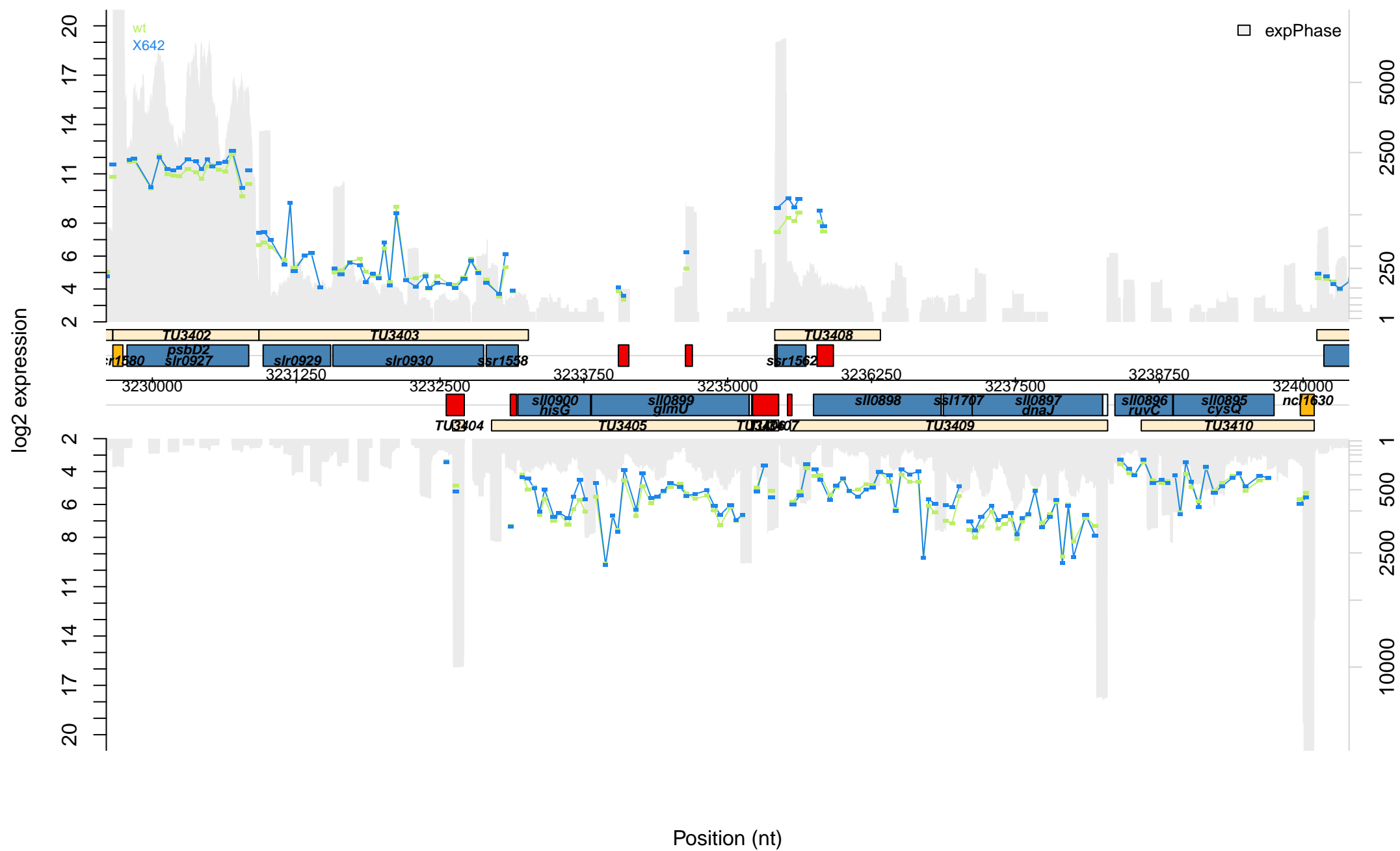

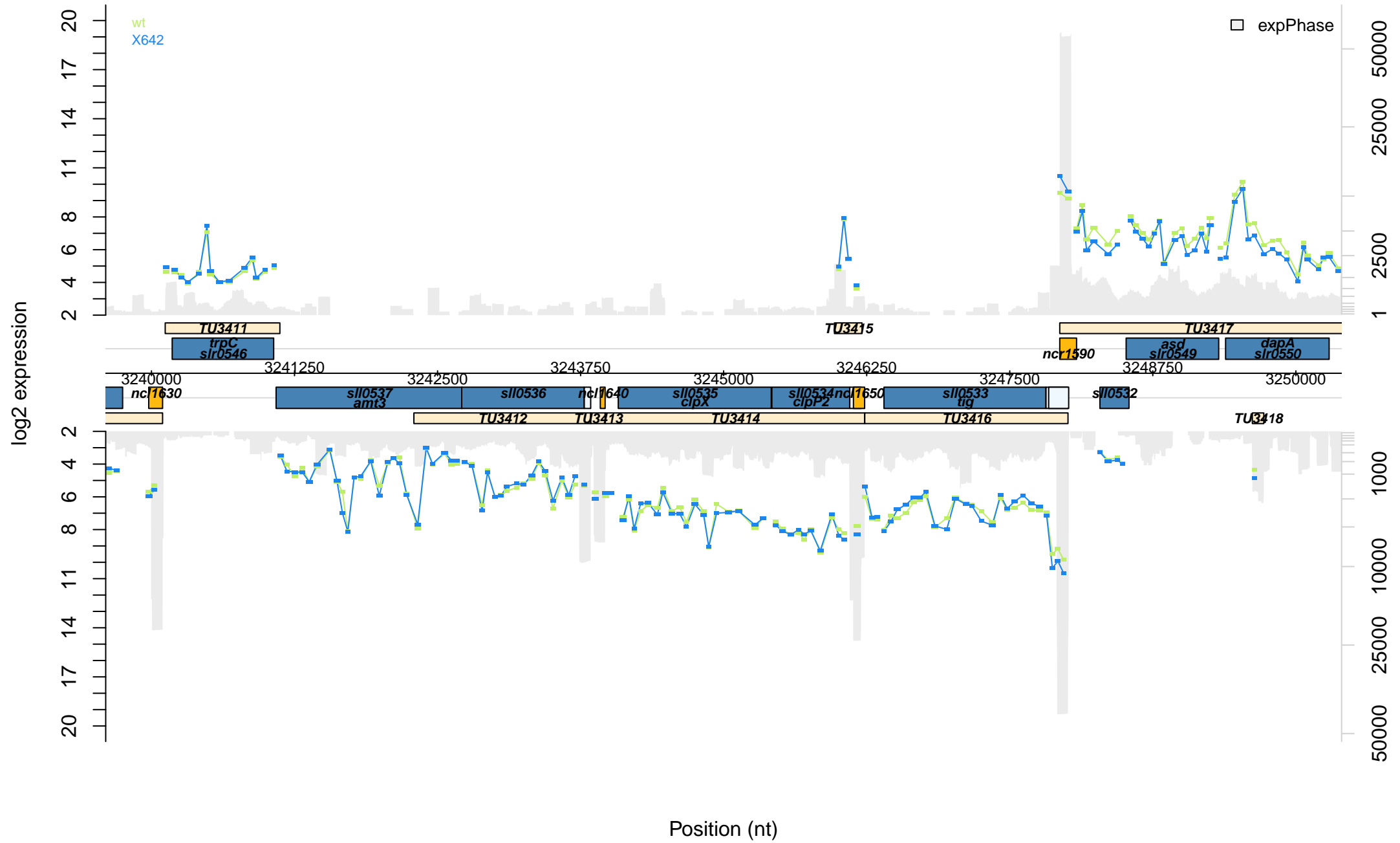

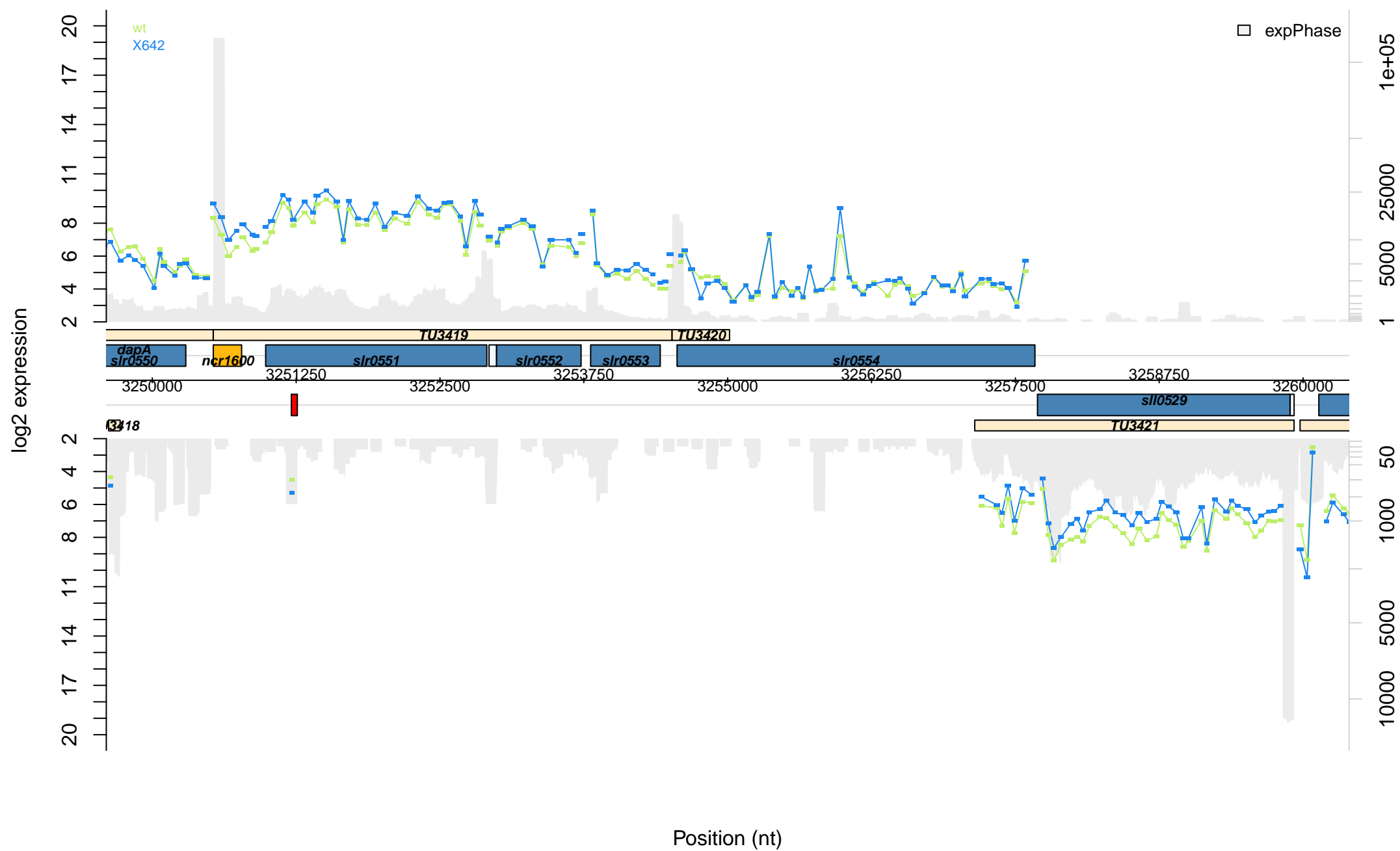

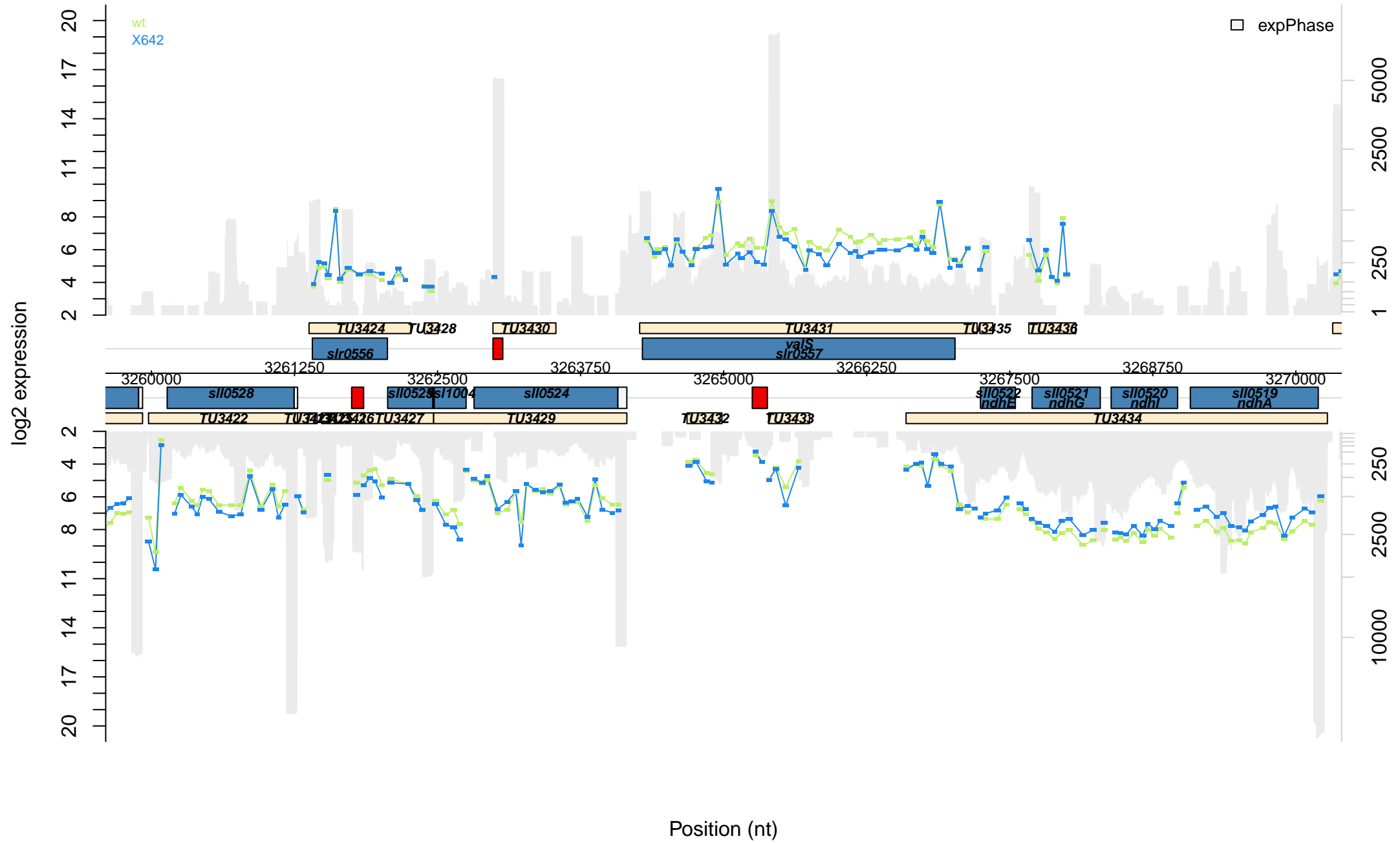

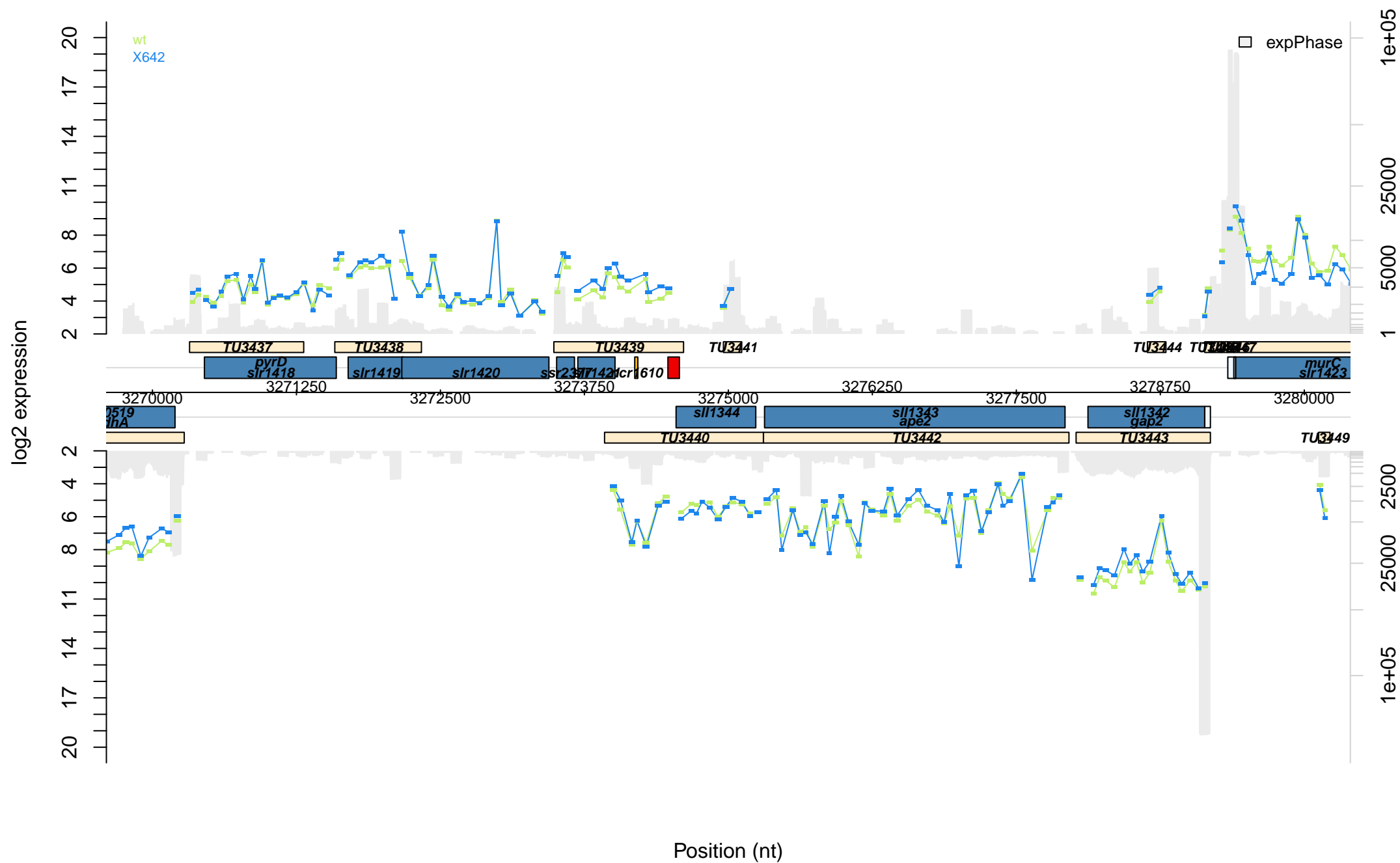

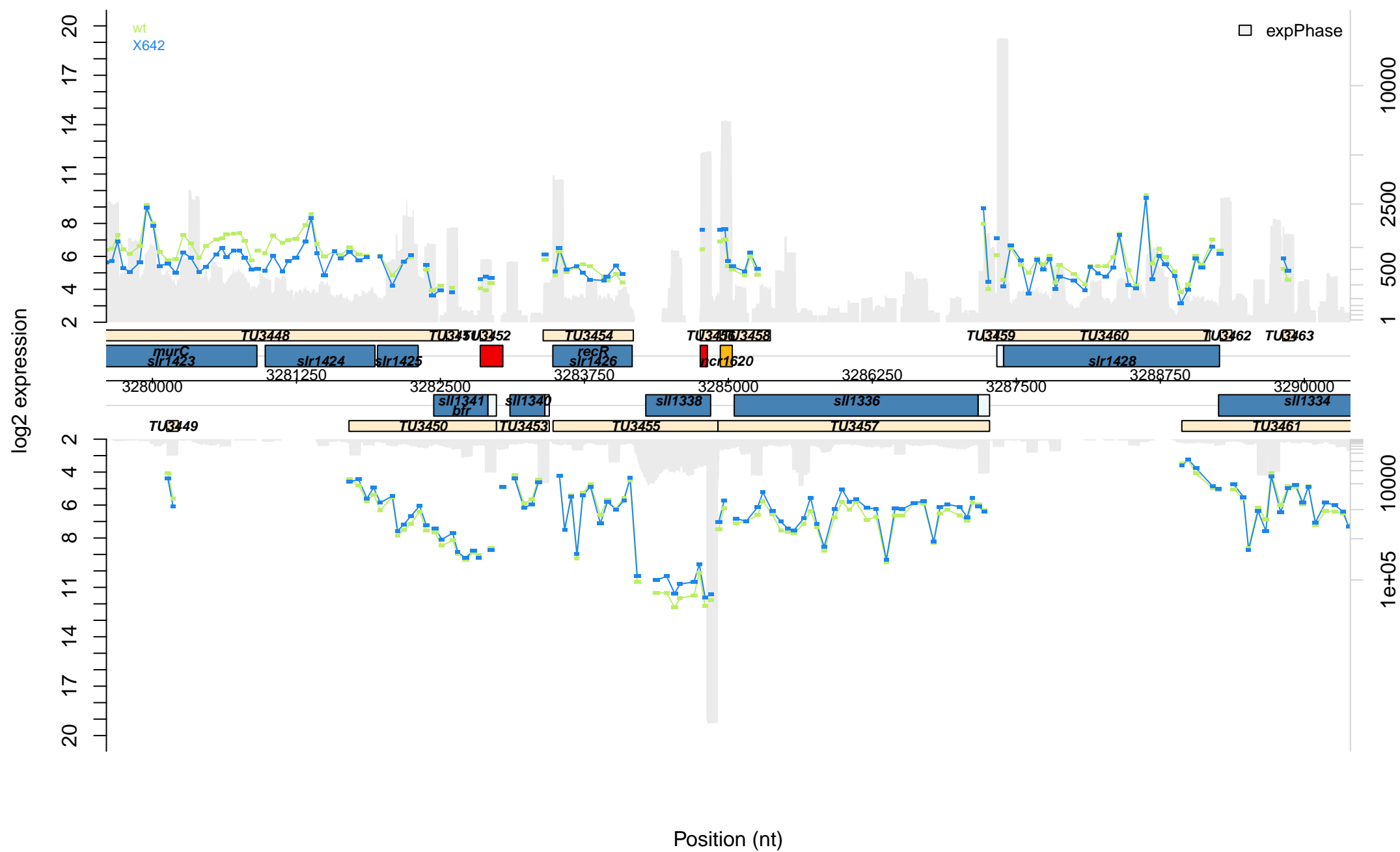

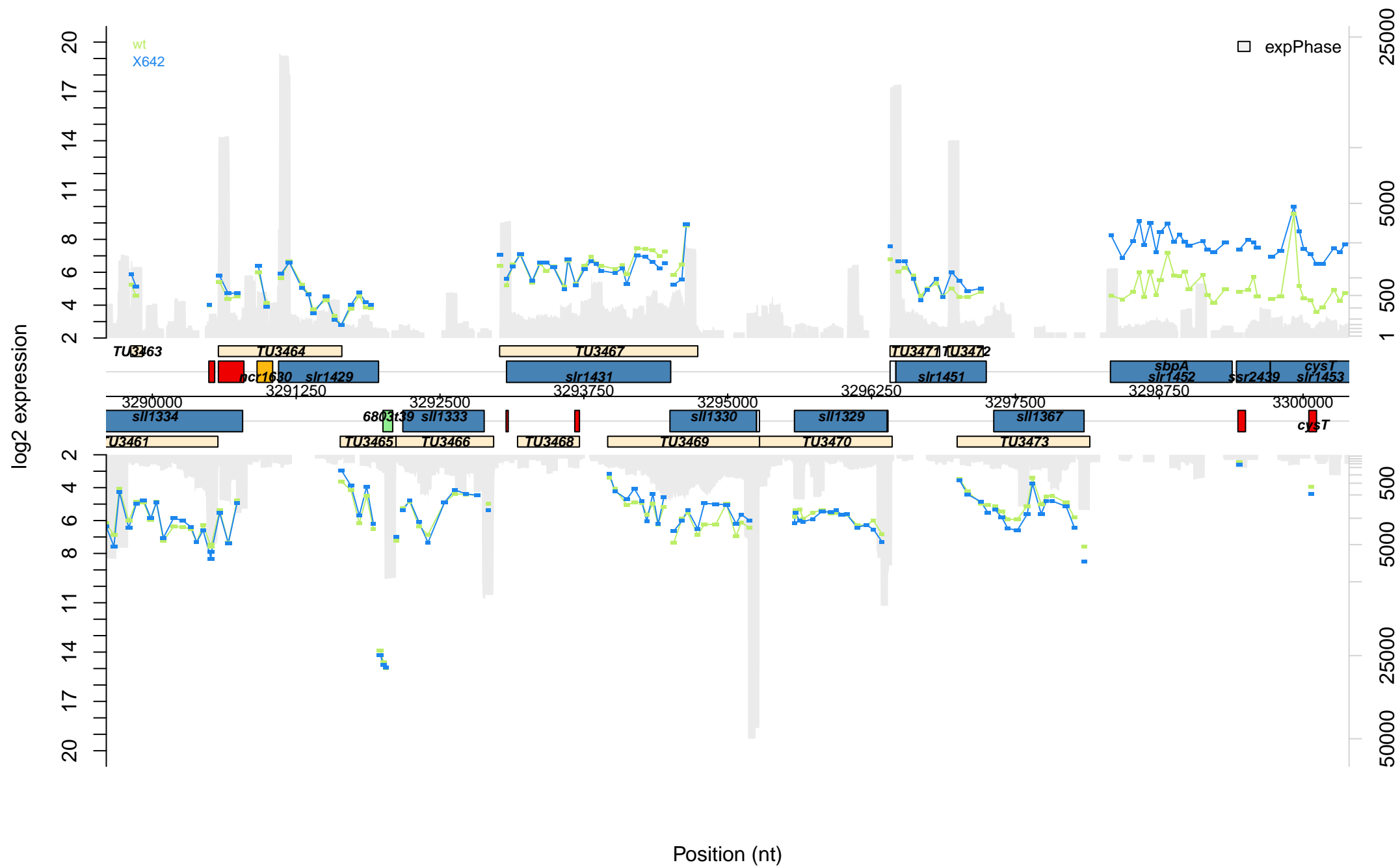

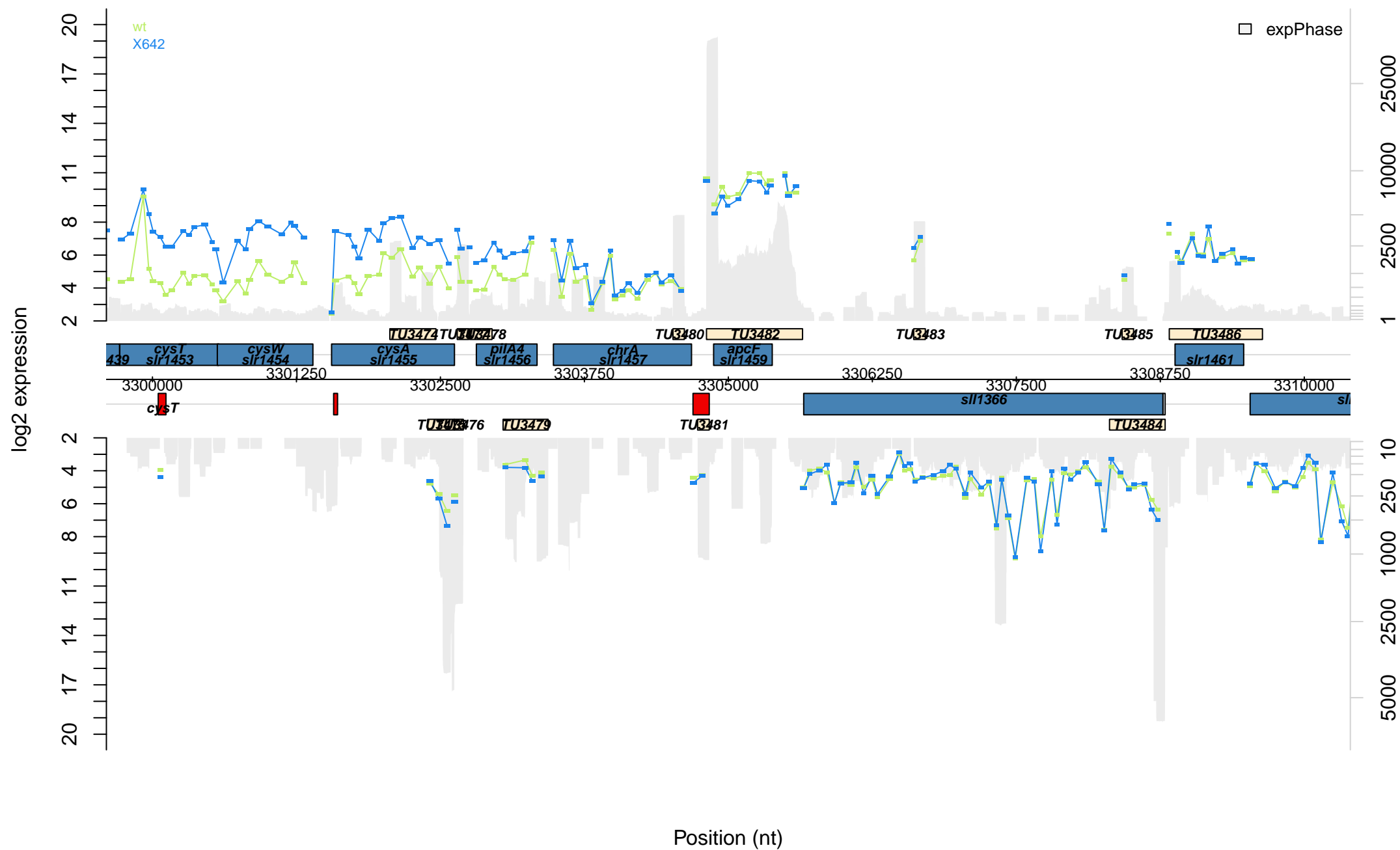

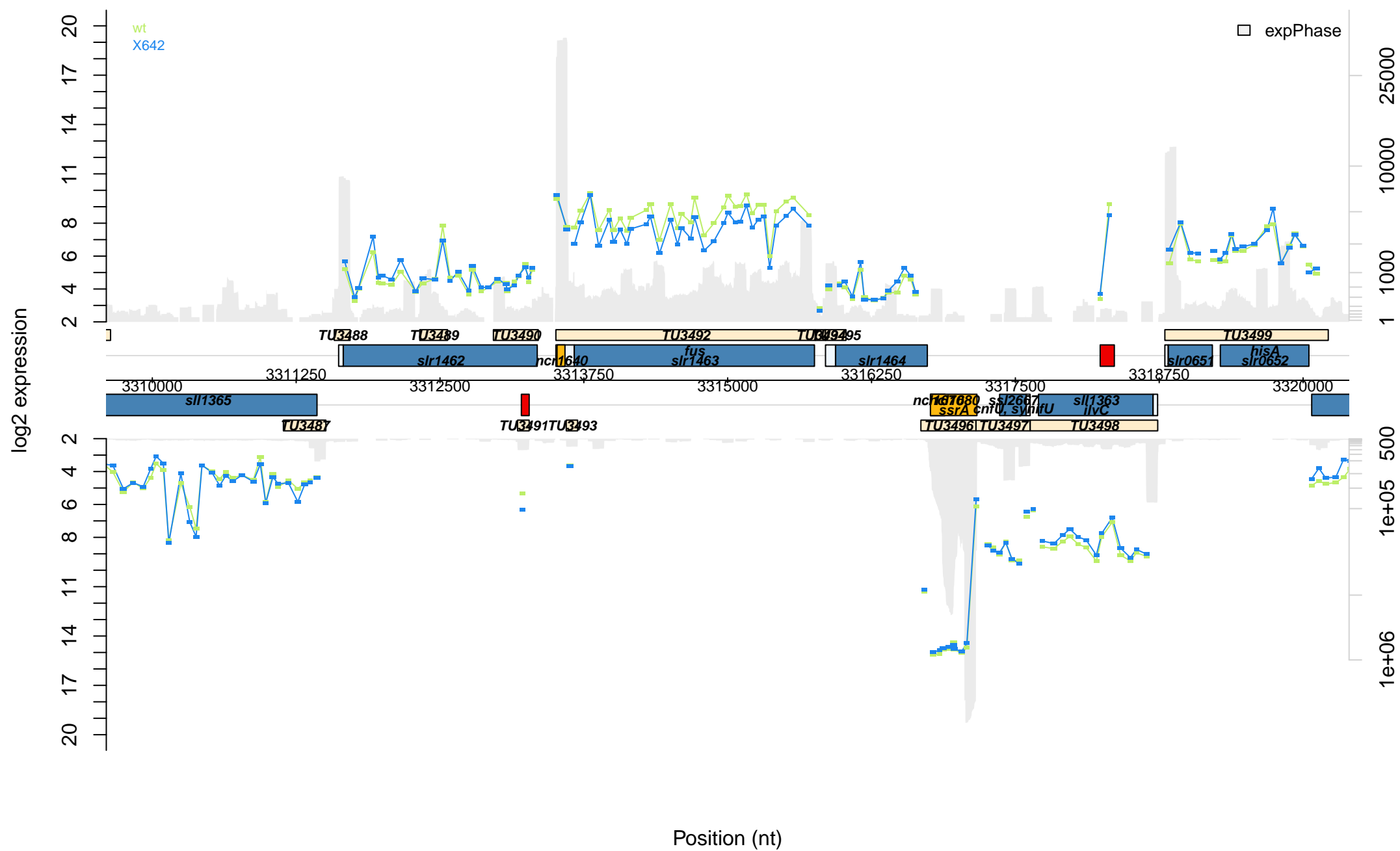

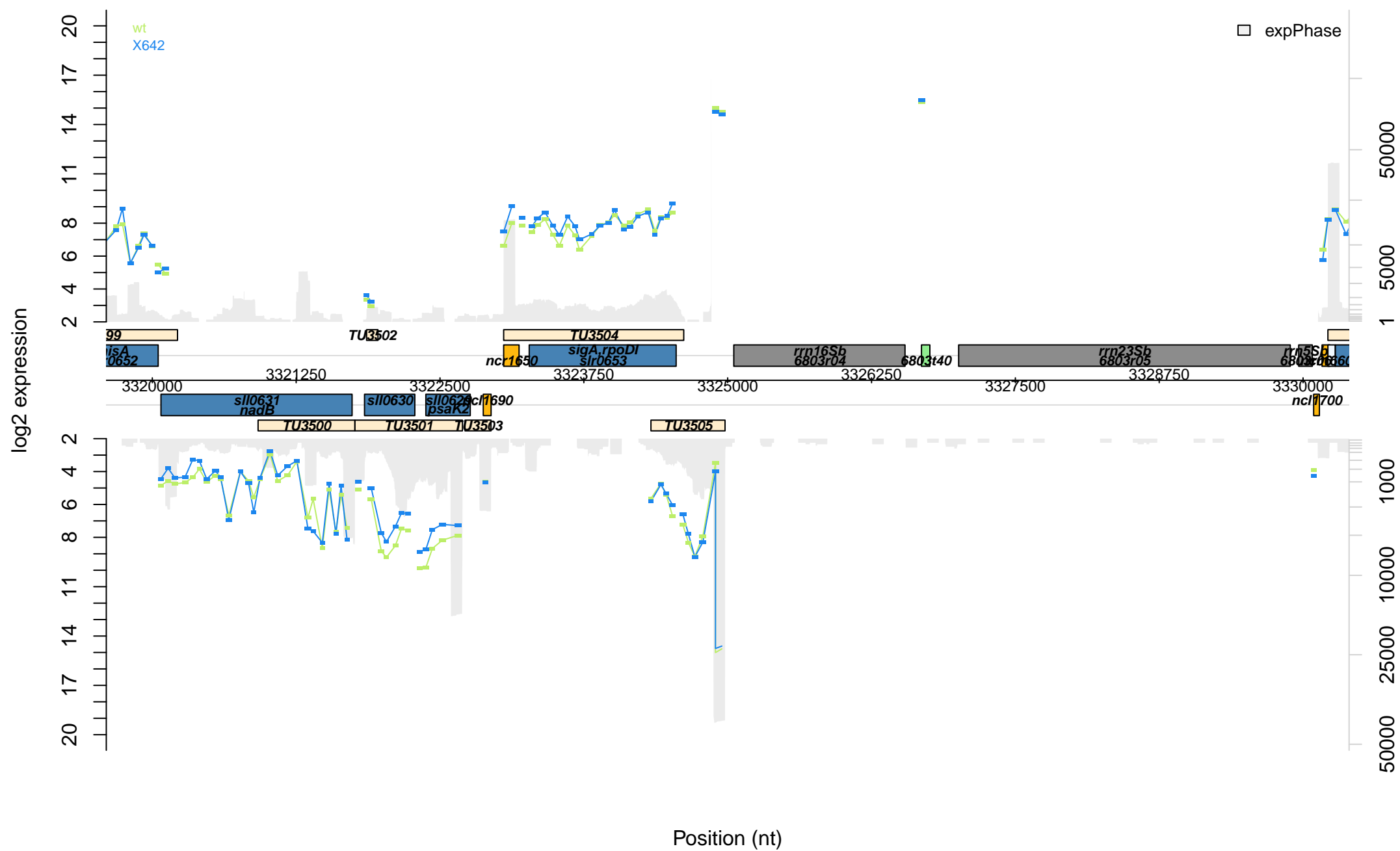

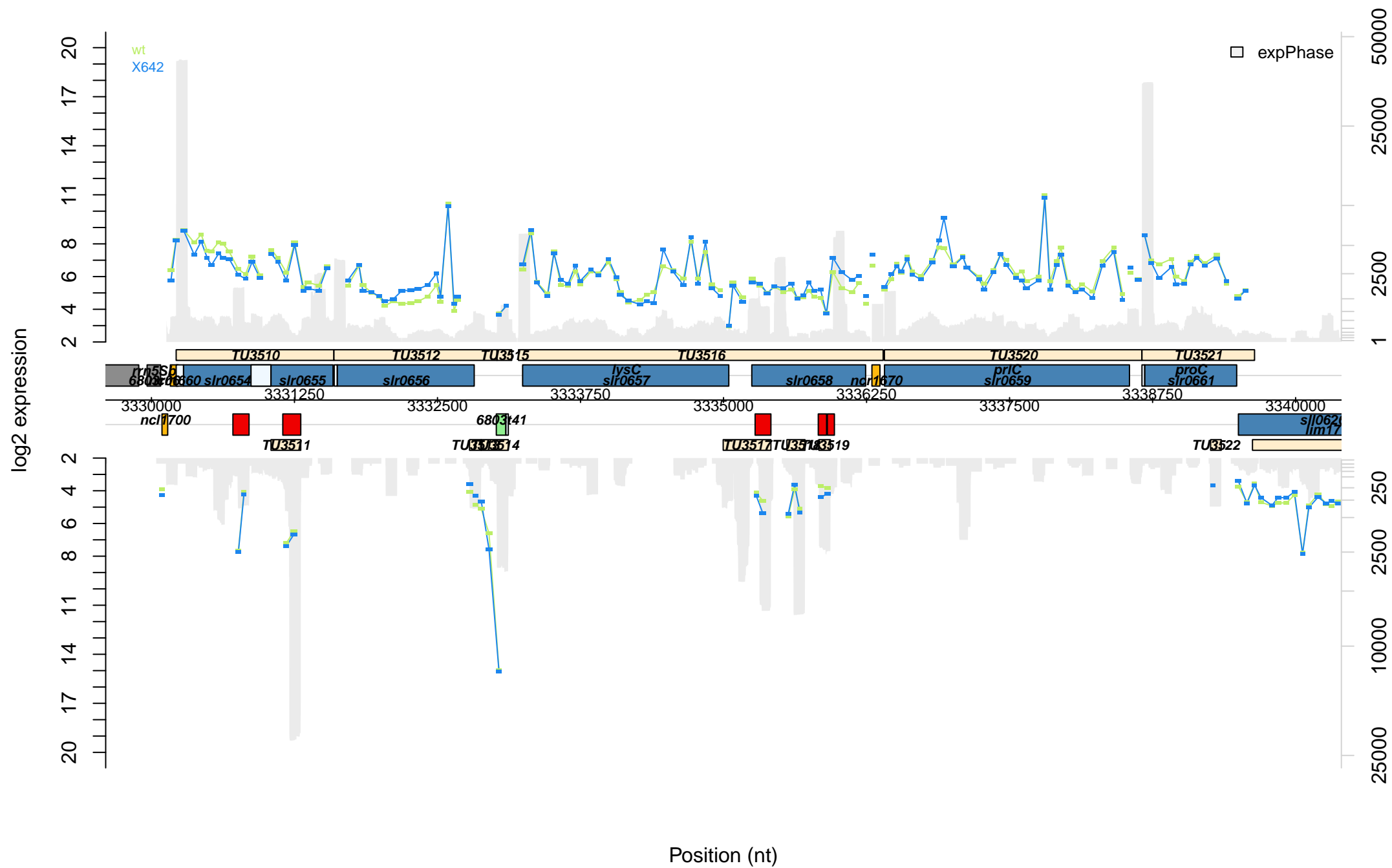

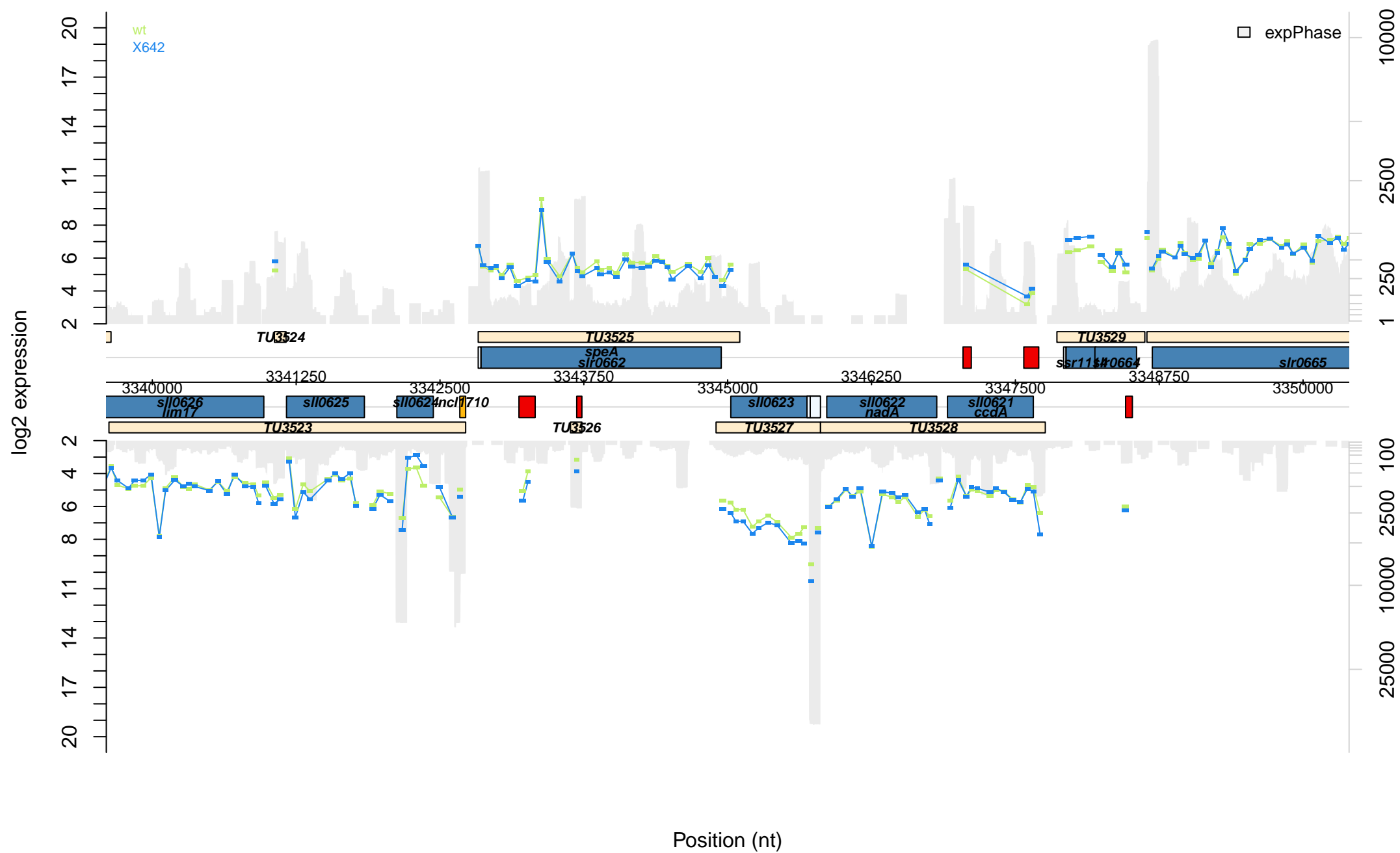

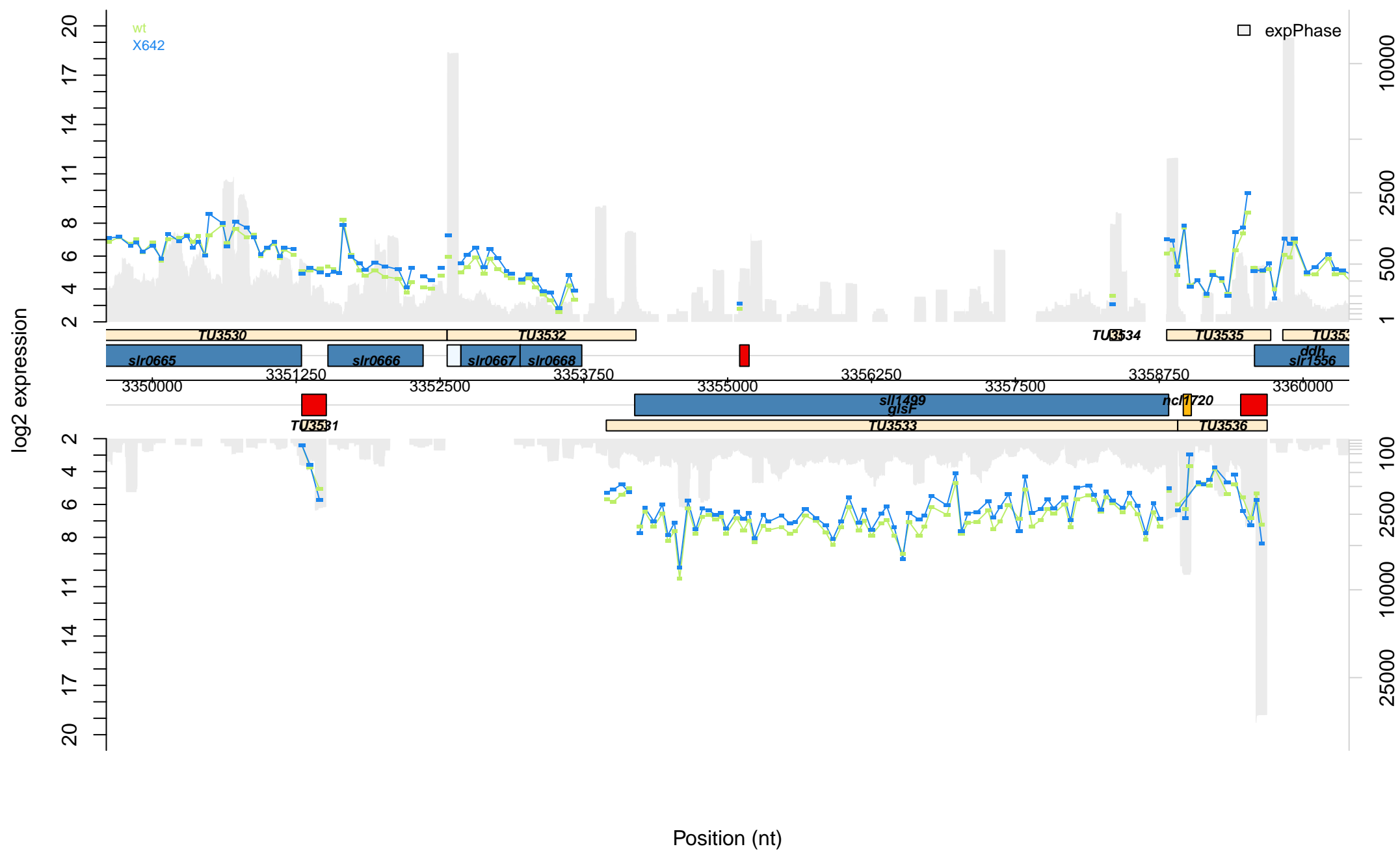

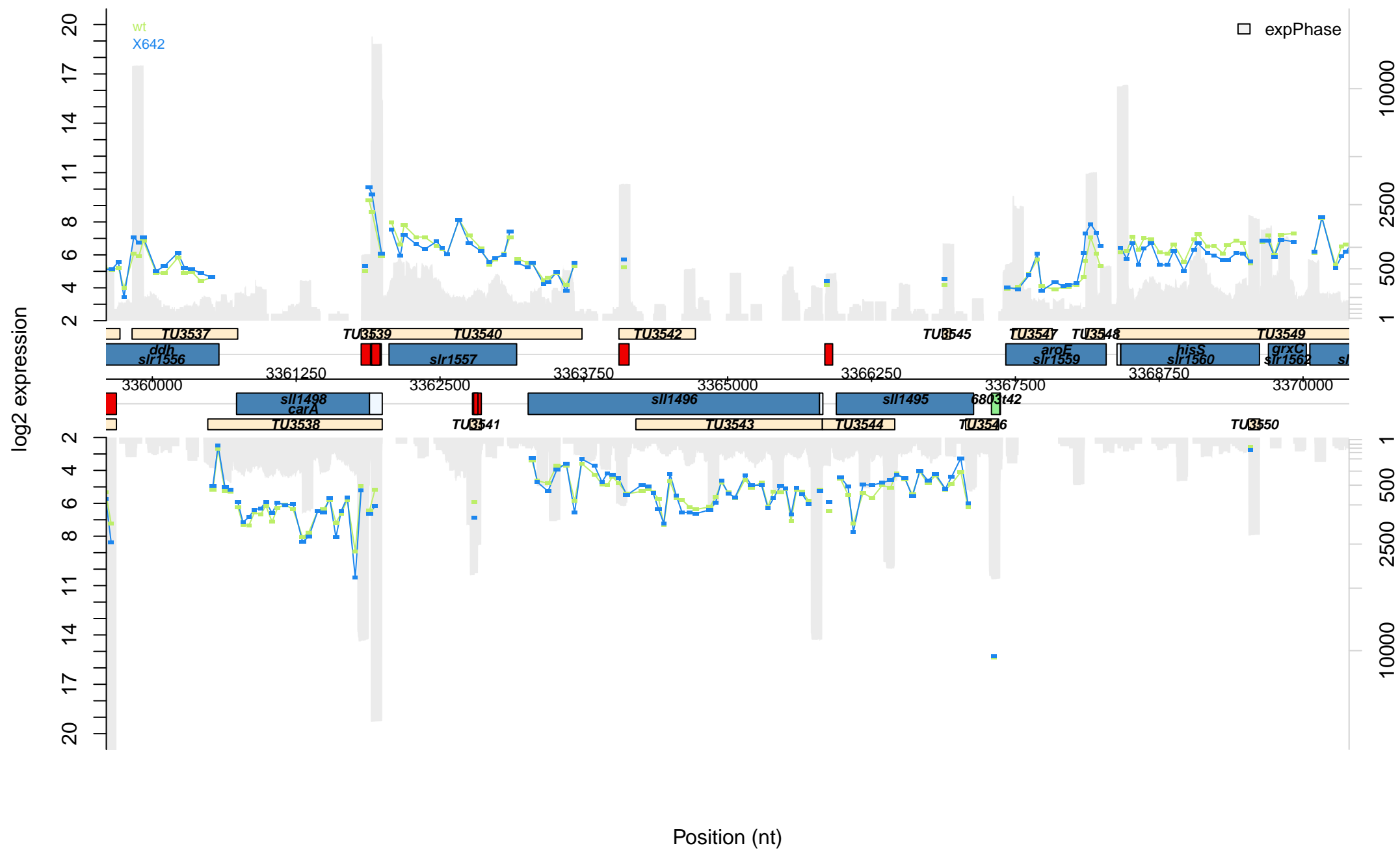

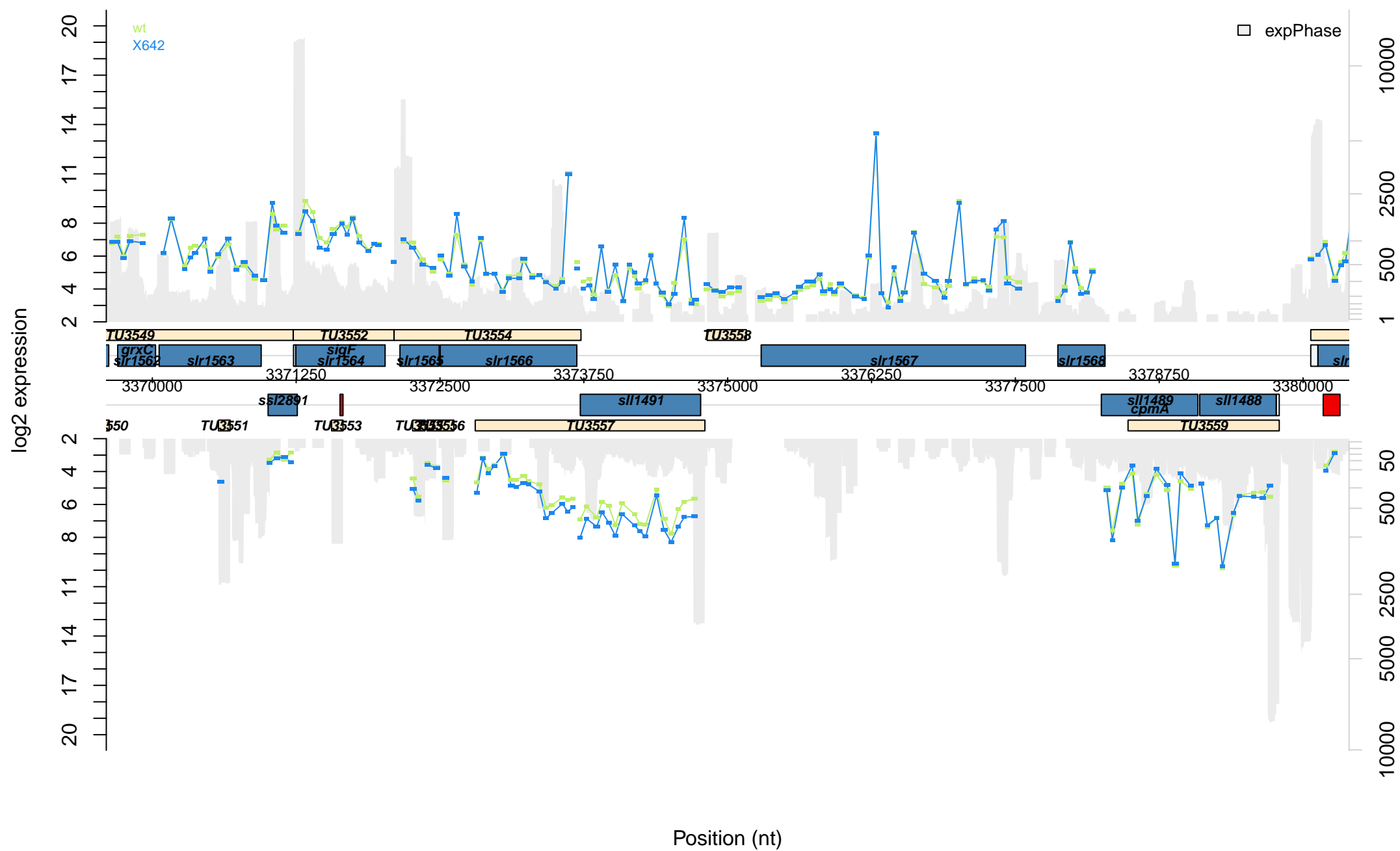

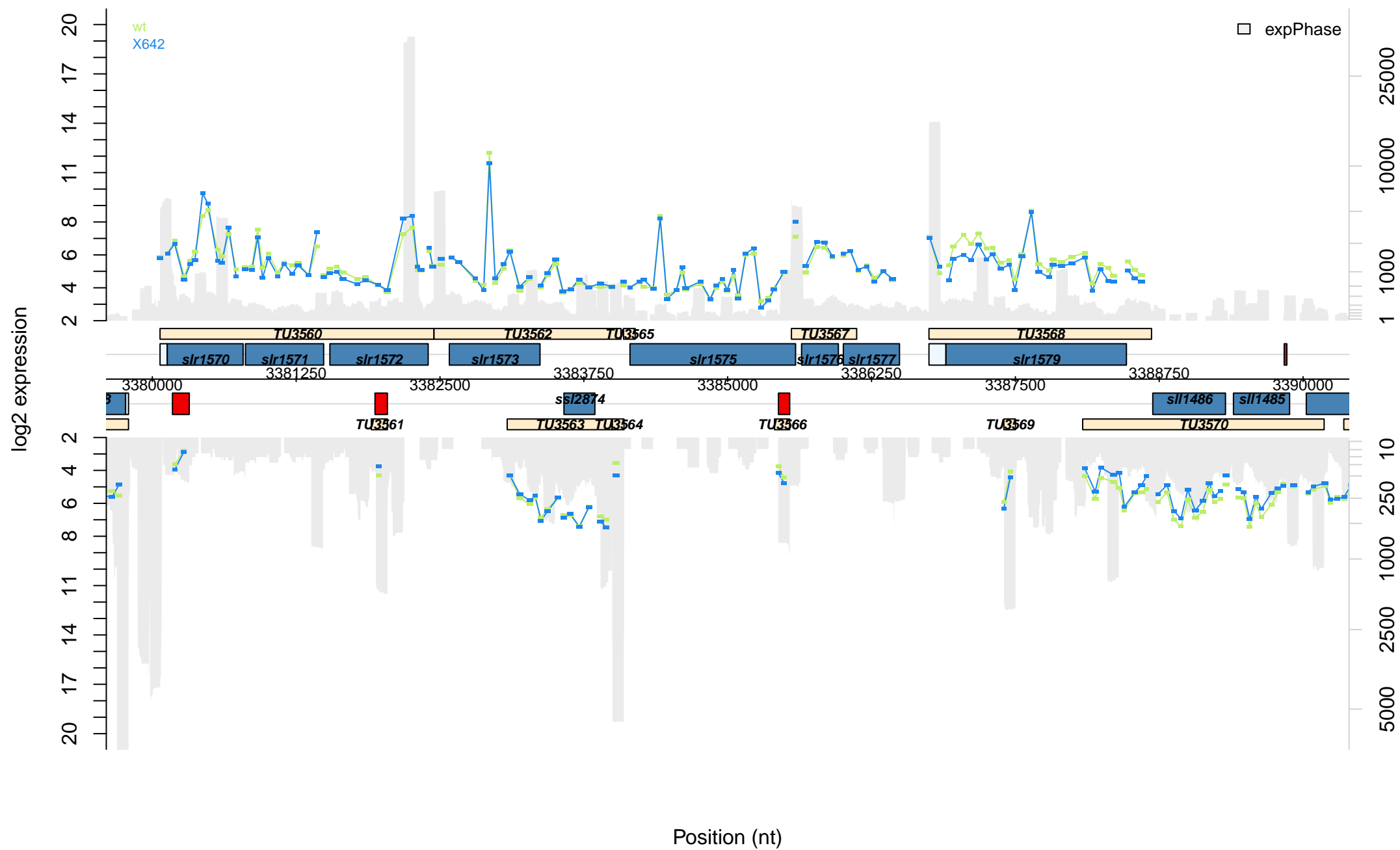

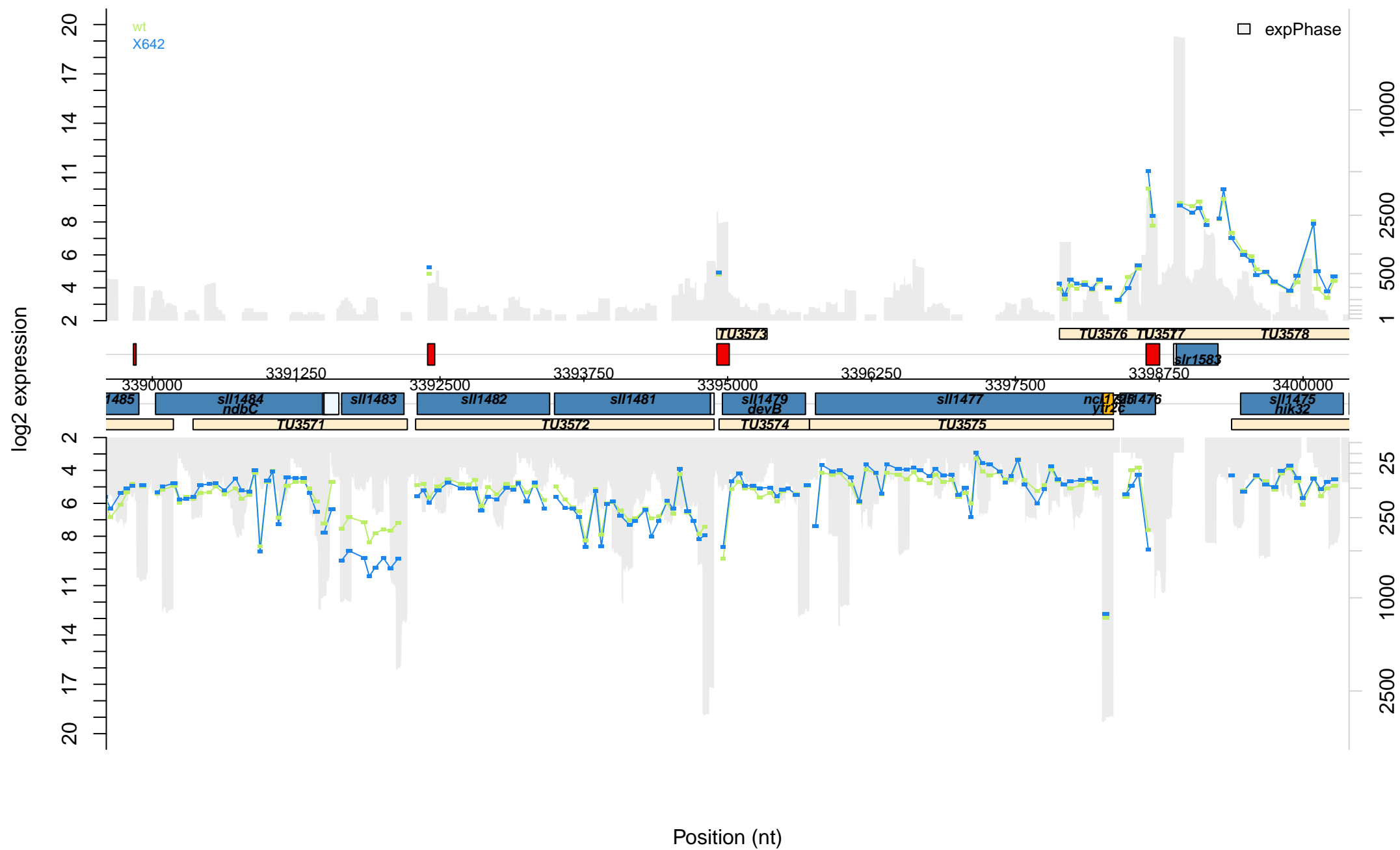

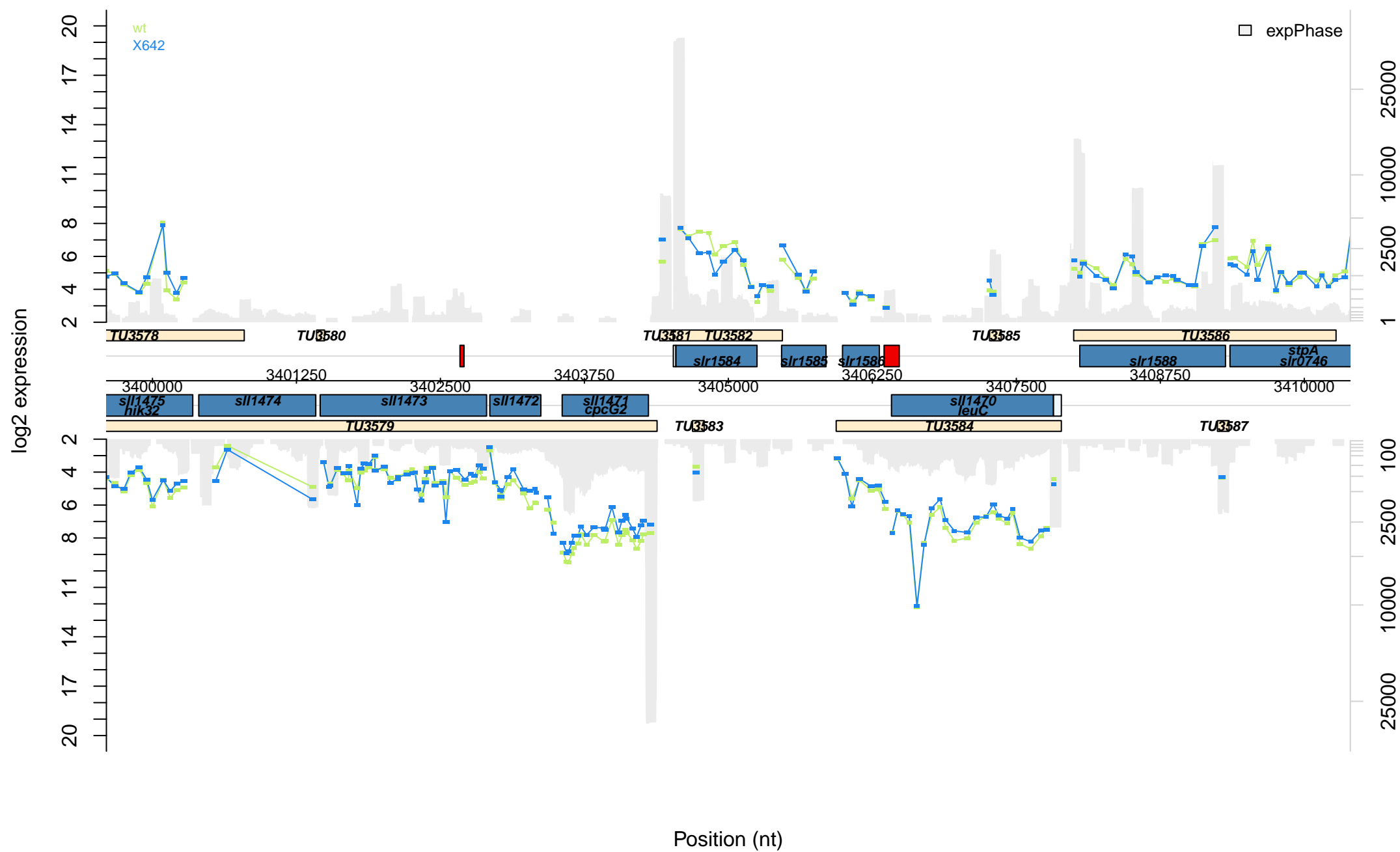

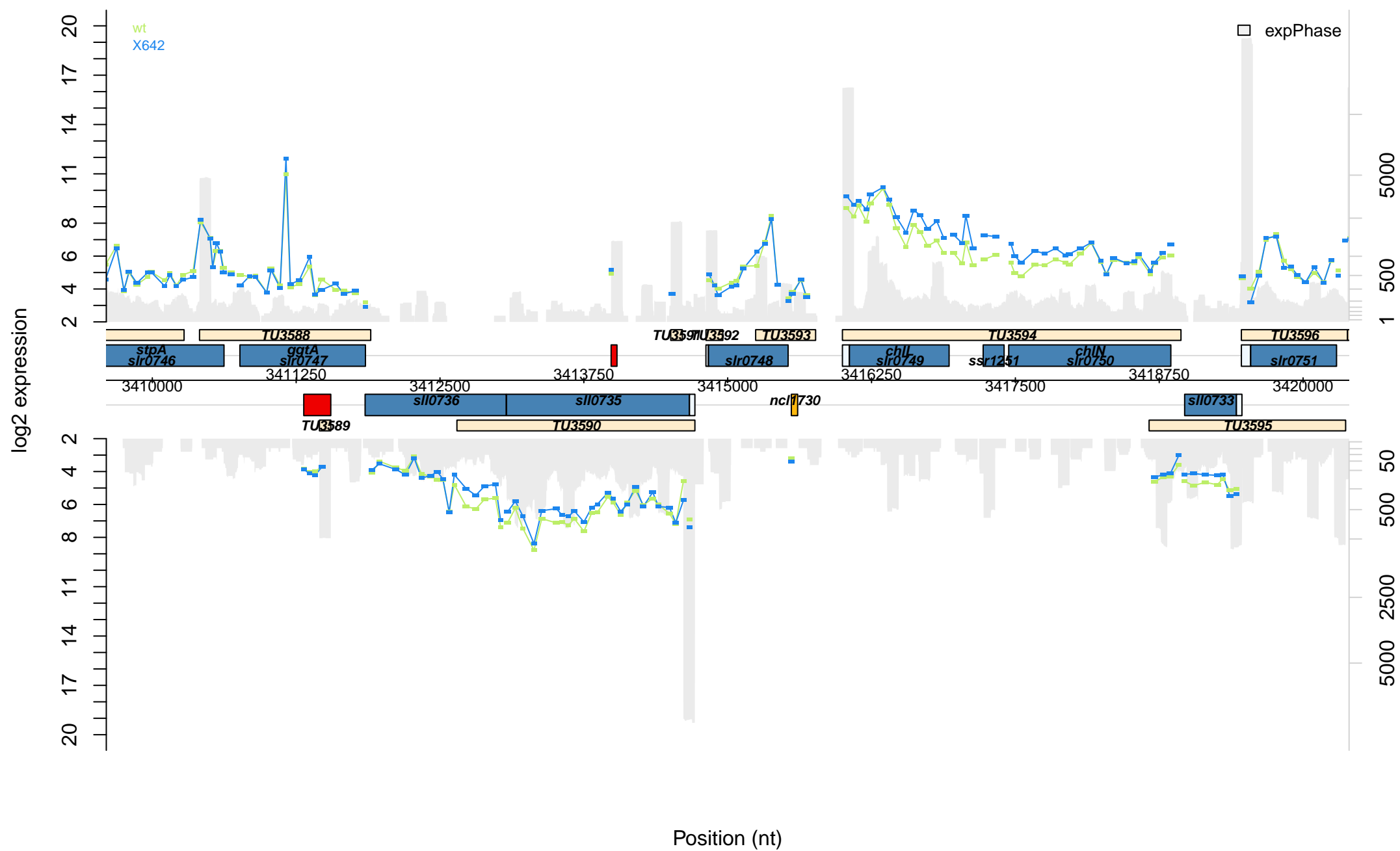

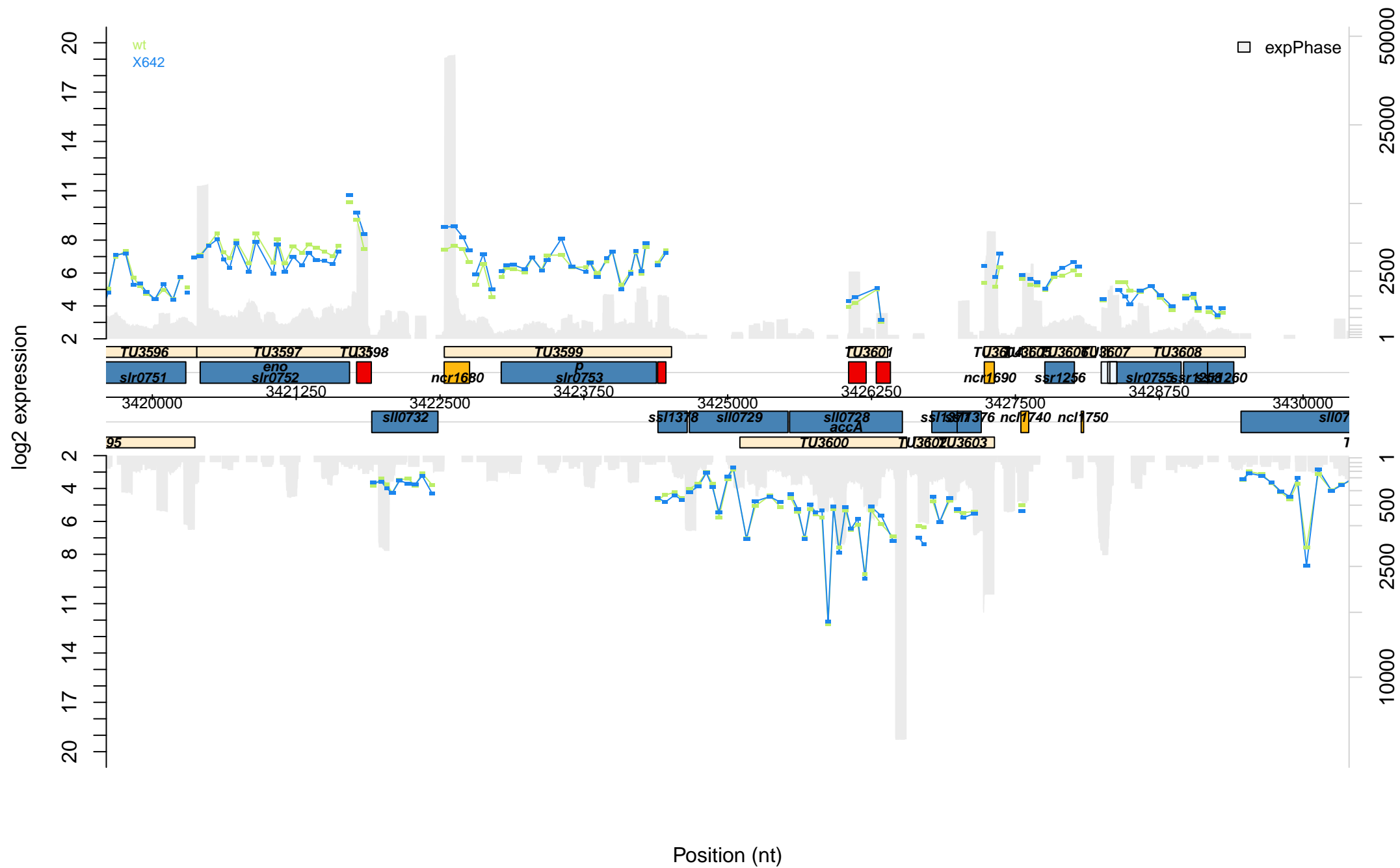

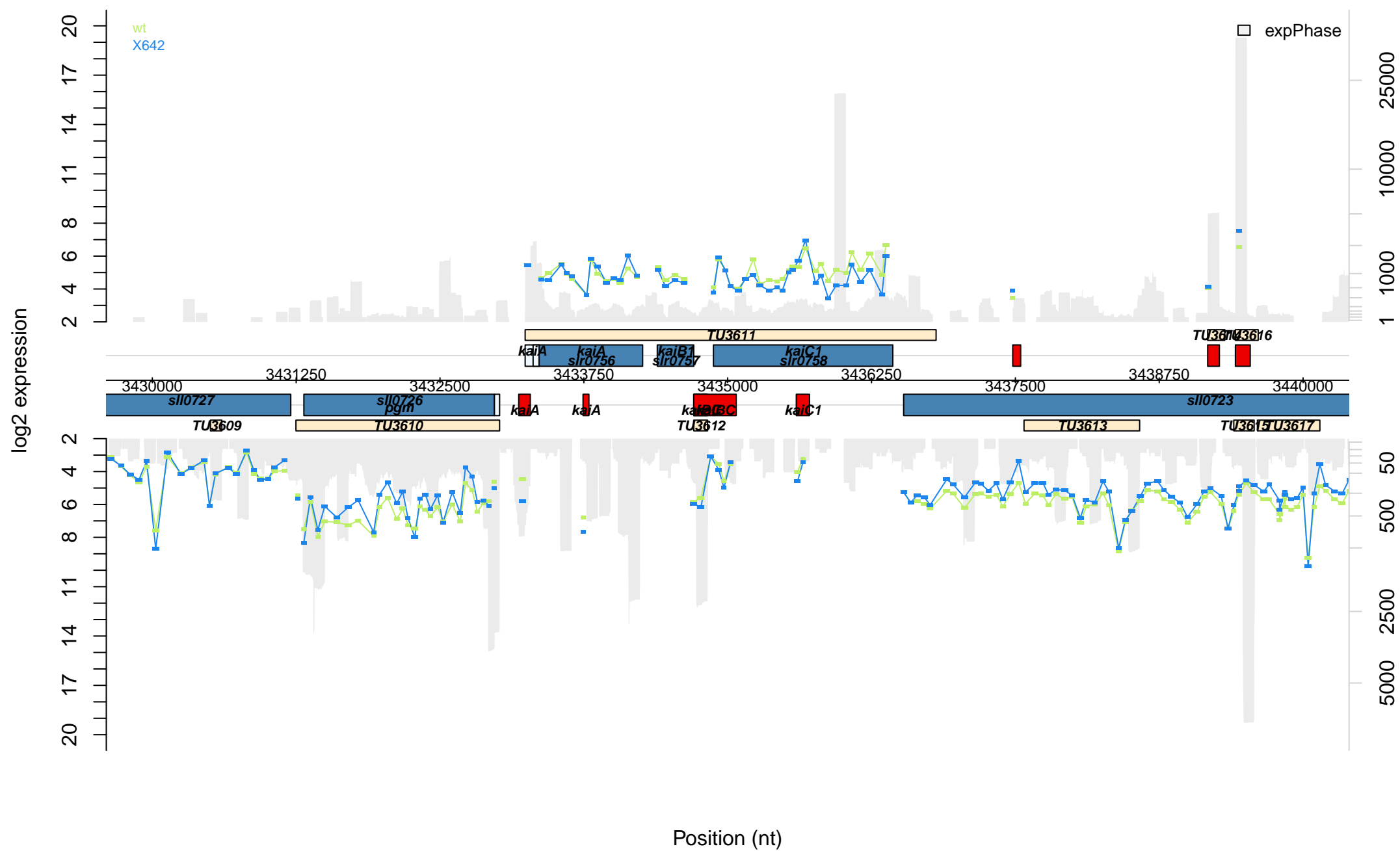

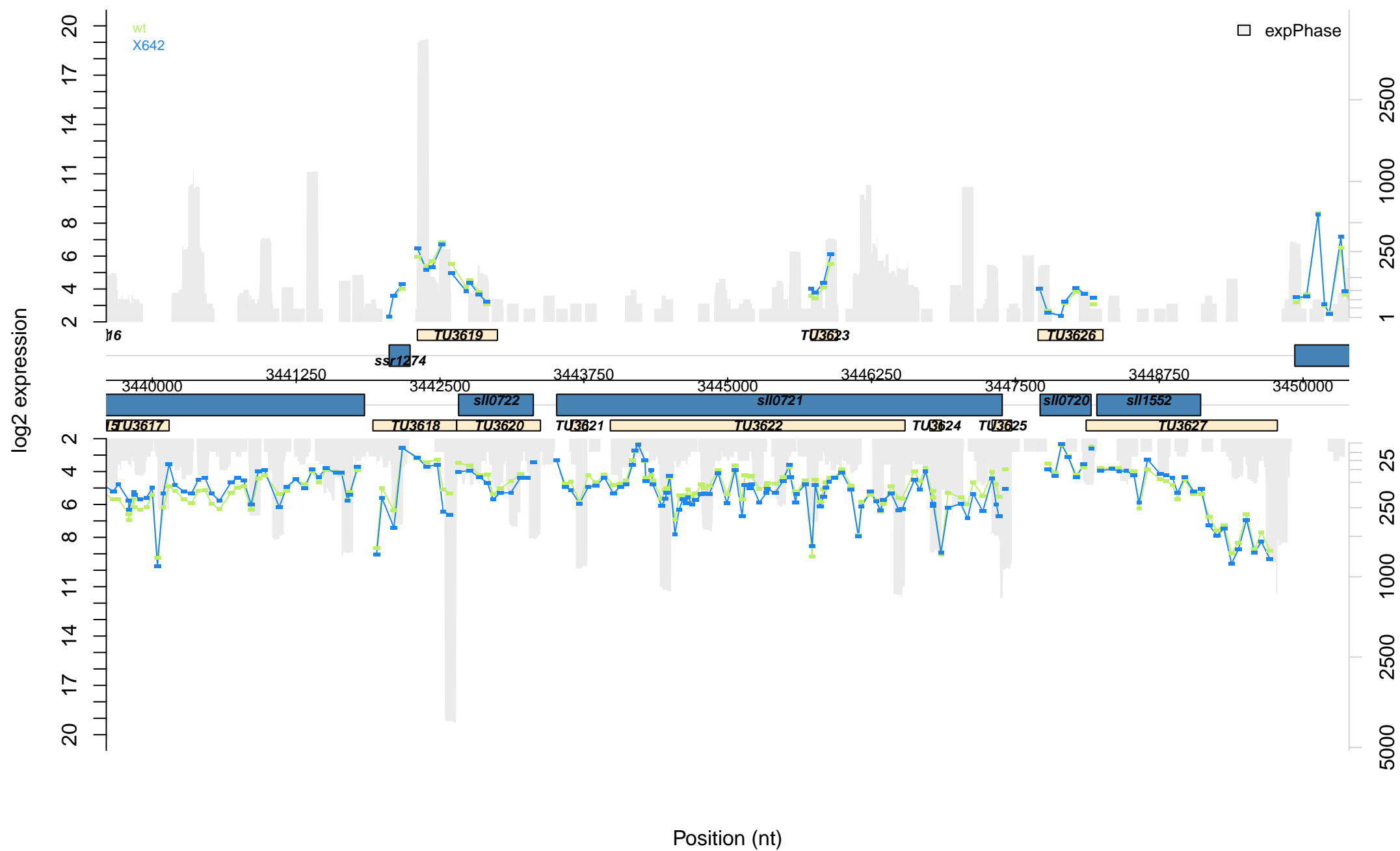

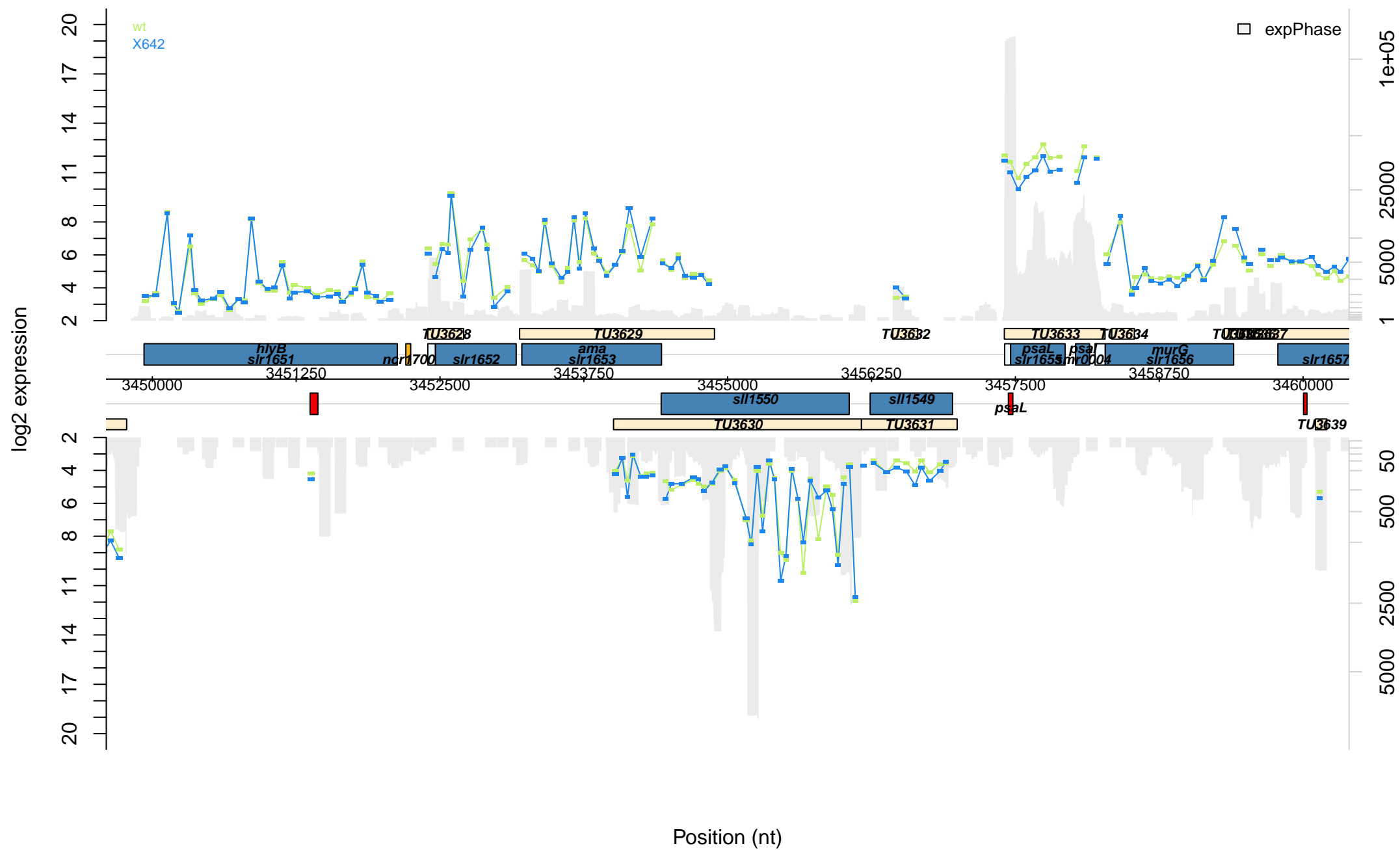

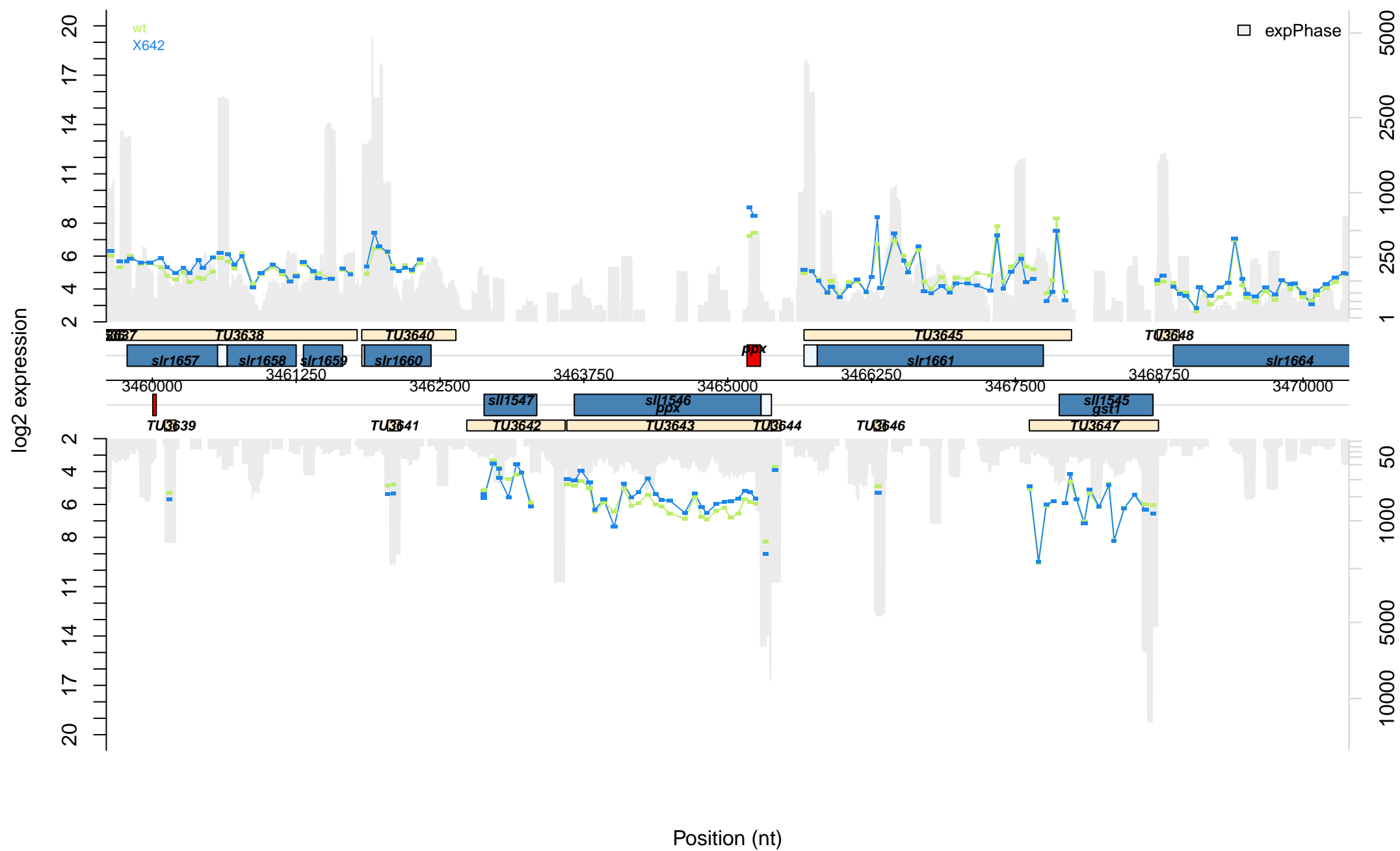

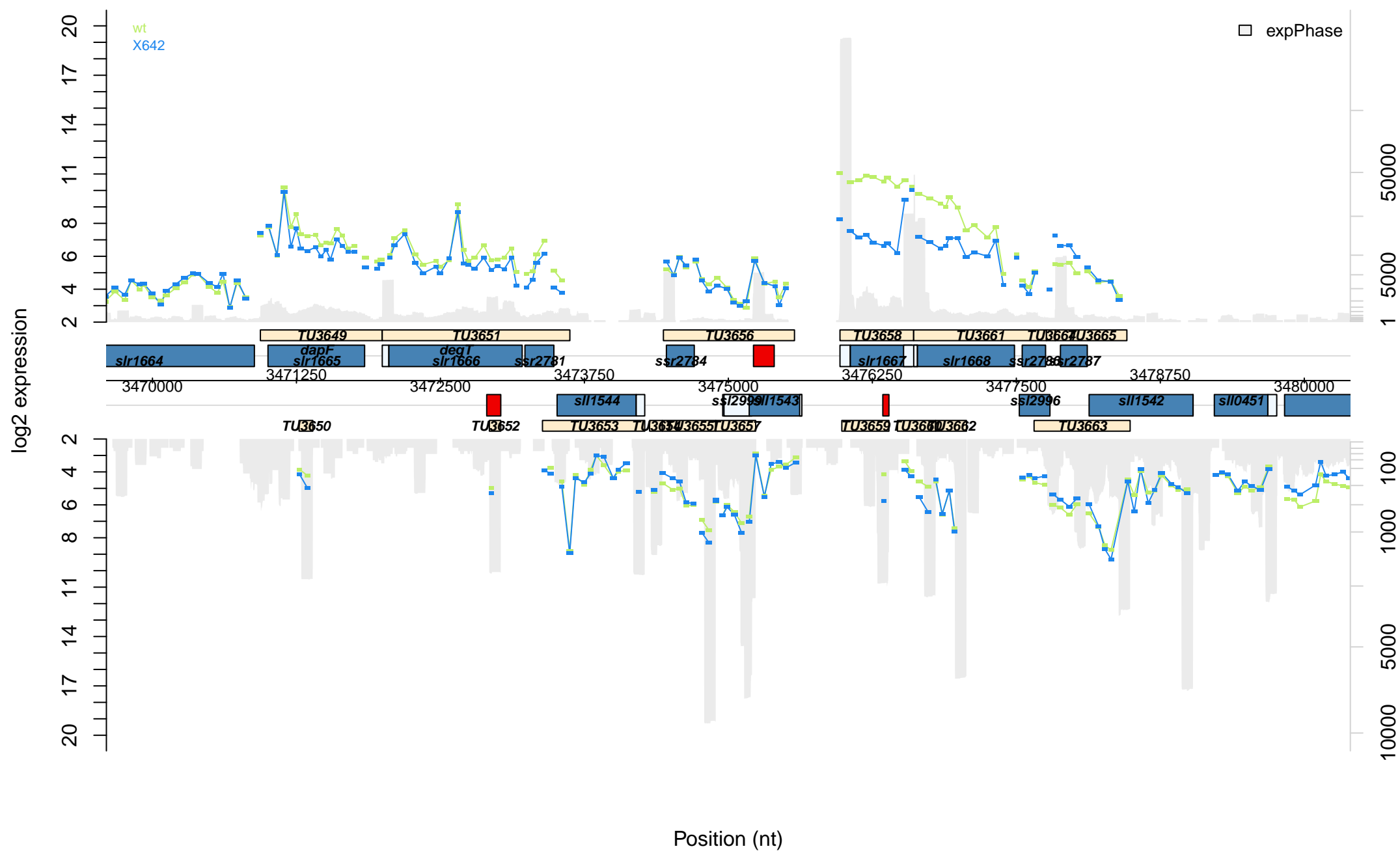

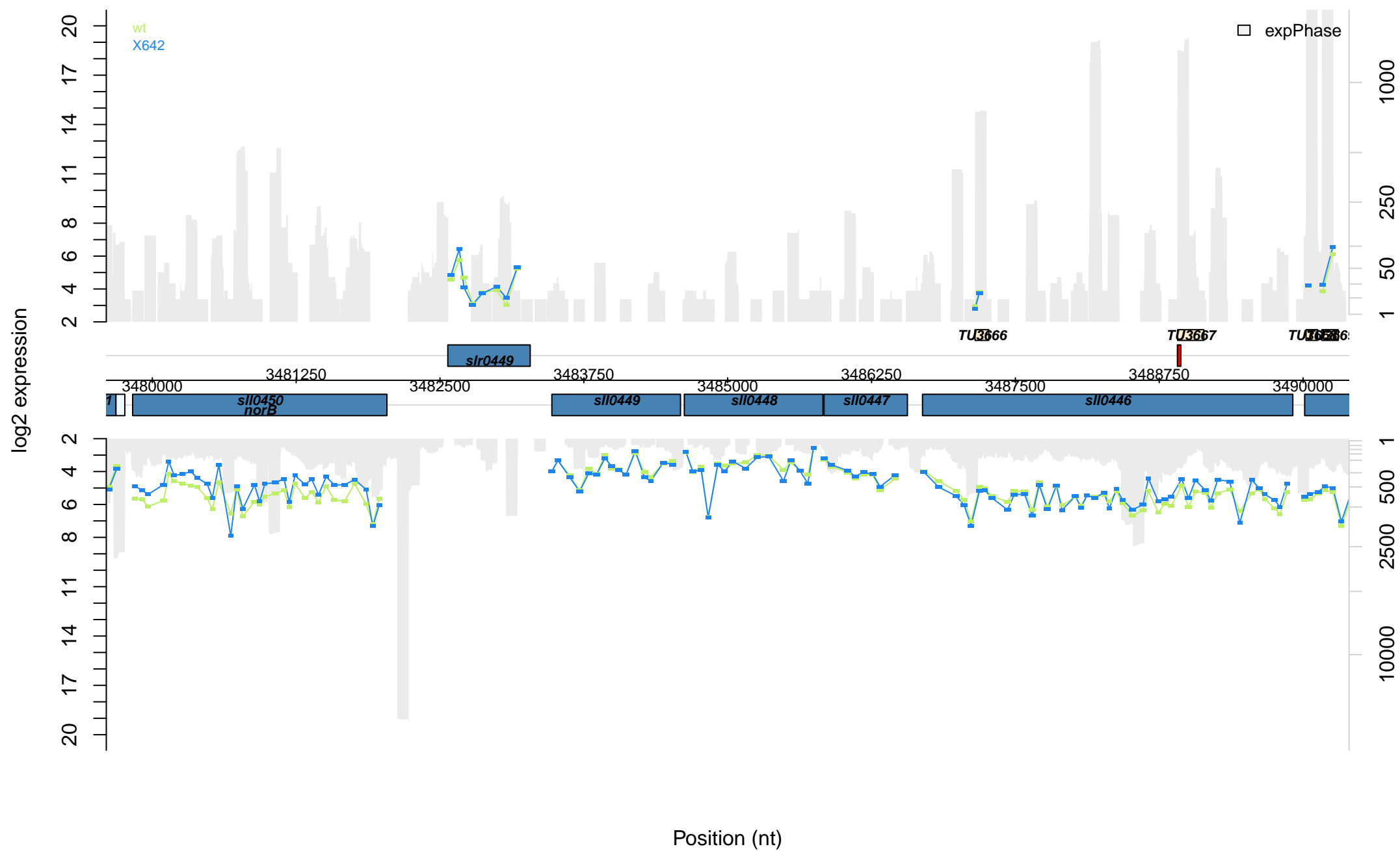

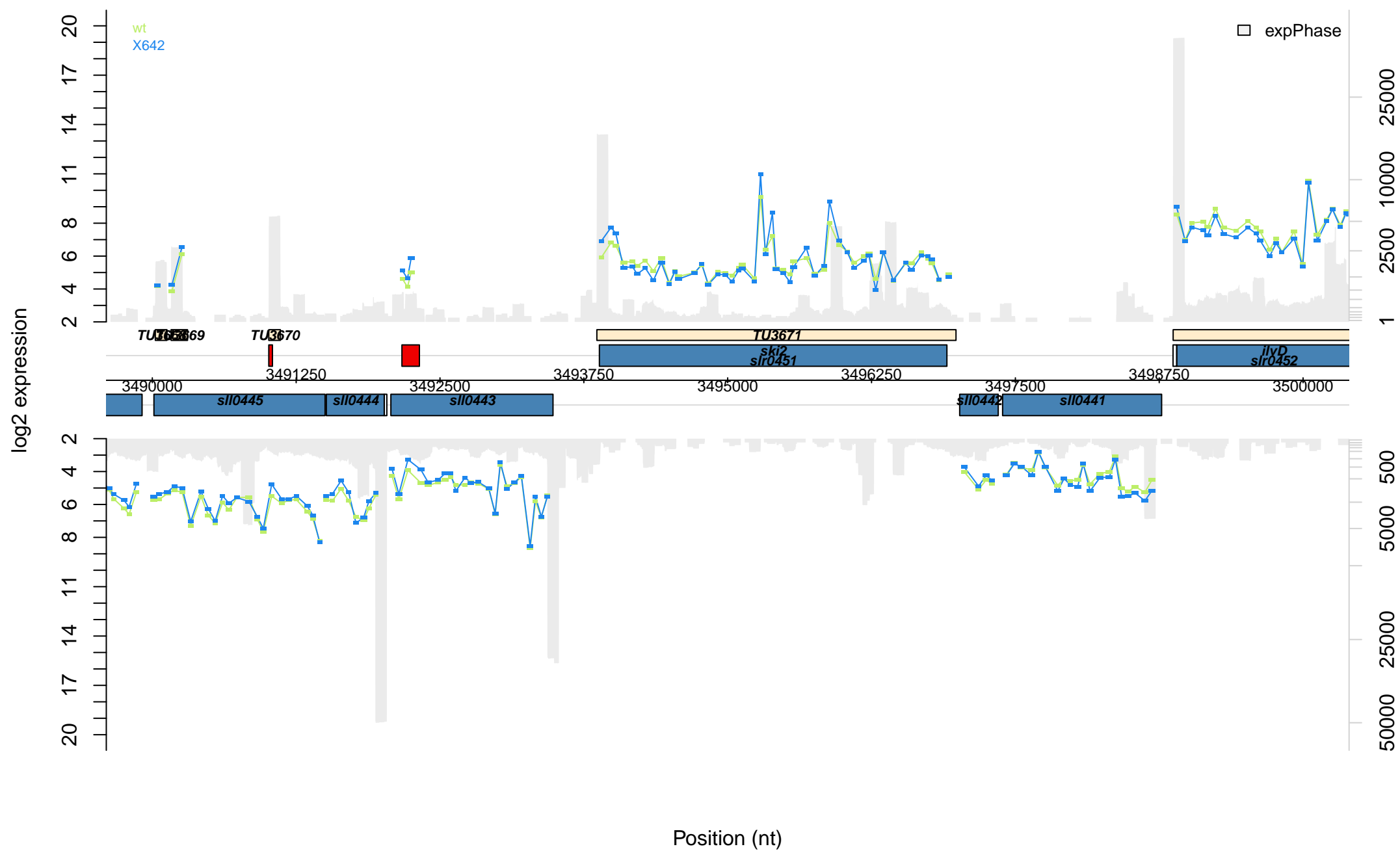

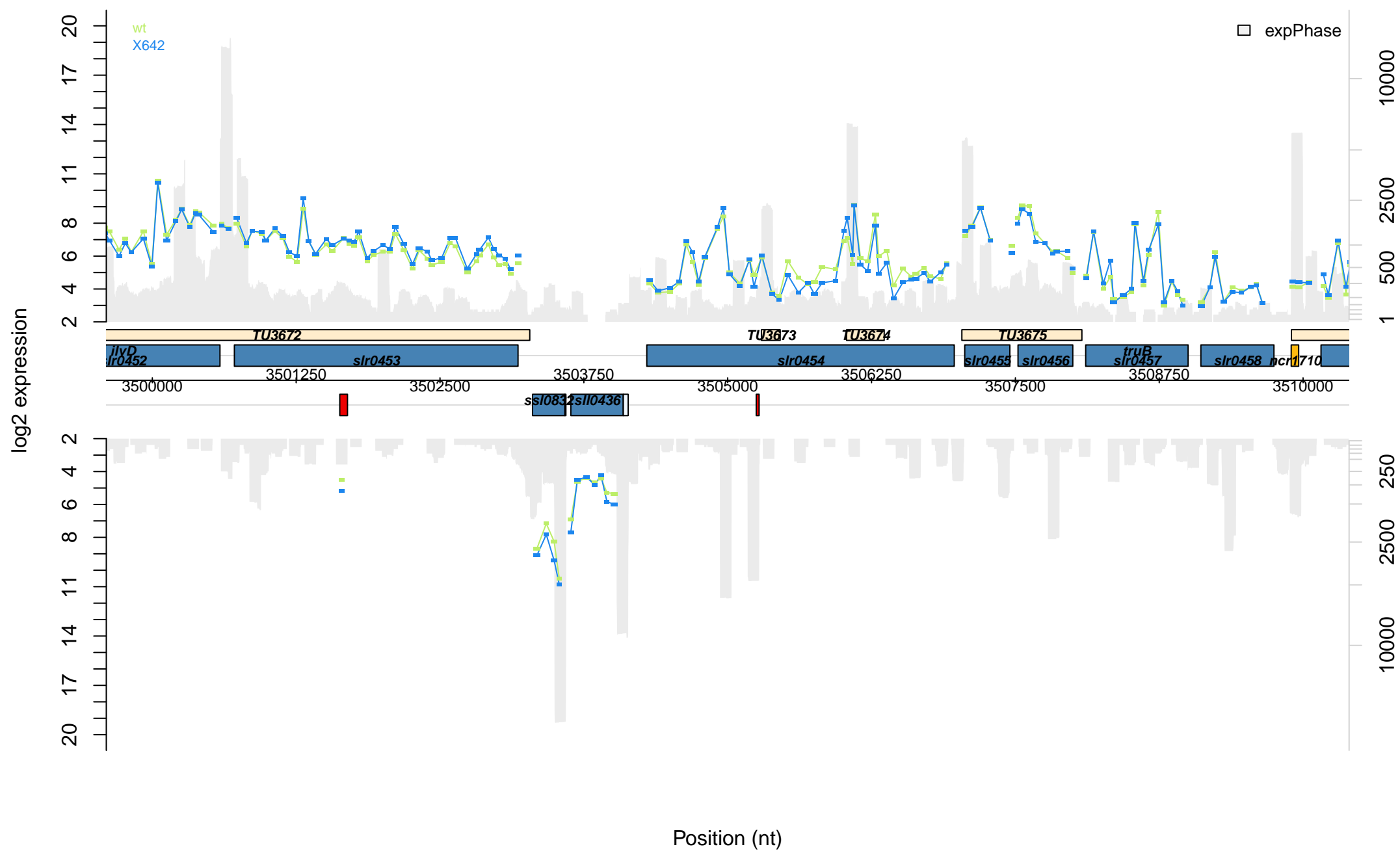

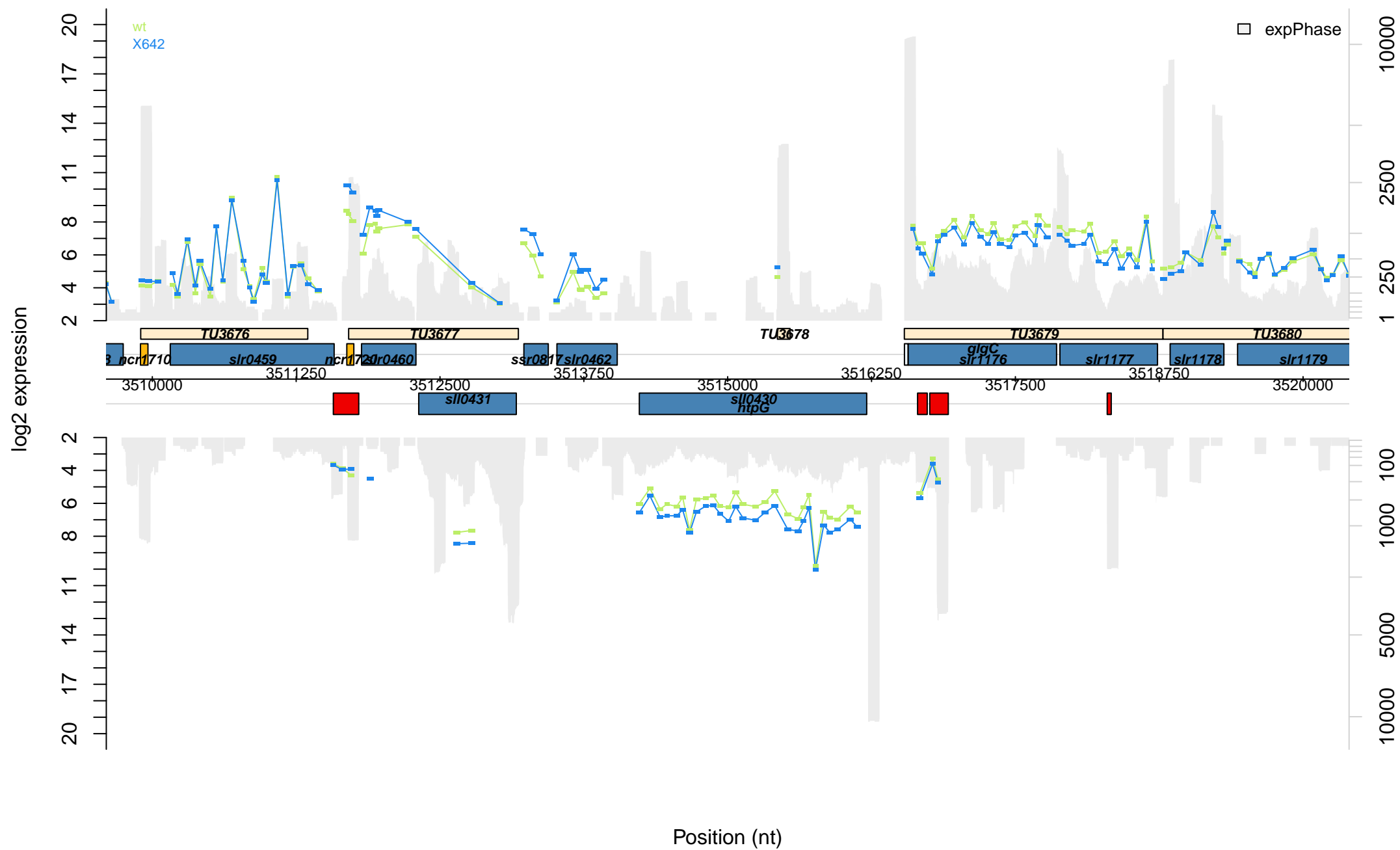

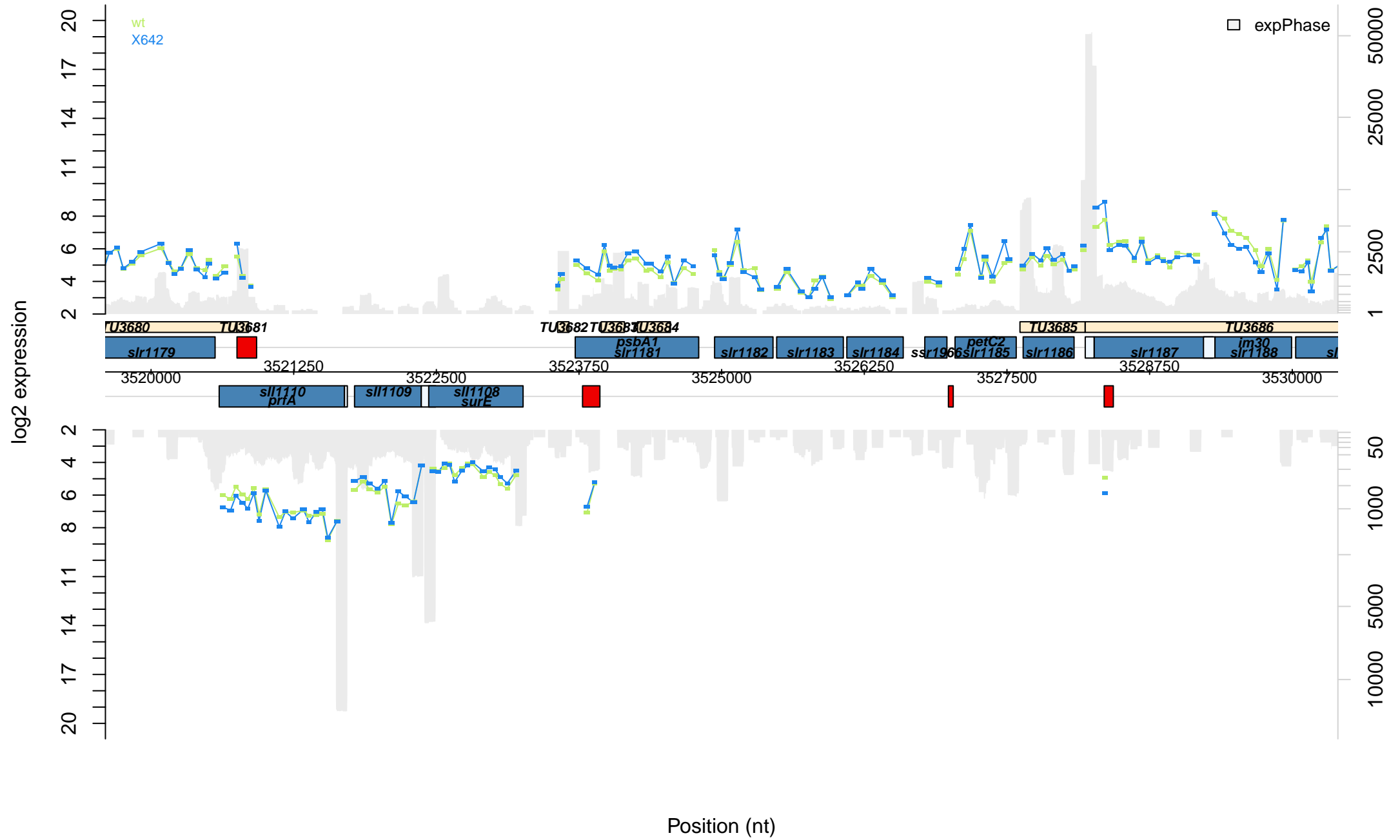

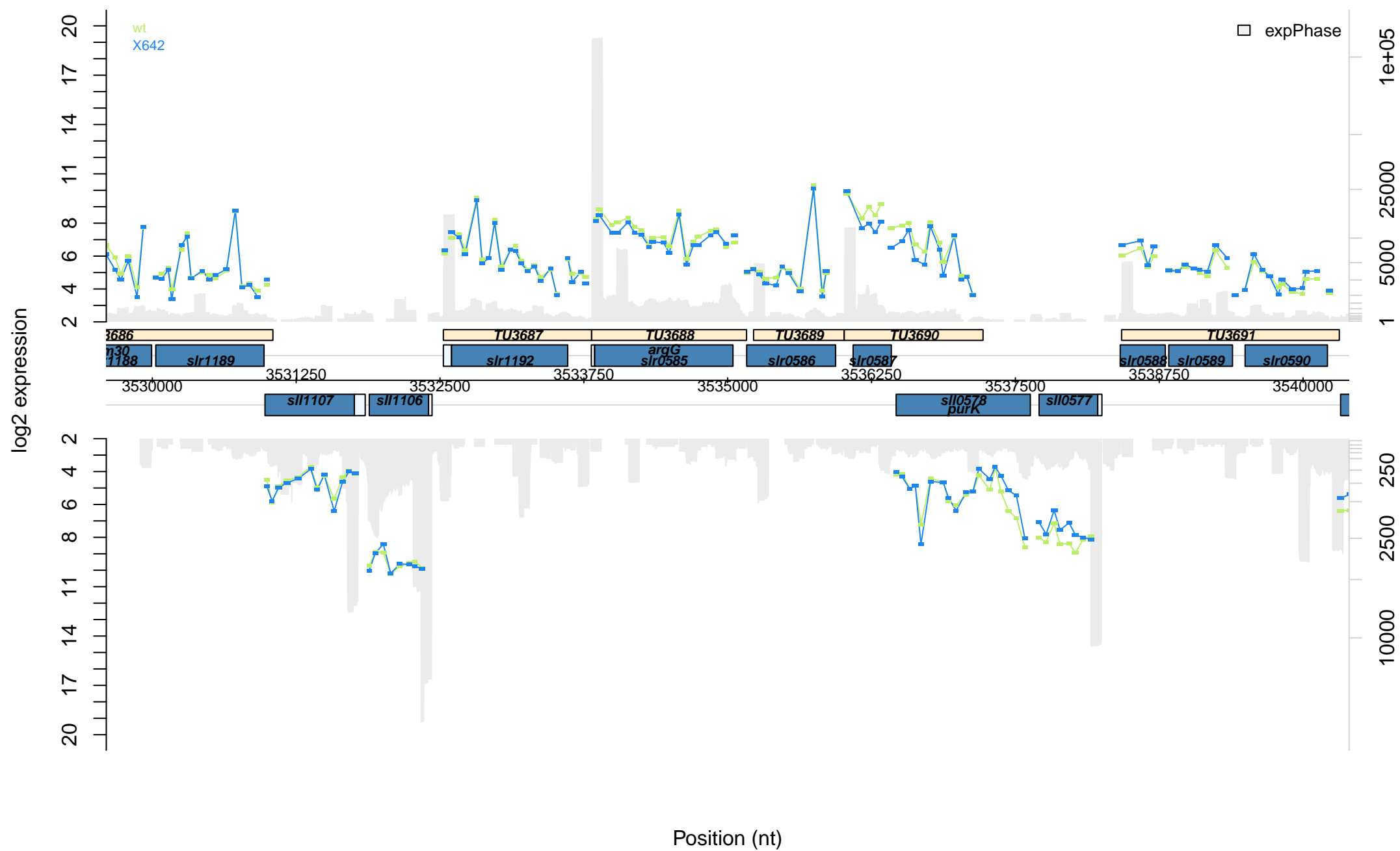

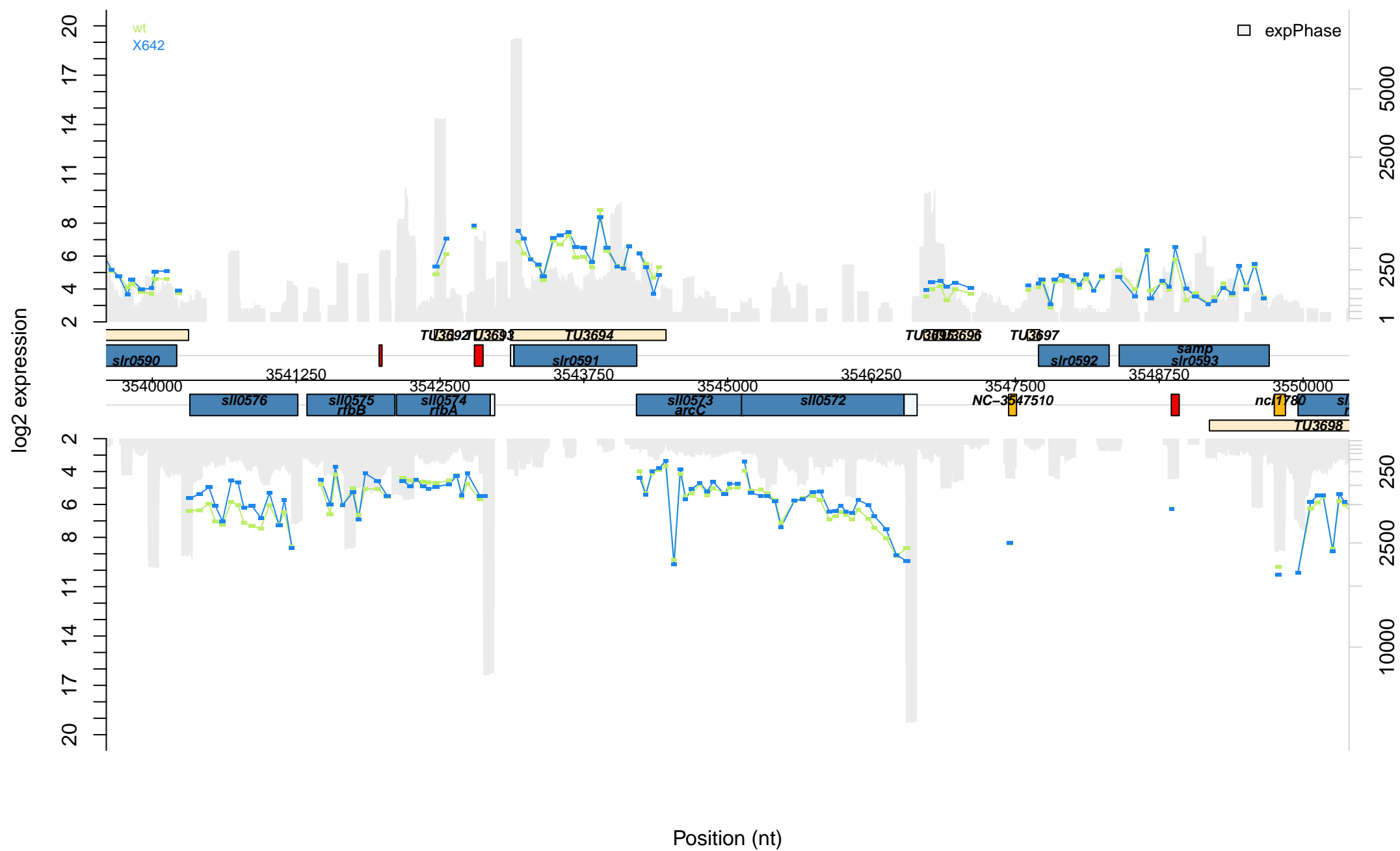

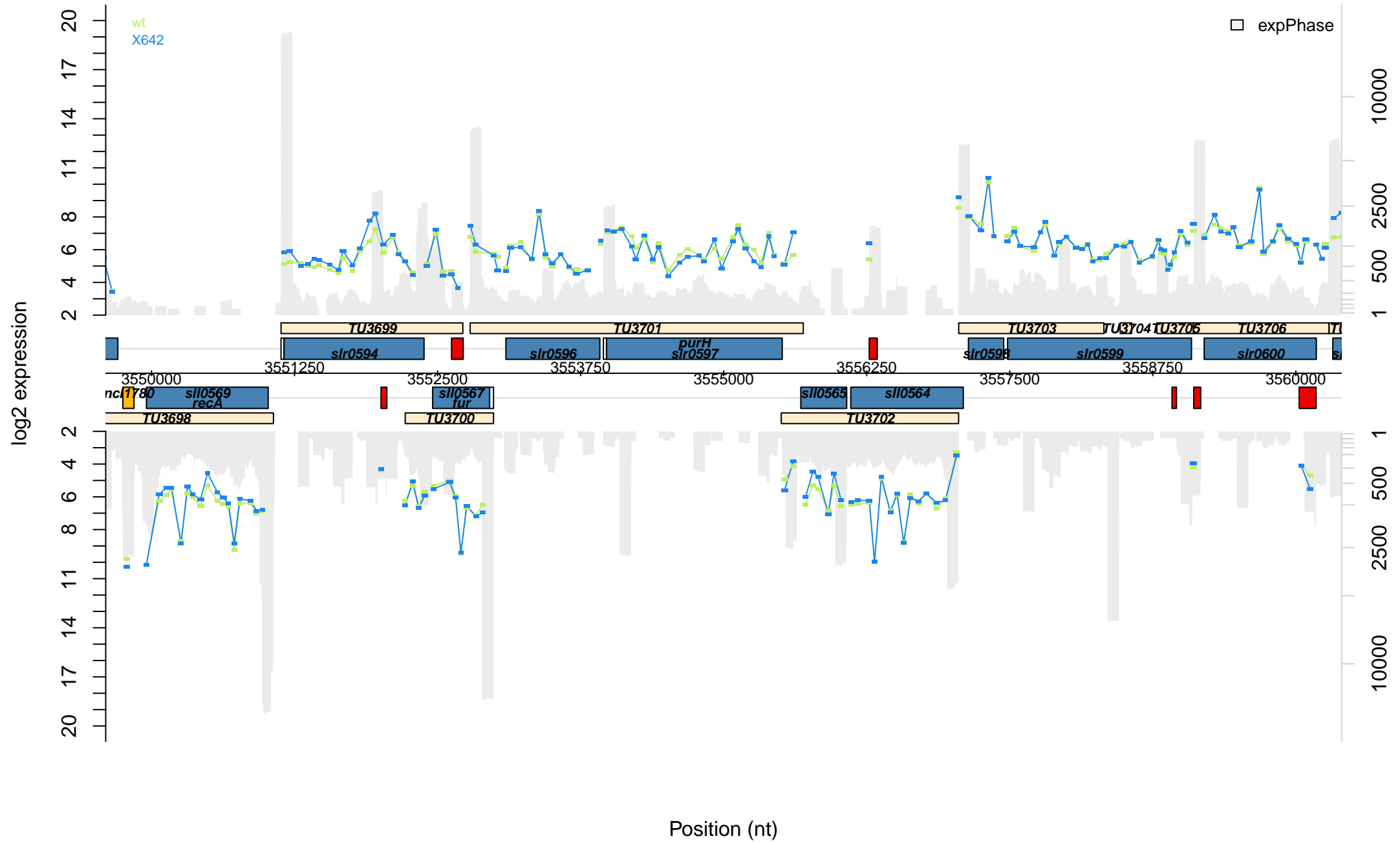

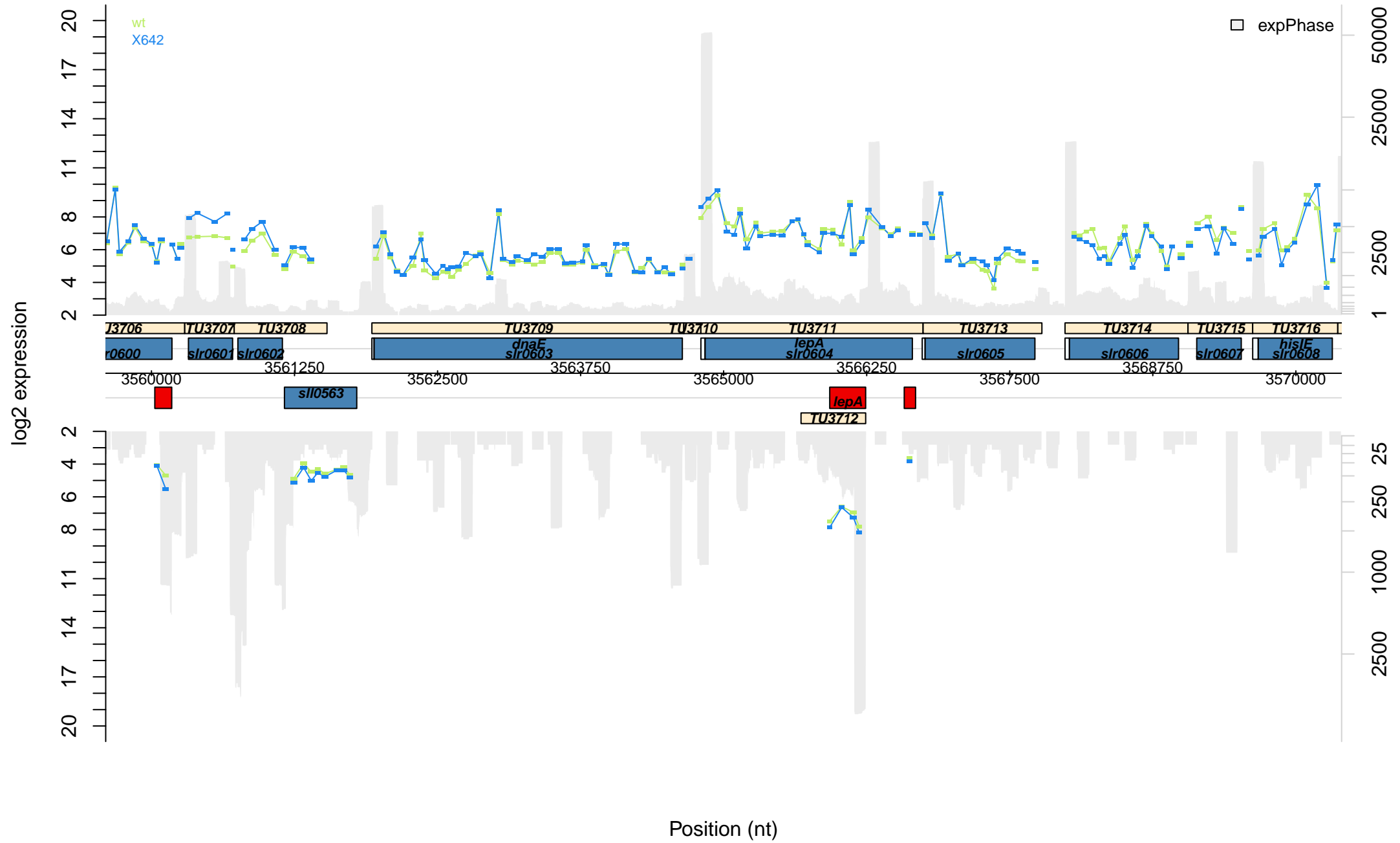

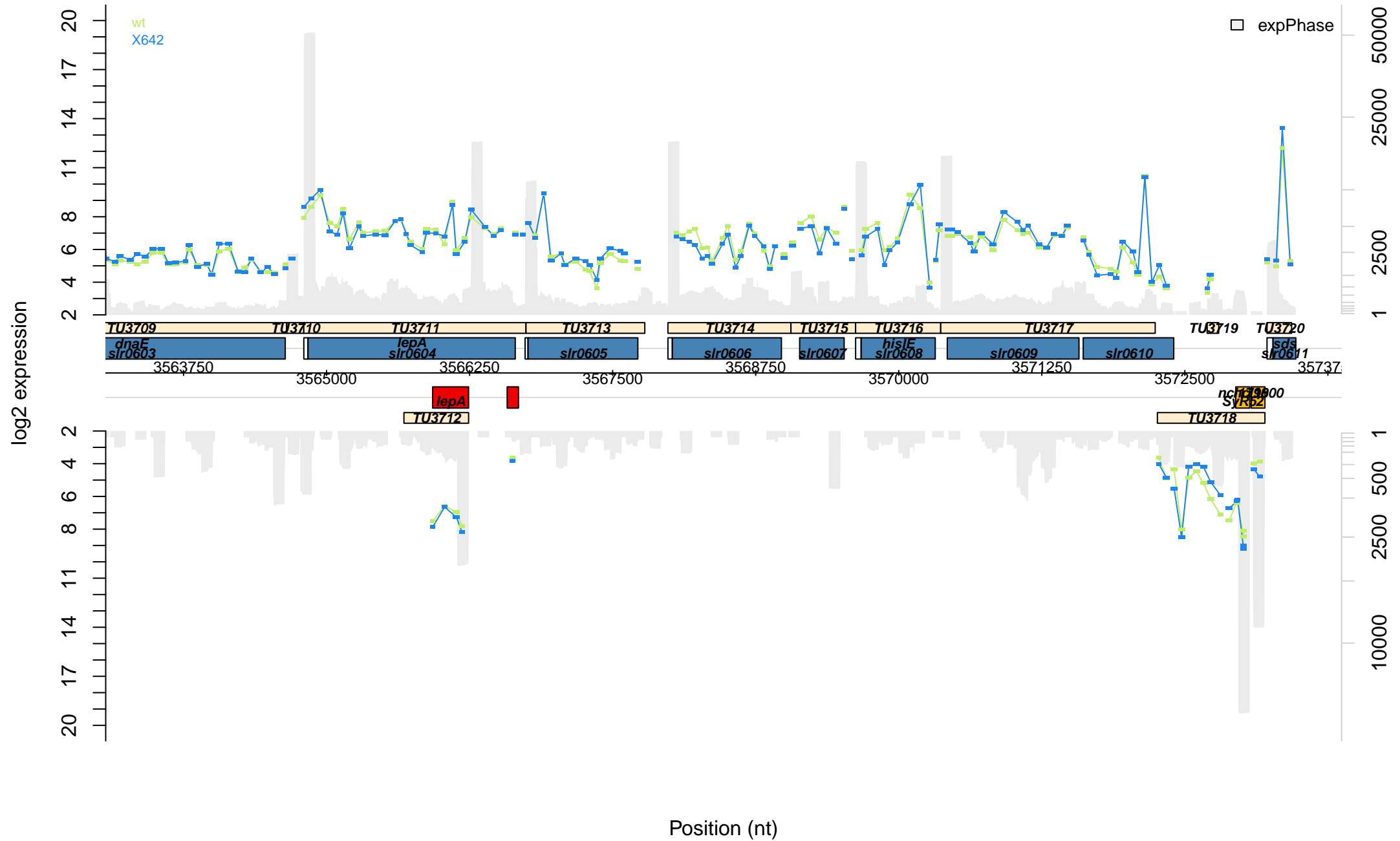

Supplement: Supplementary file 8 — 10.1186/s13068-016-0503-4 The genome plot shows the graphical overview of array signal intensities mapped along the Synechocystis chromosome. Both strands of the respective chromosomal regions are shown with the location of annotated protein-coding genes (blue boxes), antisense RNAs (red), and intergenic sRNA genes (yellow). The color-coded bars represent signals derived from individual microarray probes. The scale for the microarray signal intensities is given at the left y-axis. All probes for the same RNA feature are connected by lines. [file 13068_2016_503_MOESM8_ESM.pdf]

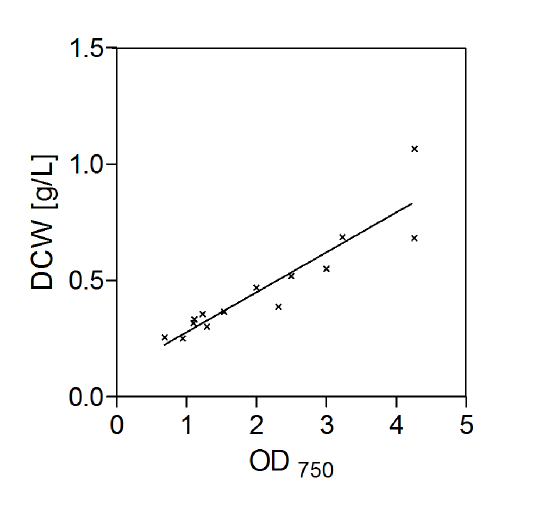

Supplement: Supplementary file 10 — 10.1186/s13068-016-0503-4 Determination of the relation OD750 to dry cell weight (DCW). With the help of this correlation, the calculation of our isoprene values to the dry weight basis was achieved. [file 13068_2016_503_MOESM10_ESM.docx]
