# Supplementary material for: Insights into isoprene production using the cyanobacterium Synechocystis sp. PCC 6803
Source: Biotechnol Biofuels. 2016 Apr 18;9:89. doi: 10.1186/s13068-016-0503-4 (PMC4836186; doi:10.1186/s13068-016-0503-4)
Supplement: Supplementary file 2 — 10.1186/s13068-016-0503-4 DNA and protein sequence of the codon-adapted cDNA of the ispS gene from Pueraria montana, which was used for the expression in Synechocystis sp. PCC 6803. Start and Stop codon of the gene sequence are highlighted. [file 13068_2016_503_MOESM2_ESM.docx]

**>*ispS*_codon-adapted**

ATGTGTGCTACCAGCTCTCAATTTACCCAAATTACCGAACATAATAGTCGGCGTTCCGCTAATTACCAGCCCAACCTCTGGAATTTTGAATTTCTGCAATCTCTGGAAAATGACCTTAAGGTGGAAAAATTAGAAGAGAAGGCCACCAAGTTAGAGGAGGAGGTACGGTGCATGATCAACCGGGTAGACACCCAACCCTTAAGCTTATTAGAATTGATCGACGATGTCCAGCGTTTAGGTTTGACCTACAAGTTTGAGAAGGACATTATCAAAGCCCTTGAGAATATTGTTTTGCTGGATGAGAATAAGAAAAATAAAAGTGACCTCCATGCTACTGCTCTCAGCTTCCGTTTACTTCGGCAACATGGCTTTGAGGTTTCCCAAGATGTGTTTGAGCGGTTTAAGGACAAGGAGGGTGGTTTCAGTGGTGAACTTAAAGGTGATGTGCAAGGGTTGCTGAGTTTATATGAAGCCTCCTATCTTGGCTTTGAGGGTGAAAATCTCTTGGAGGAGGCCCGGACCTTTTCCATTACCCATCTCAAGAACAACTTAAAAGAAGGTATTAACACCAAAGTGGCCGAACAAGTTAGTCATGCCCTGGAACTTCCCTATCATCAACGGTTGCATCGGTTAGAAGCCCGGTGGTTCCTTGACAAATATGAACCCAAGGAACCCCACCATCAGTTATTACTGGAGCTTGCCAAGTTAGATTTCAATATGGTGCAAACCTTGCACCAGAAAGAACTGCAAGACCTGTCCCGGTGGTGGACGGAGATGGGGTTAGCCAGCAAGTTAGACTTTGTCCGGGATCGGTTAATGGAAGTGTATTTTTGGGCGTTGGGTATGGCCCCTGATCCTCAATTCGGTGAATGTCGTAAAGCTGTCACTAAAATGTTTGGTTTGGTCACCATCATTGATGATGTATATGACGTTTATGGTACTTTGGATGAGTTACAACTCTTCACTGATGCTGTTGAGCGGTGGGACGTGAATGCCATTAACACCCTTCCCGACTACATGAAGTTGTGCTTCTTAGCCCTTTATAACACCGTCAATGACACGTCTTATAGCATCCTTAAAGAAAAAGGTCACAACAACCTTTCCTATTTGACCAAATCTTGGCGTGAGTTATGCAAAGCCTTCCTTCAAGAAGCCAAATGGTCCAACAACAAAATCATTCCCGCCTTTAGCAAGTACCTGGAAAATGCCTCCGTGTCCTCCTCCGGTGTGGCTTTGCTTGCTCCTTCCTACTTCTCCGTGTGCCAACAACAAGAAGATATCTCCGACCATGCTCTTCGTTCTTTAACTGATTTTCATGGCCTTGTGCGCTCCTCCTGCGTCATTTTCCGGCTCTGCAATGATTTGGCTACCTCCGCGGCTGAGTTAGAGCGGGGTGAGACGACCAATTCCATTATTTCTTATATGCACGAGAATGACGGCACTTCTGAAGAGCAAGCCCGTGAGGAGTTGCGGAAATTGATTGATGCCGAGTGGAAGAAGATGAACCGGGAGCGGGTTTCCGATTCTACCTTACTCCCCAAAGCTTTTATGGAAATTGCTGTTAACATGGCTCGGGTTTCCCATTGCACCTACCAATATGGTGACGGTCTTGGTCGGCCCGACTACGCCACCGAGAATCGGATCAAGTTGTTACTTATTGACCCCTTTCCCATCAATCAATTAATGTACGTGTAA

**>IspS_codon-adapted**

MCATSSQFTQITEHNSRRSANYQPNLWNFEFLQSLENDLKVEKLEEKATKLEEEVRCMIN

RVDTQPLSLLELIDDVQRLGLTYKFEKDIIKALENIVLLDENKKNKSDLHATALSFRLLR

QHGFEVSQDVFERFKDKEGGFSGELKGDVQGLLSLYEASYLGFEGENLLEEARTFSITHL

KNNLKEGINTKVAEQVSHALELPYHQRLHRLEARWFLDKYEPKEPHHQLLLELAKLDFNM

VQTLHQKELQDLSRWWTEMGLASKLDFVRDRLMEVYFWALGMAPDPQFGECRKAVTKMFG

LVTIIDDVYDVYGTLDELQLFTDAVERWDVNAINTLPDYMKLCFLALYNTVNDTSYSILK

EKGHNNLSYLTKSWRELCKAFLQEAKWSNNKIIPAFSKYLENASVSSSGVALLAPSYFSV

CQQQEDISDHALRSLTDFHGLVRSSCVIFRLCNDLATSAAELERGETTNSIISYMHENDG

TSEEQAREELRKLIDAEWKKMNRERVSDSTLLPKAFMEIAVNMARVSHCTYQYGDGLGRP

DYATENRIKLLLIDPFPINQLMYV*
