# Supplementary material for: Insights into isoprene production using the cyanobacterium Synechocystis sp. PCC 6803
Source: Biotechnol Biofuels. 2016 Apr 18;9:89. doi: 10.1186/s13068-016-0503-4 (PMC4836186; doi:10.1186/s13068-016-0503-4)
Supplement: Supplementary file 9 — 10.1186/s13068-016-0503-4 List of primers used in this study for RT-PCR and qPCR. [file 13068_2016_503_MOESM9_ESM.docx]

| **Primer** | **Sequence (5` → 3`)** |
| --- | --- |
|  |  |
| *ispS*_fw | GTTCCGCTAATTACCAGCCCAAC |
| *ispS*_rev | GCAACCCTTGCACATCAC |
